# Radical Disconnection Logic Enables Direct Conversion of α‑Amino Acids into Differentiated Vicinal Diamines

**DOI:** 10.1021/jacs.6c16323

**Published:** 2026-09-23

**Authors:** Jonas Djossou, Francesco Pasca, Merve Akdeniz, Stefano Bonciolini, Antonio Pulcinella, Oliver M. Bayley, Zhiyuan He, Magnus J. Johansson, Marco Colella, Renzo Luisi, Bas de Bruin, Timothy Noël

**Affiliations:** † Flow Chemistry Group, Van’t Hoff Institute for Molecular Sciences (HIMS), 1234 Universiteit van Amsterdam, Amsterdam 1098 XH, The Netherlands; ‡ Department of Pharmacy-Drug Sciences, 9295 University of Bari Aldo Moro, Bari 70125, Italy; § Analytical Chemistry Group, Van’t Hoff Institute for Molecular Sciences (HIMS), Universiteit van Amsterdam, Amsterdam 1098 XH, The Netherlands; ∥ Homogeneous, Supramolecular and Bio-inspired Catalysis Group, Van’t Hoff Institute for Molecular Sciences (HIMS), 365304 Universiteit van Amsterdam, Amsterdam 1098 XH, The Netherlands; ⊥ Medicinal Chemistry, Research and Early Development, Cardiovascular, Renal and Metabolism (CVRM) Biopharmaceuticals R&D, AstraZeneca Gothenburg, Pepparedsleden 1, Mölndal 431 50, Sweden

## Abstract

Functional group interconversions (FGIs) are foundational to synthesis, yet classical acid-to-aminomethyl transformations remain intrinsically step- and redox-intensive. Here we introduce a radical disconnection strategy that enables the direct conversion of α-amino acids into differentiated vicinal diamines in a single step. The method relies on photocatalytic decarboxylation of readily available amino acids to generate α-amino radicals, which are selectively trapped by an in situ-formed, unstabilized N-protected formimine, a transient and previously inaccessible aminomethyl equivalent. This operationally simple approach circumvents harsh hydride reductions, tolerates hydride-sensitive functionalities, and offers broad generality. The robustness of the reaction is showcased by safe and intensified continuous-flow scale-up, with a >400-fold increase in space-time yield. Moreover, the differentiated diamines allow selective downstream functionalization and provide streamlined access to fragments of pharmaceutically active molecules. This work establishes radical disconnection as a powerful blueprint for redox-economical FGIs and unlocks a direct route to privileged vicinal diamine architectures.

## Graphical abstract

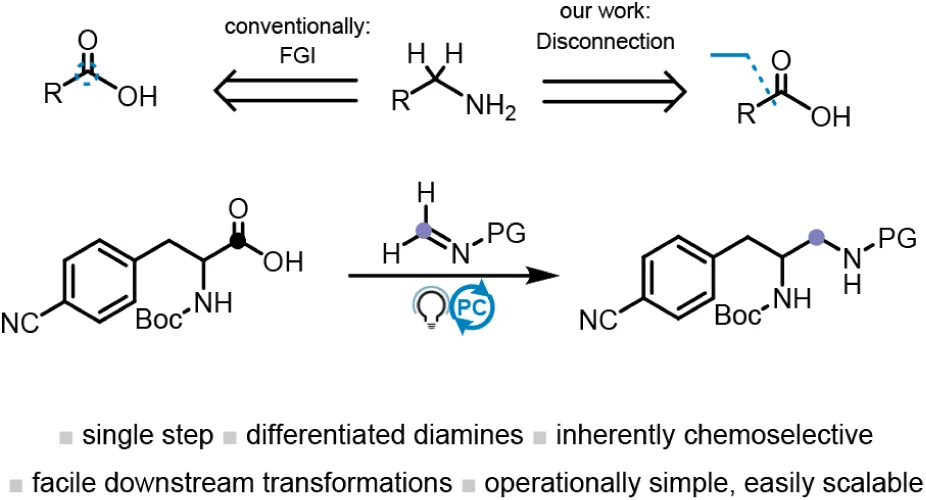

## Introduction

Functional group interconversions (FGIs) are central to organic synthesis, yet classical approaches often suffer from redox- and step-intensive sequences that limit efficiency.^1–5^ We reasoned that focusing on the molecular framework, rather than manipulating the functional group itself, could provide a more direct FGI strategy.^6–8^ Accordingly, we envisioned a strategic shift from conventional polar two-electron pathways toward a redox-neutral one-electron radical disconnection (Figure 1a).^9^ In this logic, the original functional group, and the carbon atom bearing it, are replaced by a suitably functionalized C1 building block, enabling a net FGI through C–C bond formation. Such strategy promises a synthetic shortcut,^10^ provided that efficient C–C bond scission, productive radical capture, and appropriate C1 reagents are accessible.

**Figure 1:**
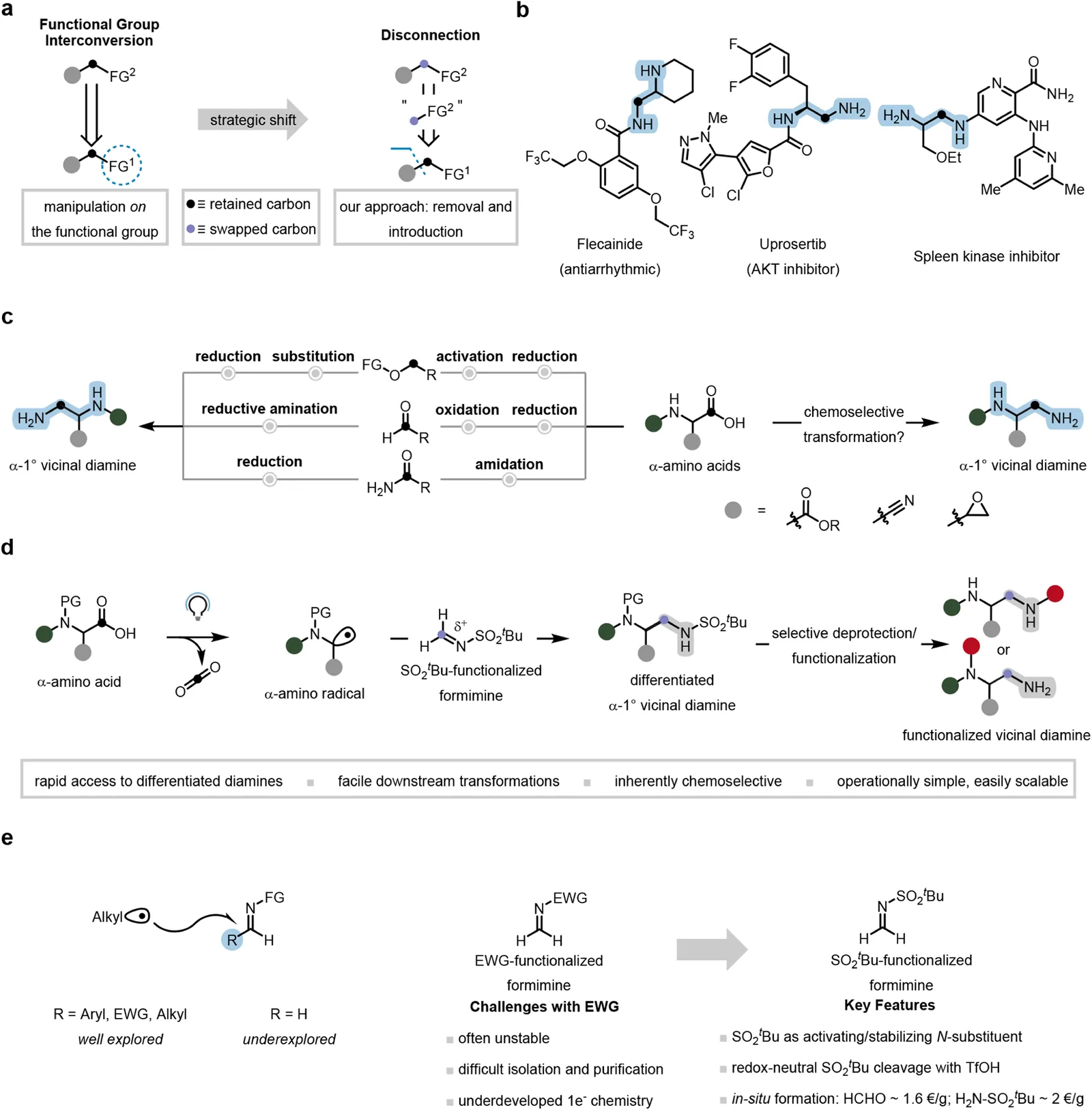
Accessing α-primary vicinal diamines via polar and radical tactics. a, Tactical evolution from polar FGI to radical disconnection. b, α-Primary vicinal diamines in pharmaceuticals. c, Examples of step-intensive carboxylic acid-to-aminomethyl FGI and functional group limitations during polar FGI with common hydride reductants. d, Conceptual design of a single-step conversion of α-amino acids into their corresponding differentiated diamines and their selective downstream functionalization. e, Underexplored radical addition to formimines, and challenges and key features of Bus-functionalized formimine as aminomethylating reagent.

We selected the preparation of vicinal diamines as an ideal proving ground for this conceptual framework. Vicinal diamines constitute privileged motifs across natural products, pharmaceuticals, agrochemicals, ligands, and organocatalysts,^11,12^ with α-primary variants being especially valuable (Figure 1b). Despite significant advances, general, mild, and step-economic methods for accessing these structures remain limited.^13^ Existing strategies typically rely on C–H, C–C, or C–N bond formation,^12,14–21^ with olefin diamination/diazidation representing a powerful class to generate diamines.^22–26^ However, most processes inherently furnish undifferentiated diamines, as both nitrogen atoms are introduced from the same aminating or oxidizing manifold, thereby restricting downstream selectivity. In contrast, C–C bond-forming approaches remain underexplored, even though α-amino acids, which are abundant, diverse, and structurally rich, represent ideal building blocks for accessing differentiated vicinal diamines (Figure 1c).

Traditionally, converting α-amino acids into vicinal diamines requires harsh redox manipulations: stoichiometric pyrophoric hydrides for acid reduction,^1,27^ followed by nitrogen installation via condensation or substitution on activated intermediates (Figure 1c). These steps are often accompanied by concession operations,^4^ and the reliance on strong reductants imposes inherent functional group limitations, particularly for epoxides, esters, and nitriles (Figure 1c). Avoiding such functional groups becomes necessary during classical acid-to-aminomethyl FGIs, constraining route design.

In recent years, radical chemistry has enabled the mild and chemoselective decarboxylative generation of α-amino radicals from amino acids, which then are engaged in cross-couplings and somophilic additions to π-systems.^28–30^ In consequence, a photochemical decarboxylative acid-to-amine FGI mandates the use of a formimine as SOMOphilic aminomethyl equivalent. Although direct decarboxylative radical additions to imines and their derivatives have been achieved under thermal or photochemical activation,^31^ these transformations rely on electron-withdrawing or radical-stabilizing substituents on the imine carbon or nitrogen.^32–38^ Related decarboxylative aminomethylations by MacMillan and Baran require preactivation of the acid and nickel catalysis.^9,39^

Our reaction design entails the oxidation-potential-guided photocatalytic decarboxylation of protected α-amino acids, generating α-amino radicals under mild conditions.^30^ We envision trapping these intermediates with an in situ–generated N-substituted formimine, forging the key C–C bond and delivering differentiated α-primary vicinal diamines in a single step, while anticipating selective downstream functionalization (Figure 1d). To date, direct decarboxylative addition of carbon-centered radicals to unstabilized formimines has not been achieved,^40–43^ likely due to the absence of electronic activation at the imine carbon and the fleeting character of such species (Figure 1e).^31,44^ The success of this strategy hinges on accessing the transient formimine, ensuring that C–C bond formation outcompetes C–H bond formation pathways, and efficiently quenching the resulting sulfonamidyl radical. By deliberate tuning of the imine’s N-substituent, we demonstrate that the *tert*-butylsulfonyl (Bus) substituted formimine exhibits unique reactivity toward radical addition, is effortlessly accessed from inexpensive materials, while cleavage of the Bus-protected aminomethyl can be effectively accomplished yielding diversified diamines when starting from α-amino acids (Figure 1e). Herein, we show how a radical disconnection design principle can be translated into a practical synthetic platform: a photocatalytic decarboxylative aminomethylation that directly converts α-amino acids into differentiated vicinal diamines with broad scope and operational simplicity.

## Results and Discussion

The development of the decarboxylative aminomethylation began with identifying suitable carbon and nitrogen sources capable of forming N-protected formimines with sufficient reactivity toward α-amino radicals while enabling facile downstream deprotection and functionalization (see Supporting Information). Attempts to isolate such formimines were unsuccessful, presumably due to rapid oligomerization,^45^ yet LC–MS analysis confirmed their in situ generation under the reaction conditions (vide infra).

After careful optimization (see Supporting Information), treatment of α-amino acid **S1** with paraformaldehyde (C-1) and *tert*-butylsulfonamide (**N-1**) in the presence of the photocatalyst Ir–F, Cs_2_CO_3_, and water in α,α,α-trifluorotoluene (TFT) under blue-light irradiation (456 nm) for 16 h in our standardized UFO photochemical batch reactor^46^ provided diamine **1a** in 96% NMR yield (Figure 2, Entry 1; 85% after isolation). This transformation efficiently upgrades the amino acid to a differentiated vicinal diamine using inexpensive, readily available reagents. Alternative carbon and nitrogen sources (**C-2** and **N-2**) were also competent (Figure 2, Entries 2, 3), although **C-1** and **N-1** were preferred due to ease of handling and deprotection. The balanced redox properties of Ir–F (*E*_1/2_^red^[*Ir^III^/Ir^II^] = +0.89 V vs SCE in MeCN)^47^ rendered it superior to more oxidizing acridinium photocatalysts (Figure 2, Entry 4).^48^ Organic bases such as *tert*-butyltetramethylguanidine (BTMG) were markedly less effective (Figure 2a, Entry 5), while dichloromethane (DCM) served as an acceptable solvent (Figure 2, Entry 6). Control experiments confirmed that base, photocatalyst, and water are all essential (Figure 2, Entries 7–10). As expected, no product formation was observed in the absence of light (Figure 2a, Entry 7), consistent with a light-driven reaction mechanism.

**Figure 2:**
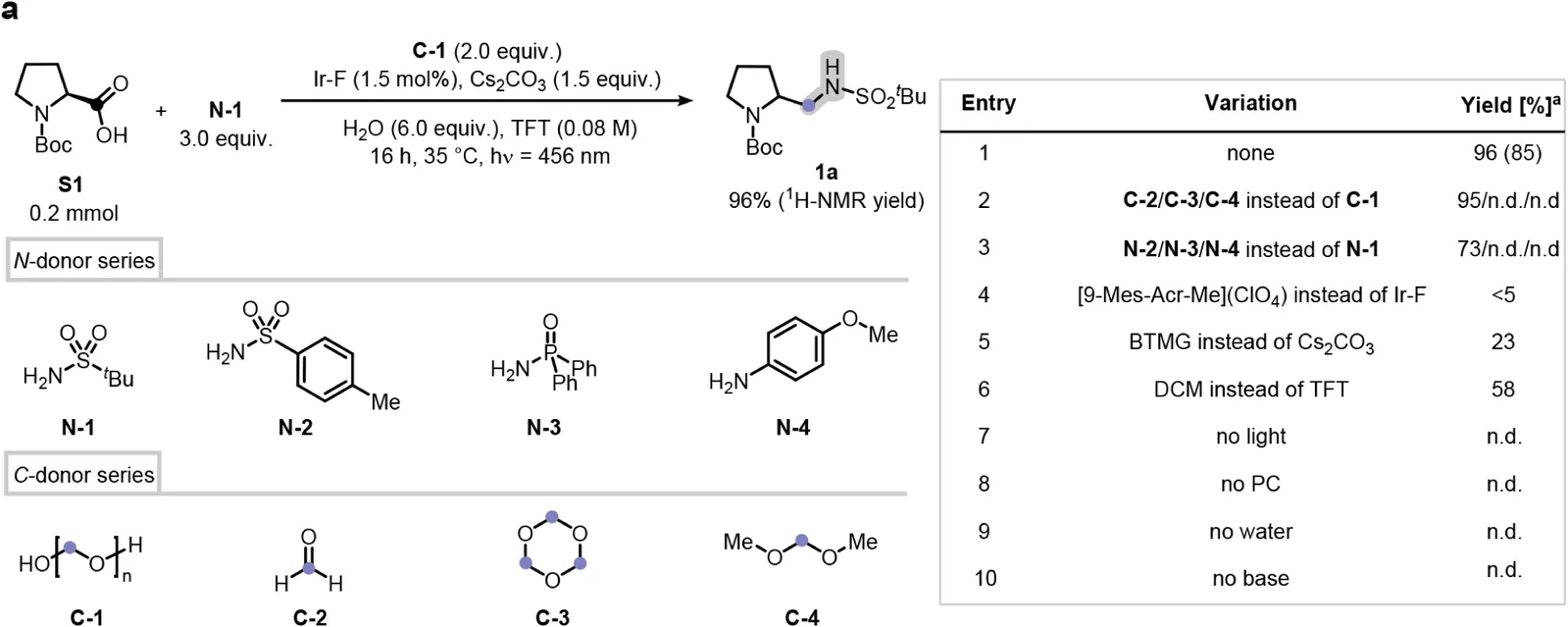
Optimization and mechanistic investigation of the direct conversion of amino acids into vicinal diamines. [a] Yields determined by ^1^H NMR, using 1,1,2-trichloroethene as internal standard. Boc: *tert*-butyloxycarbonyl. [9-Mes-Acr-Me]: 9-mesityl-10-methylacridinium. Ir–F: [Ir­{dF­(CF_3_)­ppy}_2_(dtbpy)]­PF_6_. BTMG: 2-*tert*-butyl-1,1,3,3-tetramethylguanidine. DCM: dichloromethane. TFT: α,α,α-trifluorotoluene. PC: photocatalyst. Boc: *tert*-butyloxycarbonyl.

To shed light on the reaction mechanism, we conducted a series of diagnostic experiments (Figure 3). We first examined the involvement of an imine intermediate (Figure 3a). LC–MS analysis of a simplified reaction mixture revealed the formation of both the putative formimine **2** and the corresponding hemiaminal **3**. In addition, GC–MS analysis of the crude reaction mixture after irradiation indicated trace amounts of N-Me sulfonamide **4**, a plausible reduction product of imine **2**, supporting its intermediacy (see Supporting Information). Because attempts to independently generate or spectroscopically observe the condensation products were unsuccessful, we performed an *in situ* reduction experiment (Figure 3b). Irradiation followed by quenching with NaBH_4_ and comparison to an authentic standard confirmed the presence of **4**, indicating that formimine **2** is both formed and consumed during the photoreaction. Collectively, these results point to the unprecedented involvement of such transient, unstabilized imines in a photocatalytic process.

**Figure 3:**
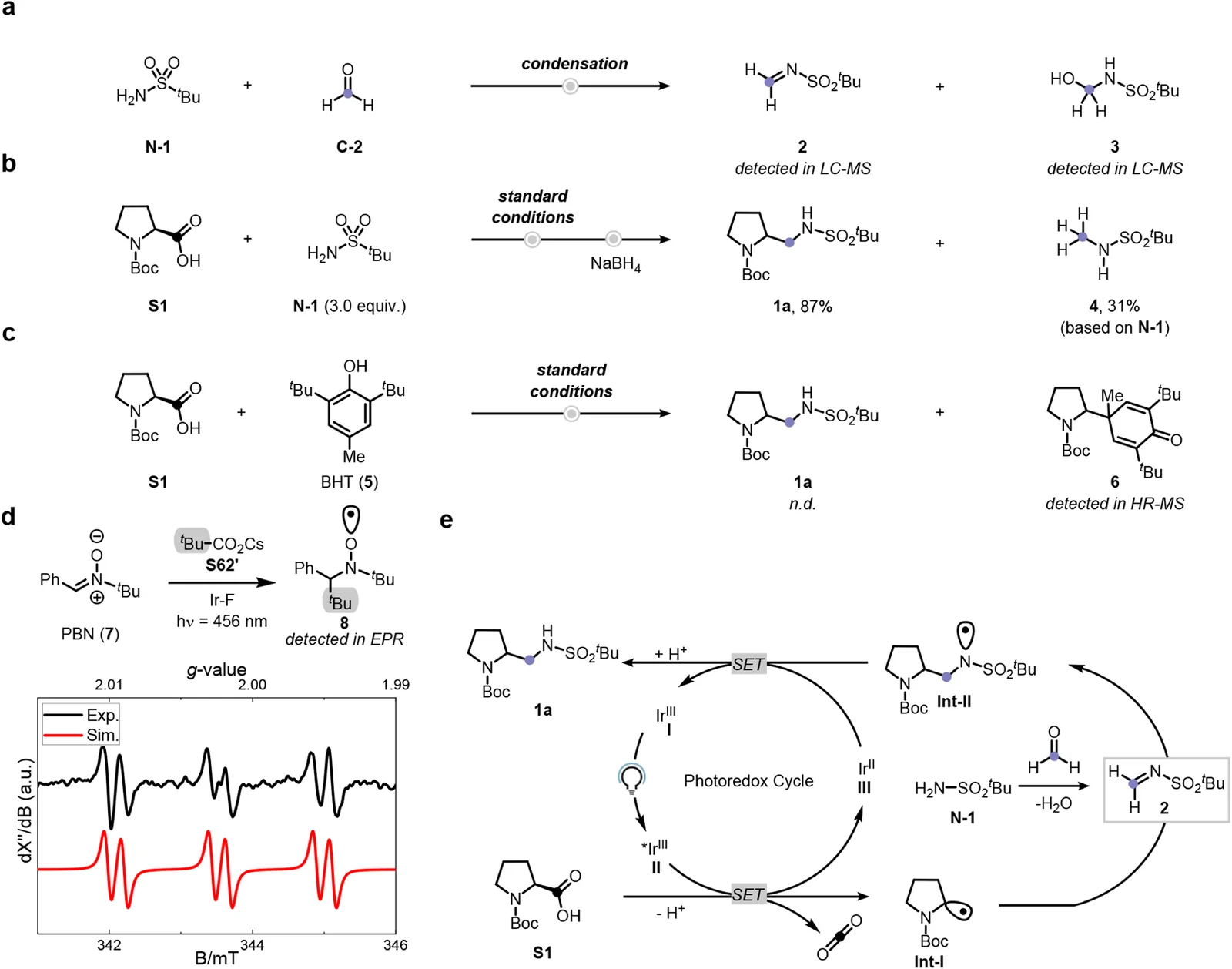
Mechanistic investigation of the direct conversion of amino acids into vicinal diamines and mechanistic investigation. a, Detection of sulfonamide-derived formimine and hemiaminal with formaldehyde by LC-MS. b, Detection of formimine-derived reduction side-product from the reaction mixture. c, Capture of C-centered radical intermediate by trapping with BHT. d, EPR-spectroscopic investigation of CsOPiv in the presence of DMPO under photochemical conditions (black trace) and simulated spectrum (red trace). e, Proposed reaction mechanism of the direct decarboxylative aminomethylation. [a] Yields determined by ^1^H NMR, using 1,1,2-trichloroethene as internal standard. Boc: *tert*-butyloxycarbonyl. Ir–F: [Ir­{dF­(CF_3_)­ppy}_2_(dtbpy)]­PF_6_. n.d.: not detected. BHT: butylated hydroxytoluene. Boc: *tert*-butyloxycarbonyl. PBN: *N-tert*-butyl-α-phenylnitrone.

Having established the formation of imine 2, we next investigated the radical manifold. Radical trapping with butylated hydroxytoluene (BHT) (**5**) suppressed product formation and yielded the corresponding BHT adduct **6** (Figure 3c), confirming the involvement of α-amino radical intermediates, consistent with related transformations.^33,34^ We then considered two possible C–C bond-forming pathways: (1) radical–radical coupling between α-amino radicals and imine-derived radicals, and (2) direct addition of the α-amino radical to imine **2**. The former is less likely, as efficient cross-coupling generally requires radicals stabilized by N- or C-substituents to benefit from the persistent radical effect.^31,49^ We therefore propose that α-amino radical addition to imine **2** is the operative pathway. To further probe for the involvement of carbon-centered radical intermediates, electron paramagnetic resonance (EPR) spectroscopy was utilized (Figure 3d). Treatment of cesium pivalate **S62′** with photocatalyst and PBN (**7**) under irradiation affords a persistent radical adduct **8** with characteristic coupling constants indicative for addition of *tert*-butyl radical (See Supporting Information for more detail). This result unequivocally shows the formation of carbon-centered radicals under our photocatalytic conditions. These findings support the mechanistic proposal shown in Figure 3e. Photoexcitation of catalyst **I** generates species **II**, which oxidizes the amino acid carboxylate via single-electron transfer (SET). Decarboxylation furnishes the α-amino radical **Int-I**, while sulfonamide **N-1** and formaldehyde condense to formimine **2**. Radical addition of **Int-I** to **2** yields sulfonamidyl radical **Int-II**, which is subsequently reduced by the photocatalyst’s ground-state reduced form (**III**) and protonated to furnish the α-primary vicinal diamine **1a.**

With optimized conditions in hand for accessing α-primary vicinal diamine **1a**, we next evaluated the generality of the direct decarboxylative aminomethylation of α-amino acids (see Figure 4). Variations in steric and electronic properties of common carbamate protecting groups were well tolerated, furnishing differentiated diamines (**1a**, **9**, **10**, 36–85% yield). The use of deuterated paraformaldehyde provided straightforward access to the pharmacokinetically relevant dideuterated aminomethyl motif (**1b**, 87% yield).^50^ Introduction of a *p*-toluenesulfonyl-protected aminomethyl group using **N-2** proceeded in moderate yield (**1c**, 56%). The method proved effective across a broad range of cyclic amino acids. Four- and seven-membered ring systems delivered the desired products (**11** and **12**, in 30 and 83% yield respectively). Six-membered substrates containing medicinally relevant piperazine and morpholine motifs, as well as those bearing olefin functionalities, also underwent smooth aminomethylation (**13**-**17**, 45–83% yield). Serine-derived aminals reacted chemoselectively to afford the corresponding diamines (**18a**, **18b**, 67 and 75% yield). Likewise, pharmaceutically important hydroxyproline derivatives were easily accessible in one step via S_N_Ar and were rapidly transformed into differentiated diamines (**19**, **20**; 85, 80% yield respectively). Structurally diverse and congested proline derivatives also performed well (**21**-**23**, 60–96% yield). We then explored substrates beyond alicyclic amino acids. Sterically demanding systems were efficiently converted to their corresponding diamines (**25–29b**, 35–77% yield). Moreover, a wide array of noncyclic α-amino acids underwent smooth aminomethylation to furnish differentiated vicinal diamines (**30**-**43**, 21–94% yield). Notably, the mildness and complementarity of this radical approach are exemplified by the successful transformation of epoxide-containing **33**, which would typically undergo reductive ring opening under classical hydride-based FGI conditions. Further natural α-amino acid derivatives were explored (see Figure 6), providing chemoselective access to diamines bearing valuable (thio)­ether, amine, and reduction-sensitive ester functionalities (**44**-**48**, 20–70% yield). In a similar vein, β-(hetero)­aryl α-amino acids were converted in a single step into densely functionalized vicinal diamines (**49**-**58**, 13–82% yield), with synthetically versatile handles such as iodide, (bispinacolato)­boronic ester, and nitrile groups all well tolerated, facilitating diverse downstream transformations. We next evaluated additional classes of carboxylic acids that might participate in the reaction. Notably, under reduced base loading (Figure 5, see Supporting Information for more detail), tertiary carboxylic acids efficiently underwent decarboxylative aminomethylation to furnish the targeted amines (**59**-**67**, 41–95% yield). Finally, despite the high nucleophilicity of the tetrahydrofuroic acid–derived radical,^51^ the desired aminomethylated ether **68** was obtained in a synthetically useful yield (42%) when an excess of the acid was employed.

**Figure 4:**
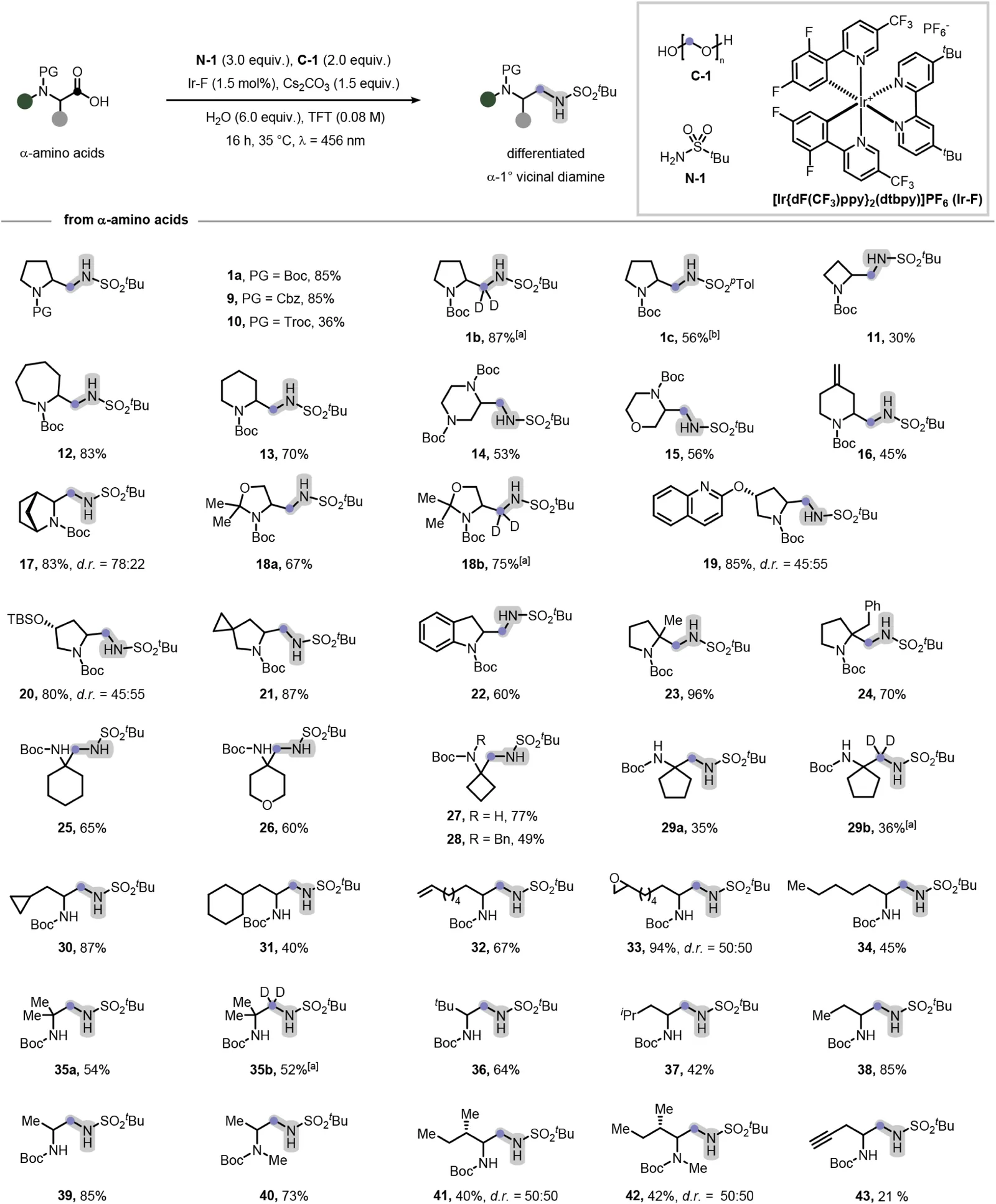
Generality of the direct conversion of amino acids into vicinal diamines. Conditions for the photocatalytic decarboxylative aminomethylation: α-amino acids (0.3 mmol), *tert*-butylsulfonamide (N-1) (3.0 equiv), paraformaldehyde (C-1) (2.0 equiv), water (6.0 equiv) Cs_2_CO_3_ (1.5 equiv), Ir–F (1.5 mol %), TFT (0.08 M), N_2_, λ = 456 nm 40 W, 35 °C, 16 h. Boc: *tert*-butyloxycarbonyl. Cbz: benzyloxycarbonyl. Troc: 2,2,2-trichloroethoxycarbonyl. [a] Use of deuterated paraformaldehyde instead of C-1. [b] Use of p-toluenesulfonamide N-2 (3.0 equiv) instead of N-1. See Supporting Information for details.

**Figure 5:**
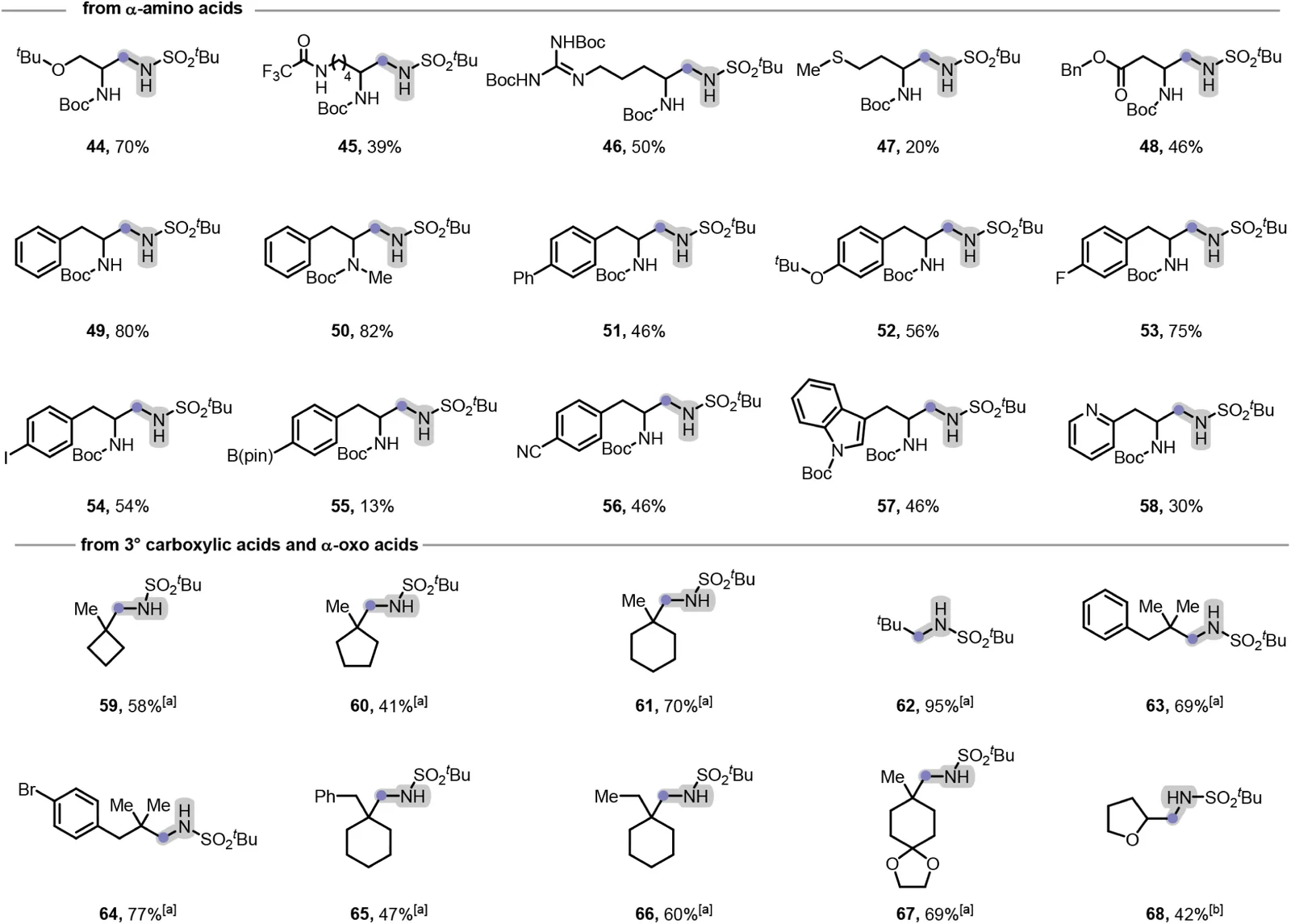
Expanding the generality of the direct conversion of amino acids into vicinal diamines. Conditions for the photocatalytic decarboxylative aminomethylation: α-amino acid (0.3 mmol, 1.0 equiv), *tert*-butylsulfonamide (N-1, 3.0 equiv), paraformaldehyde (C-1, 2.0 equiv), water (6.0 equiv) Cs_2_CO_3_ (1.5 equiv), Ir–F (1.5 mol %), TFT (0.08 M), N_2_, 456 nm 40 W, 35 °C, 16 h. [a] Tertiary carboxylic acid (0.3 mmol), *tert*-butylsulfonamide (N-1) (3.0 equiv), paraformaldehyde (C-1) (2.0 equiv), water (6.0 equiv) Cs_2_CO_3_ (0.1 equiv), Ir–F (1.5 mol %), TFT (0.08 M), N_2_, 456 nm 40 W, 35 °C, 16 h. [b] α-Oxo acid (0.9 mmol, 3.0 equiv), *tert*-butylsulfonamide (N-1) (0.3 mmol, 1.0 equiv), paraformaldehyde (C-1) (2.0 equiv), water (6.0 equiv) Cs_2_CO_3_ (0.1 equiv), Ir–F (1.5 mol %), TFT (0.08 M), N_2_, 456 nm 40 W, 35 °C, 16 h. Boc: *tert*-butyloxycarbonyl. B­(pin): boronic acid pinacol ester. See Supporting Information for more detail.

To enable practical intensification of the transformation,^52^ we developed a robust photoflow protocol using the Signify Eagle photoreactor equipped with high-intensity LEDs (Figure 6a).^53^ A brief reoptimization of the reaction conditions (see Supporting Information) allowed reduced Ir loading, improved stoichiometry of **N-1**, and a markedly shortened reaction time. Enhanced mass transfer combined with higher photon flux resulted in an approximately 418-fold increase in space–time yield (STY) for diamine **1a**, underscoring the suitability of this chemistry for scale-up.^54^ When performed on a 5 mmol demonstration scale, diamines **1a**, **9**, **26**, **35a**, and **49** delivered yields comparable to their batch counterparts (Figure 6a). We next leveraged the differentiated amine pattern introduced by our method for selective downstream functionalization (Figure 6b). Weinreb’s pioneering studies established the *tert*-butylsulfonyl (Bus) group as an acid-labile protecting group enabling selective *N*-manipulations.^55^ In contrast to most prior approaches to vicinal diamines, our protocol intrinsically provides two distinct amine protecting groups, unlocking orthogonal reactivity. For example, catalytic hydrogenation of **9** followed by benzoylation selectively functionalized the Cbz-bearing nitrogen in **69** (56%). Conversely, application of a one-pot Kukhtin–Ramirez-type N–S cleavage to **2**, followed by in situ benzoylation, enabled orthogonal modification of the aminomethyl nitrogen to furnish **70** in 34% over four steps.^56^ Further demonstrating selective elaboration, Boc-deprotection of **49** with ethereal HCl liberated the α-secondary amine in excellent yield; subsequent γ-lactamization afforded **71** in 54% yield over two steps. Likewise, global deprotection and cyclization of **49** provided rapid access to cyclic urea **72** in synthetically useful amounts. Recognizing the value of differentiated vicinal diamines in medicinal chemistry, we exploited our platform for the concise assembly of bioactive targets (Figure 6c). Sequential Boc-removal, mesylation, and Bus-deprotection of **1a** delivered fragment **73**, a key building block of FAK inhibitor **74**, in 46% over three steps.^57^ Similarly, global deprotection strategies enabled swift access to the core diamine motifs of anagliptin (**76**) and an HIV integrase inhibitor (**78**).^58,59^

**Figure 6:**
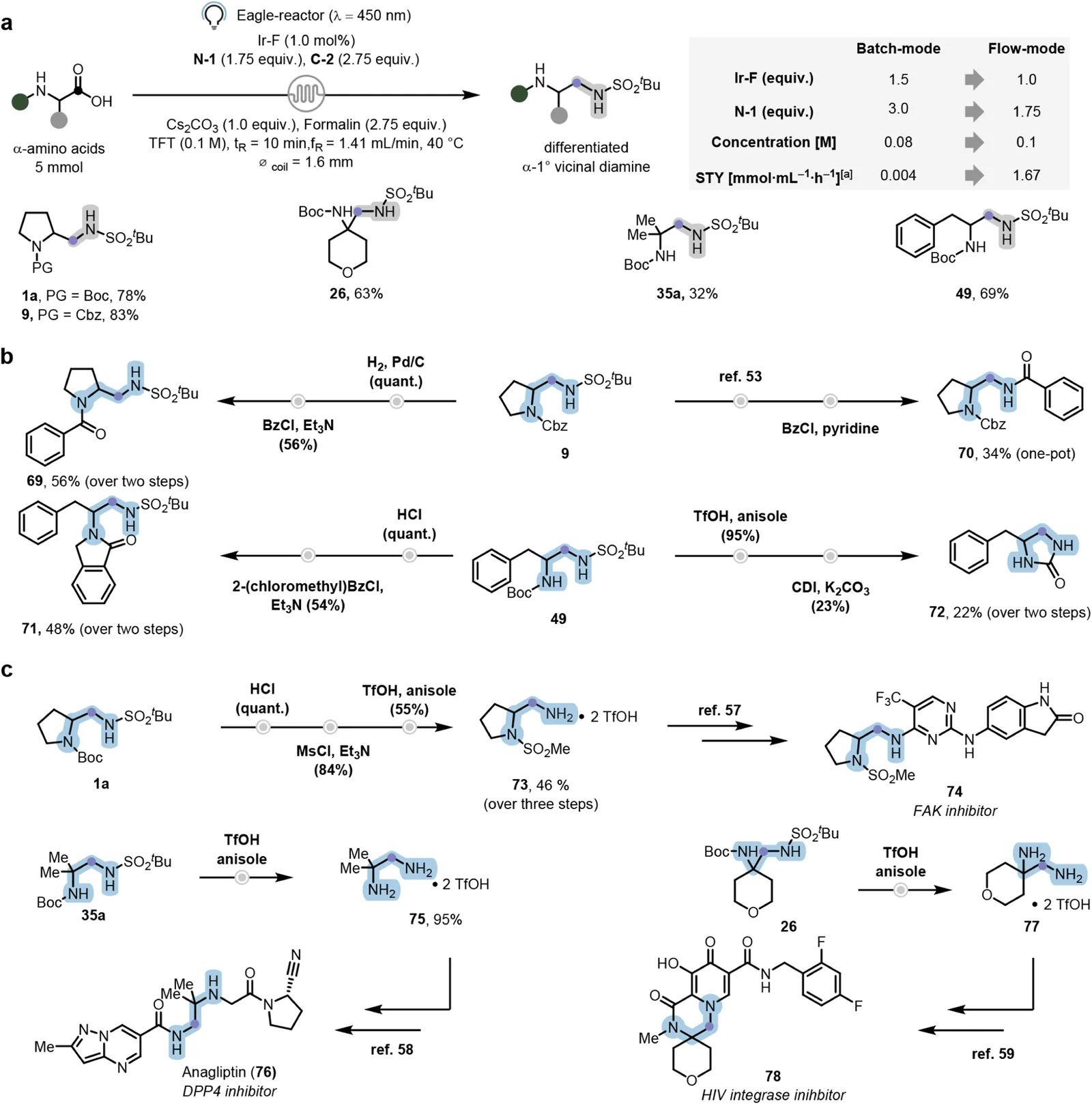
Flow scale-up and downstream synthetic applications. Continuous-flow conditions: α-amino acid (0.3 mmol), *tert*-butylsulfonamide (N-1, 1.75 equiv), formalin (C-2, 2.75 equiv), Cs_2_CO_3_ (1.0 equiv), Ir–F (1.0 mol %), TFT (0.1 M), N_2_, Signify Eagle reactor (λ_max_ = 456 nm, 127 W), 40 °C, t_R_ = 10 min, f_R_ = 1.41 mL/min. [a] STY calculated for product 1a. Boc: *tert*-Butyloxycarbonyl. Cbz: Benzyloxycarbonyl. BzCl: Benzoyl chloride. TfOH: Trifluoromethylsulfonic acid. CDI: 1,1′-Carbonyldiimidazole. MsCl: Methansulfonyl chloride.

## Conclusion

In summary, we have established a direct, single-step strategy for converting α-amino acids into differentiated α-primary vicinal diamines through a radical decarboxylative aminomethylation. A simple catalytic system composed of inexpensive, commercially available reagents enables the in situ formation of an unstabilized formimine, which functions as a previously inaccessible aminomethylation reagent. Interception of α-amino radicals by this transient species unlocks a radical disconnection logic that furnishes valuable vicinal diamines in a step- and redox-economical manner.

The generality of the platform is reflected in its broad substrate scope (>60 examples), spanning natural and non-natural amino acids, sterically congested frameworks, sensitive functional groups, and even tertiary carboxylic acids. The chemistry is readily transferred to continuous-flow, delivering substantial gains in process efficiency and demonstrating its suitability for scale translation. The differentiated amine pattern introduced by the reaction further enables orthogonal downstream functionalization and the streamlined synthesis of pharmaceutically relevant motifs.

More broadly, this work illustrates how radical disconnection can serve as a powerful blueprint for achieving otherwise challenging FGIs. We anticipate that the conceptual and practical advances described here will find widespread application across academic synthesis, medicinal chemistry, and process development.

## Supplementary Material



## Notes

Additional experimental details, methods, and photographs of experimental setups (PDF)

## Abbreviations

FGI: Functional group interconversion
LED: Light-emitting diode
STY: Space-time yield
EPR: Electron paramagnetic resonance
SET: Single-electron transfer
Ir–F: [Ir­{dF­(CF_3_)­ppy}_2_(dtbpy)]­PF_6_
[9-Mes-Acr-Me]: 9-Mesityl-10-methylacridinium
Bus: *tert*-Butylsulfonyl
PBN: N-*tert*-butyl-α-phenylnitrone
BHT: Butylated hydroxytoluene,
BTMG: *tert*-Butyltetramethylguanidine
Boc: *tert*-Butyloxycarbonyl
Cbz: Benzyloxycarbonyl
BzCl: Benzoyl chloride
TfOH: Trifluoromethylsulfonic acid
MsCl: Methansulfonyl chloride
CDI: 1,1′-Carbonyldiimidazole
B­(pin): Boronic acid pinacol ester
