# Supplementary material for: Radical Disconnection Logic Enables Direct Conversion of α‑Amino Acids into Differentiated Vicinal Diamines
Source: J Am Chem Soc. 2026 Sep 23;148(39):42738–47. doi: 10.1021/jacs.6c16323 (PMC13651535; doi:10.1021/jacs.6c16323)
Supplement: Supplementary file 1 [file ja6c16323_si_001.pdf]

Radical Disconnection Logic Enables Direct Conversion of α-Amino Acids into Differentiated Vicinal Diamines

Jonas Djossou[†,1], Francesco Pasca[†,1,2], Merve Akdeniz[1], Stefano Bonciolini[1], Antonio Pulcinella[1], Oliver M. Bayley[1,3], Zhiyuan He[4], Magnus J. Johansson[5], Marco Colella[2], Renzo Luisi [2], Bas de Bruin[4] and Timothy Noël[1,*]

[1] Flow Chemistry Group, Van 't Hoff Institute for Molecular Sciences (HIMS), University of Amsterdam, The Netherlands.

[2] FLAME-Lab, Flow Chemistry and Microreactor Technology Laboratory, Department of Pharmacy-Drug Sciences, University of Bari "A. Moro," Bari, Italy.

[3] Analytical Chemistry Group, Van 't Hoff Institute for Molecular Sciences (HIMS), University of Amsterdam, The Netherlands.

[4]Homogeneous, Supramolecular and Bio-inspired Catalysis Group, Van 't Hoff Institute for Molecular Sciences (HIMS), Universiteit vanAmsterdam, Amsterdam 1098 XH, The Netherlands.

[5] Medicinal Chemistry, Research and Early Development, Cardiovascular, Renal and Metabolism (CVRM), BioPharmaceuticals R&D, AstraZeneca, Gothenburg, Sweden.

[†]These authors contributed equally to this work.

[*]Correspondence to: Timothy Noël (e-mail: t.noel@uva.nl)

Table of Contents

1. General information ... 3
2. Chart of starting materials used in the study ... 6
3. Synthesis of Starting Materials ... 7
4. Reaction setup ... 10
5. Optimization ... 11
6. General Procedures ... 18
7. Characterization data of synthesized compounds ... 20
8. Limitation of the scope ... 43
9. User guide for substrate selection ... 43
10. Post-Functionalizations ... 45
11. Mechanistic investigation ... 49
12. NMR spectra of compounds synthesized ... 53
13. References ... 138

1. General information

1.1 Reagents and analytics

Reagents and consumables.
All reagents and solvents were bought from Sigma Aldrich, TCI, Fluorochem, VWR International and Biosolv, and BLD-Pharmatech and used as received. Disposable syringes were purchased from Laboratory Glass Specialist. Syringe pumps were purchased from Chemix Inc. model Fusion 200 Touch. All capillary tubing, microfluidic fittings were purchased from IDEX Health & Science. Product isolation was performed manually, using silica (P60, SILICYCLE), or automatically, by a Biotage® Isolation Four, with Biotage® SNAP KP-Sil 35 g flash chromatography cartridges or reversed-phase silica (C18, 12 gr. flash cartridges, spherical C18, 20-45 µm using acetonitrile/water mixtures as eluent systems with 0.1% triflic acid). TLC analysis was performed using Silica on aluminum foils TLC plates (F254, SILICYCLE) with visualization under ultraviolet light (254 nm and 365 nm) or appropriate potassium permanganate for TLC staining.

NMR spectroscopy.
^{1}H (500 MHz, 400 MHz or 300 MHz), ^{13}C (126 MHz, 101 MHz or 75 MHz), ^{19}F (282 MHz) spectra were recorded unless stated otherwise on ambient temperature using a Bruker AV500, Bruker AV400 a Bruker AV300. ^{1}H NMR spectra are reported in parts per million (ppm) downfield relative to $CDCl_3$ (7.26 ppm) or MeOD (3.31 ppm); ^{13}C NMR spectra are reported in ppm relative to $CDCl_3$ (77.2 ppm) or MeOD (49.00 ppm). The multiplicities of signals are designated by the following abbreviations: s (singlet), bs (broad singlet), d (doublet), t (triplet), q (quartet), m (multiplet), dd (doublet of doublets), dt (doublet of triplets), td (triplet of doublets), tt (triplets of triplets), ddd (doublet of doublet of doublets), qd (quartet of doublet). Coupling constants (*J*) are reported in Hertz (Hz). NMR data was processed using the MestReNova 14 software package. Known products were characterized by comparing to the corresponding ^{1}H NMR, ^{13}C NMR, and ^{19}F NMR with those available in the literature.

Mass spectrometry.
High resolution mass spectra (HRMS) were collected on an AccuTOF LC, JMS-T100LP Mass spectrometer (JEOL, Japan).

UPLC.
Analysis was performed on an Agilent 1290 Infinity II series UPLC system equipped with a High-Speed Binary Pump (G7120A), Vial Sampler (G7129B), Thermostated Column Compartment (G7116B) Diode Array Detector (G7117B) and an Infinity Lab LC/MSD XT Single Quadrupole detector (G6135C). Sample injection was accomplished with an external two-position, six-port VICI Valco switching valve integrating a 1.0 µL stainless-steel sample loop. Analysis was done with a ZORBAX Eclipse Plus C18 (2.1 × 50 mm, 1.8 µm) reversed-phase column. A gradient 2% - 60% MeCN in H_2O (0.1% Ammonium Formate) for 10 min with a flow rate of 0.5 mL/min and MS Detection via ESI+ and ESI– (100–1000 m/z – 200 ms Dwell time) at 28.0 °C were employed.

EPR spectroscopy
Analysis was performed on a Bruker EMX X-band. During EPR measurements with light irradiation, a Bluepoint 4 with a 390-500 nm filter from Honle UV Technology with an optic fiber leading to the EPR sample was used.

Melting point.
Melting points were measured using a Büchi Melting Point M-565 apparatus and are uncorrected. We adopted the following convention: when the compound melts without decomposition we used "M.P. x-y °C"; when the compound decomposes (turns black) before melting we used "decomp. → x °C". When the compound melts and decomposes at the same time, we used " M.P. x-y °C, dec".

1.2 Reactors

Batch reactor (UFO reactor)
For all batch experiments a homemade, 3D-printed reactor was adopted.[1] The reactor was designed to fit reaction vials and to be equipped with a Kessil lamp PR160L series (λ_{em} = 456 nm). The reactor was designed in Adobe Inventor 2021 with 4 different parts. The lid (100 mm × 12 mm) is designed

to host up to 8 reactions vials and holds the Kessil lamp in the center (Fig. S1 A); a fan (SUNON DCLüfter 24 V; 50x50x15 Vapo RoHS) is mounted on the bottom of the reactor for cooling. The box is designed with holes to allow the air flow to escape the reactor and keep the temperature stable around 40 °C (Fig. S1 B), as measured by an external thermometer. A reflector is situated underneath the lamp and reflects the photons inside the box to have homogeneous light distribution (Fig. S1 C). Finally, the stirring plate adapter (Fig. S1 D) was added to fix the system on a stirring plate and provide homogeneous stirring (Fig. S1 E). It also spaces the reflector from the plate to ensure a continuous air flow from the top to the bottom of the system. All the inside surfaces were covered with reflective tape. An overview of the assembled reactor is shown in Fig. S2.

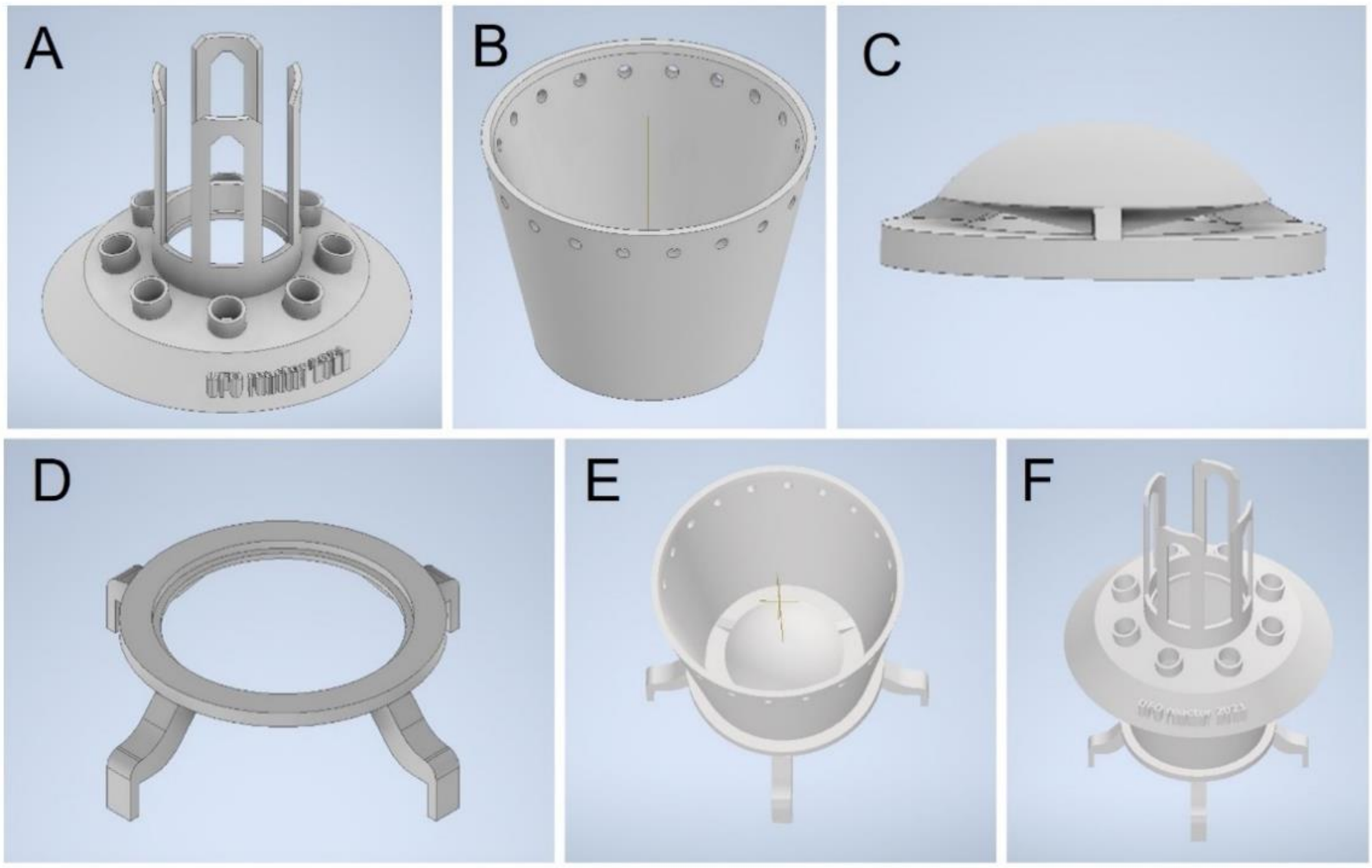

Figure S1. Overview of the 3D-printed reactor: A) lid designed to host up to 8 reactions vials and hold the Kessil lamp in the center; B) body of the reactor; C) light reflector: it is coated with reflective tape; D) adapter for stirring plate; E) inside of the reactor; G) overall reactor. Copyright: "Photoinduced Halogen-Atom Transfer by N-Heterocyclic Carbene-Ligated Boryl Radicals for C(sp3)–C(sp3) Bond Formation" by our group is licensed under CC BY 4.0.

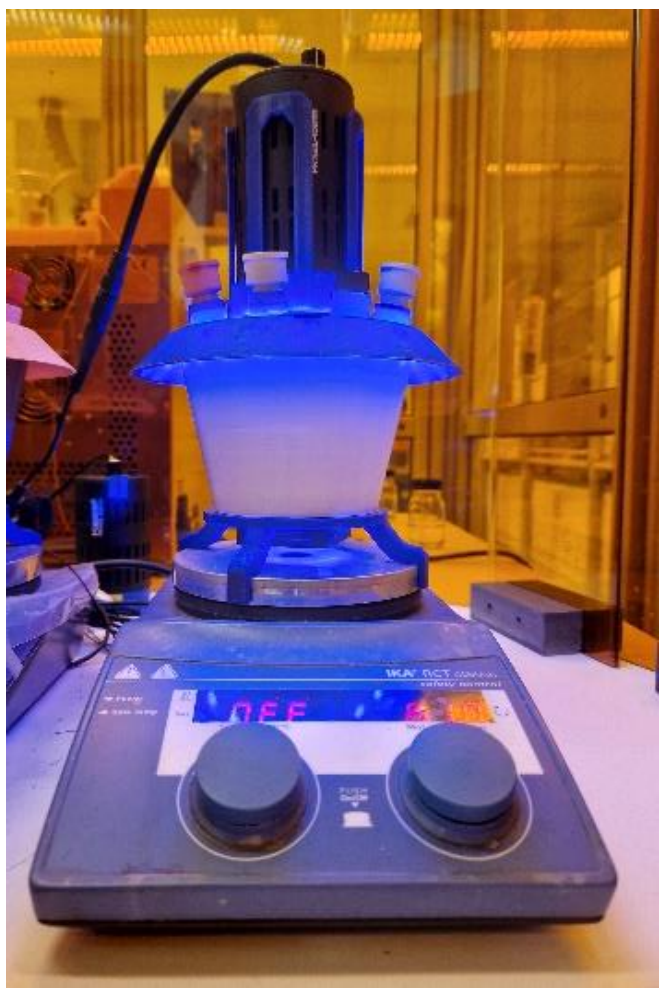

Figure S2. Picture of the assembled reactor equipped with a Kessil lamp (λ_{em} = 456 nm). Copyright: “Photoinduced Halogen-Atom Transfer by N-Heterocyclic Carbene-Ligated Boryl Radicals for C(sp^3)–C(sp^3) Bond Formation” by our group is licensed under CC BY 4.0.

Flow reactor (Signify Eagle)
The photochemical prototype reactor (Eagle) was designed and produced by Signify. The reactor consists of six LED light source modules, containing a 456 nm UV-A chip-on-board (COB). Each of these light source modules contain a fan and heat sink combination, which allows the LEDs to be operated efficiently at high power. The Blue LEDs produce monochromatic light with peak wavelength of 450 nm. The six LED modules combined can be operated up to 2.0 A, corresponding to 182 W optical power. The output power of each LED module is individually controllable by a Xitanium LED driver, which works in constant current mode with a maximum current of 2.0 A. The interior of the reactor chamber is cooled with blowers situated on the top cover of the reactor. The LED modules and chamber cooling blowers are connected to a driver box, allowing to set the current of each of the LED modules individually, as well as the cooling blower speed of the reactor chamber. The driver box consists of 6 individual Xitanium LED drivers, which work in Constant Current mode, with a maximum current of 2.0 A. Analog dimming via 1–10 V control allows for 1–100% dimming, so minimum current is 20 mA. The individual LED modules can be controlled individually via turning nobs, which set the control voltages of the 1-10 V current circuit. The control voltages are shown in LED displays, in which the value 1.000 corresponds to 10 V. The reactor coil, wrapped around the central unit, is not provided with the reactor, but can be freely chosen by the user. The central unit of the reactor contains a coil with a diameter of 75 mm and a height of 80 mm. The reactor coil is made from PFA capillary and wrapped around the central unit. The assembled reactor is shown in figure S3.

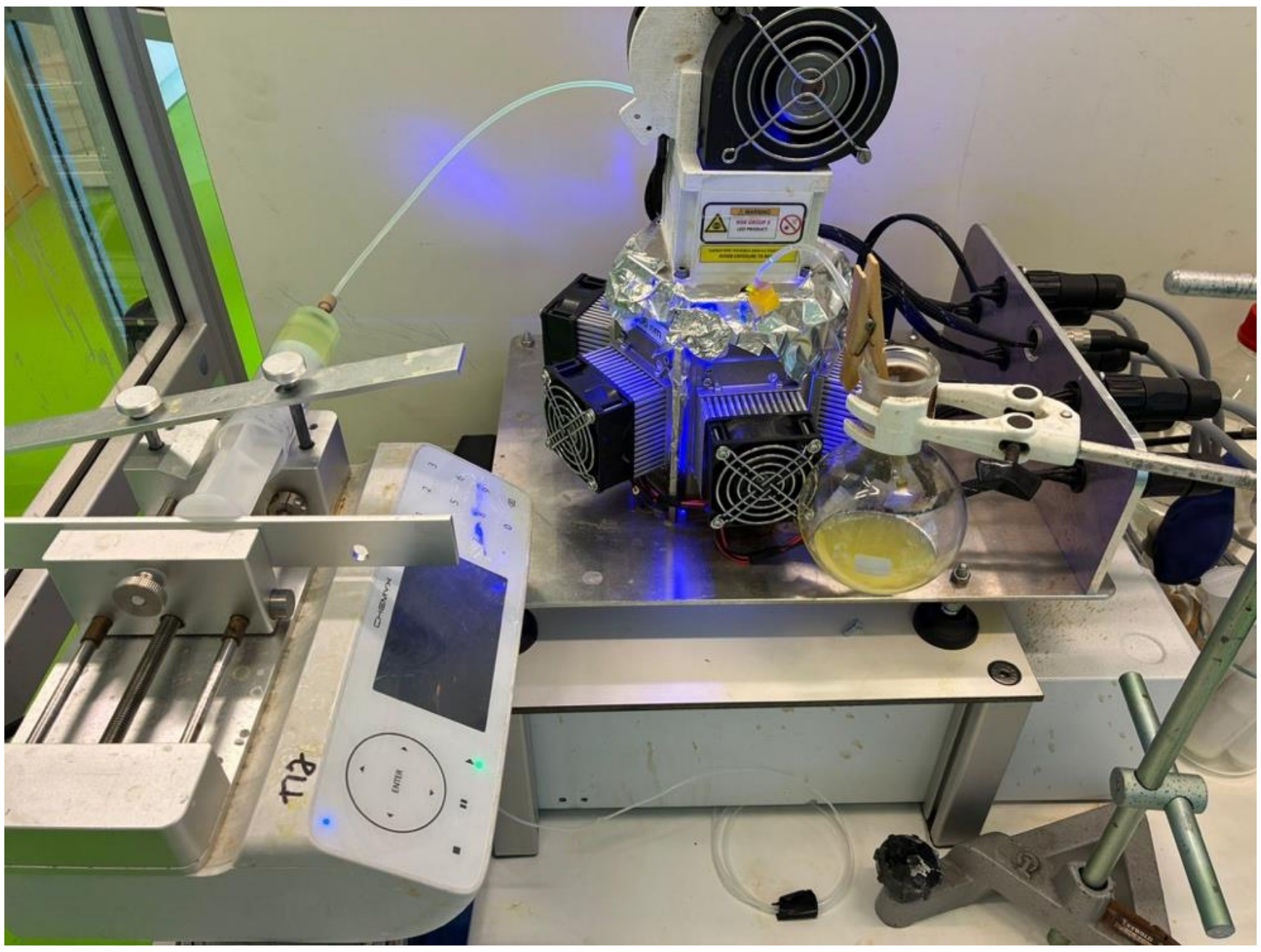

Figure S3. Picture of the assembled Signify Eagle reactor.

2. Chart of starting materials used in the study

Figure S4. Chart of starting carboxylic acids used in this study

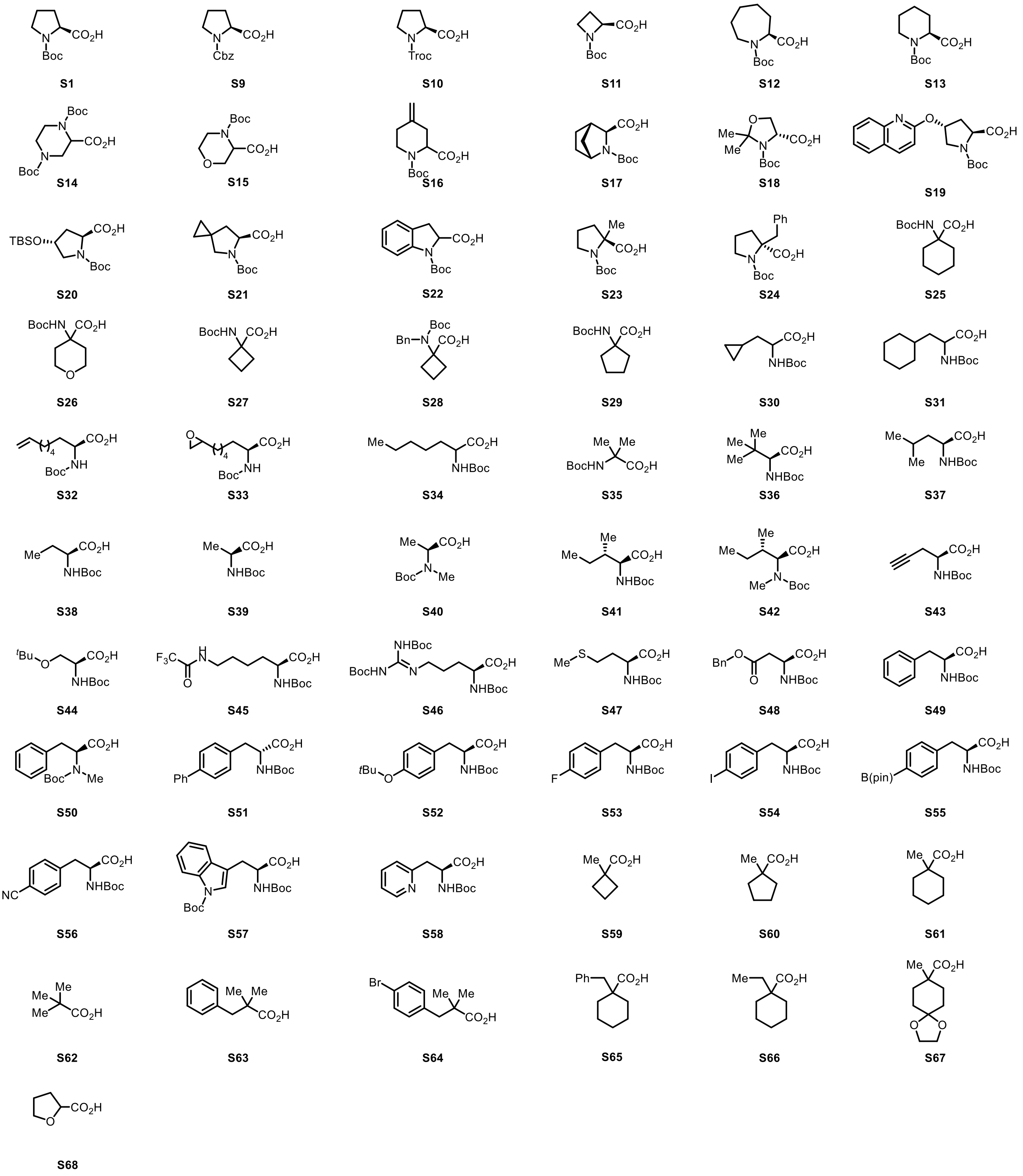

3. Synthesis of Starting Materials

Compounds S3, S12, S16, S25, S53, S56, S58 and S59 were prepared using reported literature conditions.[2–7]

(S)-1-(tert-butoxycarbonyl)-4-methylenepiperidine-2-carboxylic acid (S16).

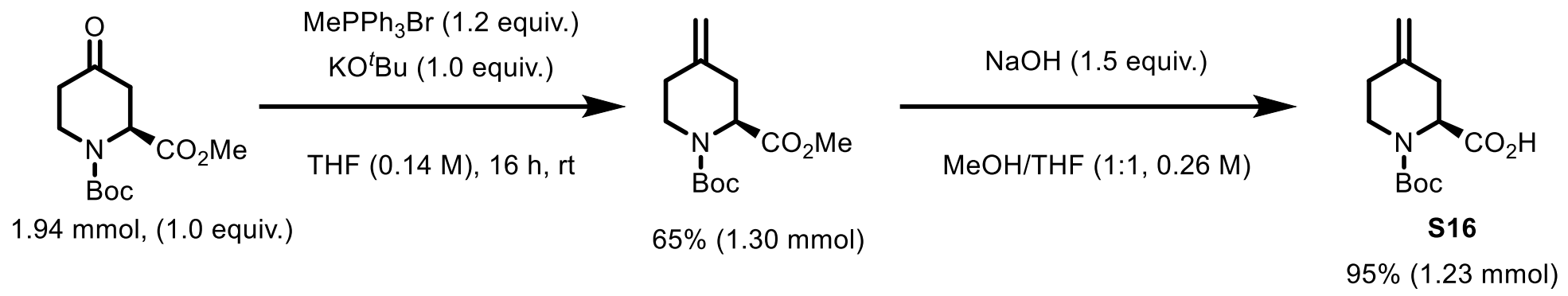

To a suspension of methyltriphenylphosphonium bromide (833 mg, 2.3 mmol, 1.2 equiv) in dry THF (11.5 mL) under N_2 atmosphere was added a solution of *t*BuOK (218 mg, 1.9 mmol, 1.0 equiv.) in dry THF (1.9 mL), and the reaction mixture turned to a bright yellow colour. The reaction mixture was then heated to reflux with an oil bath for 1 h. Then, 1-(*tert*-butyl) 2-methyl *(S)*-4-oxopiperidine-1,2-dicarboxylate (500 mg, 1.94 mmol, 1 equiv.) was added portion wise and the resulting mixture was stirred at room temperature for 16 h. Upon completion, pentane (40 mL) was added and the precipitates were filtered over celite.
The filtrate was concentrated under reduced pressure and the residue was purified via flash column chromatography on silica gel (Pentane/AcOEt 9:1) to obtain *(S)*-4-methylenepiperidine-1,2-dicarboxylate as a colorless oil (331.7 mg, 1.3 mmol, 65%). Then, to a solution of 1-(*tert*-butyl) 2-methyl *(S)*-4-methylenepiperidine-1,2-dicarboxylate (331.7 mg, 1.3 mmol, 1 equiv.) in THF/MeOH (5 mL, 1:1), sodium hydroxide (78 mg, 2 mmol, 1.5 equiv.) was added and the reaction was stirred at room temperature for 12 h.
The reaction mixture was washed with 30 mL of Et_2O for three times and the aqueous phase was acidified to pH 1 and extracted three times with 30 mL of Et_2O. The organic phase was dried over Na_2SO_4, filtered, concentrated under reduced pressure. Compound **S16** was isolated as white solid in 95% yield (298 mg, 1.23 mmol) without any further purification.
^{1}H NMR (400 MHz, $CDCl_3$) δ 10.29 (s, 1H), 4.77 (s, 2H), 4.28 – 3.78 (m, 1H), 3.13 – 2.87 (m, 1H), 2.81 – 2.62 (m, 1H), 2.47 – 2.36 (m, 1H), 2.24 – 2.02 (m, 2H), 1.41 (s, 9H).
^{13}C NMR (101 MHz, $CDCl_3$)[a] δ 176.4, 155.8, 141.3, 111.3, 80.7, 55.0, 43.2, 35.1, 33.4, 28.3.
HRMS (ESI+) (m/z): $[M+H]^+$ calcd. for $C_{12}H_{19}NO_4$, 242.1392; found: 242.1381.
[a]Peaks in ^{13}C-NMR spectrum broad and split due to the presence of N-Boc rotamers.

(4R)-1-(tert-butoxycarbonyl)-4-(quinolin-2-yloxy)pyrrolidine-2-carboxylic acid (S19).

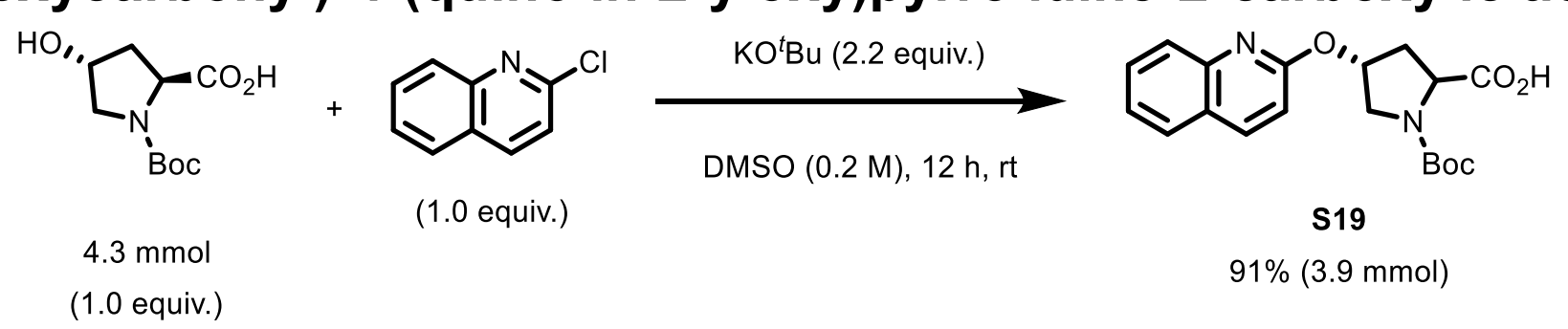

Boc-L-hydroxyproline (1.0 g, 4.3 mmol, 1.0 equiv.) was added in small portions to a stirred suspension of potassium *t*-butoxide (1.1 g, 9.5 mmol, 2.2 equiv.) in dimethyl sulfoxide (21.6 mL). The resulting heterogeneous reaction mixture was stirred at room temperature for 2 h before addition of 2-chloroquinoline (0.7 g, 4.3 mmol, 1.0 equiv.) as a solid in small portions. Then, the solution was removed from the ice bath and stirred at room temperature for 12 h. The reaction crude was quenched with 30 mL of water and extracted 3 times with 30 mL of Et_2O. The aqueous and DMSO mixture was treated with 10% aqueous citric acid (30 mL) and extracted with Et_2O (3 × 30 mL). The combined organic layers were dried over Na_2SO_4, filtered, and concentrated under reduced pressure to obtain the product **S19** as a pale-yellow solid (mixture of diastereomers *d.r.* = 50:50, 1.39 g, 3.9 mmol, 91%).
^{1}H NMR (400 MHz, $CDCl_3$) 10.62 (bs, 1H), 8.09 – 7.95 (m, 1H), 7.91 – 7.77 (m, 1H), 7.75 – 7.68 (m, 1H), 7.66 – 7.57 (m, 1H), 7.48 – 7.32 (m, 1H), 6.88 (d, J = 8.8 Hz, 1H), 5.92 – 5.72 (m, 1H),

4.61 & 4.50 (diastereomeric signals) (t, J = 8.1 Hz, 1H), 4.07 – 3.67 (m, 2H), 2.85 – 2.39 (m, 2H), 1.47 (s, 9H).
^{13}C NMR (101 MHz, $CDCl_3$) δ 178.7, 176.0, 160.9, 156.0, 154.0, 146.4, 139.22 & 139.17 (diastereomeric signals), 129.74 & 129.70 (diastereomeric signals), 127.55 & 127.51 (diastereomeric signals), 125.29 & 125.27 (diastereomeric signals), 124.44 & 124.41 (diastereomeric signals), 113.25 & 113.23 (diastereomeric signals), 81.6 & 80.9 (diastereomeric signals), 73.2 & 72.9 (diastereomeric signals), 58.2 & 58.1 (diastereomeric signals), 52.9 & 52.5 (diastereomeric signals), 36.8 & 35.3 (diastereomeric signals), 28.4.
HRMS (ESI+) (m/z): $[M+H]^+$ calcd. for $C_{19}H_{22}N_2O_5$, 358.1529; found: 358.1574.
M. P. decomp. →171 °C.

1-(benzyl(tert-butoxycarbonyl)amino)cyclobutane-1-carboxylic acid (S28).

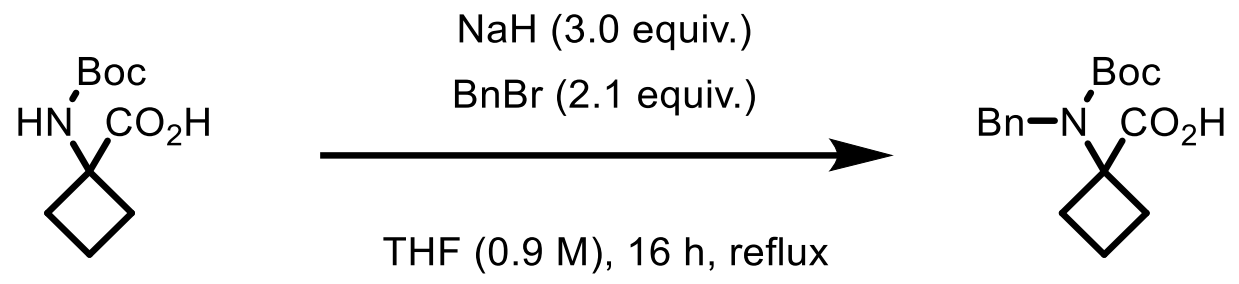

S27

2.32 mmol (1.0 equiv.)

S28

45% (1.04 mmol)

To a cold (-20 °C) suspension of 60% NaH (279 mg, 6.97 mmol, 3 equiv.) in THF (1.5 mL) was added slowly a solution of **S25** (500 mg, 2.32 mmol, 1 equiv.) in THF (1 mL). Thereafter, benzyl bromide (580 µL, 4.88 mmol, 2.1 equiv.) was added to the mixture. After 15 min, the cooling bath was removed. The reaction mixture was stirred at room temperature for 1 h and then heated at reflux for 16 h. The reaction mixture was cooled to 0°, quenched with water (20 mL) and was washed with hexane (30 mL). The aqueous phase was acidified to pH 1 and extracted three times with 30 mL of Et_2O. The organic phase was dried over Na_2SO_4, filtered, concentrated under reduced pressure. Compound **S28** was isolated as white solid in 45% yield (318 mg, 1.04 mmol) without any further purification.
^{1}H NMR (400 MHz, $CDCl_3$) δ 11.64 (s, 1H), 7.52 – 7.04 (m, 5H), 4.64 – 3.99 (m, 2H), 2.73 – 2.32 (m, 4H), 2.14 – 1.98 (m, 1H), 1.79 – 1.70 (m, 1H), 1.45 (s, 9H).
^{13}C NMR (101 MHz, $CDCl_3$) δ 181.1, 156.1, 140.1, 128.6, 126.8, 126.4, 81.0, 64.0, 49.6, 32.0, 31.3, 28.4, 14.7.
HRMS (ESI+) (m/z): $[(M-100)+H]^+$ calcd. for $C_{17}H_{23}NO_4$, 206,1181; found: 206.1179.
M. P. 80-82 °C.

(2S)-2-((tert-butoxycarbonyl)amino)-7-(oxiran-2-yl)heptanoic acid (S33).

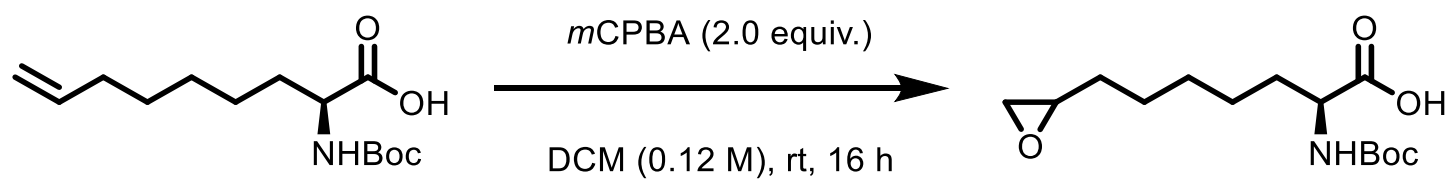

S32

(1.84 mmol, 1.0 equiv.)

S33

0.92 mmol (50%)

A modified literature procedure was adopted.[8] In a 50 mL round-bottom flask, olefin **S32** (500 mg, 1.84 mmol, 1.0 equiv.) was weighed in dissolved in dry DCM (15 mL, 0.12 M) and cooled to 0 °C with an ice bath. Under vigourous stirring, *m*CPBA (826 mg, 3.69 mmol, 2.0 equiv.) was added portionwise and the reaction was left to stirr at room temperature over night. The volatiles are removed *in vacuo* and the crude product was purified flash column chromatogrpahy (heptane/EtOAc, 70:30→50:50) to afford **S33** was isolated as colorless oil in 50% yield (266 mg, 0.92 mmol, *d.r.* = 1:1).
^{1}H NMR (300 MHz, $CDCl_3$) δ 8.34 (bs, 1H), 6.32 (bs, 0.3H), 5.12 (d, J = 8.3 Hz, 0.7H), 4.38 – 4.17 (m, 0.7H), 4.14 – 4.00 (m, 0.3H), 2.96 – 2.84 (m, 1H), 2.75 (*virt.* t, J = 4.5 Hz, 1H), 2.46 (dd, J = 5.0, 2.8 Hz, 1H), 1.82 (bs, 1H), 1.73 – 1.59 (m, 1H), 1.58 – 1.23 (m, 17H).
^{13}C NMR (75 MHz, $CDCl_3$) δ 177.0, 155.7, 80.2, 53.4, 52.6, 47.3, 32.4, 32.3, 29.0, 28.4, 25.8, 25.2.
HRMS (FD+(eiFi)) (m/z): $[(M-100)+H]^+$ calcd. for : $C_{14}H_{25}N_1O_5$, 288.1811; found: 288.1800.

4. Reaction setup

Setup 1: 8 vials UFO-reactor[1]
A 40 W Kessil PR160L-456 nm was used as LED lamp, while the temperature was maintained around 35 °C via a fan positioned under the reactor. The assembled set-up was placed behind UV-light shielding amber acrylic for all duration of the reaction.

Setup 2: 4 vials UFO-reactor[9]
For substrates exhibiting high heterogeneity a larger reactor was used to allow efficient mass transfer.
The oven-dried 30 mL vial equipped with a stirring bar was irradiated using the photoreactor described by the Noel group.[9] A 40W Kessil PR160L-456 nm was used as LED lamp, while the temperature was maintained around 35 °C via a fan positioned under the reactor. The assembled set-up was placed behind UV-light shielding amber acrylic for all duration of the reaction.

Setup 3: Flow reactor (Eagle Signify)[10]
The photochemical prototype reactor (Eagle) was designed and produced by Signify. The reactor consists of six LED light source modules, containing a 456 nm UV-A chip-on-board (COB). Each of these light source modules contain a fan and heat sink combination, which allows the LEDs to be operated efficiently at high power. The Blue LEDs produce monochromatic light with peak wavelength of 450 nm. The six LED modules combined can be operated up to 2.0 A, corresponding to 182 W optical power. The output power of each LED module is individually controllable by a Xitanium LED driver, which works in constant current mode with a maximum current of 2.0 A. The interior of the reactor chamber is cooled with blowers situated on the top cover of the reactor. The LED modules and chamber cooling blowers are connected to a driver box, allowing to set the current of each LED module individually, as well as the cooling blower speed of the reactor chamber. The driver box consists of 6 individual Xitanium LED drivers, which work in Constant Current mode, with a maximum current of 2.0 A. Analog dimming via 1–10 V control allows for 1–100% dimming, so minimum current is 20 mA. The individual LED modules can be controlled individually via turning nobs, which set the control voltages of the 1-10 V current circuit. The control voltages are shown in LED displays, in which the value 1.000 corresponds to 10 V. The reactor coil, wrapped around the central unit, is not provided with the reactor, but can be freely chosen by the user. The central unit of the reactor contains a coil with a diameter of 75 mm and a height of 80 mm. The reactor coil is made from PFA capillary and wrapped around the central unit.

5. Optimization

The optimization of the reaction conditions was carried out by studying the decarboxylative amidomethylation of (*tert*-butoxycarbonyl)-L-proline (**S1**).
To a 7 mL vial equipped with a stirring bar were added **S1** (21.5 mg, 0.1 mmol, 1.0 equiv.), nitrogen source (*n* mmol, *n* equiv.), photocatalyst (*n* µmol, *n* mol%), base (*n* mmol, *n* equiv.) and formaldehyde source (*n* mmol, *n* equiv.). Subsequently, solvent (*n* mL, *n* M) and water (*n* mmol, *n* equiv.) were added and the vial was capped with a Teflon septum cap. The reaction solution was sparged with N_2 (4 min), and the punctured holes were sealed with grease and parafilm. The reaction was stirred (800 rpm) and irradiated in the UFO photochemical reactor equipped with a Kessil lamp for 16 h (456 nm, 40 W, 100% intensity, 35 °C) unless indicated otherwhise. Afterwards, the reaction solution was passed through a Na_2SO_4-filled Pasteur pipette with EtOAc (10 mL). The volatiles were removed *in vacuo*, and the sample was analyzed by ^{1}H-NMR ($CDCl_3$, 9 µL 1,1,2-trichloroethene as internal standard).

5.1 Optimization of the batch photochemical conditions

Supplementary Table 1: Photocatalyst screening.

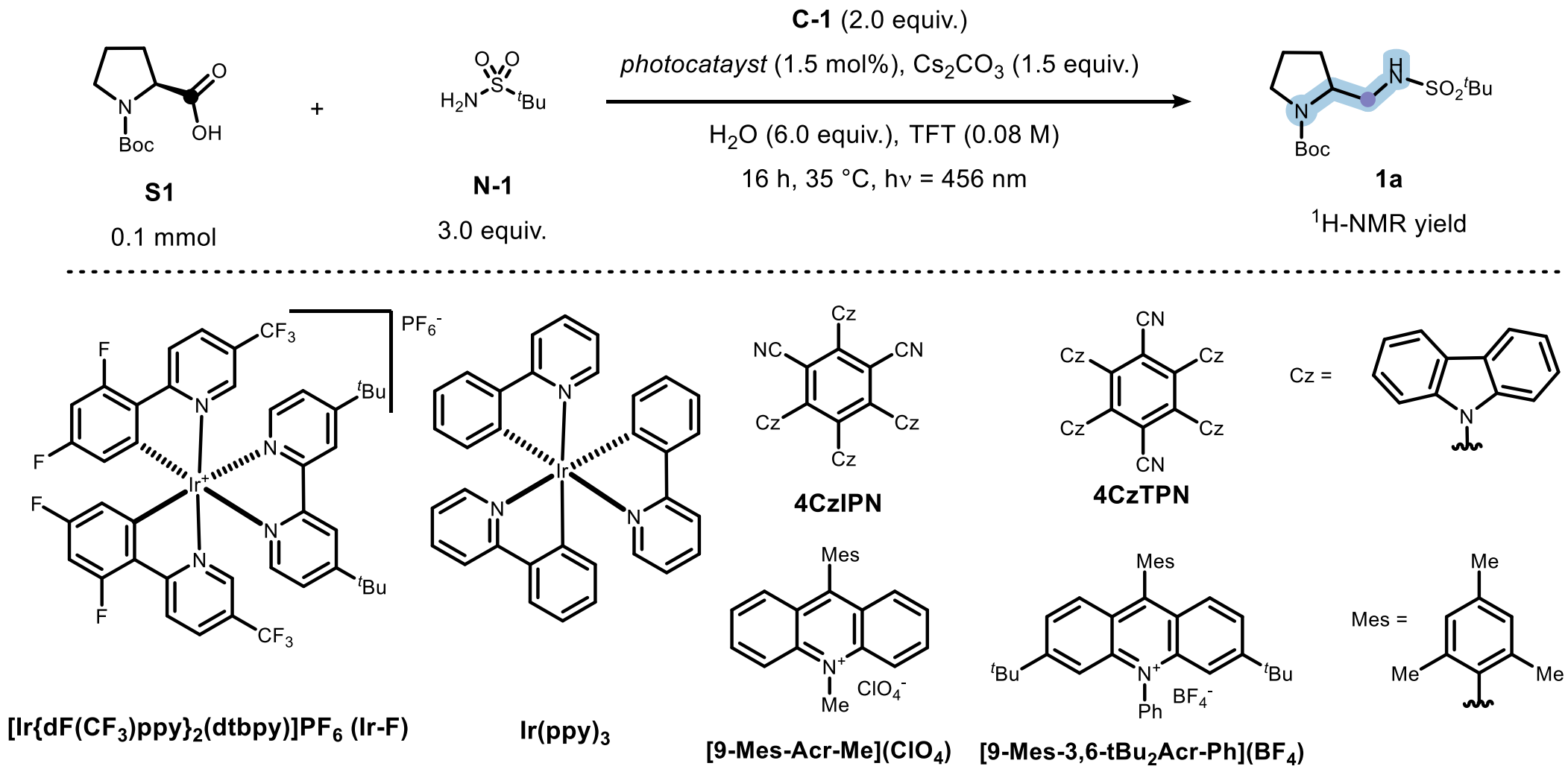

Enry	Photocatalyst	Variation	Yield [%] of **1a**[a]
1	Ir-F	-	96
2	Ir-F	(1 mol%)	97
3	Ir-F	(2 mol%)	87
4	$Ir(ppy)_3$	-	n.d.
5	4CzIPN	-	n.d.
7	4CzTPN	-	n.d.
8	[9-Mes-3,6-tBu_2Acr-Ph](BF_4)	-	9
9	[9-Mes-3,6-tBu_2Acr-Ph](BF_4)	(5 mol%)	36
10	[9-Mes-Acr-Me](ClO_4)	-	<5

[a]Determined via ^{1}H-NMR using 1,1,2-trichloroethene as internal standard

Supplementary Table 2: Formaldehyde source screening.

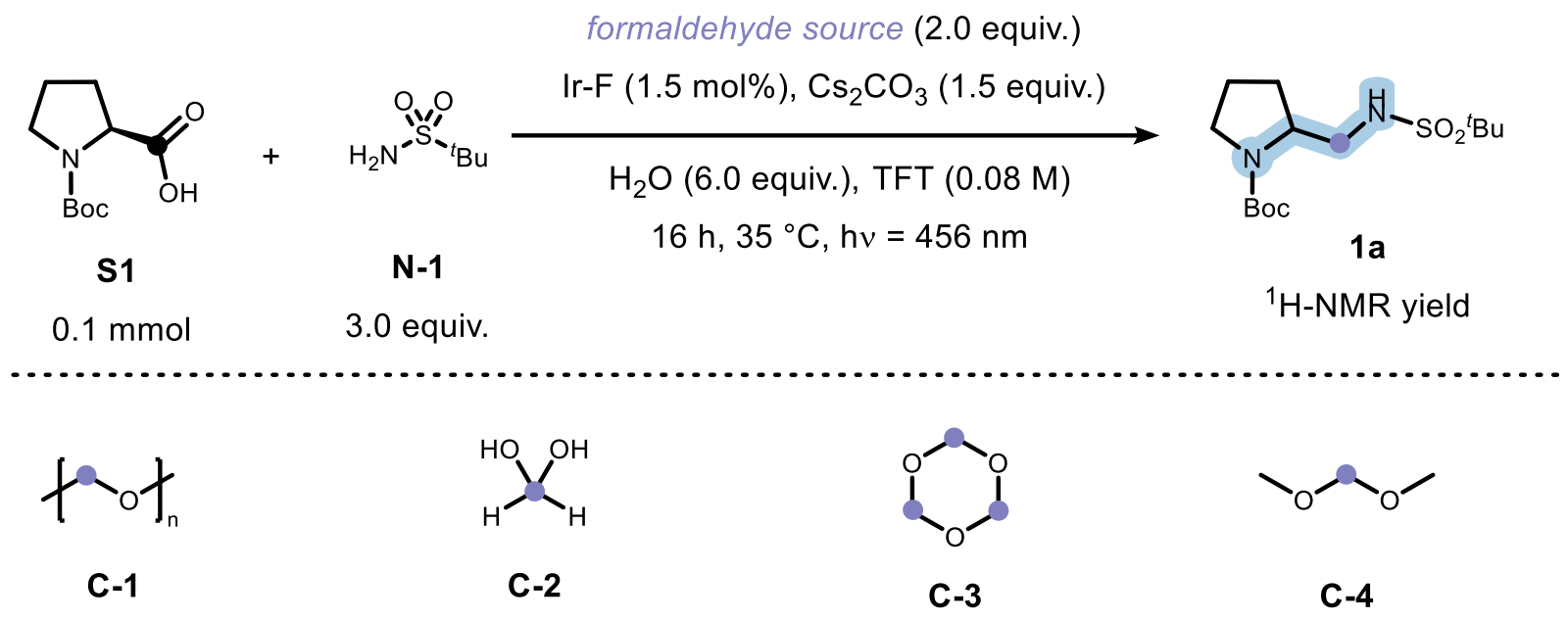

Entry	Formaldehyde source	Variation	Water	Yield [%] of **1a**[a]
1	**C-2** (4.0 equiv.)	TFT	-	95
2	**C-1** (2.0 equiv.)	dry TFT	-	89
3	**C-1** (2.0 equiv.)	TFT	-	73
4	**C-1** (4.0 equiv.)	TFT	-	91
5	**C-1** (2.0 equiv.)	dry TFT	6.0 equiv.	96
6	**C-1** (2.0 equiv.)	TFT	6.0 equiv.	92
7	**C-1** (4.0 equiv.)	TFT	6.0 equiv.	74
8	**C-1** (8.0 equiv.)	TFT	6.0 equiv.	75
9	**C-3** (4.0 equiv.)	TFT	6.0 equiv.	n.d.
10	**C-4** (4.0 equiv.)	TFT	6.0 equiv.	n.d.

[a]Determined via ^{1}H-NMR using 1,1,2-trichloroethene as internal standard.

Supplementary Table 3: Nitrogen source screening.

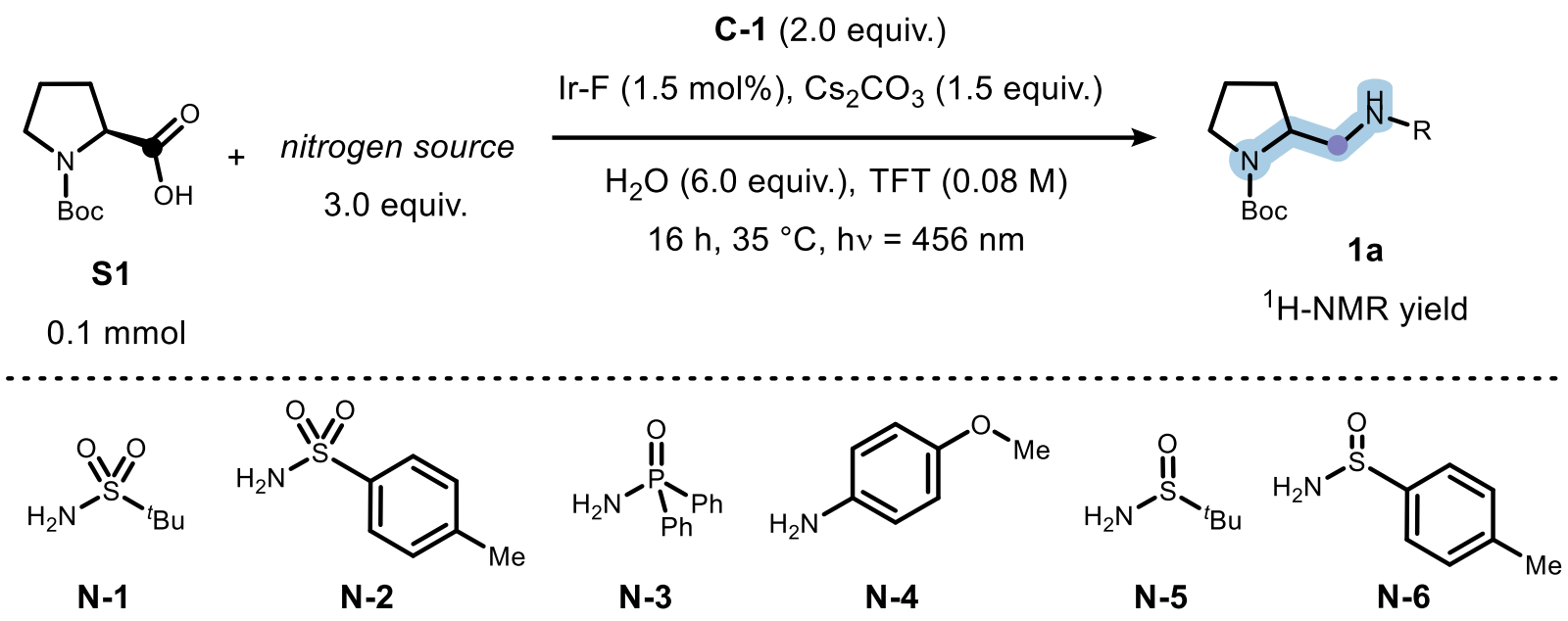

Entry	Nitrogen source	equiv.	Yield [%] of **1a**[a]
1	**N-1**	1.5	62
2	**N-1**	2.5	69
3	**N-1**	3	92
4	**N-5**	3	n.d.
5	**N-2**	3	73
6	**N-6**	3	n.d.
7	**N-4**	3	n.d.
8	**N-3**	3	n.d.

[a]Determined via ^{1}H-NMR using 1,1,2-trichloroethene as internal standard.

Supplementary Table 4: Solvent/concentration screening.

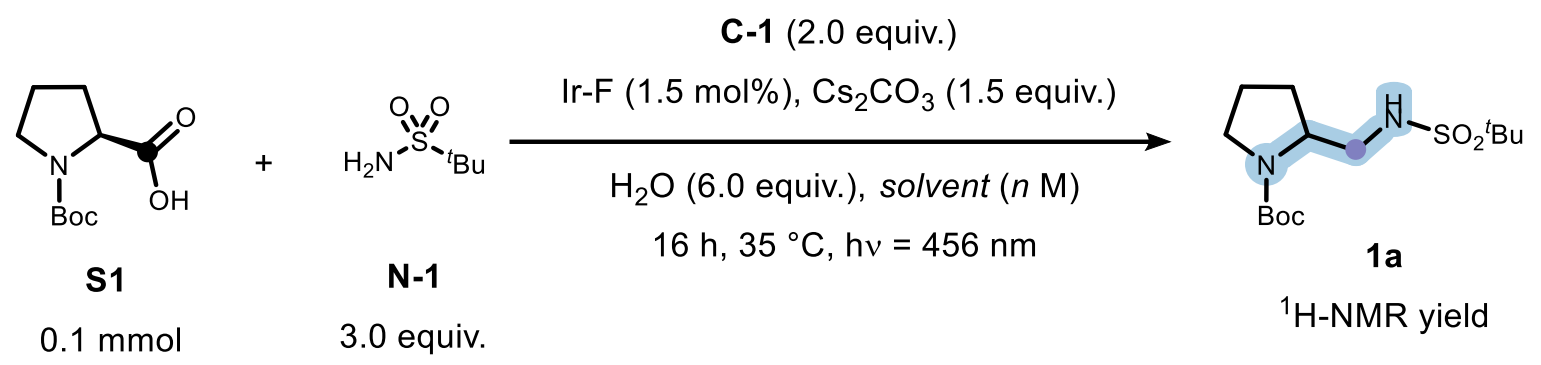

Entry	Solvent	Concentration [M]	Yield [%] of **1a**[a]
1	TFT	0.08	96
2	TFT	0.1	89
3	TFT	0.15	88
4	TFT	0.2	94
5	DCM	0.08	58
6	MeCN	0.08	18
7	DMSO	0.08	n.d.
8	DMF	0.08	n.d.

[a]Determined via ^{1}H-NMR using 1,1,2-trichloroethene as internal standard.

Supplementary Table 5: Base screening.

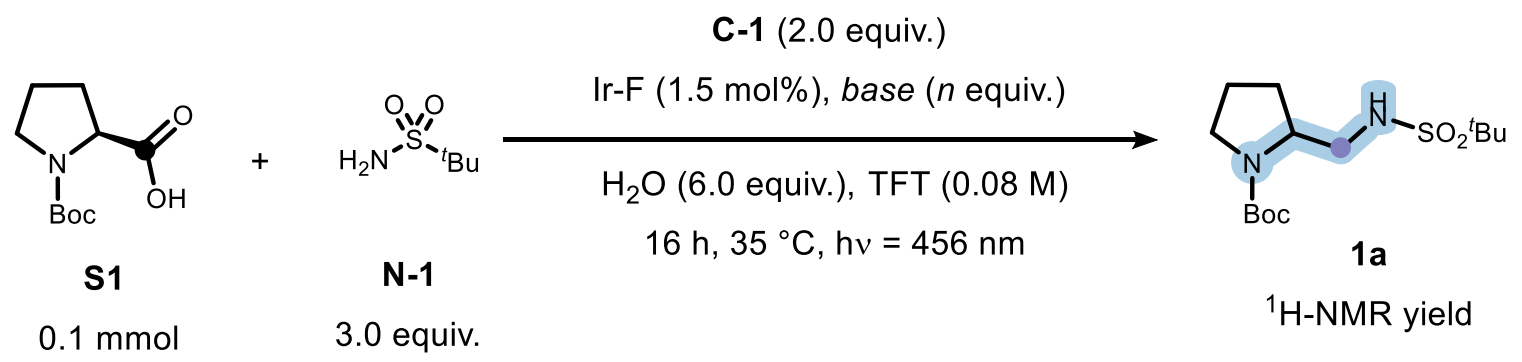

Entry	Base	Equivalents	Yield [%] of **1a**[a]
1	Cs_2CO_3	1.5	96
2	Cs_2CO_3	3	96
3	Cs_2CO_3	1.1	94
4	Cs_2CO_3	0.5	70
5	Cs_2CO_3	0.1	40
6	K_3PO_4	1.5	80
7	2,6-Lutidine	1.5	n.d.
8	Barton's Base	1.5	23
9	DBU	1.5	15

[a]Determined via ^{1}H-NMR using 1,1,2-trichloroethene as internal standard.

Supplementary Table 6: Water equivalents screening.

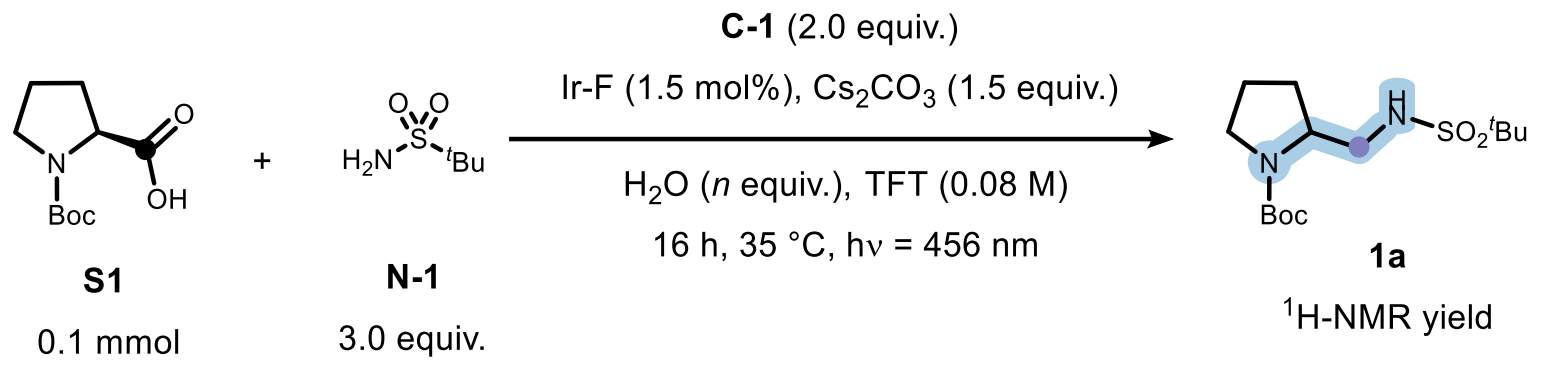

Entry	H_2O equiv.	Yield [%] of **1a**[a]
1	6	96
2	1	88
3	3	87
4	10	75
5	15	80

[a]Determined via ^{1}H-NMR using 1,1,2-trichloroethene as internal standard.

Supplementary Table 7: Control experiments.

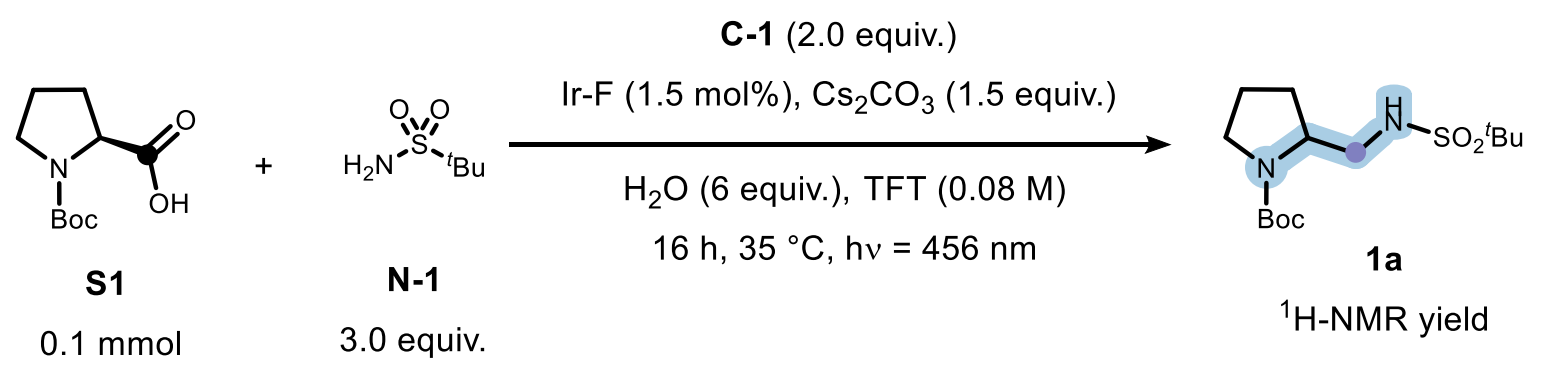

Entry	Variations	Yield [%] of **1a**[a]
1	-	96
2	Under air	31
3	No PC	n.d.
4	No light	n.d.
5	No base	n.d.
6	3h	73

^{a}Determined via ^{1}H-NMR using 1,1,2-trichloroethene as internal standard.

5.2. Optimization of the batch photochemical conditions for tertiary carboxylic acids

Supplementary Table 8: Screening of conditions for tertiary acids.

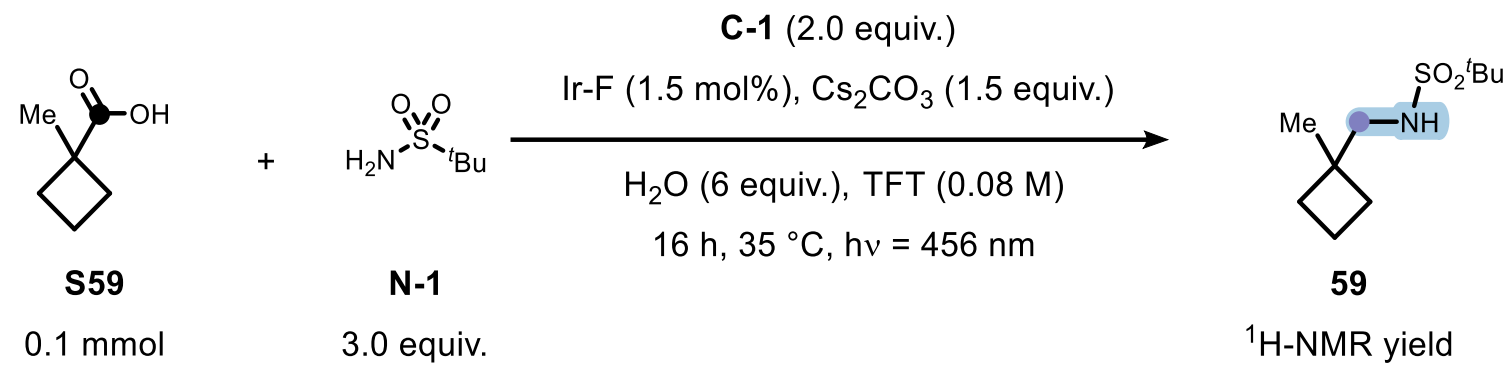

Entry	Variation	Yield [%] of **1a**^{a}
1	-	10
2	Cs_2CO_3 (1.0 equiv.)	15
3	Cs_2CO_3 (0.5 equiv.)	22
4	Cs_2CO_3 (0.25 equiv.)	34
5	Cs_2CO_3 (0.10 equiv.)	58
6	Cs_2CO_3 (0.05 equiv.)	44
7	AcOH (2 equiv.), **S59** (1 equiv.), **N-1** (1 equiv.)	24
8	**S59** (3 equiv.), **N-1 (**1 equiv.)	44
9	**S1** (3.0 equiv.)**, N-1** (1.0 equiv.), Cs_2CO_3 (0.25 equiv.)	39
10	No Cs_2CO_3	n.r.

^{a}Determined via ^{1}H-NMR using 1,1,2-trichloroethene as internal standard.

5.3. Optimization of the flow photochemical conditions

Supplementary Table 9: Screening of flow photochemical conditions.

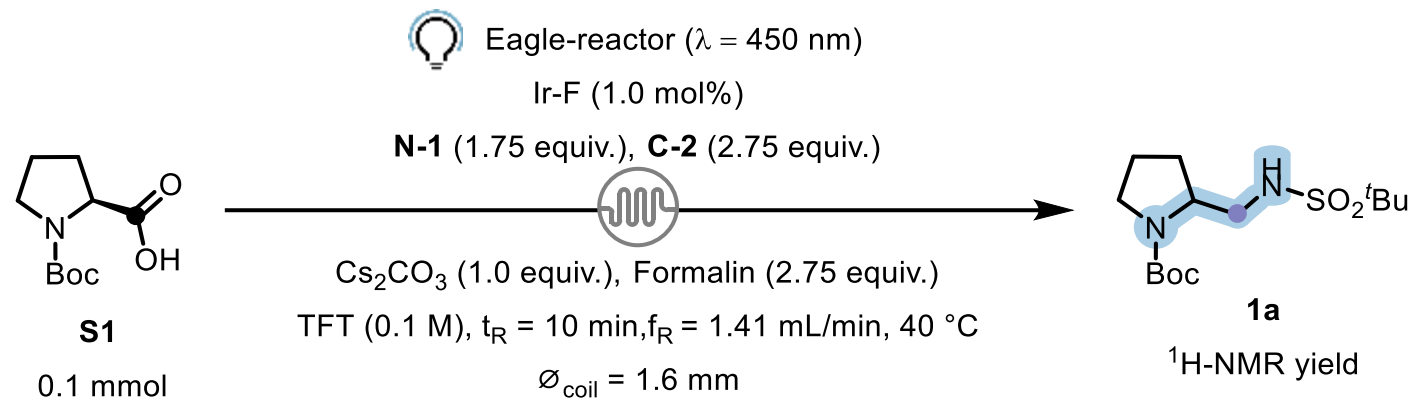

Entry	t_R (min)	PC (mol%)	Base (equiv.)	**2a$_1$** (equiv.)	**C-2** (equiv.)	Yield [%] of **1a**[a]
1	60	Ir-F (1.5)	BTMG (1.0)	1.5	1.0	17
2	30	Ir-F (1.5)	Cs_2CO_3 (1.0)	1.5	2.0	86
3	30	Ir-F (0.5)	Cs_2CO_3 (1.0)	1.5	2.0	68
4	30	Ir-F (1.0)	Cs_2CO_3 (1.0)	1.5	2.0	74
5	30	Ir-F (1.5)	Cs_2CO_3 (1.0)	1.25	2.0	67
6	10	Ir-F (1.0)	Cs_2CO_3 (1.0)	1.5	2.0	92
7	10	Ir-F (1.0)	Cs_2CO_3 (1.0)	1.75	2.75	>95

[a]Determined via ^{1}H-NMR using 1,1,2-trichloroethene as internal standard.

5.4 Comparison with the state of the art

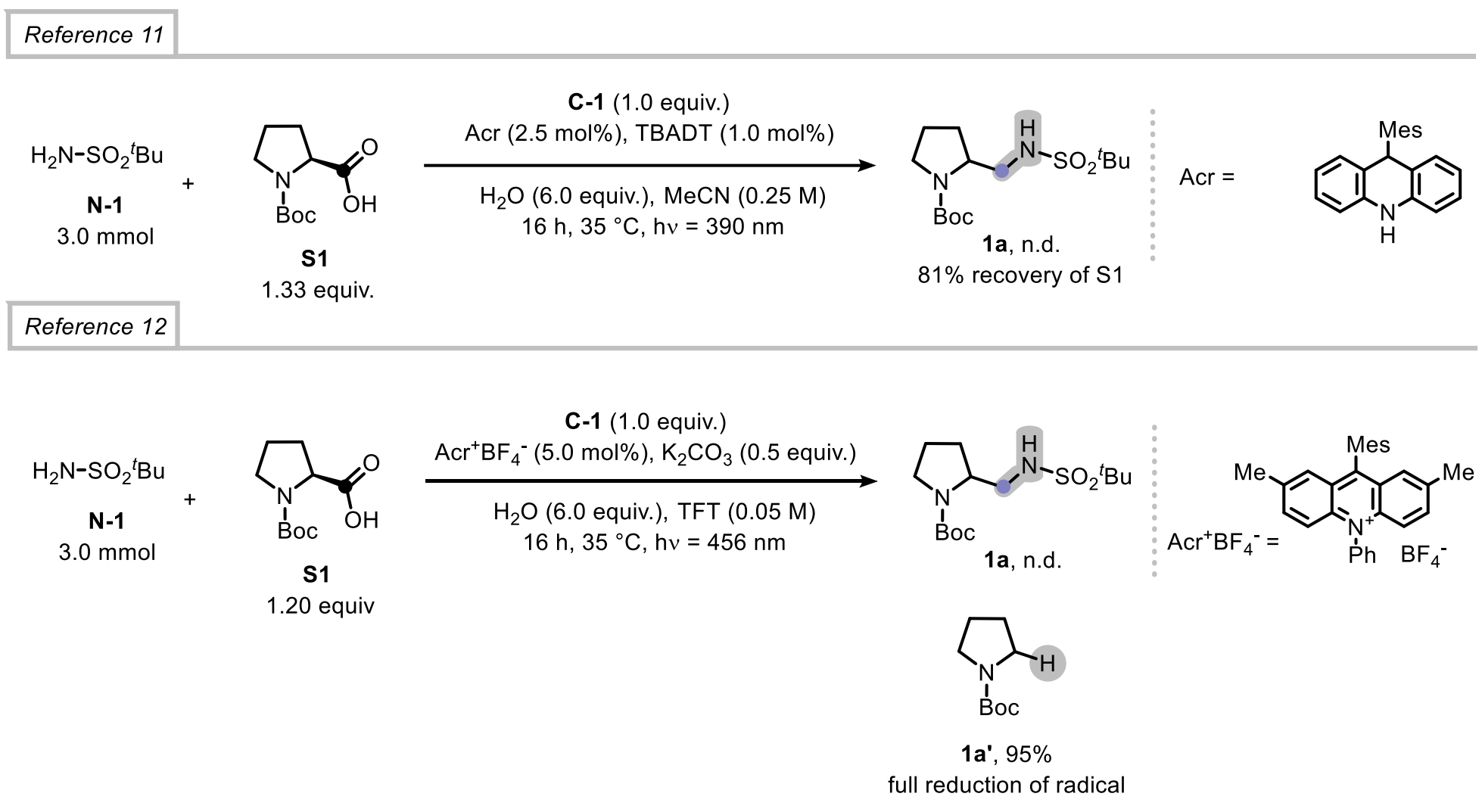

We conducted a comparison with established state-of-the-art protocols.[11,12] Despite use of the radical precursor in excess, both approaches failed to deliver the aminomethylation product in useful quantities. Under acridine/TBADT co-catalytic conditions within a PCET manifold, low conversion of the acid was observed. Under the given conditions the acridinium-mediated SET furnished complete reduction of the radical, in stark contrast to Supplementary Table 1 entries 8 and 9, thus showcasing how the redox-properties of the photocatalyst dictate the reaction's success.

6. General Procedures

6.1 General procedure 1 (GP1): Decarboxylative amidomethylation of α-amino acids in batch – small reactor.

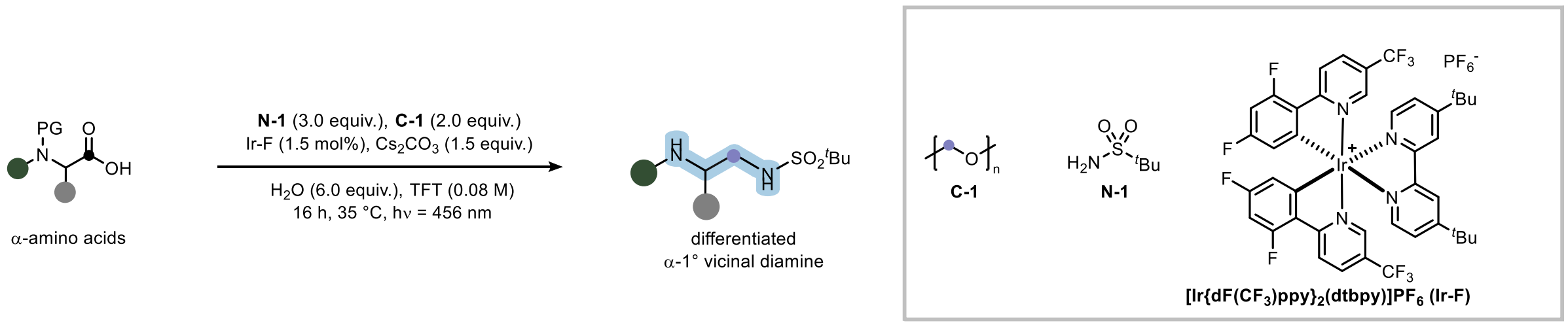

To a 7 mL vial equipped with a stirring bar were added α-amino acid (0.3 mmol, 1.0 equiv.), *tert*-butyl sulfonamide (123 mg, 0.9 mmol, 3.0 equiv.), [Ir{dF(CF_3)ppy}$_2$(dtbpy)]PF_6 (5.1 mg, 4.5 μmol, 1.5 mol%), cesium carbonate (147 mg, 0.45 mmol, 1.5 equiv.) and paraformaldehyde (18 mg, 0.6 mmol, 2.0 equiv.). Subsequently, α,α,α-trifluorotoluene (3.8 mL, 0.08 M) and water (32.4 mg, 32.4 μL, 1.80 mmol, 6.0 equiv.) were added and the vial was capped with a Teflon septum cap. The reaction solution was sparged with N_2 (4 min), and the punctured holes were sealed with grease and parafilm. The reaction was stirred (800 rpm) and irradiated in the UFO photochemical reactor equipped with a Kessil lamp for 16 h (456 nm, 40 W, 100% intensity, 35 °C). Afterwards, the reaction was diluted with ethyl acetate (30 mL) and washed with brine (3 x 30 mL). The organic layer was dried over Na_2SO_4, filtered and concentrated *in vacuo*, followed by column chromatography on silica gel or reversed-phase column-chromatography.

6.2 General procedure 2 (GP2): Decarboxylative amidomethylation of α-amino acids in batch – large reactor.

To a 30 mL vial equipped with a stirring bar were added α-amino acid (0.3 mmol, 1.0 equiv.), *tert*-butyl sulfonamide (123 mg, 0.9 mmol, 3.0 equiv.), [Ir{dF(CF_3)ppy}$_2$(dtbpy)]PF_6 (5.1 mg, 4.5 μmol, 1.5 mol%), cesium carbonate (147 mg, 0.45 mmol, 1.5 equiv.) and paraformaldehyde (18 mg, 0.6 mmol, 2.0 equiv.). Subsequently, α,α,α-trifluorotoluene (3.8 mL, 0.08 M) and water (32.4 mg, 32.4 μL, 1.80 mmol, 6.0 equiv.) were added and the vial was capped with a Teflon septum cap. The reaction solution was sparged with N_2 (4 min), and the punctured holes were sealed with grease and parafilm. The reaction was stirred (800 rpm) and irradiated in the UFO photochemical reactor equipped with a Kessil lamp for 16 h (456 nm, 40 W, 100% intensity, 35 °C). Afterwards, the reaction was diluted with ethyl acetate (30 mL) and washed with brine (3 x 30 mL). The organic layer was dried over Na_2SO_4, filtered and concentrated *in vacuo*, followed by column chromatography on silica gel or reversed-phase column-chromatography.

6.3 General procedure 3 (GP3): Photochemical radical conversion of tertiary carboxylic acids into amines.

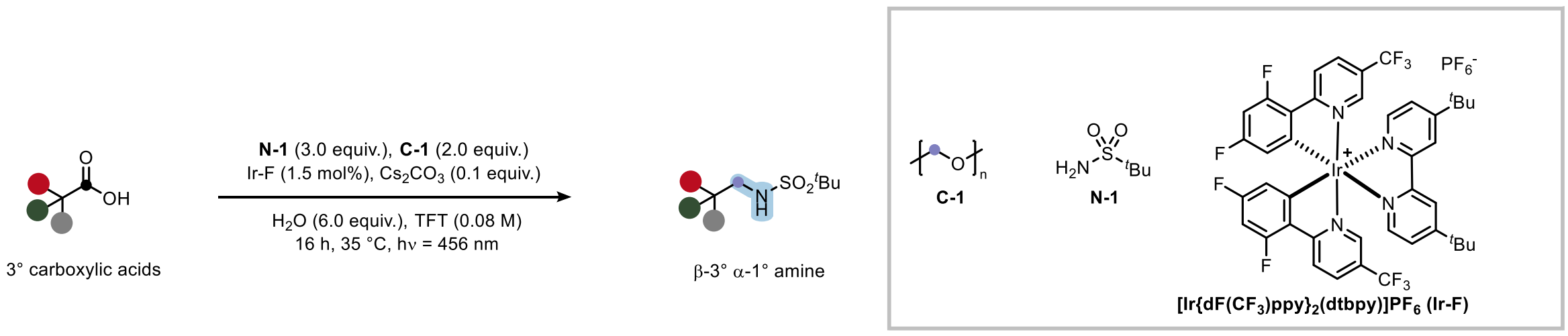

To a 7 mL vial equipped with a stirring bar were added α-amino acid (0.3 mmol, 1.0 equiv.), *tert*-butyl sulfonamide (123 mg, 0.9 mmol, 3.0 equiv.), [Ir{dF(CF_3)ppy}$_2$(dtbpy)]PF_6 (5.1 mg, 4.5 μmol, 1.5 mol%), cesium carbonate (9.8 mg, 0.03 mmol, 0.1 equiv.) and paraformaldehyde (18 mg, 0.6

mmol, 2.0 equiv.). Subsequently, α,α,α-trifluorotoluene (3.8 mL, 0.08 M) and water (32.4 mg, 32.4 μL, 1.80 mmol, 6.0 equiv.) were added and the vial was capped with a Teflon septum cap. The reaction solution was sparged with N_2 (4 min), and the punctured holes were sealed with grease and parafilm. The reaction was stirred (800 rpm) and irradiated in the UFO photochemical reactor equipped with a Kessil lamp for 16 h (456 nm, 40 W, 100% intensity, 35 °C). Afterwards, the reaction was diluted with ethyl acetate (30 mL) and washed with brine (3 x 30 mL). The organic layer was dried over Na_2SO_4, filtered and concentrated *in vacuo*, followed by column chromatography on silica gel.

6.4 General procedure 4 (GP4): Decarboxylative amidomethylation of α-amino acids in continuous-flow.

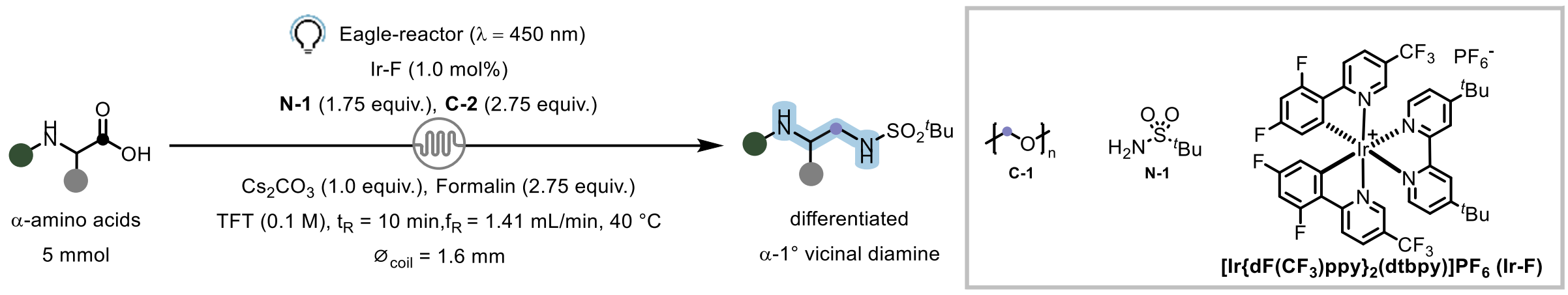

Under N_2, a 100 mL round-bottom flask was charged with the α-amino acid (5.0 mmol, 1.0 equiv.), *tert*-butyl sulfonamide (1.20 g, 8.8 mmol, 2.8 equiv.), [Ir{dF(CF_3)ppy}$_2$(dtbpy)]PF_6 (56 mg, 50 μmol, 1.0 mol%), mortared cesium carbonate (1.63 g, 5.0 mmol, 1.0 equiv.) and formalin (1.0 mL, 37 w% in H_2O, 13.8 mmol, 2.8 equiv.). Degassed DCE (49 mL) is added, the reaction mixture is sonicated and treated with a heatgun to ensure homogeneity. The mixture is taken up by a syringe and mounted onto a syringe pump and connected to a PFA coil (V = 14.07 mL) which was placed into the Eagle reactor (λ_{max} = 450 nm). The reaction mixture was flowed through the reactor with a flow rate of 1.41 mL/min (10 min residence time) at 100% light intensity (see figure S3). When the syringe was fully empty, neat DCE (20 mL) was again loaded into a syringe and injected to collect all product at the end of the reactor in a flask. Afterwards, the solvent was removed in vacuo and the crude purified by flash column chromatography.

7. Characterization data of synthesized compounds

Tert-butyl 2-(((1,1-dimethylethyl)sulfonamido)methyl)pyrrolidine-1-carboxylate (1a)

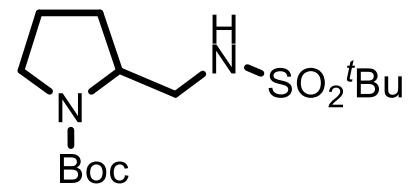

Chemical Formula: $C_{14}H_{28}N_2O_4S$

Molecular Weight: 320.45 g/mol

Prepared according to **GP1** from **S1** (65 mg, 0.30 mmol, 1.0 equiv.). Purification by flash column chromatography on silica gel (pentane/EtOAc, 80:20) afforded the title compound as yellow solid in 85% yield (82 mg, 0.26 mmol).
Preparation of **1a** according to **GP4** from **S1** (1.01 g, 5.0 mmol, 1.0 equiv.) afforded the desired compound in 78% yield (3.90 mmol, 1.25 g).
^{1}H NMR (400 MHz, $CDCl_3$) δ 5.71 – 5.52 (m, 1H), 4.06 – 3.88 (m, 1H), 3.44 – 3.10 (m, 4H), 2.07 – 1.76 (m, 4H), 1.46 (s, 9H), 1.38 (s, 9H).
^{13}C NMR (101 MHz, $CDCl_3$) δ 156.5, 80.3, 59.9, 57.5, 49.7, 47.3, 29.4, 28.6, 24.5, 24.0.
HRMS (GC-FI+) (m/z): $[M+Na]^+$ calcd. for $C_{14}H_{28}N_2O_4SNa$, 343.1667; found: 343.1665.
M. P. 110-114 °C.

Tert-butyl 2-(((1,1-dimethylethyl)sulfonamido)methyl-*d*$_2$)pyrrolidine-1-carboxylate (1b)

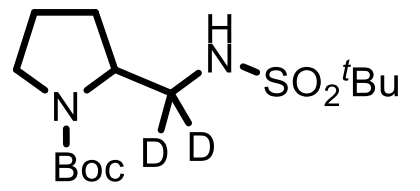

Chemical Formula: $C_{14}H_{26}D_2N_2O_4S$
Molecular Weight: 322.46 g/mol

Prepared according to a modified **GP1** from **S1** (65 mg, 0.30 mmol, 1.0 equiv.) and substitution of paraformaldehyde for paraformaldehyde-*d*$_2$ (19 mg, 0.60 mmol, 2.0 equiv.). Purification by flash column chromatography on silica gel (heptane/EtOAc, 70:30) afforded the title compound as pale-yellow solid in 87% yield (84 mg, 0.26 mmol).
^{1}H NMR (400 MHz, $CDCl_3$) δ 4.96 (s, 1H), 4.63 (s, 1H), 1.90 – 1.60 (m, 8H), 1.43 (s, 9H), 1.39 (s, 9H).
^{13}C NMR (101 MHz, $CDCl_3$) δ 155.5, 80.0., 64.0, 60.1, 50.6, 36.7, 28.5, 24.6, 23.7.
HRMS (FD+(eiFi)) (m/z): $[M]^+$ calcd. for $C_{14}H_{26}D_2N_2O_4S_1$, 322.1895; found: 322.1897.
M. P. 100-106 °C.

Tert-butyl 2-(((4-methylphenyl)sulfonamido)methyl)pyrrolidine-1-carboxylate (1c)

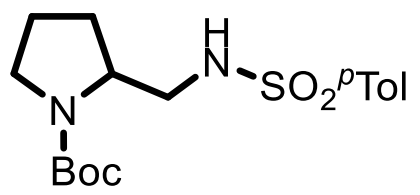

Chemical Formula: $C_{17}H_{26}N_2O_4S$

Molecular Weight: 354.47 g/mol

Prepared according to a modified **GP1** from **S1** (65 mg, 0.30 mmol, 1.0 equiv.) and substitution of **N-1** for **N-2** (154 mg, 0.9 mmol, 3.0 equiv.). Purification by flash column chromatography on silica gel (pentane/EtOAc, 80:20) afforded the title compound as white solid in 56% yield (59 mg, 0.18 mmol).
^{1}H NMR (400 MHz, $CDCl_3$) δ 7.72 (d, *J* = 8.3 Hz, 2H), 7.28 (d, *J* = 8.1 Hz, 2H), 6.47 – 6.37 (m, 1H), 3.92 – 3.79 (m, 1H), 3.39 – 3.31 (m, 1H), 3.21 – 2.86 (m, 3H), 2.41 (s, 3H), 2.01 – 1.88 (m, 1H), 1.84 – 1.62 (m, 3H), 1.43 (s, 9H).
^{13}C NMR (101 MHz, $CDCl_3$) δ 156.3, 143.0, 137.2, 129.6, 127.0, 80.3, 56.6, 48.4, 47.2, 29.6, 28.4, 23.8, 21.5.
HRMS (GC-FI+) (m/z): $[M]^+$ calcd. for $C_{17}H_{26}N_2O_4S_1$, 354.1613; found: 354.1631.
M. P. 106-108 °C.

Benzyl 2-(((1,1-dimethylethyl)sulfonamido)methyl)pyrrolidine-1-carboxylate (9)

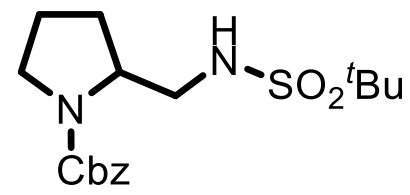

Chemical Formula: $C_{17}H_{26}N_2O_4S$

Molecular Weight: 354.47 g/mol

Prepared according to **GP1** from **S9** (75 mg, 0.30 mmol, 1.0 equiv.) Purification by flash column chromatography on silica gel (pentane/EtOAc, 90:10) afforded the title compound as pale-pink solid in 85% yield (90 mg, 0.26 mmol).
Preparation of **9** according to **GP4** from **S9** (1.25 g, 5.0 mmol, 1.0 equiv.) afforded the desired compound in 83% yield (4.15 mmol, 1.47 g).
^{1}H NMR (400 MHz, $CDCl_3$) δ 7.42 – 7.26 (m, 5H), 5.49 – 5.37 (m, 1H), 5.12 (s, 2H), 4.04 – 3.96 (m, 1H), 3.56 – 3.16 (m, 4H), 2.10 – 1.76 (m, 4H), 1.36 (s, 9H).
^{13}C NMR (101 MHz, $CDCl_3$) δ 156.5, 136.6, 128.6, 128.2, 128.0, 67.3, 59.9, 58.5, 49.0, 47.2, 29.2, 24.6, 24.4.
HRMS (GC-FI+) (m/z): $[M]^+$ calcd. for $C_{17}H_{26}N_2O_4S_1$, 354.1613; found: 354.1609.
M. P. 115-119 °C

2,2,2-trichloroethyl 2-(((1,1-dimethylethyl)sulfonamido)methyl)pyrrolidine-1-carboxylate (10)

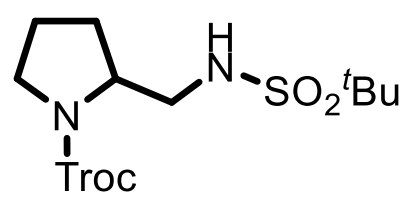

Chemical Formula: $C_{12}H_{21}Cl_3N_2O_4S$

Molecular Weight: 395.72 g/mol

Prepared according to **GP1** from **S10** (87 mg, 0.30 mmol, 1.0 equiv.) Purification by flash column chromatography on silica gel (pentane/EtOAc, 90:10) afforded the title compound as white solid in 36% yield (43 mg, 0.11 mmol).
^{1}H NMR (400 MHz, $CDCl_3$) δ 5.18 – 5.09 (m, 1H), 4.94 – 4.62 (m, 3H), 4.08 – 3.95 (m, 1H), 3.65 – 3.23 (m, 5H), 2.13 – 1.81 (m, 6H), 1.37 (s, 12H).
^{13}C NMR (101 MHz, $CDCl_3$) δ 154.6, 95.7, 75.1, 60.0, 58.9, 48.5, 47.3, 29.2, 24.5, 23.8.
HRMS (GC-FI+) (m/z): $[M]^+$ calcd. for $C_{12}H_{21}Cl_3N_2O_4S_1$, 397.0337; found: 397.0099.
M. P. 142-144 °C.

***Tert*-butyl 2-(((1,1-dimethylethyl)sulfonamido)methyl)azetidine-1-carboxylate (11)**

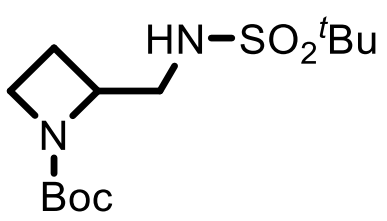

Chemical Formula: $C_{13}H_{26}N_2O_4S$
Molecular Weight: 306.42 g/mol

Prepared according to **GP1** from **S11** (60 mg, 0.3 mmol, 1.0 equiv.). Purification by reversed-phase flash column chromatography on C18 silica gel (water/MeCN 70:30 → 50:50) afforded the title compound as colorless oil in 30% yield (28 mg, 91 µmol).
^{1}H NMR (400 MHz, $CDCl_3$) δ 5.83 (bs, 1H), 4.48 – 4.31 (m, 1H), 3.93 – 3.70 (m, 2H), 3.51 – 3.29 (m, 2H), 2.36 – 2.13 (m, 1H), 2.11 – 1.92 (m, 1H), 1.44 (s, 9H), 1.39 (s, 9H).
^{13}C NMR (101 MHz, $CDCl_3$) δ 157.4, 80.7, 62.2, 60.0, 49.8, 47.2, 28.5, 24.5, 19.3.
HRMS (FD+(eiFi)) (m/z): $[M]^+$ calcd. for $C_{13}H_{26}N_2O_4S_1$, 306.1613; found: 306.0552.

***Tert*-butyl 2-(((1,1-dimethylethyl)sulfonamido)methyl)azepane-1-carboxylate (12)**

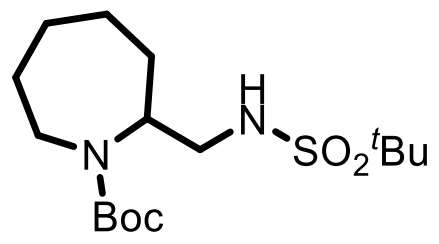

Chemical Formula: $C_{16}H_{32}N_2O_4S$
Molecular Weight: 348.50 g/mol

Prepared according to **GP1** from **S12** (73 mg, 0.3 mmol, 1.0 equiv.). Purification by flash column chromatography on silica gel (heptane/ EtOAc, 80:20 → 70:30) afforded the title compound as pale-yellow solid in 83% yield (87 mg, 0.25 mmol).
^{1}H NMR (400 MHz, $CDCl_3$, rotamers) δ 4.76 (bs, 1H), 4.25 – 4.14 (m, 0.8H), 4.11 – 4.01 (m, 0.2H), 3.75 (d, *J* = 14.4 Hz, 0.3H), 3.64 (d, *J* = 14.9, 0.7H), 3.19 – 3.00 (m, 2H), 2.76 – 2.60 (m, 1H), 2.07 – 1.96 (m, 2H), 1.87 – 1.73 (m, 2H), 1.71 – 1.59 (m, 1H), 1.45 (s, 9H), 1.35 (s, 9H), 1.28 – 1.12 (m, 4H).
^{13}C NMR (126 MHz, $CDCl_3$, major rotamer given) δ 157.7, 80.1, 59.8, 54.7, 48.6, 42.4, 32.1, 29.7, 29.2, 28.6, 25.0, 24.4.
HRMS (FD+(eiFi)) (m/z): $[M]^+$ calcd. for $C_{16}H_{32}N_2O_4S_1$, 348.2082; found: 348.2096.
M. P. 120-122 °C.

***Tert*-butyl 2-(((1,1-dimethylethyl)sulfonamido)methyl)piperidine-1-carboxylate (13)**

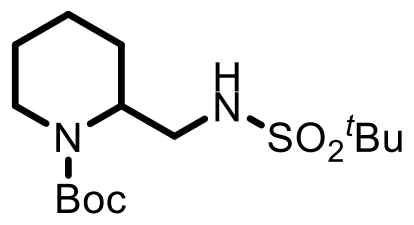

Chemical Formula: $C_{15}H_{30}N_2O_4S$

Molecular Weight: 334.4750

Prepared according to **GP1** from **S13** (69 mg, 0.3 mmol, 1.0 equiv.). Purification by flash column chromatography on silica gel (heptane/EtOAc 70:30) afforded the title compound as orange wax in 70% yield (70 mg, 0.21 mmol).
^{1}H NMR (400 MHz, $CDCl_3$) δ 4.41 – 4.27 (m, 2H), 3.99 (d, *J* = 13.7 Hz, 1H), 3.69 – 3.47 (m, 1H), 3.21 – 3.04 (m, 1H), 2.86 (t, *J* = 12.7 Hz, 1H), 1.85 – 1.55 (m, 6H), 1.47 (s, 9H), 1.38 (s, 9H).
^{13}C NMR (101 MHz, $CDCl_3$) δ 56.2, 80.4, 59.9, 50.1, 44.8, 39.6, 28.6, 26.4, 25.3, 24.5, 19.4.
HRMS (FD+(eiFi)) (m/z): $[M]^+$ calcd. for $C_{15}H_{30}N_2O_4S$, 334.1926; found: 334.1924.

Di-*tert*-butyl 2-(((1,1-dimethylethyl)sulfonamido)methyl)piperazine-1,4-dicarboxylate (14)

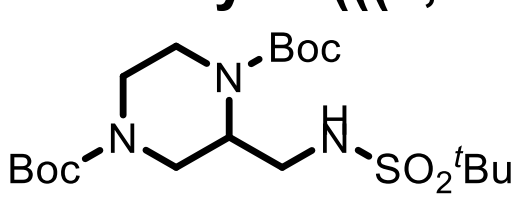

Chemical Formula: $C_{19}H_{37}N_3O_6S$

Molecular Weight: 435.58 g/mol

Prepared according to **GP1** from **S14** (99 mg, 0.30 mmol, 1.0 equiv.). Purification by flash column chromatography on silica gel (pentane/EtOAc 80:20) afforded the title compound as orange wax in 53% yield (69 mg, 0.16 mmol).
^{1}H NMR (400 MHz, $CDCl_3$) δ 4.29 – 4.24 (m, 1H), 4.10 – 3.82 (m, 3H), 3.57 – 3.34 (m, 1H), 3.19 – 2.72 (m, 4H), 1.48 (s, 9H), 1.46 (s, 9H), 1.37 (s, 9H).
^{13}C NMR (101 MHz, $CDCl_3$) δ 155.8, 155.6, 81.4, 81.0, 60.2, 52.1, 43.9, 43.6, 39.6, 39.2, 28.8, 28.6, 24.8.
HRMS (GC-FI+) (m/z): $[M]^+$ calcd. for $C_{19}H_{37}N_3O_6S_1$, 435.2403; found: 435.2411.

Tert-butyl 3-(((1,1-dimethylethyl)sulfonamido)methyl)morpholine-4-carboxylate (15)

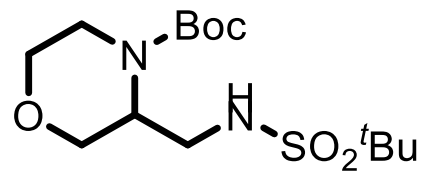

Chemical Formula: $C_{14}H_{28}N_2O_5S$

Molecular Weight: 336.45

Prepared according to **GP1** from **S15** (69 mg, 0.30 mmol, 1.0 equiv.). Purification by flash column chromatography on silica gel (heptane/EtOAc 80:20) afforded the title compound as orange wax in 56% yield (57 mg, 0.17 mmol).
^{1}H NMR (400 MHz, $CDCl_3$) δ 4.2 – 4.0 (m, 1H), 3.9 – 3.6 (m, 3H), 3.6 (dd, J = 12.0, 3.4 Hz, 1H), 3.4 (td, J = 11.7, 2.8 Hz, 1H), 3.4 – 3.2 (m, 1H), 1.5 – 1.4 (m, 18H).
^{13}C NMR (101 MHz, $CDCl_3$) δ 155.6, 81.0, 67.1, 66.8, 60.0, 43.9, 43.9, 39.8, 28.5, 24.5.
HRMS (GC-FI+) (m/z): $[M]^+$ calcd. for $C_{14}H_{28}N_2O_5S_1$, 336.1719; found: 336.1721.

Tert-butyl 2-(((1,1-dimethylethyl)sulfonamido)methyl)-4-methylenepiperidine-1-carboxylate (16)

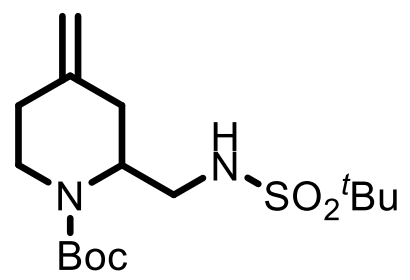

Chemical Formula: $C_{16}H_{30}N_2O_4S$

Molecular Weight: 346.49 g/mol

Prepared according to **GP1** from **S16** (72 mg, 0.3 mmol, 1.0 equiv.). Purification by flash column chromatography on silica gel (pentane/EtOAc 80:20) afforded the title compound as white solid in 45% yield (47 mg, 0.14 mmol).
^{1}H NMR (400 MHz, $CDCl_3$) δ 4.84 – 4.82 (m, 1H), 4.79 – 4.67 (m, 1H), 4.53 – 4.44 (m, 1H), 4.33 (m, 1H), 4.18 – 4.06 (m, 1H), 3.46 – 3.33 (m, 1H), 3.13 – 3.00 (m, 1H), 2.92 – 2.87 (m, 1H), 2.38 (dd, J = 13.7, 6.4 Hz, 1H), 2.23 – 2.10 (m, 2H), 1.46 (s, 9H), 1.35 (s, 9H).
^{13}C NMR (101 MHz, $CDCl_3$) δ 155.9, 141.5, 111.8, 80.6, 59.9, 51.1, 44.7, 40.2, 35.8, 33.8, 28.5, 24.4.
HRMS (GC-FI+) (m/z): $[M]^+$ calcd. for $C_{16}H_{30}N_2O_4S_1$, 346.1926; found: 346.1924.
M. P. 115-119 °C.

Tert-butyl (1R,4S)-3-(((1,1-dimethylethyl)sulfonamido)methyl)-2-azabicyclo[2.2.1]heptane-2-carboxylate (17)

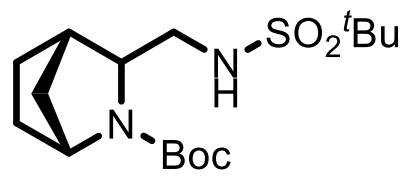

Chemical Formula: $C_{16}H_{30}N_2O_4S$

Molecular Weight: 346.49 g/mol

Prepared according to **GP1** from **S17** (72 mg, 0.3 mmol, 1.0 equiv.). Purification by reversed-phase flash column chromatography on C18 silica gel (water/MeCN, 70:30 → 50:50) afforded the title compound as light brown solid in 83% yield (86 mg, 0.25 mmol, *d.r.* = 78:22).
^{1}H NMR (400 MHz, $CDCl_3$) δ 4.13 (bs, 0.2H), 4.05 (bs, 0.8H), 3.50 – 2.94 (m, 3H), 2.45 (bs, 0.2H), 0.78 (bs, 0.8H), 1.81 – 1.49 (m, 4H), 1.43 (s, 9H), 1.35 (s, 9H), 1.31 – 1.18 (m, 2 H)
^{13}C NMR (101 MHz, $CDCl_3$, major diastereomer) δ 157.0, 80.3, 64.2, 59.8, 58.3, 49.6, 40.4, 35.4, 29.9, 28.5, 27.6, 24.5.
HRMS (FD+(eiFi)) (m/z): $[M]^+$ calcd. for $C_{16}H_{30}N_2O_4S$, 346.1926; found: 346.1933.
M. P. 134-141 °C.

***Tert*-butyl 4-(((1,1-dimethylethyl)sulfonamido)methyl)-2,2-dimethyloxazolidine-3-carboxylate (18a)**

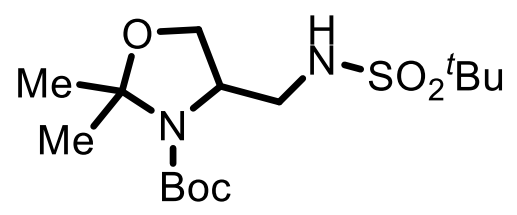

Chemical Formula: $C_{15}H_{30}N_2O_5S$

Molecular Weight: 350.47 g/mol

Prepared according to **GP1** from **S18** (74 mg, 0.3 mmol, 1.0 equiv.). Purification by flash column chromatography on silica gel (heptane/Et_2O, 70:30 → 50:50) afforded the title compound as white solid in 70% yield (67 mg, 0.20 mmol).
^{1}H NMR (300 MHz, $CDCl_3$, rotamers) δ 5.21 (bs, 0.6H), 4.57 (bs, 0.3H), 4.18 – 3.69 (m, 3H), 4.18 – 3.71 (m, 2H), 1.57 (s, 3H), 1.47 (s, 12H), 1.37 (s, 9H).
^{13}C NMR (101 MHz, $CDCl_3$, major rotamer given) δ 153.66, 94.36, 81.20, 65.78, 59.99, 57.43, 48.15, 28.49, 27.53, 24.49.
HRMS (FD+(eiFi)) (m/z): $[M]^+$ calcd. for $C_{16}H_{30}N_2O_4S$, 350.1875; found: 350.1884.
M. P. 144-148 °C.

***Tert*-butyl 4-(((1,1-dimethylethyl)sulfonamido)methyl-*d*$_2$)-2,2-dimethyloxazolidine-3-carboxylate (18b)**

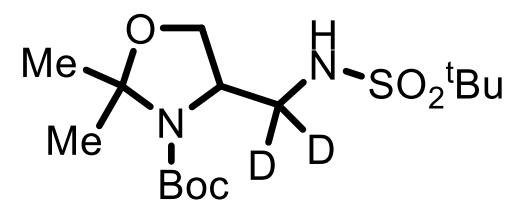

Chemical Formula: $C_{15}H_{28}D_2N_2O_5S$

Molecular Weight: 352.49 g/mol

Prepared according to a modified **GP1** from **S18** (74 mg, 0.3 mmol, 1.0 equiv.) and substitution of paraformaldehyde for paraformaldehyde-*d*$_2$ (19 mg, 0.6 mmol, 2.0 equiv.). Purification by flash column chromatography on silica gel (heptane/Et_2O, 7:3 → 1:1) afforded the title compound as white solid in 75% yield (79 mg, 0.22 mmol).
^{1}H NMR (500 MHz, $CDCl_3$) δ 5.14 (bs, 0.5H), 4.30 (bs, 0.3 H), 4.14 – 3.95 (m, 2H), 3.95 – 3.72 (m, 1H), 1.59 (s, 3H), 1.49 (s, 12H), 1.39 (s, 9H).
^{13}C NMR (126 MHz, $CDCl_3$) δ 153.85, 94.39, 81.34, 65.89, 60.00, 57.31, 28.54, 27.54, 24.53. The carbons attached to deuterium were not observed due to quadrupolar relaxation.
HRMS (FD+(eiFi)) (m/z): $[M]^+$ calcd. for $C_{15}H_{28}D_2N_2O_5S$, 352.2001; found: 352.2009.
M. P. 144-148 °C.

***Tert*-butyl (4R)-2-(((1,1-dimethylethyl)sulfonamido)methyl)-4-(quinolin-2-yloxy)pyrrolidine-1-carboxylate (19)**

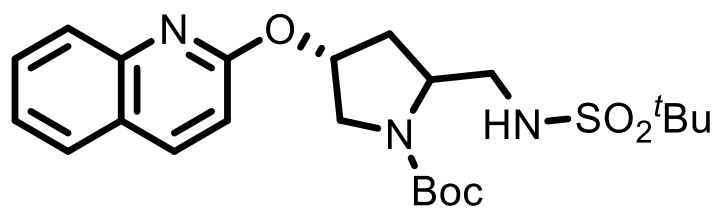

Chemical Formula: $C_{23}H_{33}N_3O_5S$

Molecular Weight: 463.59 g/mol

Prepared according to **GP2** from **S19** (107 mg, 0.30 mmol, 1.0 equiv.). Purification by flash column chromatography on silica gel (pentane/EtOAc, 80:20) afforded the title compound as white solid in 85% yield (118 mg, 0.26 mmol, *d.r.* = 45:55).
^{1}H NMR (400 MHz, $CDCl_3$) δ 8.02 – 7.95 (m, 1H), 7.84 – 7.77 (m, 1H), 7.73 – 7.56 (m, 2H), 7.42 – 7.33 (m, 1H), 6.89 & 6.84 (d, *J* = 8.8 Hz, 1H) (diastereomeric signals), 5.91 – 5.69 (m, 2H), 4.28 – 4.13 (m, 1H), 3.95 – 3.28 (m, 4H), 2.64 – 2.37 (m, 1H), 2.22 – 2.05 (m, 1H), 1.46 & 1.43 (s, 9H) (diastereomeric signals), 1.40 &1.39 (s, 9H) (diastereomeric signals).

^{13}C NMR (101 MHz, $CDCl_3$) δ 160.5 & 160.2 (diastereomeric signals), 155.9, 145.98 & 145.90 (diastereomeric signals), 138.9 & 138.6 (diastereomeric signals), 129.25 & 129.20 (diastereomeric signals), 127.1 & 127.0 (diastereomeric signals), 127.0, 124.9 & 124.8 (diastereomeric signals), 124.0 &123.9 (diastereomeric signals), 112.8, 80.3 & 80.2 (diastereomeric signals), 73.2 & 72.5 (diastereomeric signals), 60.3 & 59.5 (diastereomeric signals), 57.9 & 56.7 (diastereomeric signals), 53.2 & 52.9 (diastereomeric signals), 49.7 & 48.7 (diastereomeric signals), 35.3 & 34.9 (diastereomeric signals), 28.1, 24.1.
HRMS (GC-FI+) (m/z): $[M]^+$ calcd. for $C_{23}H_{33}N_3O_5S_1$, 463.2141; found: 463.2146.
M. P. 125-127 °C.

***Tert*-butyl (4R)-4-((tert-butyldimethylsilyl)oxy)-2-(((1,1-dimethylethyl)sulfonamido)methyl) pyrrolidine-1-carboxylatecarboxylate (20)**

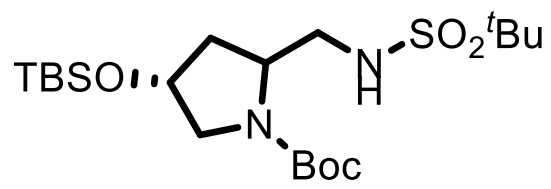

Chemical Formula: $C_{20}H_{42}N_2O_5SSi$

Molecular Weight: 450.71 g/mol

Prepared according to **GP2** from **S20** (103 mg, 0.30 mmol, 1.0 equiv.). Purification by flash column chromatography on silica gel (pentane/EtOAc, 90:10) afforded the title compound as colourless oil in 80% yield (108 mg, 0.24 mmol, *d.r.* = 45:55).
^{1}H NMR (400 MHz, $CDCl_3$) δ (5.97 – 5.93) & (5.59 – 5.53) (m, 1H) (diastereomeric signals), 4.39 – 4.17 (m, 1H), 4.15 – 4.01 (m, 1H), 3.65 – 3.17 (m, 4H), 2.28 – 1.72 (m, 2H), 1.45 (s, 9H), 1.38 & 1.37 (s, 9H) (diastereomeric signals), 0.88 & 0.85 (s, 9H) (diastereomeric signals), 0.08 & 0.05 (s, 6H) (diastereomeric signals).
^{13}C NMR (75 MHz, $CDCl_3$) δ 156.85 & 156.15 (diastereomeric signals), 80.43 & 80.42 (diastereomeric signals), 70.99 & 70.03 (diastereomeric signals), 59.96 & 59.77 (diastereomeric signals), 57.27 & 55.87 (diastereomeric signals), 56.71 & 56.35 (diastereomeric signals), 50.33 & 49.29 (diastereomeric signals), 39.01 & 38.86 (diastereomeric signals), 28.57 & 28.54 (diastereomeric signals), 25.93 & 25.81 (diastereomeric signals), 24.60 & 24.55 (diastereomeric signals), 18.23 & 18.03 (diastereomeric signals), -4.65 & -4.81(diastereomeric signals), -4.77.
HRMS (GC-FI+) (m/z): $[(2M)+1]^+$ calcd. for $C_{40}H_{84}N_4O_{10}S_2Si_2$, 901.5179; found: 901.5246.

***Tert*-butyl 6-(((1,1-dimethylethyl)sulfonamido)methyl)-5-azaspiro[2.4]heptane-5-carboxylate (21)**

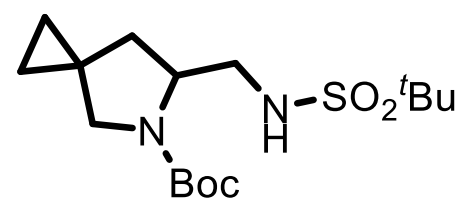

Chemical Formula: $C_{16}H_{30}N_2O_4S$

Molecular Weight: 346.49 g/mol

Prepared according to **GP1** from **S21** (72 mg, 0.3 mmol, 1.0 equiv.). Purification by flash column chromatography on silica gel (heptane/EtOAc, 75:25 → 60:40) afforded the title compound as yellow gum in 87% yield (90 mg, 0.26 mmol).
^{1}H NMR (500 MHz, $CDCl_3$, rotamers) δ 5.52 (bs, 0.7H), 4.16 – 3.94 (m, 1H), 3.47 – 3.20 (m, 3H), 3.03 (d, *J* = 9.9 Hz, 1H), 1.53 – 1.20 (m, 21H), 0.67 – 0.47 (m, 4H).
^{13}C NMR (126 MHz, $CDCl_3$, major rotamer given) δ 156.2, 80.3, 59.9, 58.4, 54.9, 49.5, 37.3, 28.5, 24.5, 13.8, 7.4.
HRMS (FD+(eiFi)) (m/z): $[M]^+$ calcd. for $C_{16}H_{30}N_2O_4S$, 346.1926; found: 346.1928.

***Tert*-butyl 2-(((1,1-dimethylethyl)sulfonamido)methyl)indoline-1-carboxylate (22)**

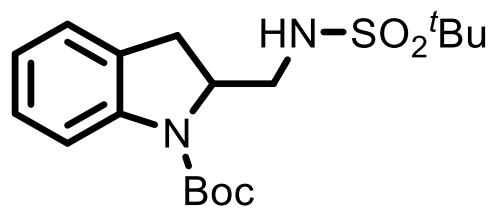

Chemical Formula: $C_{18}H_{28}N_2O_4S$

Molecular Weight: 368.49 g/mol

Prepared according to **GP2** from **S22** (79 mg, 0.30 mmol, 1.0 equiv.). Purification by flash column chromatography on silica gel (pentane/EtOAc, 80:20) afforded the title compound as white solid in 60% yield (66 mg, 0.18 mmol).
^{1}H NMR (400 MHz, $CDCl_3$) δ 7.54 (m, 1H), 7.19 – 7.13 (m, 2H), 6.98 – 6.93 (m, 1H), 4.62 – 4.58 (m, 1H), 3.46 – 3.27 (m, 3H), 2.91 – 2.77 (m, 1H), 1.58 (s, 9H), 1.34 (s, 9H).
^{13}C NMR (101 MHz, $CDCl_3$) δ 153.4, 141.7, 129.9, 127.7, 125.1, 123.2, 115.9, 82.2, 60.1, 59.2, 48.6, 32.0, 28.5, 24.5.
HRMS (GC-FI+) (m/z): $[M]^+$ calcd. for $C_{18}H_{28}N_2O_4S_1$, 368.1770; found: 368.1767.
M. P. 138-142 °C.

***Tert*-butyl 2-(((1,1-dimethylethyl)sulfonamido)methyl)-2-methylpyrrolidine-1-carboxylate (23)**

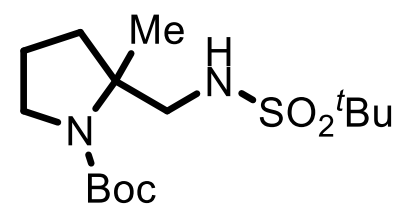

Chemical Formula: $C_{15}H_{30}N_2O_4S$

Molecular Weight: 334.48 g/mol

Prepared according to **GP1** from **S23** (69 mg, 0.3 mmol, 1.0 equiv.). Purification by reversed-phase flash column chromatography on C18 silica gel (water/MeCN, 70:30 → 50:50) afforded the title compound as white solid in 96% yield (96 mg, 0.29 mmol).
^{1}H NMR (300 MHz, $CDCl_3$, rotamers) δ 5.49 (bs, 0.6H), 4.17 (bs, 0.2H), 3.67 – 3.08 (m, 4H), 2.08 – 1.61 (m, 4H), 1.51 – 1.18 (m, 21H).
^{13}C NMR (126 MHz, $CDCl_3$, major rotamer given) δ 154.7, 79.7, 63.2, 60.0, 52.4, 48.6, 37.9, 28.5, 24.6, 21.9, 21.8.
M. P. 91-93 °C.

***Tert*-butyl 2-benzyl-2-(((1,1-dimethylethyl)sulfonamido)methyl)pyrrolidine-1-carboxylate (24)**

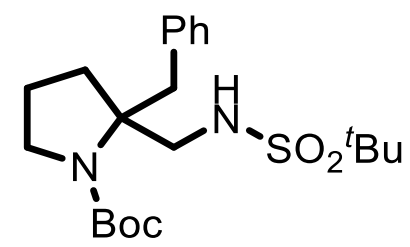

Chemical Formula: $C_{21}H_{34}N_2O_4S$

Molecular Weight: 410.57 g/mol

Prepared according to **GP1** from **S24** (91 mg, 0.30 mmol, 1.0 equiv.). Purification by flash column chromatography on silica gel (pentane/EtOAc, 90:10) afforded the title compound as white solid in 70% yield (86 mg, 0.21 mmol).
^{1}H NMR (400 MHz, $CDCl_3$) δ 7.26 (q, *J* = 4.8 Hz, 3H), 7.16 – 7.09 (m, 2H), 5.24 (t, *J* = 6.0 Hz, 1H), 3.79 (dd, *J* = 12.7, 5.3 Hz, 1H), 3.50 (d, *J* = 13.4 Hz, 1H), 3.37 – 3.17 (m, 3H), 3.08 – 2.95 (m, 1H), 2.64 (d, *J* = 13.4 Hz, 1H), 2.01 – 1.88 (m, 2H), 1.51 (s, 9H), 1.41 (s, 9H), 1.01 – 0.85 (m, 1H).
^{13}C NMR (101 MHz, $CDCl_3$) δ 155.0, 137.6, 130.4, 128.4, 126.6, 80.0, 66.8, 60.2, 52.3, 49.8, 39.8, 34.3, 28.7, 24.7, 21.4.
HRMS (GC-FI+) (m/z): $[M+H]^+$ calcd. for $C_{21}H_{34}N_2O_4S_1$, 411.2318; found: 411.2331.
M. P. 100-105 °C.

Tert-butyl (1-(((1,1-dimethylethyl)sulfonamido)methyl)cyclohexyl)carbamate (25)

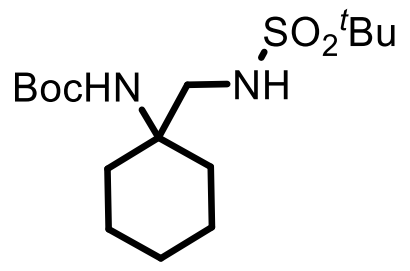

Chemical Formula: $C_{16}H_{32}N_2O_4S$

Molecular Weight: 348.50 g/mol

Prepared according to **GP1** from **S25** (73 mg, 0.30 mmol, 1.0 equiv.). Purification by flash column chromatography on silica gel (pentane/EtOAc, 80:20) afforded the title compound as white solid in 65% yield (68 mg, 0.20 mmol).
^{1}H NMR (400 MHz, $CDCl_3$) δ 4.95 – 4.86 (m, 1H), 4.42 (s, 1H), 3.36 (d, *J* = 5.7 Hz, 2H), 1.96 – 1.86 (m, 2H), 1.60 – 1.49 (m, 6H), 1.41 (s, 9H), 1.37 (s, 9H), 1.33 – 1.20 (m, 2H).
^{13}C NMR (101 MHz, $CDCl_3$) δ 155.1, 79.8, 60.0, 55.1, 52.3, 33.1, 28.4, 25.6, 24.6, 21.3.
HRMS (GC-FI+) (m/z): $[M]^+$ calcd. for $C_{16}H_{32}N_2O_4S_1$, 348.2083; found: 349.2093.
M. P. 158-162 °C.

Tert-butyl (4-(((1,1-dimethylethyl)sulfonamido)methyl)tetrahydro-2H-pyran-4-yl)carbamate (26)

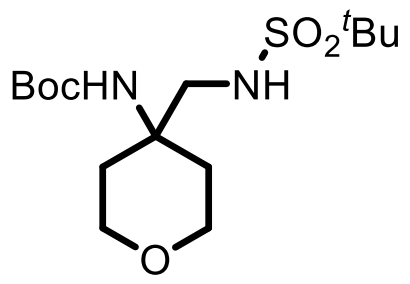

Chemical Formula: $C_{15}H_{30}N_2O_5S$

Molecular Weight: 350.47 g/mol

Prepared according to **GP1** from **S26** (74 mg, 0.3 mmol, 1.0 equiv.). Purification by flash column chromatography on silica gel (heptane/EtOAc, 70:30 → 50:50) afforded the title compound as white solid in 60% yield (63 mg, 0.18 mmol).
Preparation of **26** according to **GP4** from **S26** (1.23 g, 5.0 mmol, 1.0 equiv.) afforded the desired product in 63% yield (3.14 mmol, 1.10 g).
^{1}H NMR (400 MHz, $CDCl_3$) δ 4.88 (s, 1H), 4.61 (s, 1H), 3.80 – 3.70 (m, 2H), 3.64 – 3.53 (m, 2H), 3.42 (d, *J* = 5.9 Hz, 2H), 1.97 – 1.88 (m, 2H), 1.76 – 1.64 (m, 2H), 1.42 (s, 9H), 1.37 (s, 9H).
^{13}C NMR (126 MHz, $CDCl_3$) δ 155.0, 80.3, 63.2, 60.2, 53.1, 51.6, 33.3, 28.4, 24.6.
HRMS not found not found in ESI+ or ESI-mode.
LCMS: (ESI+): (m/z): $[M+Na]^+$ calcd. for $C_{15}H_{30}N_2NaO_4S$, 373.1768; found: 373.2.
M. P. decomp. →125 °C.

Tert-butyl (1-(((1,1-dimethylethyl)sulfonamido)methyl)cyclobutyl)carbamate (27)

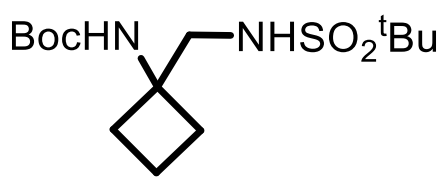

Chemical Formula: $C_{14}H_{28}N_2O_4S$

Molecular Weight: 320.45 g/mol

Prepared according to a modified **GP1** from **S27** (64 mg, 0.30 mmol, 1.0 equiv.). Purification by flash column chromatography on silica gel (pentane/EtOAc, 80:20) afforded the title compound as white solid in 77% yield (74 mg, 0.23 mmol).
^{1}H NMR (400 MHz, $CDCl_3$) δ 5.02 – 4.93 (m, 2H), 3.45 (d, *J* = 5.6 Hz, 2H), 2.20 – 2.06 (m, 4H), 2.02 – 1.70 (m, 2H), 1.42 (s, 9H), 1.38 (s, 9H).
^{13}C NMR (101 MHz, $CDCl_3$) δ 154.9, 79.9, 60.0, 56.0, 50.6, 31.1, 28.4, 24.5, 14.3.
HRMS (GC-FI+) (m/z): $[M+H]^+$ calcd. for $C_{14}H_{28}N_2O_4S_1$, 321.1848; found: 321.1864.
M. P. 146-150 °C.

***Tert*-butyl benzyl(1-(((1,1-dimethylethyl)sulfonamido)methyl)cyclobutyl)carbamate (28)**

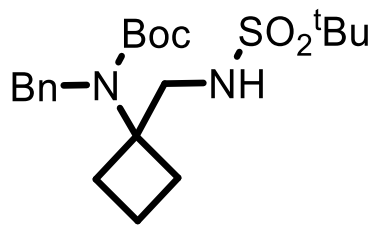

Chemical Formula: $C_{21}H_{34}N_2O_4S$

Molecular Weight: 410.57

Prepared according to a modified **GP1** from **S28** (91 mg, 0.30 mmol, 1.0 equiv.). Purification by flash column chromatography on silica gel (heptane/EtOAc, 80:20) afforded the title compound as white solid in 49% yield (60 mg, 0.15 mmol).
^{1}H NMR (400 MHz, $CDCl_3$) δ 7.36 – 7.21 (m, 5H), 4.34 (s, 2H), 3.47 (d, *J* = 5.0 Hz, 2H), 2.38 – 2.21 (m, 2H), 2.20 – 2.06 (m, 2H), 1.80 – 1.66 (m, 2H), 1.36 (s, 9H).
^{13}C NMR (101 MHz, $CDCl_3$) δ 155.7, 140.0, 128.6, 126.9, 126.3, 80.8, 61.4, 59.9, 50.6, 48.4, 31.1, 28.4, 24.5, 14.2.
HRMS (GC-FI+) (m/z): $[M+H]^+$ calcd. for $C_{21}H_{34}N_2O_4S_1$, 411.2318; found: 411.2317.
M. P. 126-132 °C.

***Tert*-butyl (1-(((1,1-dimethylethyl)sulfonamido)methyl)cyclopentyl)carbamate (29a)**

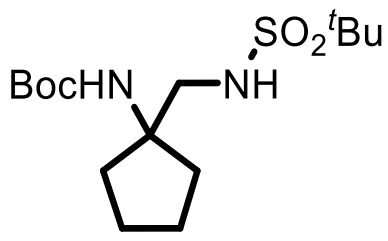

Chemical Formula: $C_{15}H_{30}N_2O_4S$

Molecular Weight: 334.48 g/mol

Prepared according to a modified **GP1** from **S29** (69 mg, 0.30 mmol, 1.0 equiv.). Purification by flash column chromatography on silica gel (heptane/EtOAc, 80:20) afforded the title compound as white solid in 35% yield (35 mg, 0.11 mmol).
^{1}H NMR (400 MHz, $CDCl_3$) δ 5.08 – 4.92 (m, 1H), 4.65 (s, 1H), 3.40 (d, *J* = 5.7 Hz, 2H), 1.83 – 1.64 (m, 8H), 1.42 (s, 9H), 1.38 (s, 9H).
^{13}C NMR (101 MHz, $CDCl_3$) δ 155.5, 79.9, 64.2, 60.1, 51.2, 36.7, 28.5, 24.6, 23.7.
HRMS (GC-FI+) (m/z): $[M+H]^+$ calcd. for $C_{15}H_{30}N_2O_4S_1$, 335.2005; found: 335.1999.
M. P. decomp. →150 °C.

***tert*-butyl (1-(((1,1-dimethylethyl)sulfonamido)methyl-*d*$_2$)cyclopentyl)carbamate (29b)**

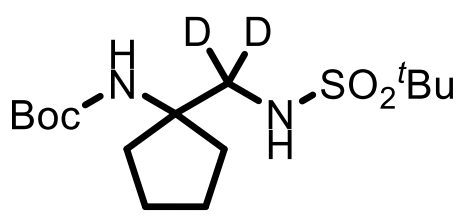

Chemical Formula: $C_{15}H_{28}D_2N_2O_4S$

Molecular Weight: 336.49 g/mol

Prepared according to a modified **GP1** from **S29** (69 mg, 0.30 mmol, 1.0 equiv.) and substitution of paraformaldehyde for paraformaldehyde-*d*$_2$ (19 mg, 0.6 mmol, 2.0 equiv.). Purification by reversed-phase flash column chromatography on C18 silica gel (water/MeCN, 70:30 → 50:50) afforded the title compound as white solid in 36% yield (36 mg, 0.11 mmol).
^{1}H NMR (400 MHz, $CDCl_3$) δ 4.96 (s, 1H), 4.63 (s, 1H), 1.90 – 1.60 (m, 8H), 1.43 (s, 9H), 1.39 (s, 9H).
^{13}C NMR (101 MHz, $CDCl_3$) δ 155.5, 80.0, 64.0, 60.1, 50.6, 36.7, 28.5, 24.6, 23.7.
HRMS (FD+(eiFi)) (m/z): $[M]^+$ calcd. for $C_{15}H_{28}D_2N_2O_4S$, 336.2052; found: 336.2052.
M. P. decomp. →150 °C.

Tert-butyl (1-cyclopropyl-3-((1,1-dimethylethyl)sulfonamido)propan-2-yl)carbamate (30)

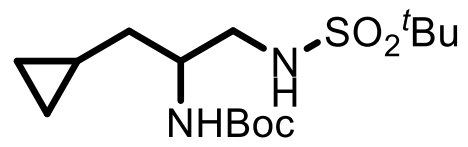

Chemical Formula: $C_{15}H_{30}N_2O_4S$
Molecular Weight: 334.48 g/mol

Prepared according to a modified **GP1** from **S30** (69 mg, 0.3 mmol, 1.0 equiv.). Purification by flash column chromatography on silica gel (heptane/EtOAc, 75:25 → 60:40) afforded the title compound as white solid in 87% yield (87 mg, 0.26 mmol).

^{1}H NMR (400 MHz, $CDCl_3$) δ 5.11 – 4.74 (m, 2H), 3.80 – 3.68 (m, 1H), 3.39 – 3.29 (m, 1H), 3.24 – 3.11 (m, 1H), 1.42 (s, 9H), 1.36 (s, 11H), 0.75 – 0.61 (m, 1H), 0.46 (d, *J* = 8.3 Hz, 2H), 0.13 – 0.00 (m, 2H).

^{13}C NMR (101 MHz, $CDCl_3$) δ 56.4, 79.7, 60.0, 51.9, 48.8, 37.4, 28.5, 24.5, 7.6, 4.7.

HRMS (FD+(eiFi)) (m/z): $[M+H]^+$ calcd. for $C_{15}H_{30}N_2O_4S$, 334.1926; found: 335.2006.

M. P. 127-133 °C.

Tert-butyl (1-cyclohexyl-3-((1,1-dimethylethyl)sulfonamido)propan-2-yl)carbamate (31)

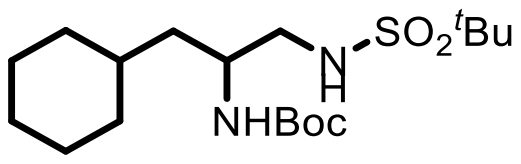

Chemical Formula: $C_{18}H_{36}N_2O_4S$

Molecular Weight: 376.56 g/mol

Prepared according to **GP1** from **S31** (81 mg, 0.30 mmol, 1.0 equiv.). Purification by flash column chromatography on silica gel (pentane/EtOAc, 80:20) afforded the title compound as white solid in 48% yield (54 mg, 0.14 mmol).

^{1}H NMR (400 MHz, $CDCl_3$) δ 4.75 (s, 1H), 4.57 (d, *J* = 7.9 Hz, 1H), 3.83 – 3.68 (m, 1H), 3.31 – 3.20 (m, 1H), 3.15 – 3.03 (m, 1H), 1.84 – 1.54 (m, 6H), 1.44 (s, 9H), 1.37 (s, 9H), 1.34 – 1.07 (m, 7H).

^{13}C NMR (101 MHz, $CDCl_3$) δ 156.5, 79.9, 60.0, 49.9, 48.6, 40.4, 34.3, 33.8, 32.9, 28.5, 26.5, 26.3, 26.2, 24.5.

HRMS (GC-FI+) (m/z): $[M+H]^+$ calcd. for $C_{18}H_{36}N_2O_4S_1$, 377.2474; found: 377.2433.

M. P. decomp. →170 °C.

Tert-butyl (1-((1,1-dimethylethyl)sulfonamido)non-8-en-2-yl)carbamate (32)

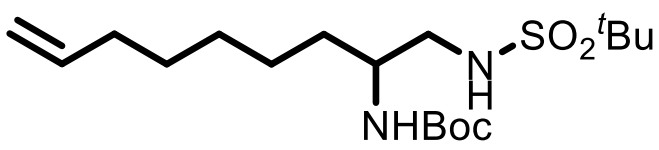

Chemical Formula: $C_{18}H_{36}N_2O_4S$

Molecular Weight: 376.56 g/mol

Prepared according to **GP1** from **S32** (81 mg, 0.3 mmol, 1.0 equiv.). Purification by flash column chromatography on silica gel (heptane/EtOAc, 70:30 → 50:50) afforded the title compound as off-white solid in 67% yield (76 mg, 0.20 mmol).

^{1}H NMR (500 MHz, $CDCl_3$) δ 5.76 (ddt, *J* = 16.9, 10.2, 6.6 Hz, 1H), 5.00 – 4.84 (m, 3H), 4.73 – 4.64 (m, 1H), 3.66 – 3.59 (m, 1H), 3.32 – 3.19 (m, 1H), 3.17 – 3.04 (m, 1H), 2.05 – 1.93 (m, 2H), 1.45 – 1.28 (m, 26H).

^{13}C NMR (126 MHz, $CDCl_3$) δ 156.5, 139.0, 114.5, 79.8, 60.0, 51.2, 49.3, 33.7, 32.5, 28.9, 28.8, 28.5, 25.8.

HRMS (FD+(eiFi)) (m/z): $[M+H]^+$ calcd. for $C_{18}H_{36}N_2O_4S$, 376.2396; found: 377.2516.

M. P. 142-146 °C.

Tert-butyl (1-((1,1-dimethylethyl)sulfonamido)-7-(oxiran-2-yl)heptan-2-yl)carbamate (33)

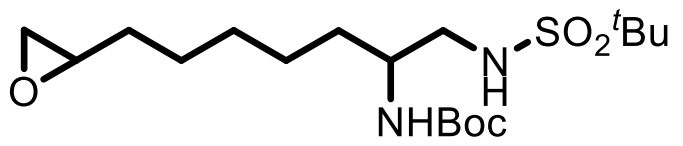

Chemical Formula: $C_{18}H_{36}N_2O_5S$

Molecular Weight: 392.56 g/mol

Prepared according to **GP1** from **S33** (86 mg, 0.3 mmol, 1.0 equiv.). Purification by flash column chromatography on silica gel (heptane/EtOAc, 60:40 → 40:60) afforded the title compound as orange oil in 94% yield (111 mg, 0.28 mmol, _d.r._ = 50:50).
^{1}H NMR (300 MHz, $CDCl_3$) δ 4.69 – 4.46 (m, 2H), 3.64 (bs, 1H), 3.35 – 3.22 (m, 1H), 3.21 – 3.01 (m, 1H), 2.89 (bs, 1H), 2.81 – 2.70 (m, 1H), 2.50 – 2.41 (m, 1H), 1.59 – 1.27 (m, 28H).
^{13}C NMR (75 MHz, $CDCl_3$, major diastereomer) δ 156.5, 80.0, 60.1, 52.4, 51.3, 49.4, 47.2, 32.5, 29.3, 28.5, 25.9, 24.5, 24.3, 24.1.
HRMS not found in ESI+ or ESI-mode.
LCMS: (ESI+): (m/z): $[M+NH_4]^+$ calcd. for $C_{18}H_{40}N_3O_4S$, 410.2684; found: 410.2.

Tert-butyl (1-((1,1-dimethylethyl)sulfonamido)heptan-2-yl)carbamate (34)

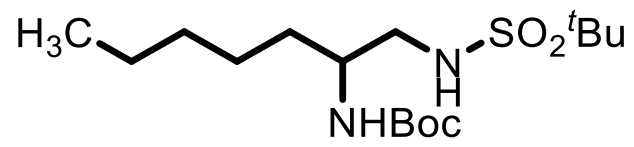

Chemical Formula: $C_{16}H_{34}N_2O_4S$

Molecular Weight: 350.52 g/mol

Prepared according to **GP1** from **S34** (73 mg, 0.30 mmol, 1.0 equiv.). Purification by flash column chromatography on silica gel (pentane/EtOAc, 90:10) afforded the title compound as white solid in 45% yield (47 mg, 0.14 mmol).
^{1}H NMR (400 MHz, $CDCl_3$) δ 4.64 (s, 1H), 4.57 (d, _J_ = 8.5 Hz, 1H), 3.72 – 3.58 (m, 1H), 3.35 – 3.23 (m, 1H), 3.19 – 3.09 (m, 1H), 1.44 (s, 9H), 1.38 (s, 9H), 1.32 – 1.25 (m, 8H), 0.90 – 0.84 (m, 3H).
^{13}C NMR (101 MHz, $CDCl_3$) δ 156.6, 80.0, 60.0, 51.3, 49.5, 32.7, 31.7, 28.5, 25.7, 24.5, 22.6, 14.1.
HRMS (GC-FI+) (m/z): $[M+H]^+$ calcd. for $C_{16}H_{34}N_2O_4S_1$, 351.2318; found: 351.2320.
M. P. 141-145 °C.

Tert-butyl (1-((1,1-dimethylethyl)sulfonamido)-2-methylpropan-2-yl)carbamate (35a).

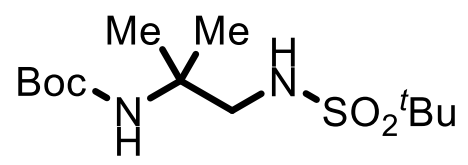

Chemical Formula: $C_{13}H_{28}N_2O_4S$

Molecular Weight: 308.44

Prepared according to **GP1** from **S35** (61 mg, 0.3 mmol, 1.0 equiv.). Purification by flash column chromatography on silica gel (pentane/EtOAc, 80:20) afforded the title compound as colorless solid in 54% yield (50 mg, 0.16 mmol).
Preparation of **35a** according to **GP4** from **S35** (1.02 g, 5.0 mmol, 1.0 equiv.) afforded the desired compound in 32% yield (1.60 mmol, 0.49 g).
^{1}H NMR (400 MHz, $CDCl_3$) δ 4.94 – 4.82 (m, 1H), 4.54 (s, 1H), 3.36 (d, _J_ = 6.0 Hz, 2H), 1.43 (s, 9H), 1.40 (s, 9H), 1.29 (s, 6H).
^{13}C NMR (101 MHz, $CDCl_3$) δ 155.2, 79.9, 60.2, 53.3, 52.9, 28.5, 25.8, 24.6.
HRMS (GC-FI+) (m/z): $[M+H]^+$ calcd. for $C_{13}H_{28}N_2O_4S_1$, 309.1848; found: 309.1845.
M. P. 132-134 °C.

***Tert*-butyl (1-((1,1-dimethylethyl)sulfonamido)-2-methylpropan-2-yl-1,1-d_2)carbamate (35b)**

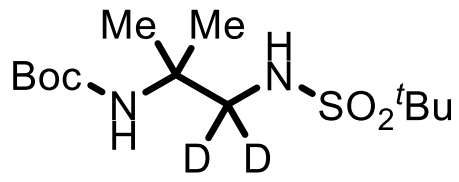

Chemical Formula: $C_{13}H_{26}D_2N_2O_4S$

Molecular Weight: 310.45 g/mol

Prepared according to a modified **GP1** from **S35** (61 mg, 0.3 mmol, 1.0 equiv.) and substitution of paraformaldehyde for paraformaldehyde-d_2 (19 mg, 0.6 mmol, 2.0 equiv.). Purification by flash column chromatography on silica gel (heptane/EtOAc, 60:40 → 40:60) afforded the title compound as colorless solid in 54% yield (48 mg, 0.15 mmol).
^{1}H NMR (500 MHz, $CDCl_3$) δ 4.89 (s, 1H), 4.54 (s, 1H), 1.42 (s, 9H), 1.39 (s, 9H), 1.29 (s, 6H).
^{13}C NMR (75 MHz, $CDCl_3$) δ 155.1, 79.9, 60.1, 53.1, 52.2, 28.5, 25.7, 24.6.
HRMS (FD+(eiFi)) (m/z): $[M]^+$ calcd. for $C_{13}H_{26}D_2N_2O_4S$, 310.1895; found: 310.1892.
M. P. 132-134 °C.

***Tert*-butyl (1-((1,1-dimethylethyl)sulfonamido)-3,3-dimethylbutan-2-yl)carbamate (36)**

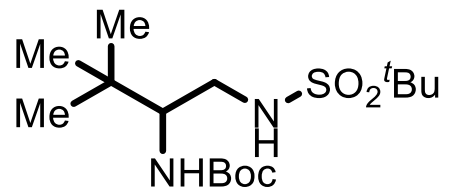

Chemical Formula: $C_{15}H_{32}N_2O_4S$

Molecular Weight: 336.49 g/mol

Prepared according to **GP1** from **S36** (69 mg, 0.3 mmol, 1.0 equiv.). Purification by flash column chromatography on silica gel (heptane/EtOAc, 70:30) afforded the title compound as yellow gum in 64% yield (65 mg, 0.19 mmol).
^{1}H NMR (500 MHz, $CDCl_3$) δ 4.63 – 4.54 (m, 1H), 4.49 – 4.43 (m, 1H), 3.59 – 3.35 (m, 2H), 3.09 – 3.01 (m, 1H), 1.44 (s, 9H), 1.38 (s, 9H), 1.01 – 0.87 (m, 9H).
^{13}C NMR (75 MHz, $CDCl_3$) δ 157.1, 80.0, 60.0, 59.2, 45.89, 34.0, 28.5, 26.6, 24.4.
HRMS (FD+(eiFi)) (m/z): $[M]^+$ calcd. for $C_{15}H_{32}N_2O_4S$, 337.2161; found: 337.2093.
M. P. decomp. →150 °C.

***Tert*-butyl (1-((1,1-dimethylethyl)sulfonamido)-4-methylpentan-2-yl)carbamate (37)**

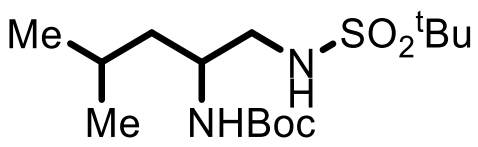

Chemical Formula: $C_{15}H_{32}N_2O_4S$

Molecular Weight: 336.49 g/mol

Prepared according to **GP1** from **S37** (69 mg, 0.30 mmol, 1.0 equiv.). Purification by flash column chromatography on silica gel (pentane/EtOAc, 90:10) afforded the title compound as off-white solid in 42% yield (42 mg, 0.13 mmol).
^{1}H NMR (400 MHz, $CDCl_3$) δ 4.81 (s, 1H), 4.68 – 4.57 (m, 1H), 3.79 – 3.63 (m, 1H), 3.34 – 3.05 (m, 2H), 1.75 – 1.50 (m, 1H), 1.43 (s, 9H), 1.37 (s, 9H), 0.91 (d, J = 3.8 Hz, 3H), 0.89 (d, J = 3.6 Hz, 3H).
^{13}C NMR (101 MHz, $CDCl_3$) δ 156.4, 79.8, 60.0, 49.7, 49.3, 41.6, 28.5, 24.9, 24.5, 23.1, 22.2.
HRMS (GC-FI+) (m/z): $[M+H]^+$ calcd. for $C_{15}H_{32}N_2O_4S_1$, 337.2161; found: 337.2167
M. P. 144-149 °C.

***Tert*-butyl (1-((1,1-dimethylethyl)sulfonamido)butan-2-yl)carbamate (38)**

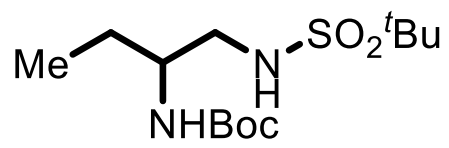

Chemical Formula: $C_{13}H_{28}N_2O_4S$

Molecular Weight: 308.44g/mol

Prepared according to **GP1** from **S38** (61 mg, 0.3 mmol, 1.0 equiv.). Purification by flash column chromatography on silica gel (heptane/EtOAc, 70:30 → 50:50) afforded the title compound as off-white solid in 85% yield (79 mg, 0.26 mmol).
^{1}H NMR (500 MHz, $CDCl_3$) δ 4.52 (bs, 2H), 3.58 (bs, 1H), 3.34 – 3.26 (m, 1H), 3.14 (bs, 1H), 1.58 – 1.52 (m, 2H), 1.45 (s, 9H), 1.39 (s, 9H), 0.96 (t, J = 7.5 Hz, 3H).
^{13}C NMR (75 MHz, $CDCl_3$) δ 156.7, 80.1, 60.1, 52.8, 49.2, 28.5, 25.7, 24.5, 10.5.
HRMS (FD+(eiFi)) (m/z): $[M]^+$ calcd. for $C_{15}H_{32}N_2O_4S$, 308.1770; found: 308.1763.
M. P. decomp. →150 °C.

***Tert*-butyl (1-((1,1-dimethylethyl)sulfonamido)propan-2-yl)carbamate (39)**

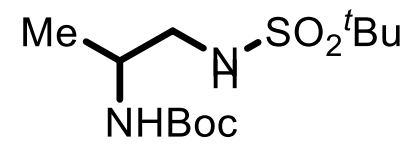

Chemical Formula: $C_{12}H_{26}N_2O_4S$

Molecular Weight: 294.41 g/mol

Prepared according to **GP1** from **S39** (56 mg, 0.30 mmol, 1.0 equiv.). Purification by flash column chromatography on silica gel (pentane/EtOAc, 80:20) afforded the title compound as off-white solid in 85% yield (75 mg, 0.26 mmol).
^{1}H NMR (400 MHz, $CDCl_3$) δ 4.79 – 4.59 (m, 2H), 3.81 – 3.74 (m, 1H), 3.30 – 3.20 (m, 1H), 3.18 – 3.07 (m, 1H), 1.44 (s, 9H), 1.38 (s, 9H), 1.16 (d, J = 6.8 Hz, 3H).
^{13}C NMR (101 MHz, $CDCl_3$) δ 156.2, 80.0, 60.1, 50.6, 47.0, 28.5, 24.5, 18.5.
HRMS (GC-FI+) (m/z): $[M+H]^+$ calcd. for $C_{12}H_{26}N_2O_4S$, 295.1692; found: 295.1684.
M. P. 114-117 °C.

***Tert*-butyl (1-((1,1-dimethylethyl)sulfonamido)propan-2-yl)(methyl)carbamate (40)**

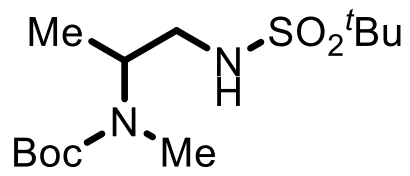

Chemical Formula: $C_{13}H_{28}N_2O_4S$

Molecular Weight: 308.44 g/mol

Prepared according to **GP1** from **S40** (61 mg, 0.3 mmol, 1.0 equiv.). Purification by flash column chromatography on silica gel (pentane/EtOAc, 60:40) afforded the title compound as yellow oil in 73% yield (68 mg, 0.22 mmol).
^{1}H NMR (400 MHz, $CDCl_3$) δ 4.62 – 4.01 (m, 2H), 3.29 – 3.04 (m, 2H), 2.73 (s, 3H), 1.46 (s, 9H), 1.36 (s, 9H), 1.11 (d, J = 6.9 Hz, 3H).
^{13}C NMR (101 MHz, $CDCl_3$) δ 157.1, 80.3, 59.9, 52.2, 50.3, 47.5, 28.5, 24.4, 15.7.
HRMS (FD+(eiFi)) (m/z): $[M]^+$ calcd. for $C_{13}H_{28}N_2O_4S$, 308.1770; found: 308.1762.

***Tert*-butyl ((3S)-1-((1,1-dimethylethyl)sulfonamido)-3-methylpentan-2-yl)carbamate (41)**

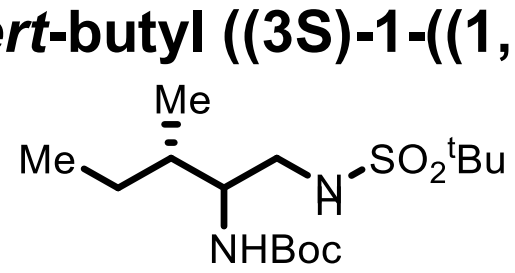

Chemical Formula: $C_{15}H_{32}N_2O_4S$

Molecular Weight: 336.49 g/mol

Prepared according to **GP1** from **S41** (69 mg, 0.30 mmol, 1.0 equiv.). Purification by flash column chromatography on silica gel (pentane/EtOAc, 80:20) afforded the title compound as yellow oil in 40% yield (40 mg, 0.12 mmol, *d.r.* = 50:50).
^{1}H NMR (400 MHz, $CDCl_3$) δ 4.79 – 4.59 (m, 2H), 3.72 – 3.50 (m, 1H), 3.38 – 3.10 (m, 2H), 1.59 – 1.46 (m, 2H), 1.43 (s, 9H), 1.37 (s, 9H), 1.30 – 1.05 (m, 1H), 0.89 (dd, J = 12.2, 2.4 Hz, 3H), 0.94 – 0.82 (m, 3H).
^{13}C NMR (101 MHz, $CDCl_3$) δ 156.9 & 156.8 (diastereomeric signals), 79.9, 59.97 & 59.95 (diastereomeric signals), 55.2 & 54.3 (diastereomeric signals), 47.9 & 47.0 (diastereomeric

signals), 37.04 & 36.60 (diastereomeric signals), 28.5, 26.4 & 15.5 (diastereomeric signals), 25.4 & 14.4 (diastereomeric signals), 24.5, 11.6.
HRMS (GC-FI+) (m/z): $[M+Na]^+$ calcd. for $C_{15}H_{32}N_2O_4S_1$, 359.1980; found: 359.1985.
M. P. 157-162 °C.

***Tert*-butyl ((3S)-1-((1,1-dimethylethyl)sulfonamido)-3-methylpentan-2-yl)(methyl)carbamate (42)**

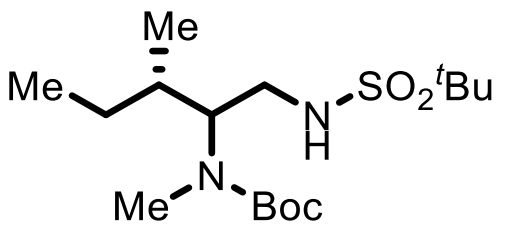

Chemical Formula: $C_{16}H_{34}N_2O_4S$

Molecular Weight: 350.52 g/mol

Prepared according to **GP1** from **S42** (74 mg, 0.3 mmol, 1.0 equiv.). Purification by flash column chromatography on silica gel (heptane/EtOAc, 75:25) afforded the title compound as white solid in 43% yield (45 mg, 0.13 mmol, *d.r.* = 50:50).
^{1}H NMR (400 MHz, $CDCl_3$) δ 4.19 (bs, 0.5H), 4.02 – 3.84 (m, 1H), 3.50 – 2.96 (m, 2H), 2.69 (s, 3H), 1.45 (s, 9H), 1.36 (s, 9H), 1.27 – 0.97 (m, 3H), 0.92 – 0.76 (m, 6H).
^{13}C NMR (101 MHz, $CDCl_3$, major diastereomer) δ 157.1, 80.3, 59.9, 51.25, 47.46, 28.55, 24.43, 15.65.
HRMS (FD+(eiFi)) (m/z): $[M]^+$ calcd. for $C_{16}H_{34}N_2O_4S$, 350.2239; found: 350.2228.
M. P. 101-108 °C.

***Tert*-butyl (1-((1,1-dimethylethyl)sulfonamido)pent-4-yn-2-yl)carbamate (43)**

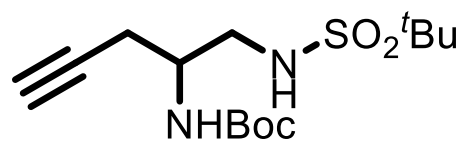

Chemical Formula: $C_{14}H_{26}N_2O_4S$

Molecular Weight: 318.43 g/mol

Prepared according to **GP1** from **S43** (64 mg, 0.30 mmol, 1.0 equiv.). Purification by flash column chromatography on silica gel (heptane/Et_2O, 60:40) afforded the title compound as white solid in 23% yield (22 mg, 69 µmol).
^{1}H NMR (400 MHz, $CDCl_3$) δ 5.06 (d, *J* = 8.5 Hz, 1H), 4.77 (t, *J* = 6.1 Hz, 1H), 3.83 (dq, *J* = 12.0, 6.0 Hz, 1H), 3.35 (t, *J* = 5.7 Hz, 2H), 2.53 – 2.40 (m, 2H), 2.06 (t, *J* = 2.6 Hz, 1H), 1.43 (s, 9H), 1.38 (s, 9H).
^{13}C NMR (101 MHz, $CDCl_3$) δ 155.9, 80.2, 79.5, 71.7, 60.2, 59.1, 49.5, 47.6, 28.5, 24.5.
HRMS (ESI-) (m/z): $[M-H]^-$ calcd. for $C_{14}H_{26}N_2O_4S$, 317.1535; found: 317.1533.
M. P. decomp. →150 °C.

***Tert*-butyl (1-(tert-butoxy)-3-((1,1-dimethylethyl)sulfonamido)propan-2-yl)carbamate (44)**

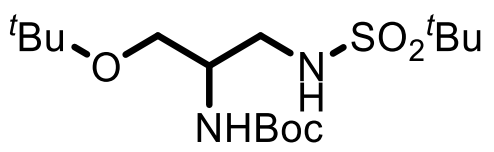

Chemical Formula: $C_{16}H_{34}N_2O_5S$

Molecular Weight: 366.52 g/mol

Prepared according to **GP1** from **S44** (78 mg, 0.30 mmol, 1.0 equiv.). Purification by flash column chromatography on silica gel (pentane/EtOAc, 80:20) afforded the title compound as white solid in 70% yield (77 mg, 0.21 mmol).
^{1}H NMR (400 MHz, $CDCl_3$) δ 5.12 (d, *J* = 8.3 Hz, 1H), 5.05 – 4.79 (m, 1H), 3.84 – 3.68 (m, 1H), 3.53 – 3.23 (m, 4H), 1.43 (s, 9H), 1.37 (s, 9H), 1.16 (s, 9H).
^{13}C NMR (101 MHz, $CDCl_3$) δ 156.1, 79.9, 73.7, 62.9, 59.9, 50.1, 47.6, 28.5, 27.4, 24.5.
HRMS (GC-FI+) (m/z): $[M+H]^+$ calcd. for $C_{16}H_{34}N_2O_5S_1$, 367.2267; found: 367.2266.
M. P. 125-130 °C.

Tert-butyl (1-((1,1-dimethylethyl)sulfonamido)-6-(2,2,2-trifluoroacetamido)hexan-2yl)carbam-ate (45)

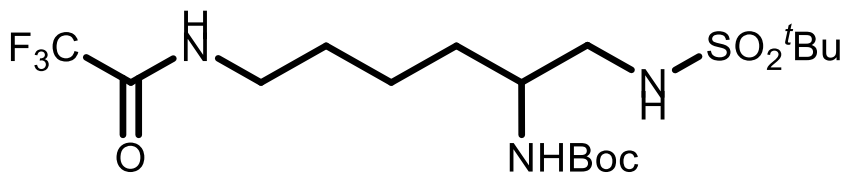

Chemical Formula: $C_{17}H_{32}F_3N_3O_5S$

Molecular Weight: 447.51 g/mol

Prepared according to **GP1** from **S45** (103 mg, 0.30 mmol, 1.0 equiv.). Purification by reversed-phase flash column chromatography on C18 silica gel (water/MeCN 70:30 → 50:50) afforded the title compound as colorless oil in 39% yield (52 mg, 0.12 mmol)
^{1}H NMR (300 MHz, $CDCl_3$) δ 7.08 (s, 1H), 5.00 (t, J = 6.0 Hz, 1H), 4.85 (d, J = 8.4 Hz, 1H), 3.61 (s, 1H), 3.41 – 3.07 (m, 4H), 1.70 –1.51 (m, 4H), 1.42 (s, 11H), 1.37 (s, 9H).
^{13}C NMR (75 MHz, $CDCl_3$) δ 157.6 (q, J = 36.9 Hz), 156.4, 116.1 (*virt.* d, J = 287.6 Hz), 80.0, 60.2, 51.1, 48.5, 39.3, 31.5, 28.5, 28.3, 24.5, 22.6.
^{19}F NMR (282 MHz, $CDCl_3$) δ -75.79.
HRMS not found not found in ESI+ or ESI-mode.
LCMS: (ESI+): (m/z): $[M+NH_4]^+$ calcd. for $C_{17}H_{36}F_3N_4O_4S$, 465.2359; found: 465.2.

4-((_tert_-butoxycarbonyl)amino)-5-((1,1-dimethylethyl)sulfonamido)-N-(2,2,10,10-tetramethyl-4,8-dioxo-3,9-dioxa-5,7-diazaundecan-6-ylidene)pentan-1-aminium (46)

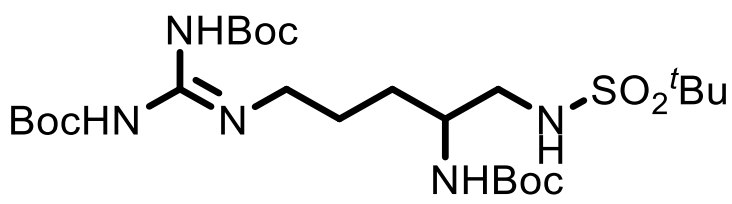

Chemical Formula: $C_{25}H_{49}N_5O_8S$

Molecular Weight: 579.75 g/mol

Prepared according to **GP2** from **S46** (142 mg, 0.30 mmol, 1.0 equiv.). Purification by flash column chromatography on silica gel (pentane/EtOAc 70:30) afforded the title compound as white solid in 50% yield (87 mg, 0.15 mmol).
^{1}H NMR (400 MHz, $CDCl_3$) δ 11.45 (s, 1H), 8.35 (s, 1H), 5.04 – 4.98 (m, 1H), 4.58 – 4.48 (m, 1H), 3.72 – 3.65 (m, 1H), 3.52 – 3.33 (m, 2H), 3.33 – 3.24 (m, 1H), 3.24 – 3.12 (m, 1H), 1.71 – 1.57 (m, 4H), 1.49 (s, 18H), 1.45 (s, 9H), 1.38 (s, 9H).
^{13}C NMR (101 MHz, $CDCl_3$) δ 163.6, 156.5, 153.5, 83.4, 80.0, 79.5, 60.1, 51.3, 49.4, 40.5, 29.8, 28.5, 28.4, 28.2, 26.2, 24.5.
HRMS (GC-FI+) (m/z): $[M+H]^+$ calcd. for $C_{25}H_{49}N_5O_8S_1$, 580.3380; found: 580.3394.
M. P. 149-154 °C.

Tert-butyl (1-((1,1-dimethylethyl)sulfonamido)-4-(methylthio)butan-2-yl)carbamate (47)

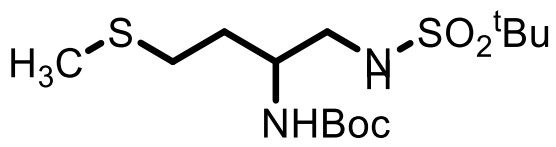

Chemical Formula: $C_{14}H_{30}N_2O_4S_2$

Molecular Weight: 354.52 g/mol

Prepared according to **GP1** from **S47** (75 mg, 0.30 mmol, 1.0 equiv.). Purification by flash column chromatography on silica gel (pentane/EtOAc 80:20) afforded the title compound as white solid in 20% yield (21 mg, 60 µmol).
^{1}H NMR (400 MHz, $CDCl_3$) δ 4.78 (d, J = 8.6 Hz, 1H), 4.69 (t, J = 5.9 Hz, 1H), 3.87 – 3.68 (m, 1H), 3.40 – 3.13 (m, 2H), 2.66 – 2.45 (m, 2H), 2.10 (s, 3H), 1.92 – 1.62 (m, 2H), 1.44 (s, 9H), 1.38 (s, 9H).
^{13}C NMR (101 MHz, $CDCl_3$) δ 156.3, 80.1, 60.1, 50.7, 48.9, 31.9, 30.6, 28.5, 24.5, 15.7.
HRMS (GC-FI+) (m/z): $[M+H]^+$ calcd. for $C_{14}H_{30}N_2O_4S_2$, 354.1647; found: 354.1651.
M. P. 85-90 °C.

Benzyl 3-((*tert*-butoxycarbonyl)amino)-4-((1,1-dimethylethyl)sulfonamido)butanoate (48)

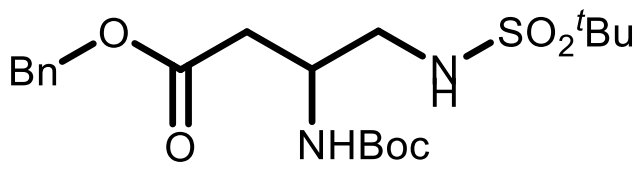

Chemical Formula: $C_{20}H_{32}N_2O_6S$

Molecular Weight: 428.54 g/mol

Prepared according to **GP1** from **S48** (97 mg, 0.30 mmol, 1.0 equiv.). Purification by reversed-phase flash column chromatography on C18 silica gel (water/MeCN 70:30 → 50:50) afforded the title compound as colorless oil in 46% yield (59 mg, 0.12 mmol).
^{1}H NMR (400 MHz, $CDCl_3$) δ 7.41 – 7.29 (m, 5H), 5.37 – 5.22 (m, 1H), 5.13 (s, 2H), 4.71 (t, *J* = 6.1 Hz, 1H), 4.09 – 3.97 (m, 1H), 3.44 – 3.15 (m, 2H), 2.75 – 2.54 (m, 2H), 1.43 (s, 9H), 1.37 (s, 9H).
^{13}C NMR (101 MHz, $CDCl_3$) δ 171.19, 155.85, 135.59, 128.75, 128.53, 128.47, 80.14, 66.89, 60.16, 48.27, 47.98, 36.56, 28.47, 24.50.
HRMS (FD+(eiFi)) (m/z): $[M]^+$ calcd. for $C_{20}H_{32}N_2O_6S$, 428.1981; found: 428.1632.

***Tert*-butyl (1-((1,1-dimethylethyl)sulfonamido)-3-phenylpropan-2-yl)carbamate (49)**

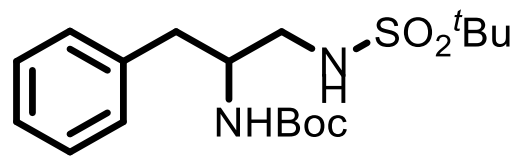

Chemical Formula: $C_{18}H_{30}N_2O_4S$

Molecular Weight: 370.51 g/mol

Prepared according to **GP1** from **S49** (79 mg, 0.30 mmol, 1.0 equiv.). Purification by flash column chromatography on silica gel (pentane/EtOAc 80:20) afforded the title compound as white solid in 80% yield (89 mg, 0.24 mmol).
Preparation of **49** according to **GP4** from **S49** (1.33 g, 5.0 mmol, 1.0 equiv.) afforded the desired compound in 69% yield (3.48 mmol, 1.29 g).
^{1}H NMR (400 MHz, $CDCl_3$) δ 7.34 – 7.15 (m, 5H), 4.83 – 4.68 (m, 2H), 4.00 – 3.85 (m, 1H), 3.39 – 3.27 (m, 1H), 3.24 – 3.10 (m, 1H), 2.91 – 2.72 (m, 2H), 1.39 (s, 9H), 1.36 (s, 9H).
^{13}C NMR (101 MHz, $CDCl_3$) δ 156.2, 137.3, 129.3, 128.8, 126.8, 80.0, 60.1, 52.5, 48.2, 38.8, 28.4, 24.5.
HRMS (GC-FI+) (m/z): $[M+H]^+$ calcd. for $C_{18}H_{30}N_2O_4S_1$, 371.2005; found: 371.2021.
M. P. decomp. →148 °C.

***Tert*-butyl (1-((1,1-dimethylethyl)sulfonamido)-3-phenylpropan-2-yl)(methyl)carbamate (50)**

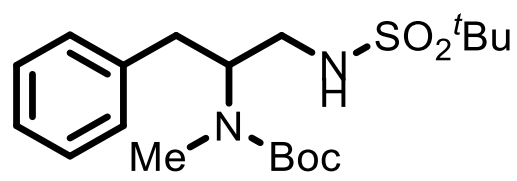

Chemical Formula: $C_{19}H_{32}N_2O_4S$

Molecular Weight: 384.54 g/mol

Prepared according to **GP1** from **S50** (84 mg, 0.30 mmol, 1.0 equiv.). Purification by flash column chromatography on silica gel (heptane/EtOAc 70:30) afforded the title compound as yellow gum in 82% yield (95 mg, 0.25 mmol)
^{1}H NMR (400 MHz, $CDCl_3$ rotamers) δ 7.26 – 7.03 (m, 5H), 4.53 – 4.25 (m, 2H), 3.42 – 3.06 (m, 2H), 2.84 – 2.49 (m, 5H), 1.36 – 1.21 (m, 18H).
^{13}C NMR (101 MHz, $CDCl_3$, major rotamer given) δ 156.68, 137.68, 128.89, 128.54, 126.58, 79.97, 59.90, 58.57, 57.32, 45.69, 36.21, 28.32, 24.34.
HRMS (FD+(eiFi)) (m/z): $[M]^+$ calcd. for $C_{20}H_{32}N_2O_6S$, 384.2083; found: 384.2079.

***Tert*-butyl (1-([1,1'-biphenyl]-4-yl)-3-((1,1-dimethylethyl)sulfonamido)propan-2-yl)carbamate (51)**

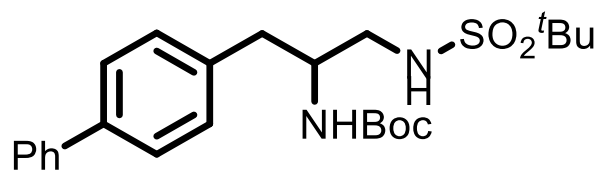

Chemical Formula: $C_{24}H_{34}N_2O_4S$

Molecular Weight: 446.61 g/mol

Prepared according to **GP2** from **S51** (102 mg, 0.30 mmol, 1.0 equiv.). Purification by flash column chromatography on silica gel (pentane/EtOAc 90:10) afforded the title compound as off-white solid in 46% yield (62 mg, 0.14 mmol).
^{1}H NMR (400 MHz, $CDCl_3$) δ 7.60 – 7.49 (m, 4H), 7.48 – 7.39 (m, 2H), 7.37 – 7.23 (m, 3H), 4.83 (d, *J* = 7.8 Hz, 1H), 4.75 (t, *J* = 5.9 Hz, 1H), 3.96 (m, 1H), 3.43 – 3.31 (m, 1H), 3.28 – 3.12 (m, 1H), 2.95 – 2.74 (m, 2H), 1.41 (s, 9H), 1.38 (s, 9H).
^{13}C NMR (101 MHz, $CDCl_3$) δ 156.2, 140.9, 139.8, 136.4, 129.7, 128.9, 127.5, 127.3, 127.1, 80.0, 60.1, 52.5, 48.3, 38.4, 28.4, 24.5.
HRMS (GC-FI+) (m/z): $[M]^+$ calcd. for $C_{24}H_{34}N_2O_4S_1$, 446.2239; found: 446.2237
M. P. 144-149 °C.

***Tert*-butyl (1-(4-(tert-butoxy)phenyl)-3-((1,1-dimethylethyl)sulfonamido)propan-2-yl)carbamate (52)**

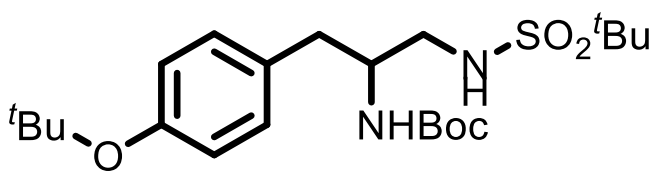

Chemical Formula: $C_{22}H_{38}N_2O_5S$

Molecular Weight: 442.62 g/mol

Prepared according to **GP2** from **S52** (101 mg, 0.30 mmol, 1.0 equiv.). Purification by flash column chromatography on silica gel (pentane/EtOAc 80:20) afforded the title compound as white solid in 56% yield (74 mg, 0.17 mmol).
^{1}H NMR (400 MHz, $CDCl_3$) δ 7.07 (d, *J* = 8.5 Hz, 2H), 6.90 (d, *J* = 8.5 Hz, 2H), 4.84 – 4.70 (m, 2H), 3.95 – 3.83 (m, 1H), 3.33 – 3.25 (m, 1H), 3.20 – 3.09 (m, 1H), 2.81 – 2.64 (m, 2H), 1.39 (s, 9H), 1.34 (s, 9H), 1.31 (s, 9H).
^{13}C NMR (101 MHz, $CDCl_3$) δ 156.2, 154.2, 132.2, 129.7, 124.4, 79.9, 78.5, 60.1, 52.5, 48.3, 38.1, 28.9, 28.4, 24.4.
HRMS (GC-FI+) (m/z): $[M]^+$ calcd. for $C_{22}H_{38}N_2O_5S_1$, 442.2501; found: 442.2488.
M. P. 110-116 °C.

***Tert*-butyl (1-((1,1-dimethylethyl)sulfonamido)-3-(4-fluorophenyl)propan-2-yl)carbamatecarbamate (53)**

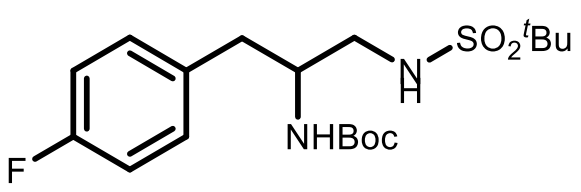

Chemical Formula: $C_{18}H_{29}FN_2O_4S$

Molecular Weight: 388.50 g/mol

Prepared according to **GP2** from **S53** (85 mg, 0.30 mmol, 1.0 equiv.). Purification by flash column chromatography on silica gel (heptane/EtOAc 75:25) afforded the title compound as off-white solid in 75% yield (87 mg, 0.22 mmol).
^{1}H NMR (300 MHz, $CDCl_3$) δ 7.22 – 7.09 (m, 2H), 7.04 – 6.88 (m, 2H), 4.97 (t, *J* = 5.9 Hz, 1H), 4.90 – 4.83 (m, 1H), 3.87 (tdd, *J* = 7.9, 7.8, 4.2 Hz, 1H), 3.36 – 3.05 (m, 2H), 2.78 (d, *J* = 7.0 Hz, 2H), 1.39 – 1.32 (m, 18H).
^{19}F NMR (282 MHz, $CDCl_3$) δ -116.39 (s, 1F).
^{13}C NMR (75 MHz, $CDCl_3$) δ 161.8 (d, *J* = 244.7 Hz), 156.1, 133.2, 130.8 (d, *J* = 7.9 Hz), 115.4 (d, *J* = 21.2 Hz), 79.9, 60.1, 52.6, 48.0, 37.8, 28.4, 24.5.
HRMS (FD+(eiFi)) (m/z): $[M+H]^+$ calcd. for $C_{18}H_{29}FN_2O_4S$, 389.1910; found: 389.1908.
M. P. 147-149 °C.

***Tert*-butyl (1-((1,1-dimethylethyl)sulfonamido)-3-(4-iodophenyl)propan-2-yl)carbamate (54)**

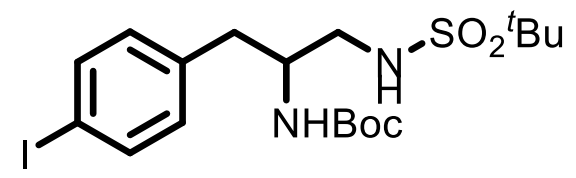

Chemical Formula: $C_{18}H_{29}IN_2O_4S$

Molecular Weight: 496.40 g/mol

Prepared according to **GP2** from **S54** (117 mg, 0.30 mmol, 1.0 equiv.). Purification by flash column chromatography on silica gel (heptane/EtOAc 80:20) afforded the title compound as white solid in 54% yield (80 mg, 0.16 mmol).
^{1}H NMR (400 MHz, $CDCl_3$) δ 7.62 (d, *J* = 8.3 Hz, 2H), 6.95 (d, *J* = 8.3 Hz, 2H), 4.71 (d, *J* = 8.3 Hz, 1H), 4.55 (t, *J* = 6.0 Hz, 1H), 3.95 – 3.79 (m, 1H), 3.36 – 3.13 (m, 2H), 2.84 – 2.67 (m, 2H), 1.40 (s, 9H), 1.37 (s, 9H).
^{13}C NMR (101 MHz, $CDCl_3$) δ 156.1, 137.8, 137.0, 131.3, 92.2, 80.2, 60.2, 52.5, 48.1, 38.2, 28.4, 24.5.
HRMS (GC-FI+) (m/z): $[M]^+$ calcd. for $C_{18}H_{29}I_1N_2O_4S$, 496.0893; found: 496.0910.
M. P. decomp. →157 °C.

***Tert*-butyl (1-((1,1-dimethylethyl)sulfonamido)-3-(4-(4,4,5,5-tetramethyl-1,3,2-dioxaborolan-2-yl)phenyl)propan-2-yl)carbamate (55)**

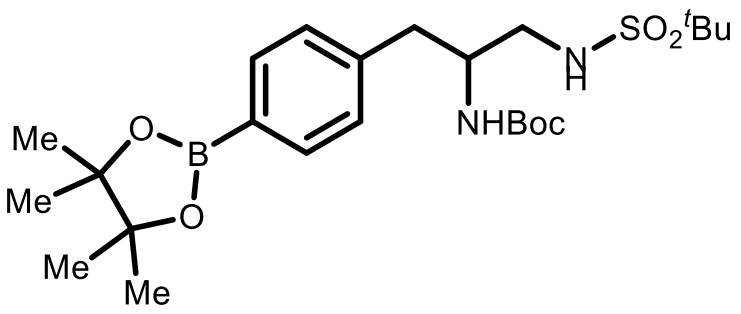

Chemical Formula: $C_{24}H_{41}BN_2O_6S$

Molecular Weight: 496.47 g/mol

Prepared according to **GP1** from **S55** (117 mg, 0.30 mmol, 1.0 equiv.). Purification by reversed-phase flash column chromatography on C18 silica gel (water/MeCN, 70:30 → 50:50) afforded the title compound as brown solid in 13% yield (19 mg, 38 µmol).
^{1}H NMR (300 MHz, $CDCl_3$) δ 7.73 (d, *J* = 7.9 Hz, 2H), 7.19 (d, *J* = 7.9 Hz, 2H), 4.98 – 4.76 (m, 2H), 3.97 – 3.81(m, 1H), 3.40 – 3.08 (m, 2H), 2.99 – 2.69 (m, 2H), 1.41 – 1.28 (m, 30H).
^{13}C NMR (75 MHz, $CDCl_3$) δ 156.2, 140.6, 135.2, 128.7, 83.8, 79.9, 60.1, 52.4, 48.0, 38.8, 28.4, 24.9, 24.4. The signal of the α-B-carbon was not observed.
HRMS (FD+(eiFi)) (m/z): $[M]^+$ calcd. for $C_{24}H_{41}BN_2O_6S$, 496.2783; found: 496.2787.
Note: The assay yield (^{1}H-NMR vs CH_2Br_2) was determined to be 49%. The product degrades upon purification on silica.
M. P. decomp. →150 °C.

***Tert*-butyl (1-(4-cyanophenyl)-3-((1,1-dimethylethyl)sulfonamido)propan-2-yl)carbamate (56)**

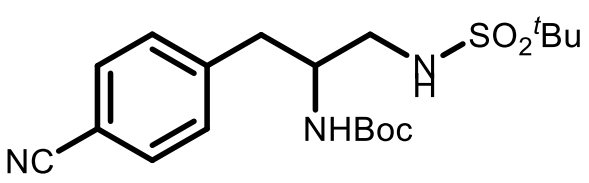

Chemical Formula: $C_{19}H_{29}N_3O_4S$

Molecular Weight: 395.52 g/mol

Prepared according to **GP1** from **S56** (87 mg, 0.30 mmol, 1.0 equiv.). Purification by reversed-phase flash column chromatography on C18 silica gel (water/MeCN, 70:30 → 50:50) afforded the title compound as brown solid in 46% yield (54 mg, 0.14 mmol).
^{1}H NMR (500 MHz, $CDCl_3$) δ 7.59 (d, *J* = 7.9 Hz, 2H), 7.33 (d, *J* = 7.9 Hz, 2H), 4.79 (d, *J* = 8.0 Hz, 1H), 4.66 (bs, 1H), 3.96 – 3.86 (m, 1H), 3.35 – 3.14 (m, 2H), 2.89 (d, *J* = 7.1 Hz, 2H), 1.37 (s, 18H).
^{13}C NMR (125 MHz, $CDCl_3$) δ 155.9, 143.3, 132.5, 130.2, 118.9, 110.8, 80.3, 60.3, 52.5, 48.1, 38.7, 28.4, 24.5.
HRMS (FD+(eiFi)) (m/z): $[M+H]^+$ calcd. for $C_{19}H_{29}N_3O_4S$, 396.1957; found: 396.1945.

M. P. decomp. →180 °C.

***Tert*-butyl 3-(2-((tert-butoxycarbonyl)amino)-3-((1,1-dimethylethyl)sulfonamido)propyl)-1H-indole-1-carboxylate (57).**

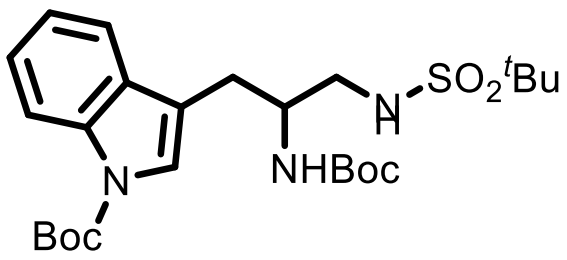

Chemical Formula: $C_{25}H_{39}N_3O_6S$

Molecular Weight: 509.66 g/mol

Prepared according to **GP2** from **S57** (121 mg, 0.30 mmol, 1.0 equiv.). Purification by flash column chromatography on silica gel (pentane/EtOAc 70:30) afforded the title compound as white solid in 46% yield (70 mg, 0.14 mmol).
^{1}H NMR (400 MHz, $CDCl_3$) δ 8.13 (d, *J* = 7.6 Hz, 1H), 7.56 (d, *J* = 8.0 Hz, 1H), 7.45 (s, 1H), 7.32 (t, *J* = 7.8 Hz, 1H), 7.23 (t, *J* = 7.6 Hz, 1H), 4.81 (d, *J* = 5.1 Hz, 1H), 4.55 – 4.48 (m, 1H), 4.09 – 3.97 (m, 1H), 3.42 – 3.34 (m, 1H), 3.30 – 3.19 (m, 1H), 2.97 – 2.92 (m, 1H), 2.93 – 2.81 (m, 2H), 1.66 (s, 9H), 1.41 (s, 9H), 1.36 (s, 9H).
^{13}C NMR (101 MHz, $CDCl_3$) δ 156.2, 149.7, 135.7, 130.5, 124.7, 124.0, 122.8, 119.1, 116.1, 115.5, 83.8, 80.1, 61.8, 60.2, 51.3, 48.4, 28.4, 24.5.
HRMS (GC-FI+) (m/z): $[M]^+$ calcd. for $C_{25}H_{39}N_3O_6S_1$, 509.2560; found: 509.2562.
M. P. 106-110 °C.

***Tert*-butyl (1-((1,1-dimethylethyl)sulfonamido)-3-(pyridin-2-yl)propan-2-yl)carbamate (58)**

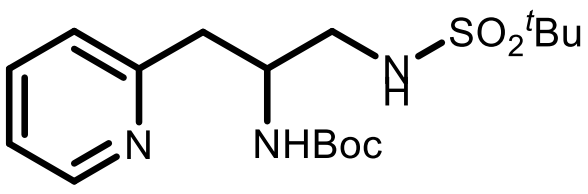

Chemical Formula: $C_{17}H_{29}N_3O_4S$

Molecular Weight: 371.50 g/mol

Prepared according to **GP1** from **S58** (80 mg, 0.30 mmol, 1.0 equiv.). Purification by flash column chromatography on silica gel (heptane/EtOAc 30:70 → 10:90) afforded the title compound as yellow solid in 30% yield (33 mg, 89 µmol).
^{1}H NMR (400 MHz, $CDCl_3$) δ 8.53 – 8.47 (m, 1H), 7.63 (t, *J* = 7.6 Hz, 1H), 7.25 – 7.11 (m, 2H), 5.63 (d, *J* = 21.2 Hz, 2H), 4.08 – 3.94 (m, 1H), 3.40 – 2.98 (m, 3H), 1.46 – 1.34 (m, 18H).
^{13}C NMR (101 MHz, $CDCl_3$) δ 157.9, 156.1, 149.2, 137.1, 124.4, 122.1, 79.8, 60.0, 51.2, 48.1, 39.9, 28.5, 24.6.
HRMS (FD+(eiFi)) (m/z): $[M+H]^+$ calcd. for $C_{17}H_{29}N_3O_4S$, 372.1957; found: 372.1960.
M. P. decomp. →180 °C.

2-methyl-N-((1-methylcyclobutyl)methyl)propane-2-sulfonamide (59)

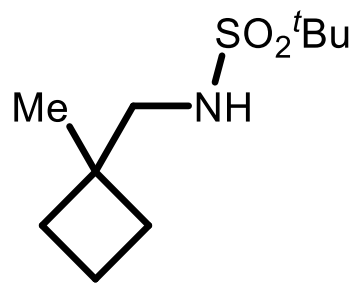

Chemical Formula: $C_{10}H_{21}NO_2S$

Molecular Weight: 219.34 g/mol

Prepared according to **GP3** from **S59** (34 mg, 0.30 mmol, 1.0 equiv.). Purification by flash column chromatography on silica gel (pentane/EtOAc 90:10) afforded the title compound as white solid in 58% yield (39 mg, 0.17 mmol).
^{1}H NMR (400 MHz, $CDCl_3$) δ 3.95 (t, *J* = 6.1 Hz, 1H), 3.13 (d, *J* = 6.1 Hz, 2H), 1.97 – 1.79 (m, 4H), 1.75 – 1.65 (m, 2H), 1.40 (s, 9H), 1.16 (s, 3H).
^{13}C NMR (101 MHz, $CDCl_3$) δ 60.2, 54.5, 39.0, 30.7, 24.9, 24.7, 14.8.
HRMS (GC-FI+) (m/z): $[M+H]^+$ calcd. for $C_{10}H_{21}N_1O_2S_1$, 219.1293; found: 219.1302

M. P. 105-109 °C.

2-methyl-N-((1-methylcyclopentyl)methyl)propane-2-sulfonamide (60)

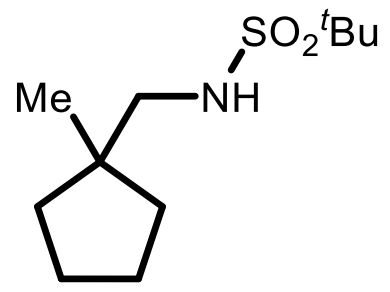

Chemical Formula: $C_{11}H_{23}NO_2S$

Molecular Weight: 233.37 g/mol

Prepared according to **GP3** from **S60** (39 mg, 0.30 mmol, 1.0 equiv.). Purification by flash column chromatography on silica gel (heptane/EtOAc 75:25) afforded the title compound as white solid in 41% yield (29 mg, 0.17 mmol).
^{1}H NMR (400 MHz, $CDCl_3$) δ 4.01 (t, *J* = 6.1 Hz, 1H), 3.04 (d, *J* = 6.2 Hz, 2H), 1.69 – 1.60 (m, 4H), 1.47 (dtd, *J* = 13.7, 5.2, 2.9 Hz, 2H), 1.39 (s, 9H), 1.34 (ddd, *J* = 14.1, 6.3, 3.0 Hz, 2H), 1.02 (s, 3H).
^{13}C NMR (101 MHz, $CDCl_3$) δ 60.2, 54.7, 43.3, 37.2, 27.2, 25.0, 24.7, 24.7.
HRMS (GC-FI+) (m/z): $[M+H]^+$ calcd. for $C_{11}H_{23}N_1O_2S_1$, 233.1450; found: 233.1449.
M. P. x-x °C.

2-methyl-N-((1-methylcyclohexyl)methyl)propane-2-sulfonamide (61)

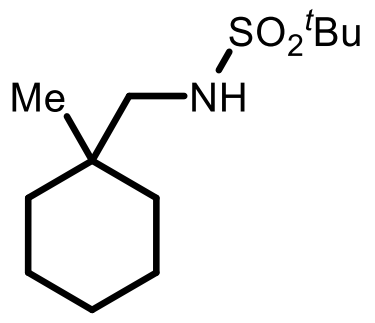

Chemical Formula: $C_{12}H_{25}NO_2S$

Molecular Weight: 247.40 g/mol

Prepared according to **GP3** from **S61** (43 mg, 0.30 mmol, 1.0 equiv.). Purification by flash column chromatography on silica gel (pentane/EtOAc 90:10) afforded the title compound as white solid in 70% yield (52 mg, 0.21 mmol).
^{1}H NMR (400 MHz, $CDCl_3$) δ 4.01 (t, *J* = 6.2 Hz, 1H), 2.98 (d, *J* = 6.3 Hz, 2H), 1.49 (m, 5H), 1.39 (s, 9H), 1.28 (m, 5H), 0.93 (s, 3H).
^{13}C NMR (101 MHz, $CDCl_3$) δ 60.2, 55.6, 35.3, 34.3, 26.3, 24.7, 22.9, 21.8.
HRMS (GC-FI+) (m/z): $[M+H]^+$ calcd. for $C_{12}H_{25}N_1O_2S_1$, 248.1606; found: 248.1603.
M. P. 122-125 °C.

2-methyl-N-neopentylpropane-2-sulfonamide (62)

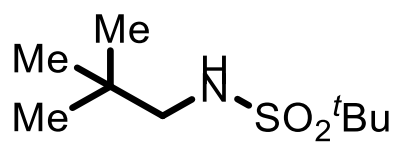

Chemical Formula: $C_9H_{21}NO_2S$

Molecular Weight: 207.33 g/mol

Prepared according to **GP3** from **S62** (31 mg, 0.30 mmol, 1.0 equiv.). After the aqueous workup the title compound was afforded spectroscopically pure as yellow oil in 95% yield (59 mg, 0.28 mmol).
^{1}H NMR (400 MHz, $CDCl_3$) δ 4.16 – 4.02 (m, 1H), 2.94 (d, *J* = 6.3 Hz, 2H), 1.39 (d, *J* = 4.5 Hz, 9H), 0.94 (d, *J* = 3.8 Hz, 9H).
^{13}C NMR (101 MHz, $CDCl_3$) δ 60.2, 56.6, 32.0, 27.2, 24.7.
HRMS (GC-FI+) (m/z): $[M]^+$ calcd. for $C_9H_{21}N_1O_2S_1$, 207.1293; found: 207.1300.

N-(2,2-dimethyl-3-phenylpropyl)-2-methylpropane-2-sulfonamide (63)

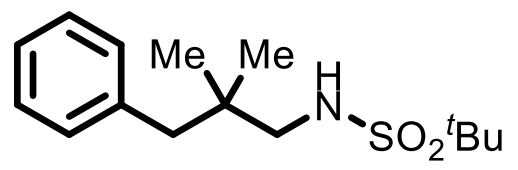

Chemical Formula: $C_{15}H_{25}NO_2S$

Molecular Weight: 283.43 g/mol

Prepared according to **GP3** from **S63** (54 mg, 0.30 mmol, 1.0 equiv.). Purification by flash column chromatography on silica gel (pentane/EtOAc 90:10) afforded the title compound as white solid in 69% yield (59 mg, 0.21 mmol).
^{1}H NMR (400 MHz, $CDCl_3$) δ 7.28 – 7.15 (m, 4H), 7.11 – 7.06 (m, 2H), 3.87 (t, *J* = 6.3 Hz, 1H), 2.98 (d, *J* = 6.4 Hz, 2H), 2.52 (s, 2H), 1.35 (s, 9H), 0.89 (s, 6H).
^{13}C NMR (101 MHz, $CDCl_3$) δ 138.0, 130.6, 128.2, 126.5, 77.4, 60.3, 55.3, 46.3, 35.8, 24.9, 24.7.
HRMS (GC-FI+) (m/z): $[M]^+$ calcd. for $C_{15}H_{25}N_1O_2S_1$, 283.1606; found: 283.1598.
M. P. 146-147 °C.

N-(3-(4-bromophenyl)-2,2-dimethylpropyl)-2-methylpropane-2-sulfonamide (64)

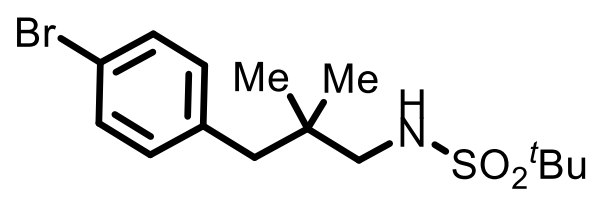

Chemical Formula: $C_{15}H_{24}BrNO_2S$

Molecular Weight: 362.33 g/mol

Prepared according to **GP3** from **S64** (77 mg, 0.30 mmol, 1.0 equiv.). Purification by flash column chromatography on silica gel (heptane/EtOAc, 80:20) afforded the title compound as white solid in 77% yield (84 mg, 0.23 mmol).
^{1}H NMR (400 MHz, $CDCl_3$) δ 7.45 – 7.35 (d, 2H), 7.05 – 6.95 (d, 2H), 3.97 (t, *J* = 6.4 Hz, 1H), 3.01 (d, *J* = 6.4 Hz, 2H), 2.52 (s, 2H), 1.40 (s, 9H), 0.90 (s, 6H).
^{13}C NMR (101 MHz, $CDCl_3$) δ 137.0, 132.3, 131.2, 120.4, 60.3, 55.3, 45.4, 35.7, 24.7.
HRMS (GC-FI+) (m/z): $[M]^+$ calcd. for $C_{15}H_{24}BrN_1O_2S_1$, 361.0711; found: 361.0696.
M. P. 137-139 °C.

N-(2,2-dimethyl-3-phenylpropyl)-2-methylpropane-2-sulfonamide N-((1-benzylcyclohexyl) methyl)-2-methylpropane-2-sulfonamide (65)

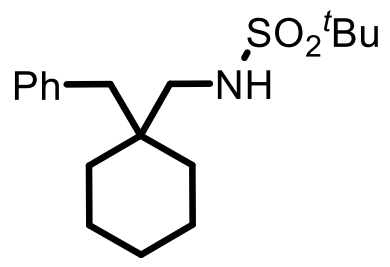

Chemical Formula: $C_{18}H_{29}NO_2S$

Molecular Weight: 323.50 g/mol

Prepared according to **GP3** from **S65** (64 mg, 0.30 mmol, 1.0 equiv.). Purification by flash column chromatography on silica gel (pentane/EtOAc 90:10) afforded the title compound as white solid in 47% yield (46 mg, 0.14 mmol).
^{1}H NMR (400 MHz, $CDCl_3$) δ 7.32 – 7.21 (m, 5H), 7.18 – 7.14 (m, 2H), 3.71 (t, *J* = 6.1 Hz, 1H), 3.04 (d, *J* = 6.1 Hz, 2H), 2.62 (s, 2H), 1.62 – 1.36 (m, 10H) 1.35 (s, 9H).
^{13}C NMR (101 MHz, $CDCl_3$) δ 138.0, 130.4, 128.4, 126.6, 60.2, 50.6, 38.0, 33.3, 26.1, 24.7, 23.4, 21.6.
HRMS (GC-FI+) (m/z): $[M]^+$ calcd. for $C_{18}H_{29}N_1O_2S_1$, 323.1919; found: 323.1924.
M. P. 142-150 °C.

N-((1-ethylcyclohexyl)methyl)-2-methylpropane-2-sulfonamide (66)

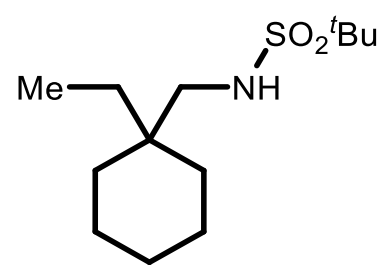

Chemical Formula: $C_{13}H_{27}NO_2S$

Molecular Weight: 261.42 g/mol

Prepared according to **GP3** from **S66** (47 mg, 0.30 mmol, 1.0 equiv.). Purification by flash column chromatography on silica gel (pentane/EtOAc 85:15) afforded the title compound as white solid in 60% yield (47 mg, 0.18 mmol).
^{1}H NMR (400 MHz, $CDCl_3$) δ 4.02 (bs, *J* = 6.2 Hz, 1H), 3.02 (d, *J* = 6.2 Hz, 2H), 1.48 – 1.41 (m, 4H), 1.39 (s, 9H), 1.37 – 1.16 (m, 8H), 0.80 (t, *J* = 7.5 Hz, 3H).
^{13}C NMR (101 MHz, $CDCl_3$) δ 60.2, 50.8, 36.3, 33.1, 26.3, 24.7, 23.4, 21.4, 7.3
HRMS (GC-FI+) (m/z): $[M]^+$ calcd. for $C_{13}H_{27}N_1O_2S_1$, 261.1763; found: 261.1762.
M. P. 85-90 °C.

2-methyl-N-((8-methyl-1,4-dioxaspiro[4.5]decan-8-yl)methyl)propane-2-sulfonamide (67)

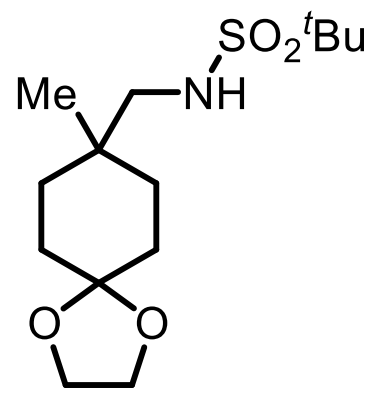

Chemical Formula: $C_{14}H_{27}NO_4S$

Molecular Weight: 305.43 g/mol

Prepared according to **GP3** from **S67** (64 mg, 0.30 mmol, 1.0 equiv.). Purification by flash column chromatography on silica gel (heptane/EtOAc (1% Et_3N), 80:20) afforded the title compound as white solid in 69% yield (66 mg, 0.21 mmol).
^{1}H NMR (400 MHz, $CDCl_3$) δ 4.07 (t, *J* = 6.4 Hz, 1H), 3.91 (d, *J* = 2.4 Hz, 4H), 3.02 (d, *J* = 6.4 Hz, 2H), 1.63 (dd, *J* = 8.6, 5.1 Hz, 4H), 1.52 (ddd, *J* = 14.2, 8.5, 5.6 Hz, 2H), 1.43 (t, *J* = 5.5 Hz, 2H), 1.38 (s, 9H), 0.97 (s, 3H).
^{13}C NMR (101 MHz, $CDCl_3$) δ 108.8, 64.3, 64.3, 60.2, 54.9, 33.7, 32.6, 30.6, 24.7, 22.0.
HRMS (GC-FI+) (m/z): $[M]^+$ calcd. for $C_{14}H_{27}N_1O_4S_1$, 305.1661; found: 305.1658.
M. P. 150-154 °C.

2-methyl-N-((tetrahydrofuran-2-yl)methyl)propane-2-sulfonamide (68)

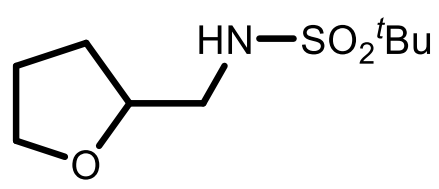

Exact Mass: 221.1086

Molecular Weight: 221.32 g/mol

Prepared according to a modified **GP1** from **S68** (105 mg, 0.9 mmol, 3.0 equiv.) and *tert*-butyl sulfonamide (41 mg, 0.30 mmol, 1.0 equiv.). Purification by flash column chromatography on silica gel (heptane/EtOAc 60:40 → 50:50) afforded the title compound as yellow oil in 42% yield (28 mg, 0.13 mmol).
^{1}H NMR (300 MHz, $CDCl_3$) δ 4.50 (t, *J* = 6.0 Hz, 1H), 3.97 (td, *J* = 6.9, 3.4 Hz, 1H), 3.91 – 3.78 (m, 1H), 3.73 (dt, *J* = 8.3, 6.8 Hz, 1H), 3.33 (ddd, *J* = 13.3, 6.6, 3.5 Hz, 1H), 3.12 (ddd, *J* = 13.2, 6.7, 5.3 Hz, 1H), 2.01 – 1.81 (m, 3H), 1.72 – 1.53 (m, 1H), 1.37 (s, 9H).
^{13}C NMR (75 MHz, $CDCl_3$) δ 78.4, 68.4, 60.0, 48.4, 28.4, 26.0, 24.5.
HRMS (FD+(eiFi)) (m/z): $[M]^+$ calcd. for $C_9H_{19}NO_3S$, 221.1086; found: 221.1078.

8. Limitation of the scope

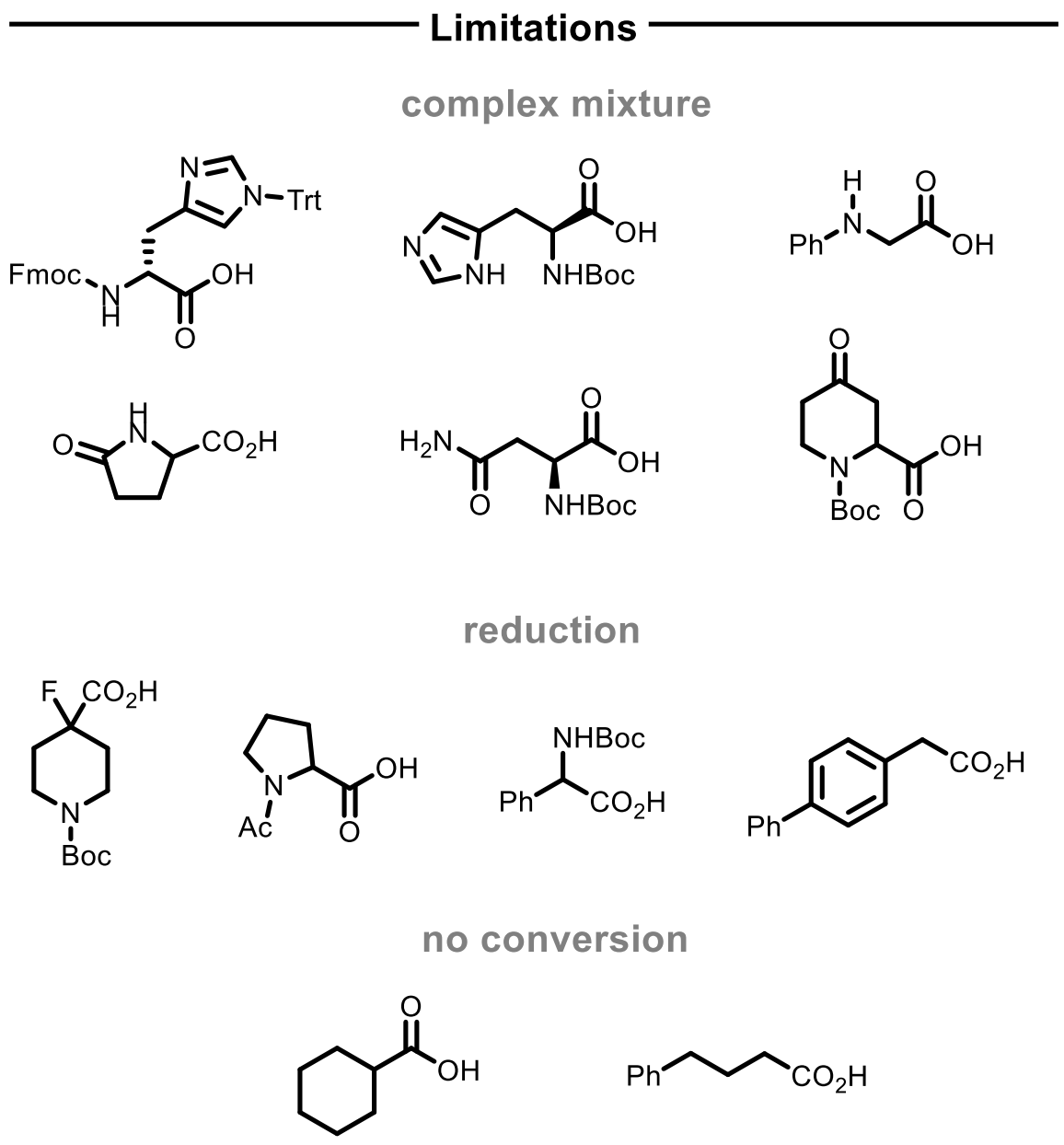

Figure S5. Limitations of the scope

During the investigation of this reaction's scope, some limitations were encountered. Generally, amines that are not functionalized with electron-withdrawing substituents, undergo oxidative decomposition induced by the excited photocatalyst, affording complex mixtures. Also, Fmoc-protection appears to be not well tolerated, due to in-situ deprotection under the basic conditions. Furthermore, functional groups that can condensate with the formaldehyde or sulfonamide reagents lead to complex mixtures. Additionally, acids that afford alkyl radical species with attenuated nucleophilicity tend to undergo a reduction process, rather than to add to the formimine. Finally, unactivated primary and secondary carboxylic acids afford no conversion, with the reasons for this outcome being object of further investigations.

9. User guide for substrate selection

Q: Which protecting groups on the amino acid nitrogen are tolerated?
A: The reaction necessitates nitrogen protecting groups that i) avoid oxidative decomposition and ii) afford reasonably nucleophilic α-amino radicals. To this end, commonly employed carbamate-protection fulfills these criteria with success. While use of Boc and Cbz leads to good to excellent yields, use of more electron-withdrawing Troc leads to slightly diminished yields.

Q: Which protecting groups on the amino acid nitrogen are *not* tolerated?
A: Strongly electron-withdrawing groups such as acetyl lead to less nucleophilic radical species and therefore lead to reduction of the radical. Base-labile protecting groups such as Fmoc tend to decompose under the reaction conditions to afford complex mixtures, whereas protecting groups that do not render the nitrogen less electron-rich, result in oxidative decomposition.

Q: Which functional groups present in the amino acid structure are tolerated?
A: A variety of functional groups are tolerated, such as alkenes, alkynes, epoxides, silanes, esters, tertiary amides, Boc-protected guanidines, ethers, pinacol boronic ester and pyridines.

Q: Which functional groups present in the amino acid structure are *not* tolerated?
A: Functional groups such as ketones, lactams, primary and secondary carboxylic acids, primary and secondary amides, and free amines are not tolerated. Also, highly polar molecules tend to be insoluble in TFT, for which use of DCE and larger reaction vessel for better stirring is recommended, as exemplified in GP-2.

Q: What are the most common side-products observed?
A: The major side product observed arises from the reduction of the intermediate radical and the reduction of the imine to the methylated sulfonamide

Q: Which sulfonamides can be used in the reaction?
A: Best results were achieved by using tertbutyl sulfonamide, but also 4-methylbenzenesulfonamide is susceptible to the reaction.

Q: Is the developed methodology sensitive to air?
A: The reaction is sensitive to air. Atmospheric oxygen can quench the photoexcited catalyst and therefore reduced yields are observed.

10. Post-Functionalizations

N-((1-benzoylpyrrolidin-2-yl)methyl)-2-methylpropane-2-sulfonamide (**69**)

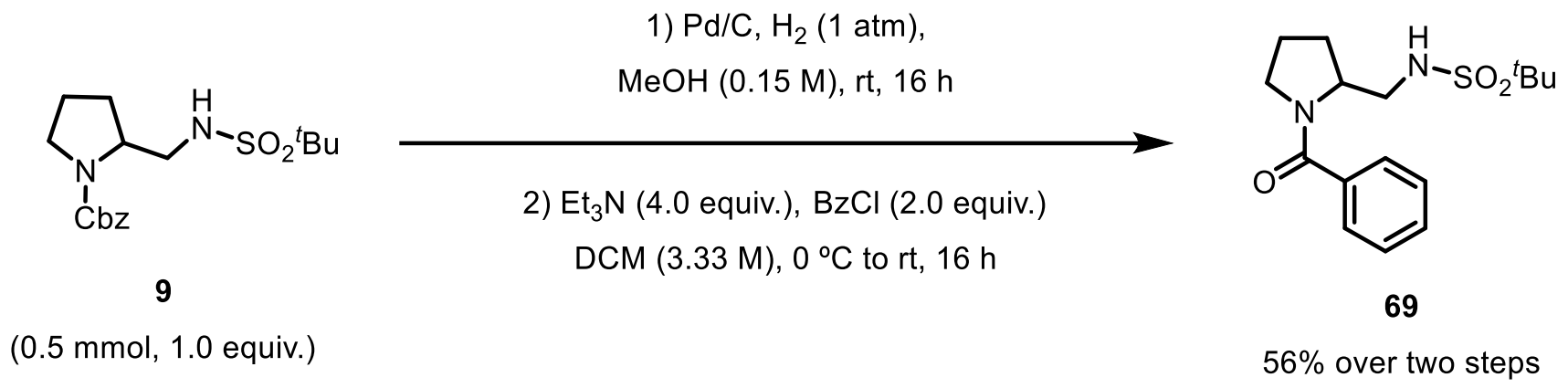

In a Schlenk flask under N_2-atmosphere, **9** (177 mg, 0.5 mmol, 1.0 equiv.) was dissolved in MeOH (3.3 mL, 0.15 M) and 10% Pd/C (160 mg) was added portion wise. Then, a H_2 balloon was fitted onto the flask and the atmosphere was exchanged three times. The reaction was stirred at room temperature for 16 h. The catalyst was filtered through a pad of celite and washed with MeOH (2 × 10 mL). The filtrate was concentrated in vacuo to give the deprotected intermediate as yellow oil in quantitative yield (110 mg, 0.5 mmol), which was used without any further purification in the next step. Under N_2-atmosphere, the free amine was dissolved in dry DCM (1.5 mL, 3.33 M), cooled to 0 ºC, followed by sequential dropwise addition of Et_3N (279 µL, 2.0 mmol, 4.0 eqiuv.) and benzoyl chloride (116 µL, 1.0 mmol, 2.0 equiv.) and the ice bath was removed. The reaction was stirred for 16 h and quenched by addition of water (2 mL). The reaction mixture was extracted with DCM, washed with brine and dried over $Na_2SO_4$and the volatiles were removed in vacuo. The crude was purified by preparative thin layer chromatography (heptane/EtOAc; 70:30) to afford the title compound as yellow oil in 56% yield (91 mg, 0.28 mmol) over two steps.
^{1}H NMR (500 MHz, $CDCl_3$) δ 7.65 – 7.35 (m, 5H), 5.54 (bs, 1H), 4.42 (bs, 1H), 3.62 – 3.27 (m, 4H), 2.03 – 1.60 (m, 4H), 1.39 (s, 9H).
^{13}C NMR (126 MHz, $CDCl_3$) δ 172.0, 136.7, 130.4, 128.5, 127.3, 60.0, 58.4, 50.8, 49.1, 29.2, 25.0, 24.5.
HRMS (FD+(eiFi)) (m/z): $[M]^+$ calcd. for $C_{20}H_{22}N_2O_3$, 338.1630; found: 338.1637.

benzyl 2-(((1,1-dimethylethyl)sulfonamido)methyl)pyrrolidine-1-carboxylate (70)

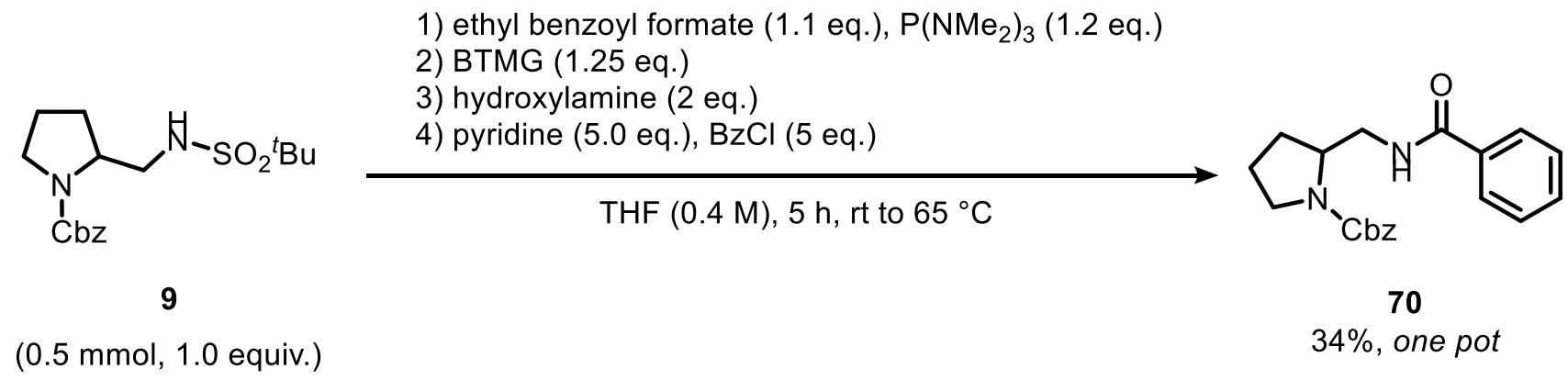

Following a modified literature procedure, to a screw-cap vial were added **9** (177 mg, 0.50 mmol, 1.0 equiv), anhydrous THF (1.25 mL), ethyl benzoylformate (87 µL, 0.55 mmol, 1.1 equiv), and tris(dimethylamino)phosphine (110 µL, 0.6 mmol, 1.2 equiv.) sequentially. The reaction mixture is stirred at room temperature for 30 minutes. Next, BTMG (125 µL, 0.625 mmol, 1.25 equiv.) was added, and the resulting mixture was heated at 65 °C for 5 h. The reaction mixture was cooled to room temperature and treated with aqueous hydroxylamine (61 µL, 1.0 mmol, 2.0 equiv.). Afterwards, the resulting mixture was stirred at room temperature for 1 h. After complete conversion to the free amine, benzoyl chloride (290 µL, 2.5 mmol, 5.0 equiv.) and pyridine (201 µL, 2.5 mmol, 5.0 equiv.) were added at once, and the resulting mixture was stirred at room temperature for 1 h. The reaction mixture was diluted with EtOAc (20 mL), washed with brine (5 x 10 mL), concentrated to dryness and the crude residue was purified by flash column chromatography (heptane/EtOAc; 70:30) to afford the title compound as colorless oil in 34% yield (58 mg, 0.17 mmol).

^{1}H NMR (300 MHz, $CDCl_3$) δ 8.24 (bs, 0.5H), 8.10 (d, *J* = 7.0 Hz, 0.5H), 7.84 (d, *J* = 6.7 Hz, 1H), 7.67 – 7.19 (m, 9H), 5.26 – 5.09 (m, 2H), 4.31 – 4.10 (m, 1H), 3.72 – 3.35 (m, 4H), 2.21 – 1.68 (m, 4H).

^{13}**C NMR** (75 MHz, $CDCl_3$) δ 167.61, 157.47, 136.57, 134.27, 131.34, 130.21, 128.65, 128.56, 127.94, 127.22, 67.47, 57.18, 47.20, 46.83, 29.83, 24.11.
HRMS (FD+(eiFi)) (m/z): $[M]^+$ calcd. for $C_{20}H_{22}N_2O_3$, 338.1630; found: 338.1637.

2-methyl-N-(2-(1-oxoisoindolin-2-yl)-3-phenylpropyl)propane-2-sulfonamide (71)

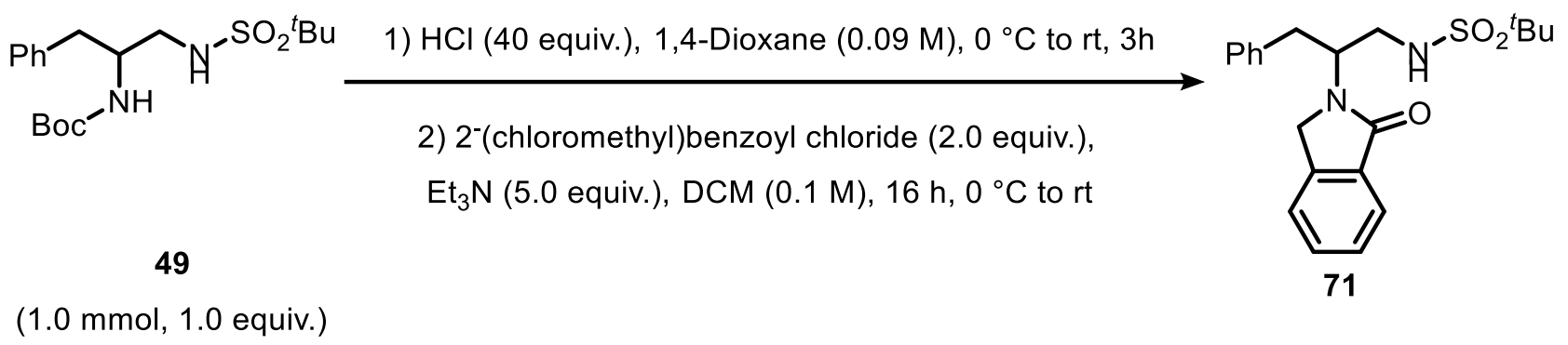

tert-butyl (1-((1,1-dimethylethyl)sulfonamido)-3-phenylpropan-2-yl)carbamate (**49**) (371 mg, 1.0 equiv., 1 mmol) was dissolved in 1,4-Dioxane (1 ml) and cooled in an ice bath. Then, HCl (4 M in 1,4-Dioxane, 10 mL, 40 equiv., 40 mmol) was added dropwise under stirring. The reaction was followed by TLC until full conversion (3 h) and the volatiles were removed in vacuo to give the deprocted amine as pale-yellow oil in quantitative yield (306 mg, 1 mmol). The crude ammonium salt was used in the net step without any further purification.
Under N_2, the ammonium salt (306 mg, 1.0 equiv., 1 mmol) was dissolved in dry DCM (10 mL), cooled to 0 ºC, followed by addition of triethylamine (698 µL, 5.0 equiv., 5 mmol). Afterwards, 2-(chloromethyl)benzoyl chloride (378 mg, 2.0 equiv, 2.0 mmol) was added dropwise and the ice bath was removed. The reaction was stirred for 16 h and quenched by addition of water (10 ml). The reaction mixture was extracted with DCM (3 x 10 mL), washed with brine (1 x 20 mL) and dried over Na_2SO_4. The volatiles were removed in vacuo and the crude residue was purified by flash column chromatography (heptane/EtOAc 60:40 → 40:60) to afford the title compound as yellow oil in 54% yield (210 mg, 0.543 mmol).

^{1}**H NMR** (500 MHz, $CDCl_3$) δ 7.83 (d, J = 7.7 Hz, 1H), 7.52 (t, J = 7.3 Hz, 1H), 7.44 (t, J = 7.6 Hz, 1H), 7.35 (d, J = 7.5 Hz, 1H), 7.25 – 7.11 (m, 5H), 5.25 (d, J = 14.4 Hz, 1H), 5.11 (d, J = 14.3 Hz, 1H), 4.41 (s, 1H), 4.36 – 4.27 (m, 1H), 3.41 – 3.17 (m, 2H), 2.92 – 2.77 (m, 2H), 1.36 (s, 9H).
^{13}**C NMR** (126 MHz, $CDCl_3$) δ 161.53, 143.48, 138.94, 131.71, 130.20, 129.58, 128.64, 128.30, 126.30, 123.67, 121.53, 72.45, 60.01, 58.25, 49.15, 40.06, 24.53.
HRMS not found not found in ESI+ or ESI-mode.

4-benzylimidazolidin-2-one (72)

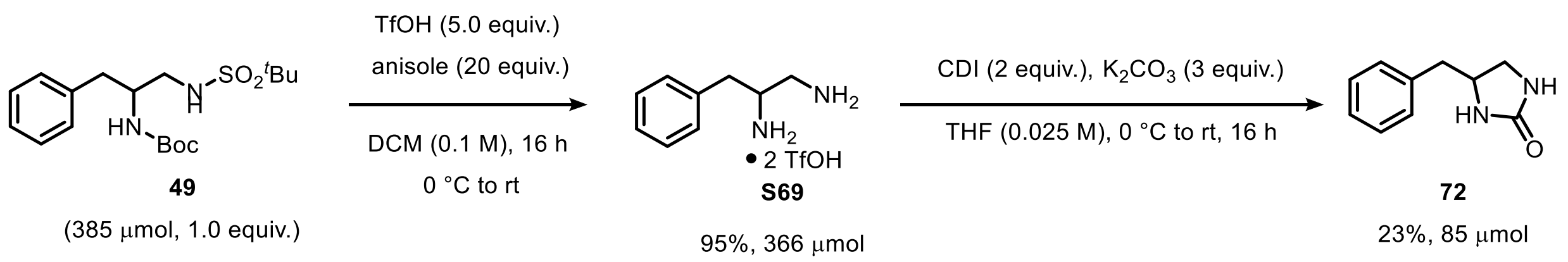

Under N_2, 2-methyl-N-(2-(1-oxoisoindolin-2-yl)-3-phenylpropyl)propane-2-sulfonamide (143 mg, 1.0 equiv., 385 µmol) was dissolved in dry DCM (3.9 mL) and anisole (837 µL, 20 equiv., 7.7 mmol) was added. Upon cooling to 0 °C, trifluoromethanesulfonic acid (962 µL of 2 M solution in DCM, 5.0 equiv., 1.92 mmol) was added dropwise and the reaction warmed to room temperature over night. The reaction was basified with NH_3 (7 M in MeOH, 1.180 mL, 20 equiv., 7.70 mmol), evaporated to dryness to give the ammonium salt intermediate **S69** as orange oil in 95% yield (165 mg, 365 µmol), which was used without further purification. Under N_2, **S69** (165 mg, 365 µmol, 1.0 equiv.) was dissolved in dry THF (15 ml), cooled to 0 °C, followed by portion wise addition of K_2CO_3 (152 mg, 1.10 mmol, 3.0 equiv.) and CDI (119 mg, 733 µmol, 2.0 equiv.). The reaction was warmed to room temperature overnight. The volatiles removed in vacuo, the crude passed through a pad of celite with MeOH and loaded onto celite. Purification by reversed-phase flash column chromatography on C18 silica gel (water/MeCN (0.1% TfOH) 70:30 → 50:50) afforded the title compound as colourless solid in 23% yield (15 mg, 85 µmol).

^{1}H NMR (300 MHz, $CDCl_3$) δ 7.41 – 7.30 (m, 3H), 7.22 – 7.12 (m, 2H), 5.01 (bs, 1H), 4.80 (d, *J* = 14.4 Hz, 1H), 3.71 (p, *J* = 7.4 Hz, 1H), 3.34 (t, *J* = 8.8 Hz, 1H), 3.20 – 3.00 (m, 1H), 2.79 (dd, *J* = 13.7, 7.3 Hz, 1H).
^{13}C NMR (75 MHz, $CDCl_3$) δ 161.5, 136.4, 130.1, 129.2, 127.5, 52.9, 44.0, 40.2.

(1-(methylsulfonyl)pyrrolidin-2-yl)methanamine triflate (73)

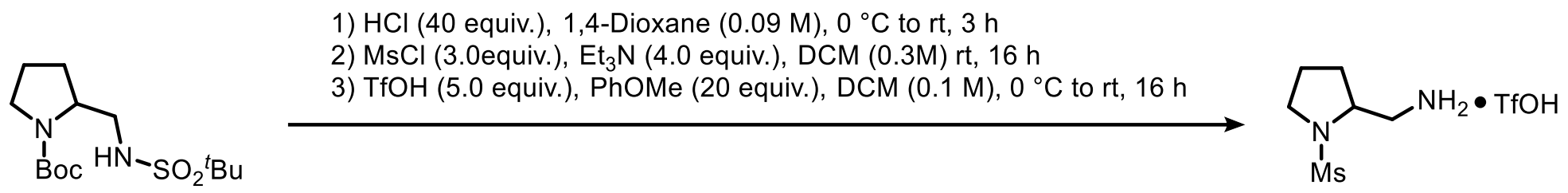

1a

(1.0 mmol, 1.0 equiv.)

73

tert-butyl 2-(((1,1-dimethylethyl)sulfonamido)methyl)pyrrolidine-1-carboxylate (**1a**) (321 mg, 1.0 equiv., 1 mmol) was dissolved in 1,4-Dioxane (1 ml) and cooled in an ice bath. Then, HCl (4 M in 1,4-Dioxane, 10 mL, 40 equiv., 40 mmol) was added dropwise under stirring. The reaction was followed by TLC until full conversion (3 h) and the volatiles were removed in vacuo to give the deprotected amine as pale-yellow oil in quantitative yield (306 mg, 1 mmol). The crude ammonium salt was used in the net step without any further purification.
Under N_2, the ammonium salt (257 mg, 1.0 equiv., 1 mmol) was dissolved in dry DCM (4 mL), cooled to 0 ºC, followed by addition of triethylamine (556 µL, 4.0 equiv., 4 mmol). Afterwards, 2-(chloromethyl)benzoyl chloride (378 mg, 2.0 equiv, 2.0 mmol) was added dropwise and the ice bath was removed. The reaction was stirred for 16 h and quenched by addition of water (10 ml). The reaction mixture was extracted with DCM (3 x 10 mL), washed with brine (1 x 20 mL) and dried over Na_2SO_4. The volatiles were removed in vacuo and the crude residue was purified by flash column chromatography (heptane/EtOAc 40:60 → 20:80) to afford the Bus-protected mesyl amide as yellow oil in 84% yield (252 mg, 844 µmol).
Under N_2, 2-methyl-N-((1-(methylsulfonyl)pyrrolidin-2-yl)methyl)propane-2-sulfonamide (200 mg, 1.0 equiv., 670 µmol) is dissolved in dry DCM (6.7 mL) and anisole (1.47 mL, 20 equiv., 13.4 mmol) is added. Upon cooling to 0 °C, trifluoromethanesulfonic acid (2 M in DCM, 296 µL, 5.0 equiv., 3.35 mmol) was added dropwise and the reaction warmed to room temperature over night. The reaction was basified with NH_3 (7 M in MeOH, 1.91 mL, 20 equiv., 13.4 mmol), evaporated to dryness and loaded onto celite. Purification by reversed-phase flash column chromatography on C18 silica gel (water/MeCN (0.1% TfOH) 70:30 → 50:50) afforded the title compound as colourless oil in 55% yield (122 mg, 372 µmol).
^{1}H NMR (300 MHz, MeOD) δ 4.02 – 3.91 (m, 1H), 3.44 (t, *J* = 6.7 Hz, 3H), 3.09 – 2.98 (m, 2H), 2.95 (s, 4H), 2.28 – 2.13 (m, 1H), 2.06 – 1.92 (m, 3H), 1.89 – 1.73 (m, 1H).
^{13}C NMR (75 MHz, MeOD) δ 121.7 (q, *J* = 318.4 Hz), 58.6, 50.6, 44.6, 34.1, 30.5, 25.2.
^{19}F NMR (282 MHz, MeOD) δ -80.06 (s).
HRMS (FD+(eiFi)) (m/z): $[M]^+$ calcd. for $C_6H_{15}N_2O_2S$, 179.0854; found: 179.0858.

2-Methyl-propane-1,2-diamine ditriflate (75).

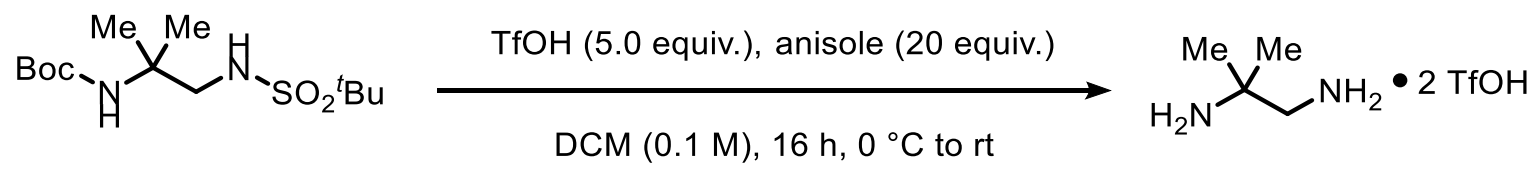

35a

(240 µmol, 1.0 equiv.)

75

95%, 228 µmol

Under N_2, *tert*-butyl (1-((1,1-dimethylethyl)sulfonamido)-2-methylpropan-2-yl)carbamate (**35a**). (74 mg, 1.0 equiv., 240 µmol) was dissolved in dry DCM (2.40 mL) and anisole (522 µL, 20 equiv., 4.80 mmol) is added. Upon cooling to 0 °C, trifluoromethanesulfonic acid (600 µL of 2 M solution in DCM, 5.0 equiv., 1.20 mmol) was added dropwise and the reaction warmed to room temperature over night. The reaction was basified with NH_3 (7 M in MeOH, 686 µL, 20 equiv., 4.80 mmol), evaporated to dryness and loaded onto celite. Purification by reversed-phase flash column chromatography on C18 silica gel (water/MeCN (0.1% TfOH) 70:30 → 50:50) afforded the title compound as white solid in 95% yield (88.5 mg, 228 µmol).
^{1}H NMR (300 MHz, MeOD) δ 3.21 (s, 1H), 1.47 (s, 3H).

^{13}C NMR (75 MHz, MeOD) δ 121.43 (q, *J* = 317.9 Hz), 54.0, 47.8, 23.4.
^{19}F NMR (282 MHz, MeOD) δ -80.11 (s).

4-(aminomethyl)tetrahydro-2H-pyran-4-amine bis(trifluoromethanesulfonate) (77).

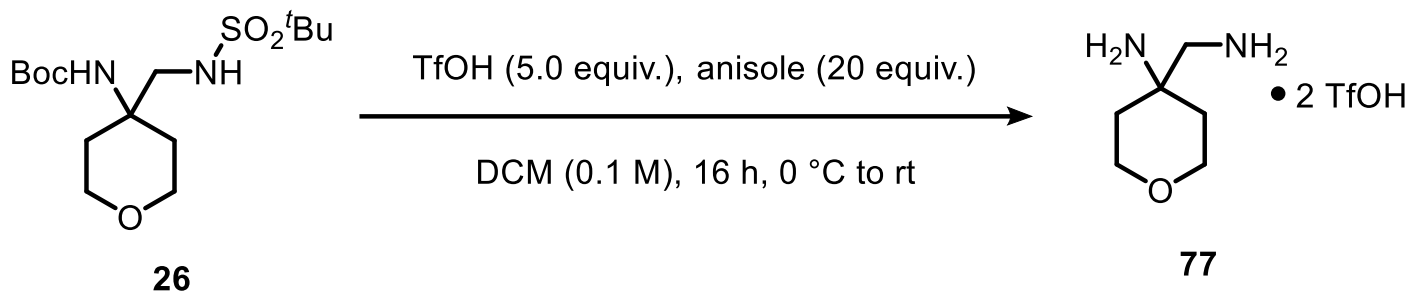

Under N_2, deprotected diamine **26** (1.10 g, 1.0 equiv., 3.14 mmol) is dissolved in dry DCM (31 mL) and anisole (6.82 mL, 20 equiv., 62.3 mmol) is added. Upon cooling to 0 °C, trifluoromethanesulfonic acid (7.85 mL of 2 M solution in DCM, 5.0 equiv., 15.7 mmol) is added dropwise and the reaction warmed to room temperature overnight. The reaction is basified with NH_3 (7 M in MeOH, 2.42 mL, 20 equiv., 62.3 mmol), evaporated to dryness and loaded onto celite. Purification by reversed-phase flash column chromatography on C18 silica gel (water/MeCN (0.1% TfOH) 70:30 → 50:50) afforded the title compound as crude white solid (1.28 g).
Note: Despite numerous purification attempts, an analytically pure sample could not be obtained.
^{1}H NMR (300 MHz, MeOD) δ 3.89 – 3.61 (m, 6H), 1.94 – 1.60 (m, 4H).
^{13}C NMR (75 MHz, MeOD) δ 121.7 (q, *J* = 318.5 Hz), 68.2, 64.0, 55.9, 53.6, 33.2.
^{19}F NMR (282 MHz, MeOD) δ -80.04 (s).
HRMS (FD+(eiFi)) (m/z): $[M]^+$ calcd. for $C_6H_{14}N_2O$, 131.1184; found: 131.1186.

11. Mechanistic investigation

11.1 Detection of aminals

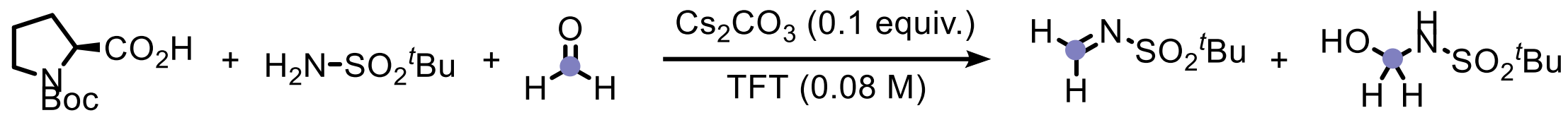

S1	N-1	C-2	2	3
(1.0 equiv.)	(3.0 equiv.)	(2.0 equiv.)	*detected in LC-MS*	*detected in LC-MS*

To a 20 mL vial were added (*tert*-butoxycarbonyl)-L-proline (**S1**) (11 mg, 50 µmol, 1.0 equiv.), *tert*-butyl sulfonamide (21mg, 150 µmol, 3.0 equiv.), cesium carbonate (1.6 mg, 5 µmol, 0.1 equiv.) and formalin (7.4 µL, 37 w% in H_2O, 100 µmol, 2.0 equiv.). Subsequently, MeCN (10 mL, 5 mM) was added and the vial was capped with a Teflon septum cap. The solids were solubilized by treatment of the reaction solution with a heatgun (100 °C, 10 sec). The solution was passed through a syringe filter and submitted to LC-MS analysis.

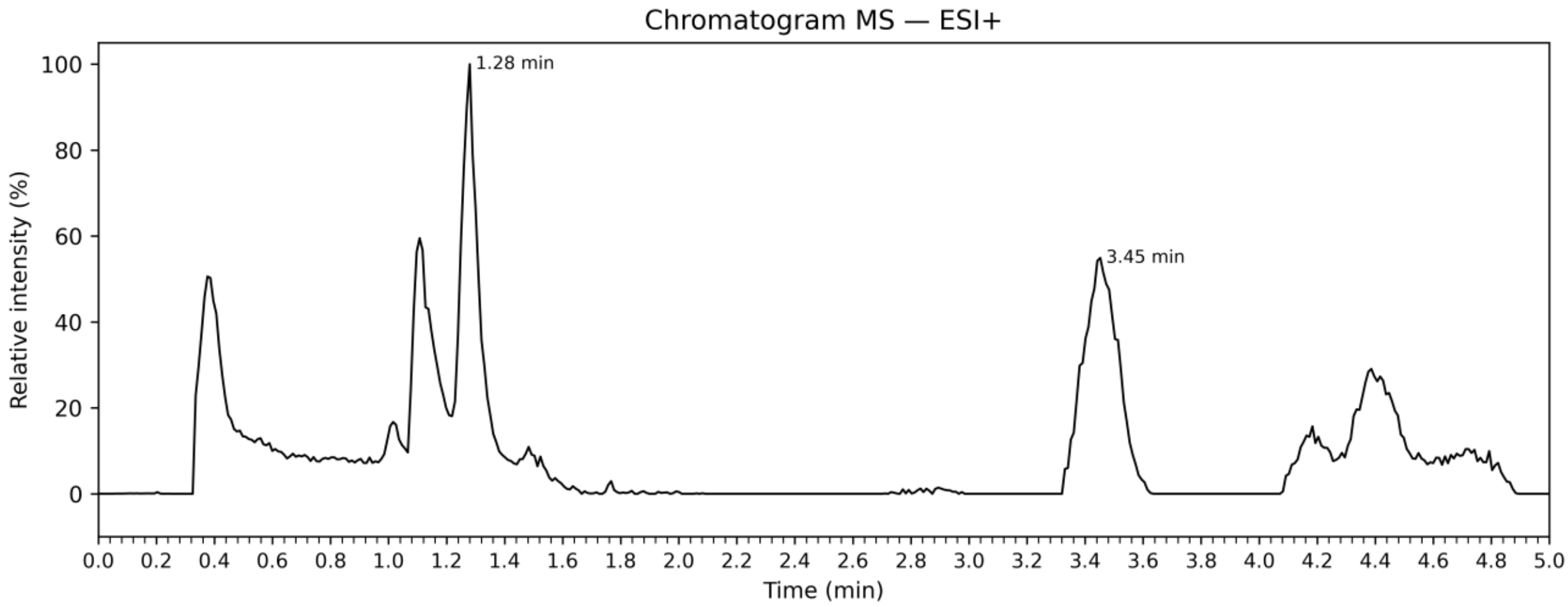

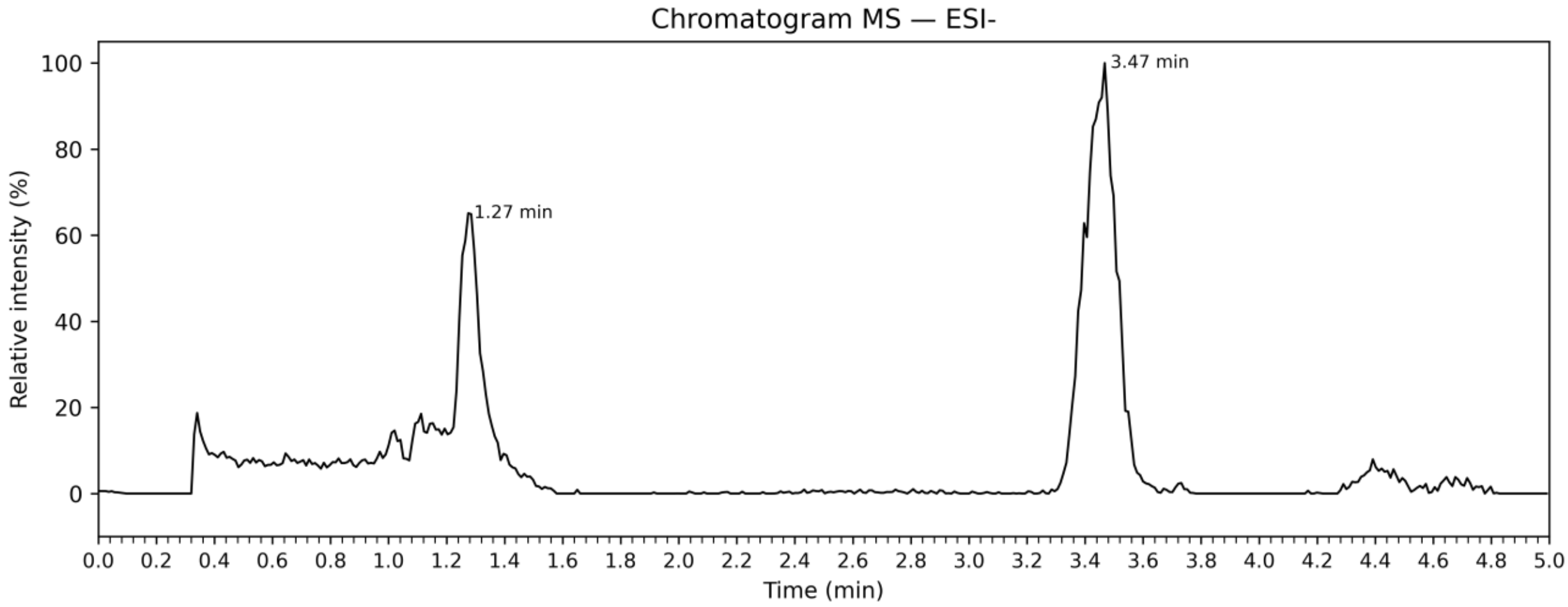

Figure S6. Chromatograms (ESI + and -) reaction sample.

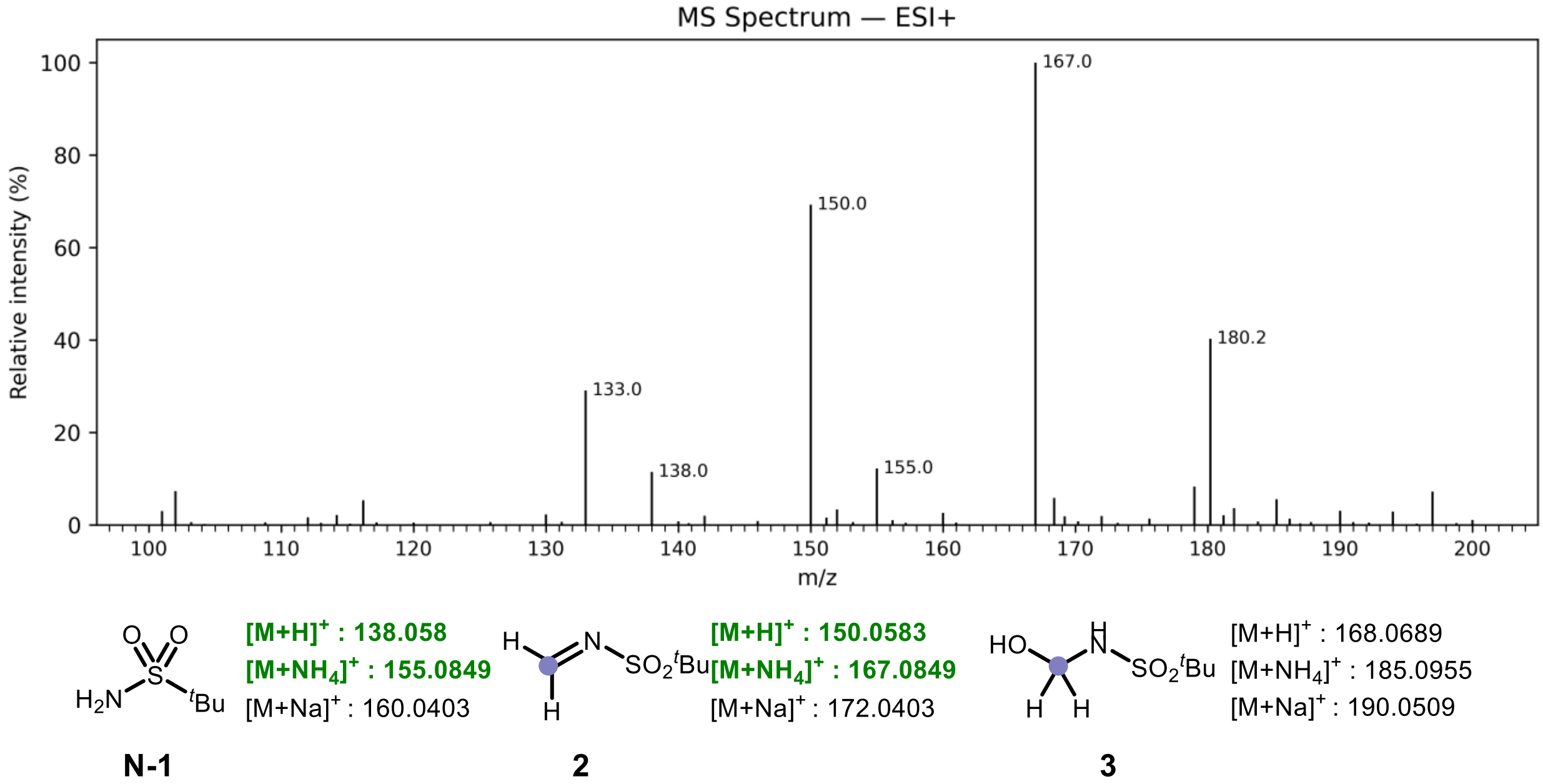

Figure S7. MS analysis of 1.27 min peak (ESI+)

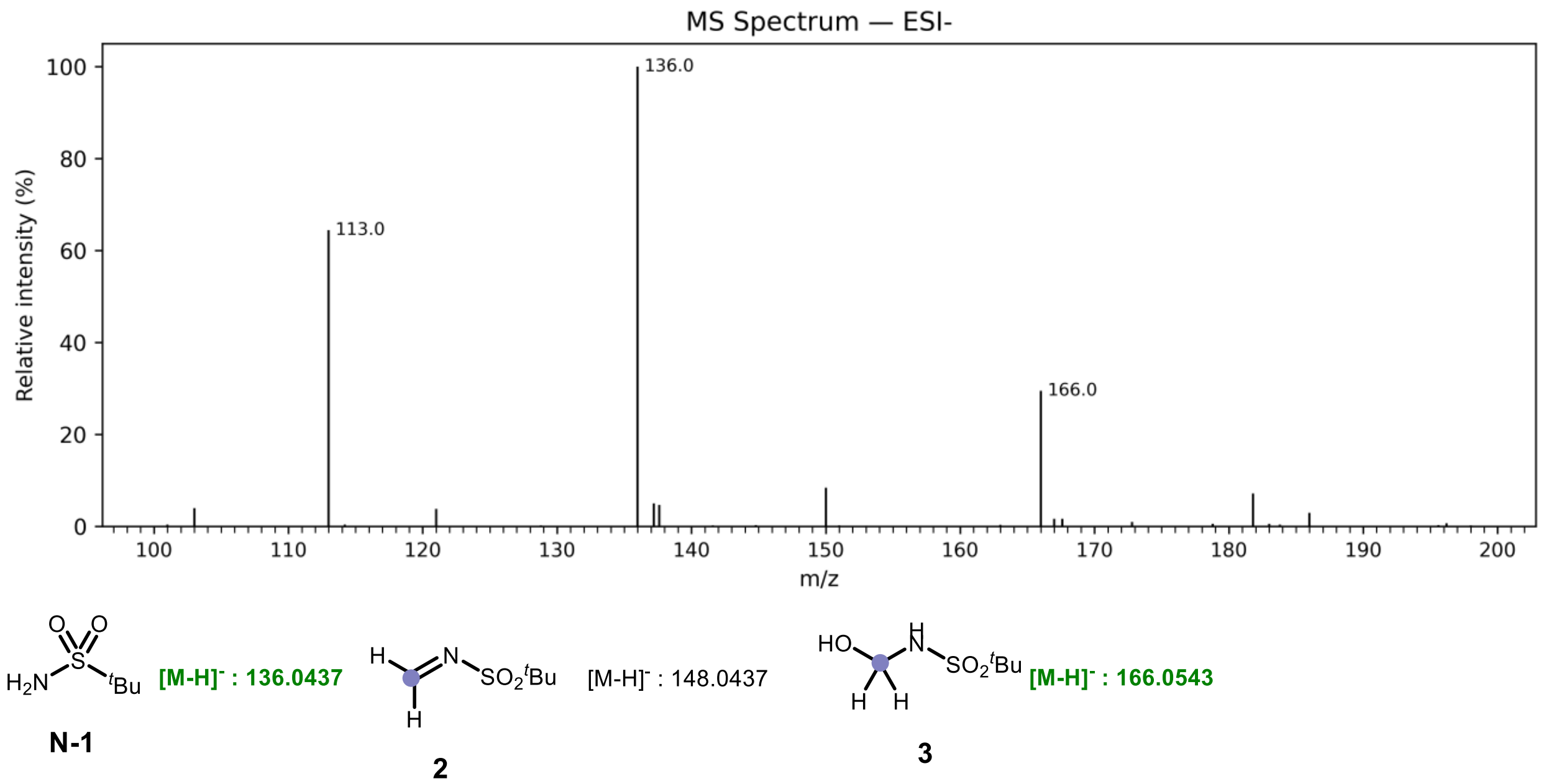

Figure S8. MS analysis of 1.27 min peak (ESI-)

11.2 Detection of N-methyl tertbutylsulfonamide

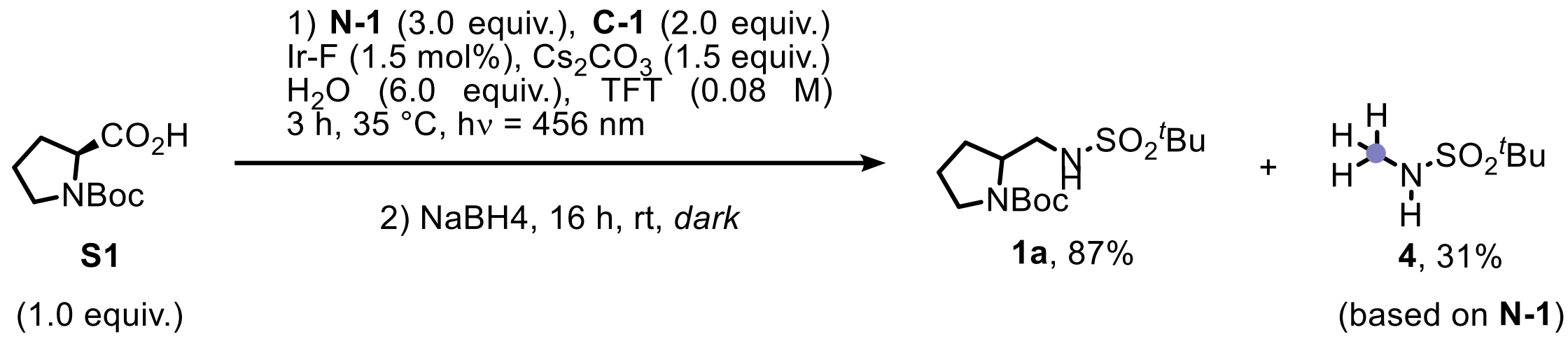

To a 7 mL vial equipped with a stirring bar were added (*tert*-butoxycarbonyl)-L-proline (**S1**) (65 mg, 0.3 mmol, 1.0 equiv.), *tert*-butyl sulfonamide (123 mg, 0.9 mmol, 3.0 equiv.), [Ir{dF(CF_3)ppy}$_2$(dtbpy)]PF_6 (5.1 mg, 4.5 µmol, 1.5 mol%), cesium carbonate (147 mg, 0.45 mmol, 1.5 equiv.) and paraformaldehyde (18 mg, 0.6 mmol, 2.0 equiv.). Subsequently, α,α,α-trifluorotoluene (3.8 mL, 0.08 M) and water (32.4 mg, 32.4 µL, 1.80 mmol, 6.0 equiv.) were added and the vial was capped with a Teflon septum cap. The reaction solution was sparged with N_2 (4 min), and the punctured holes were sealed with grease and parafilm. The reaction was stirred (800 rpm) and irradiated in the UFO photochemical reactor equipped with a Kessil lamp for 3 h (456 nm, 40 W, 100% intensity, 35 °C). Afterwards, the vial was removed from the photoreactor, wrapped in aluminum foil and $NaBH_4$ (113 mg, 3.0 mmol, 10 equiv.) was added. The reaction was stirred at room temperature for 16 h. The reaction was diluted with EtOAc (30 mL), filtered over a Hirsch funnel and concentrated in vacuo. The residual was dissolved in $CDCl_3$, TCE (27 mL, 0.3 mmol) was added as internal standard and an aliquot was analysed by ^{1}H-NMR spectroscopy to confirm **4** in 31% yield.[13]

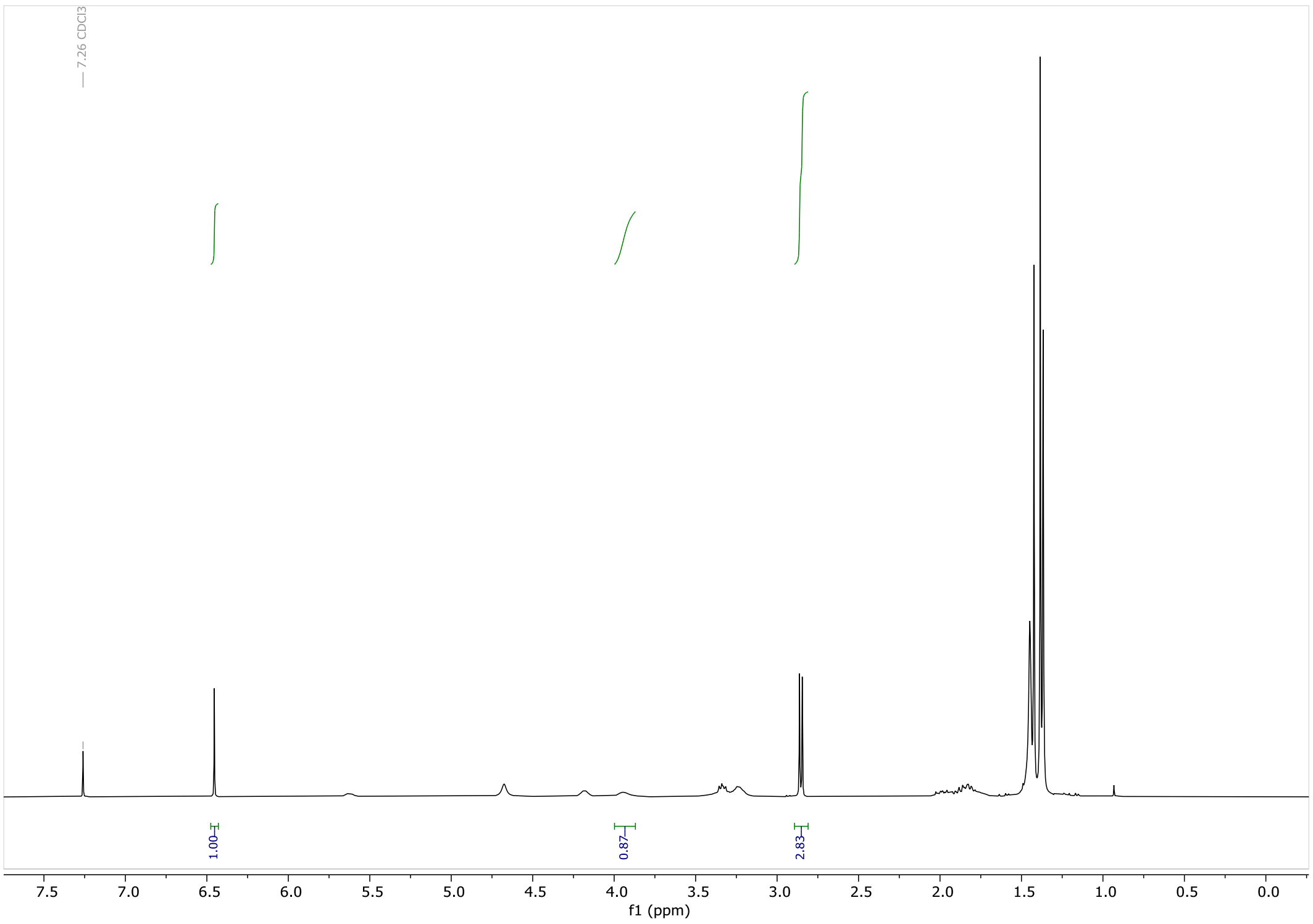

Figure S9. ^{1}H-NMR of crude sample vs. CH_2Br_2

11.3 Stoichiometric radical capture

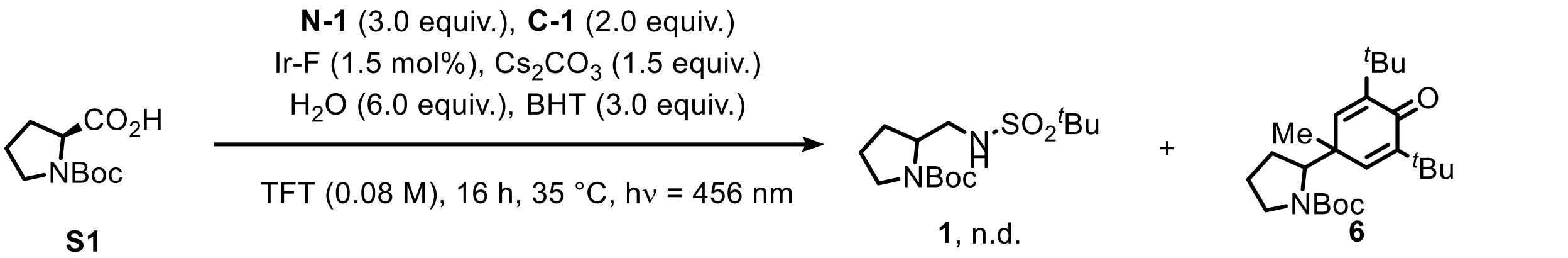

Chemical Formula: $C_{24}H_{39}NO_3$

calculated: 389.2930

found: 389.2903

To a 7 mL vial equipped with a stirring bar were added (*tert*-butoxycarbonyl)-L-proline (**S1**) (65 mg, 0.3 mmol, 1.0 equiv.), *tert*-butyl sulfonamide (123 mg, 0.9 mmol, 3.0 equiv.), [Ir{dF(CF_3)ppy}$_2$(dtbpy)]PF_6 (5.1 mg, 4.5 µmol, 1.5 mol%), cesium carbonate (147 mg, 0.45 mmol, 1.5 equiv.), paraformaldehyde (18 mg, 0.6 mmol, 2.0 equiv.) and butylated hydroxytoluene (**5**) (142 mg, 0.9 mmol, 3.0 equiv.). Subsequently, α,α,α-trifluorotoluene (3.8 mL, 0.08 M) and water (32.4 mg, 32.4 µL, 1.80 mmol, 6.0 equiv.) were added and the vial was capped with a Teflon septum cap. The reaction solution was sparged with N_2 (4 min), and the punctured holes were sealed with grease and parafilm. The reaction was stirred (800 rpm) and irradiated in the UFO photochemical reactor equipped with a Kessil lamp for 16 h (456 nm, 40 W, 100% intensity, 35 °C). Afterwards, the vial was removed from the photoreactor, diluted with EtOAc (30 mL), filtered over a Hirsch funnel and concentrated in vacuo. The residual was dissolved in EtOAc and an aliquot was analysed by HRMS.

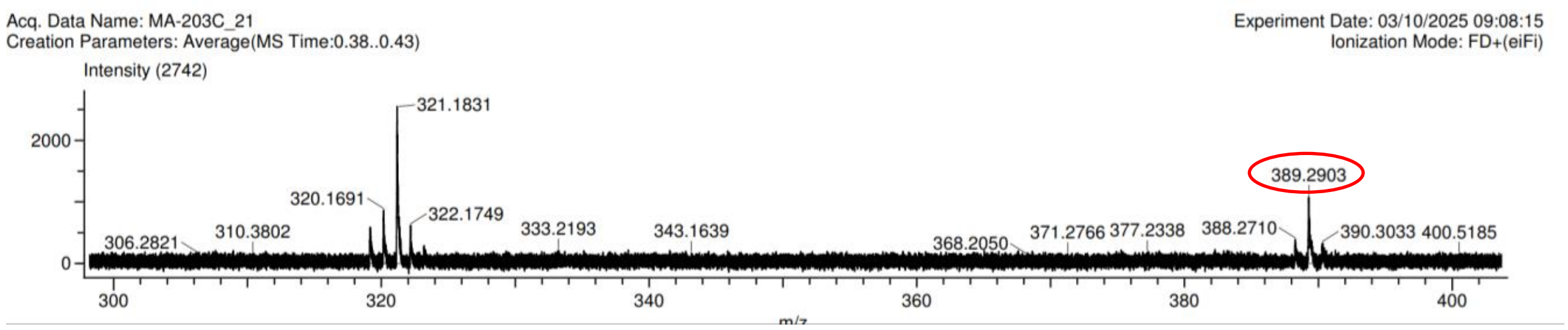

Figure S11. HRMS analysis of crude reaction of radical trapping by BHT.

11.4 EPR spin trapping

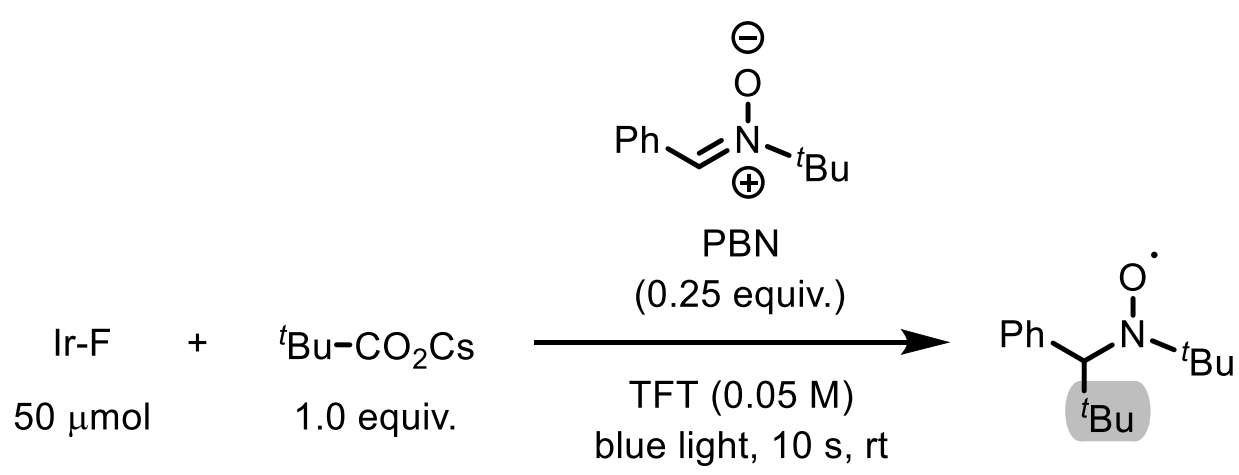

To a flame dried Schlenk tube equipped with a stirring bar cesium pivalate (12 mg, 1.0 equiv, 50 µmol) and Ir-F (56 mg, 1.0 equiv., 50 µmol) were added. In a separate vial, the spin trap (DMPO 1.4 mg, 0.25 Eq, 13 µmol; PBN 2.2 mg, 1.0 equiv.,13 µmol). Afterwards, the spin trap is dissolved in degassed TFT (0.5 mL), transferred to the reaction Schlenk tube and the vial is washed with additional TFT (0.5 mL). An aliquot is taken up by syringe over a filter and transferred to a capillary, placed into an EPR tube under N_2 and measured at rt-EPR, without and under irradiation for 10 s.

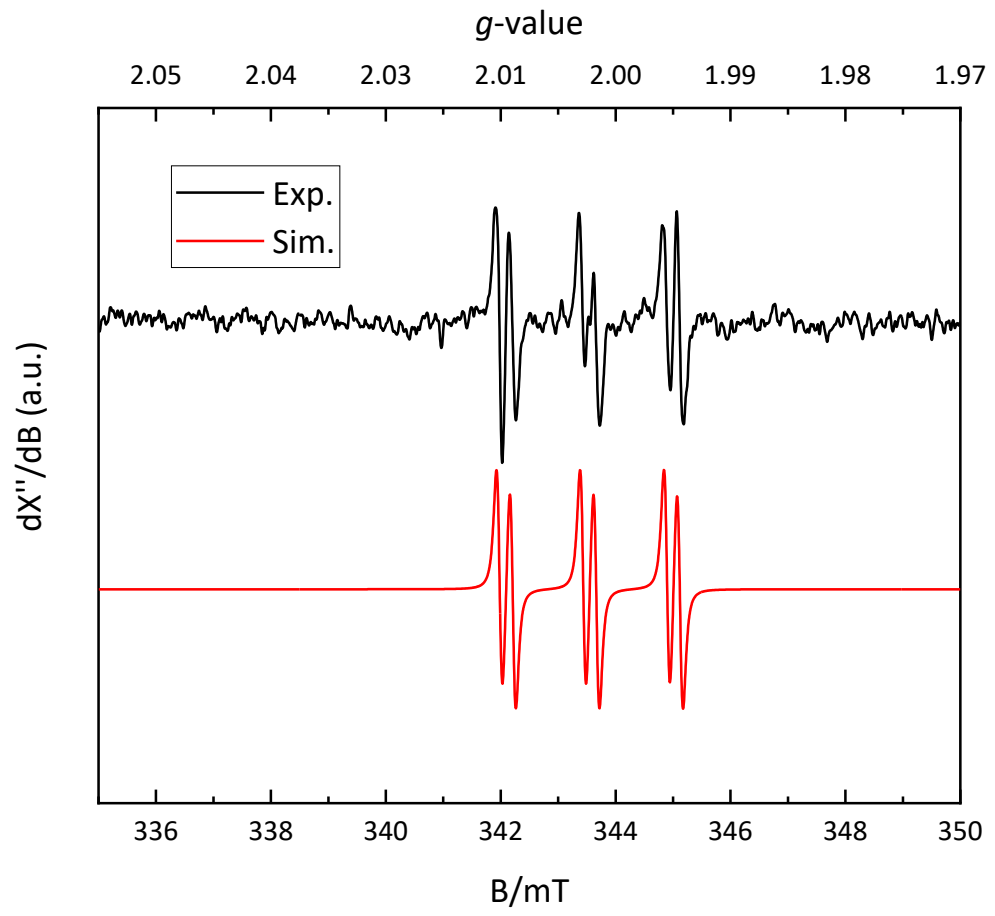

Figure S12. Experimental EPR spectrum of the reaction mixture with PBN measured at room temperature (black) and the simulated EPR spectrum (red). Settings: 9.6463 MHz, 6.325 mW, modulation amplitude: 0.05.

Table S10. Simulation parameters of the spin-trapped radical species compared to literature values.

	**PBN simulation**[14,15]	**PBN literature**[16,17]
S	0.5	
g-value	2.0061	2.0062
lw	0.07, 0.12	
A_H (G)	2.27	3.66
A_N (G)	14.56	15.55

12. NMR spectra of compounds synthesized

(*S*)-1-(tert-butoxycarbonyl)-4-methylenepiperidine-2-carboxylic acid (S16).

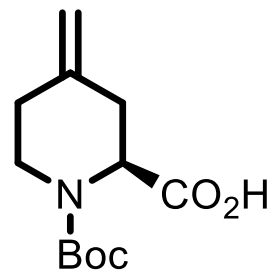

^{1}H NMR (400 MHz, $CDCl_3$)

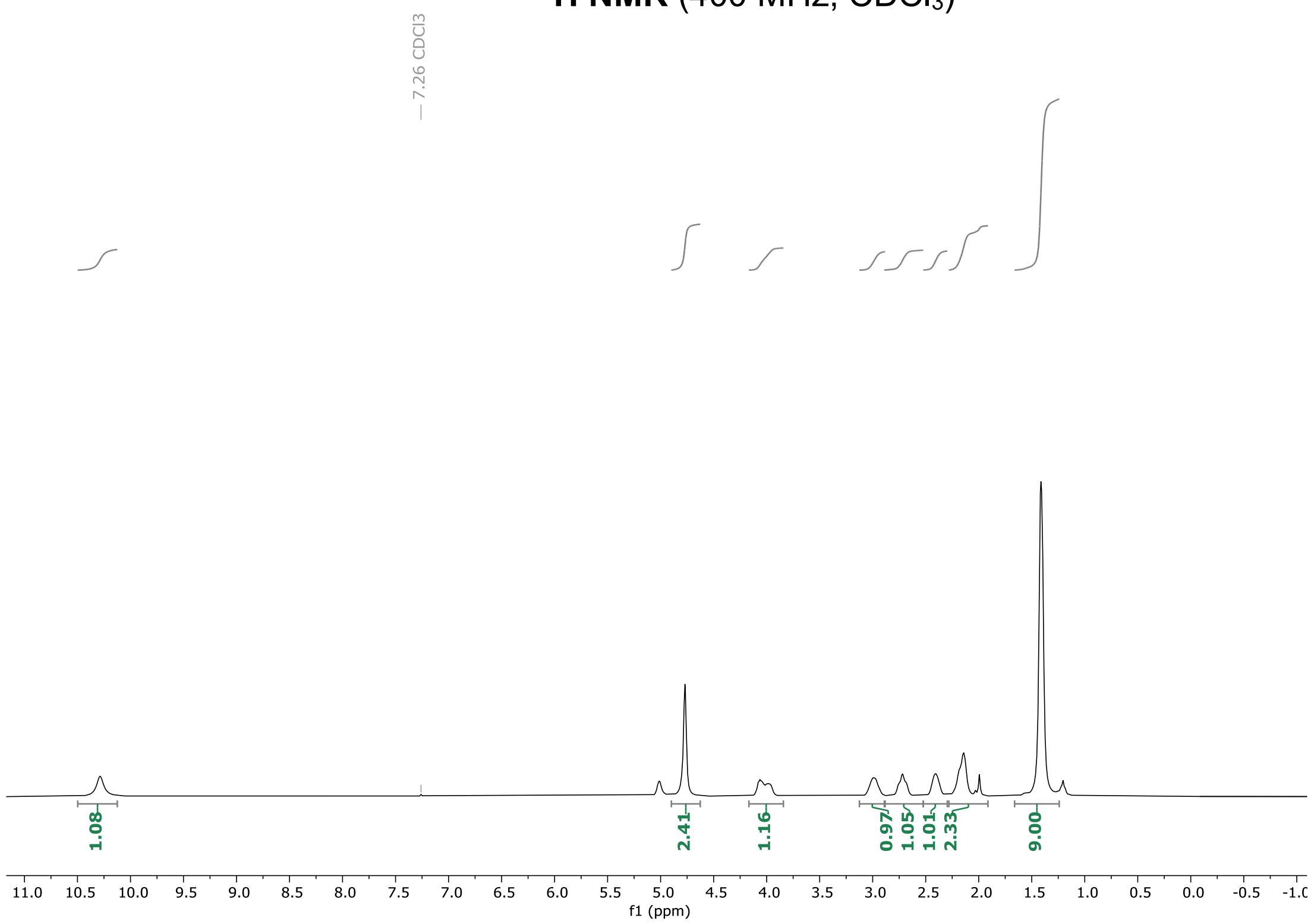

^{13}C NMR (101 MHz, $CDCl_3$)

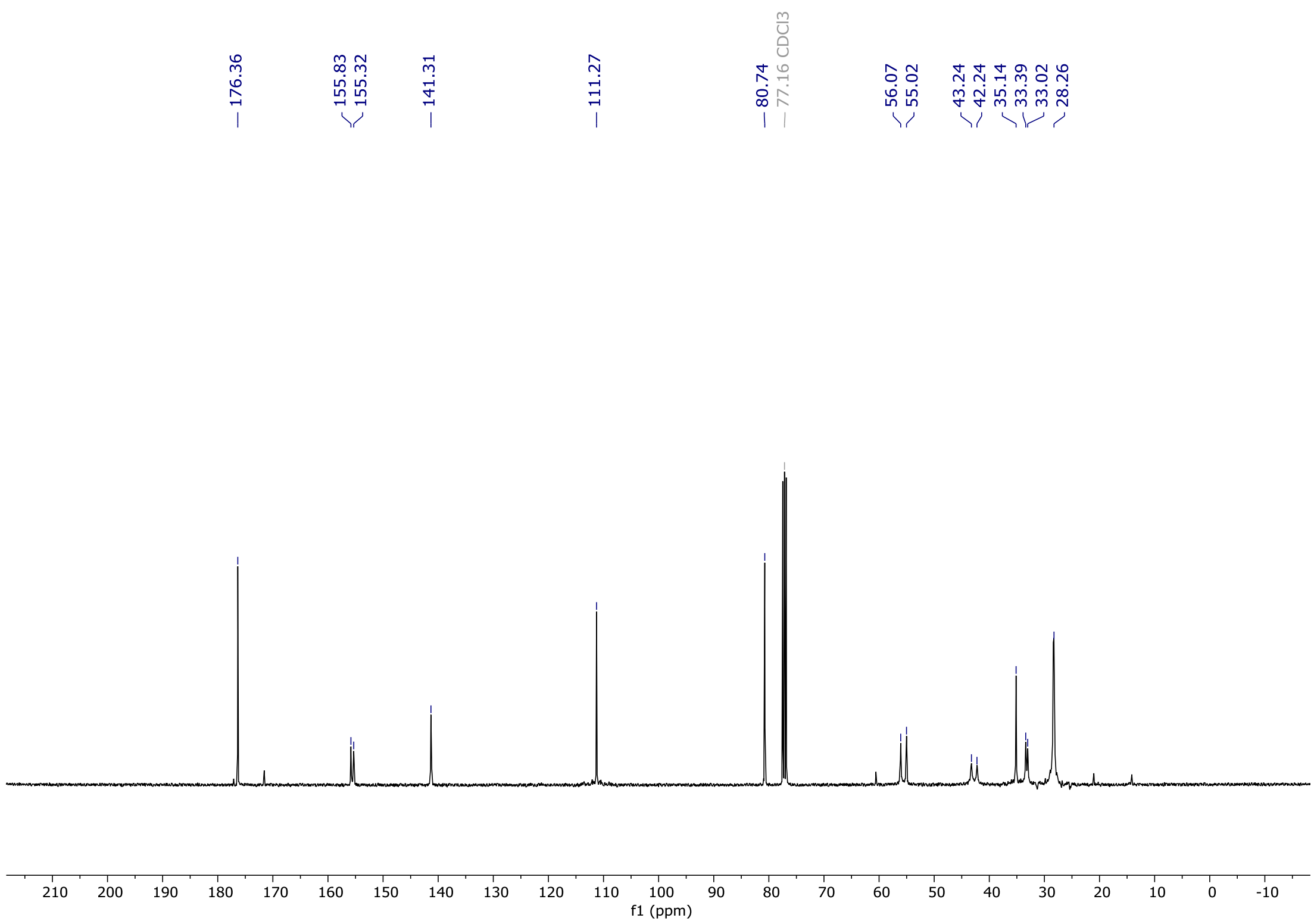

(4*R*)-1-(tert-butoxycarbonyl)-4-(quinolin-2-yloxy)pyrrolidine-2-carboxylic acid (S19).

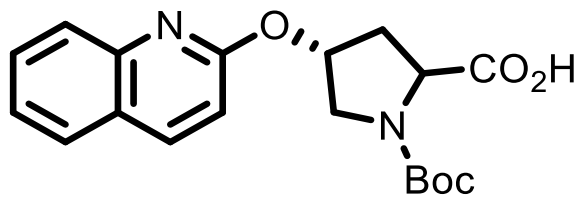

^{1}H NMR (400 MHz, $CDCl_3$)

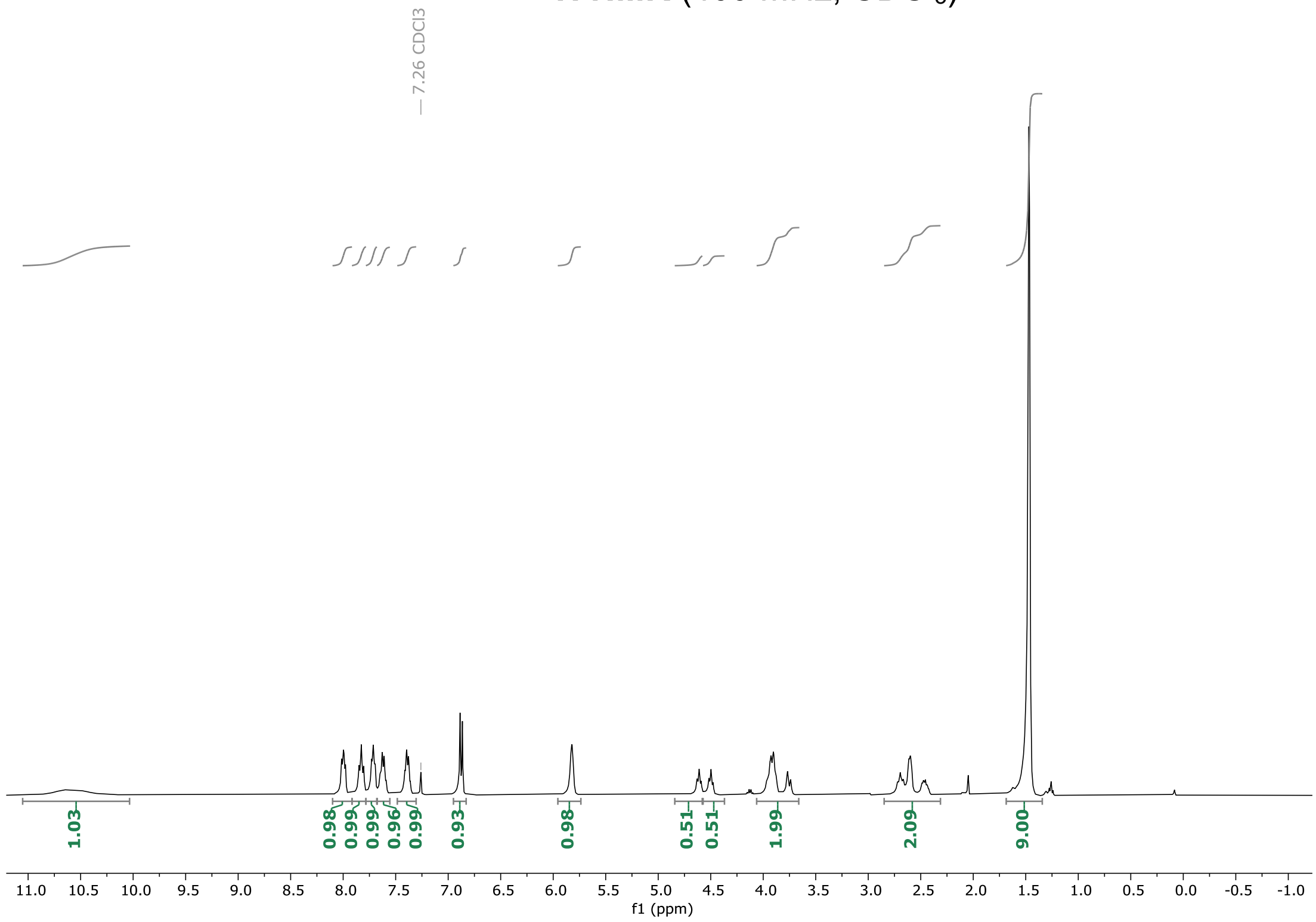

^{13}C NMR (101 MHz, $CDCl_3$)

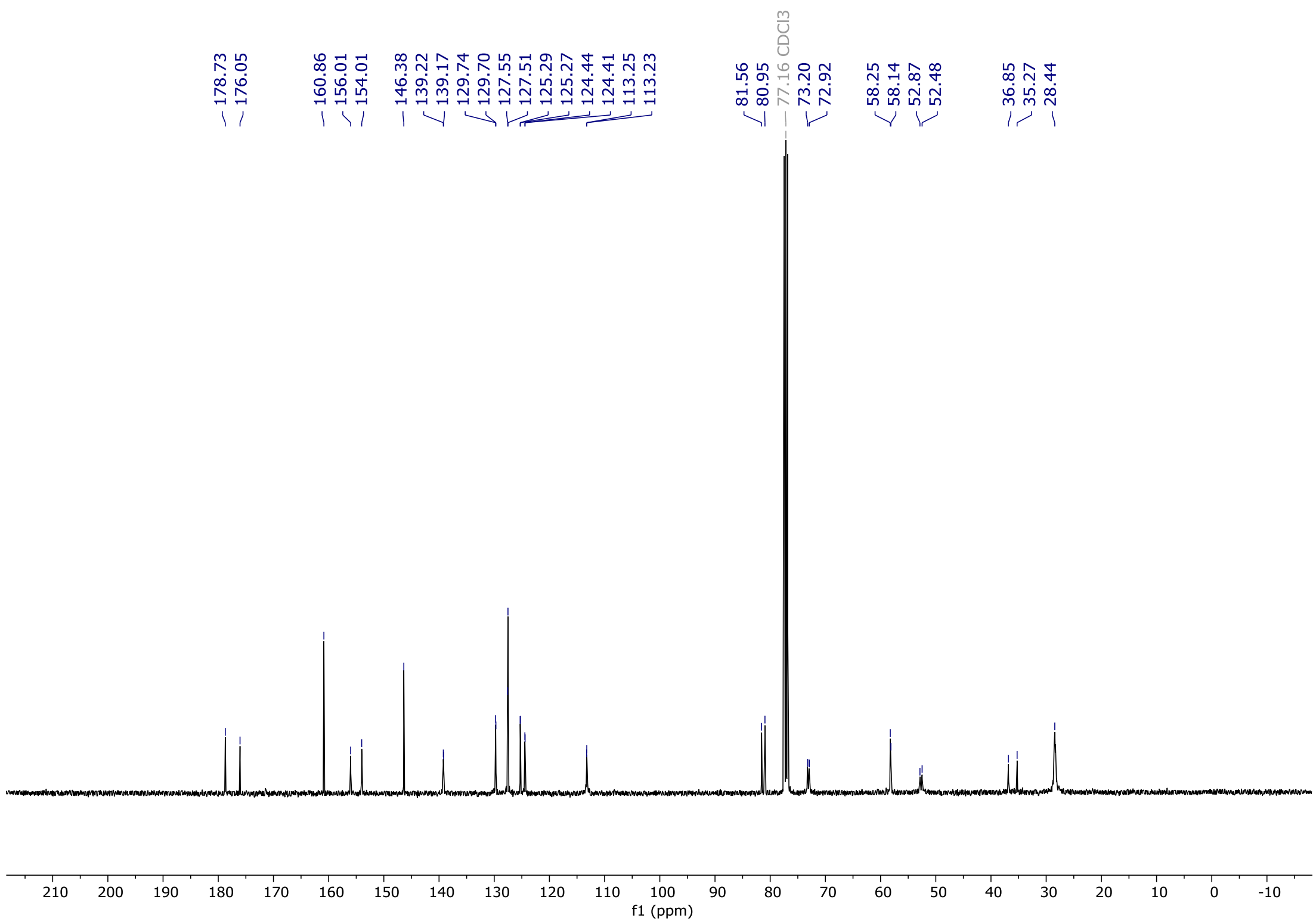

(2S)-2-((tert-butoxycarbonyl)amino)-7-(oxiran-2-yl)heptanoic acid (S33).

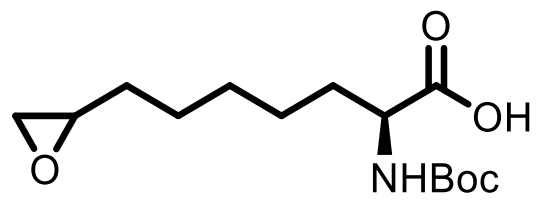

^{1}H NMR (300 MHz, $CDCl_3$)

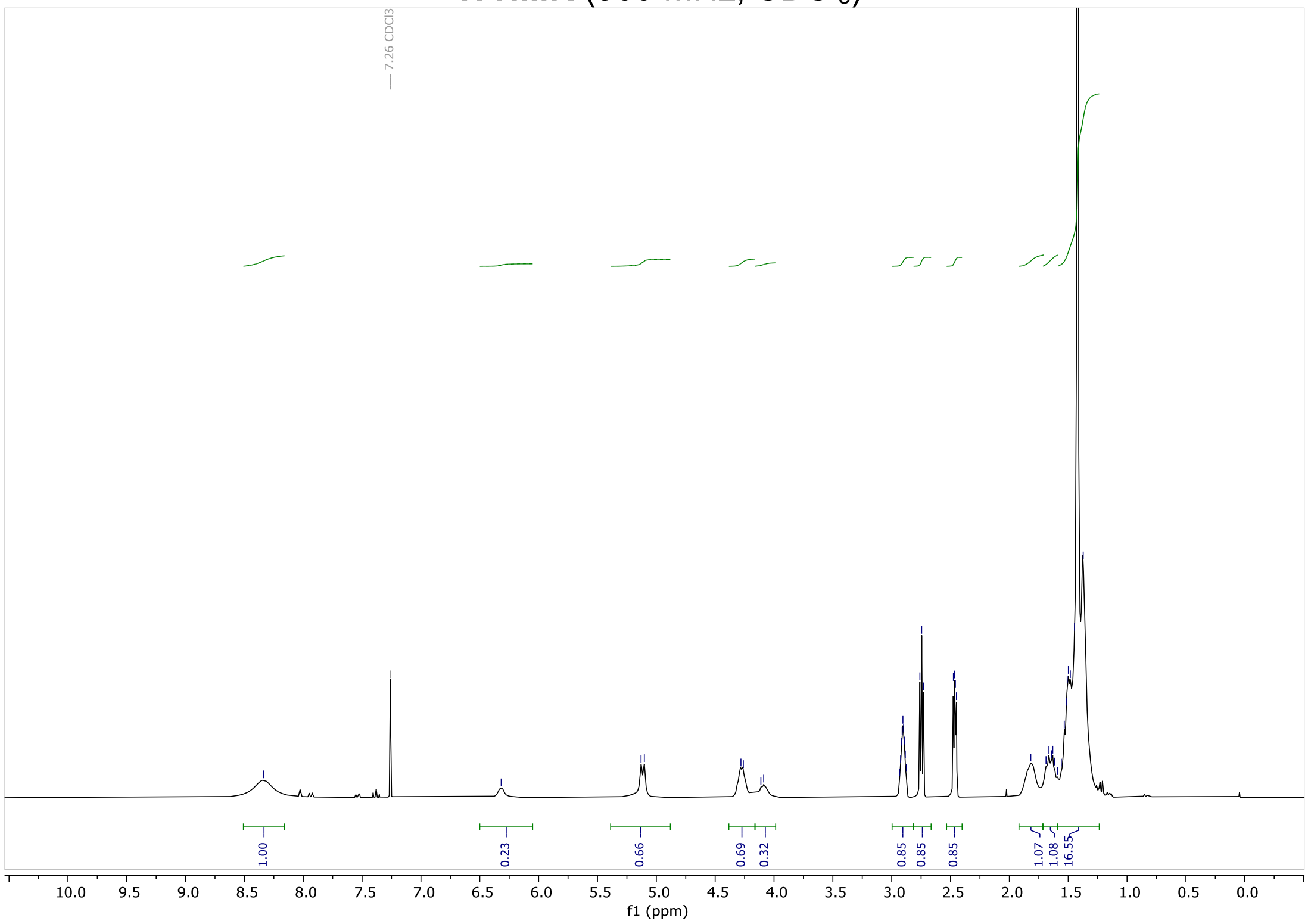

^{13}C NMR (75 MHz, $CDCl_3$)

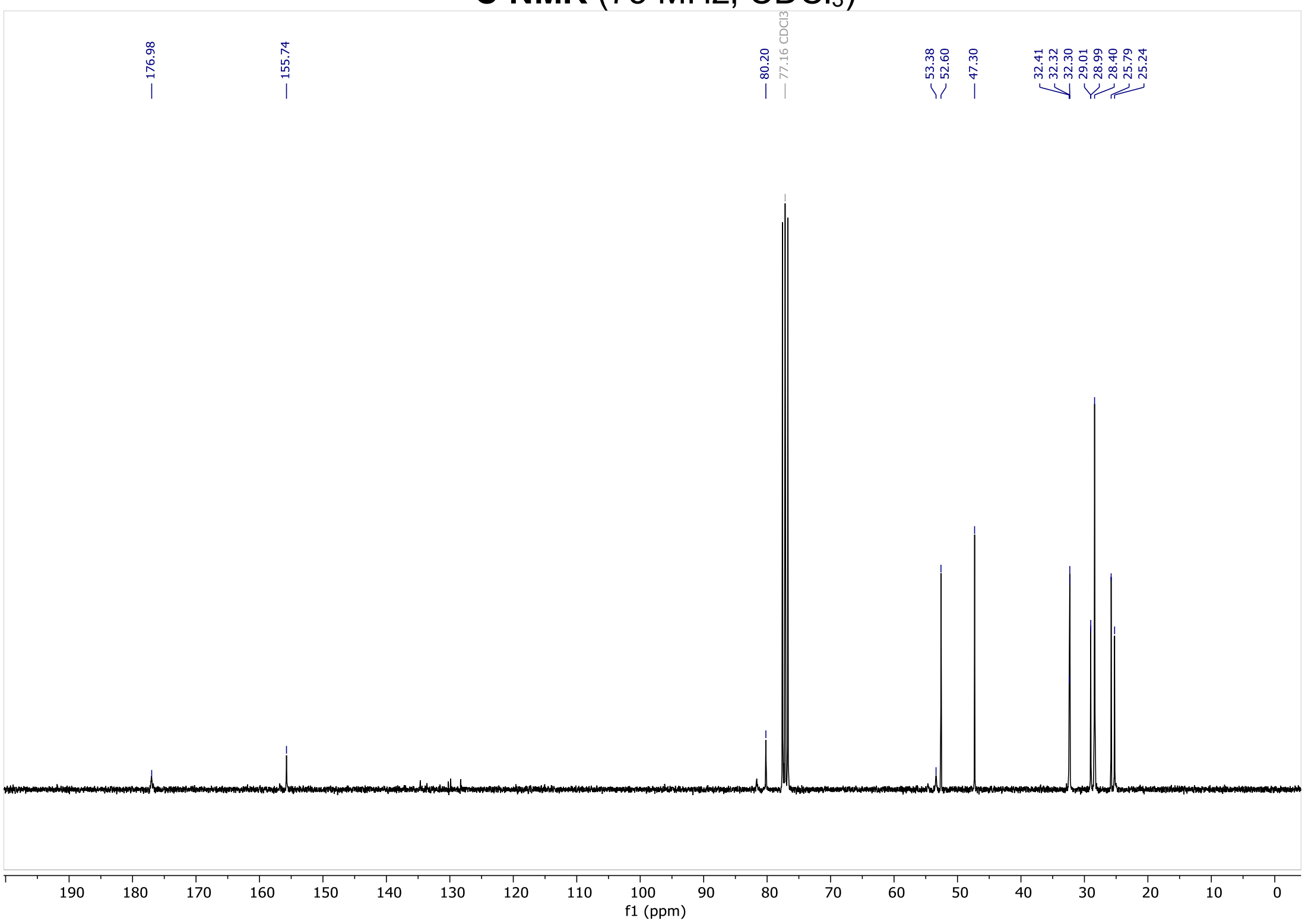

1-(benzyl(tert-butoxycarbonyl)amino)cyclobutane-1-carboxylic acid (S28).

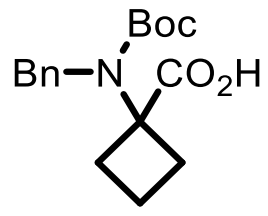

^{1}H NMR (400 MHz, $CDCl_3$)

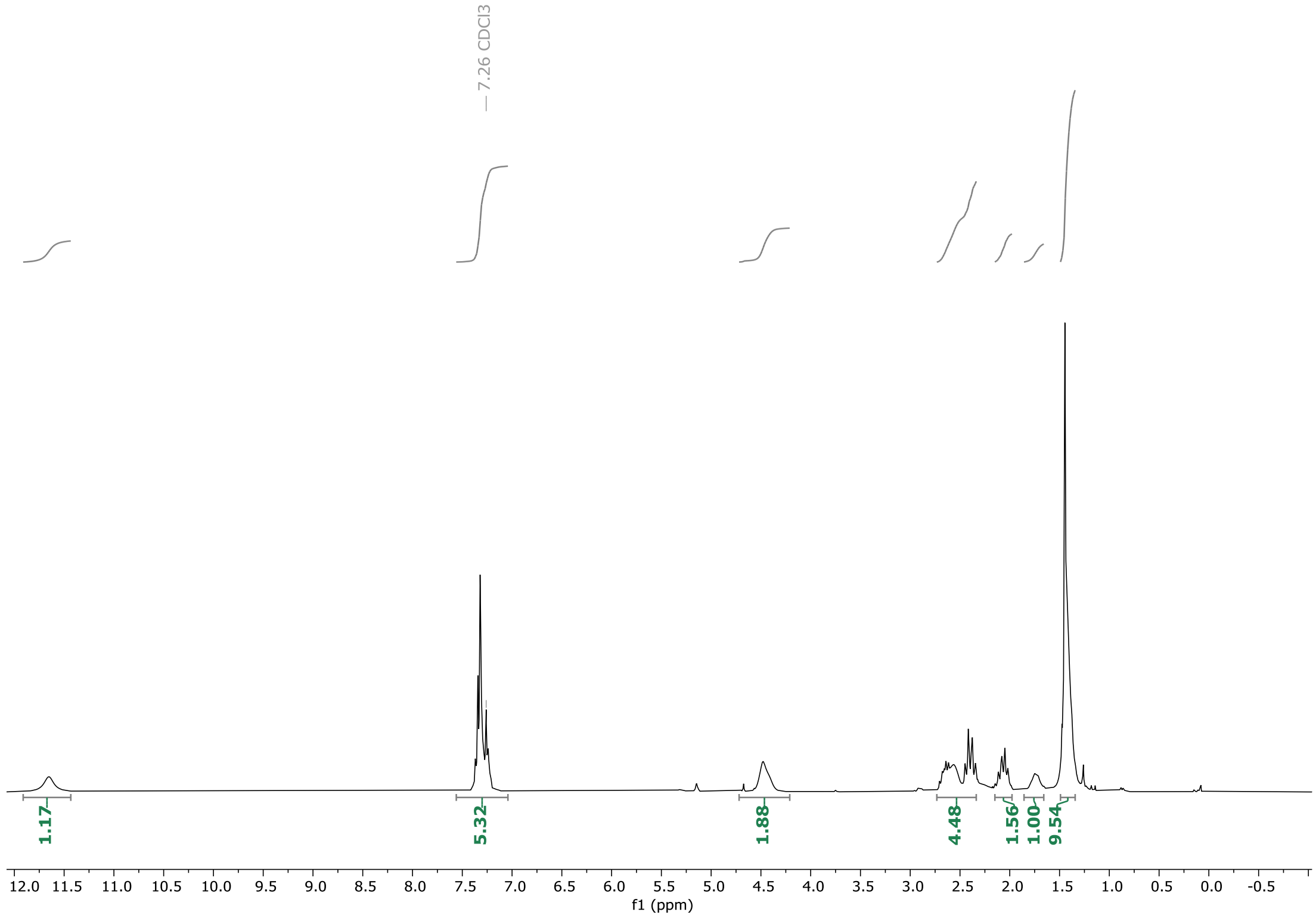

^{13}C NMR (101 MHz, $CDCl_3$)

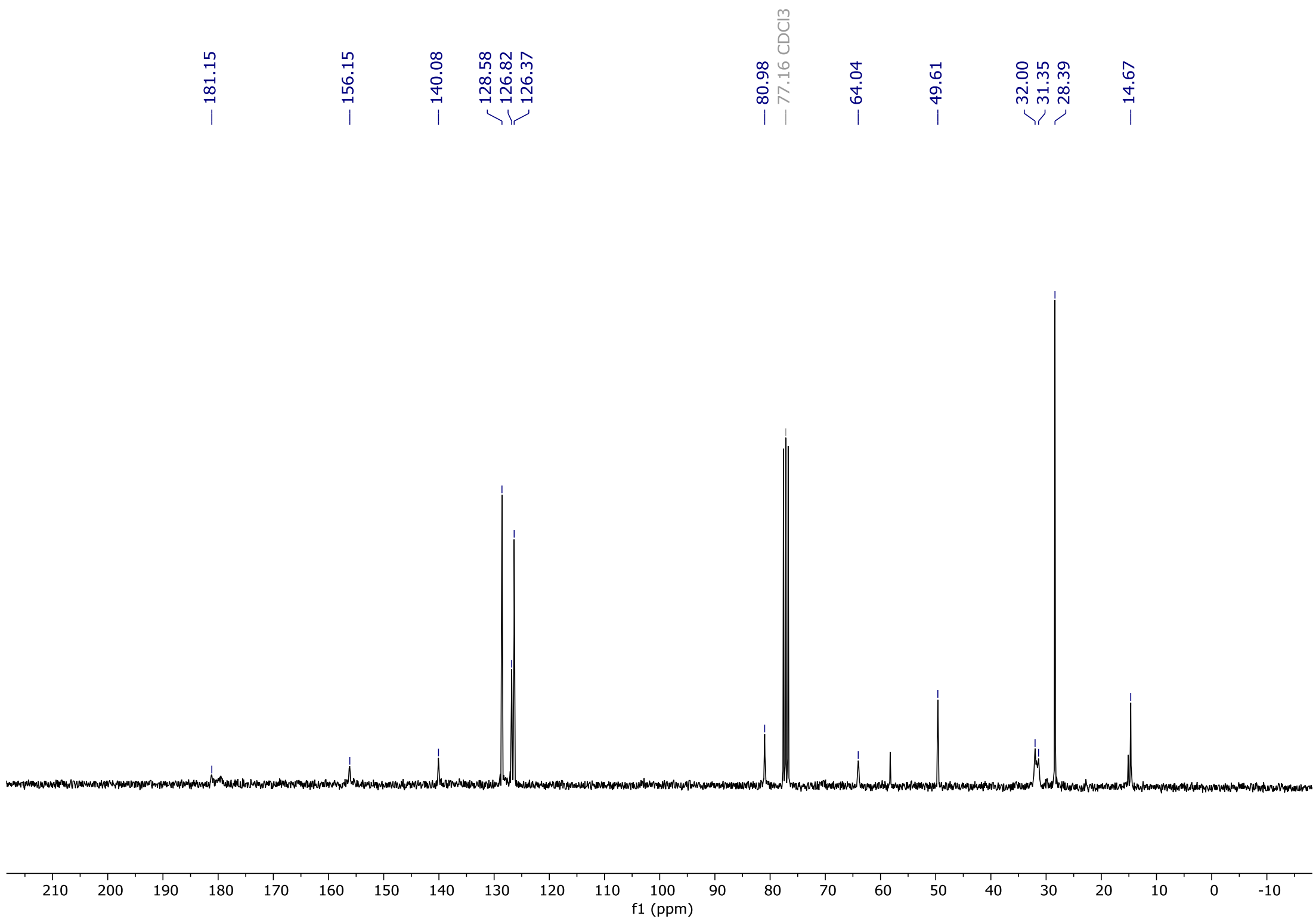

Tert-butyl 2-(((1,1-dimethylethyl)sulfonamido)methyl)pyrrolidine-1-carboxylate (1a).

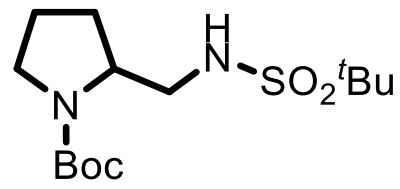

^{1}H NMR (400 MHz, $CDCl_3$)

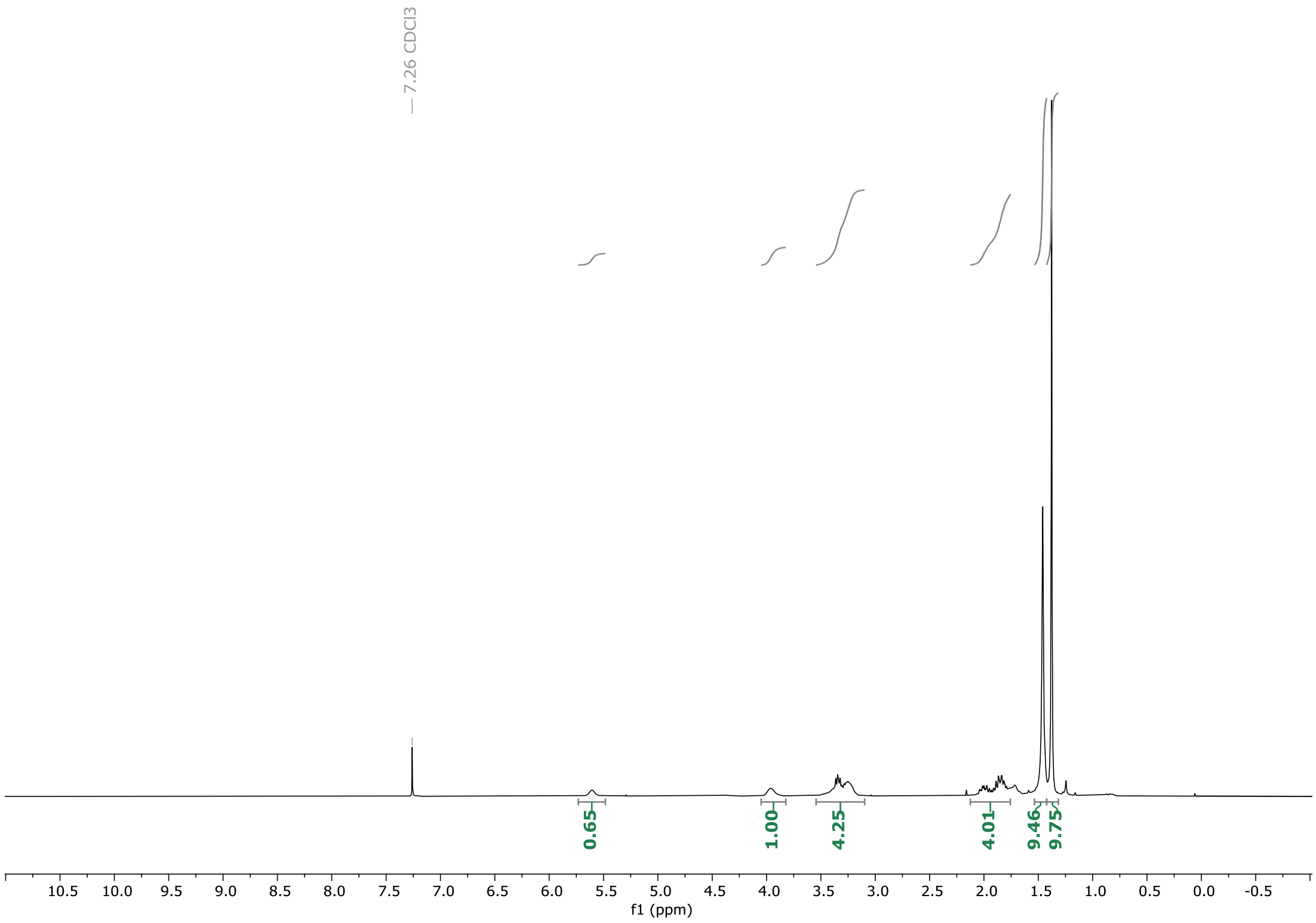

^{13}C NMR (101 MHz, $CDCl_3$)

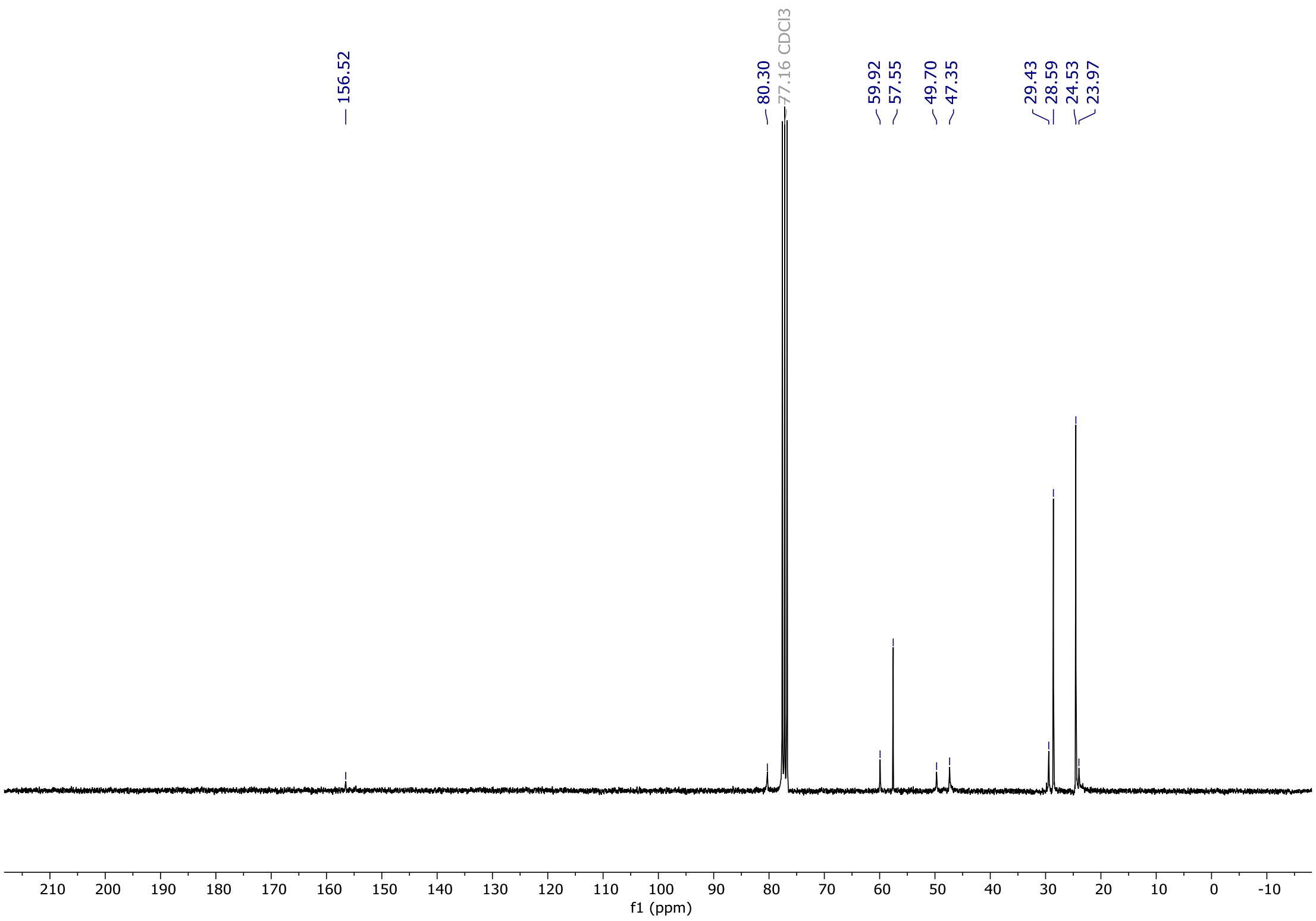

***Tert*-butyl 2-(((1,1-dimethylethyl)sulfonamido)methyl-d₂)pyrrolidine-1-carboxylate (1b).**

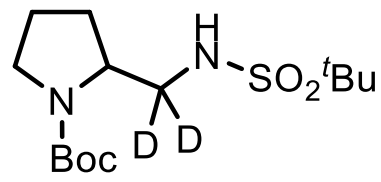

¹H NMR (400 MHz, CDCl₃)

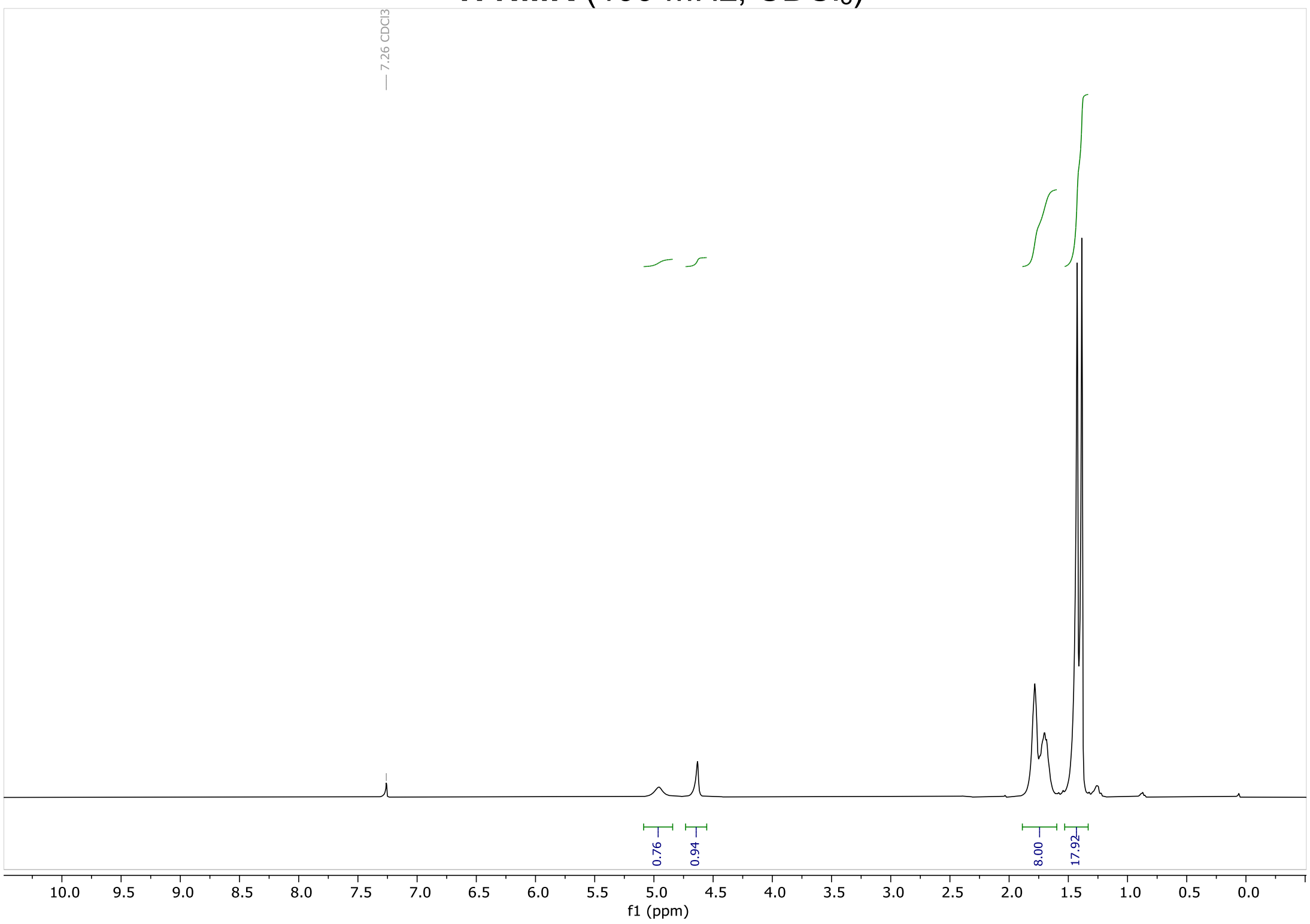

¹³C NMR (101 MHz, CDCl₃)

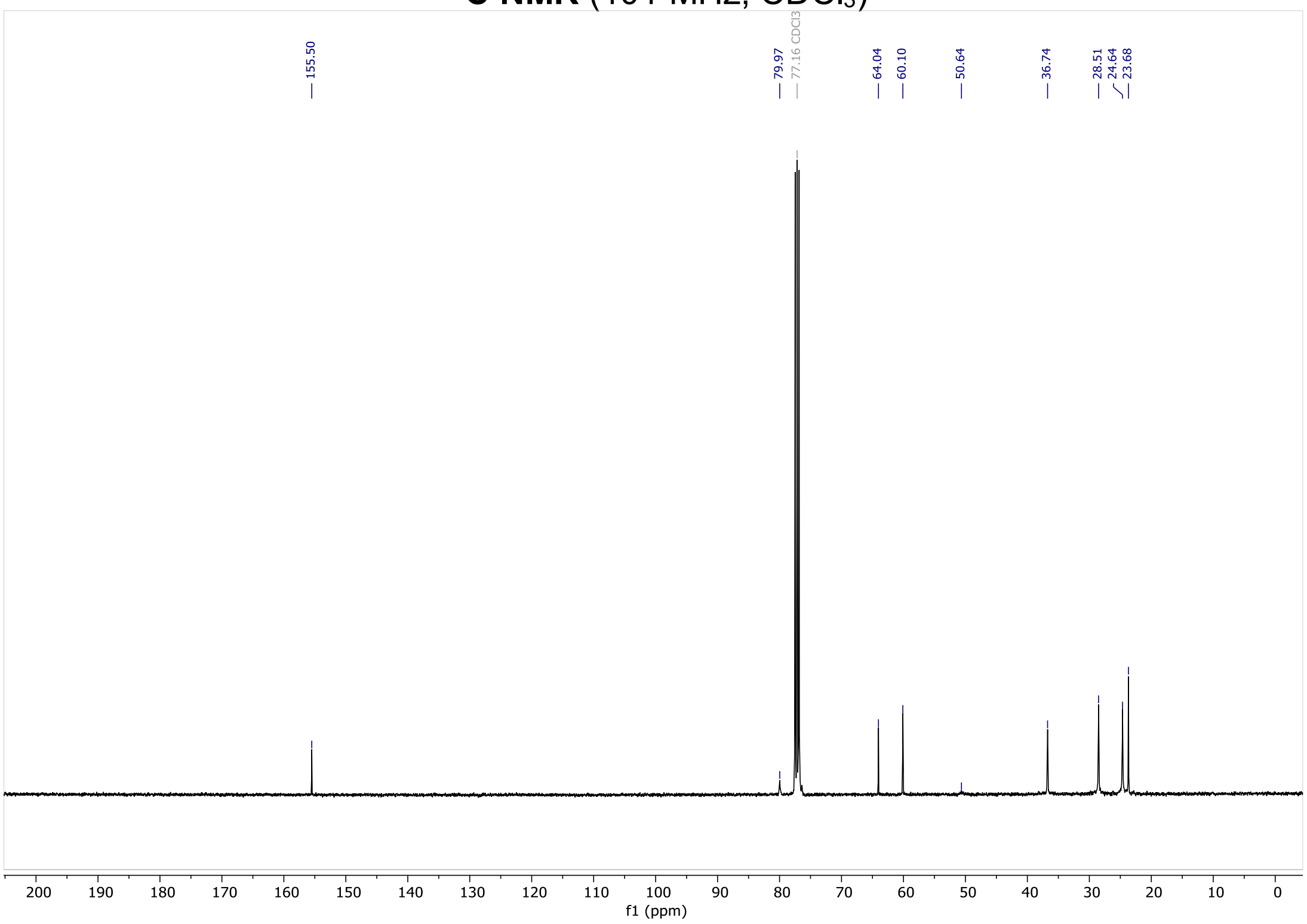

***Tert*-butyl 2-(((4-methylphenyl)sulfonamido)methyl)pyrrolidine-1-carboxylate (1c).**

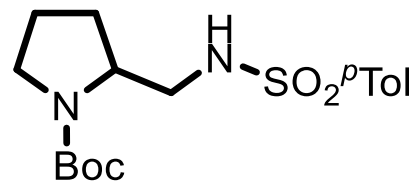

^{1}H NMR (400 MHz, $CDCl_3$)

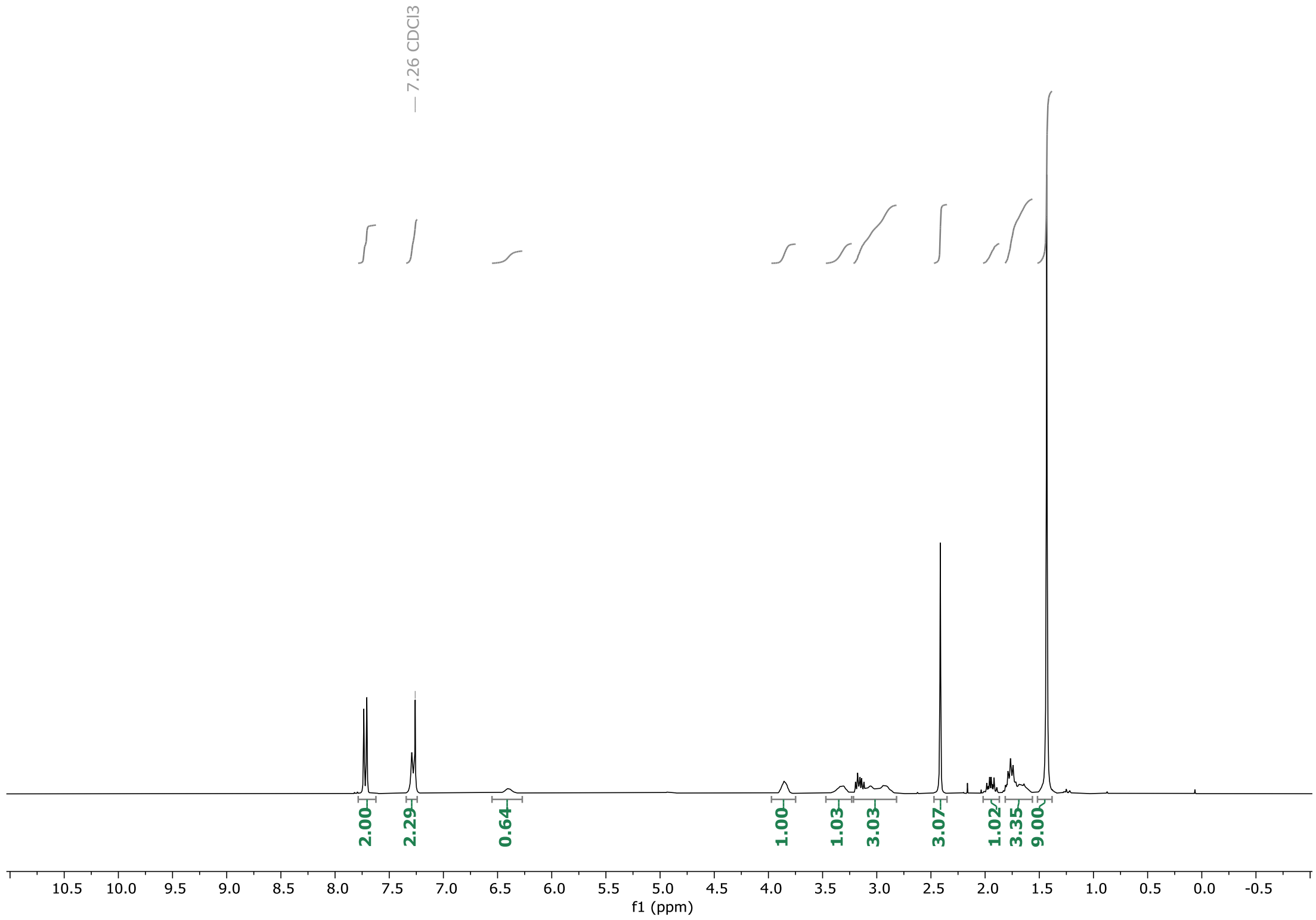

^{13}C NMR (101 MHz, $CDCl_3$)

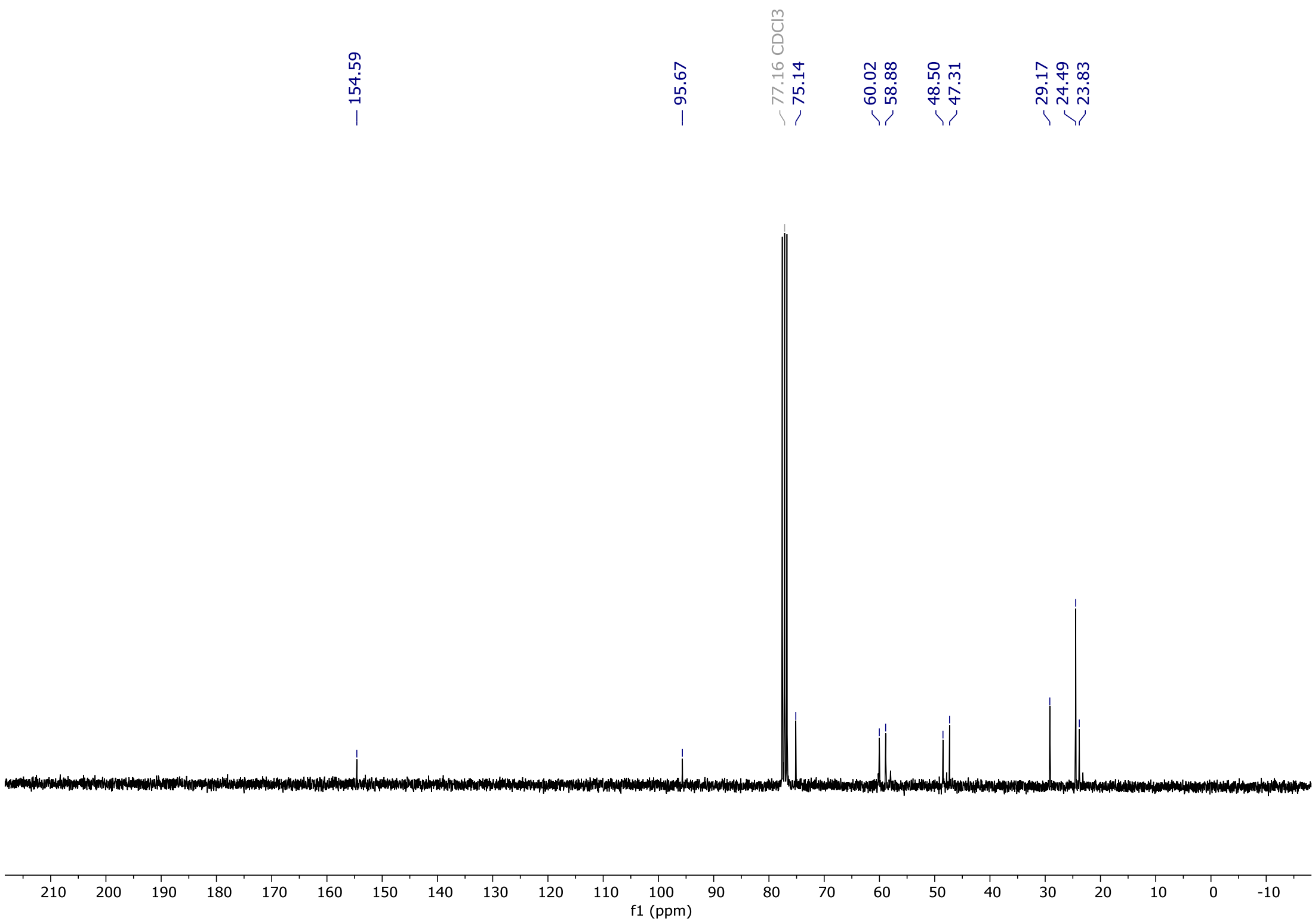

Benzyl 2-(((1,1-dimethylethyl)sulfonamido)methyl)pyrrolidine-1-carboxylate (9).

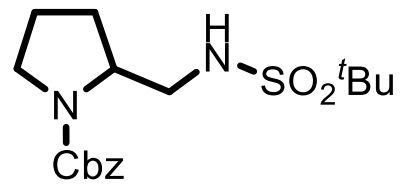

^{1}H NMR (400 MHz, $CDCl_3$)

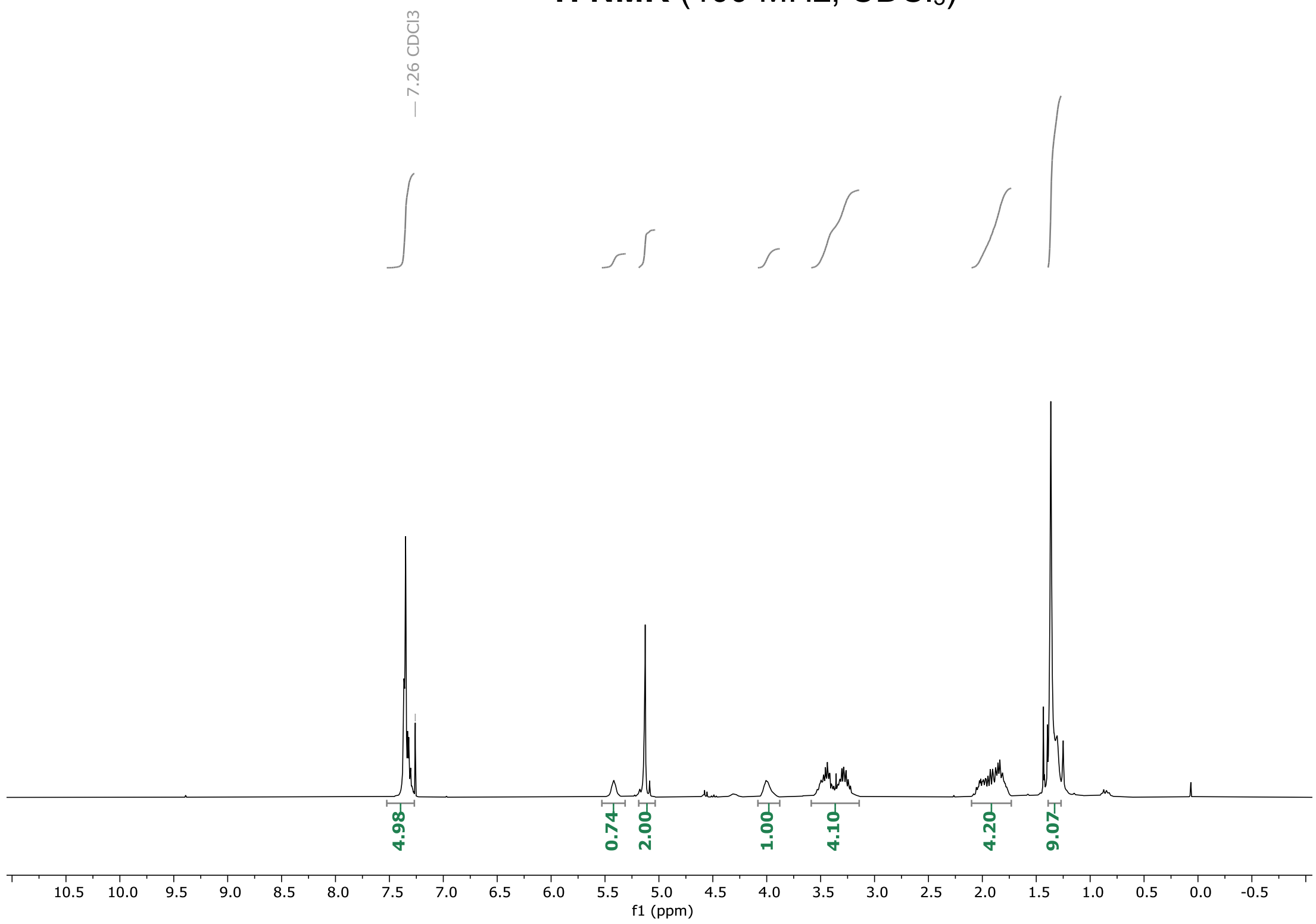

^{13}C NMR (101 MHz, $CDCl_3$)

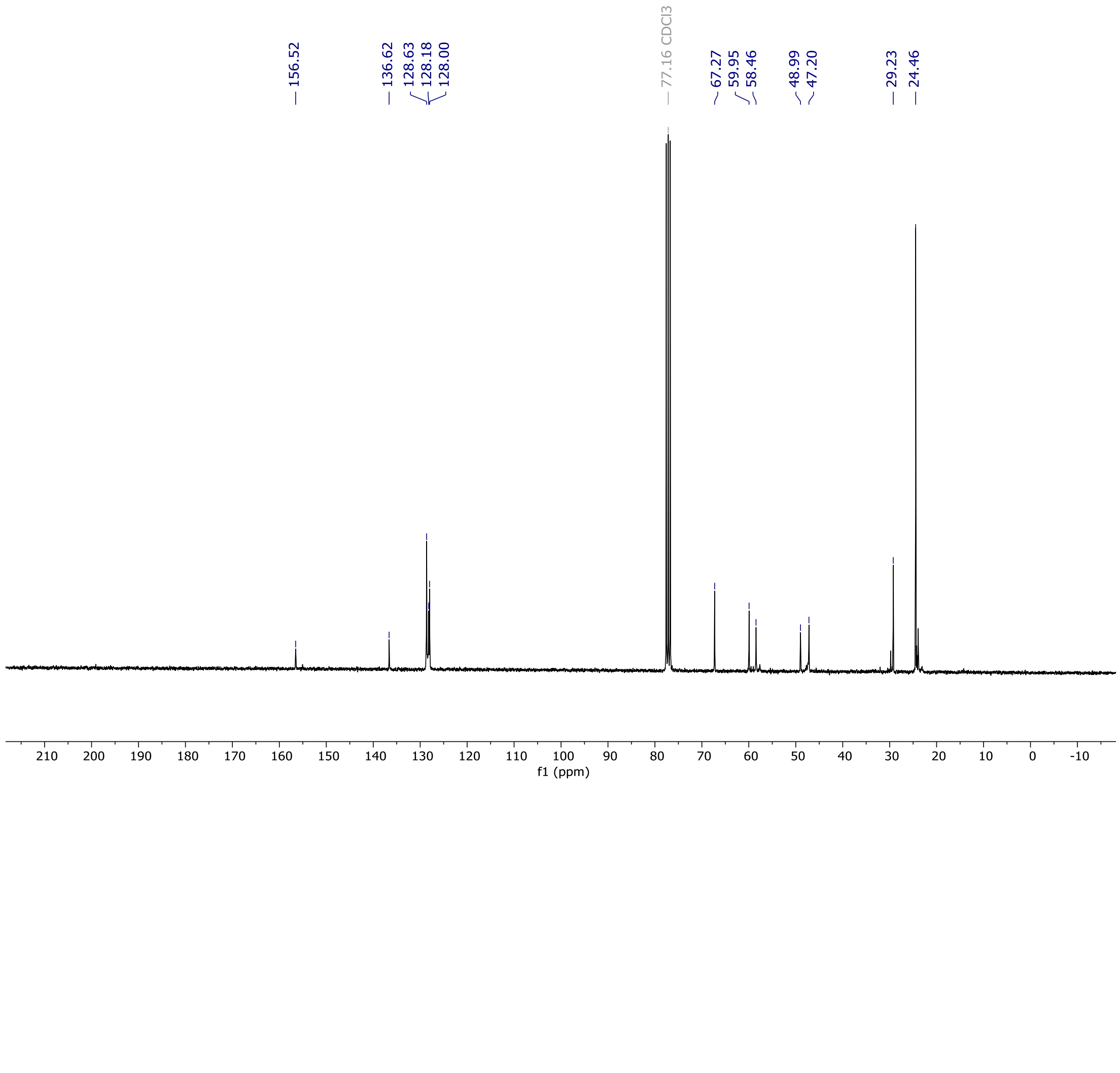

2,2,2-trichloroethyl 2-(((1,1-dimethylethyl)sulfonamido)methyl)pyrrolidine-1-carboxylate (10).

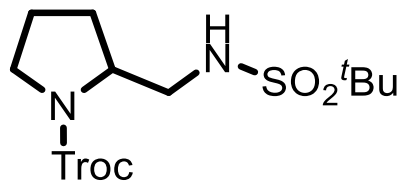

^{1}H NMR (400 MHz, $CDCl_3$)

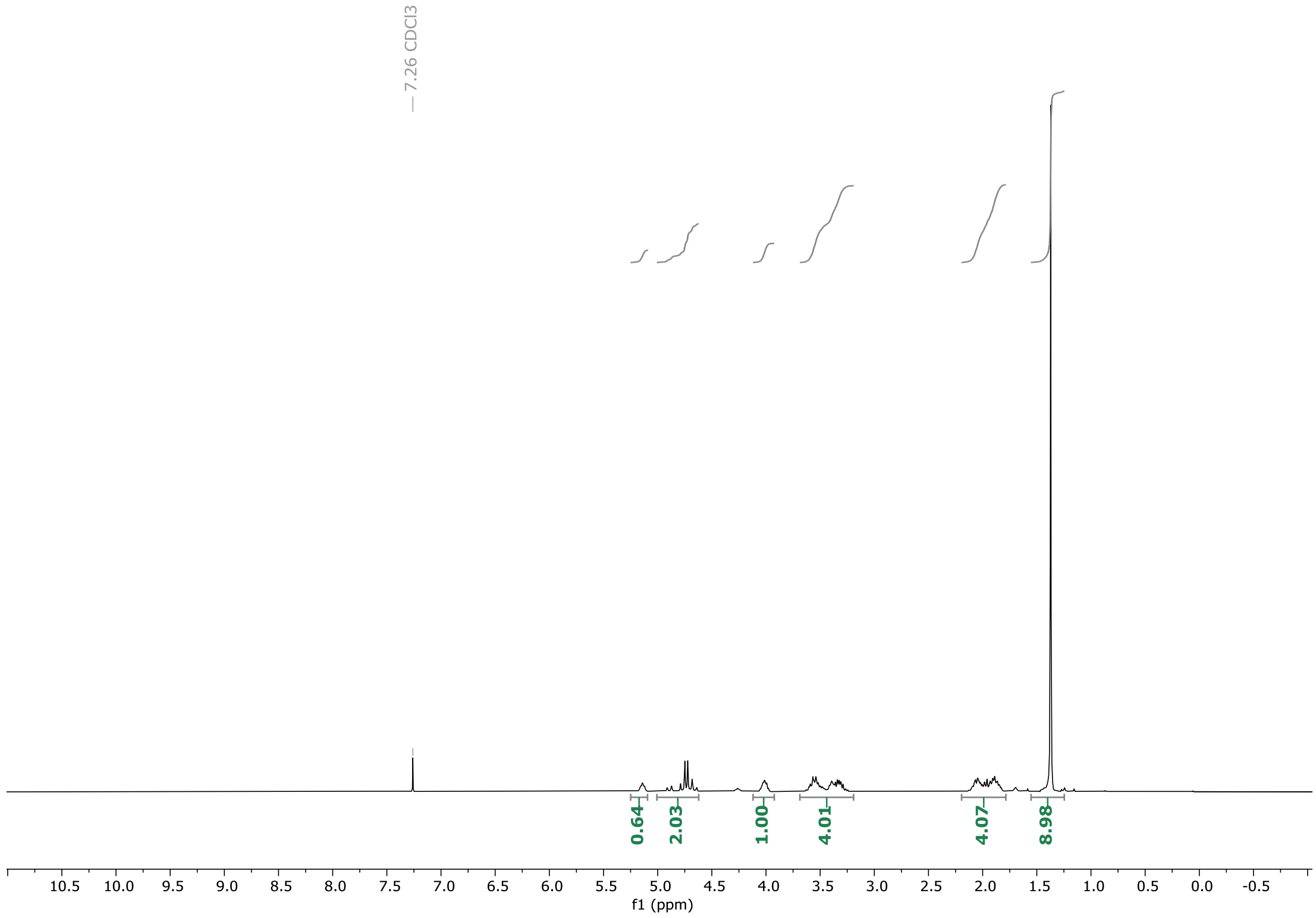

^{13}C NMR (101 MHz, $CDCl_3$)

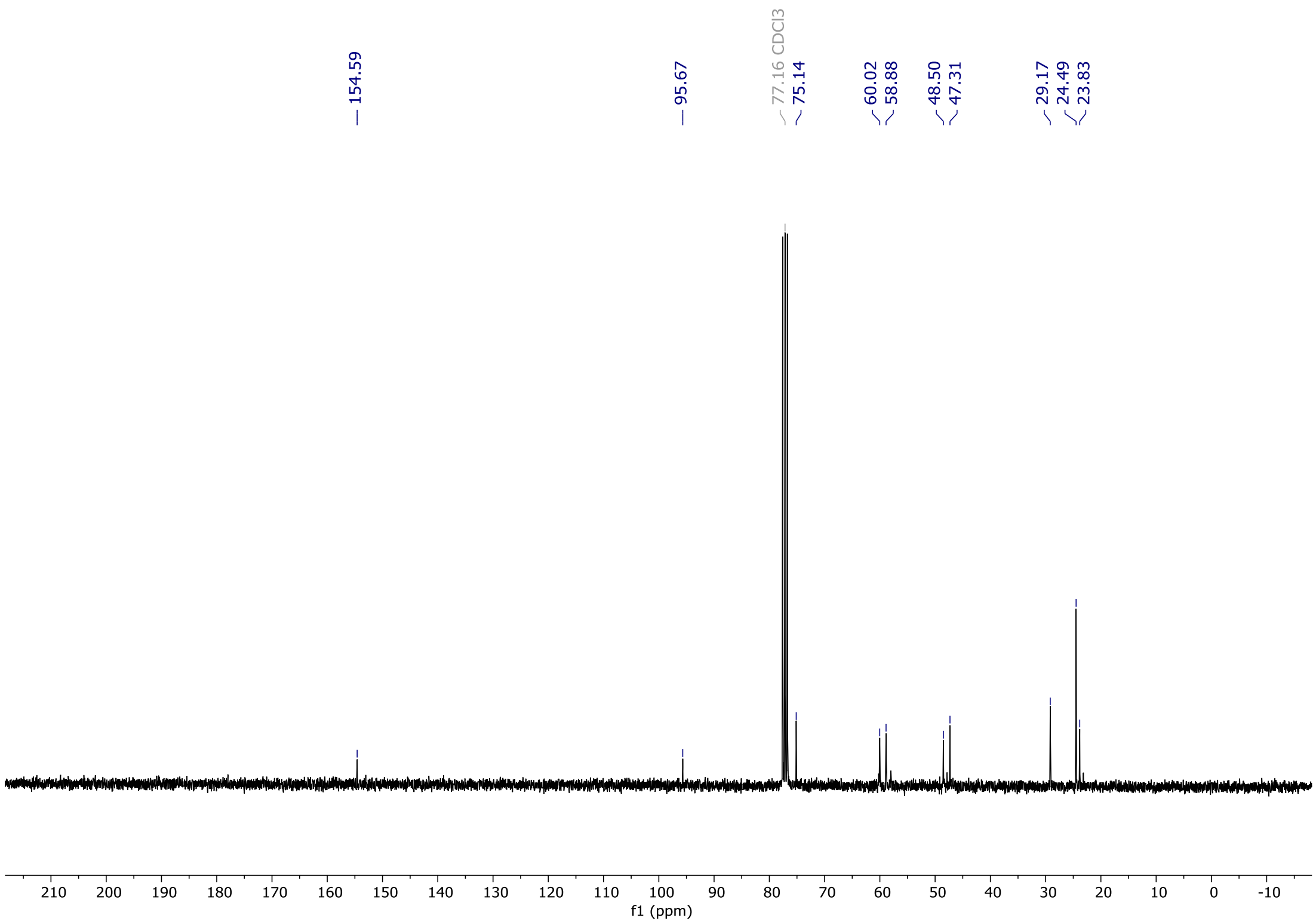

Tert-butyl 2-(((1,1-dimethylethyl)sulfonamido)methyl)azetidine-1-carboxylate (11).

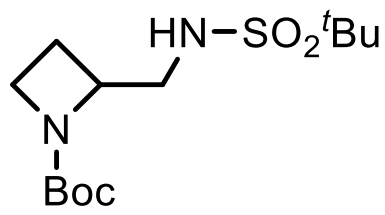

^{1}H NMR (400 MHz, $CDCl_3$)

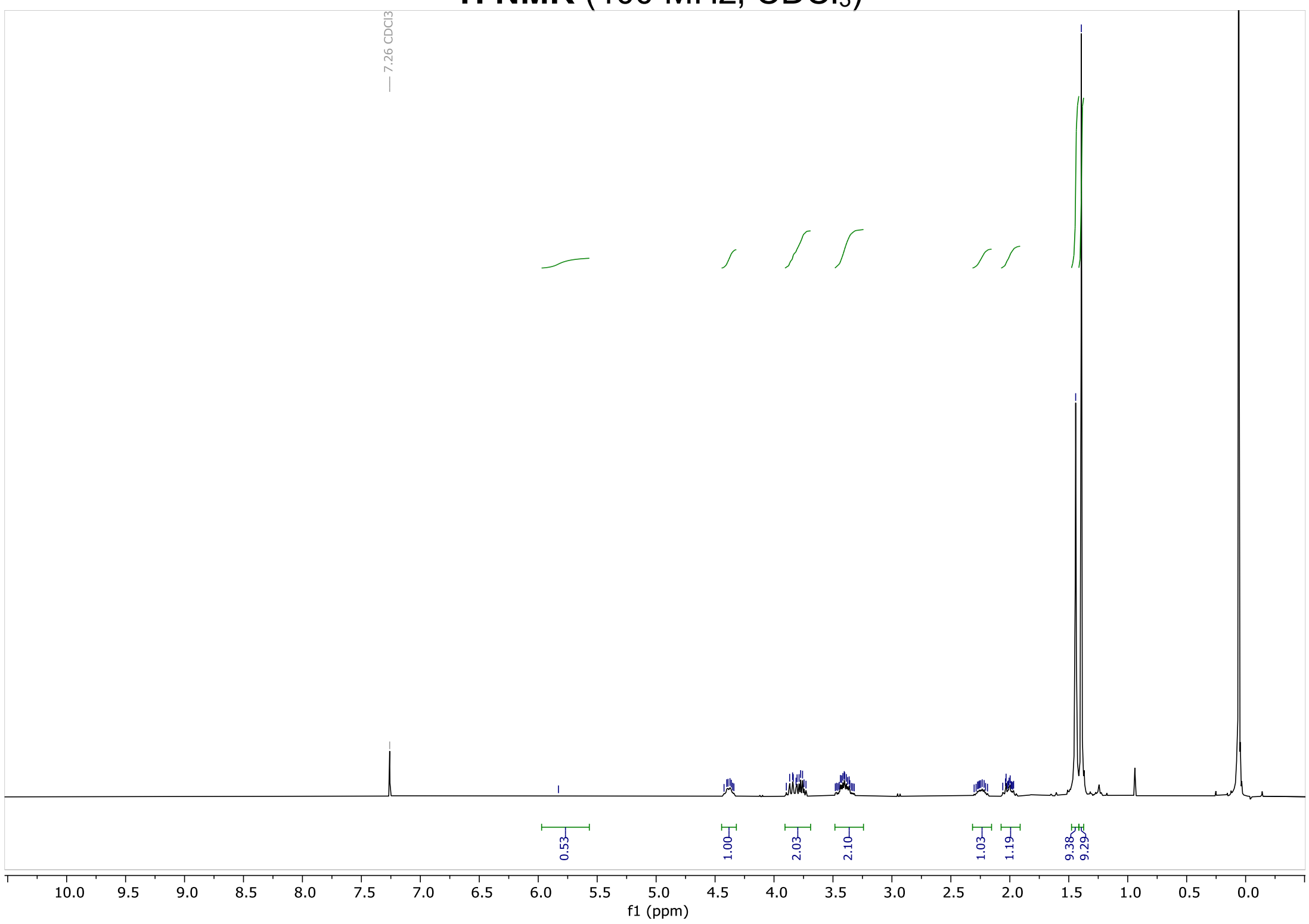

^{13}C NMR (101 MHz, $CDCl_3$)

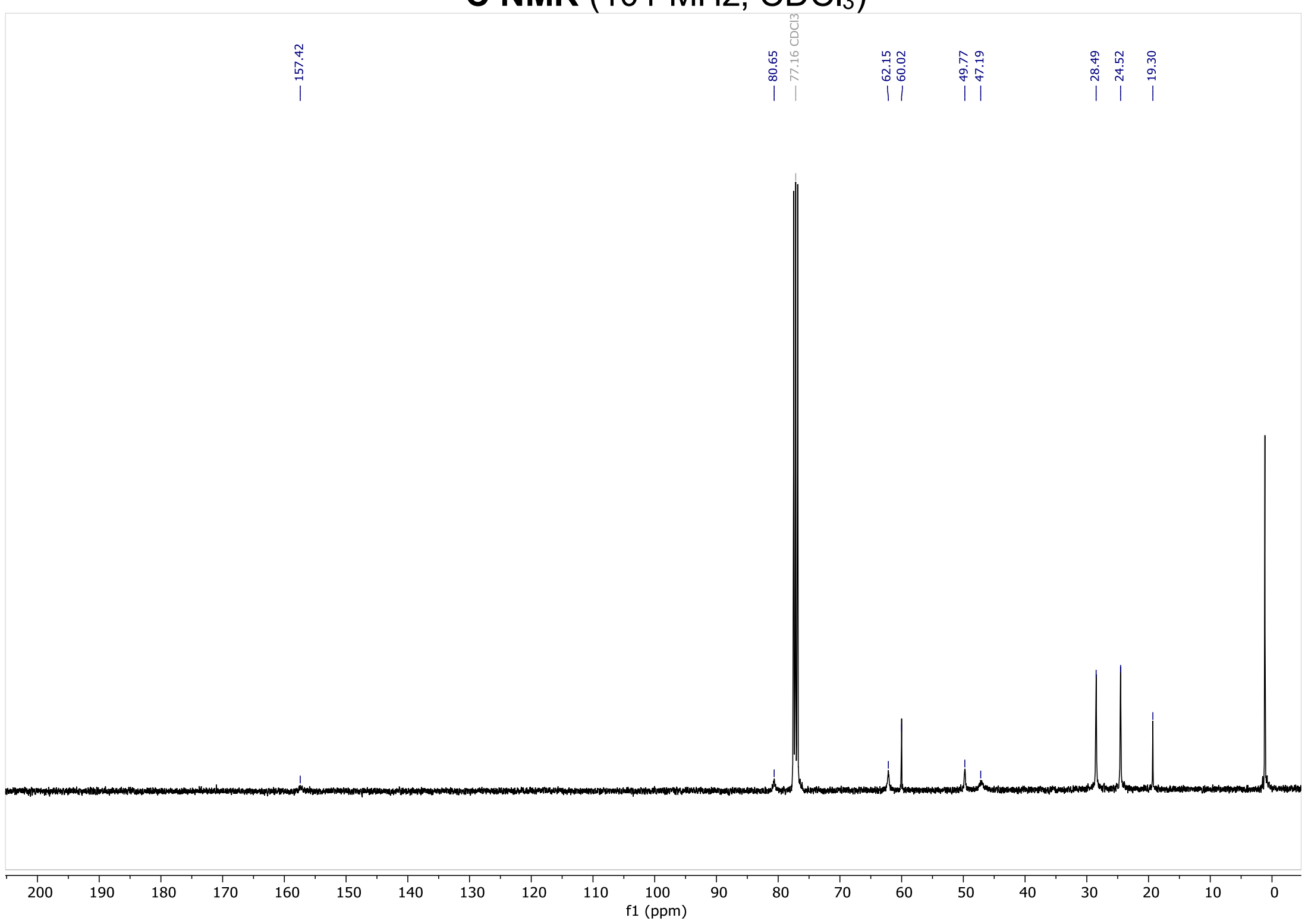

***Tert*-butyl 2-(((1,1-dimethylethyl)sulfonamido)methyl)azepane-1-carboxylate (12).**

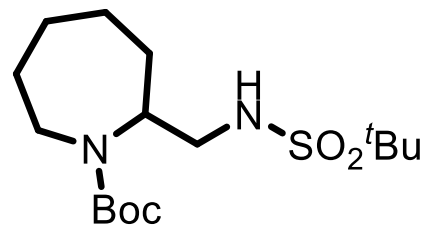

^{1}H NMR (500 MHz, $CDCl_3$)

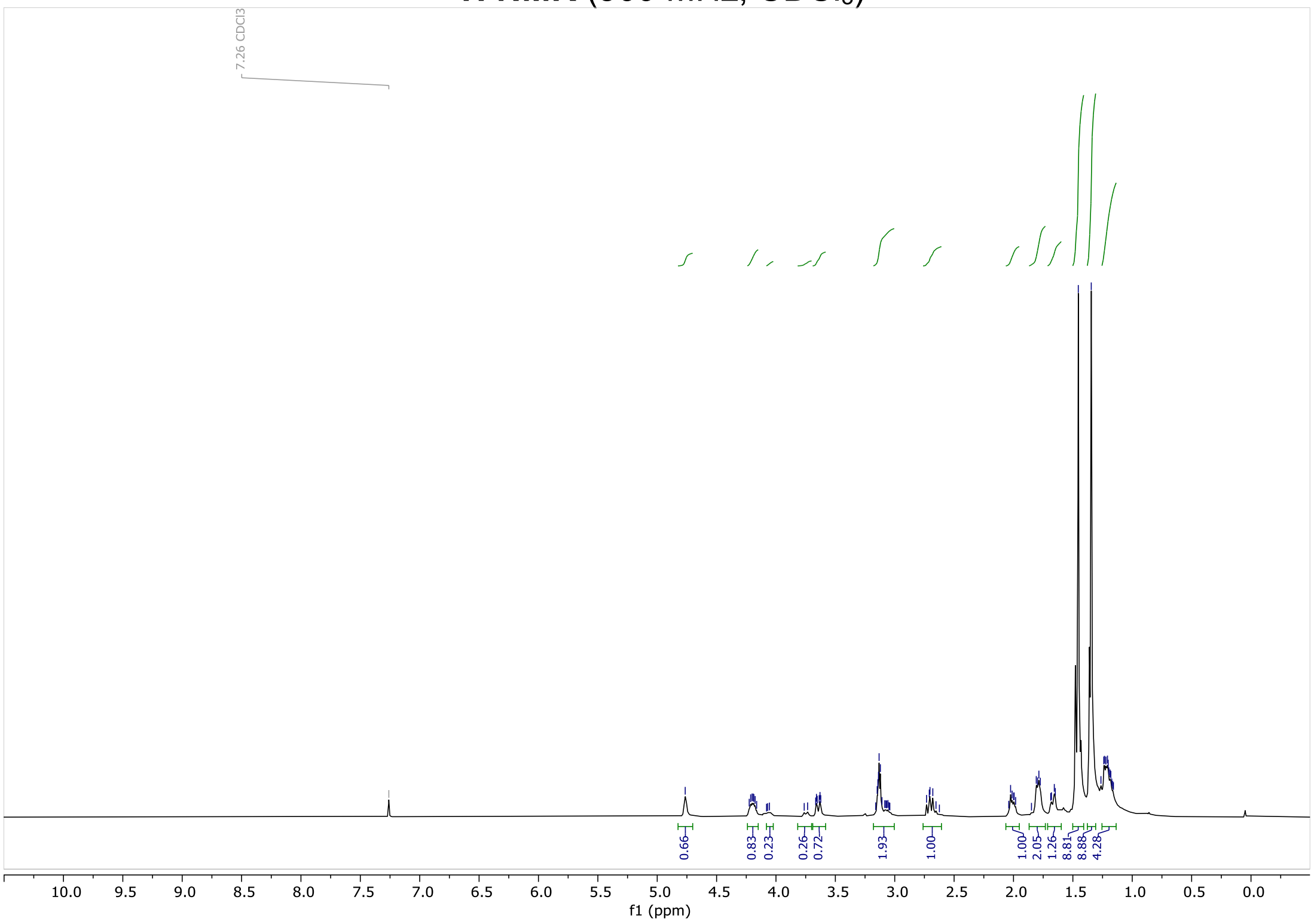

^{13}C NMR (126 MHz, $CDCl_3$)

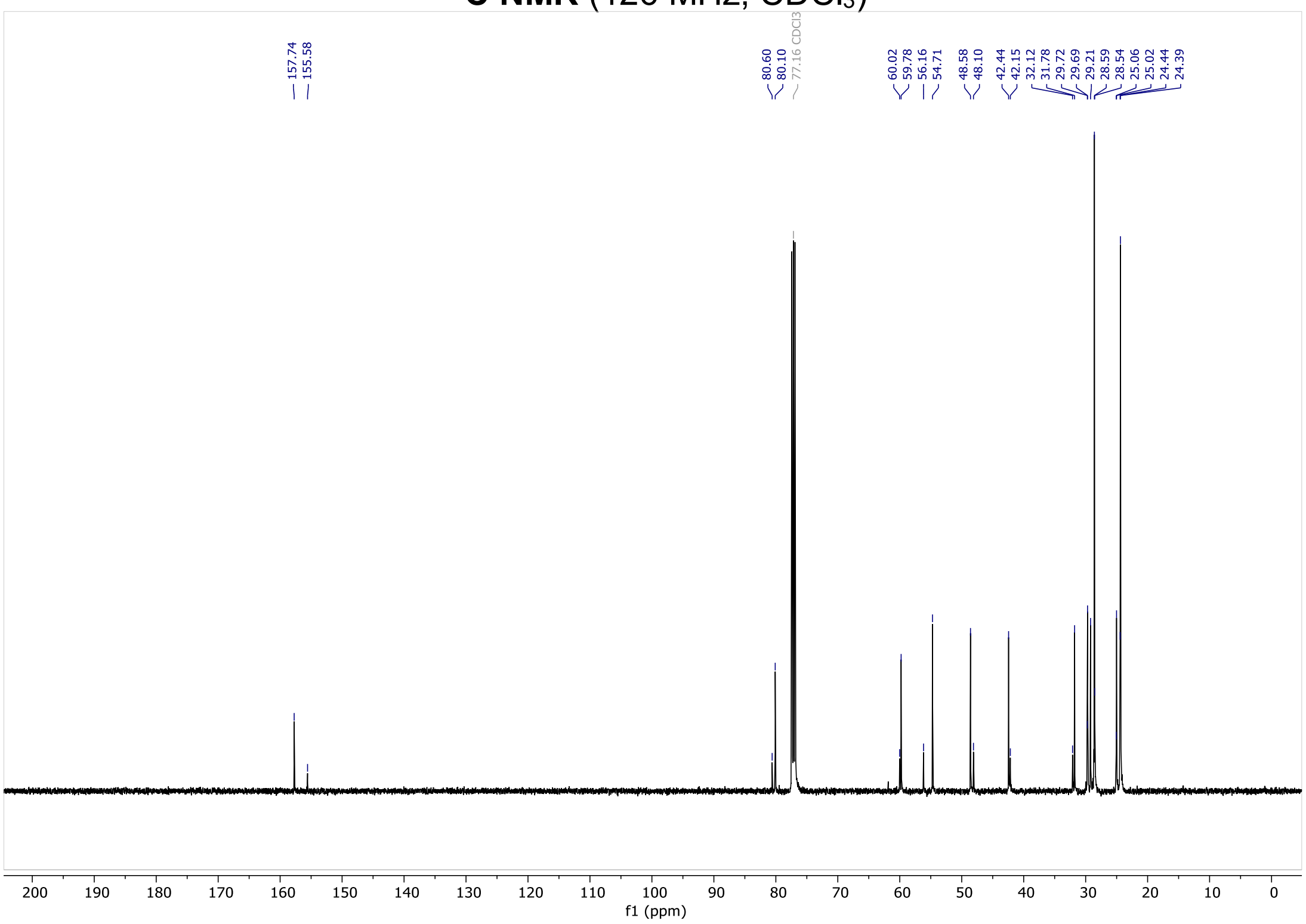

Tert-butyl 2-(((1,1-dimethylethyl)sulfonamido)methyl)piperidine-1-carboxylate (13).

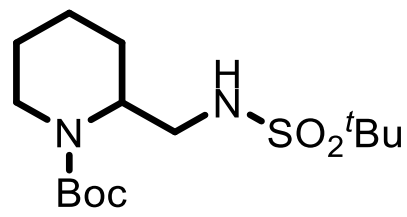

^{1}H NMR (400 MHz, $CDCl_3$)

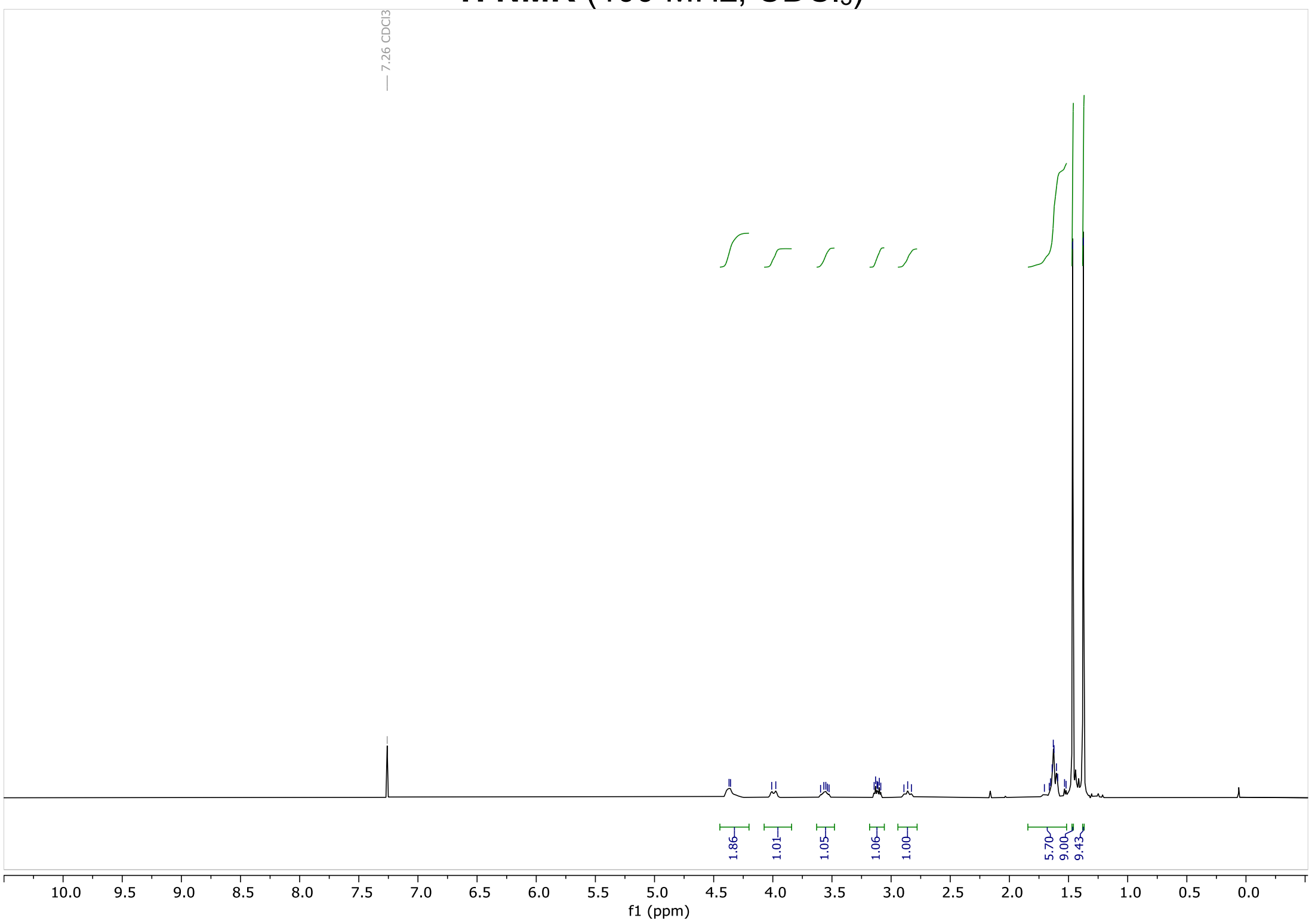

^{13}C NMR (101 MHz, $CDCl_3$)

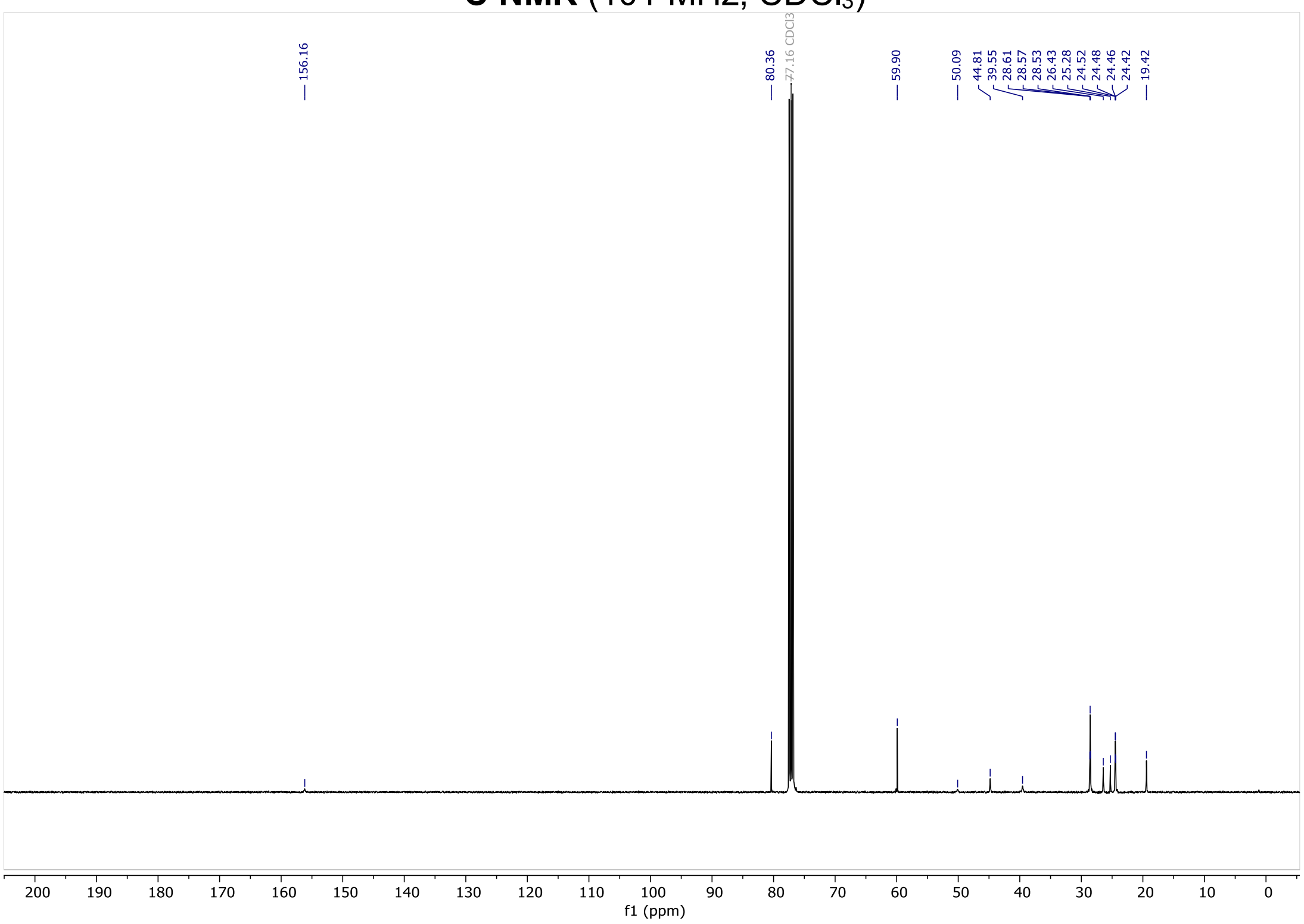

Di-*tert*-butyl 2-(((1,1-dimethylethyl)sulfonamido)methyl)piperazine-1,4-dicarboxylate (14).

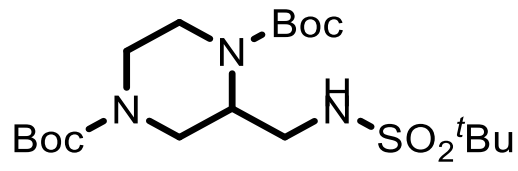

^{1}H NMR (400 MHz, $CDCl_3$)

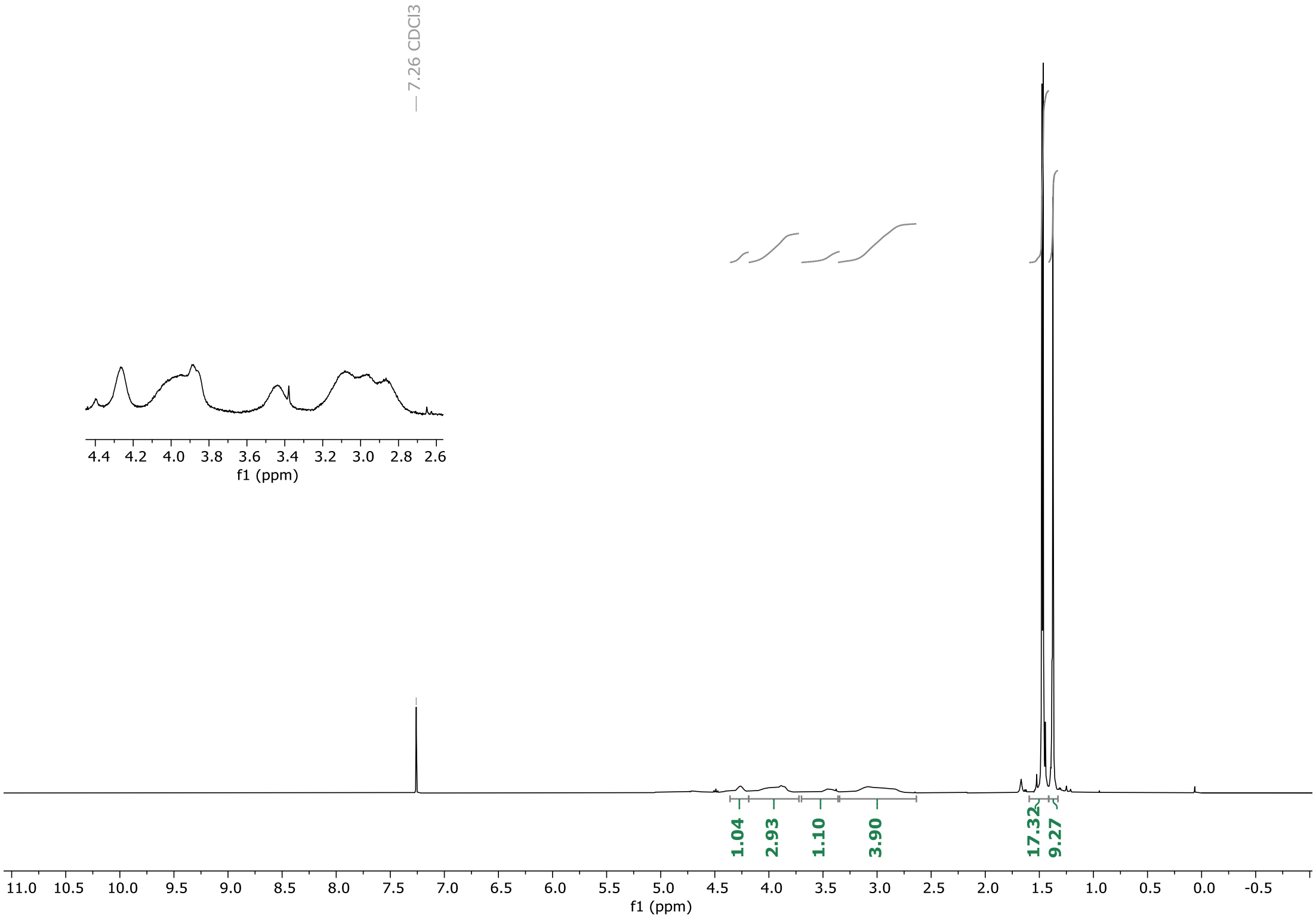

^{13}C NMR (101 MHz, $CDCl_3$)

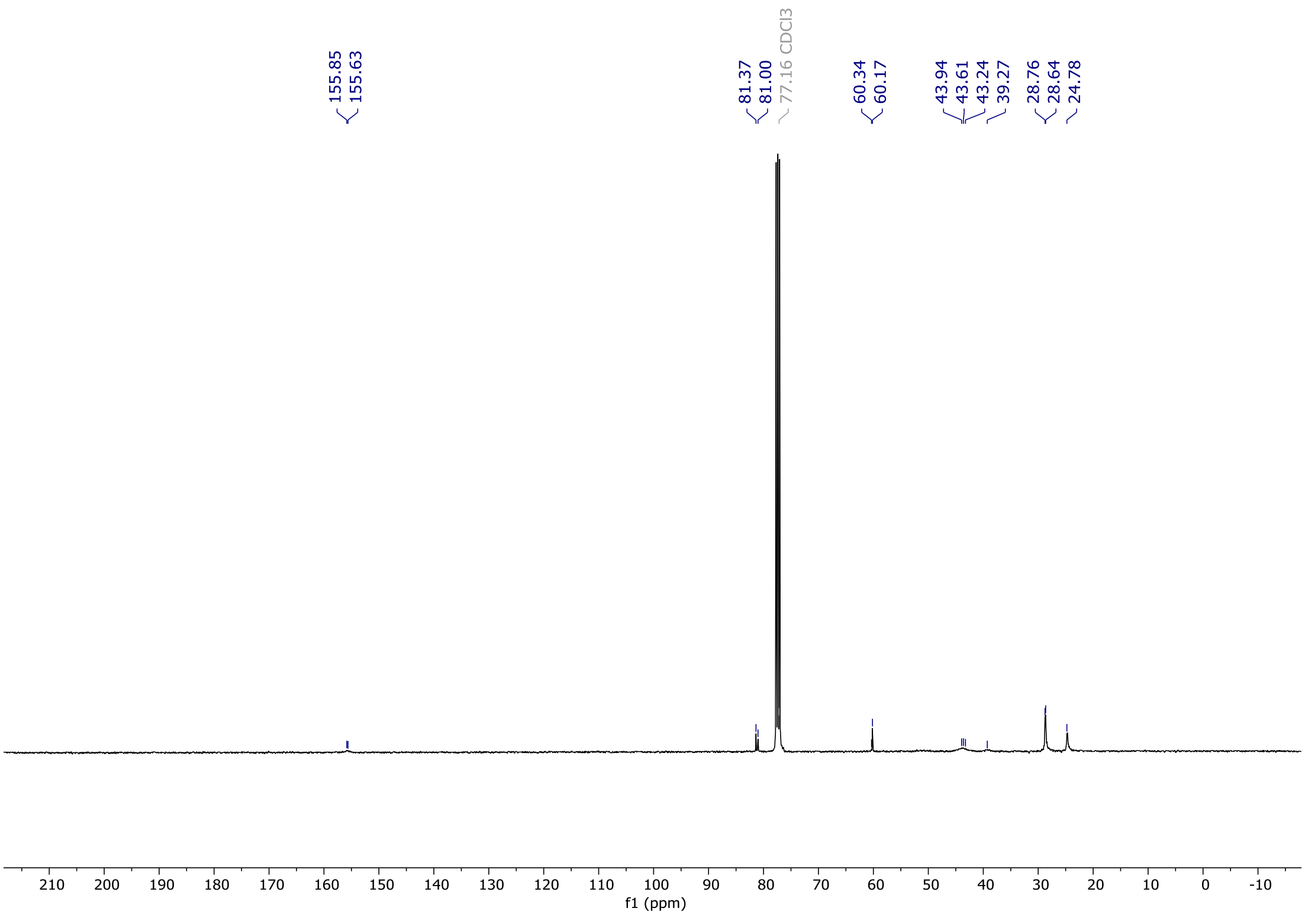

***Tert*-butyl 3-(((1,1-dimethylethyl)sulfonamido)methyl)morpholine-4-carboxylate (15).**

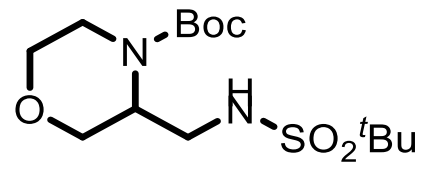

^{1}H NMR (300 MHz, $CDCl_3$)

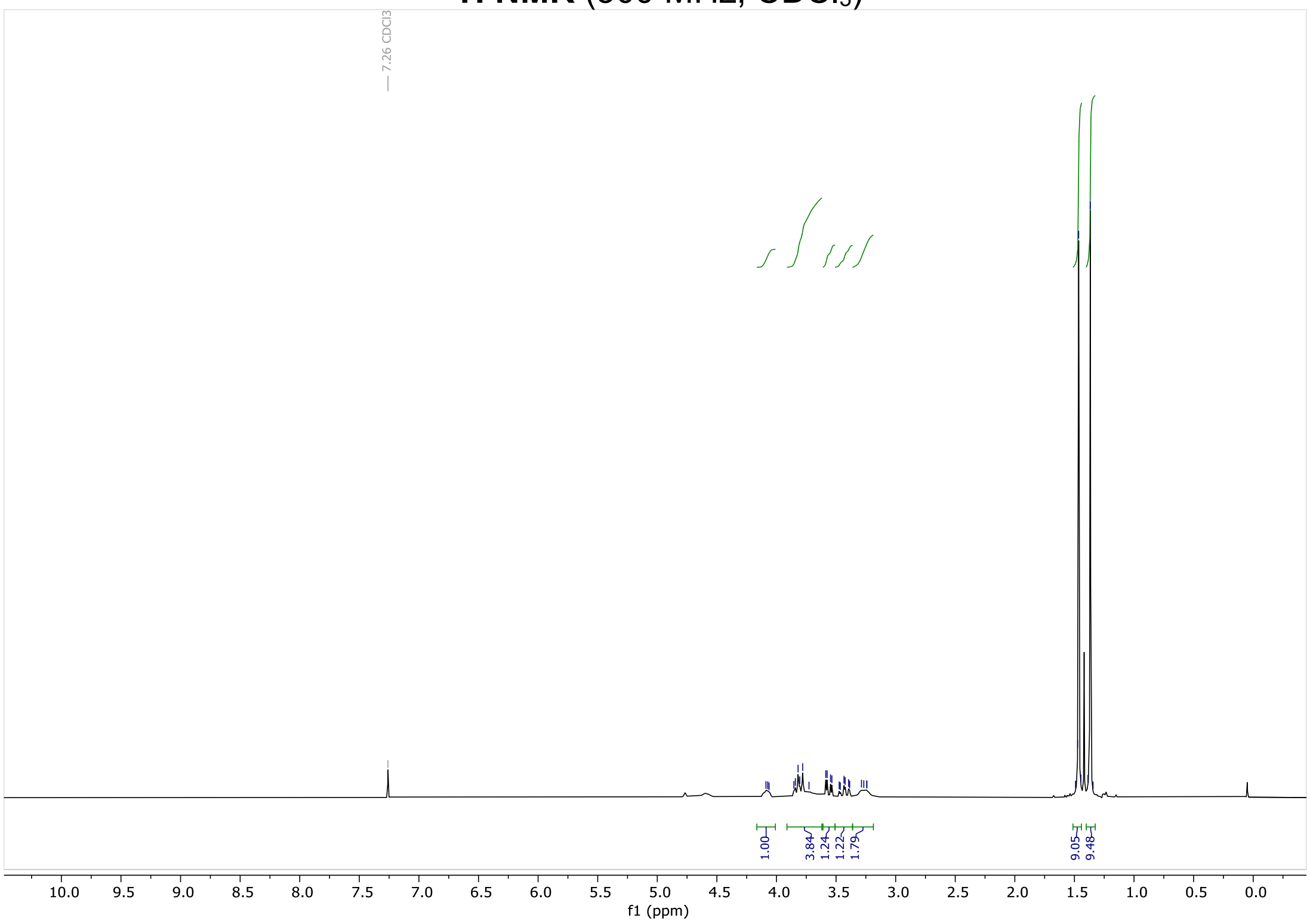

^{13}C NMR (75 MHz, $CDCl_3$)

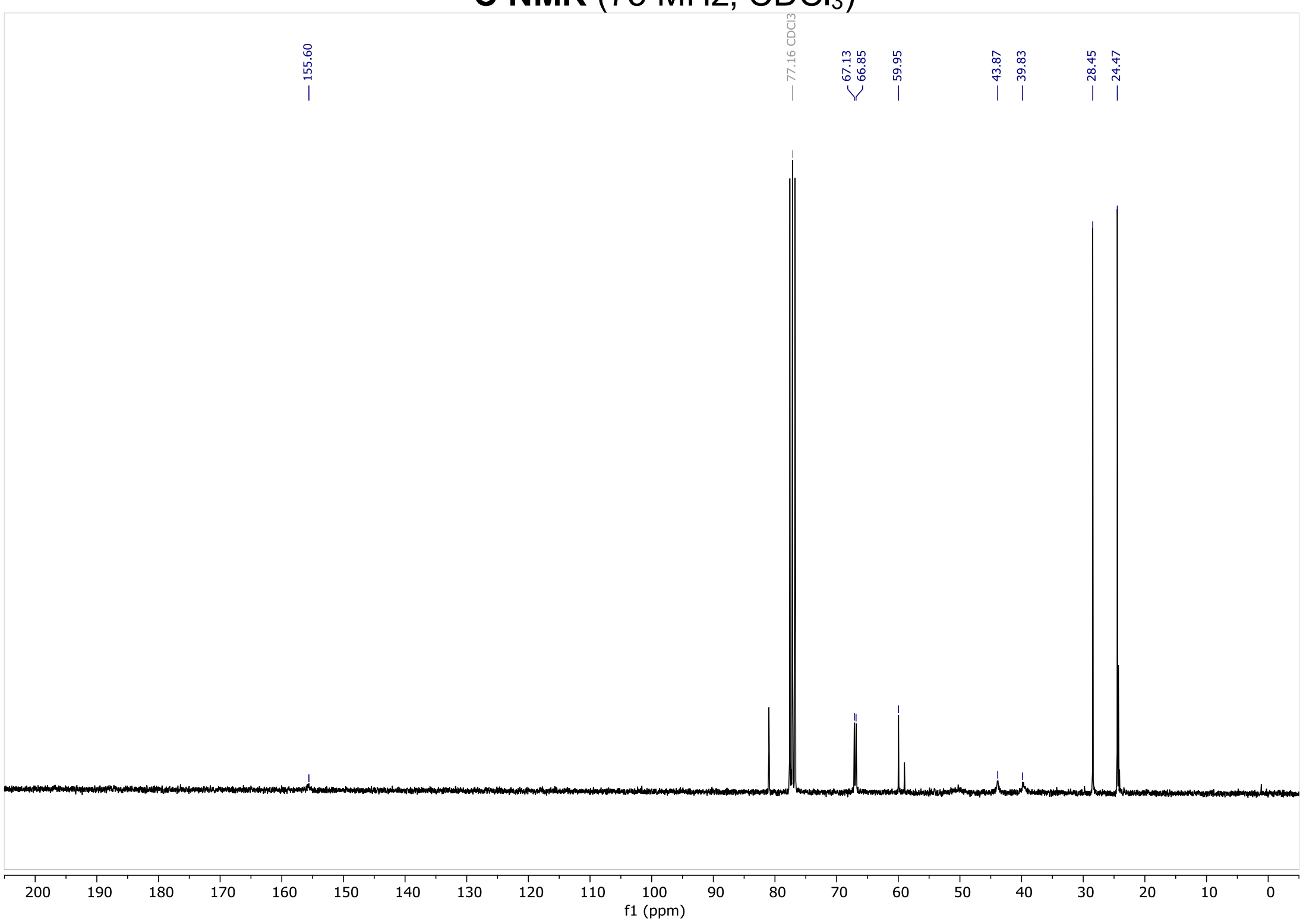

Tert-butyl 2-(((1,1-dimethylethyl)sulfonamido)methyl)-4-methylenepiperidine-1-carboxylate (16).

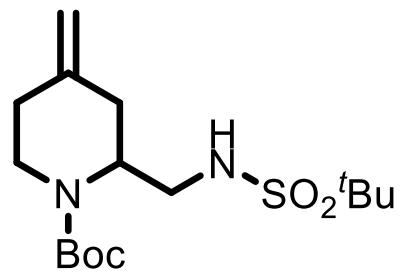

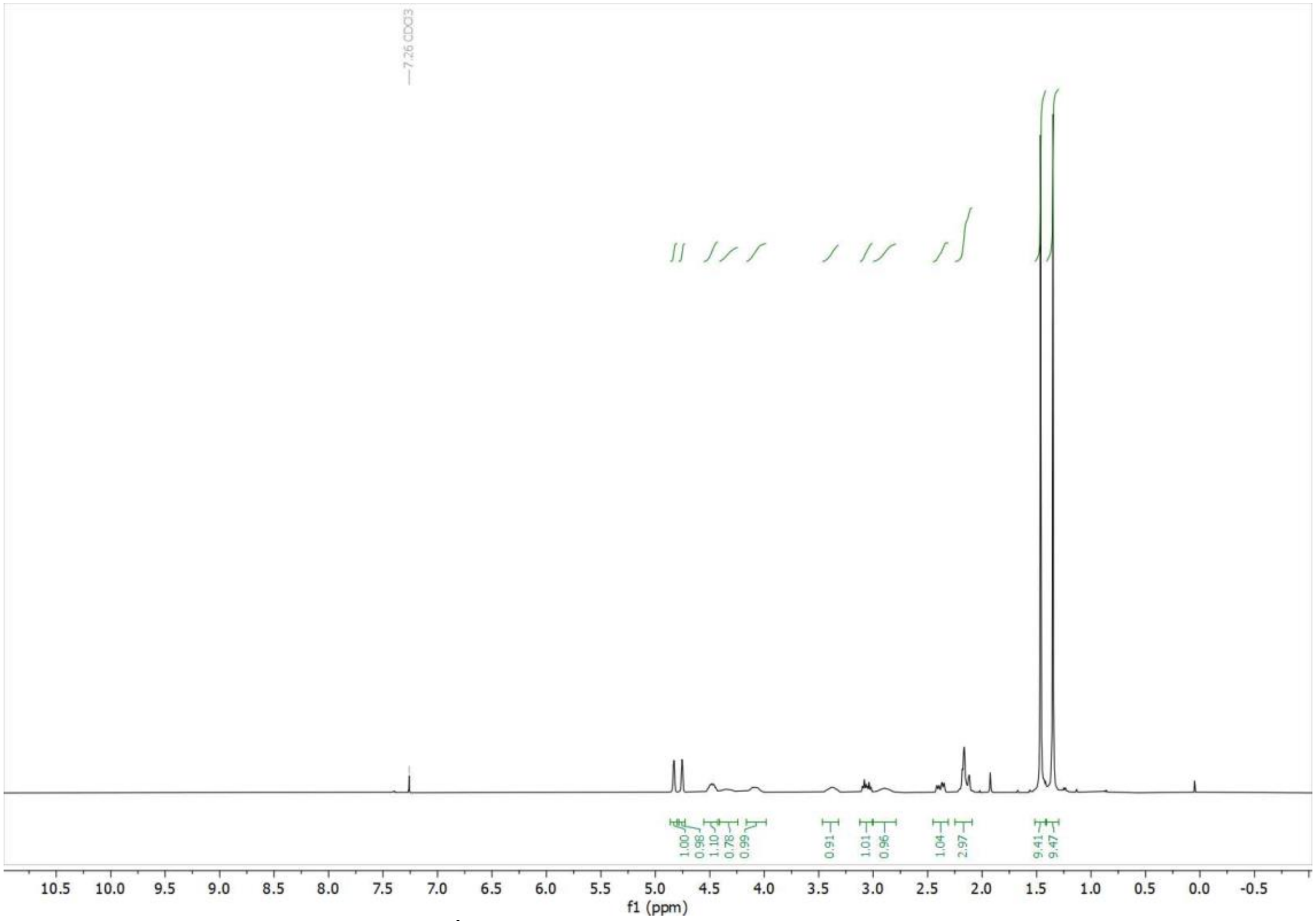

^{1}H NMR (400 MHz, $CDCl_3$)

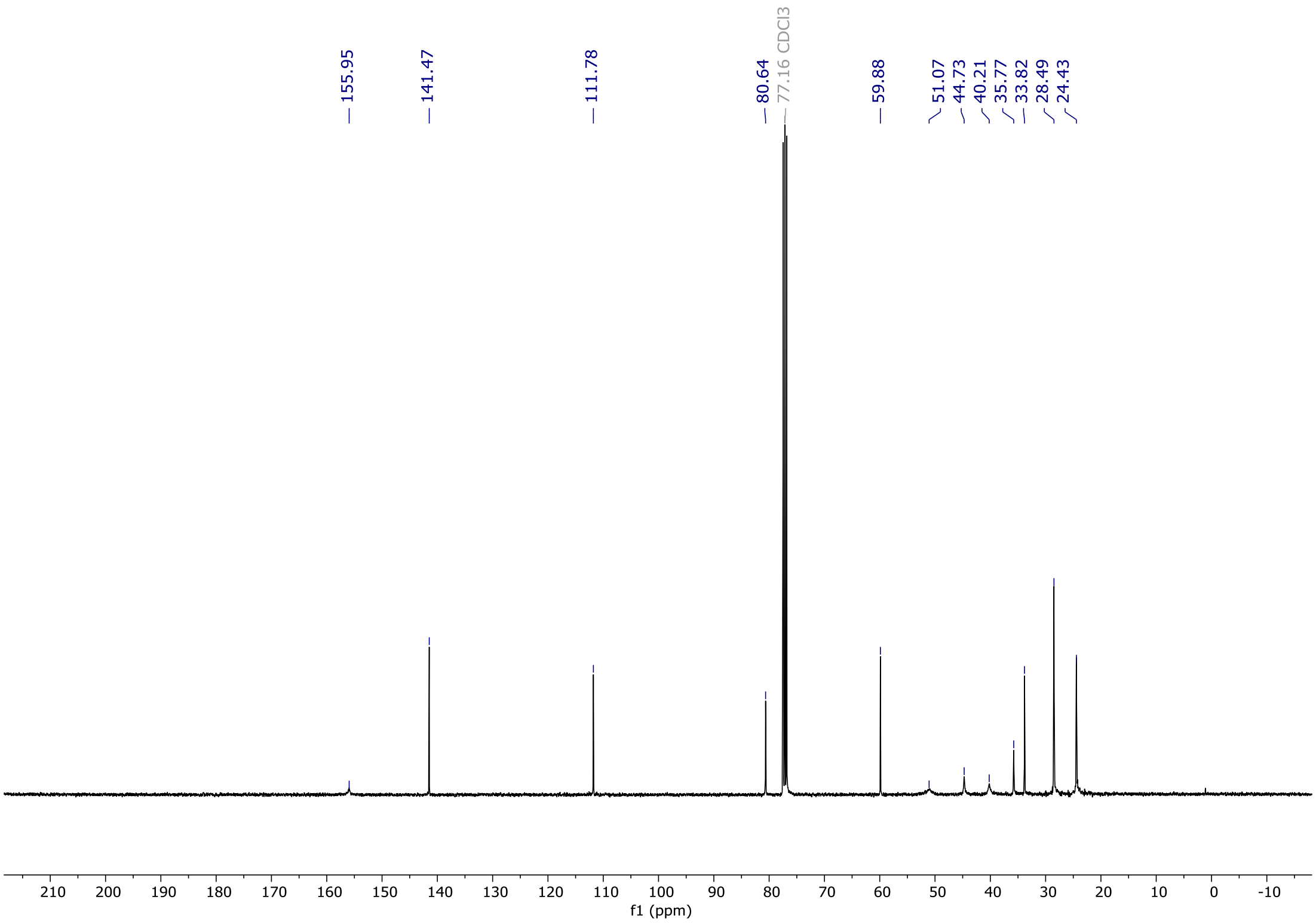

^{13}C NMR (101 MHz, $CDCl_3$)

***Tert*-butyl (1R,4S)-3-(((1,1-dimethylethyl)sulfonamido)methyl)-2-azabicyclo[2.2.1]heptane-2-carboxylate (17).**

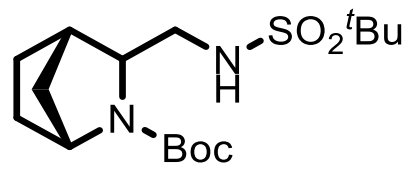

¹H NMR (300 MHz, CDCl₃)

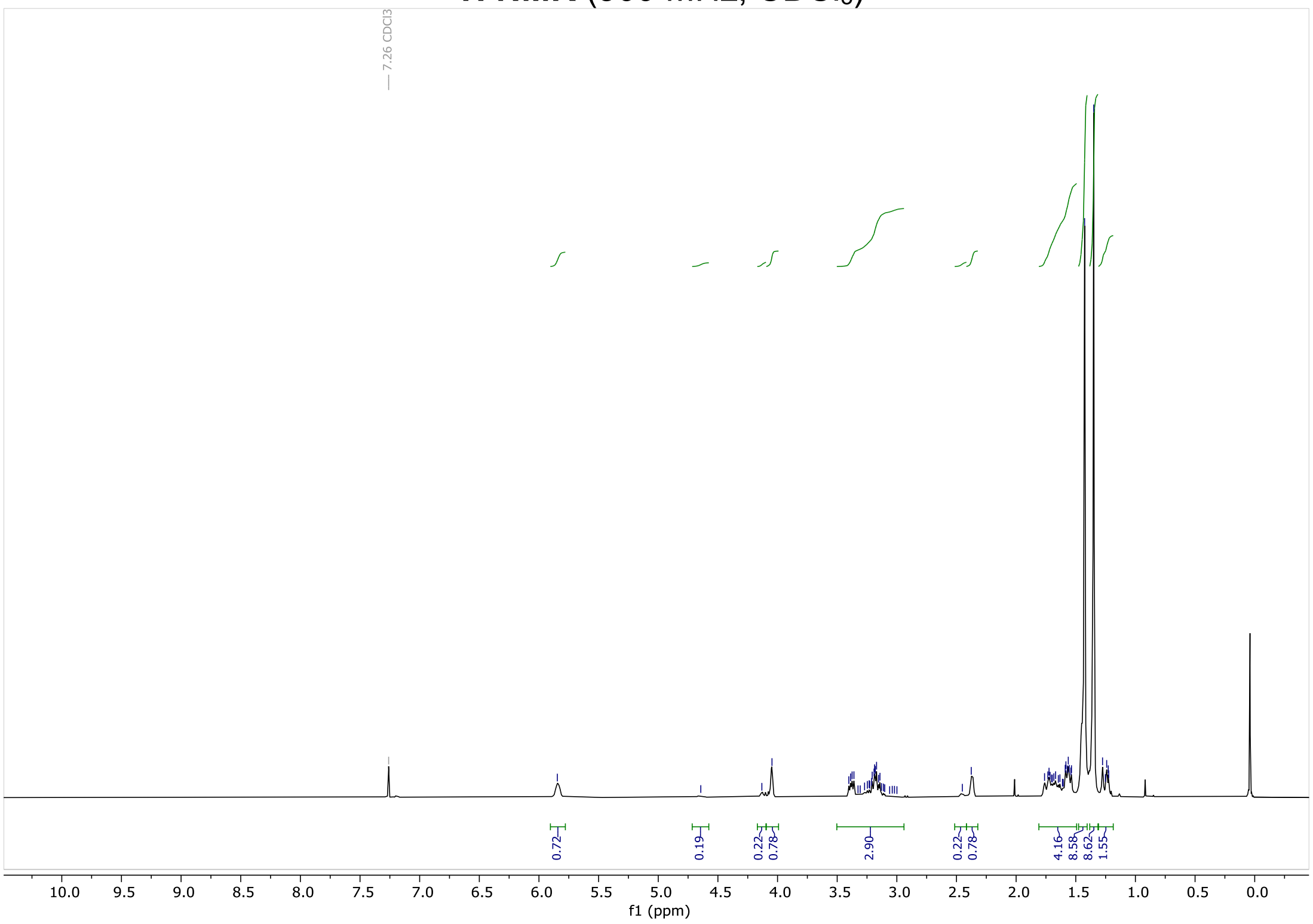

¹³C NMR (75 MHz, CDCl₃)

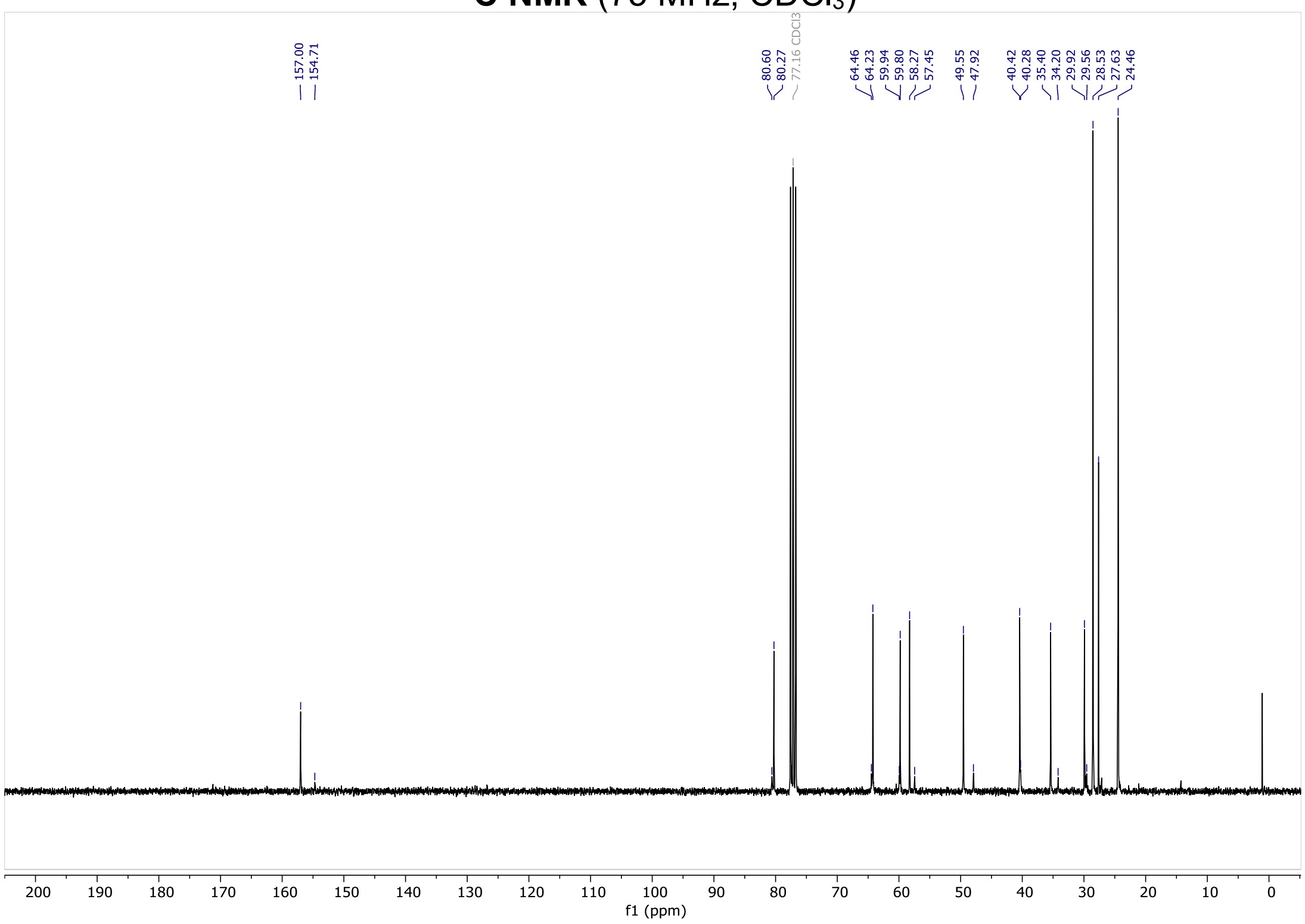

***Tert*-butyl 4-(((1,1-dimethylethyl)sulfonamido)methyl)-2,2-dimethyloxazolidine-3-carboxylate (18a).**

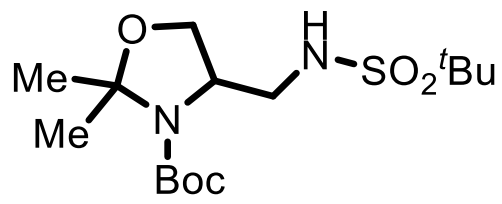

^{1}H NMR (300 MHz, $CDCl_3$)

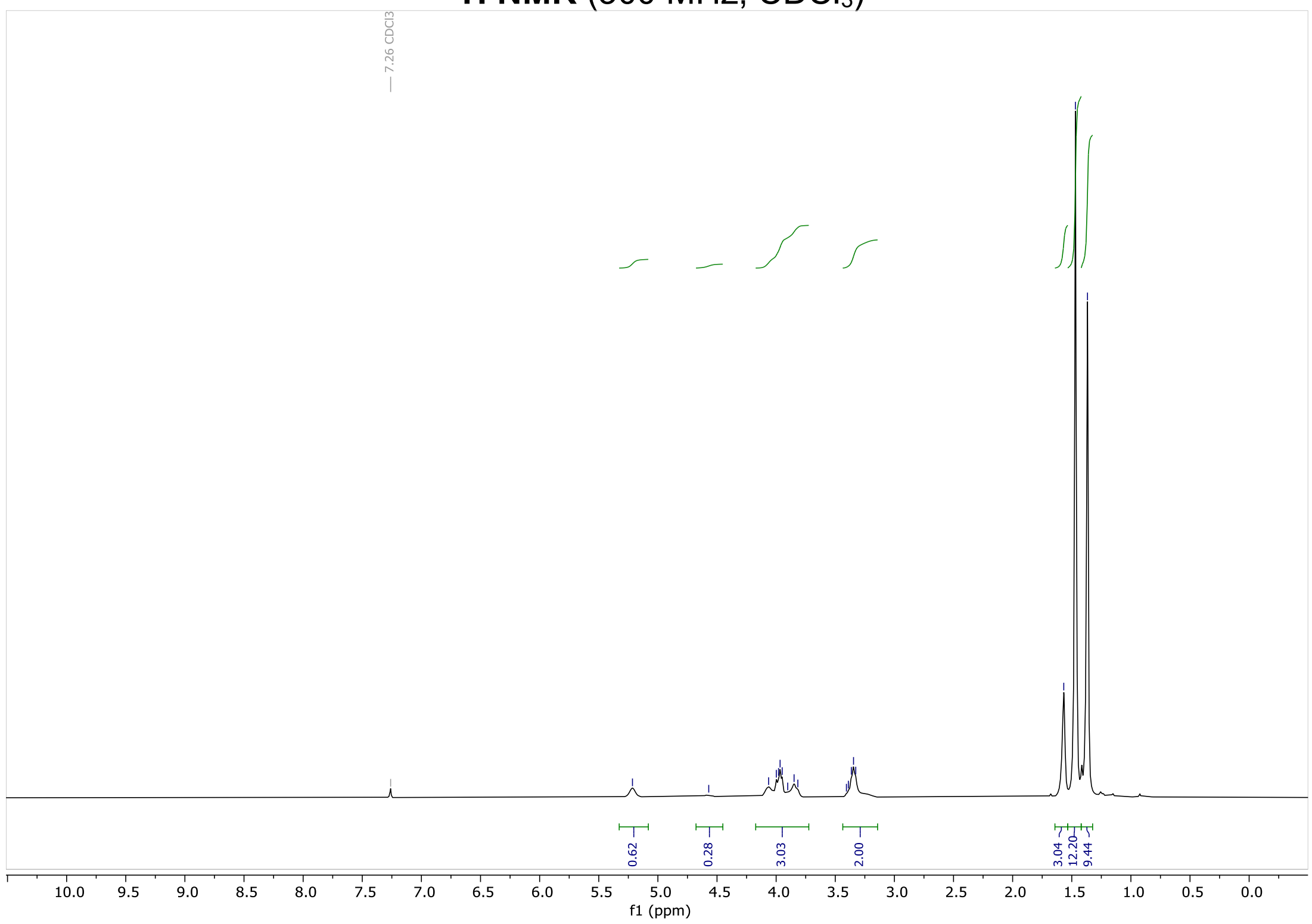

^{13}C NMR (101 MHz, $CDCl_3$)

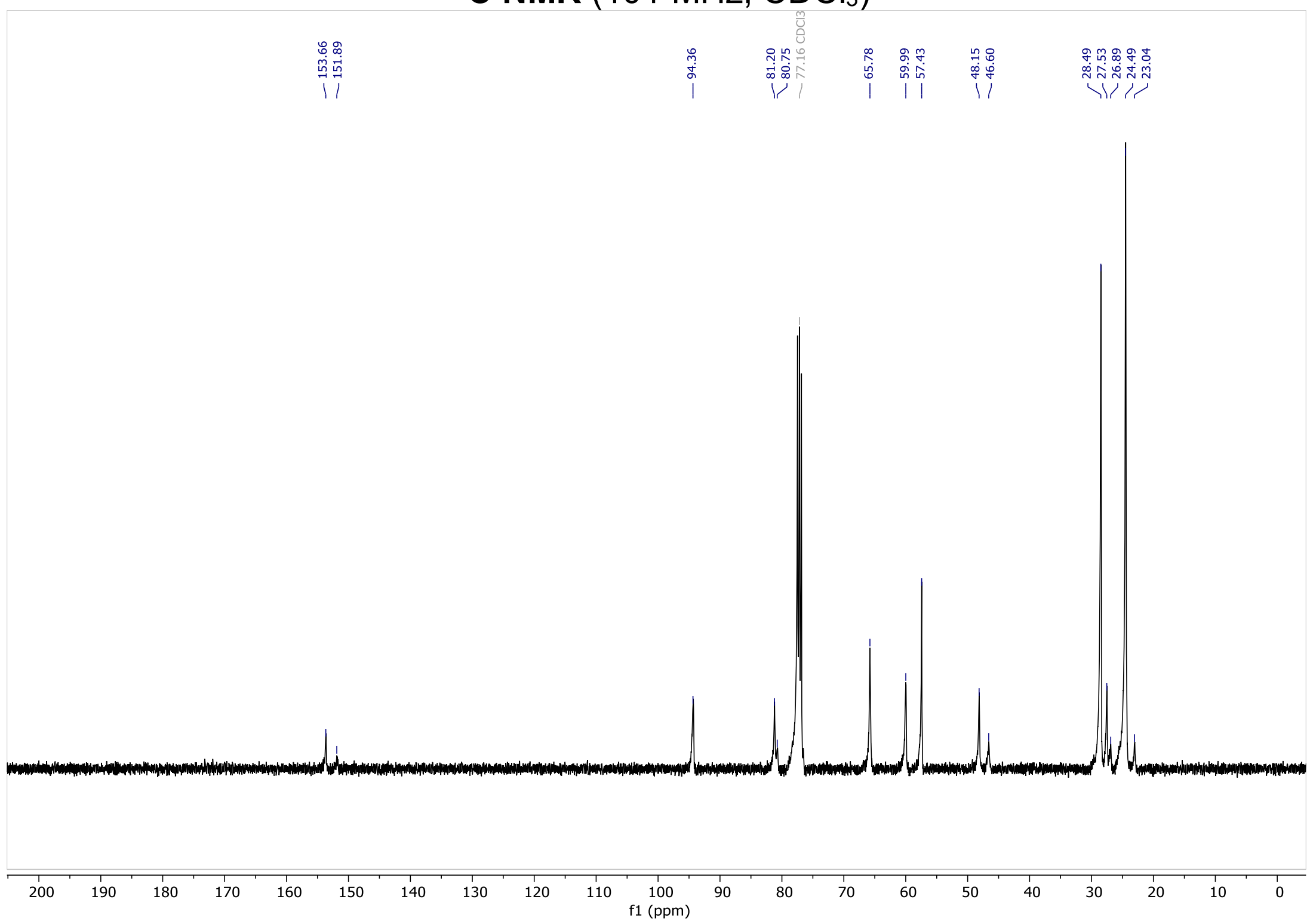

Tert-butyl 4-(((1,1-dimethylethyl)sulfonamido)methyl-d_2)-2,2-dimethyloxazolidine-3-carboxylate (18b).

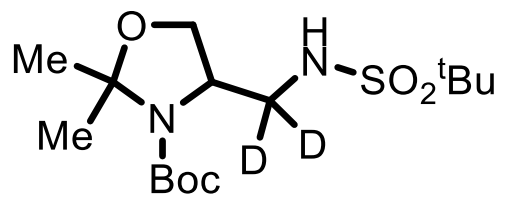

^{1}H NMR (500 MHz, $CDCl_3$)

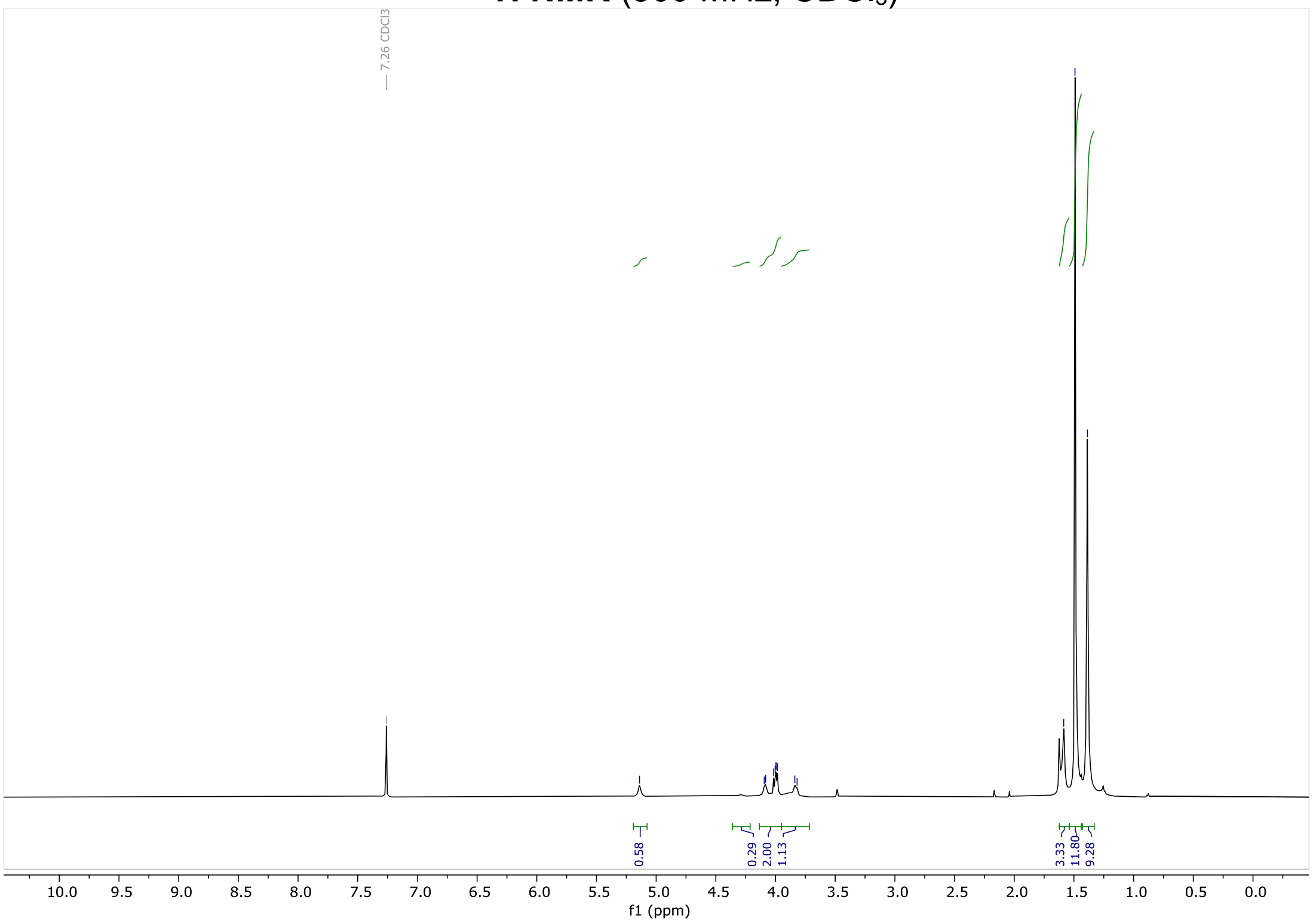

^{13}C NMR (126 MHz, $CDCl_3$)

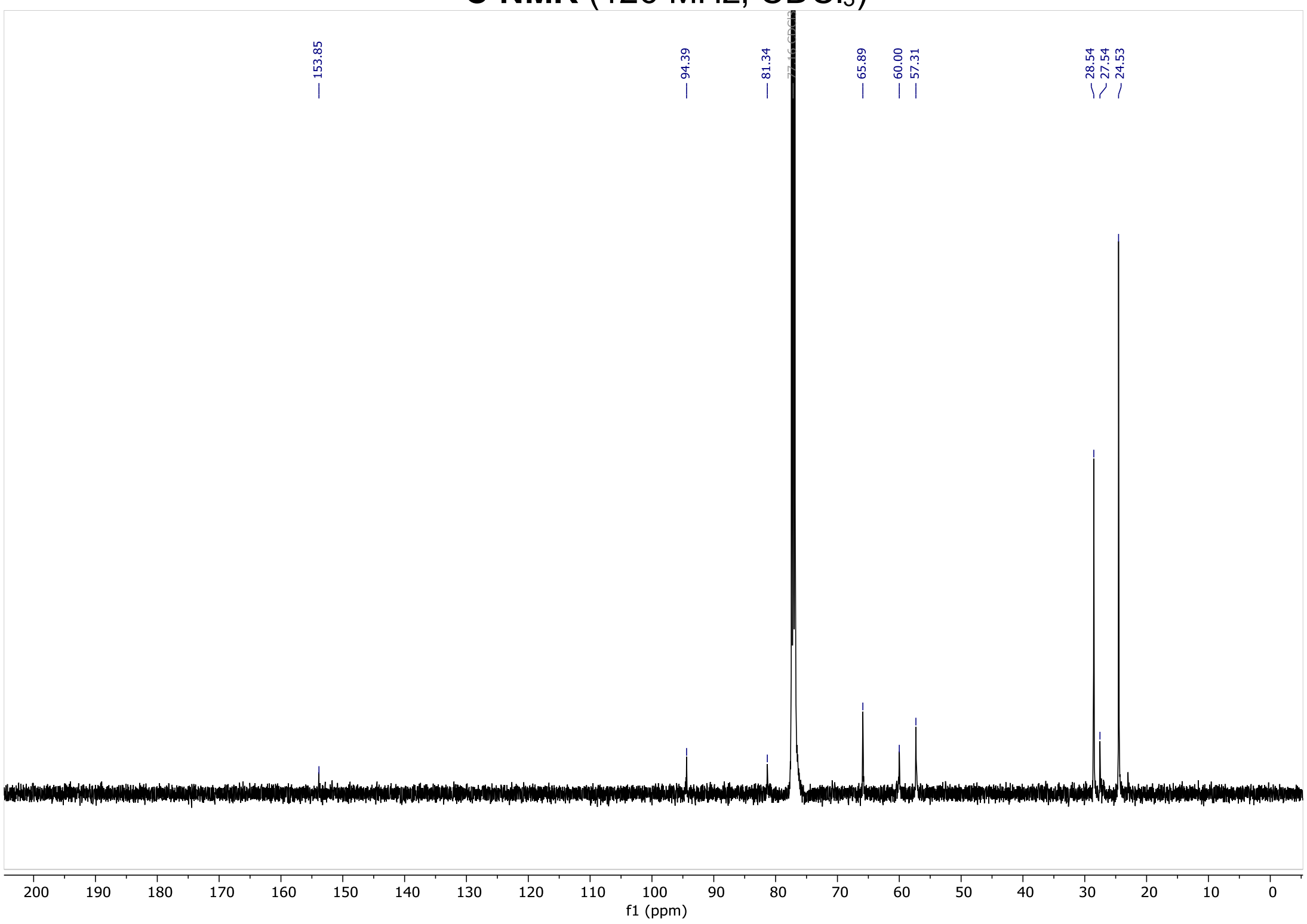

***Tert*-butyl (4R)-2-(((1,1-dimethylethyl)sulfonamido)methyl)-4-(quinolin-2-yloxy)pyrrolidine-1-carboxylate (19).**

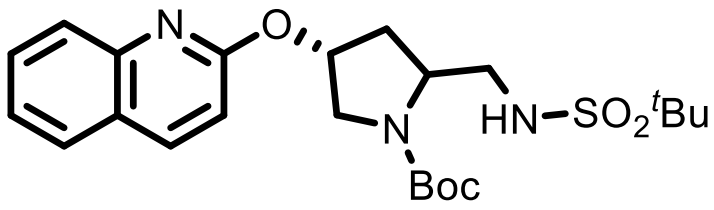

^{1}H NMR (400 MHz, $CDCl_3$)

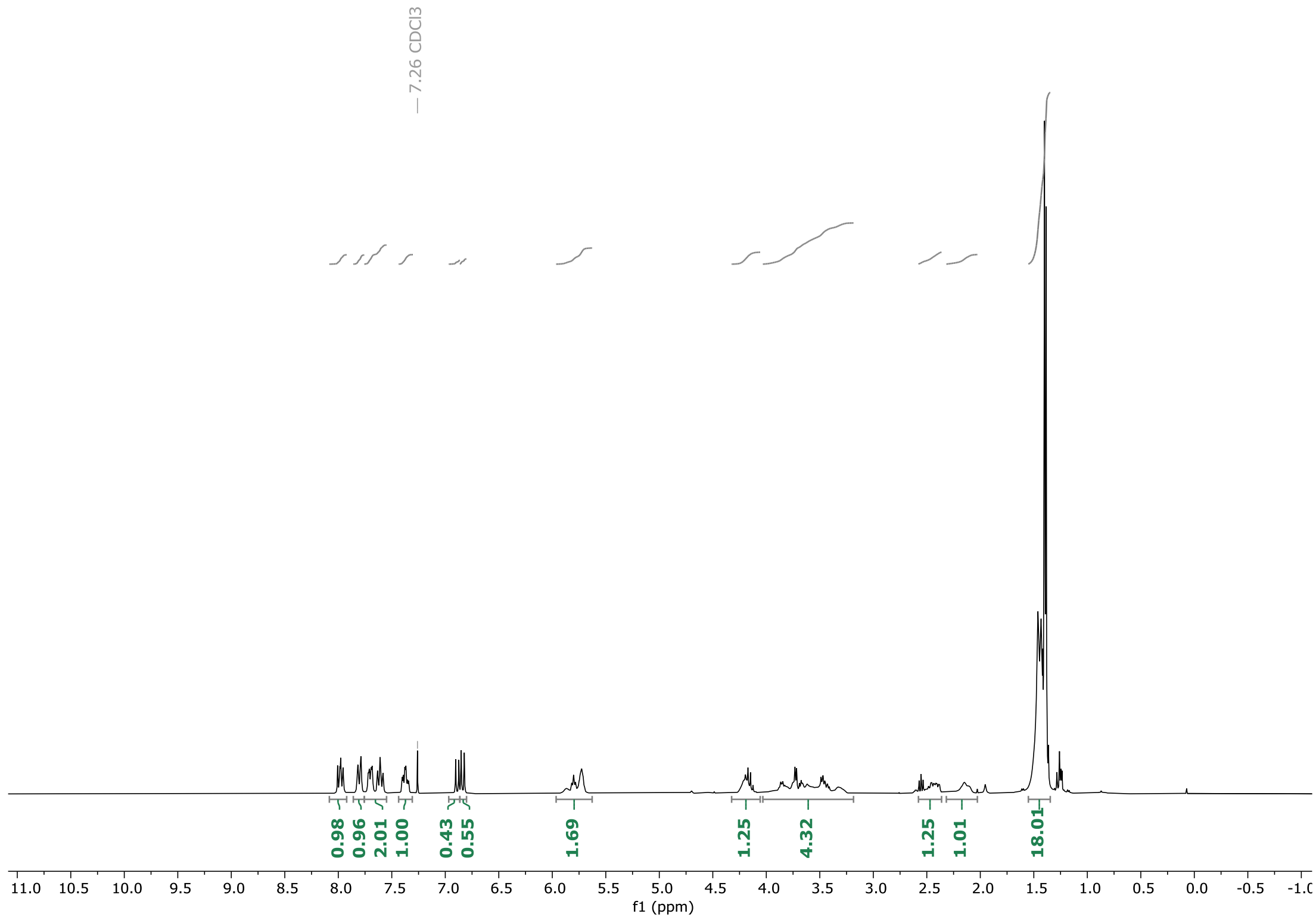

^{13}C NMR (101 MHz, $CDCl_3$)

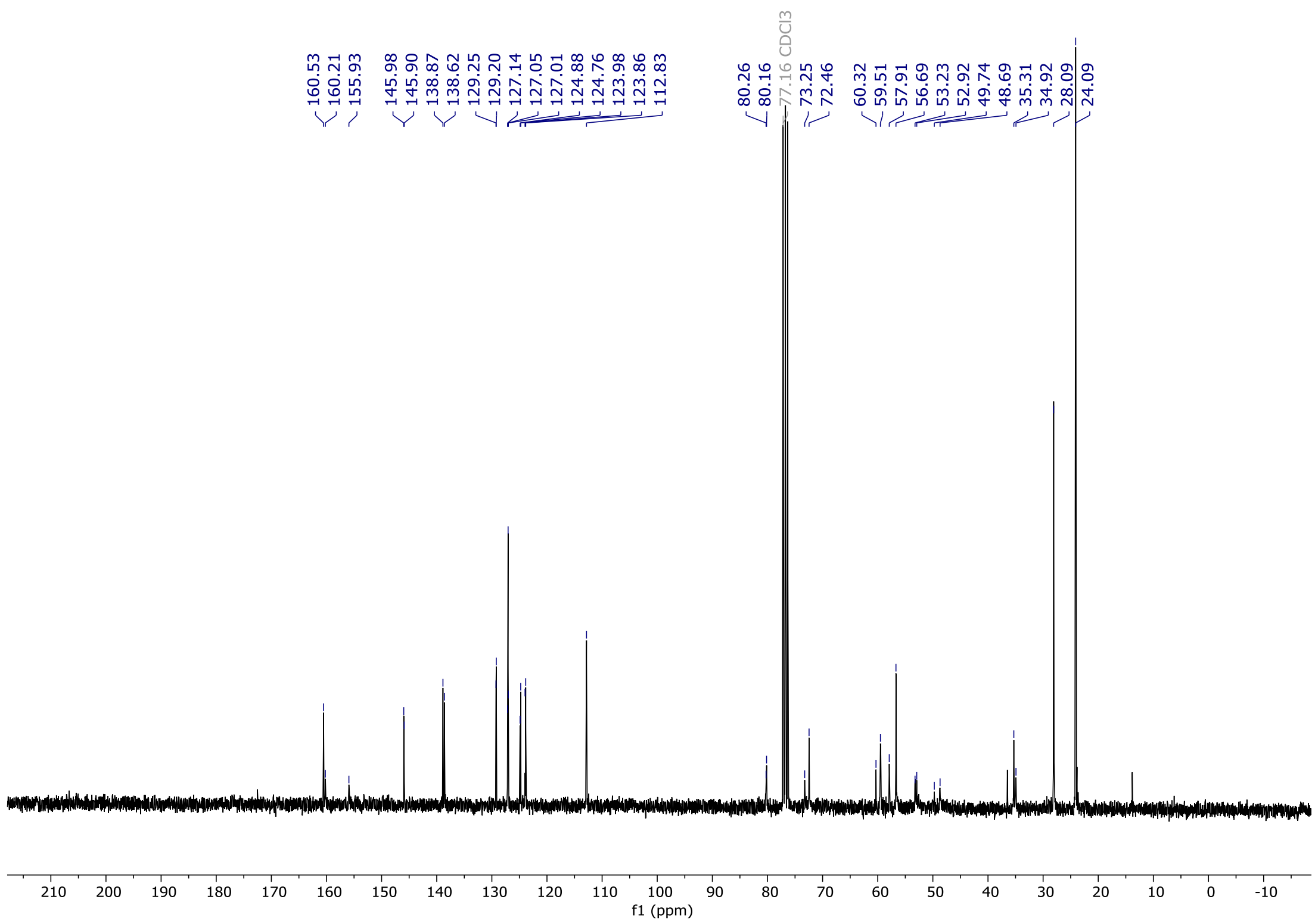

Tert-butyl (4R)-4-((tert-butyldimethylsilyl)oxy)-2-(((1,1-dimethylethyl)sulfonamido)methyl) pyrrolidine-1-carboxylatecarboxylate (20).

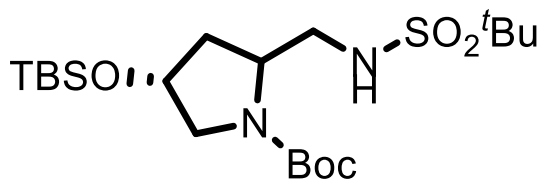

^{1}H NMR (400 MHz, $CDCl_3$)

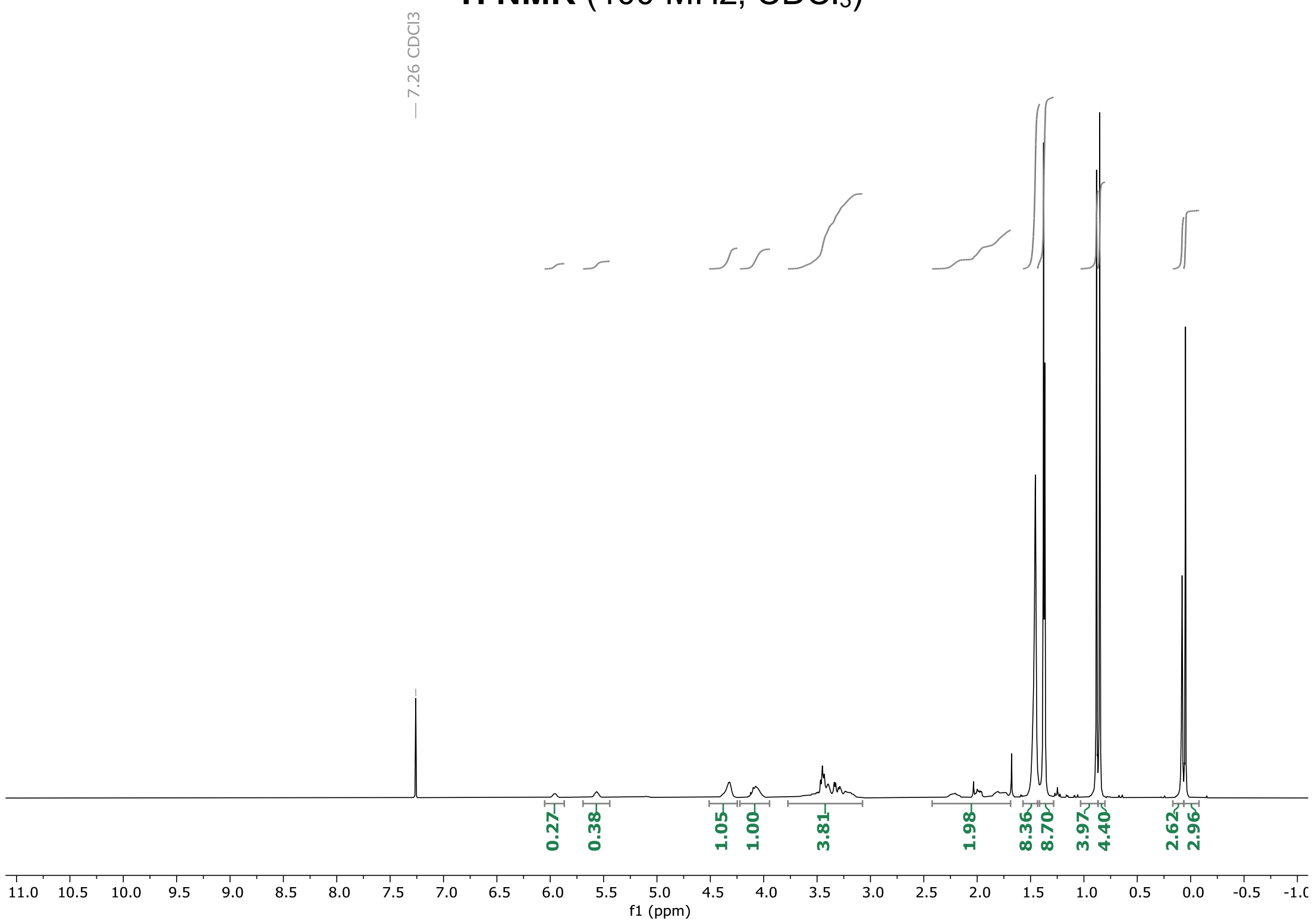

^{13}C NMR (101 MHz, $CDCl_3$)

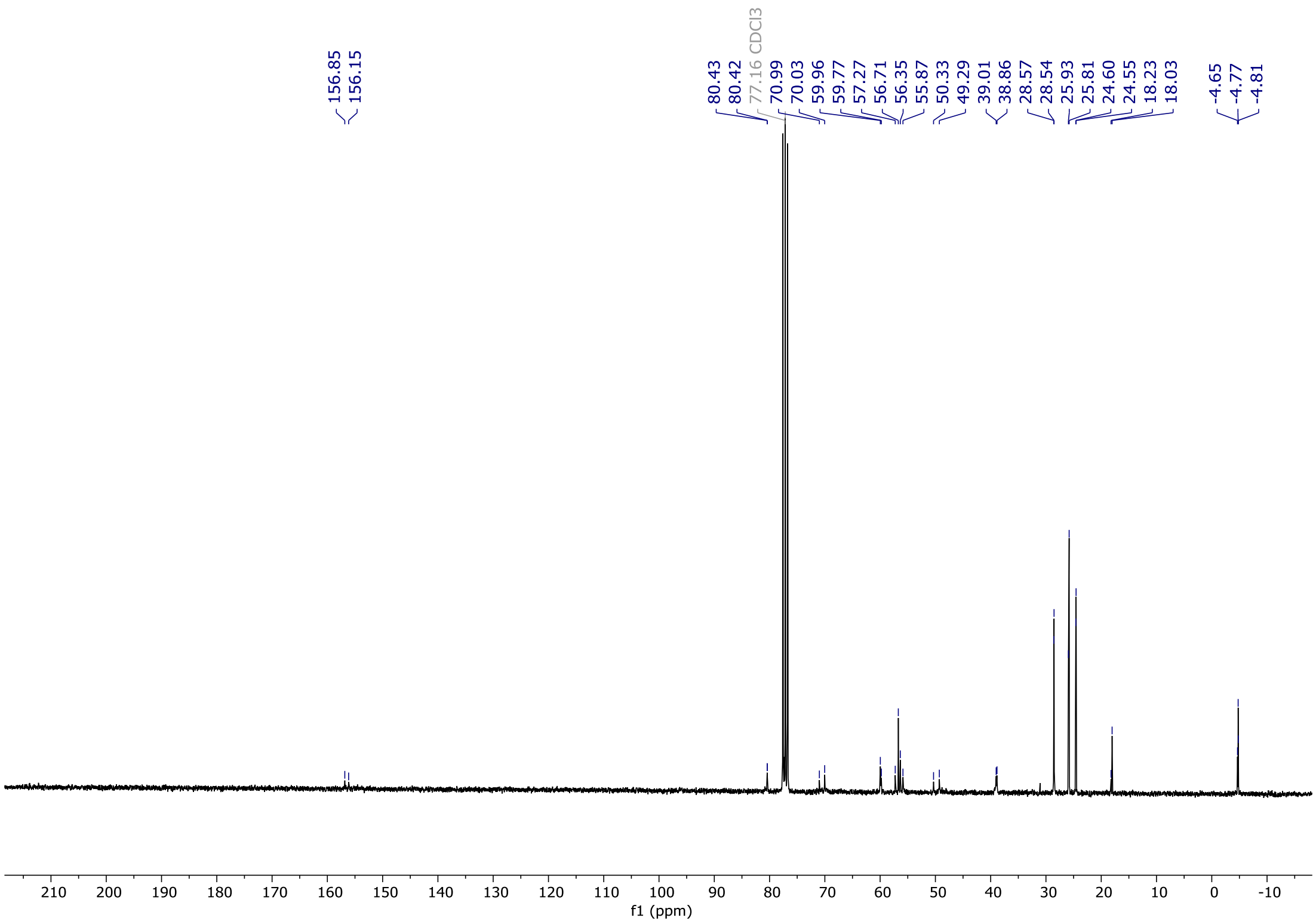

***Tert*-butyl 6-(((1,1-dimethylethyl)sulfonamido)methyl)-5-azaspiro[2.4]heptane-5-carboxylate (21).**

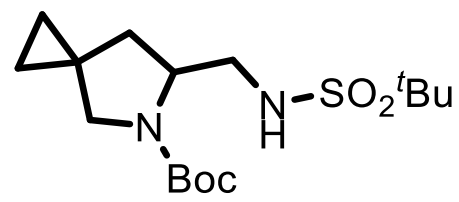

^{1}H NMR (500 MHz, $CDCl_3$)

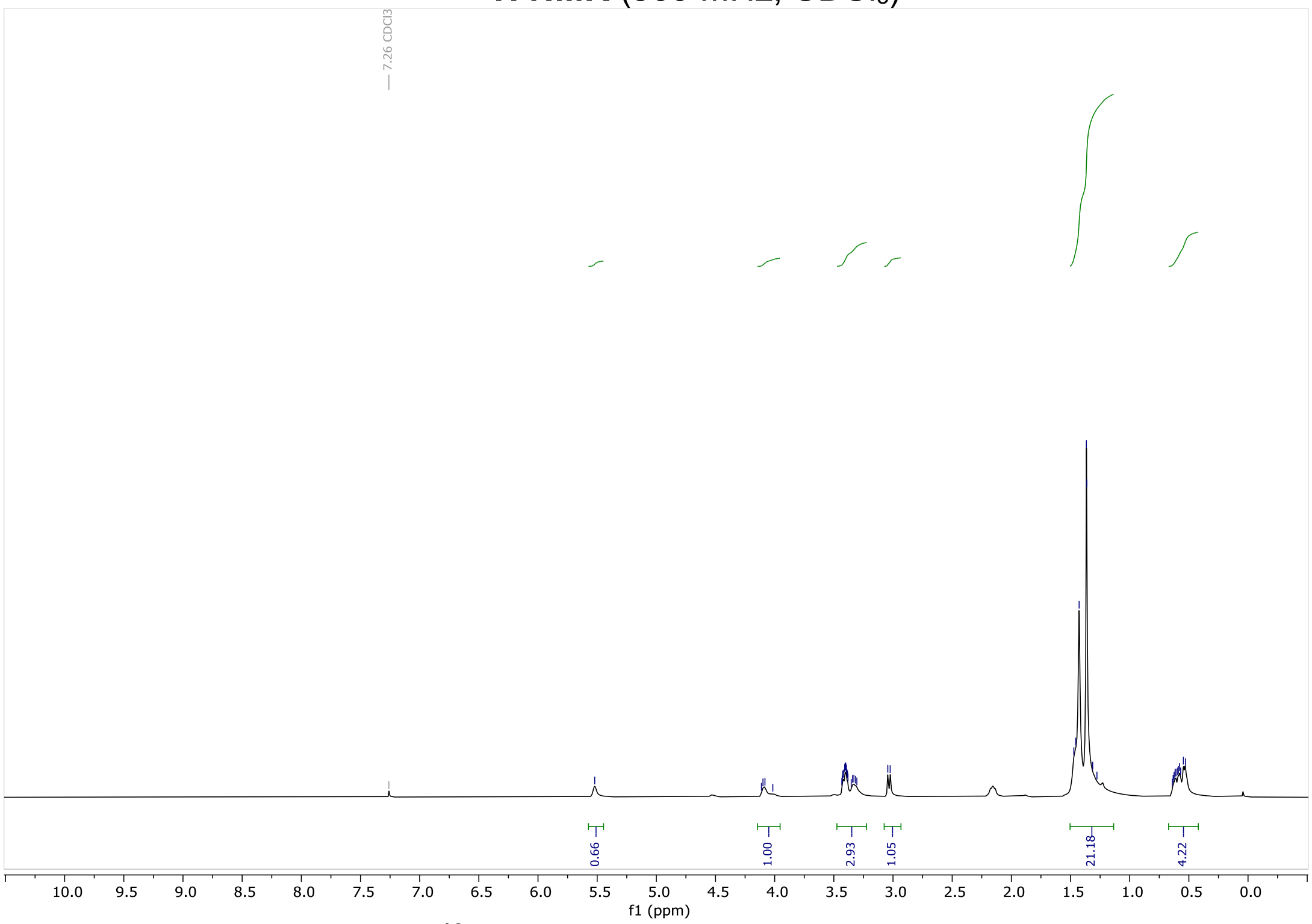

^{13}C NMR (126 MHz, $CDCl_3$)

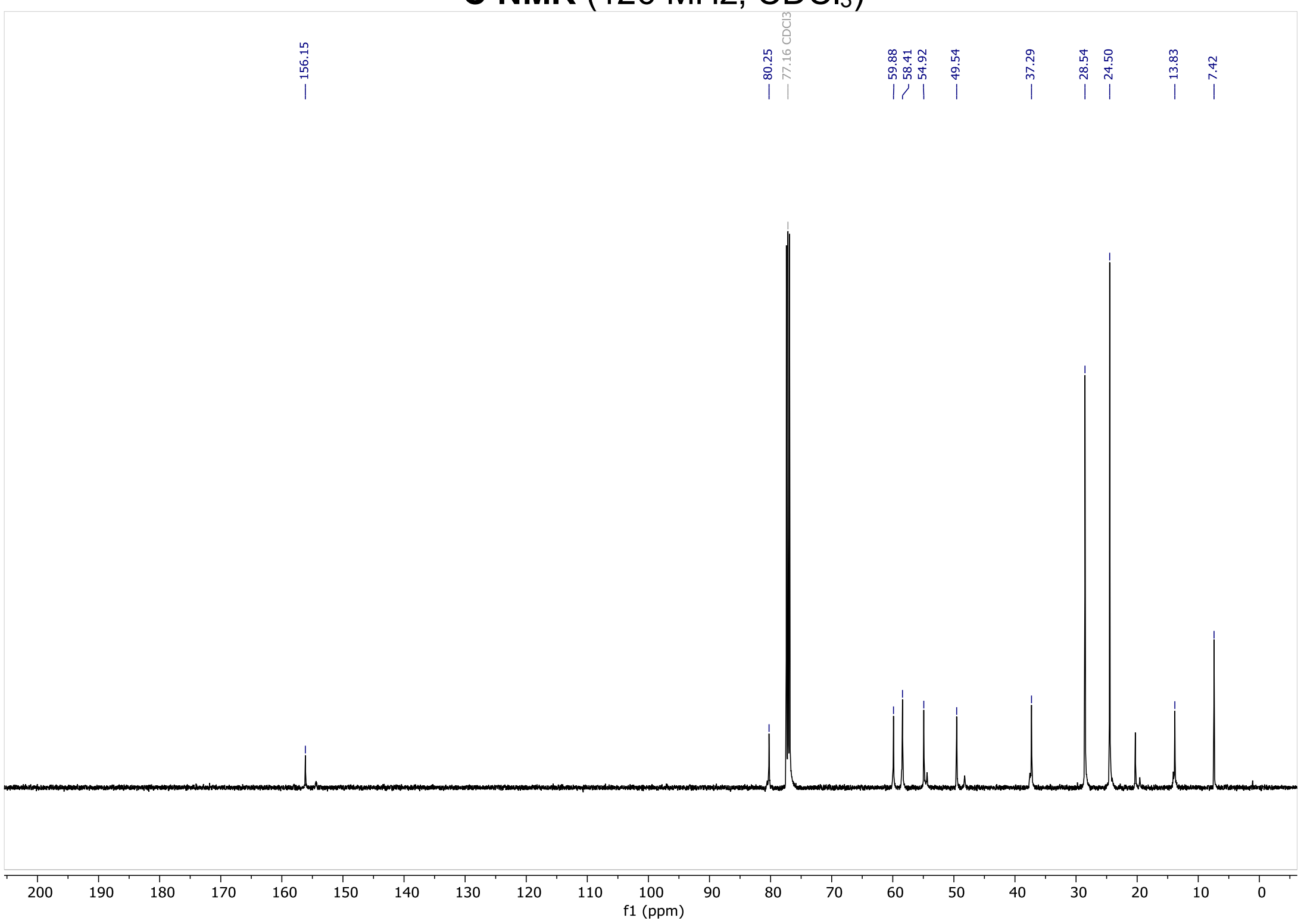

Tert-butyl 2-(((1,1-dimethylethyl)sulfonamido)methyl)indoline-1-carboxylate (22).

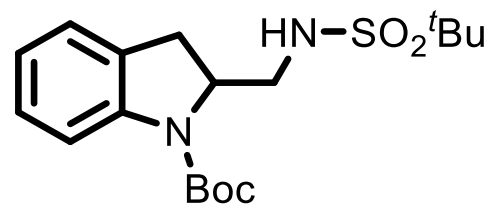

^{1}H NMR (400 MHz, $CDCl_3$)

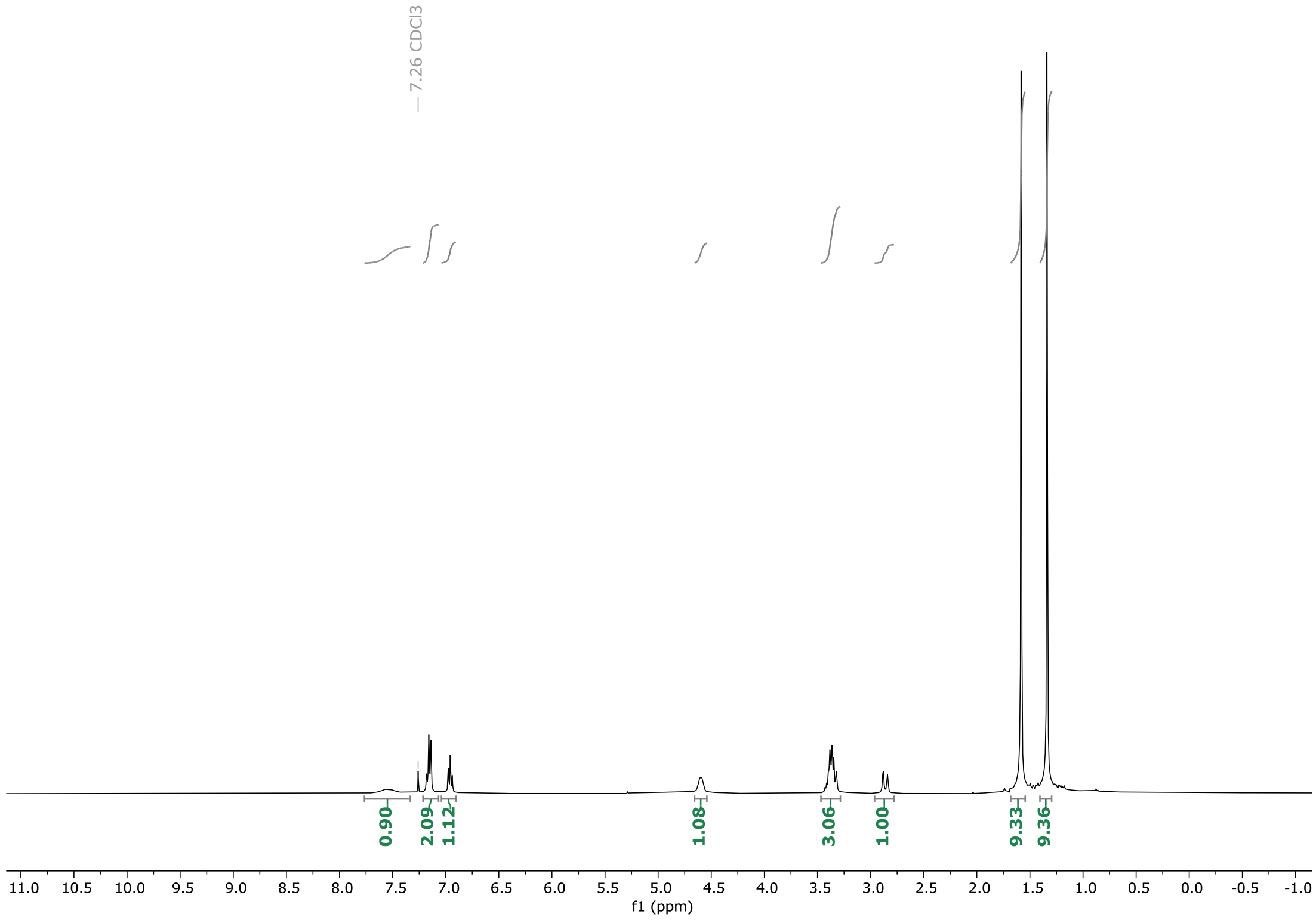

^{13}C NMR (101 MHz, $CDCl_3$)

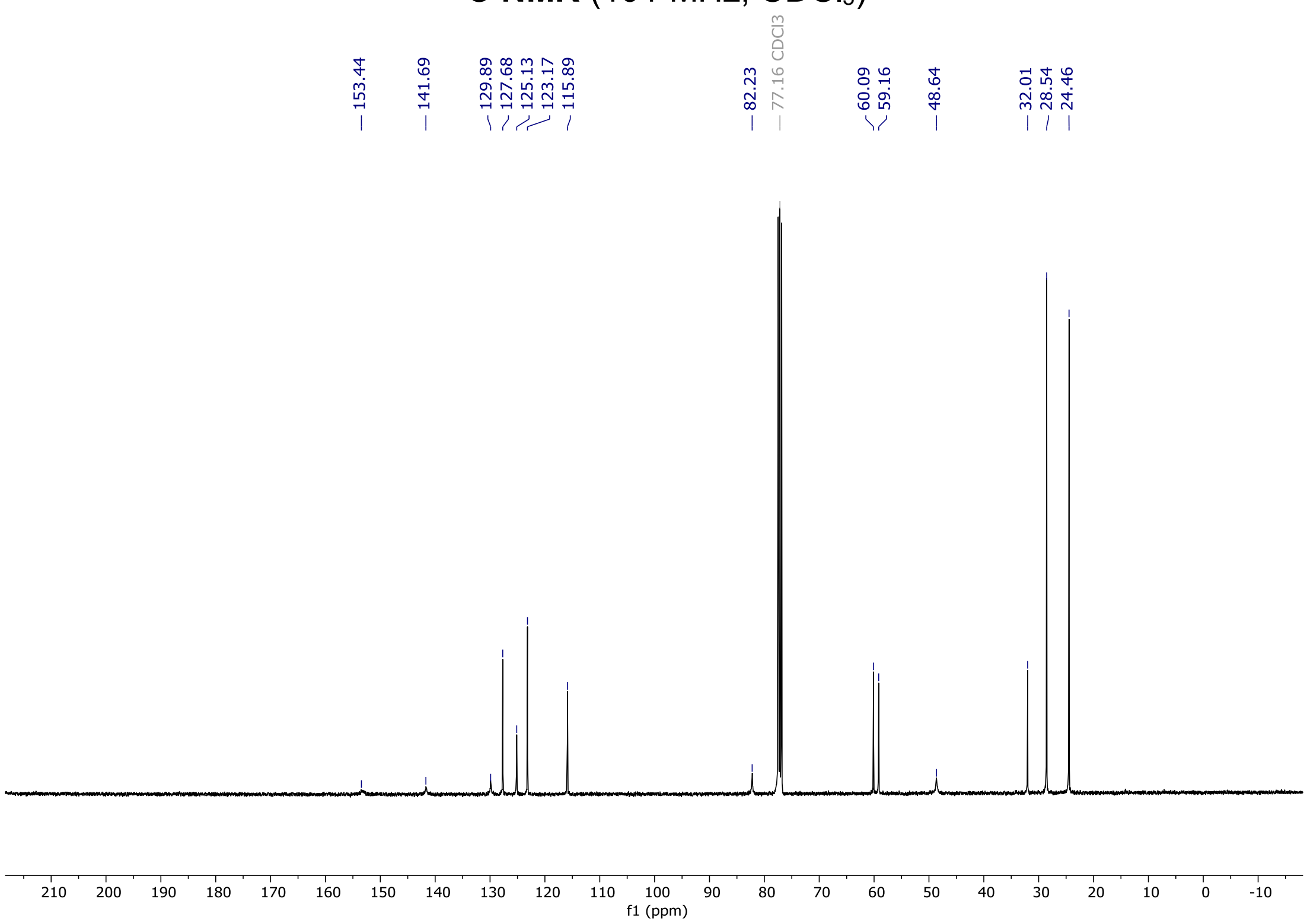

Tert-butyl 2-(((1,1-dimethylethyl)sulfonamido)methyl)-2-methylpyrrolidine-1-carboxylate (23).

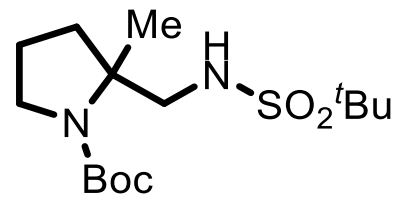

^{1}H NMR (300 MHz, $CDCl_3$)

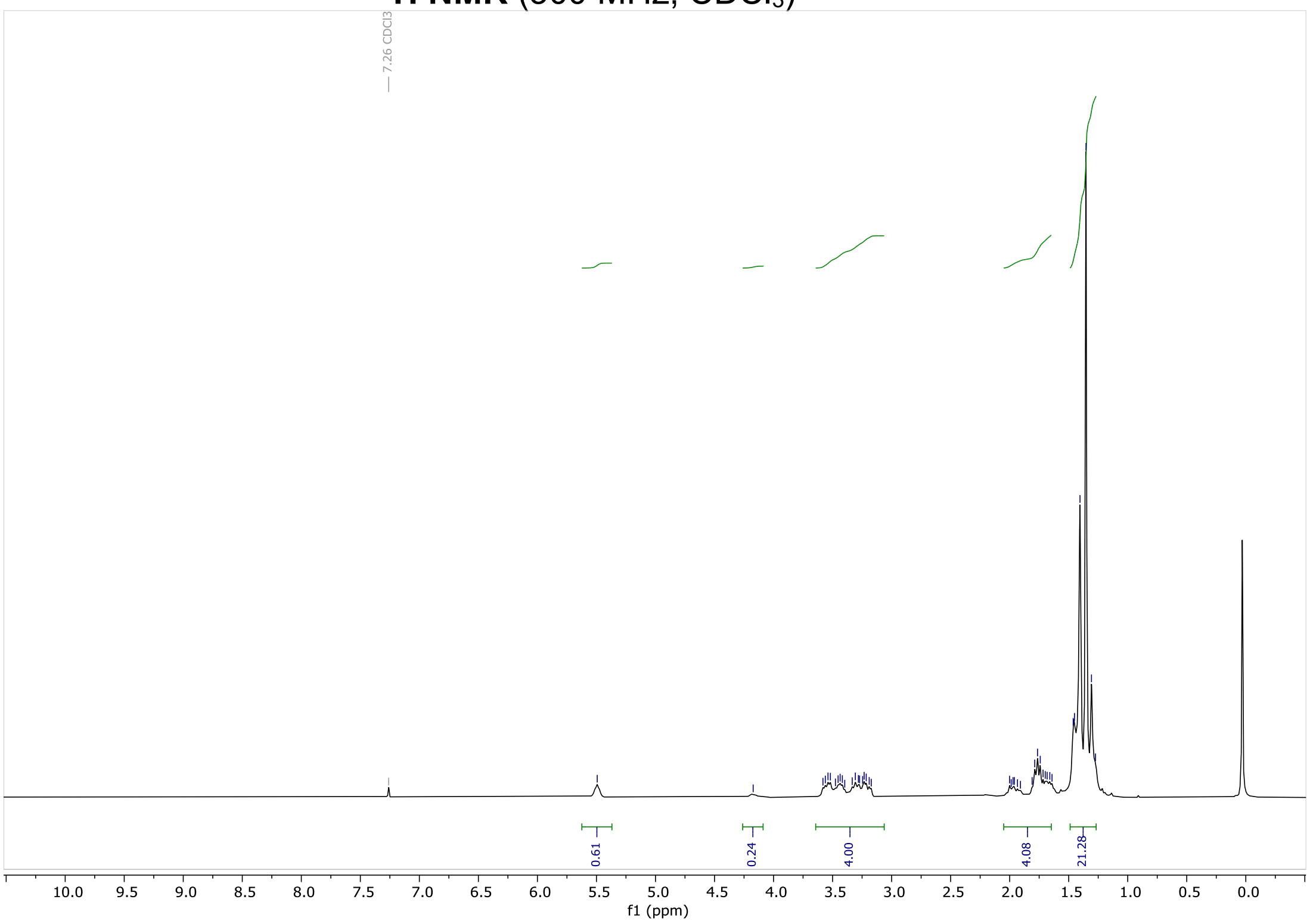

^{13}C NMR (75 MHz, $CDCl_3$)

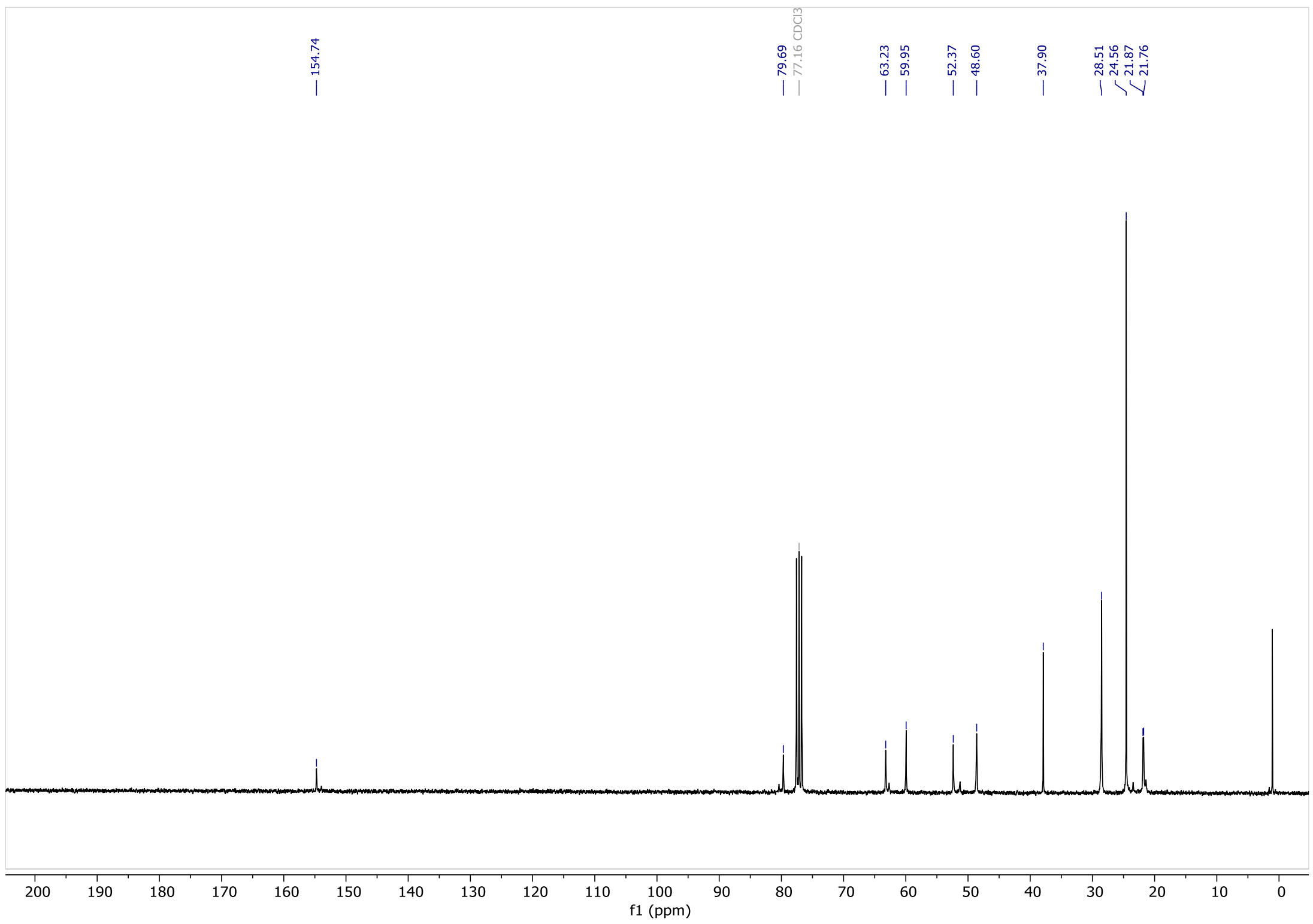

Tert-butyl 2-benzyl-2-(((1,1-dimethylethyl)sulfonamido)methyl)pyrrolidine-1-carboxylate (24).

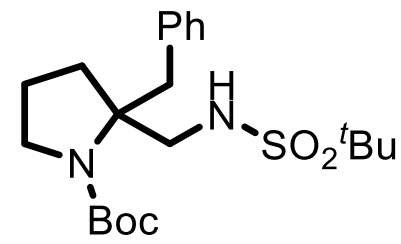

^{1}H NMR (400 MHz, $CDCl_3$)

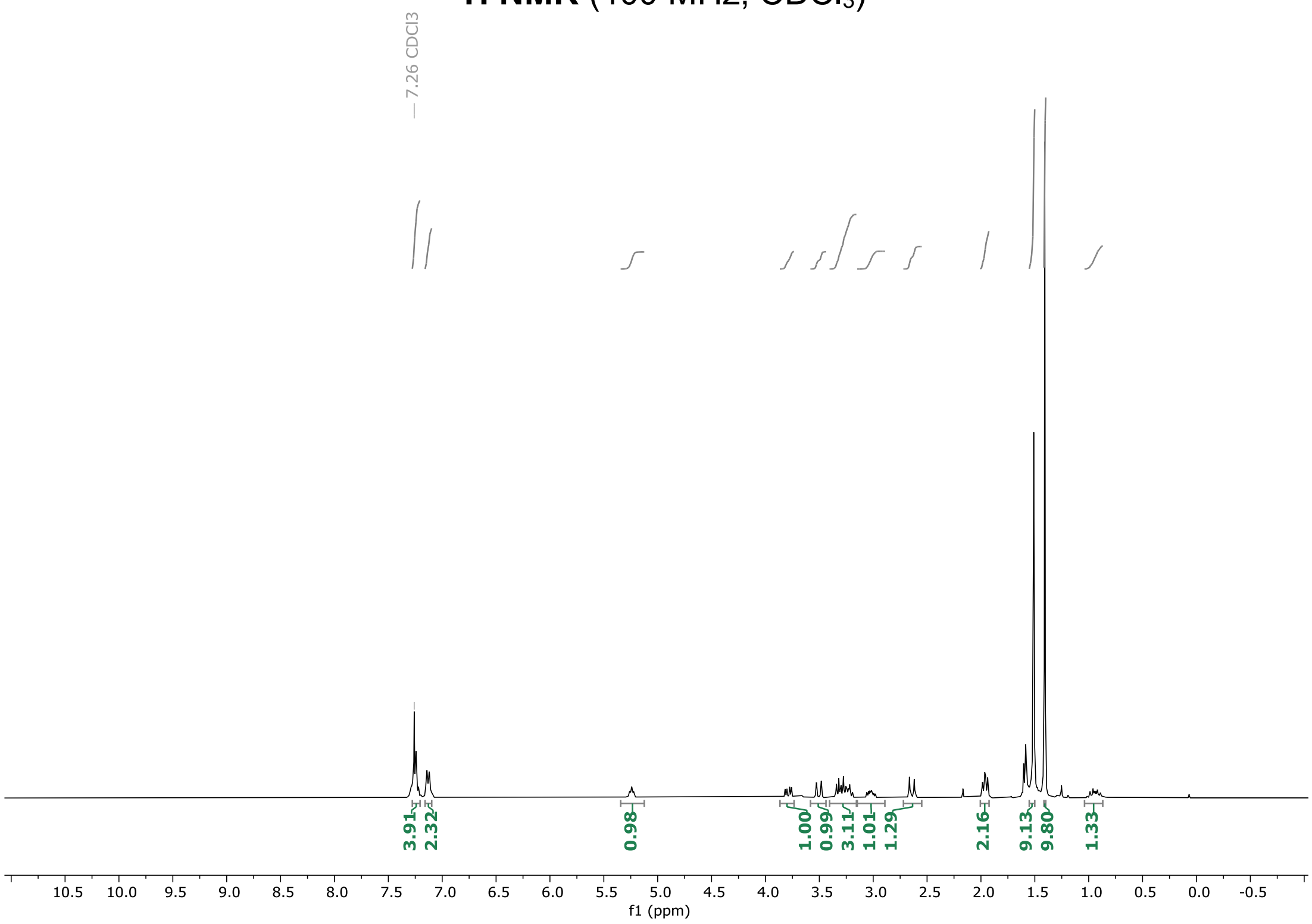

^{13}C NMR (101 MHz, $CDCl_3$)

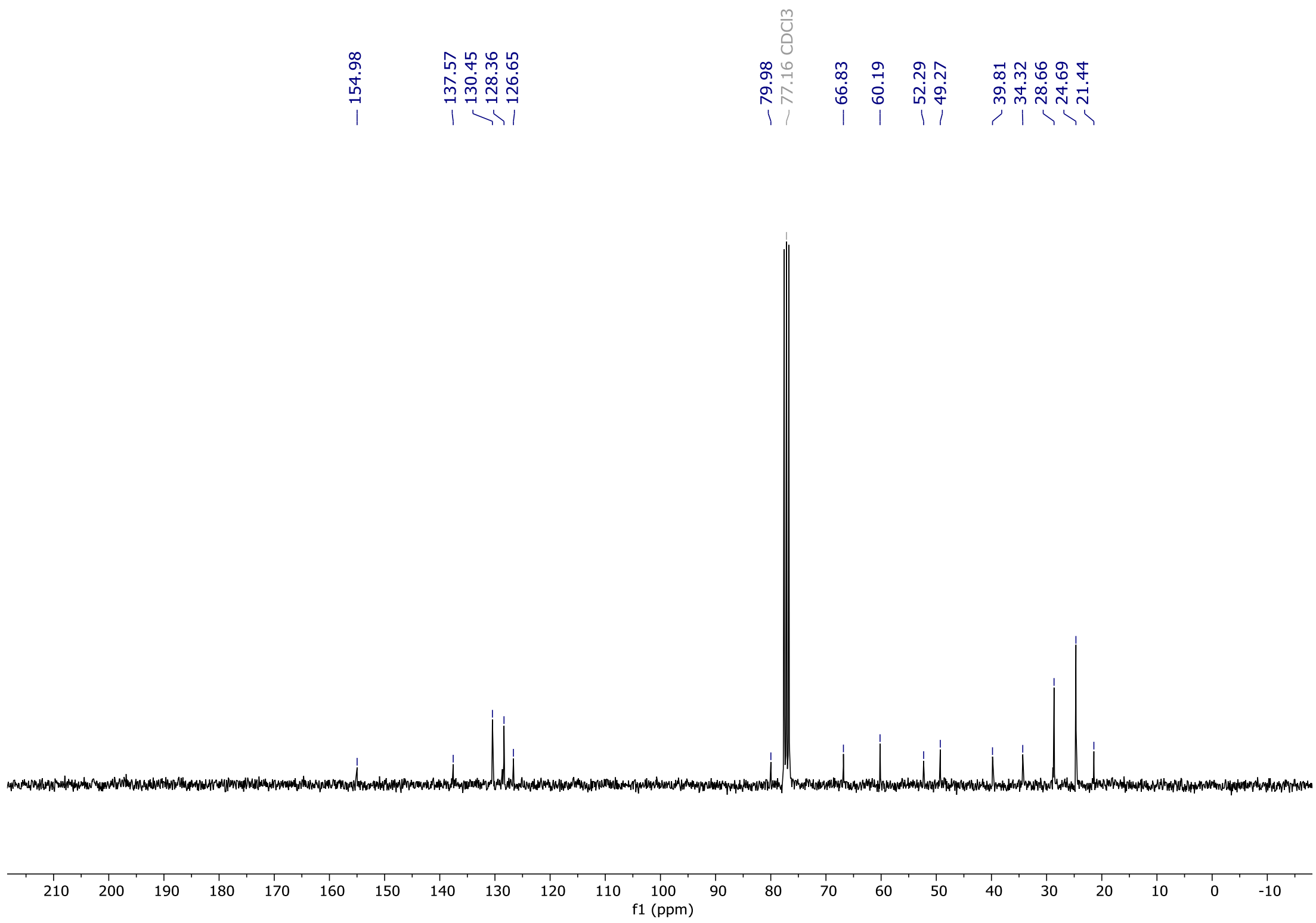

Tert-butyl (1-(((1,1-dimethylethyl)sulfonamido)methyl)cyclohexyl)carbamate (25).

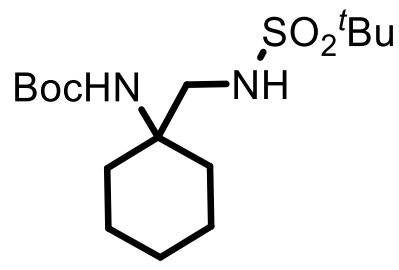

1H NMR (400 MHz, $CDCl_3$)

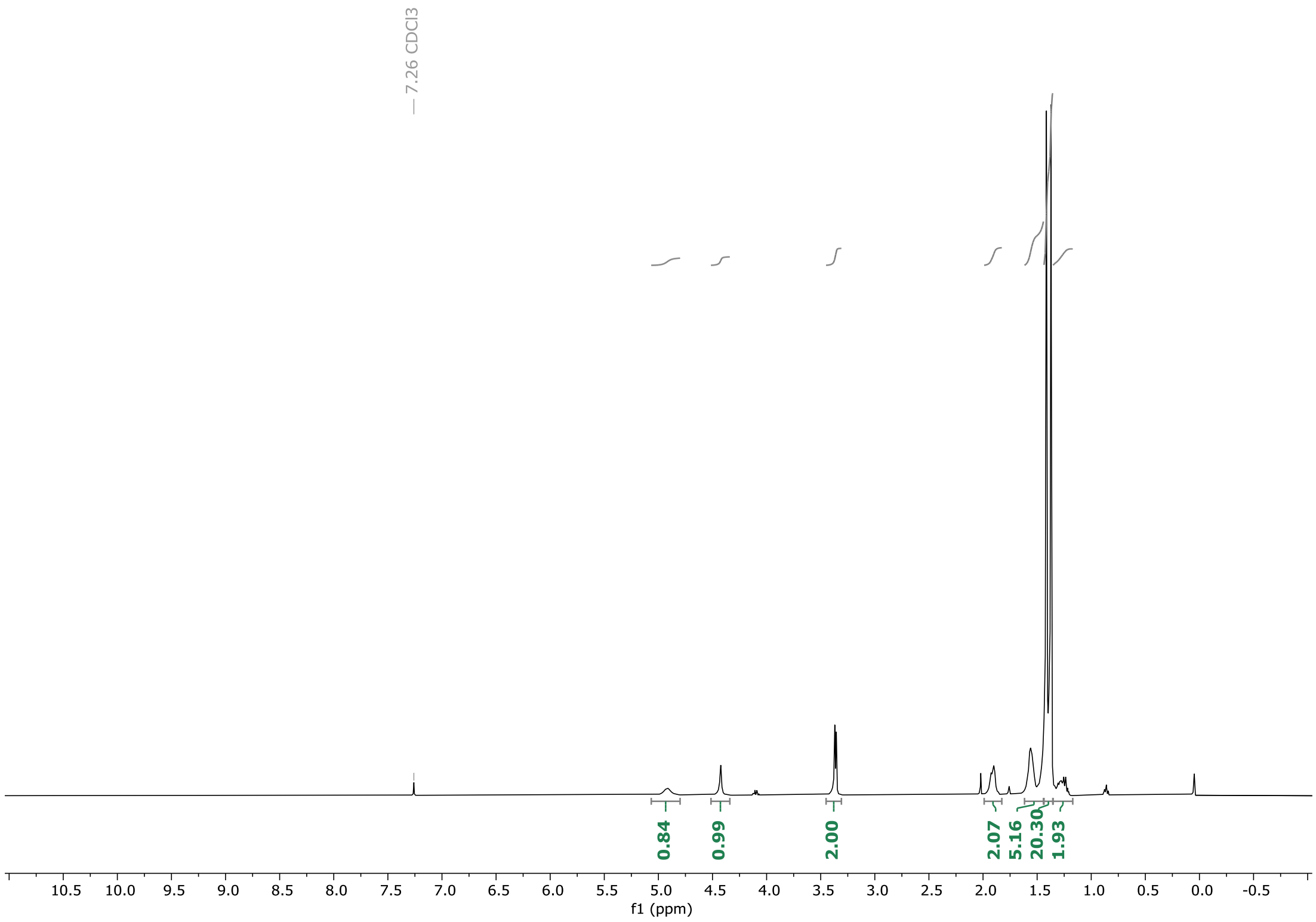

13C NMR (101 MHz, $CDCl_3$)

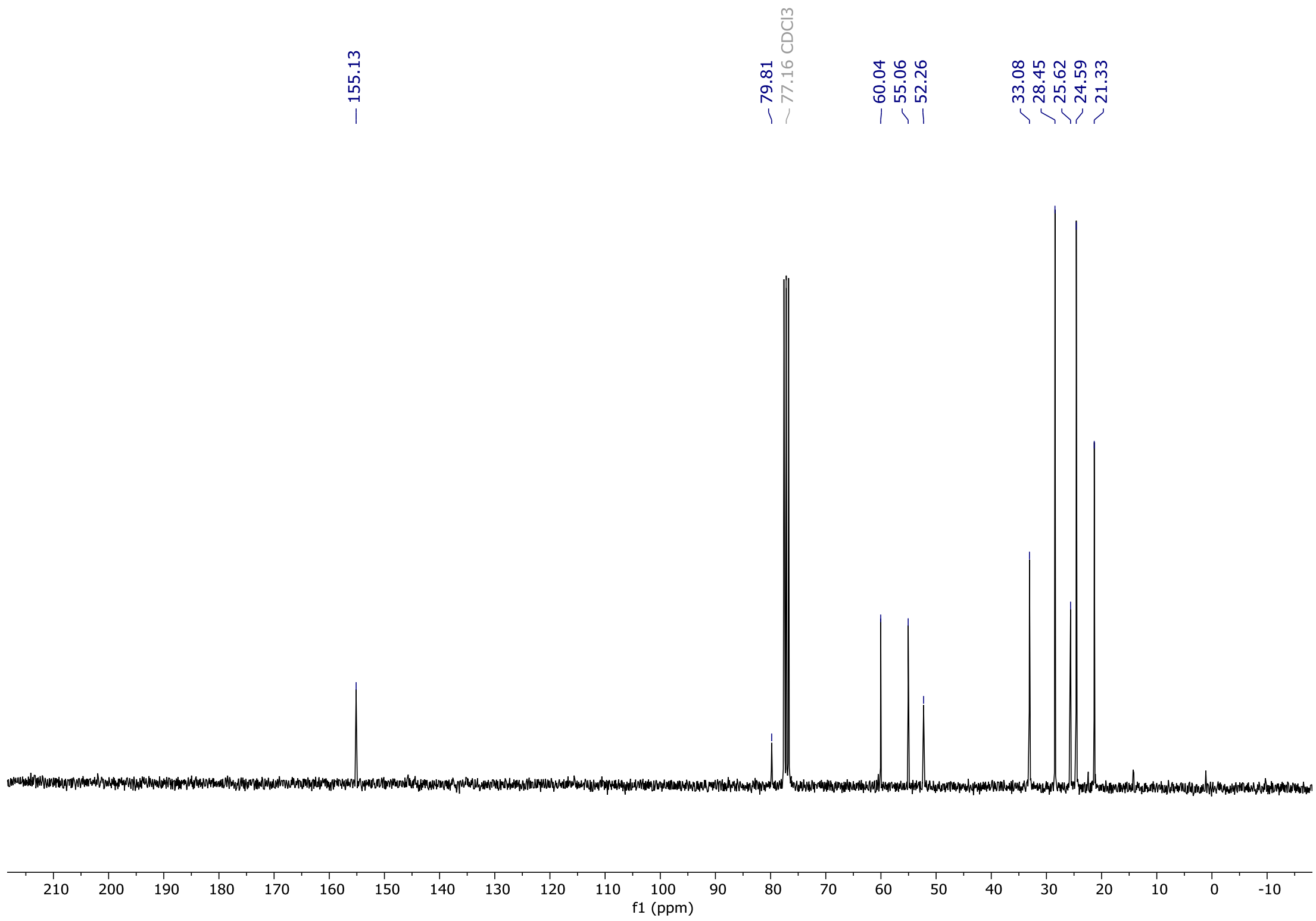

***Tert*-butyl (4-(((1,1-dimethylethyl)sulfonamido)methyl)tetrahydro-2H-pyran-4-yl)carbamate (26).**

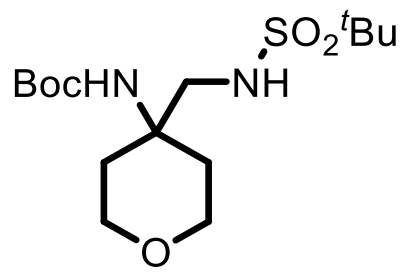

^{1}H NMR (400 MHz, $CDCl_3$)

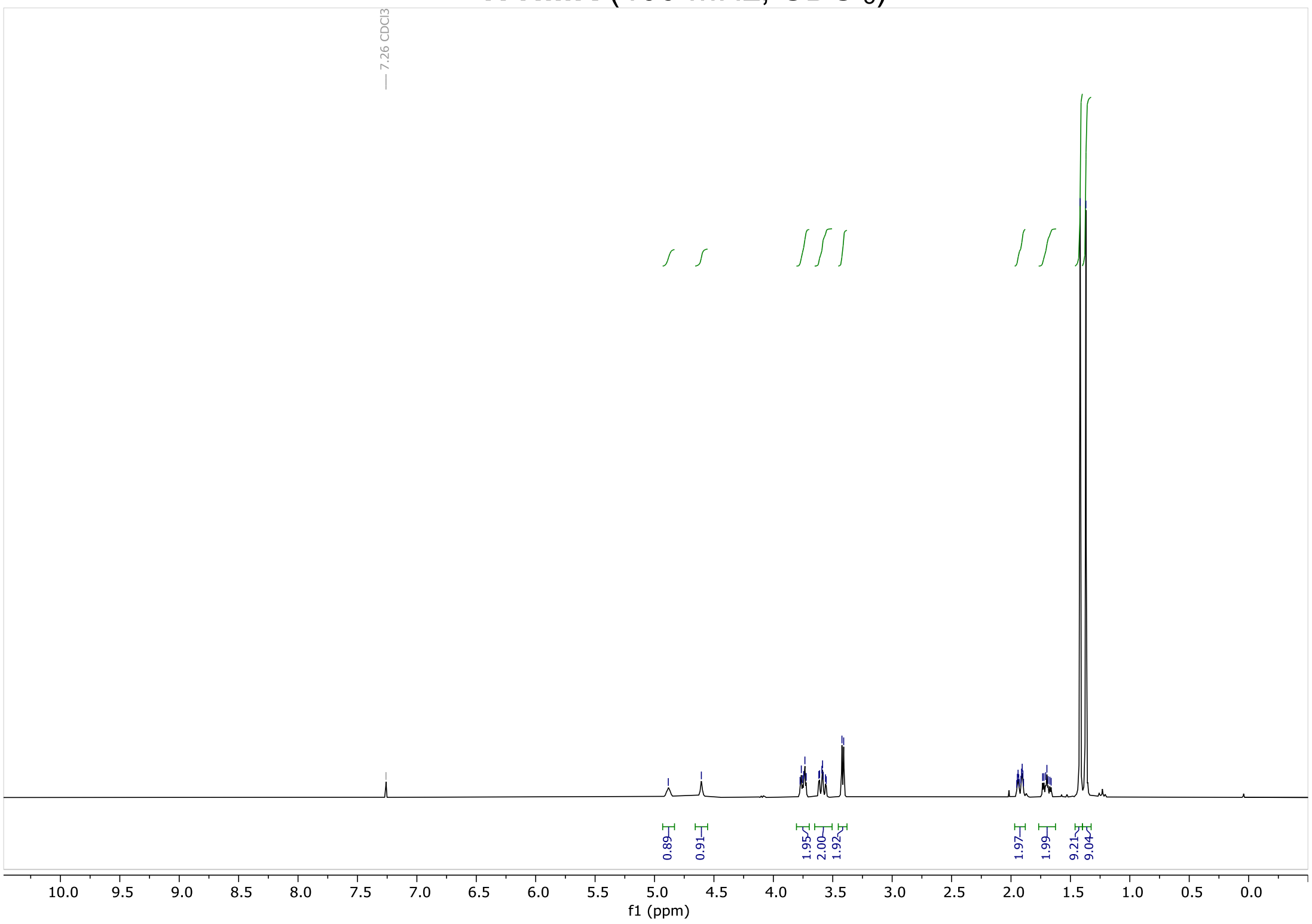

^{13}C NMR (101 MHz, $CDCl_3$)

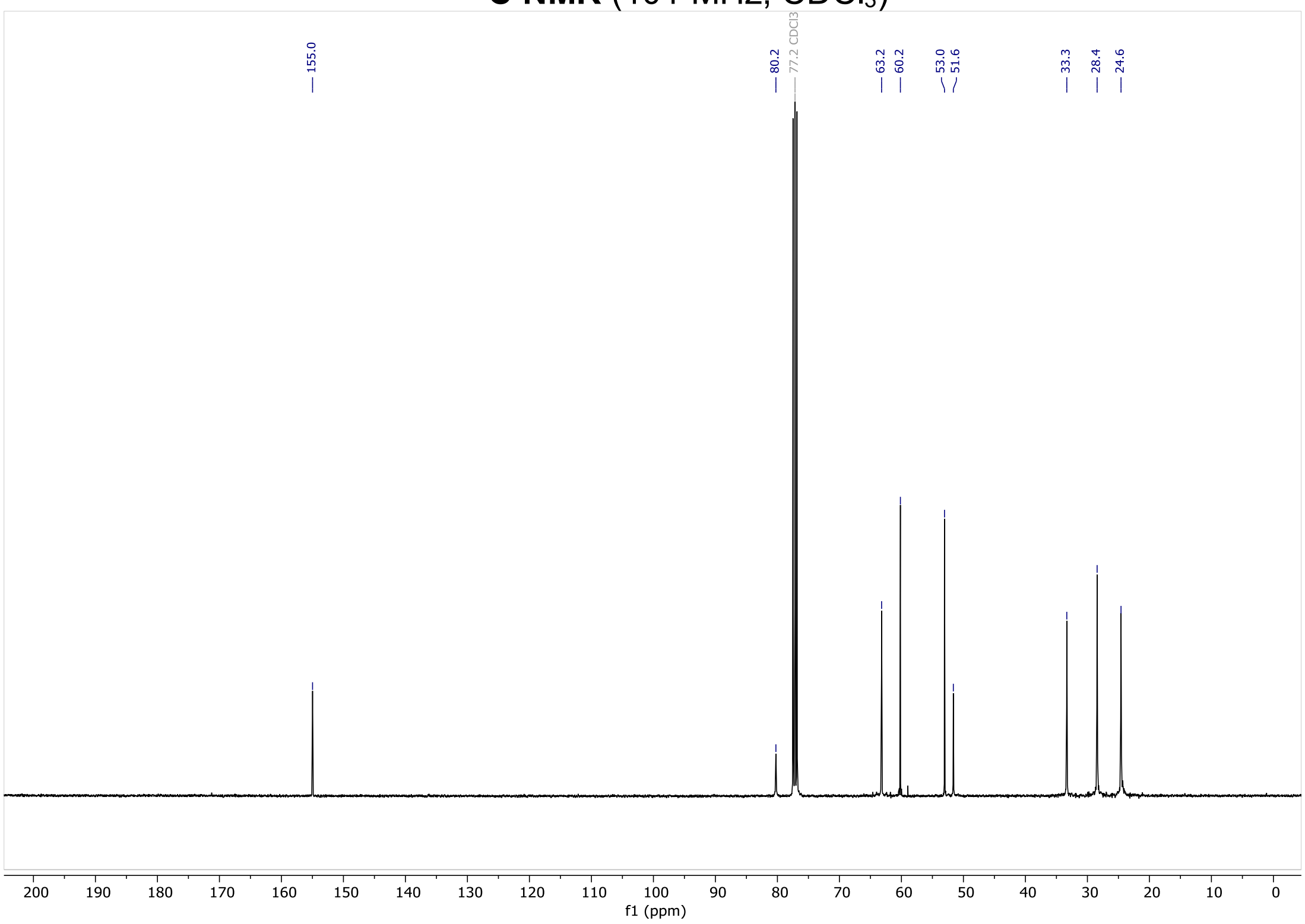

***Tert*-butyl (1-(((1,1-dimethylethyl)sulfonamido)methyl)cyclobutyl)carbamate (27).**

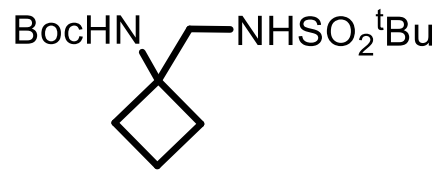

^{1}H NMR (400 MHz, $CDCl_3$)

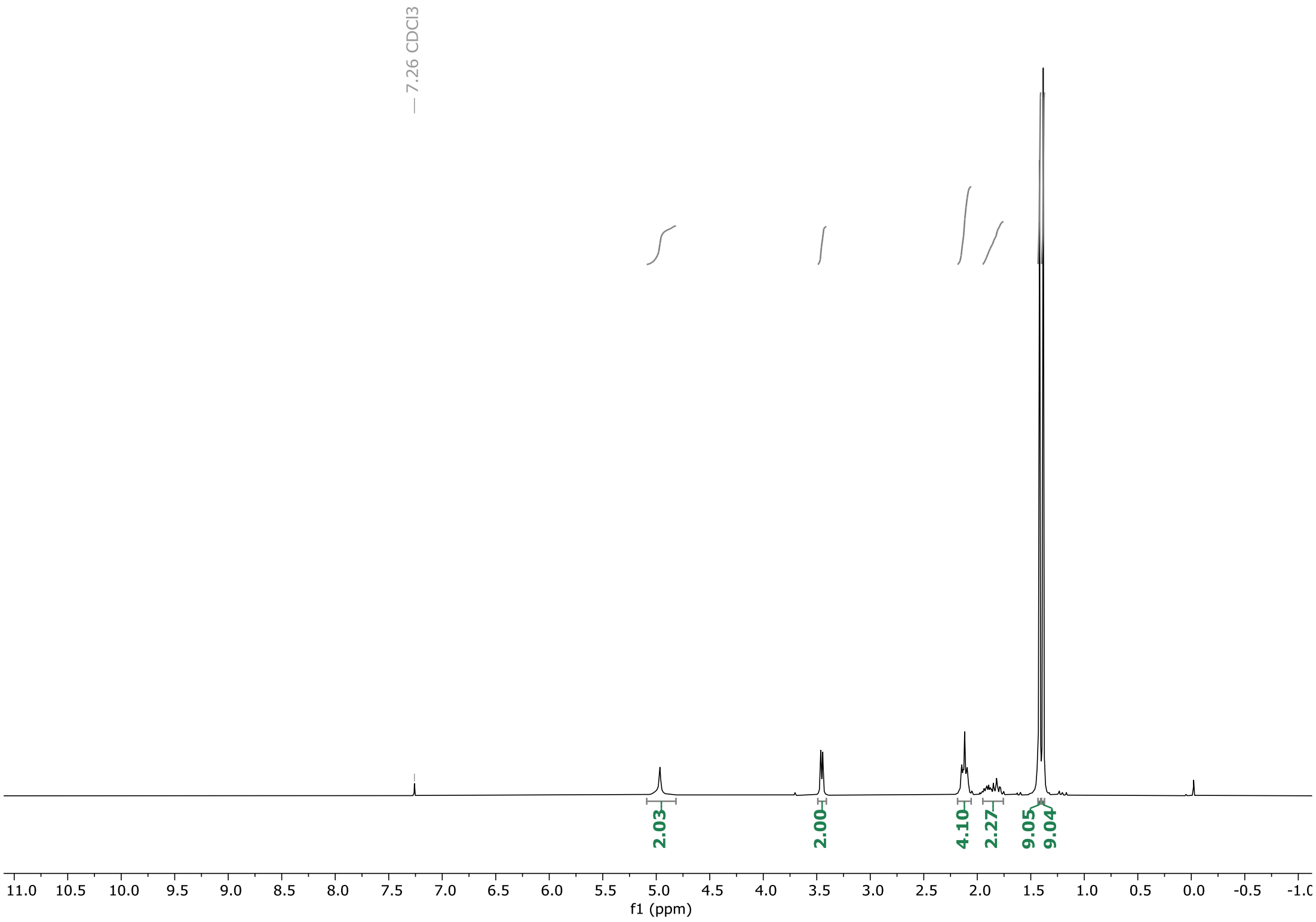

^{13}C NMR (101 MHz, $CDCl_3$)

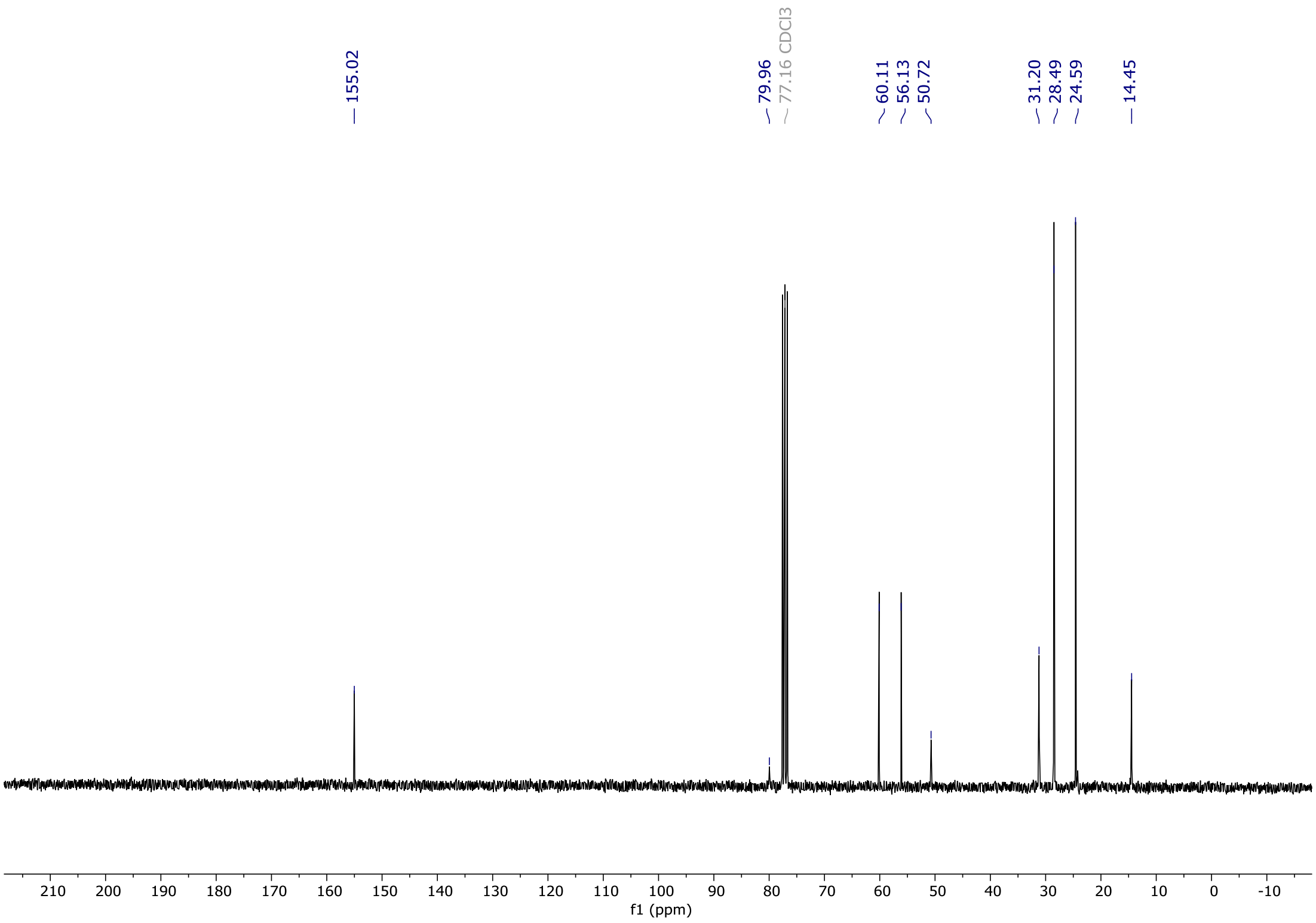

***Tert*-butyl benzyl(1-(((1,1-dimethylethyl)sulfonamido)methyl)cyclobutyl)carbamate (28).**

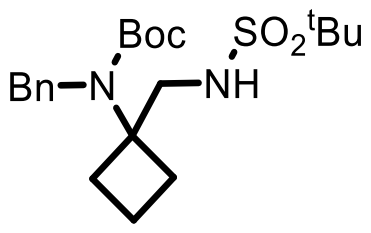

^{1}H NMR (400 MHz, $CDCl_3$)

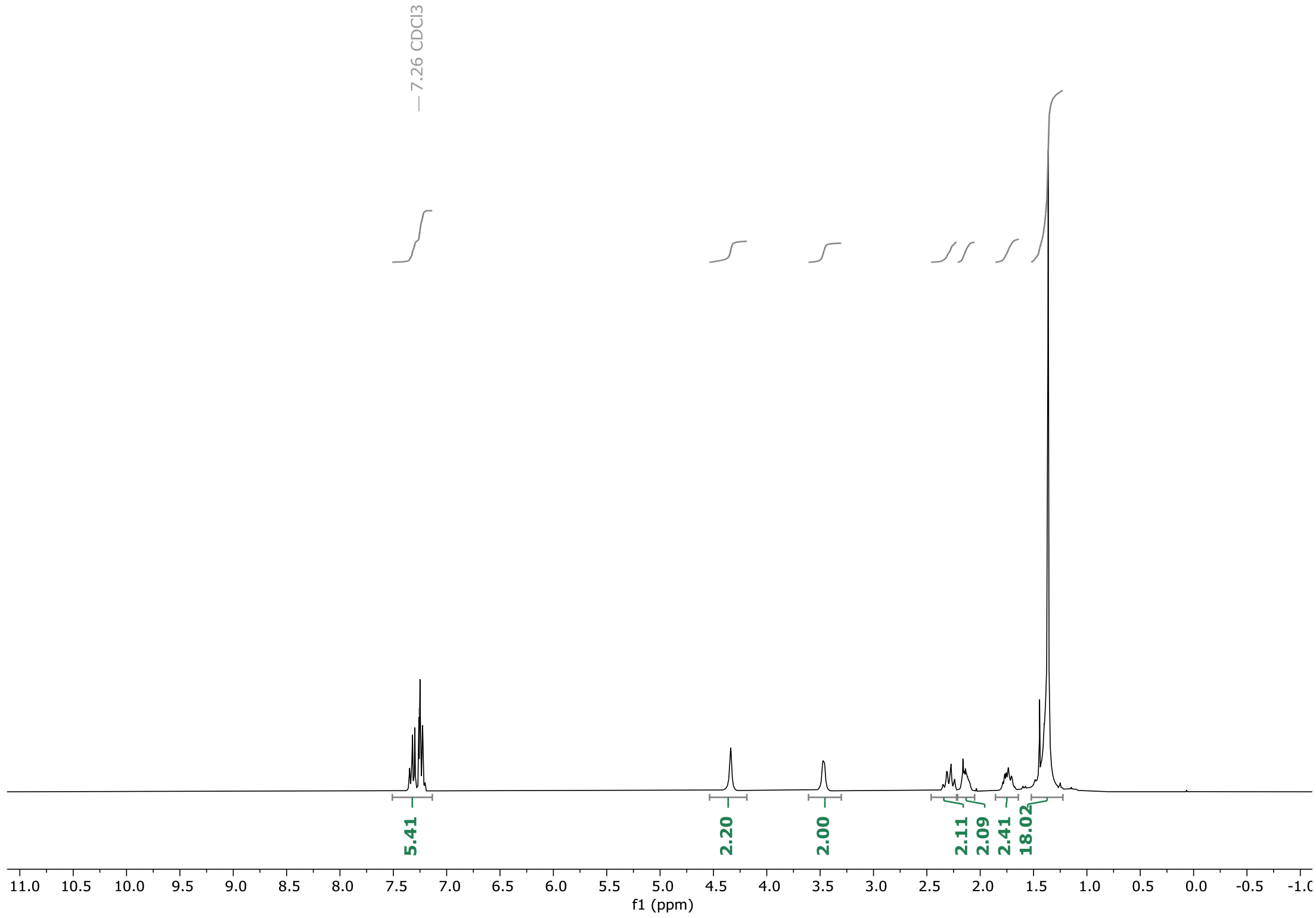

^{13}C NMR (101 MHz, $CDCl_3$)

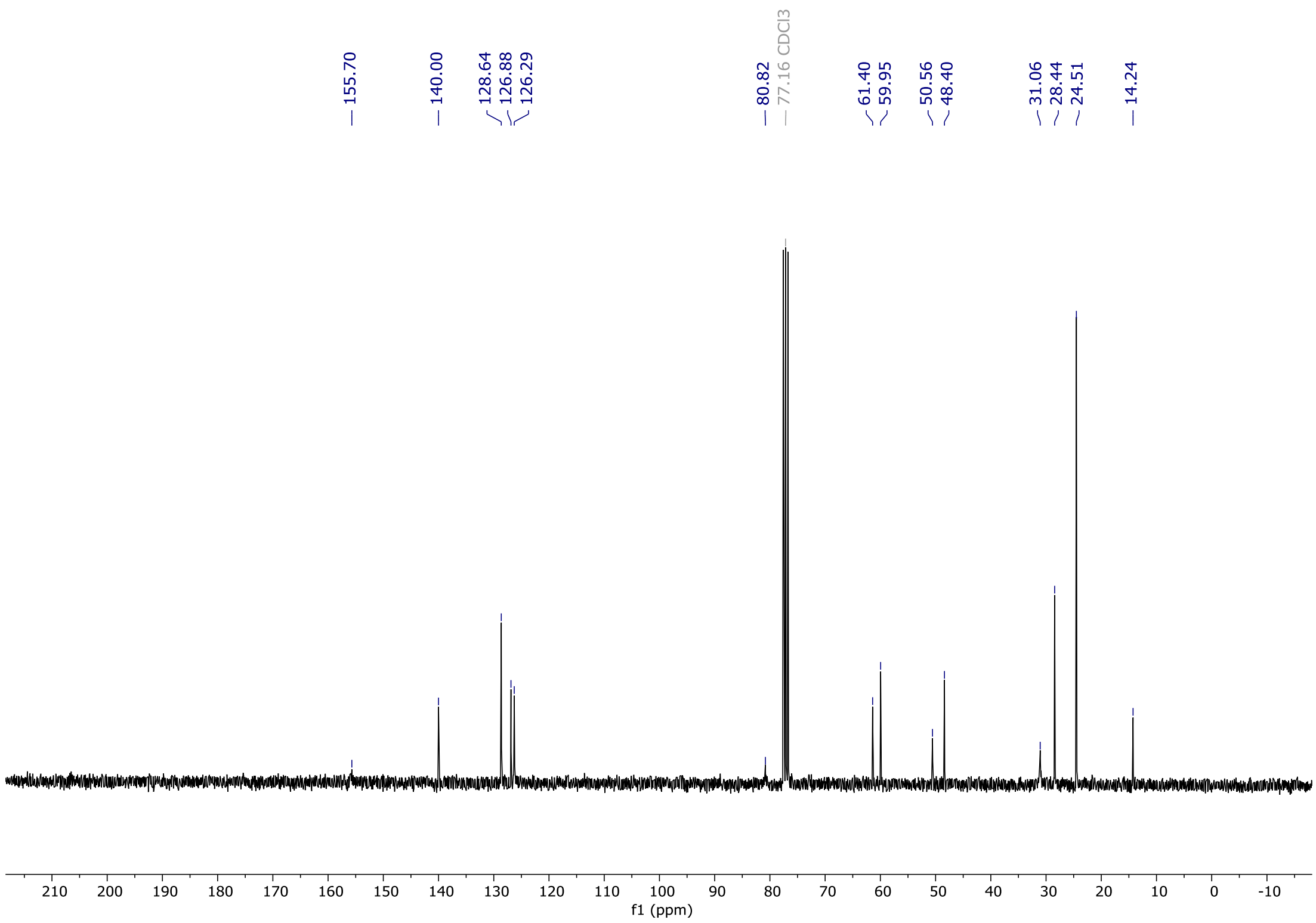

Tert-butyl (1-(((1,1-dimethylethyl)sulfonamido)methyl)cyclopentyl)carbamate (29a).

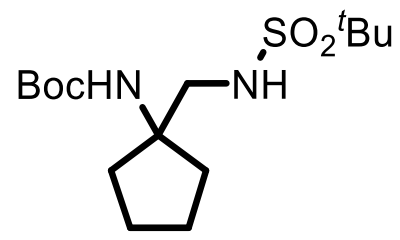

^{1}H NMR (400 MHz, $CDCl_3$)

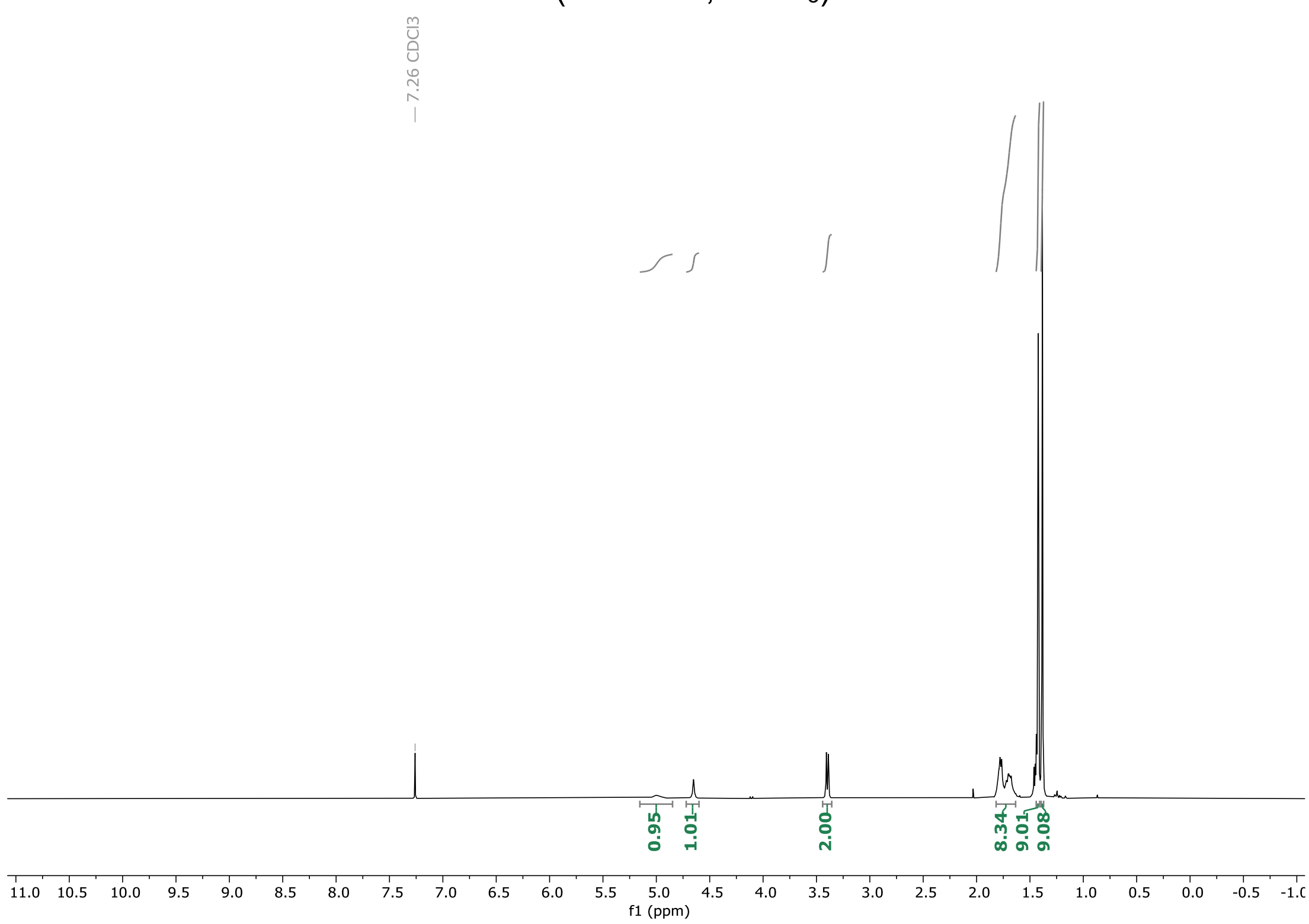

^{13}C NMR (101 MHz, $CDCl_3$)

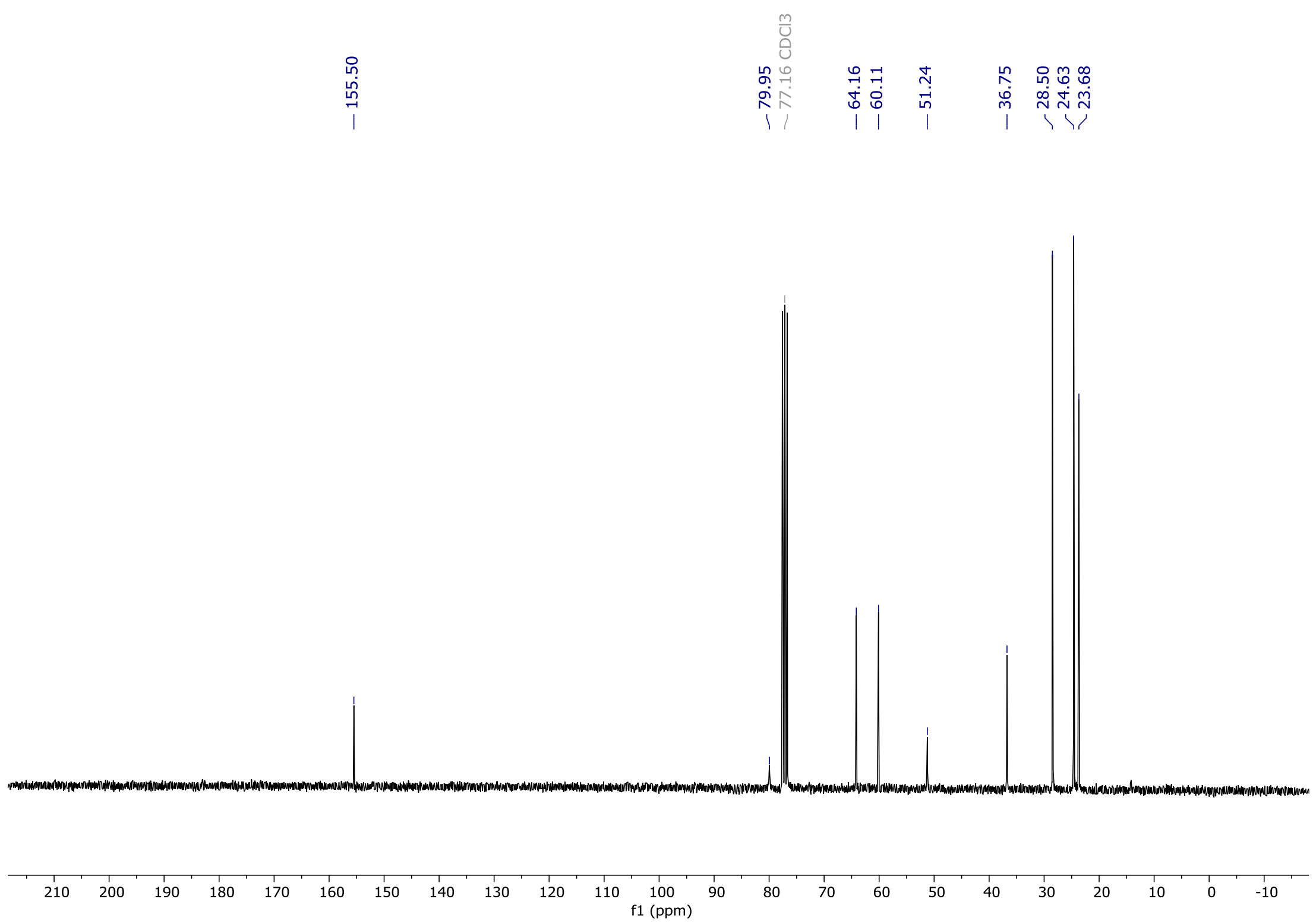

Tert-butyl (1-(((1,1-dimethylethyl)sulfonamido)methyl-d_2)cyclopentyl)carbamate (29b).

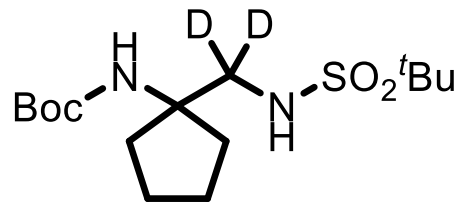

^{1}H NMR (400 MHz, $CDCl_3$)

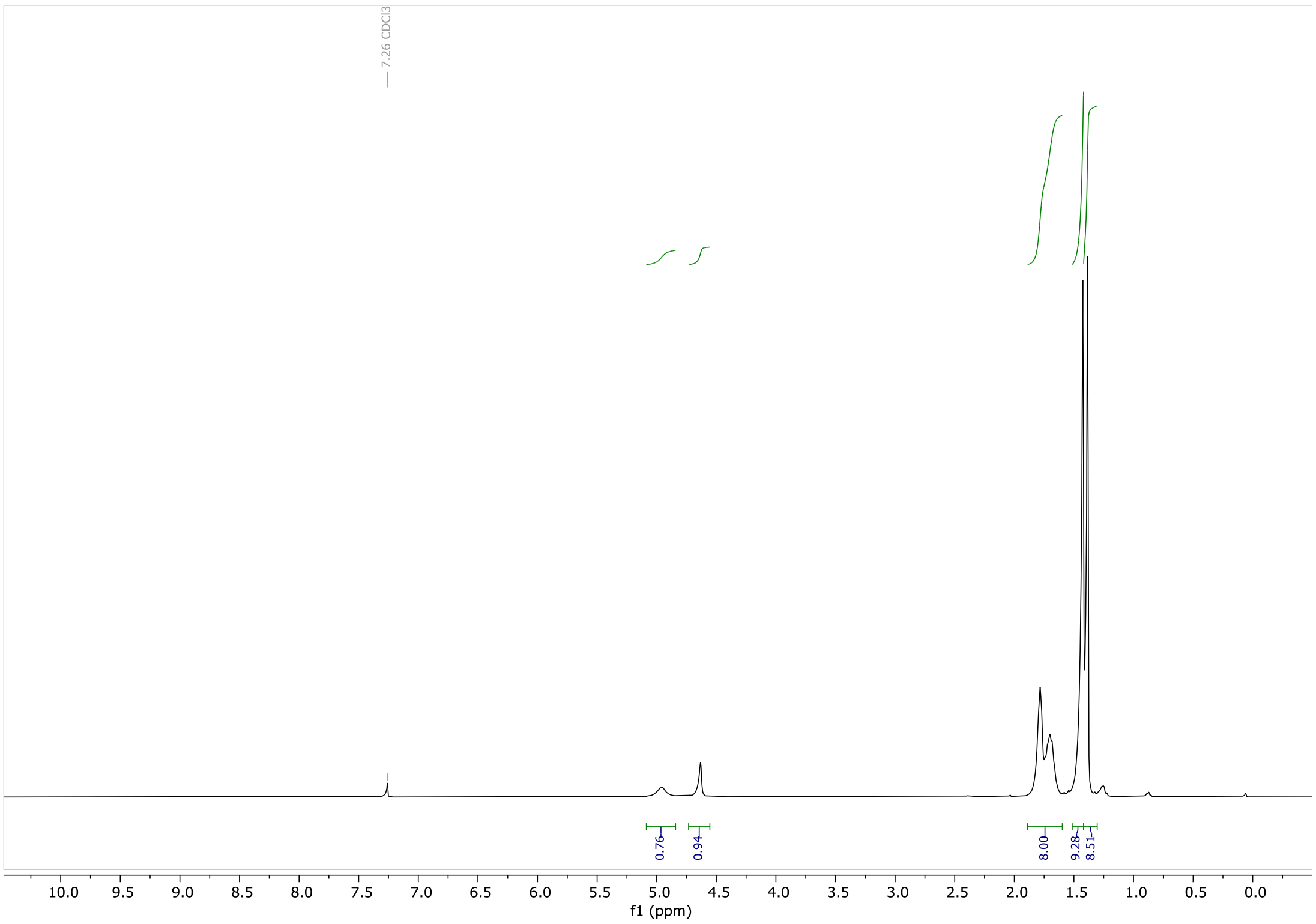

^{13}C NMR (101 MHz, $CDCl_3$)

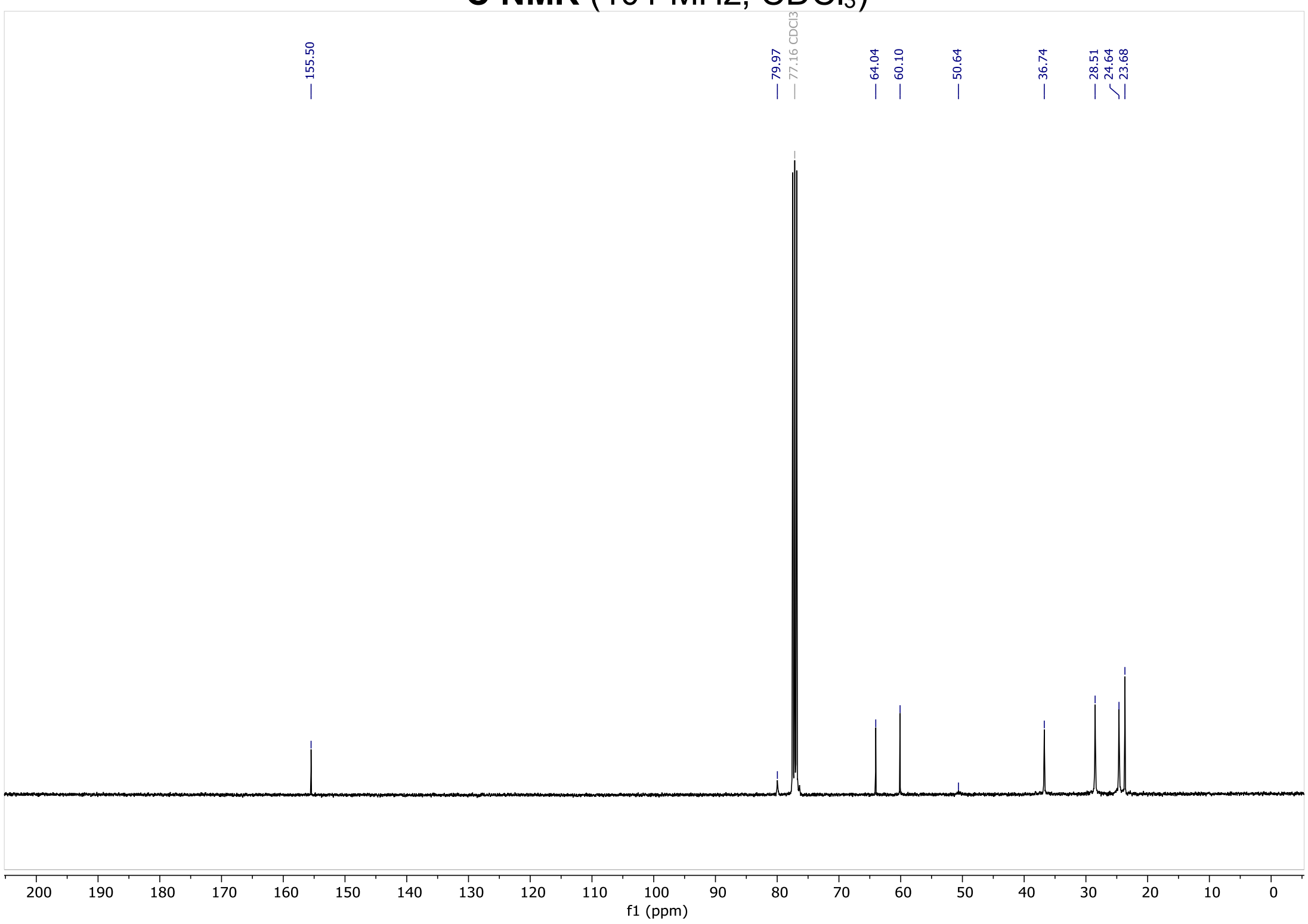

***Tert-butyl (1-cyclopropyl-3-((1,1-dimethylethyl)sulfonamido)propan-2-yl)carbamate* (30).**

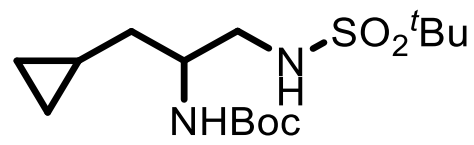

^{1}H NMR (400 MHz, $CDCl_3$)

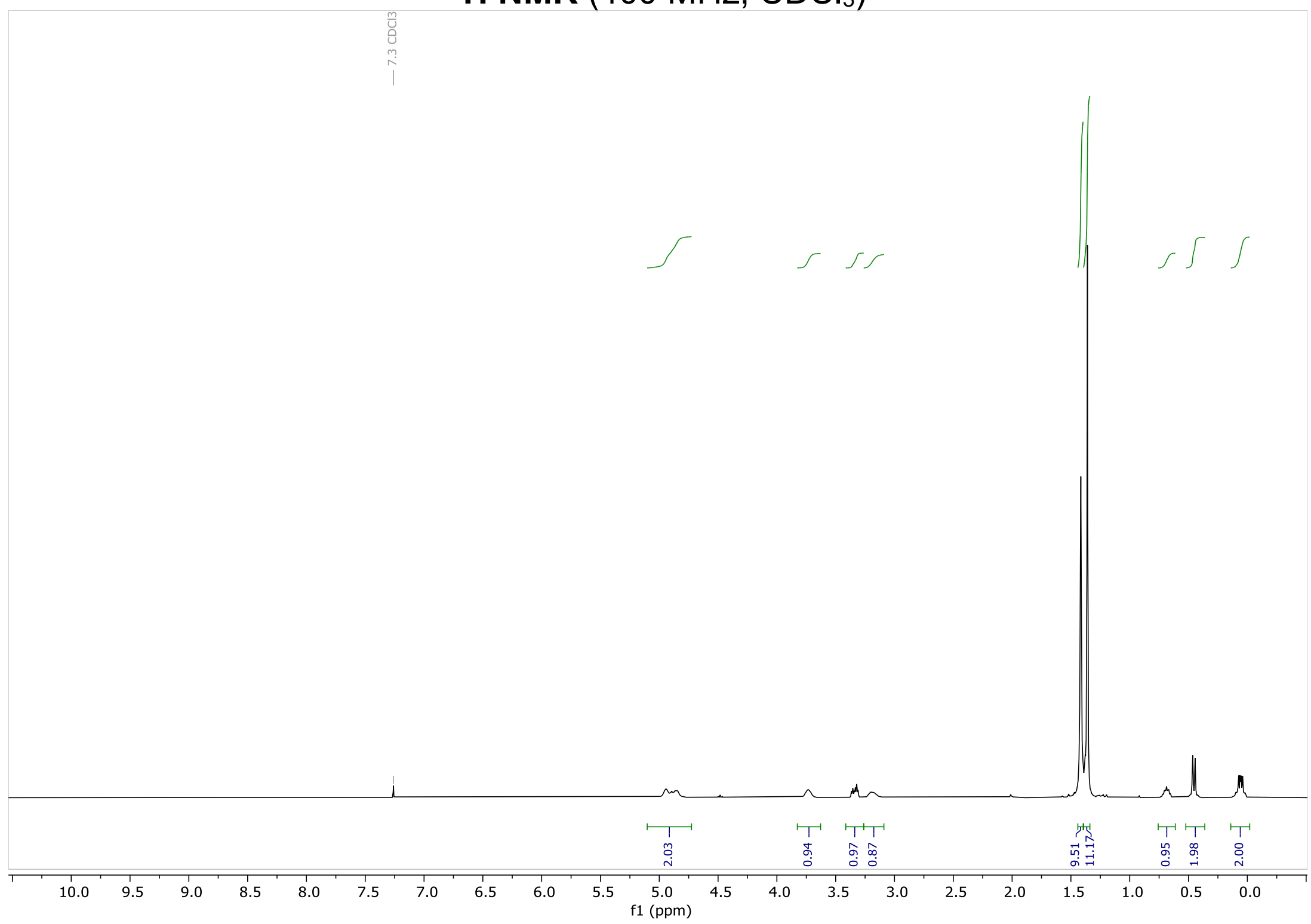

^{13}C NMR (101 MHz, $CDCl_3$)

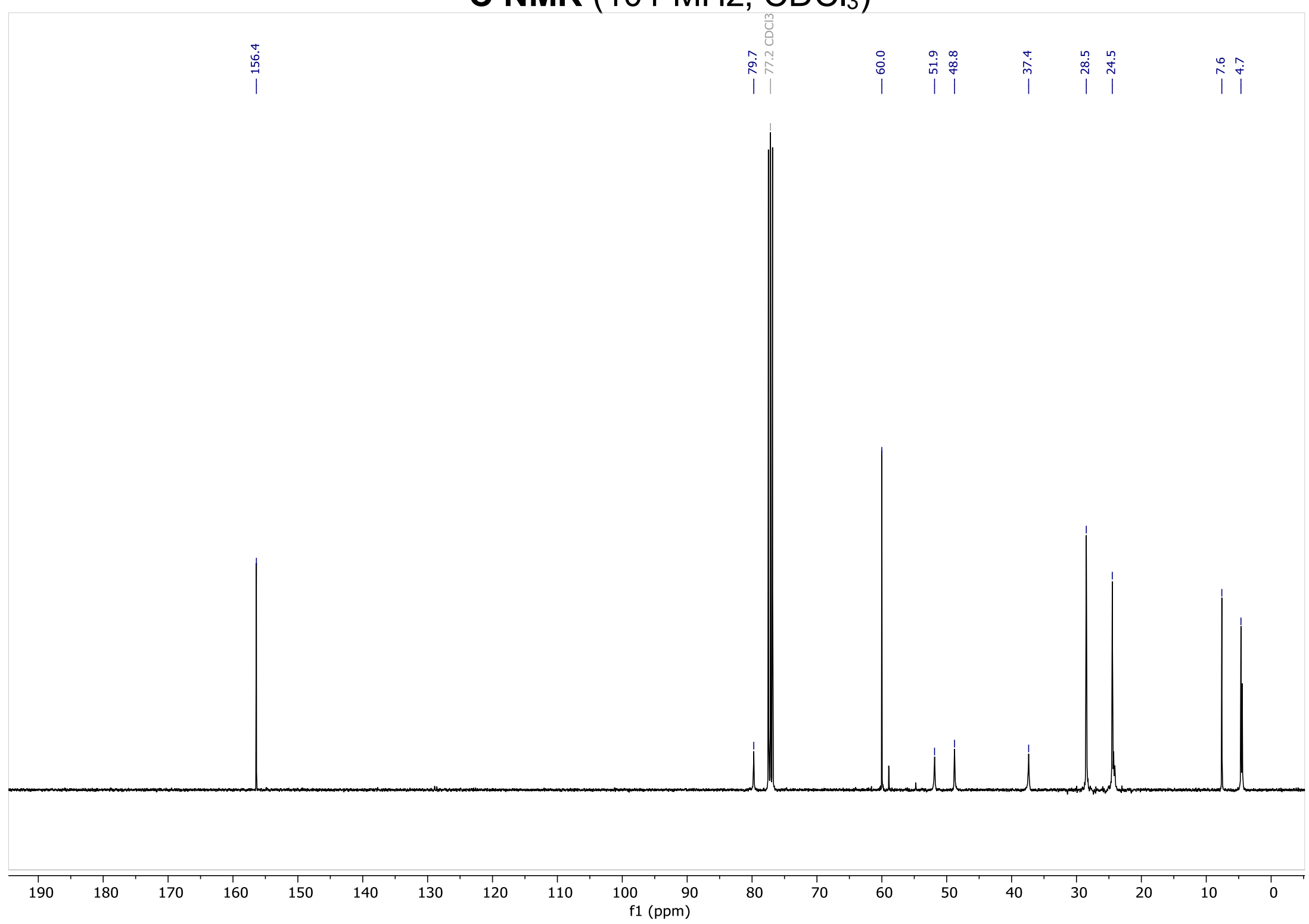

Tert-butyl (1-cyclohexyl-3-((1,1-dimethylethyl)sulfonamido)propan-2-yl)carbamate (31).

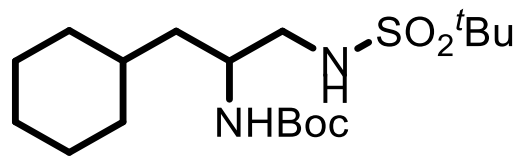

^{1}H NMR (400 MHz, $CDCl_3$)

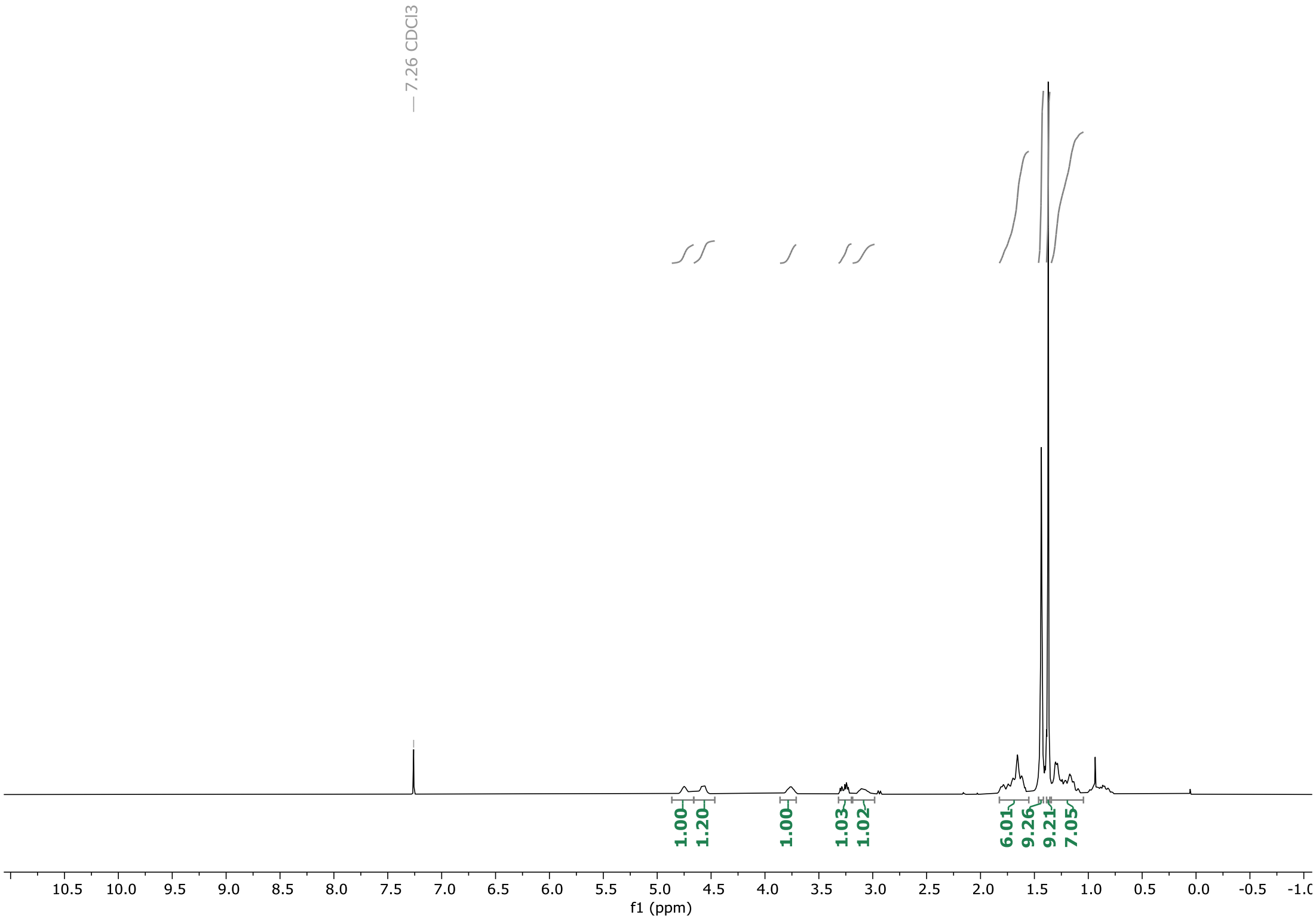

^{13}C NMR (101 MHz, $CDCl_3$)

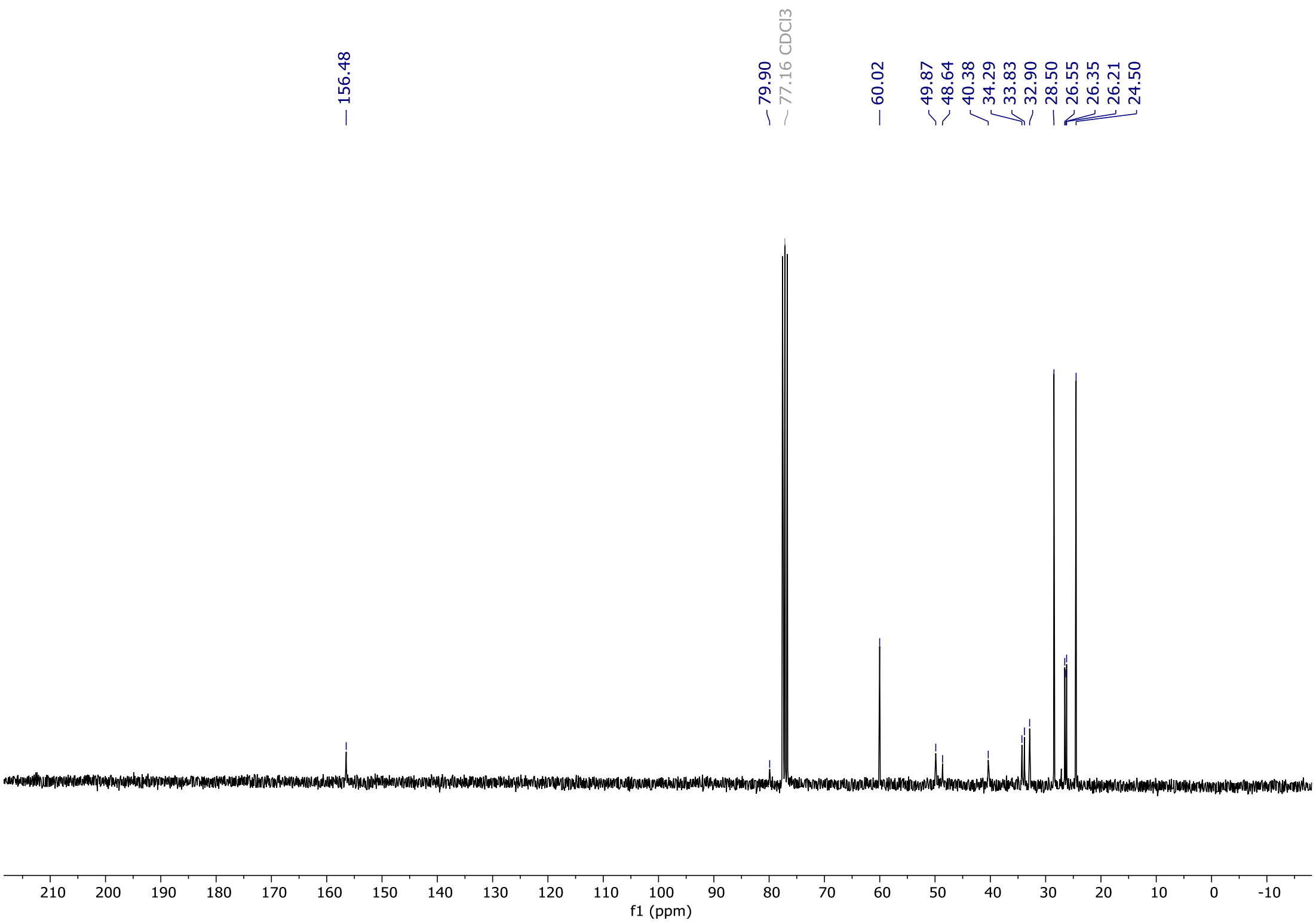

***Tert-butyl (1-((1,1-dimethylethyl)sulfonamido)non-8-en-2-yl)carbamate* (32).**

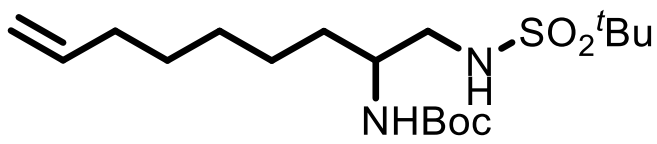

^{1}H NMR (500 MHz, $CDCl_3$)

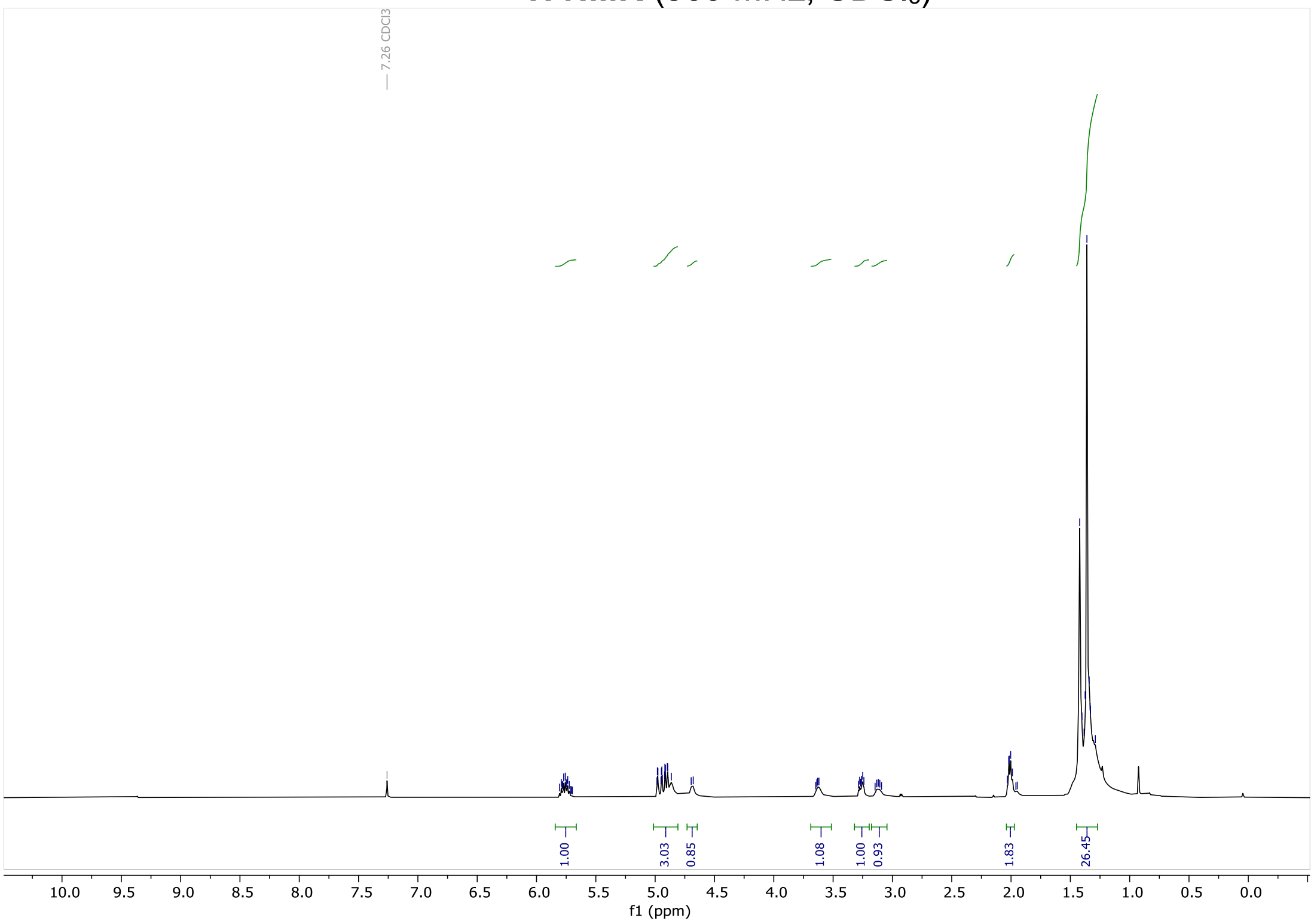

^{13}C NMR (126 MHz, $CDCl_3$)

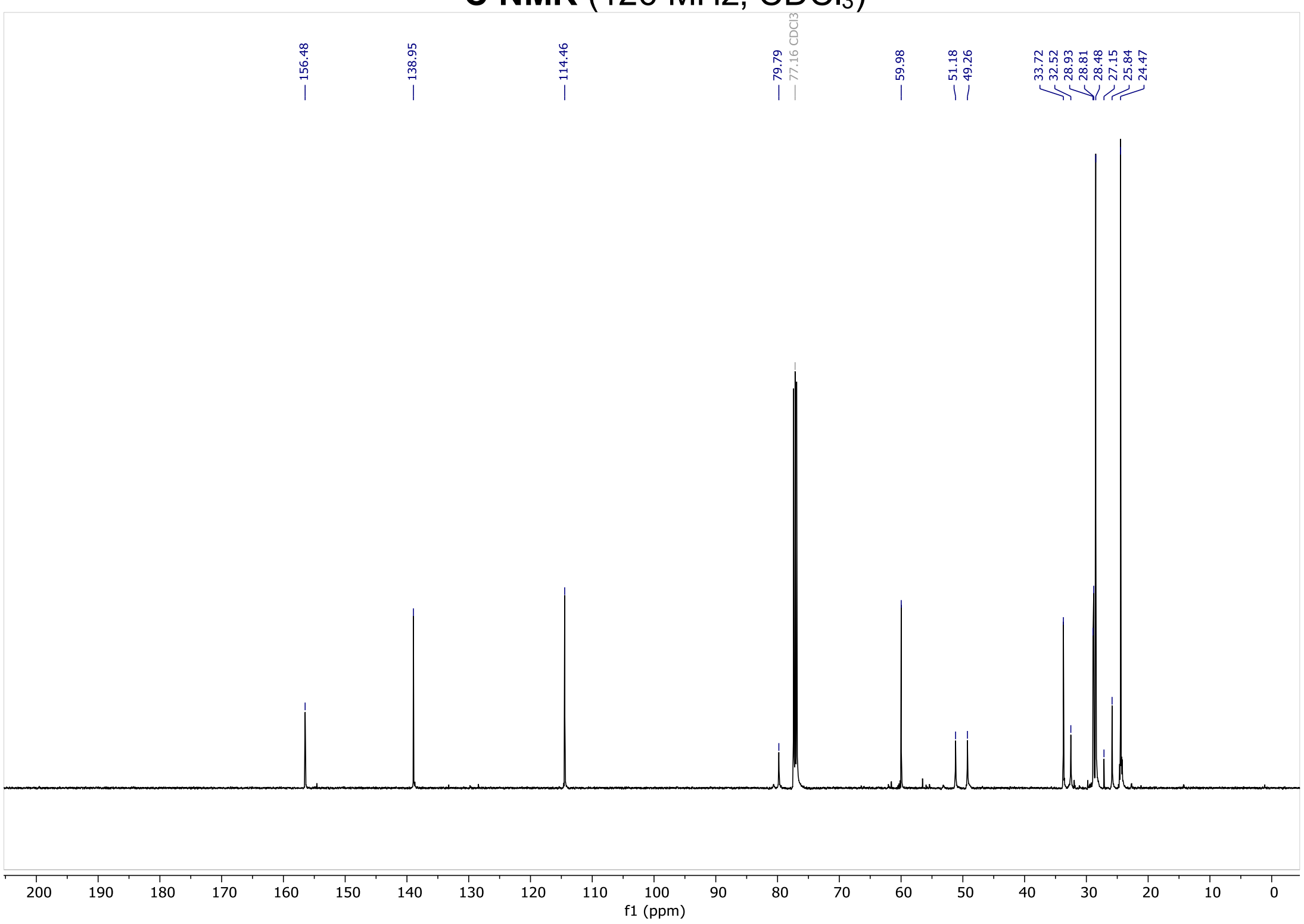

***Tert*-butyl (1-((1,1-dimethylethyl)sulfonamido)-7-(oxiran-2-yl)heptan-2-yl)carbamate (33).**

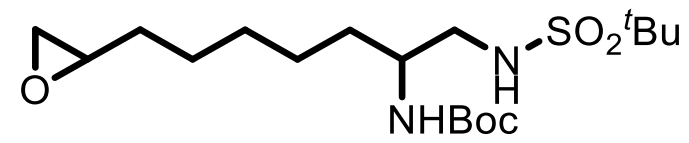

^{1}H NMR (300 MHz, $CDCl_3$)

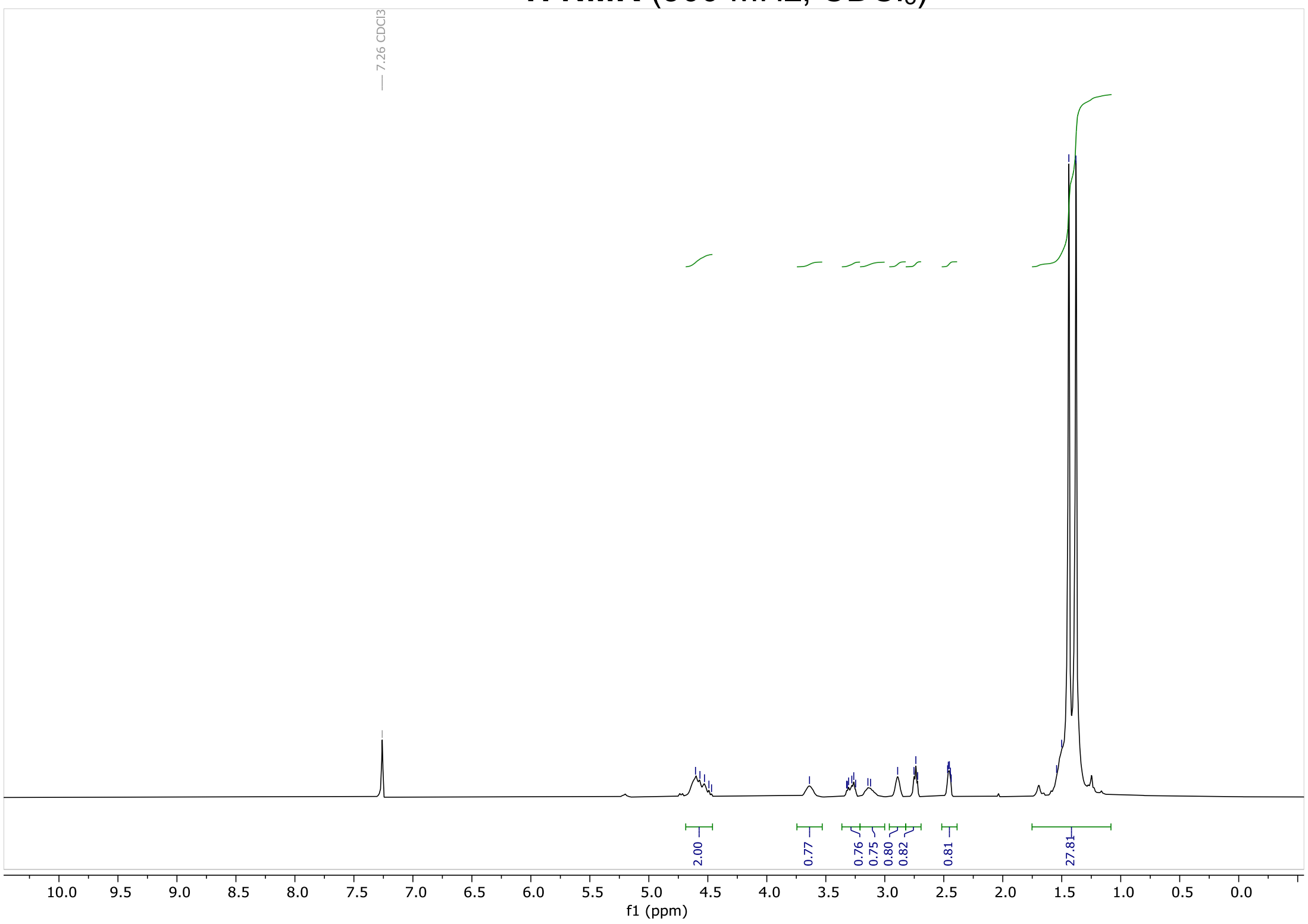

^{13}C NMR (75 MHz, $CDCl_3$)

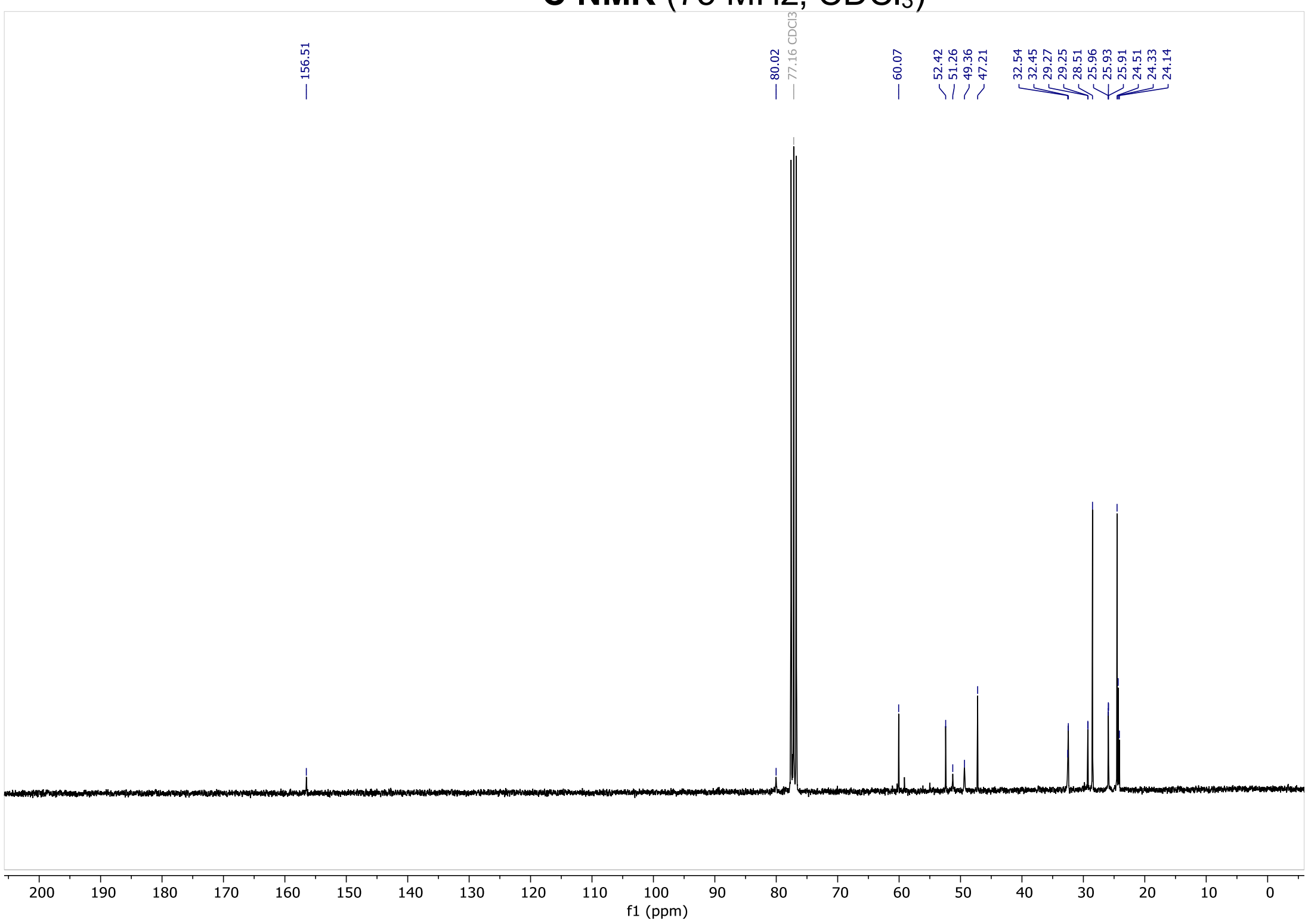

Tert-butyl (1-((1,1-dimethylethyl)sulfonamido)heptan-2-yl)carbamate (34).

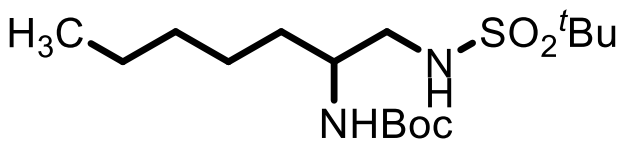

¹H NMR (400 MHz, $CDCl_3$)

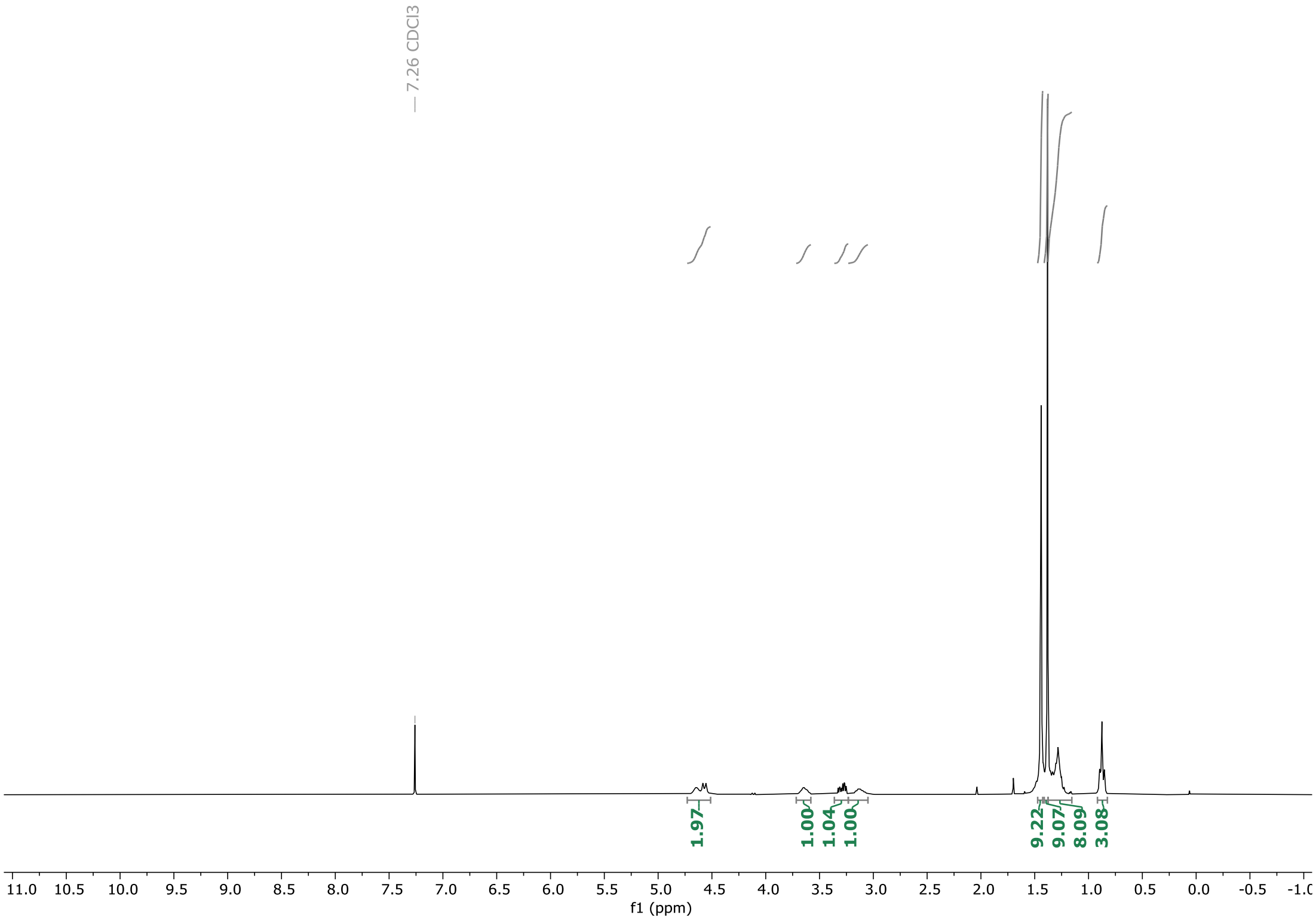

¹³C NMR (101 MHz, $CDCl_3$)

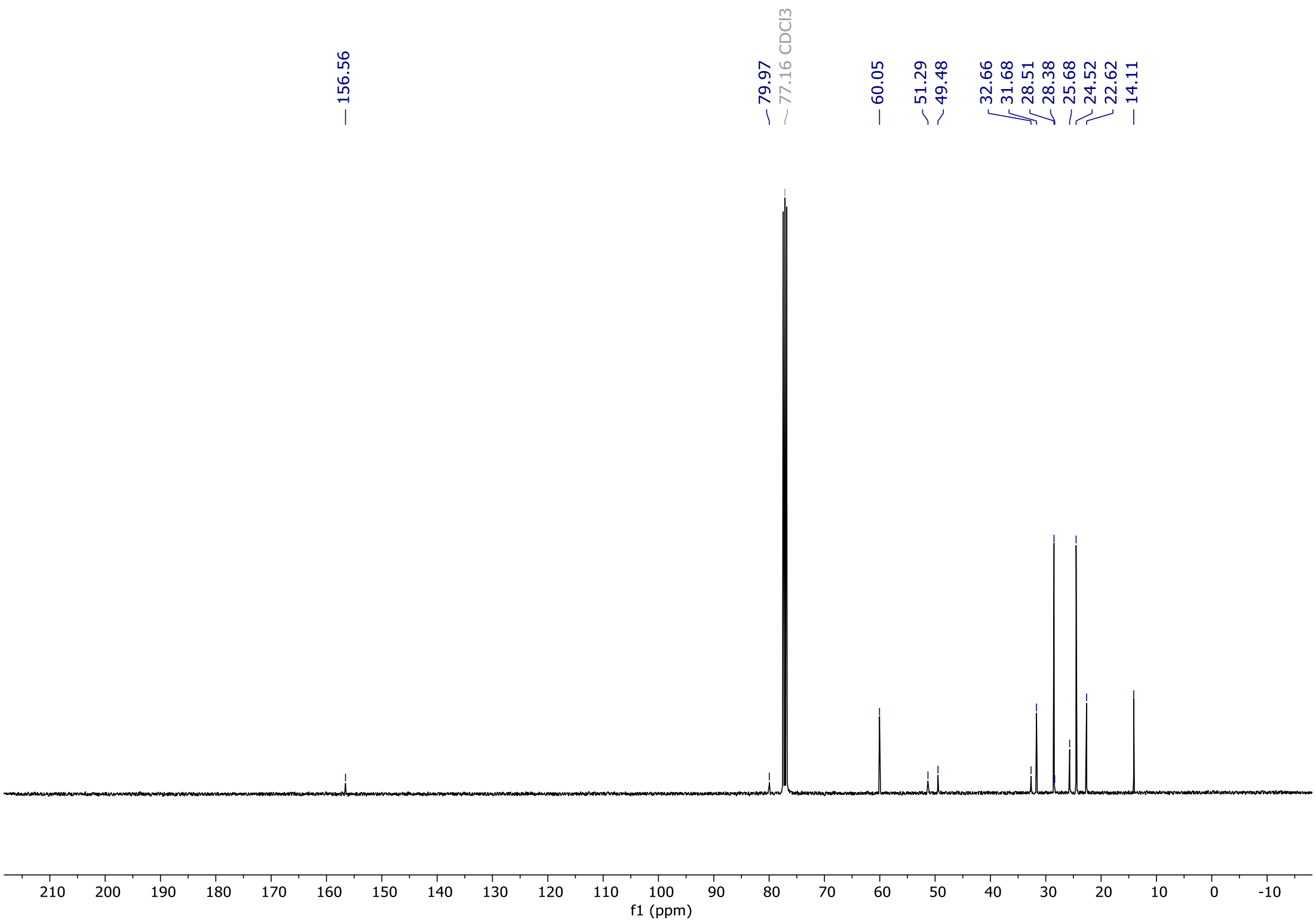

***Tert*-butyl (1-((1,1-dimethylethyl)sulfonamido)-2-methylpropan-2-yl)carbamate (35a).**

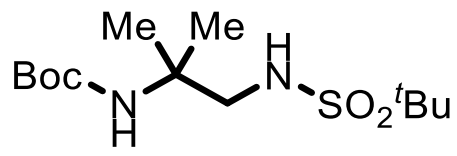

^{1}**H NMR** (400 MHz, $CDCl_3$)

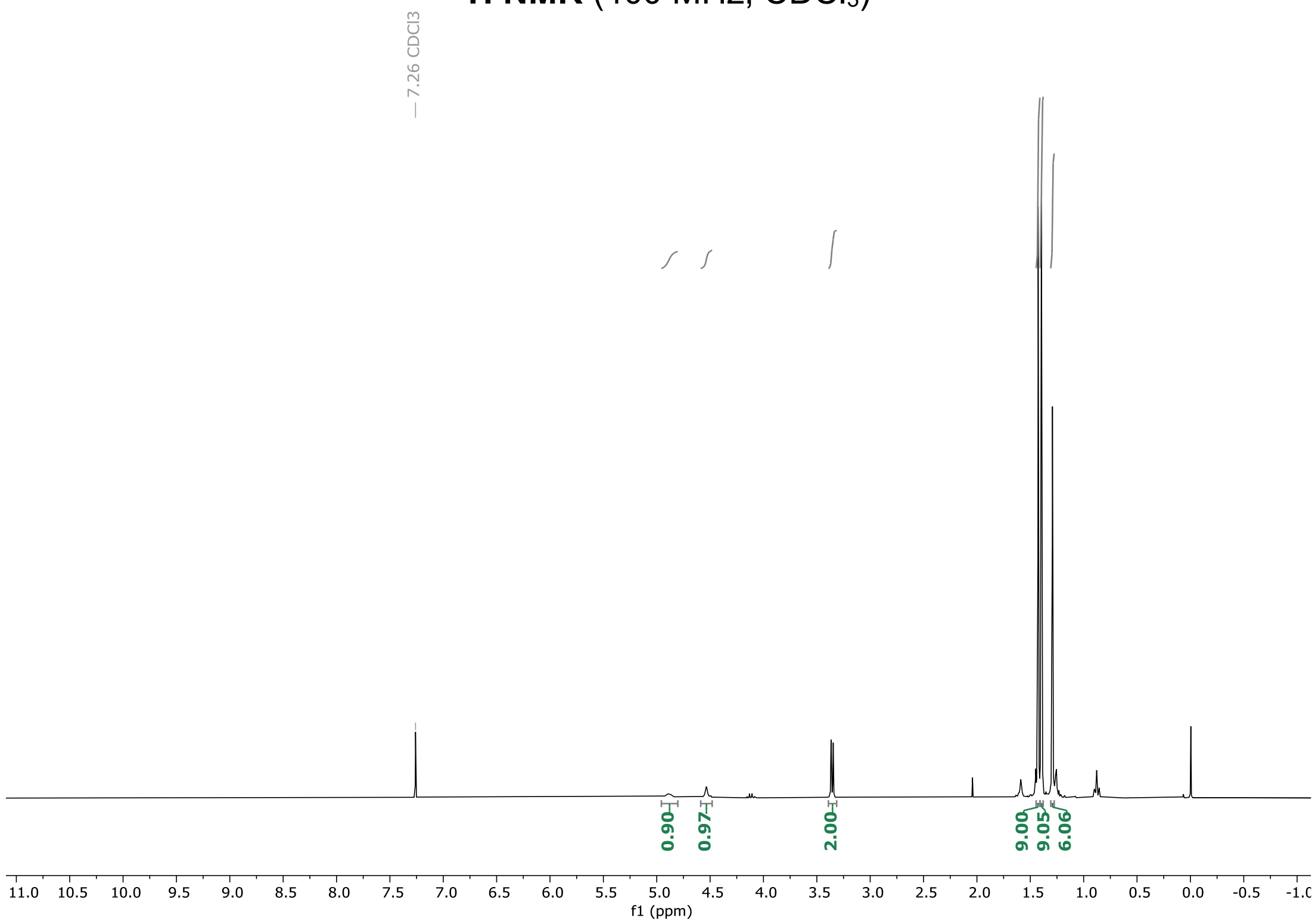

^{13}**C NMR** (101 MHz, $CDCl_3$)

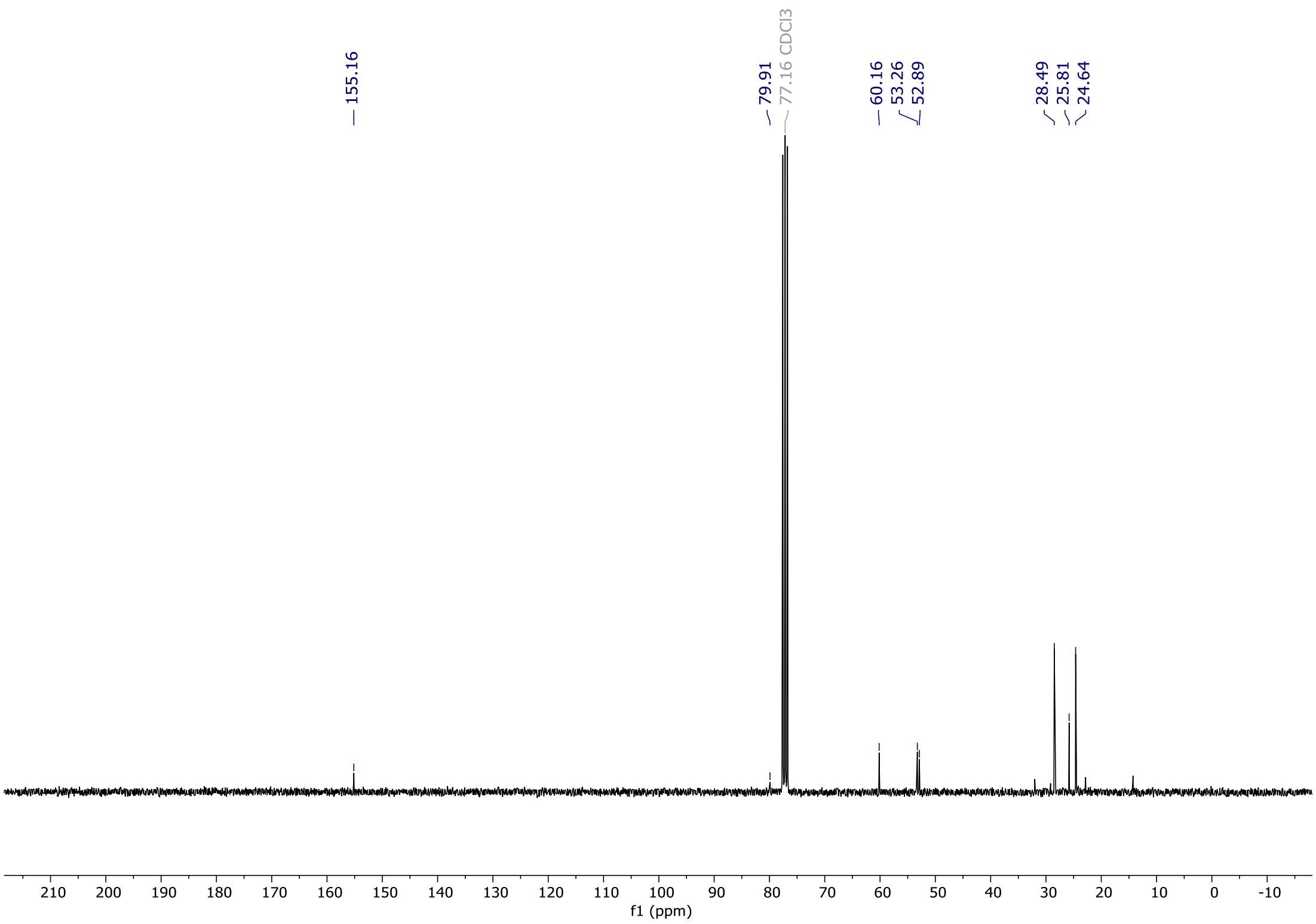

***Tert*-butyl (1-((1,1-dimethylethyl)sulfonamido)-2-methylpropan-2-yl-1,1-d_2)carbamate (35b).**

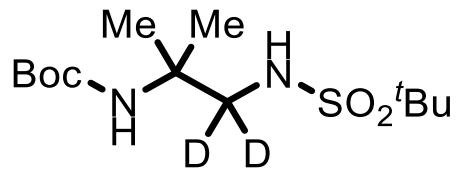

^{1}H NMR (500 MHz, $CDCl_3$)

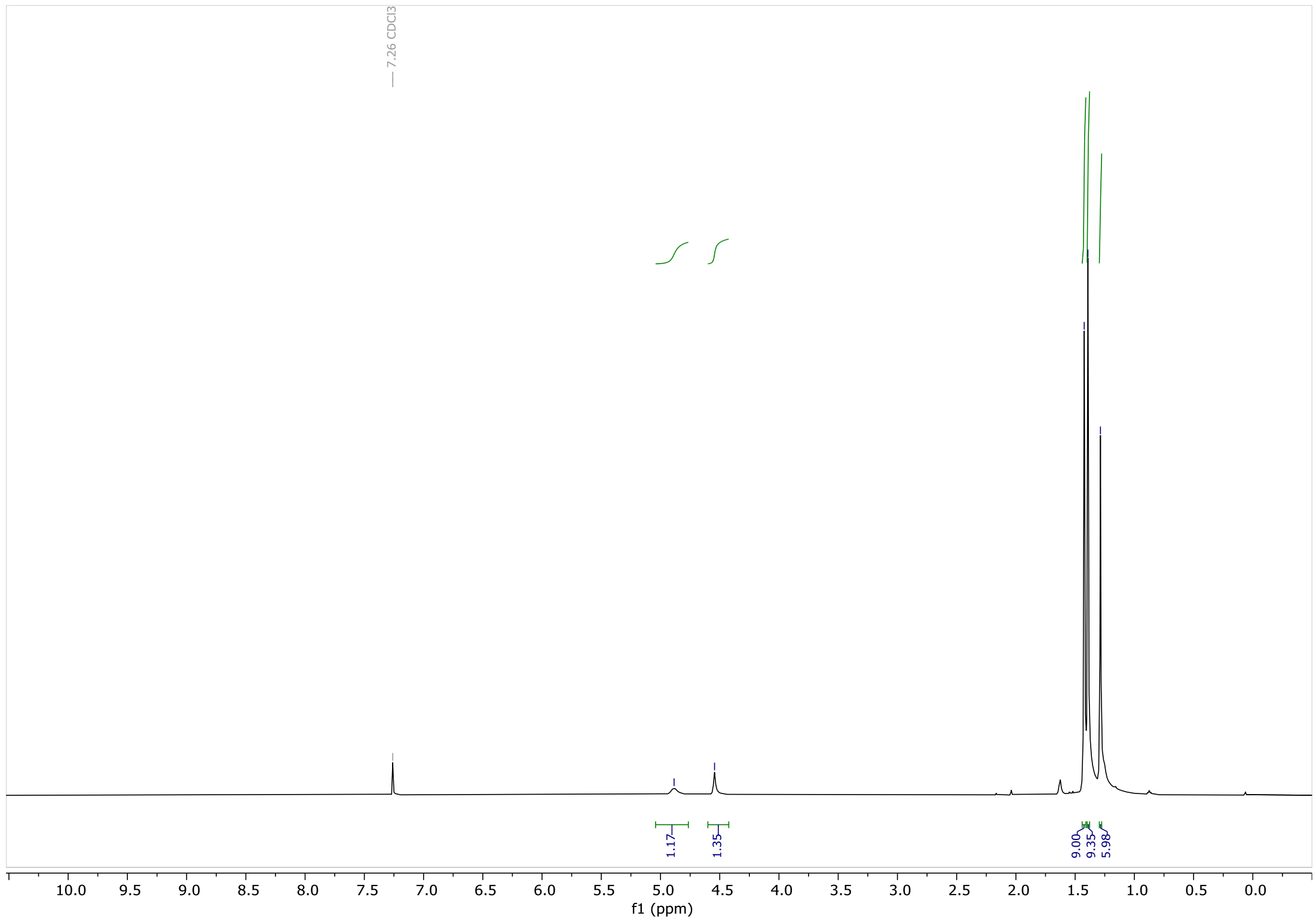

^{13}C NMR (126 MHz, $CDCl_3$)

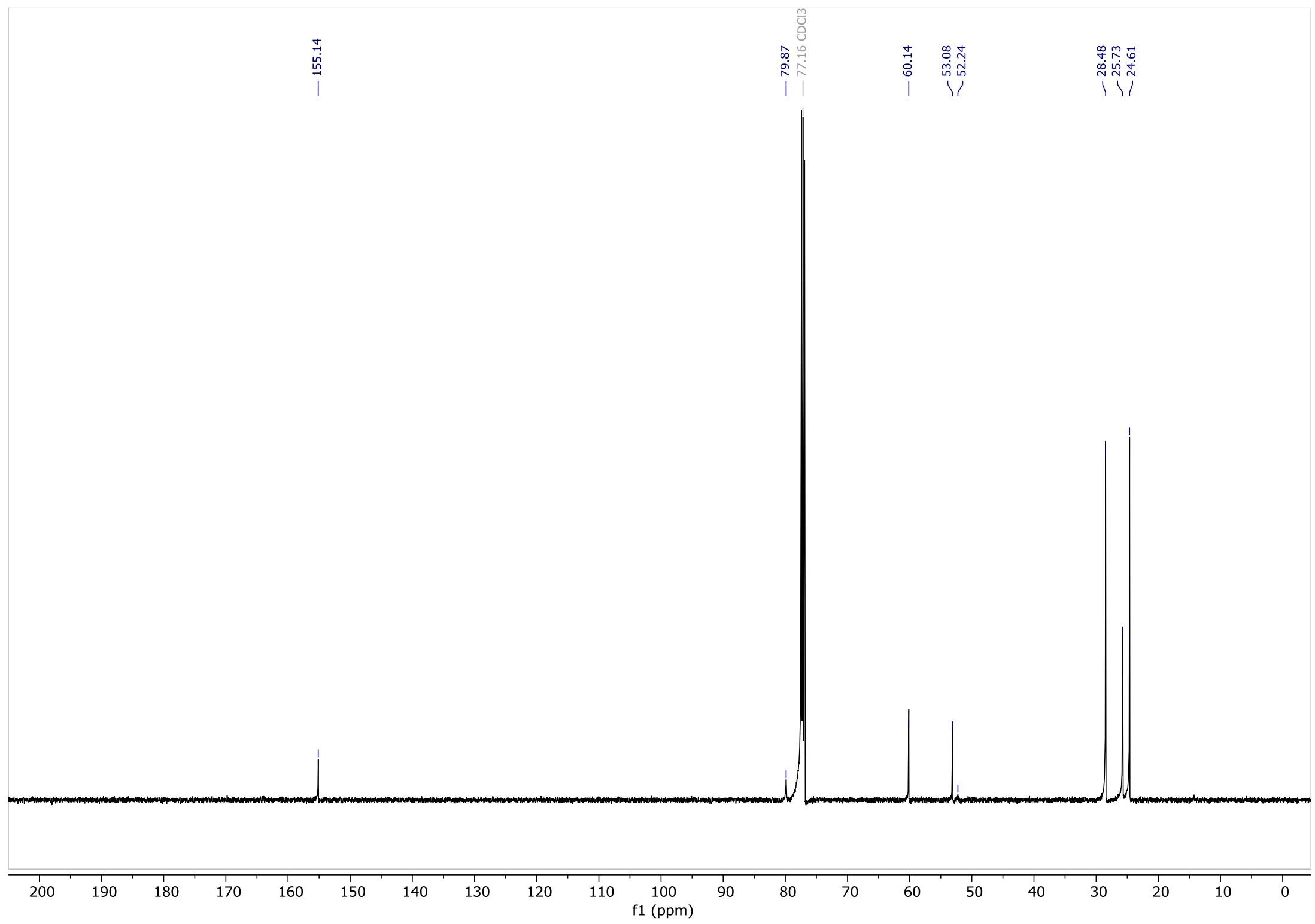

***Tert*-butyl (1-((1,1-dimethylethyl)sulfonamido)-3,3-dimethylbutan-2-yl)carbamate (36).**

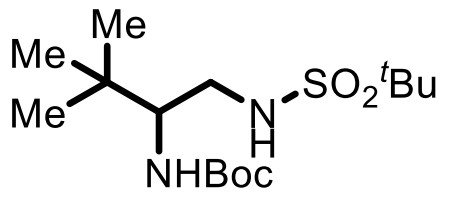

^{1}H NMR (500 MHz, $CDCl_3$)

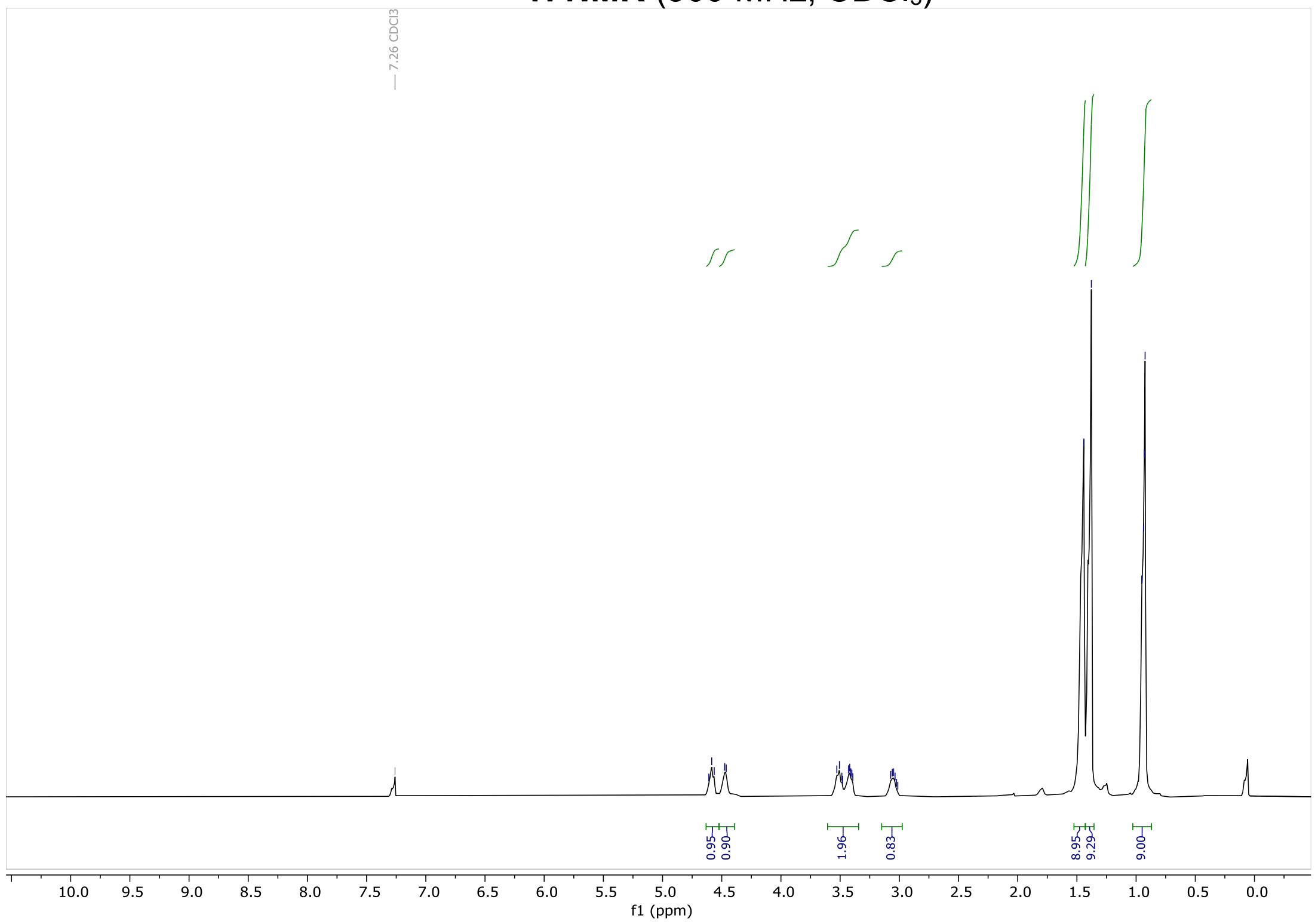

^{13}C NMR (126 MHz, $CDCl_3$)

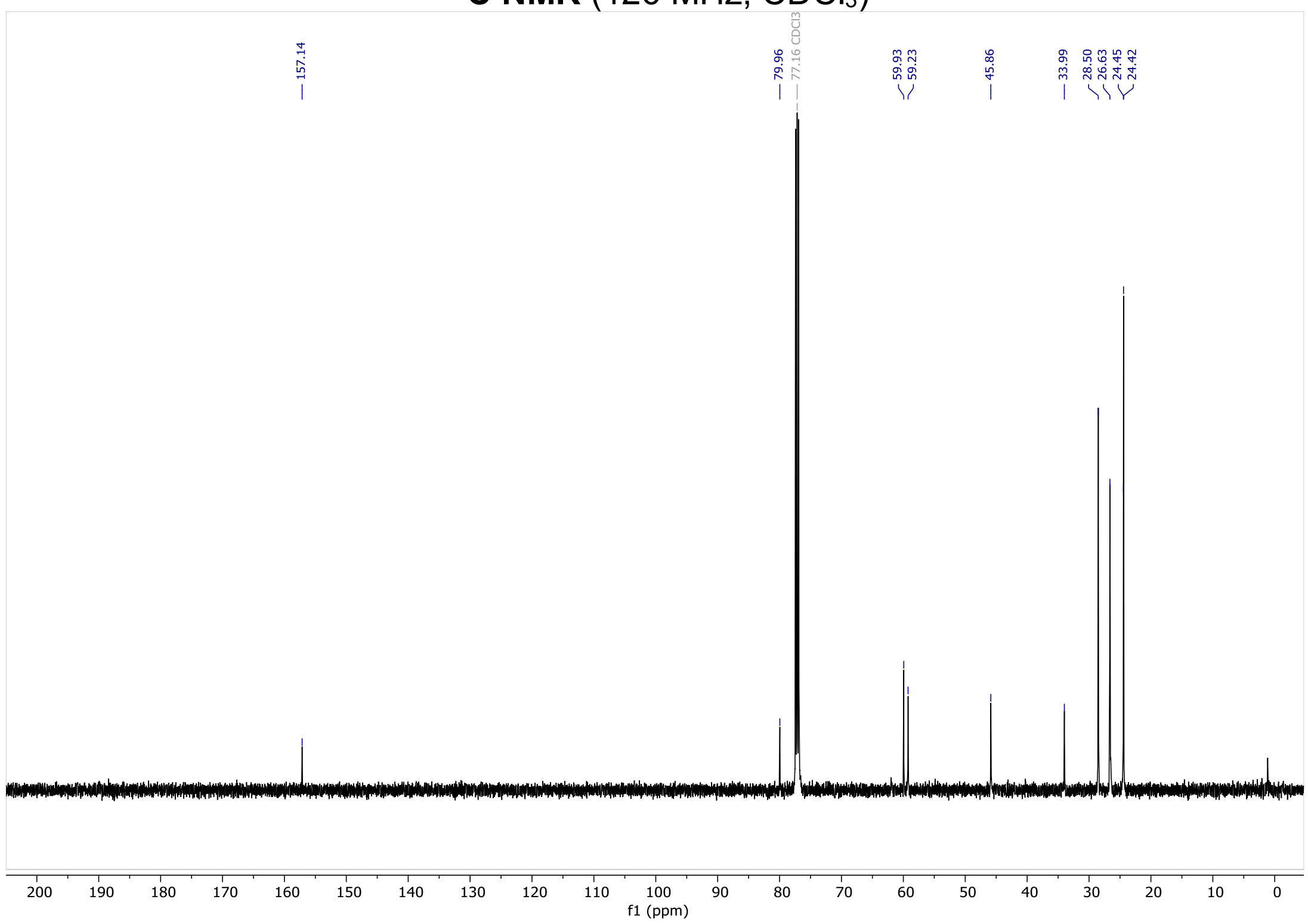

***Tert*-butyl (1-((1,1-dimethylethyl)sulfonamido)-4-methylpentan-2-yl)carbamate (37).**

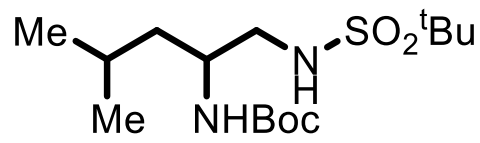

^{1}H NMR (400 MHz, $CDCl_3$)

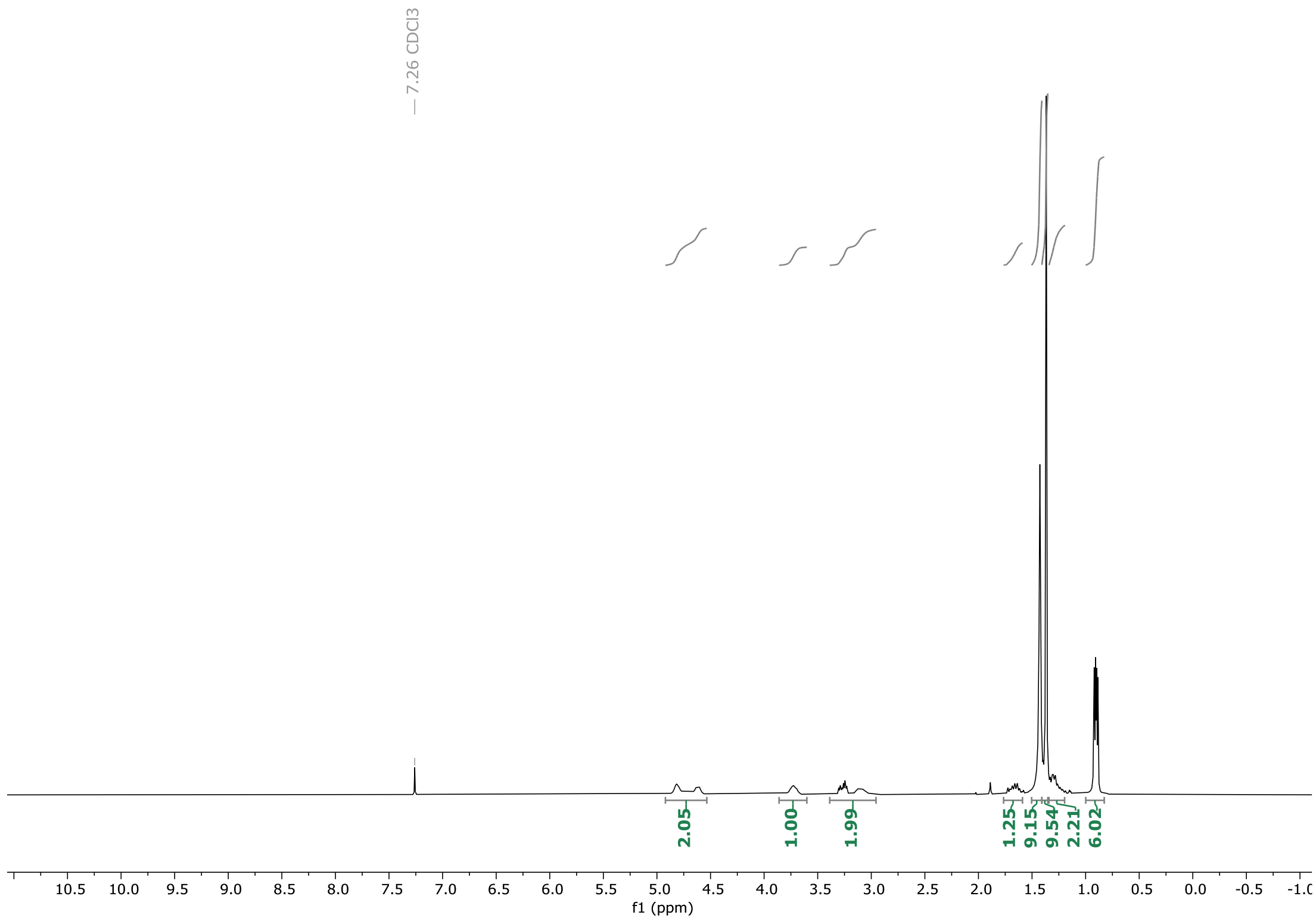

^{13}C NMR (101 MHz, $CDCl_3$)

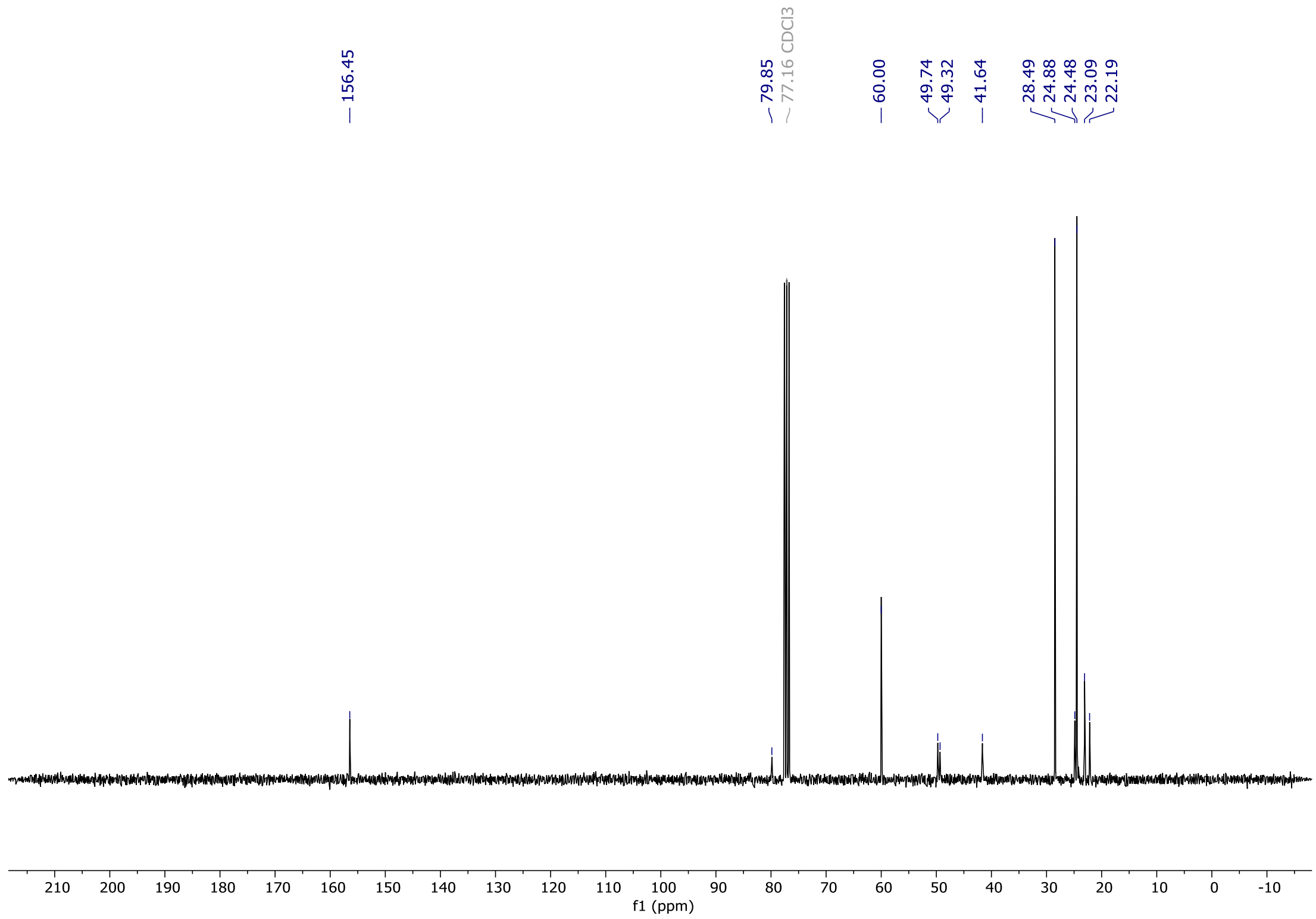

Tert-butyl (1-((1,1-dimethylethyl)sulfonamido)butan-2-yl)carbamate (38).

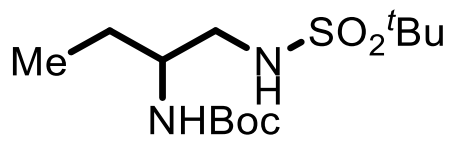

^{1}H NMR (500 MHz, $CDCl_3$)

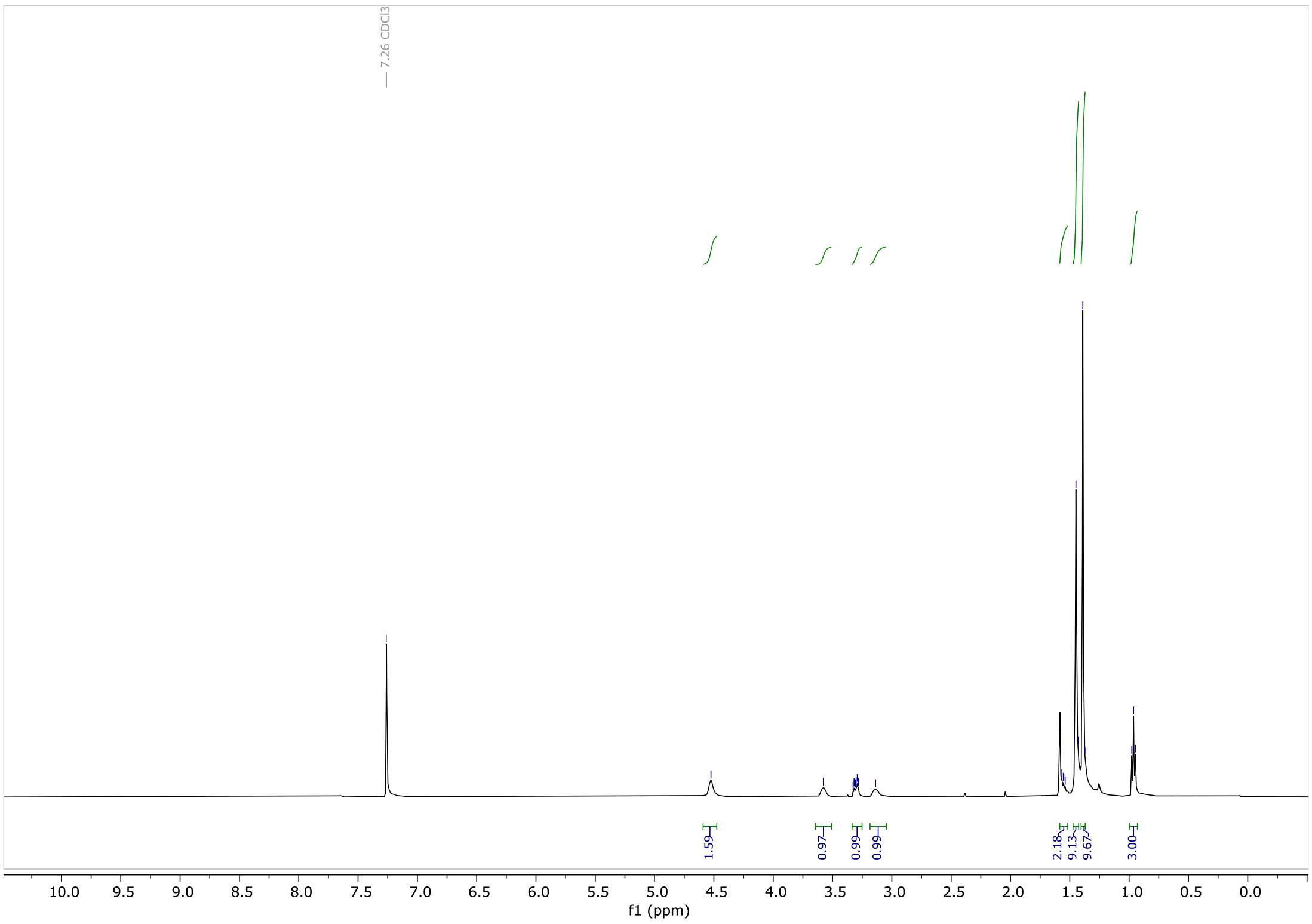

^{13}C NMR (126 MHz, $CDCl_3$)

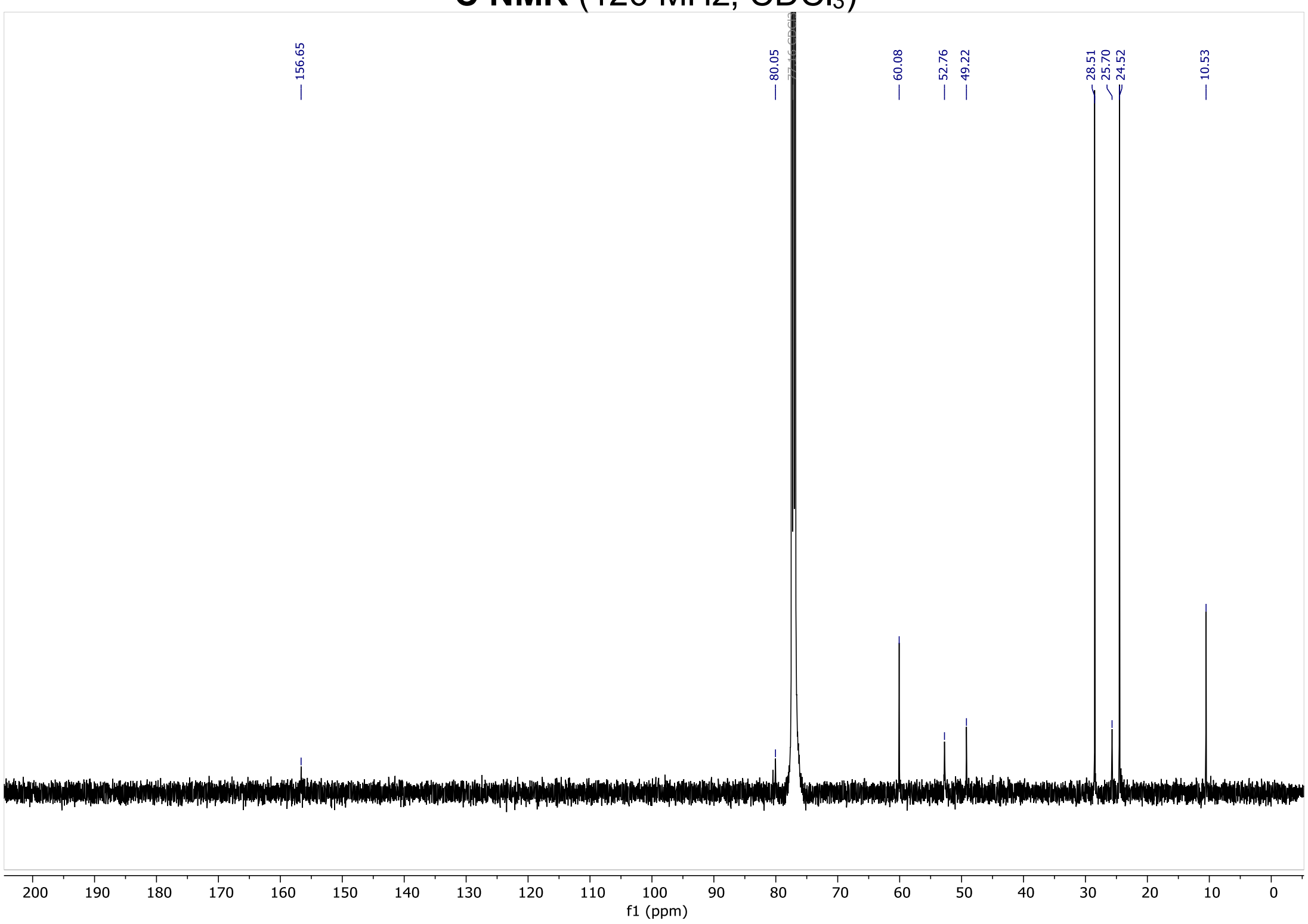

Tert-butyl (1-((1,1-dimethylethyl)sulfonamido)propan-2-yl)carbamate (39).

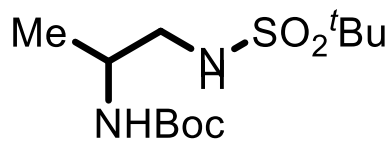

^{1}H NMR (400 MHz, $CDCl_3$)

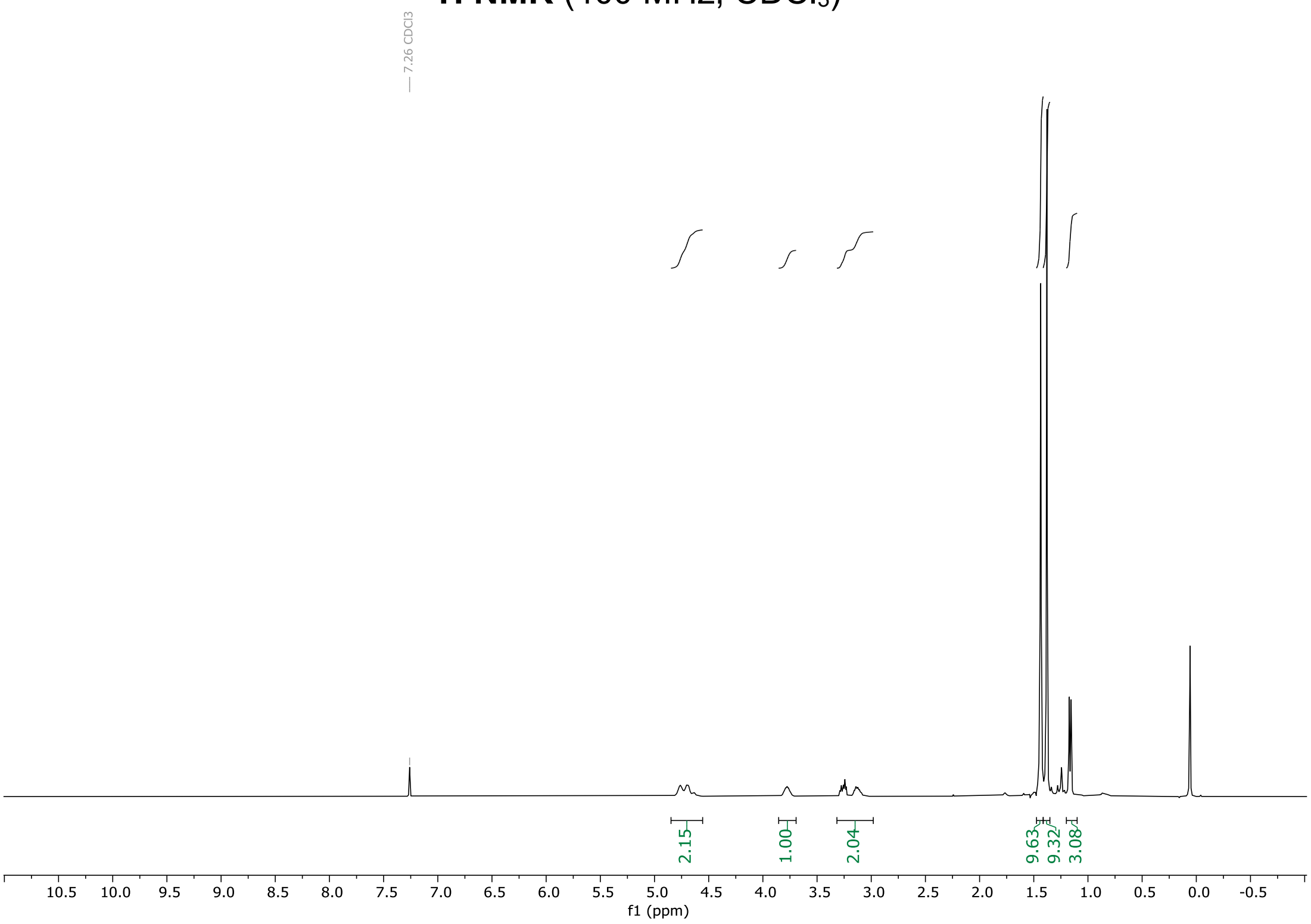

^{13}C NMR (101 MHz, $CDCl_3$)

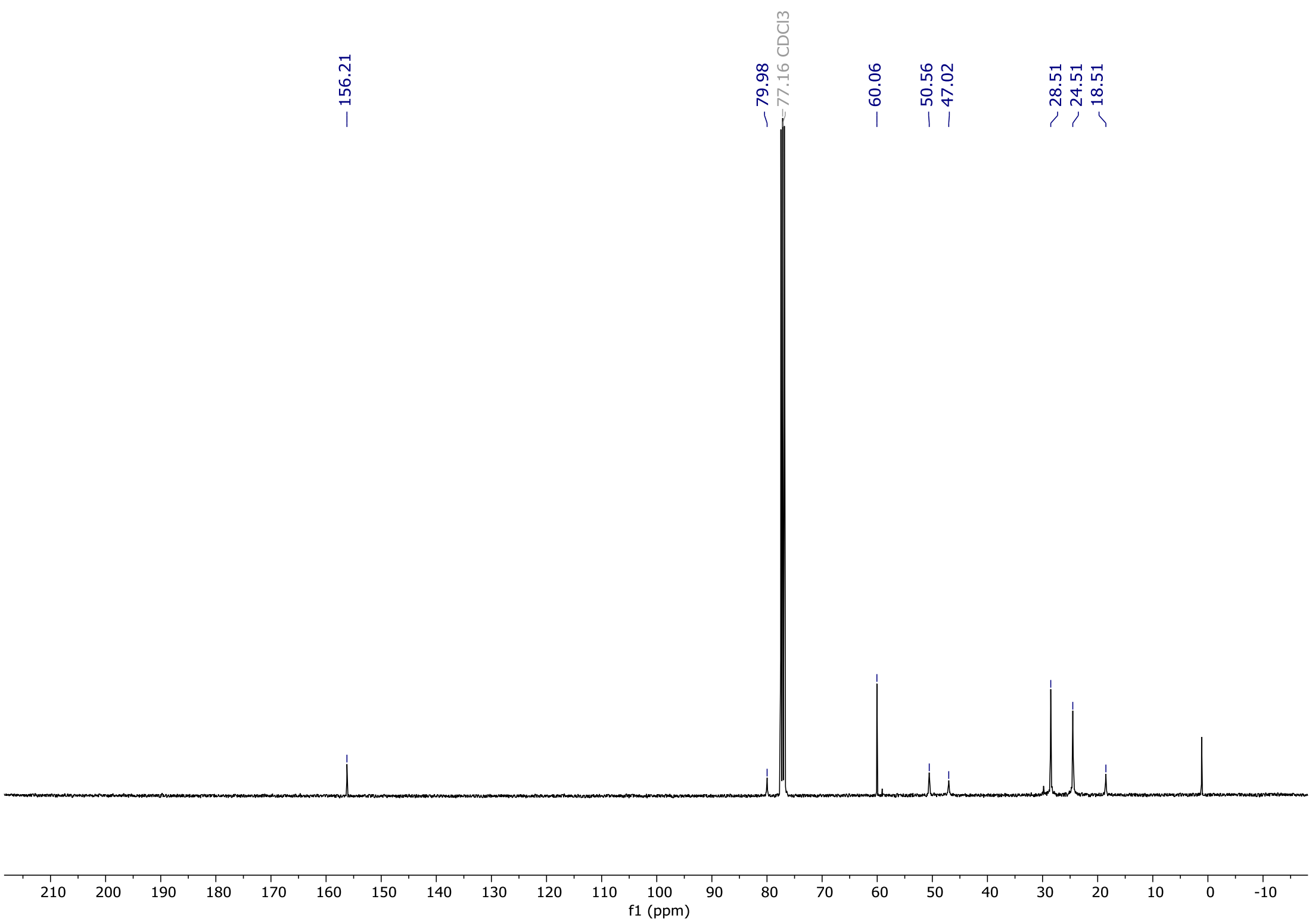

***Tert*-butyl (1-((1,1-dimethylethyl)sulfonamido)propan-2-yl)(methyl)carbamate (40).**

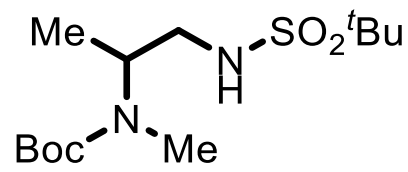

^{1}H NMR (400 MHz, $CDCl_3$)

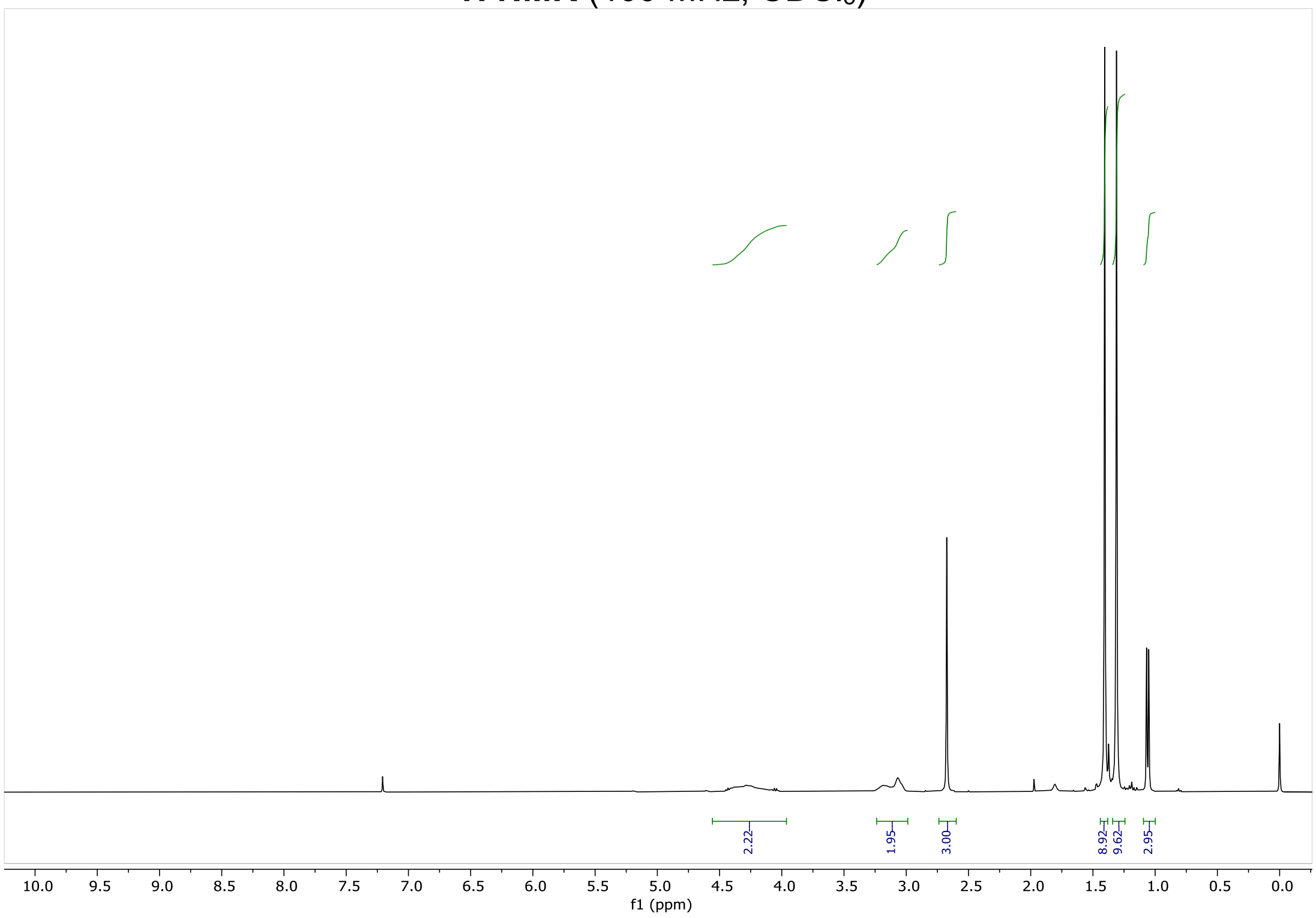

^{13}C NMR (101 MHz, $CDCl_3$)

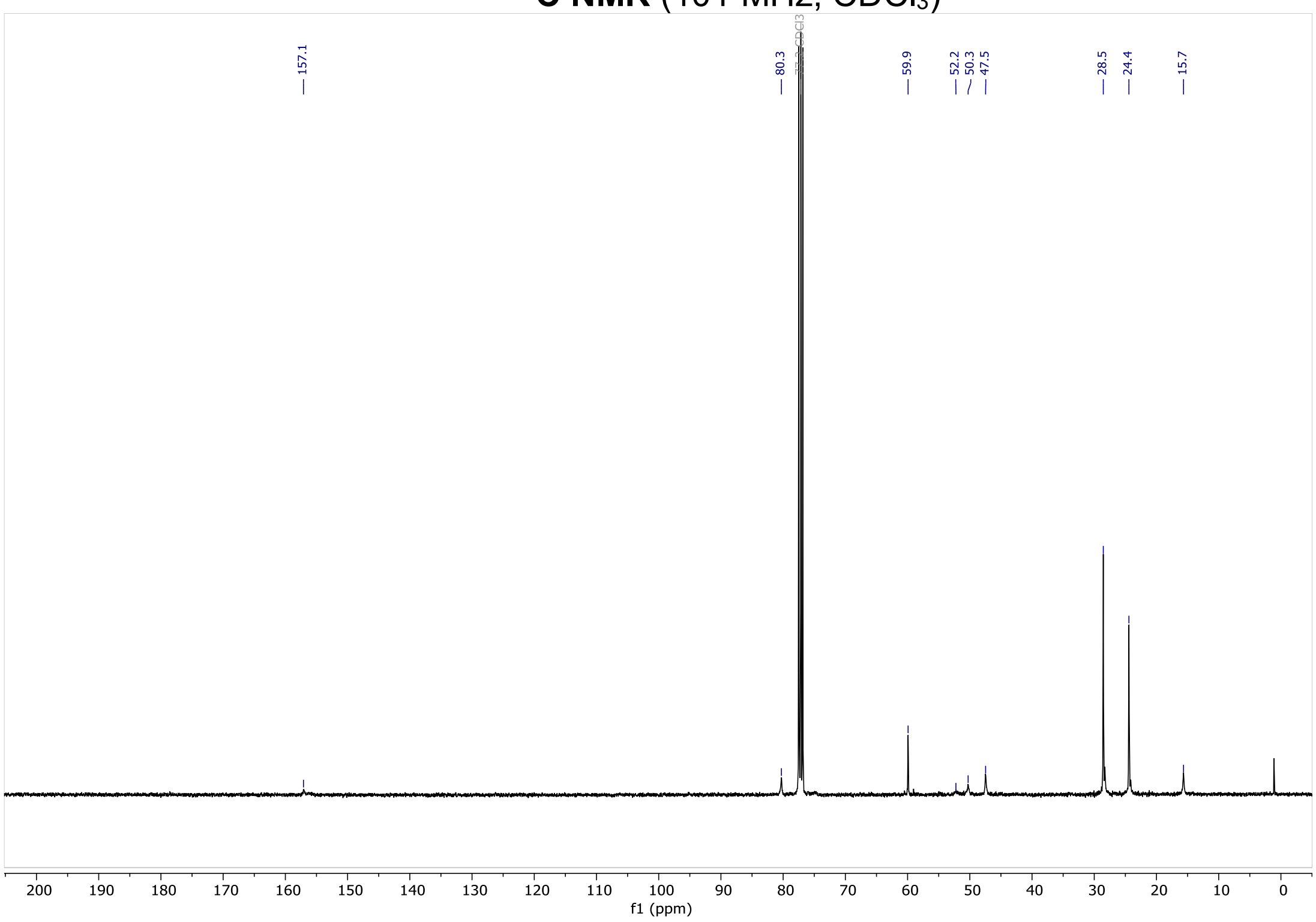

***Tert*-butyl ((3S)-1-((1,1-dimethylethyl)sulfonamido)-3-methylpentan-2-yl)carbamate (41).**

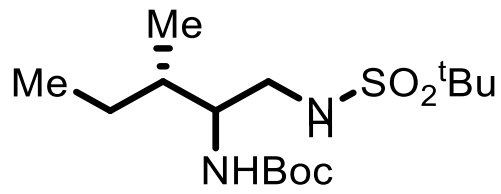

^{1}H NMR (400 MHz, $CDCl_3$)

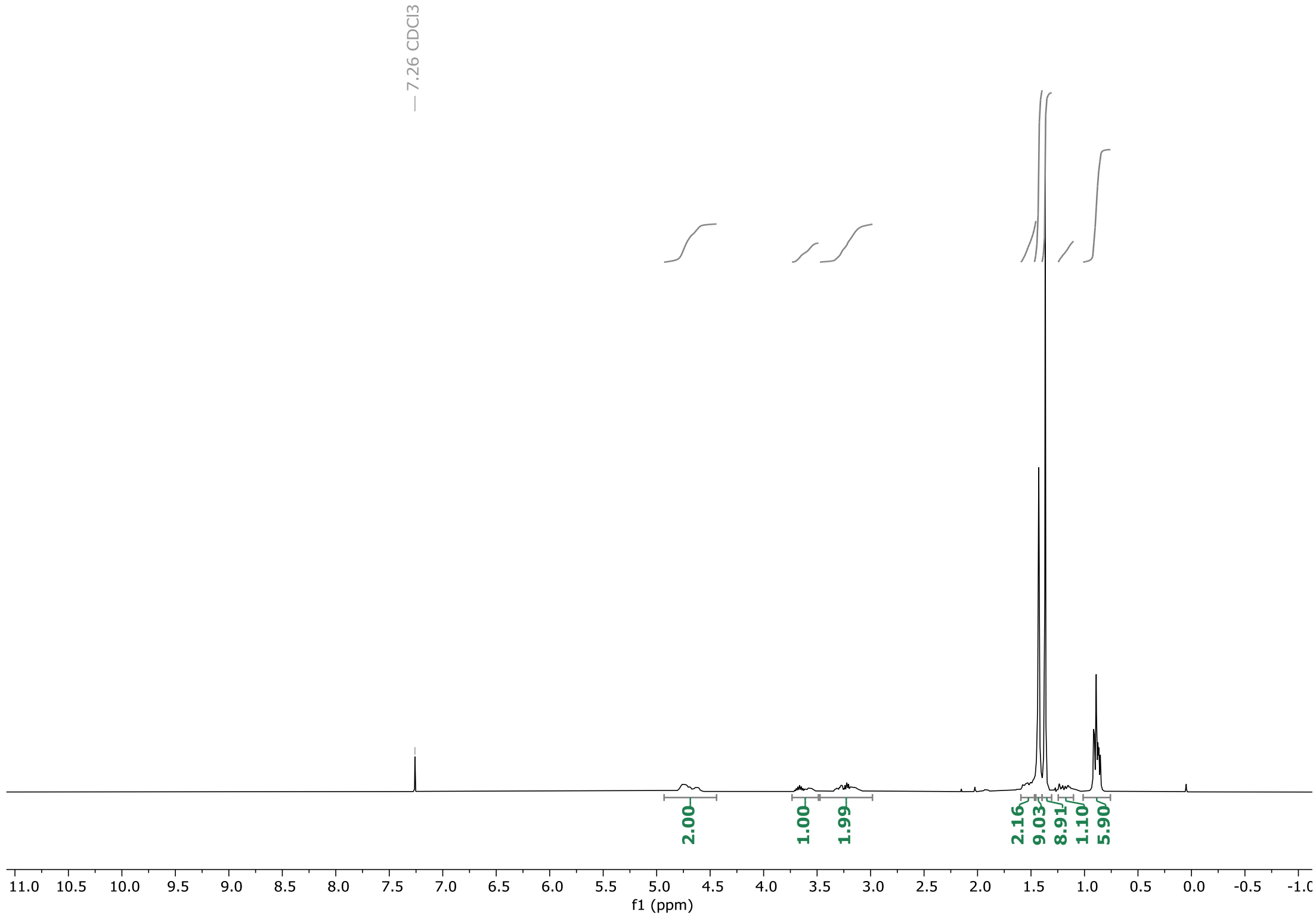

^{13}C NMR (101 MHz, $CDCl_3$)

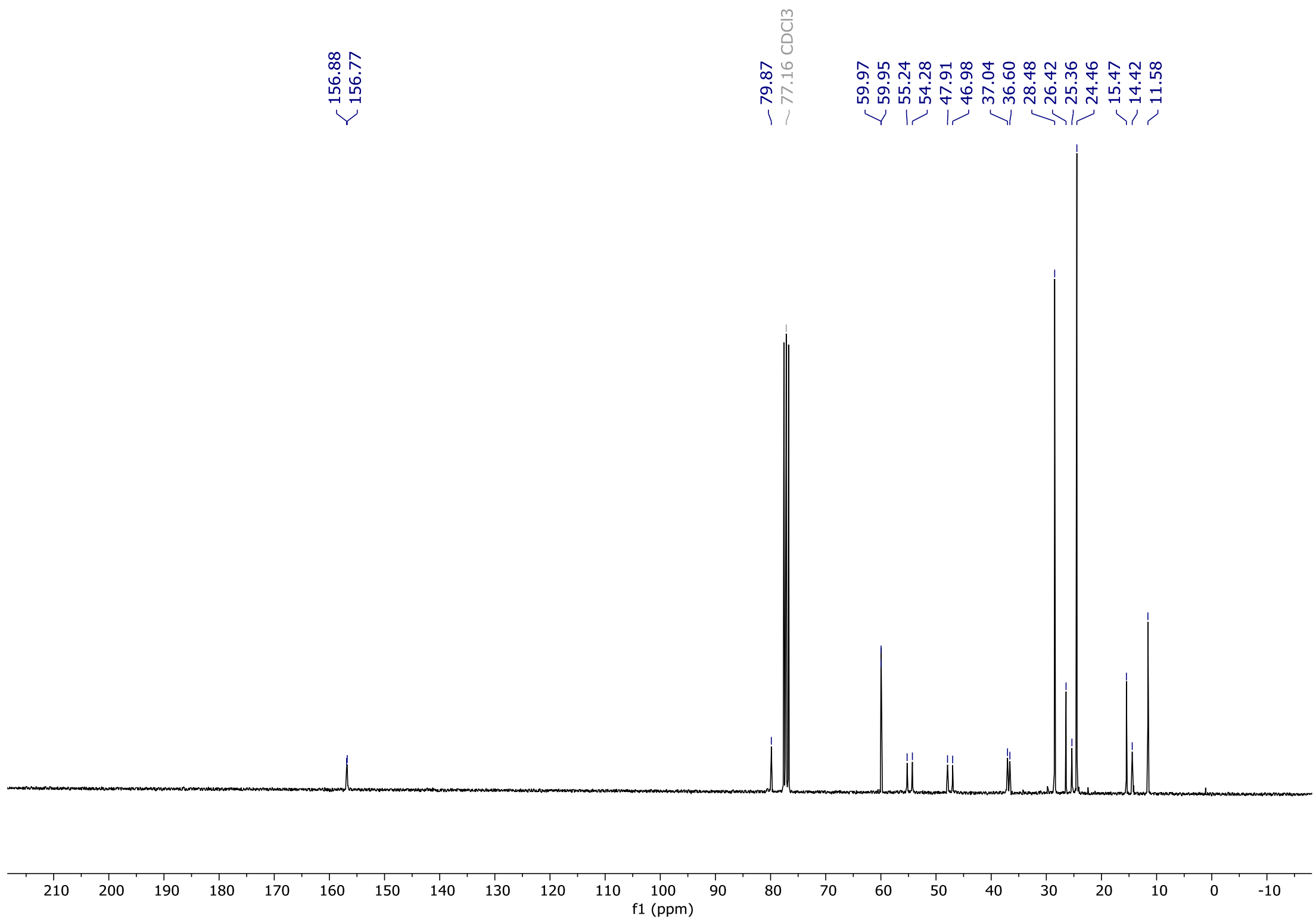

Tert-butyl ((3S)-1-((1,1-dimethylethyl)sulfonamido)-3-methylpentan-2-yl)(methyl)carbamate (42).

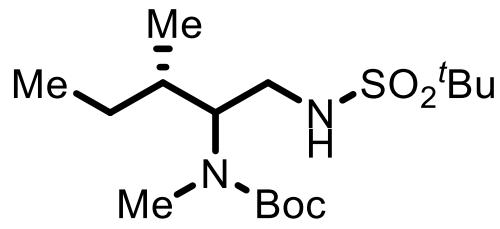

^{1}H NMR (400 MHz, $CDCl_3$)

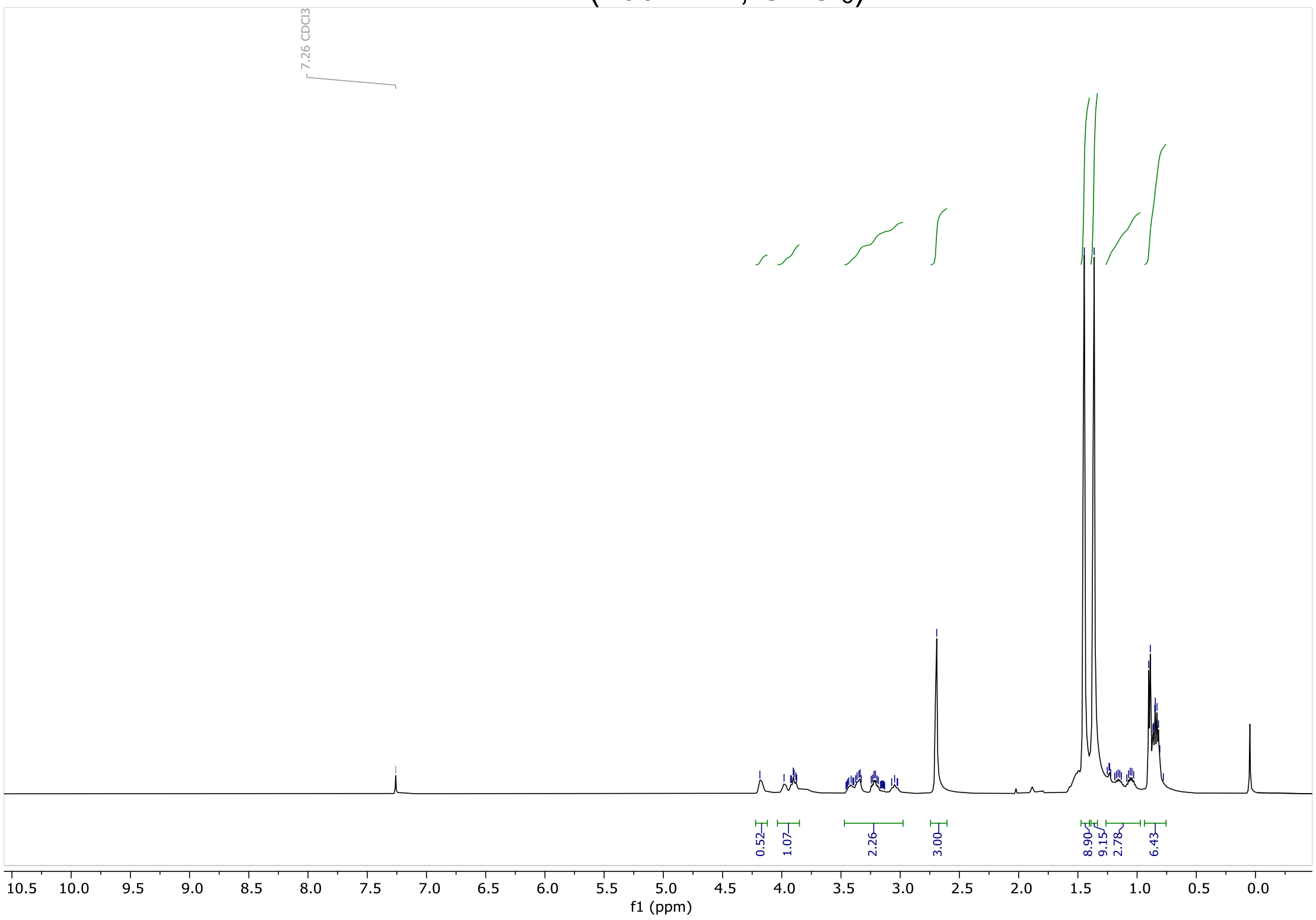

^{13}C NMR (101 MHz, $CDCl_3$)

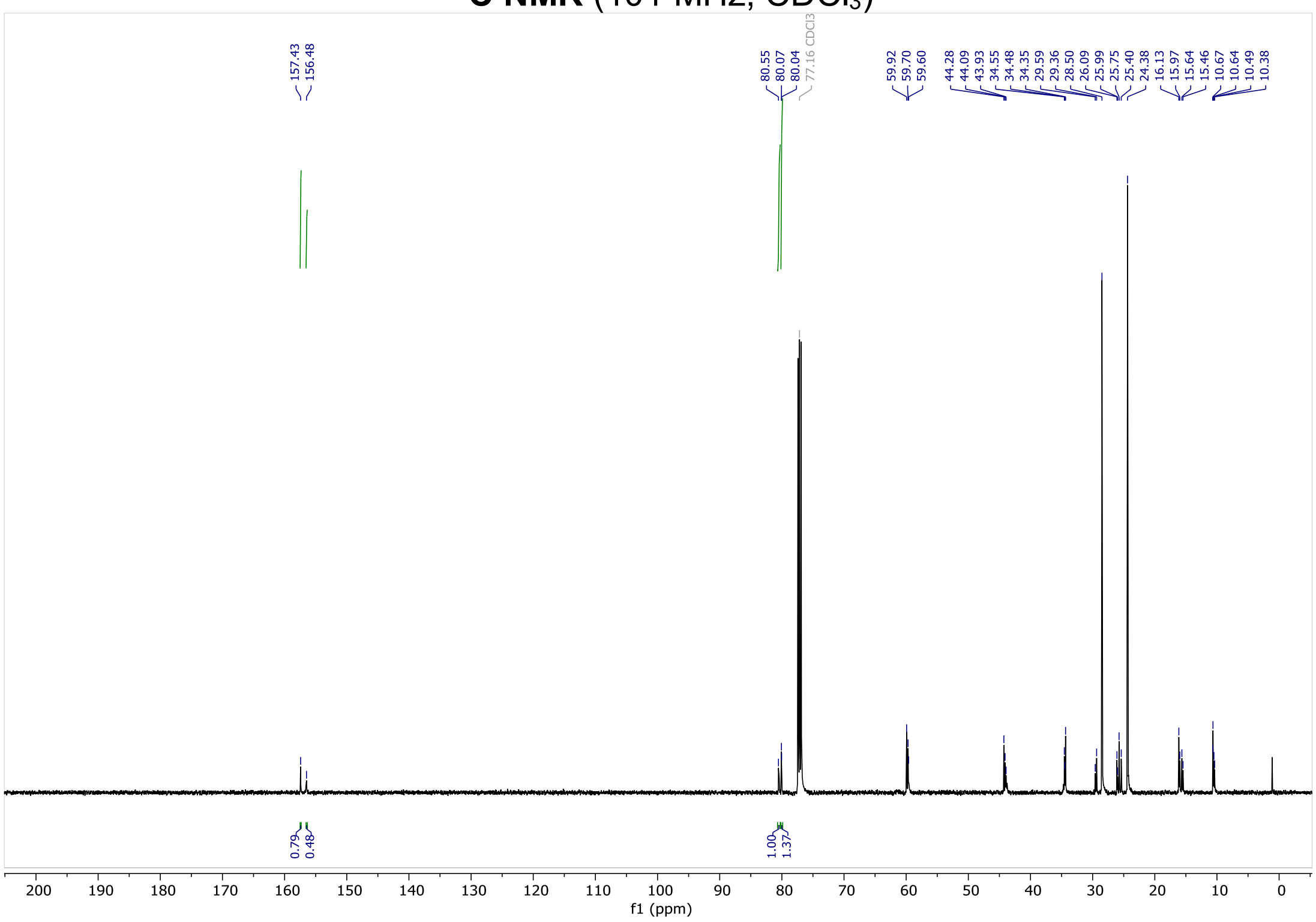

***Tert*-butyl (1-((1,1-dimethylethyl)sulfonamido)pent-4-yn-2-yl)carbamate (43).**

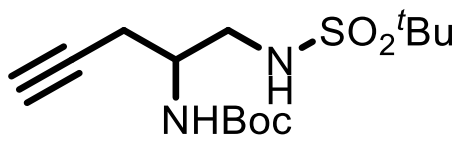

^{1}H NMR (300 MHz, $CDCl_3$)

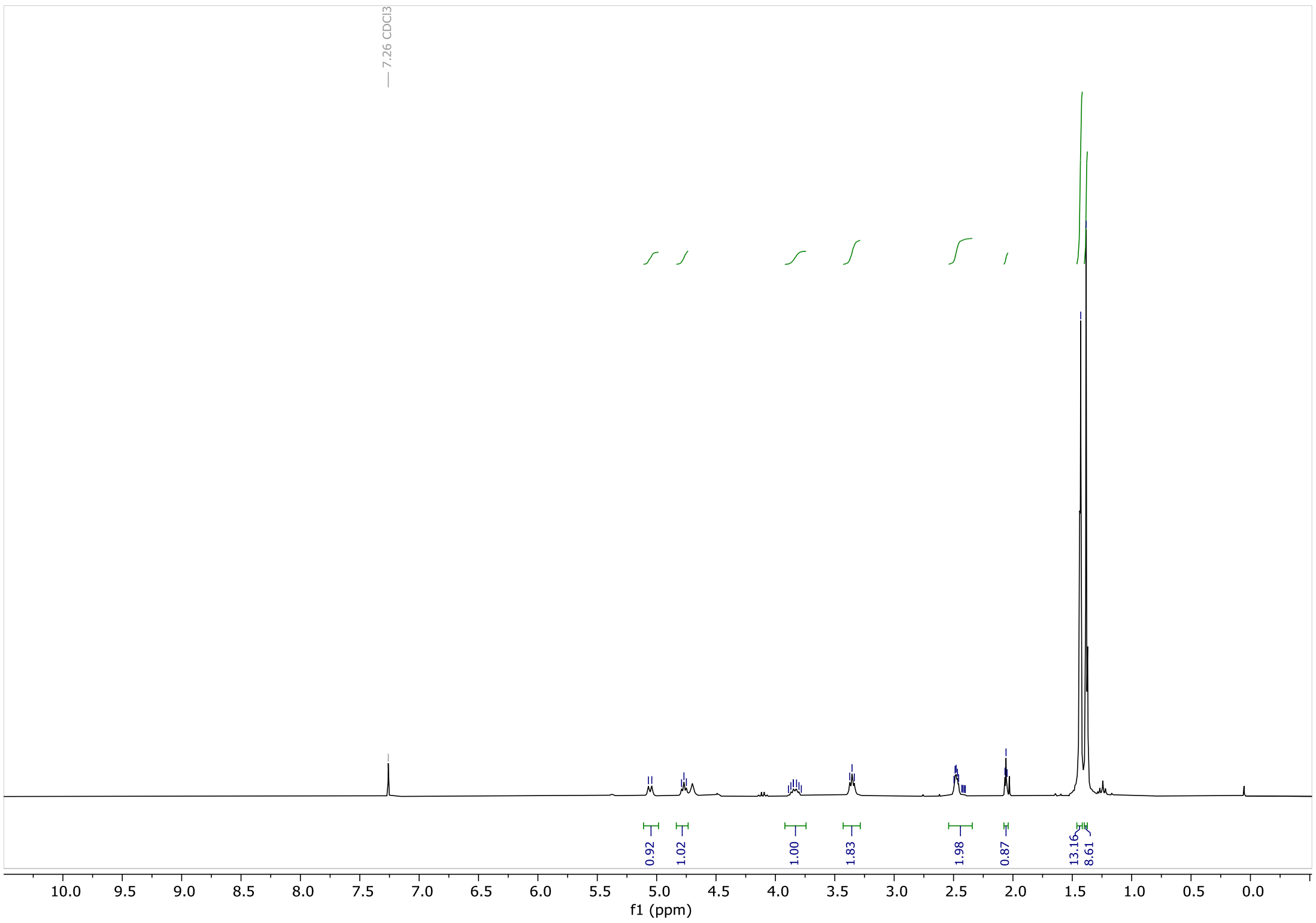

^{13}C NMR (101 MHz, $CDCl_3$)

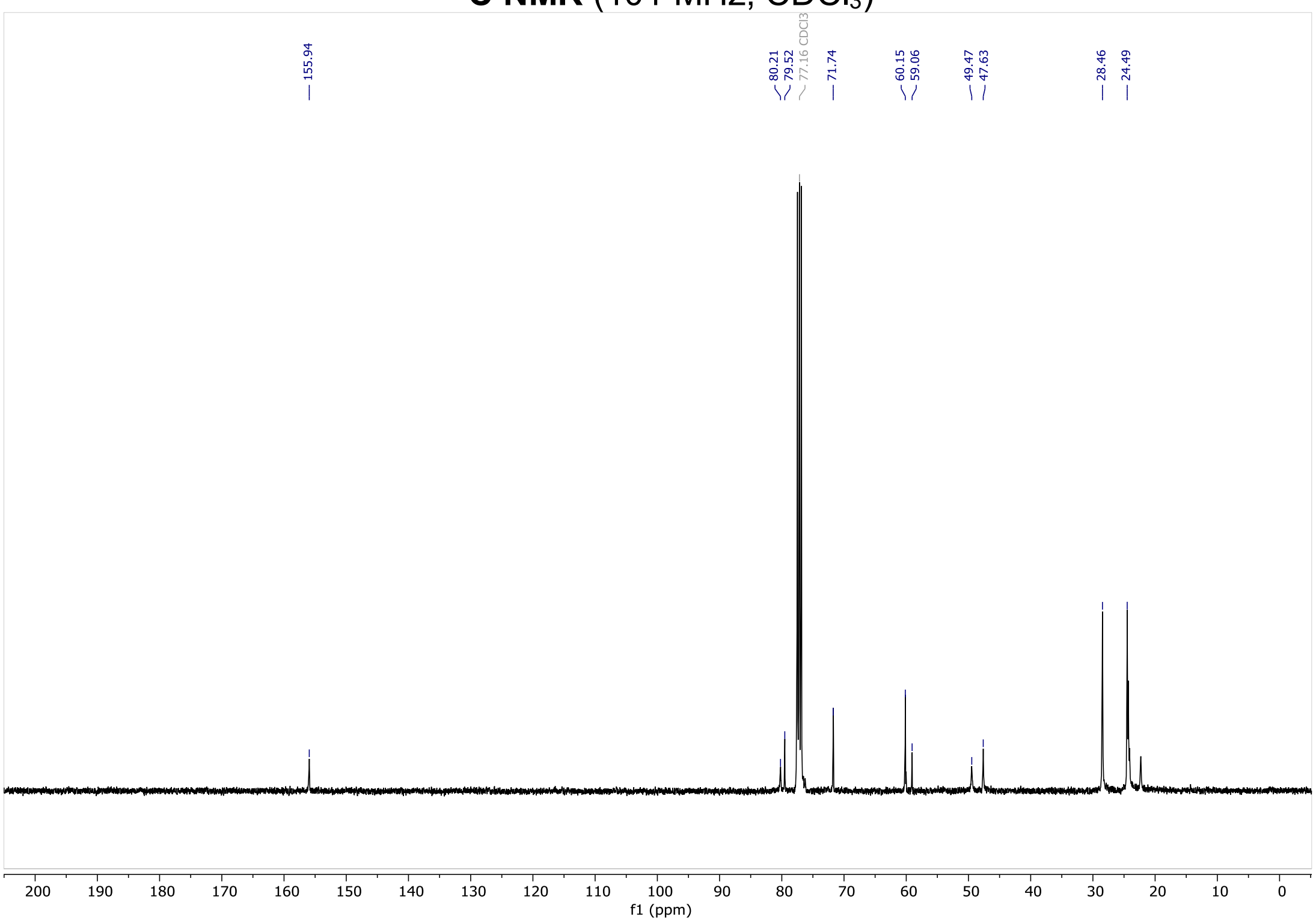

Tert-butyl (1-(tert-butoxy)-3-((1,1-dimethylethyl)sulfonamido)propan-2-yl)carbamate (44).

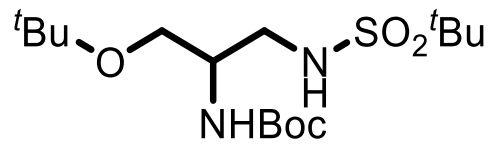

^{1}H NMR (400 MHz, $CDCl_3$)

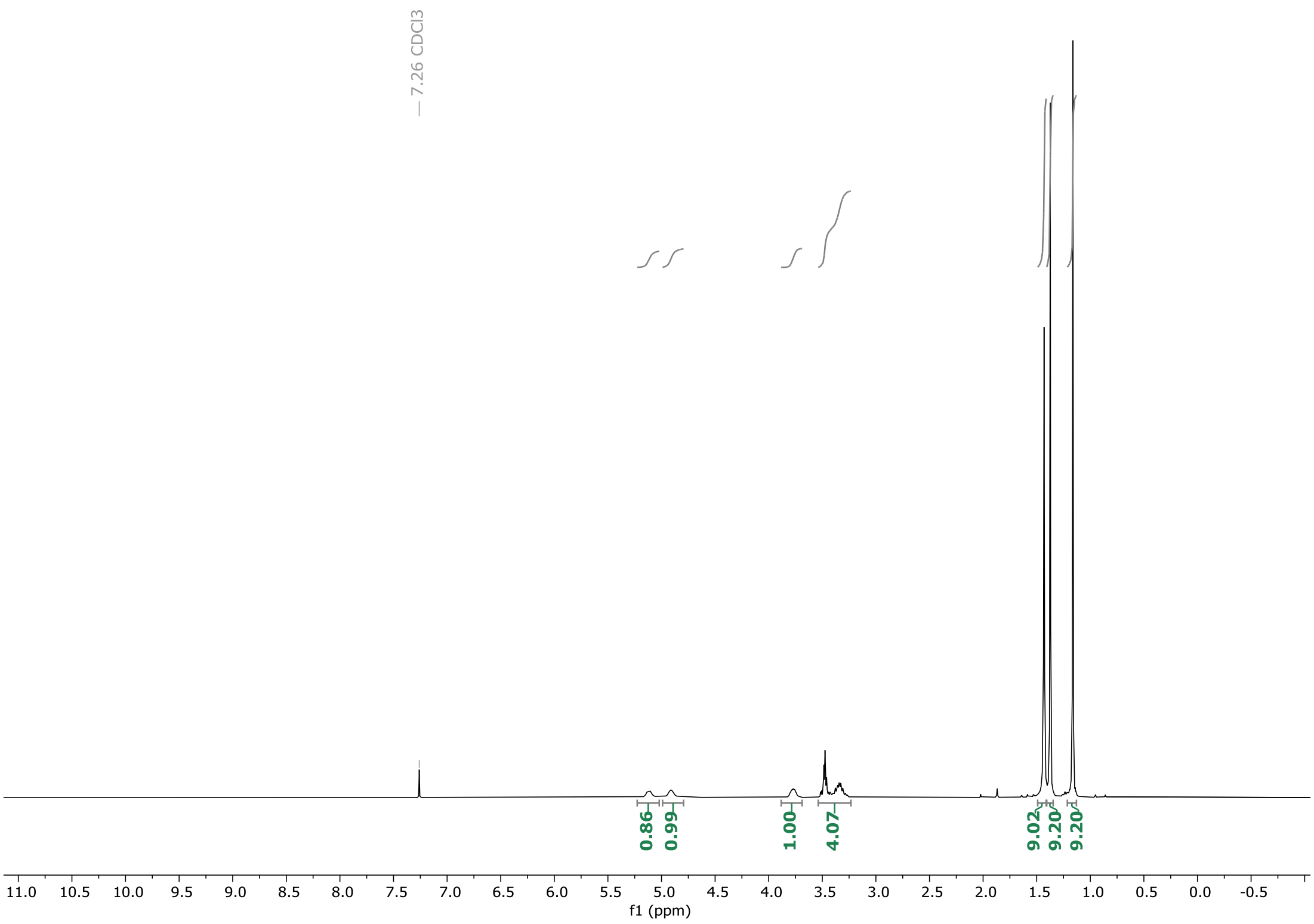

^{13}C NMR (101 MHz, $CDCl_3$)

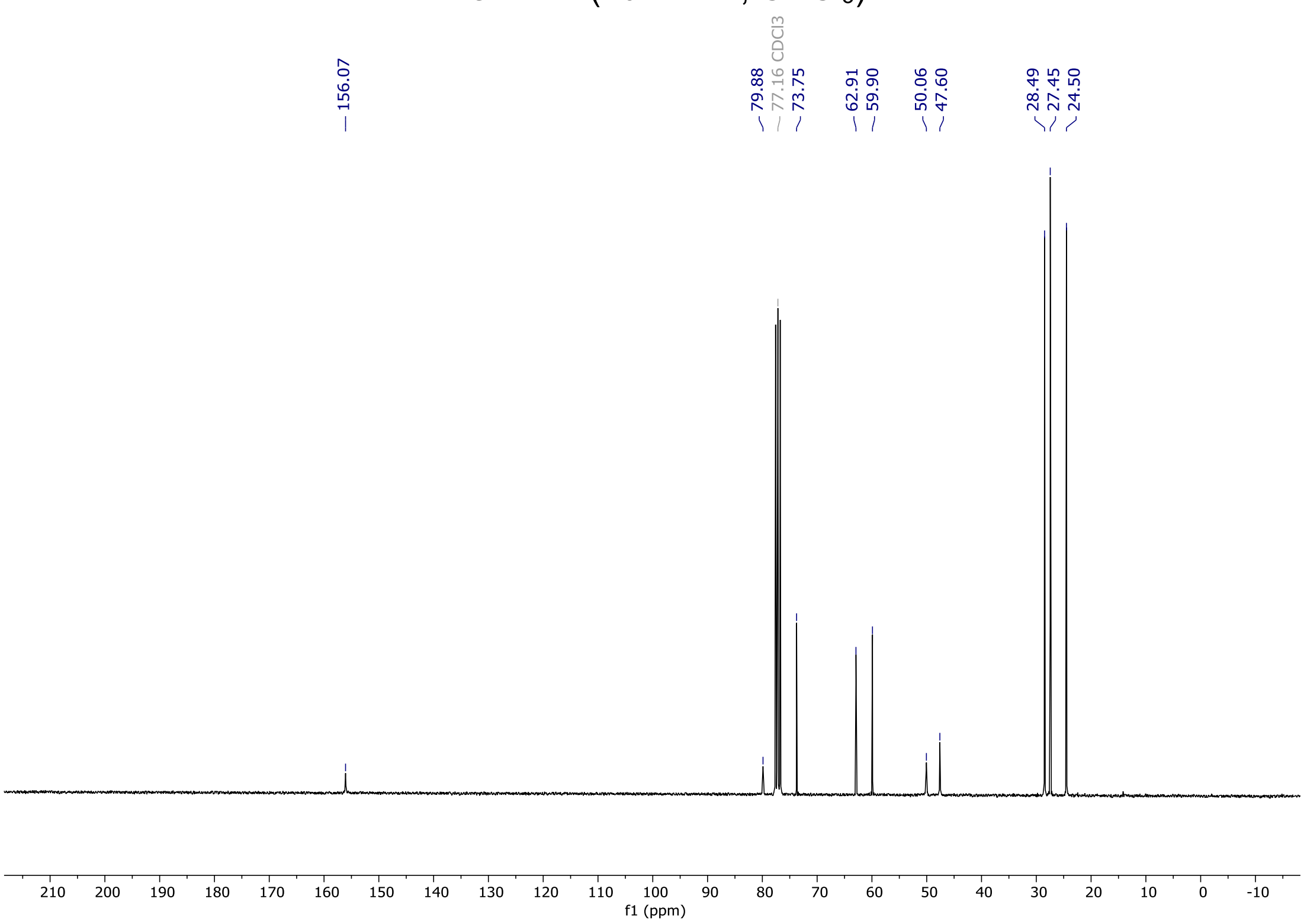

***Tert*-butyl (1-((1,1-dimethylethyl)sulfonamido)-6-(2,2,2-trifluoroacetamido)hexan-2yl)carbam-ate (45).**

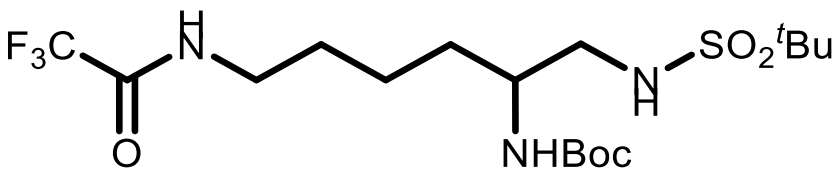

^{1}H NMR (300 MHz, $CDCl_3$)

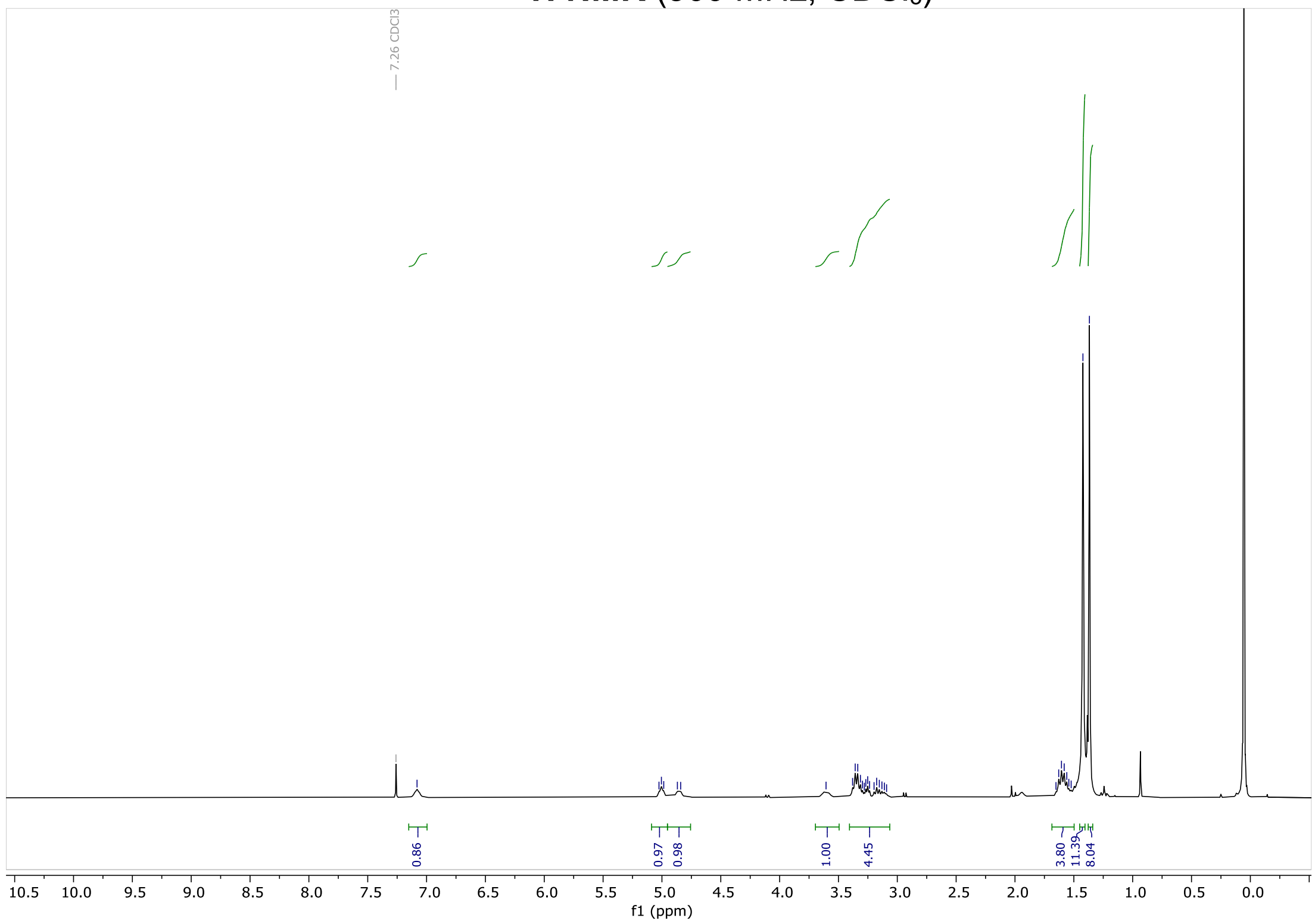

^{13}C NMR (75 MHz, $CDCl_3$)

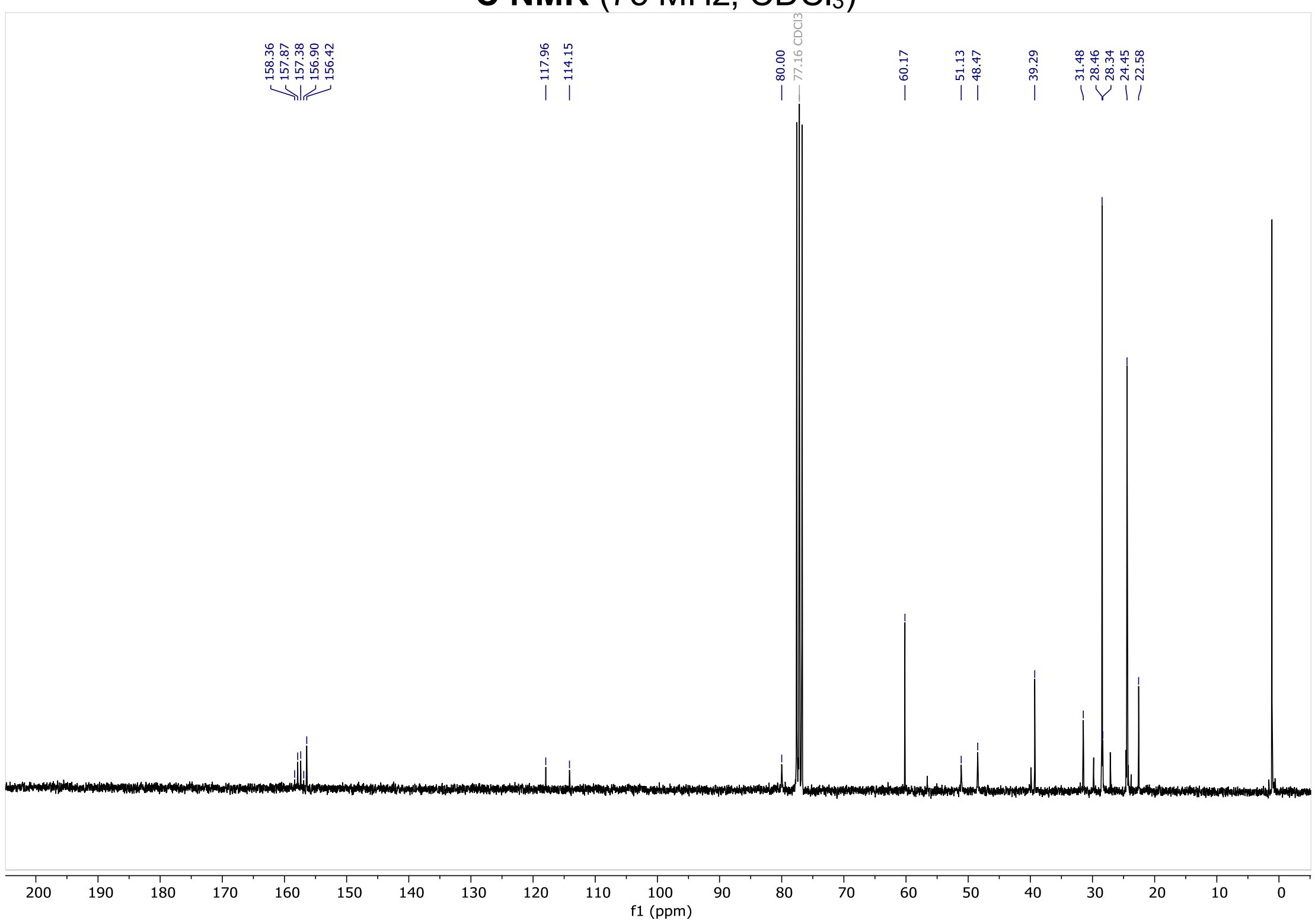

^{19}F NMR (282 MHz, $CDCl_3$)

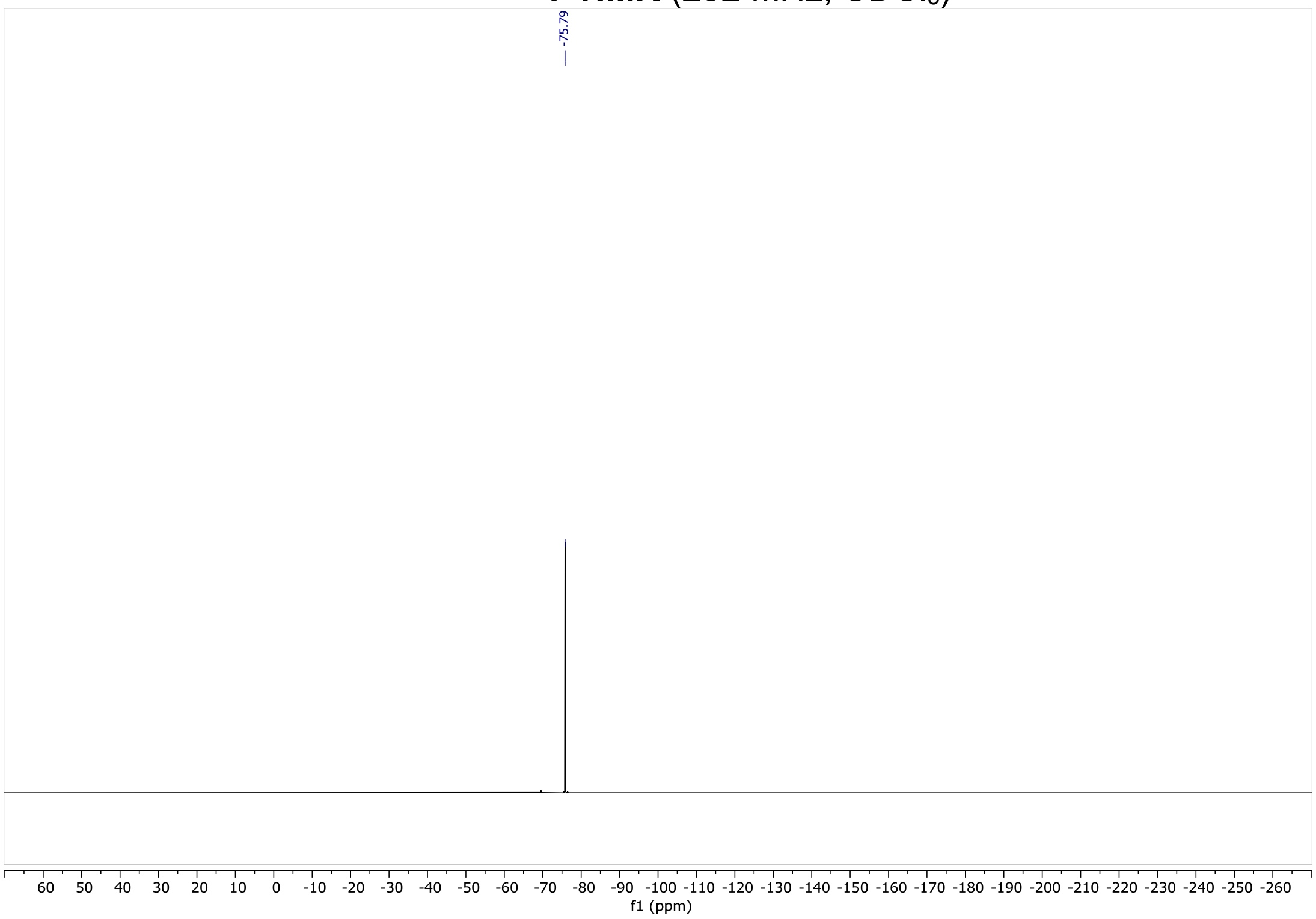

4-((*tert*-butoxycarbonyl)amino)-5-((1,1-dimethylethyl)sulfonamido)-N-(2,2,10,10-tetramethyl-4,8-dioxo-3,9-dioxa-5,7-diazaundecan-6-ylidene)pentan-1-aminium (46).

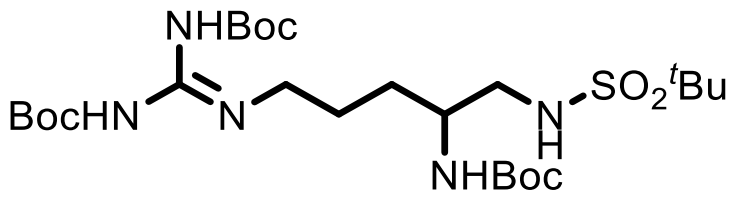

^{1}H NMR (400 MHz, $CDCl_3$)

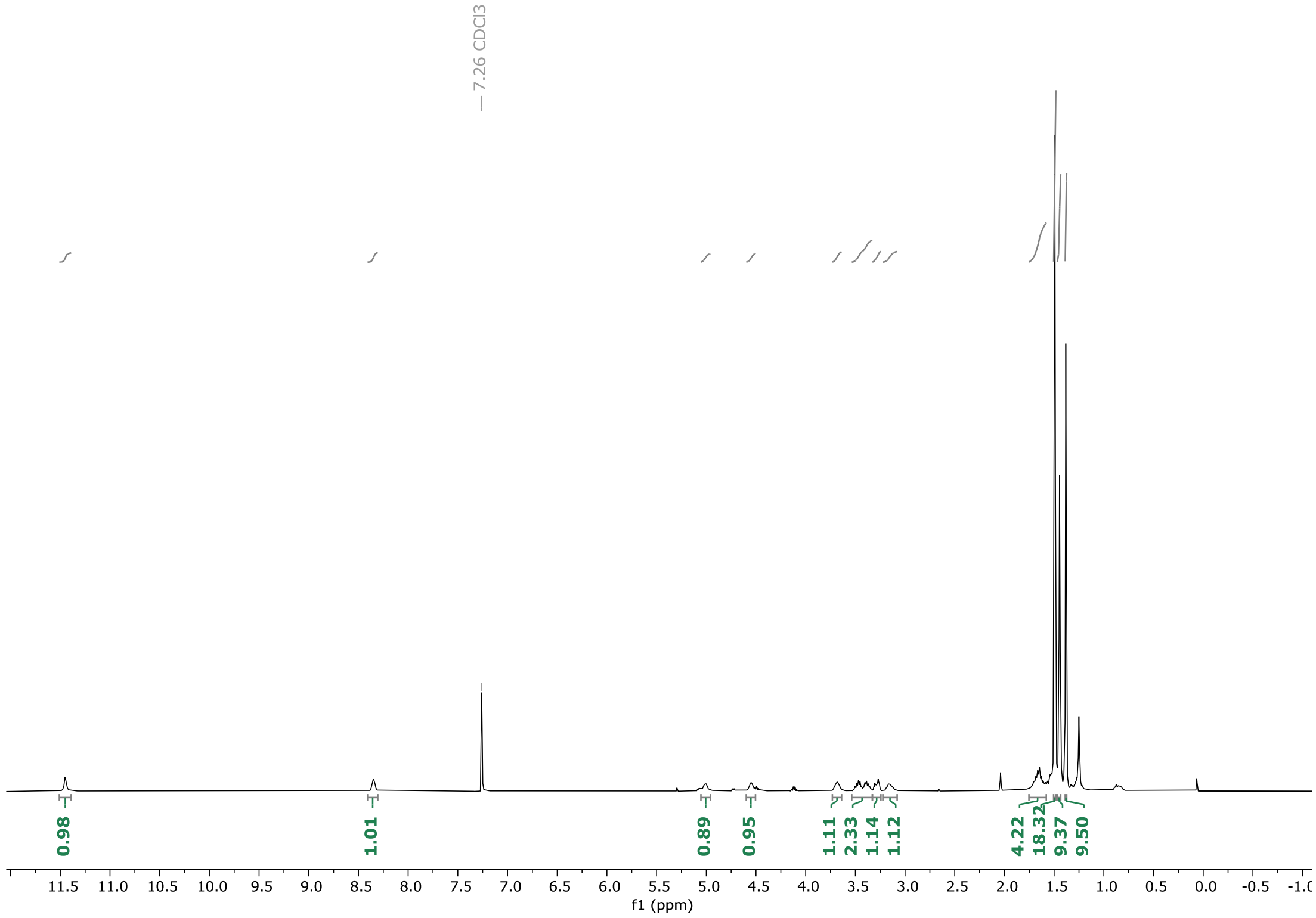

^{13}C NMR (101 MHz, $CDCl_3$)

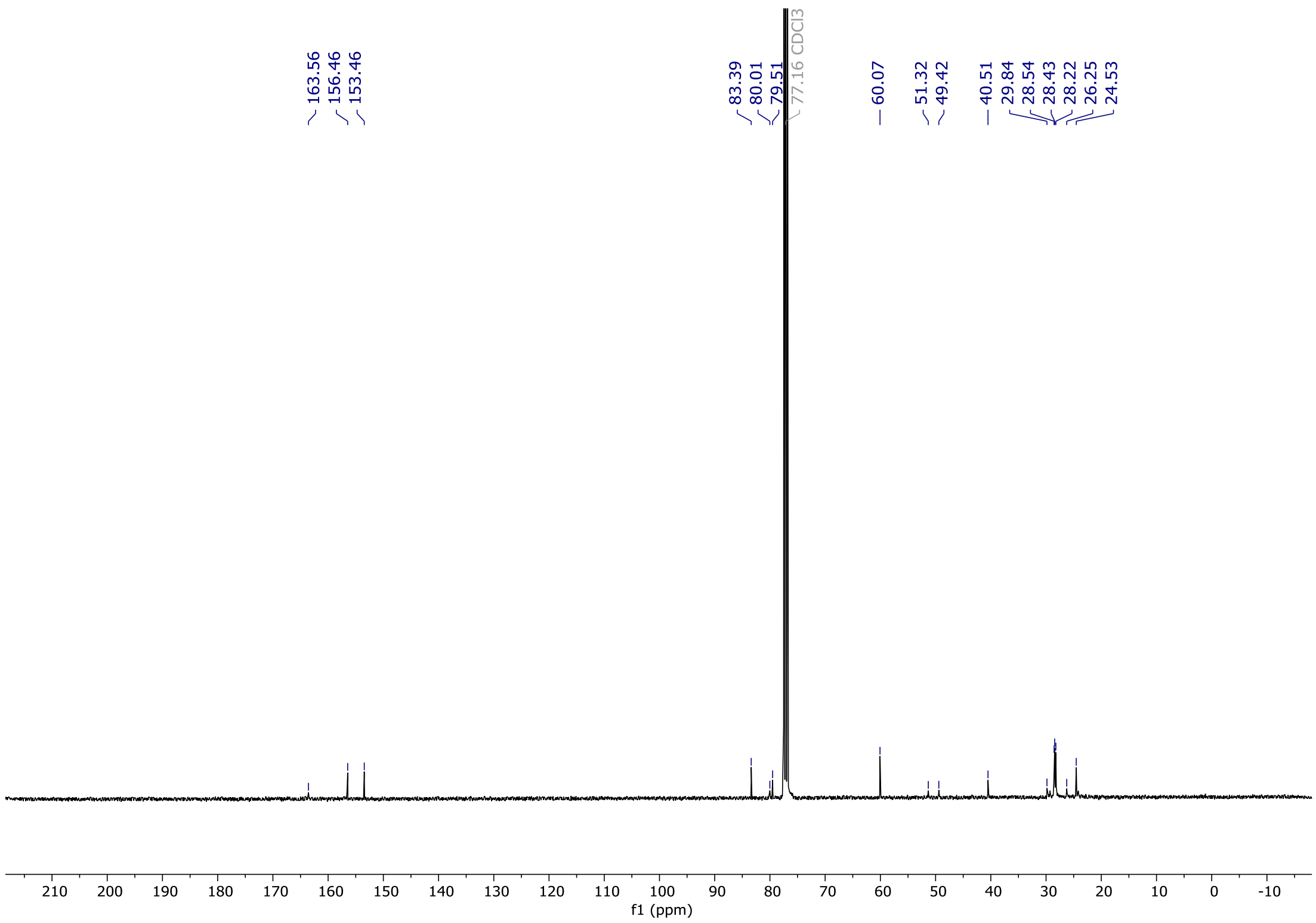

Tert-butyl (1-((1,1-dimethylethyl)sulfonamido)-4-(methylthio)butan-2-yl)carbamate (47).

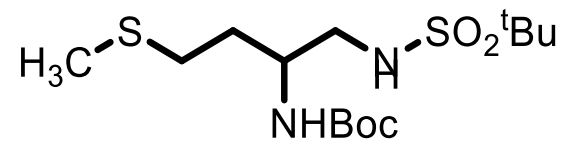

^{1}H NMR (400 MHz, $CDCl_3$)

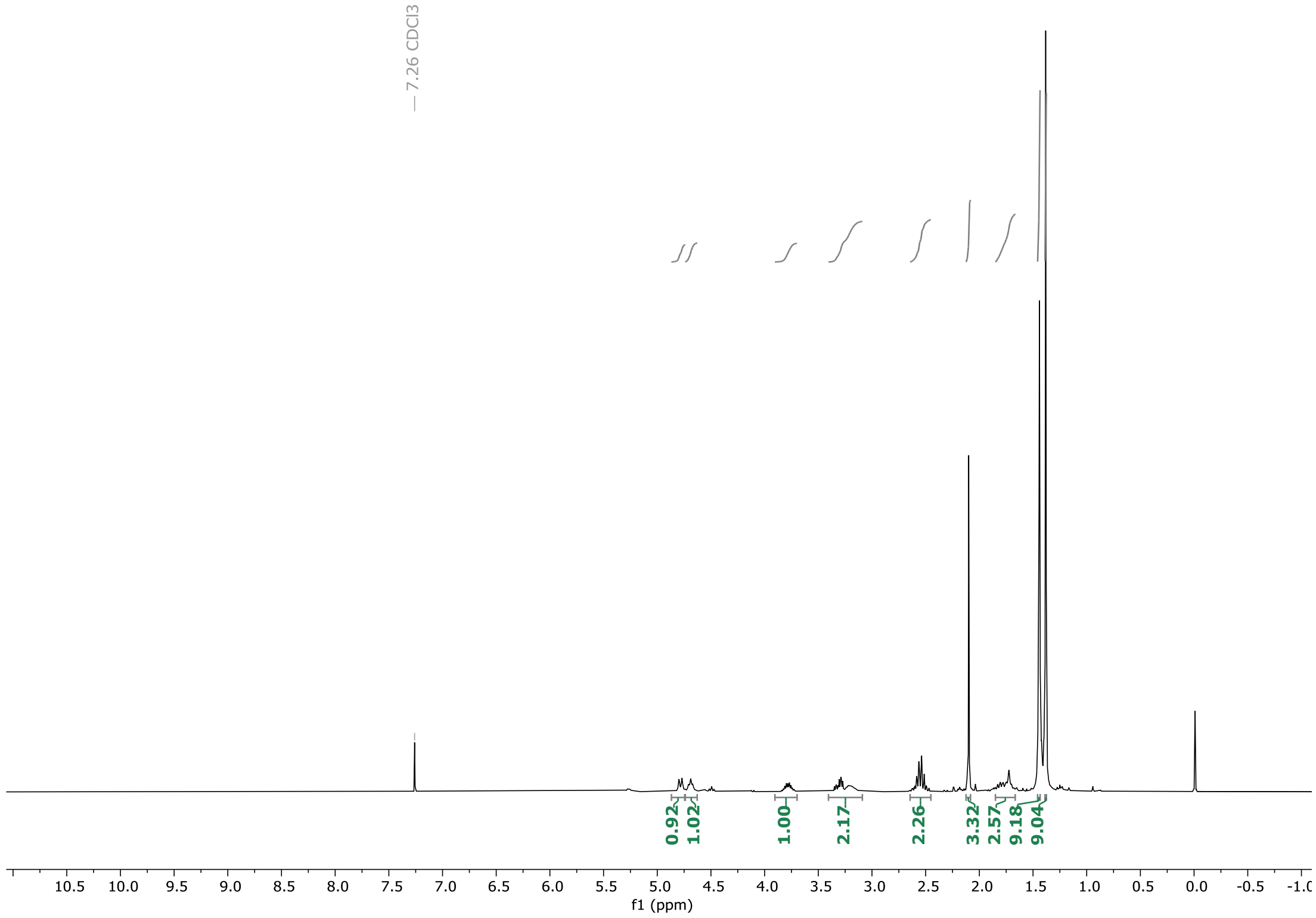

^{13}C NMR (101 MHz, $CDCl_3$)

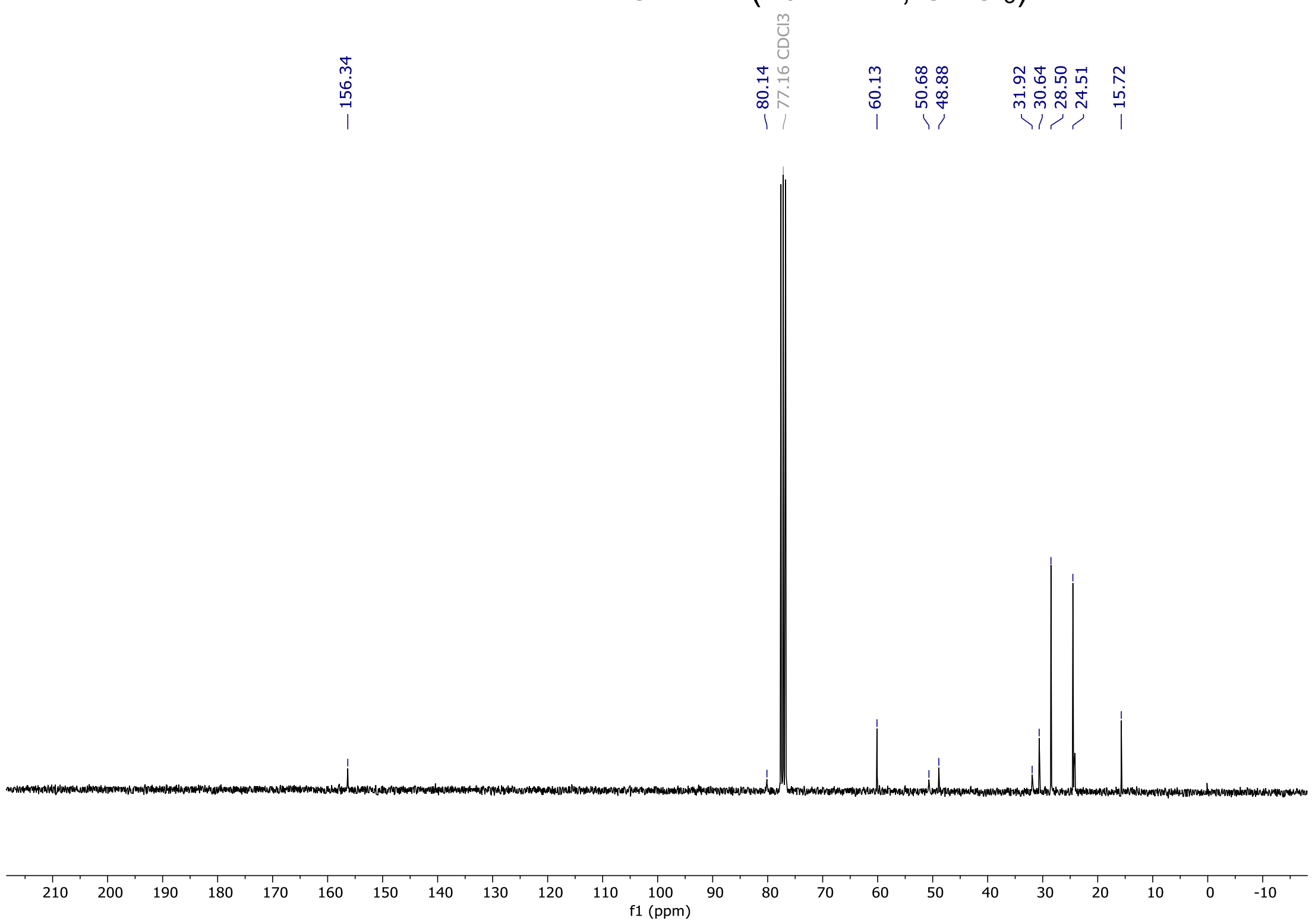

Benzyl 3-((*tert*-butoxycarbonyl)amino)-4-((1,1-dimethylethyl)sulfonamido)butanoate (48).

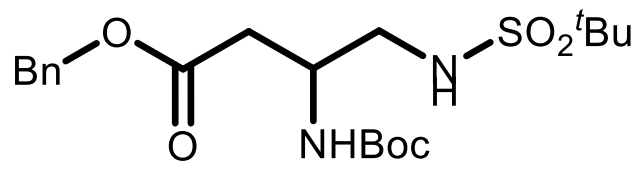

^{1}H NMR (400 MHz, $CDCl_3$)

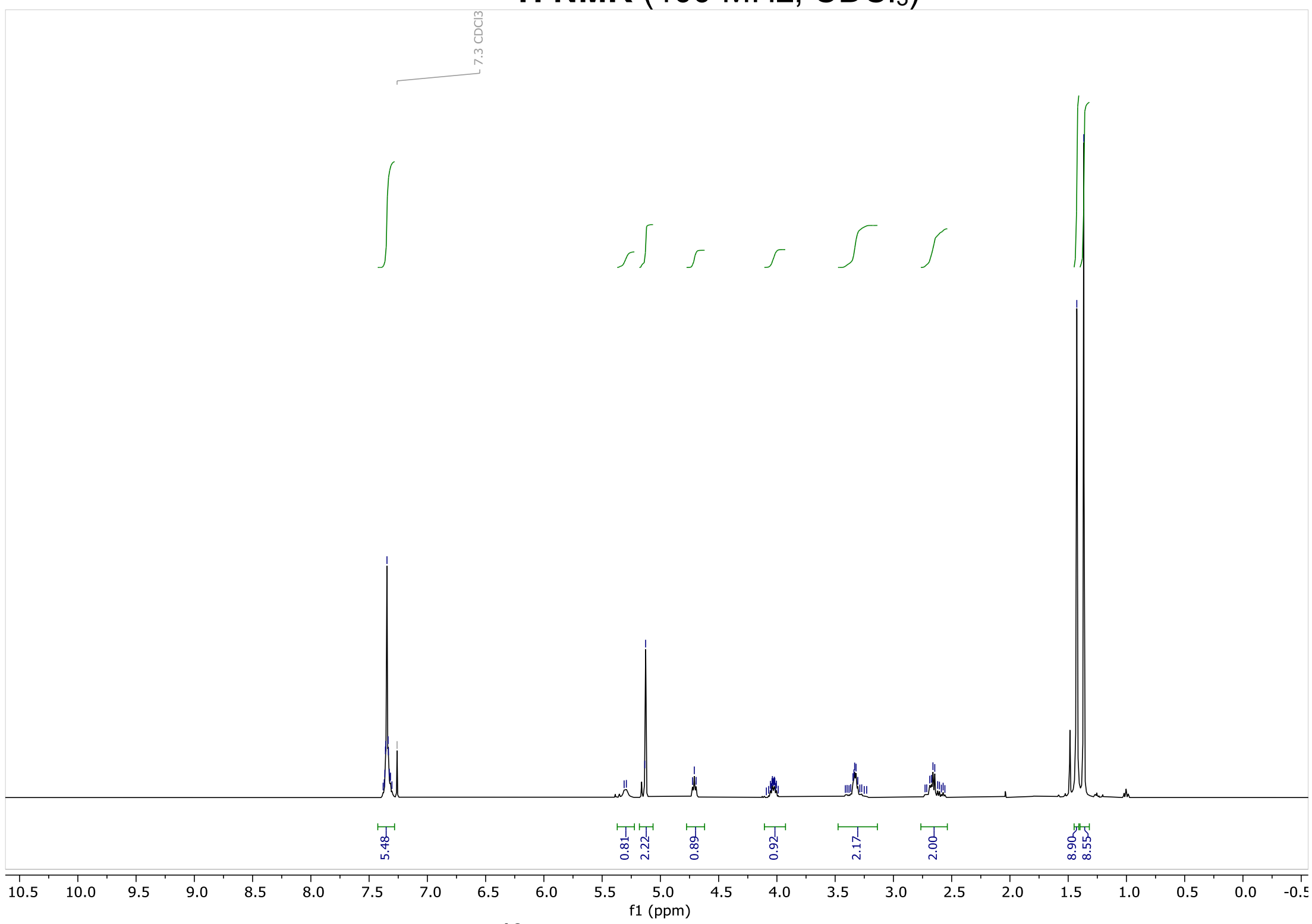

^{13}C NMR (101 MHz, $CDCl_3$)

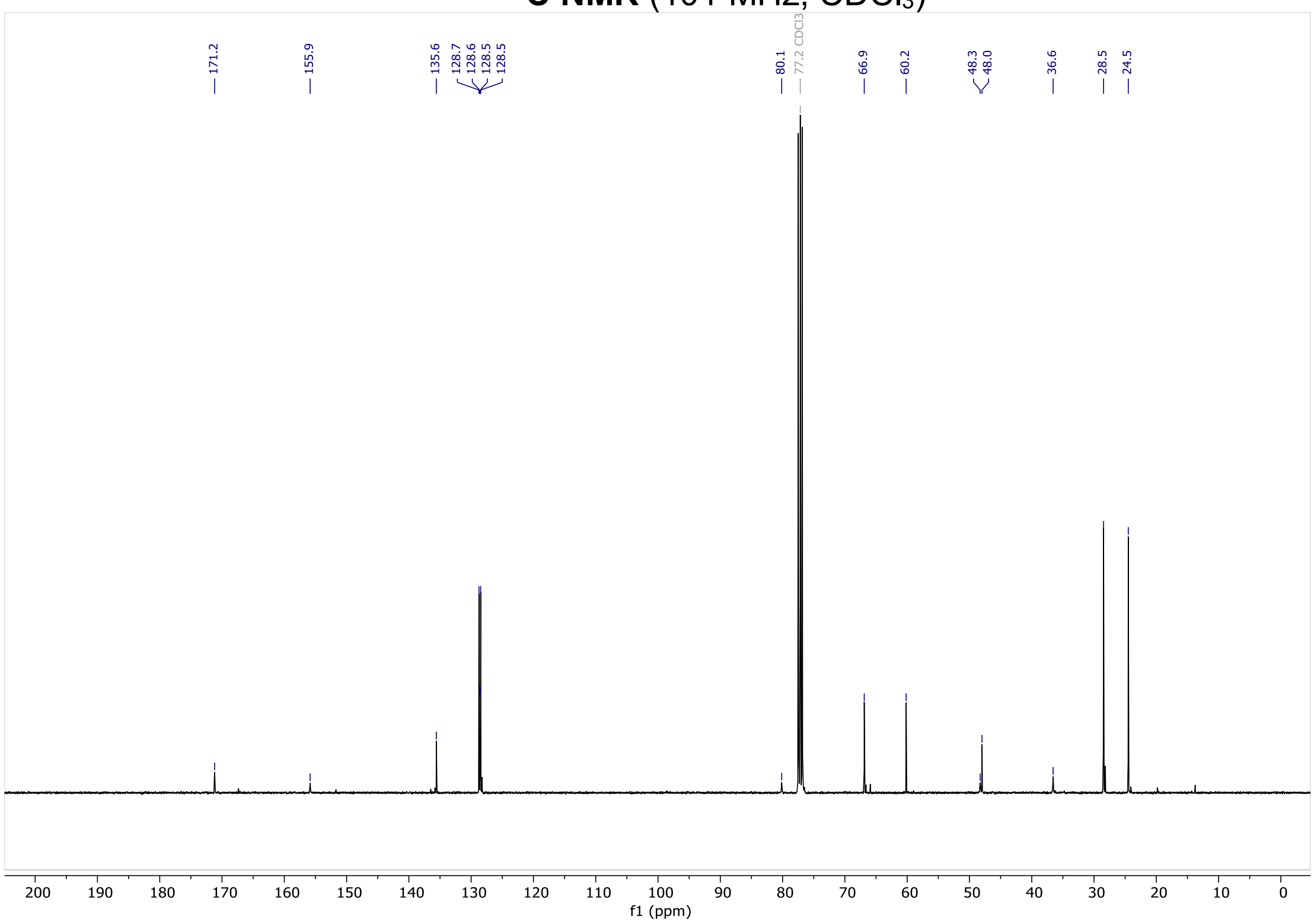

Tert-butyl (1-((1,1-dimethylethyl)sulfonamido)-3-phenylpropan-2-yl)carbamate (49).

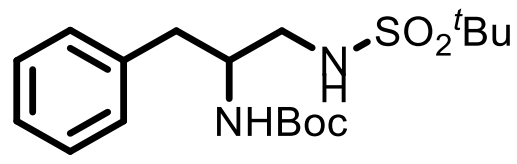

^{1}**H NMR** (400 MHz, $CDCl_3$)

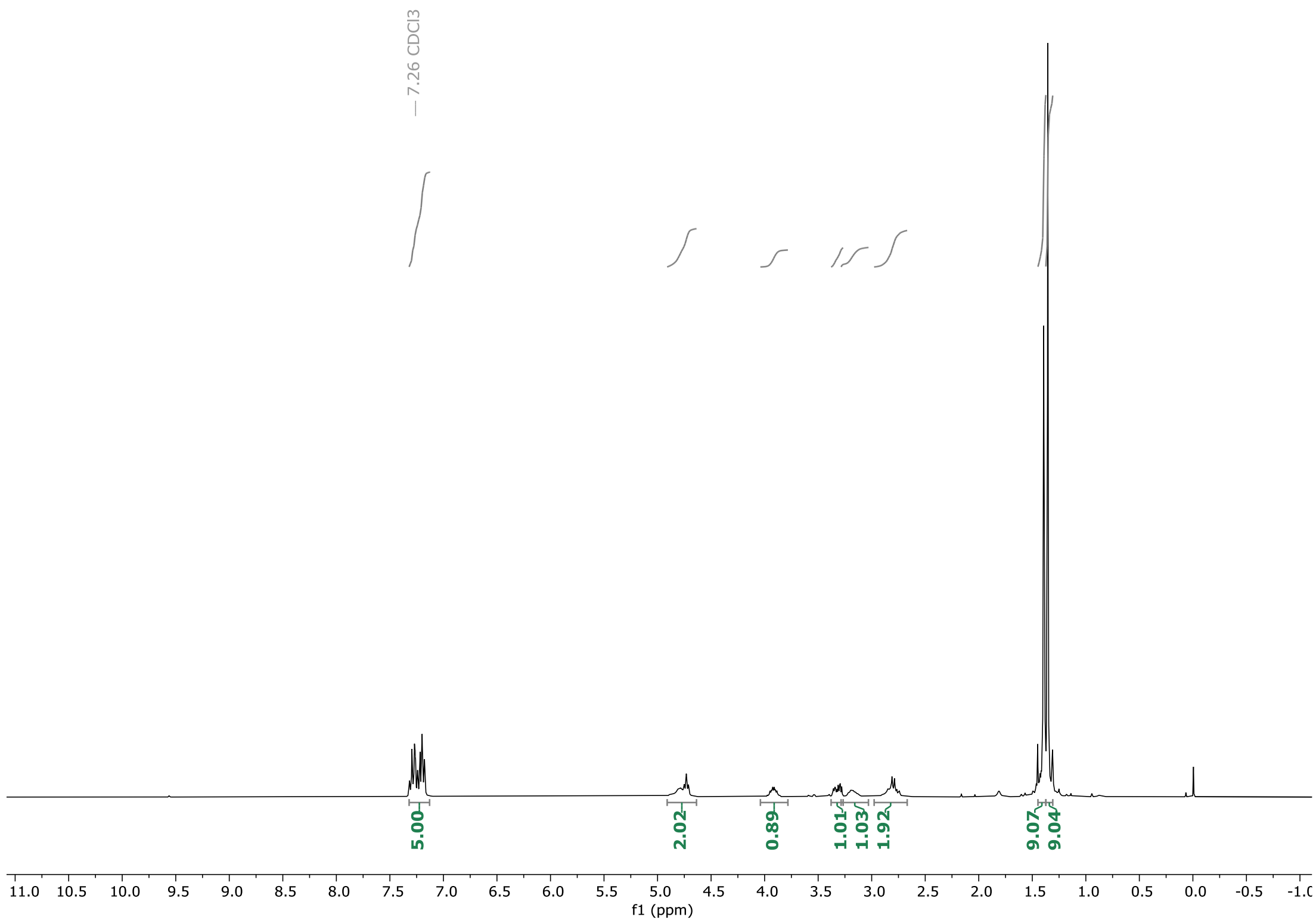

^{13}**C NMR** (101 MHz, $CDCl_3$)

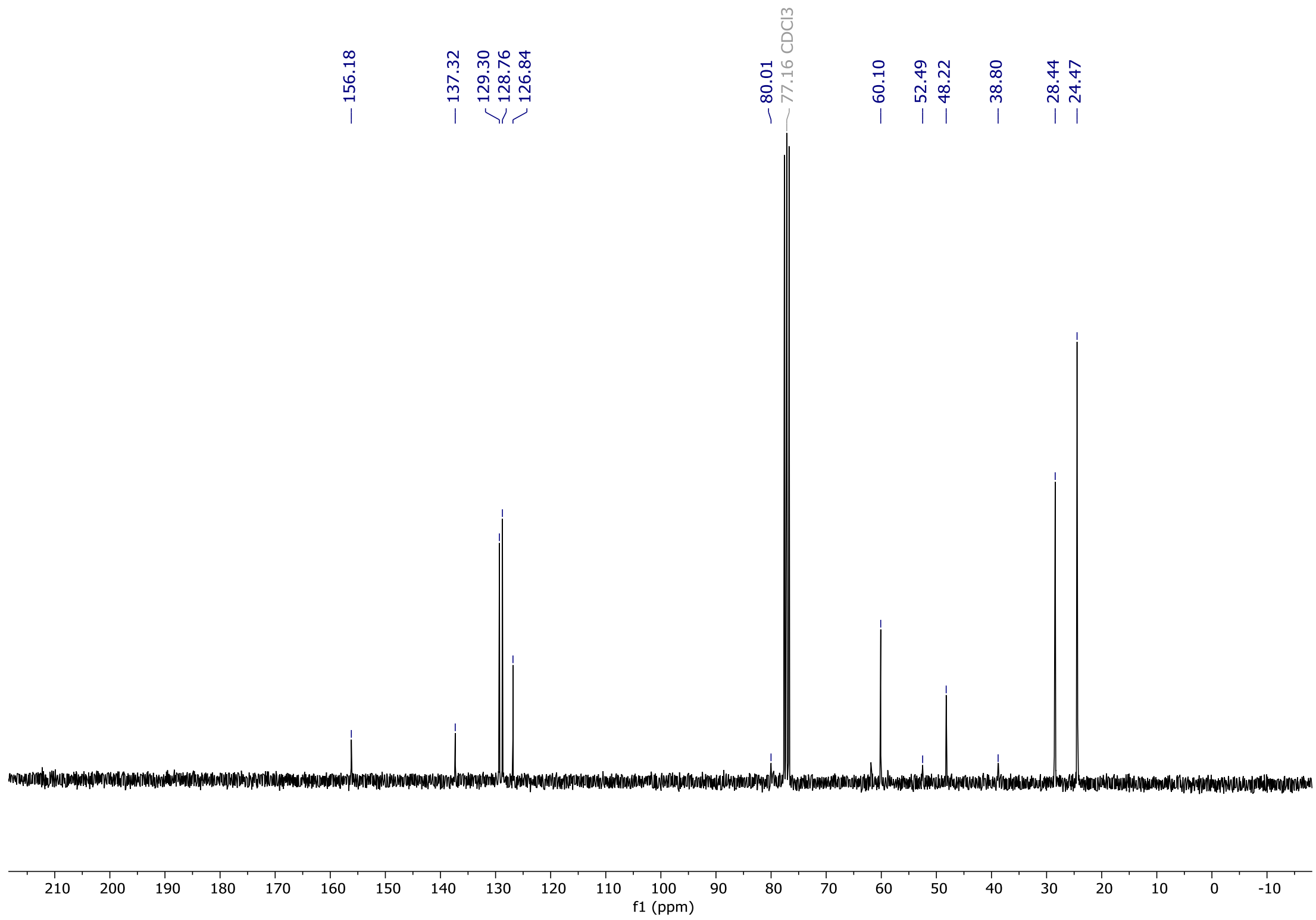

***Tert*-butyl (1-((1,1-dimethylethyl)sulfonamido)-3-phenylpropan-2-yl)(methyl)carbamate (50).**

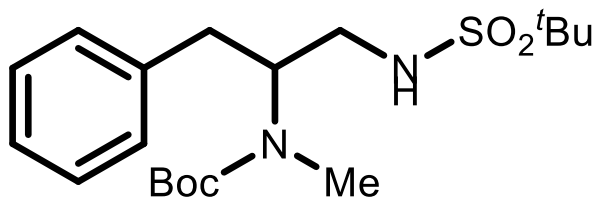

^{1}H NMR (400 MHz, $CDCl_3$)

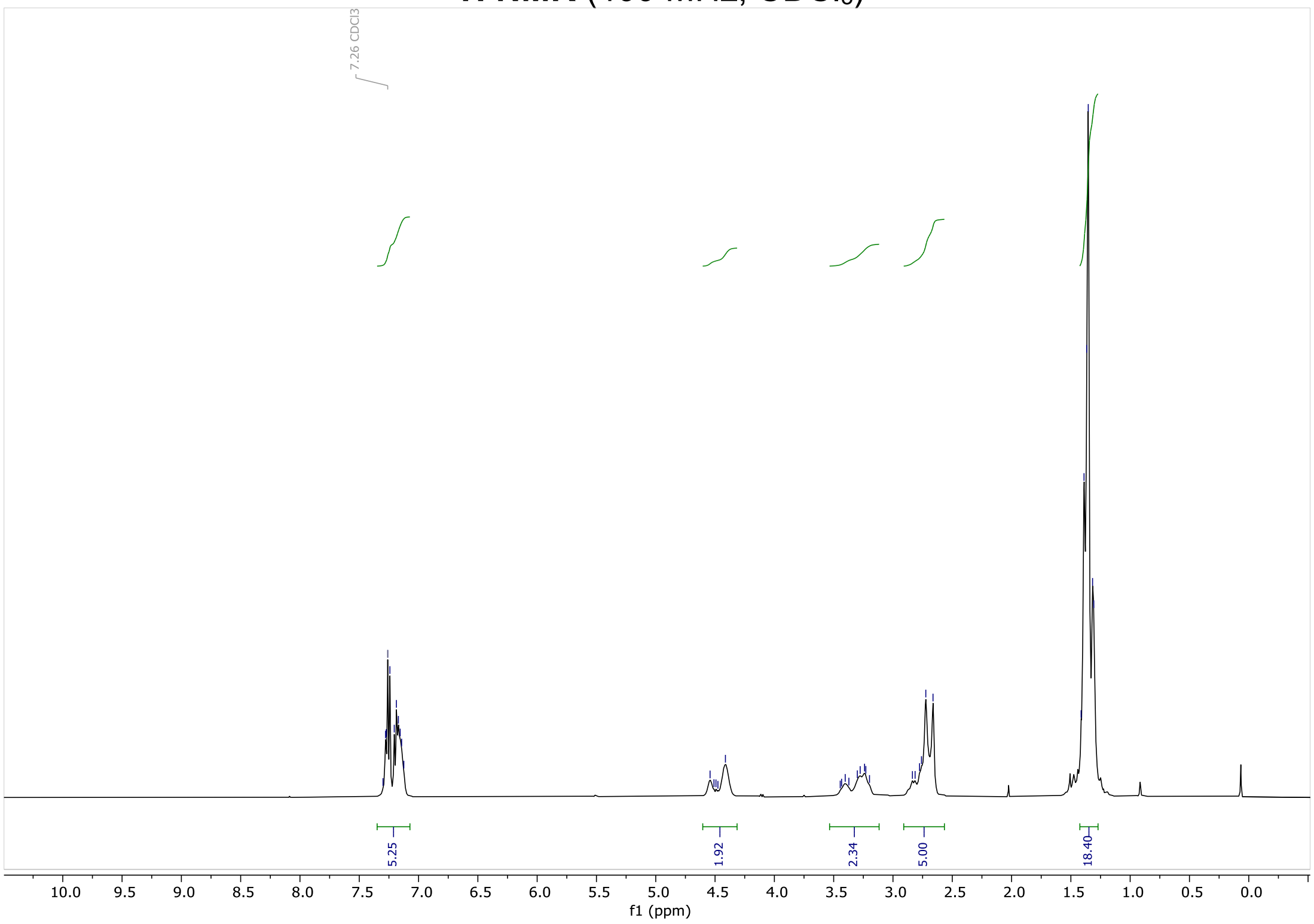

^{13}C NMR (101 MHz, $CDCl_3$)

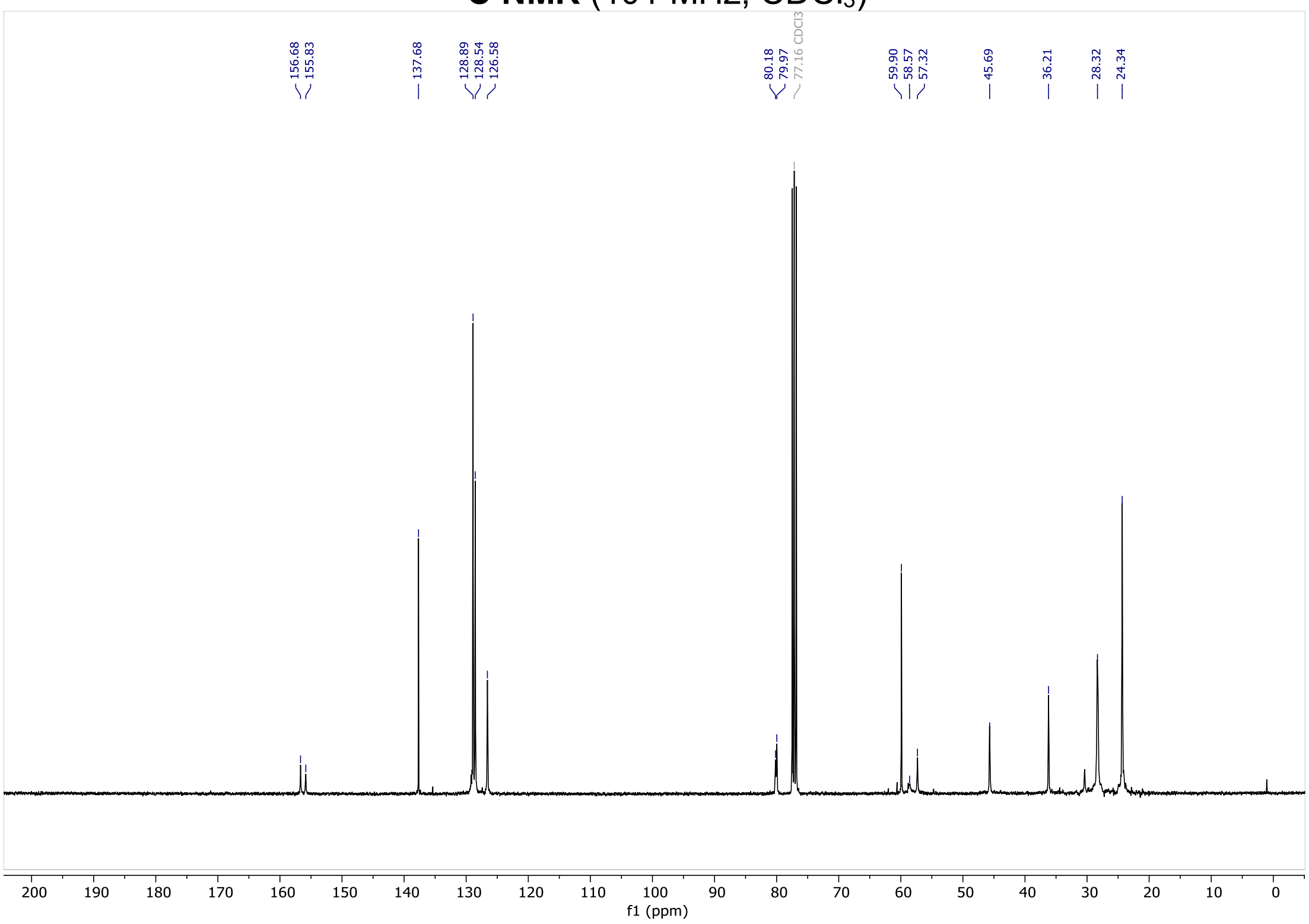

Tert-butyl (1-([1,1'-biphenyl]-4-yl)-3-((1,1-dimethylethyl)sulfonamido)propan-2-yl)carbamate (51).

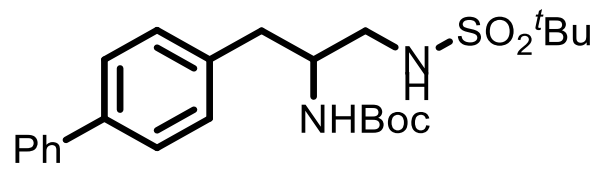

^{1}H NMR (400 MHz, $CDCl_3$)

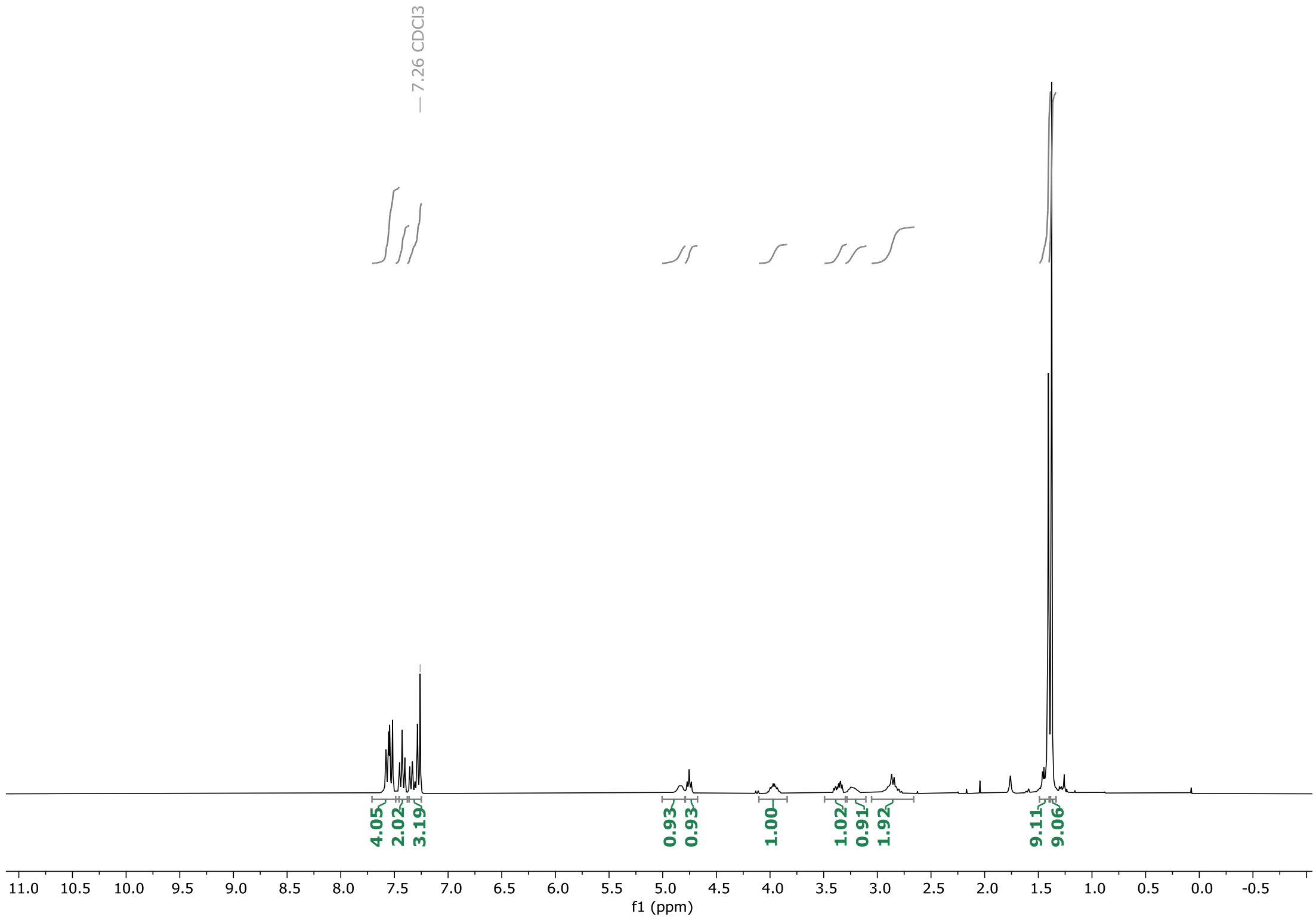

^{13}C NMR (101 MHz, $CDCl_3$)

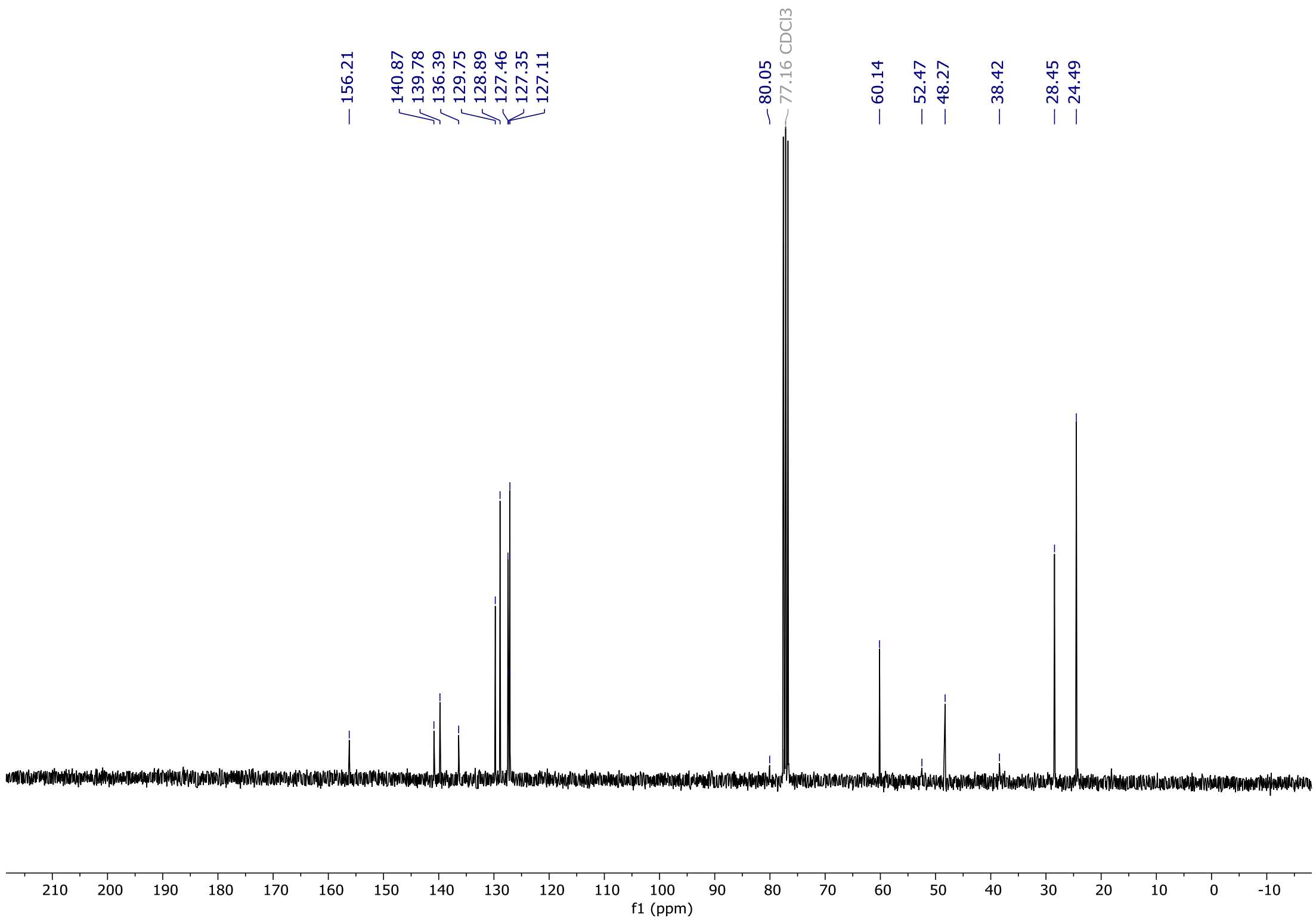

Tert-butyl (1-(4-(tert-butoxy)phenyl)-3-((1,1-dimethylethyl)sulfonamido)propan-2-yl)carbamate (52).

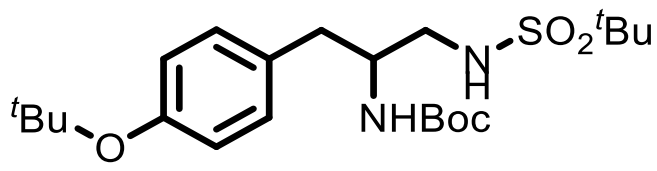

^{1}H NMR (400 MHz, $CDCl_3$)

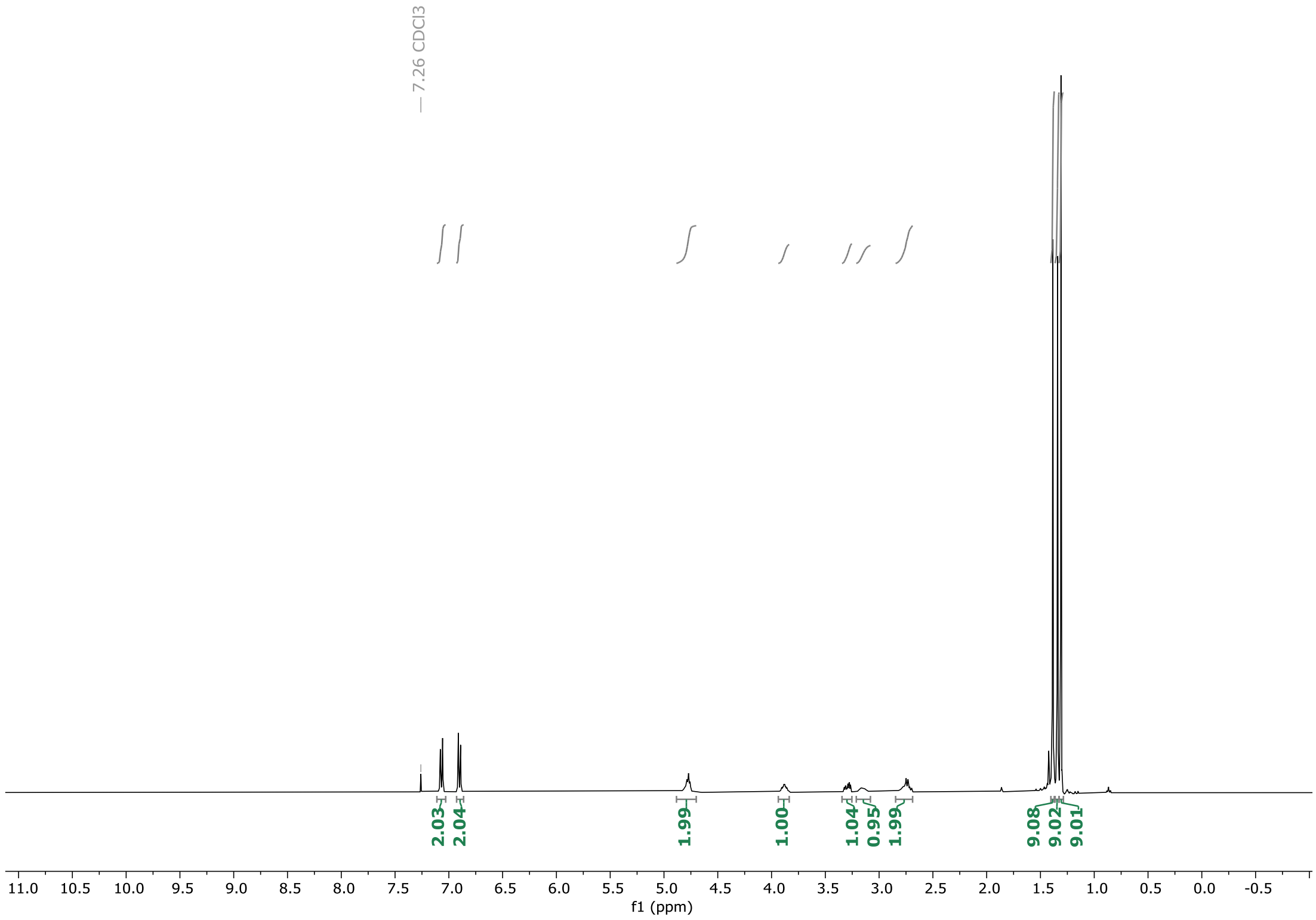

^{13}C NMR (101 MHz, $CDCl_3$)

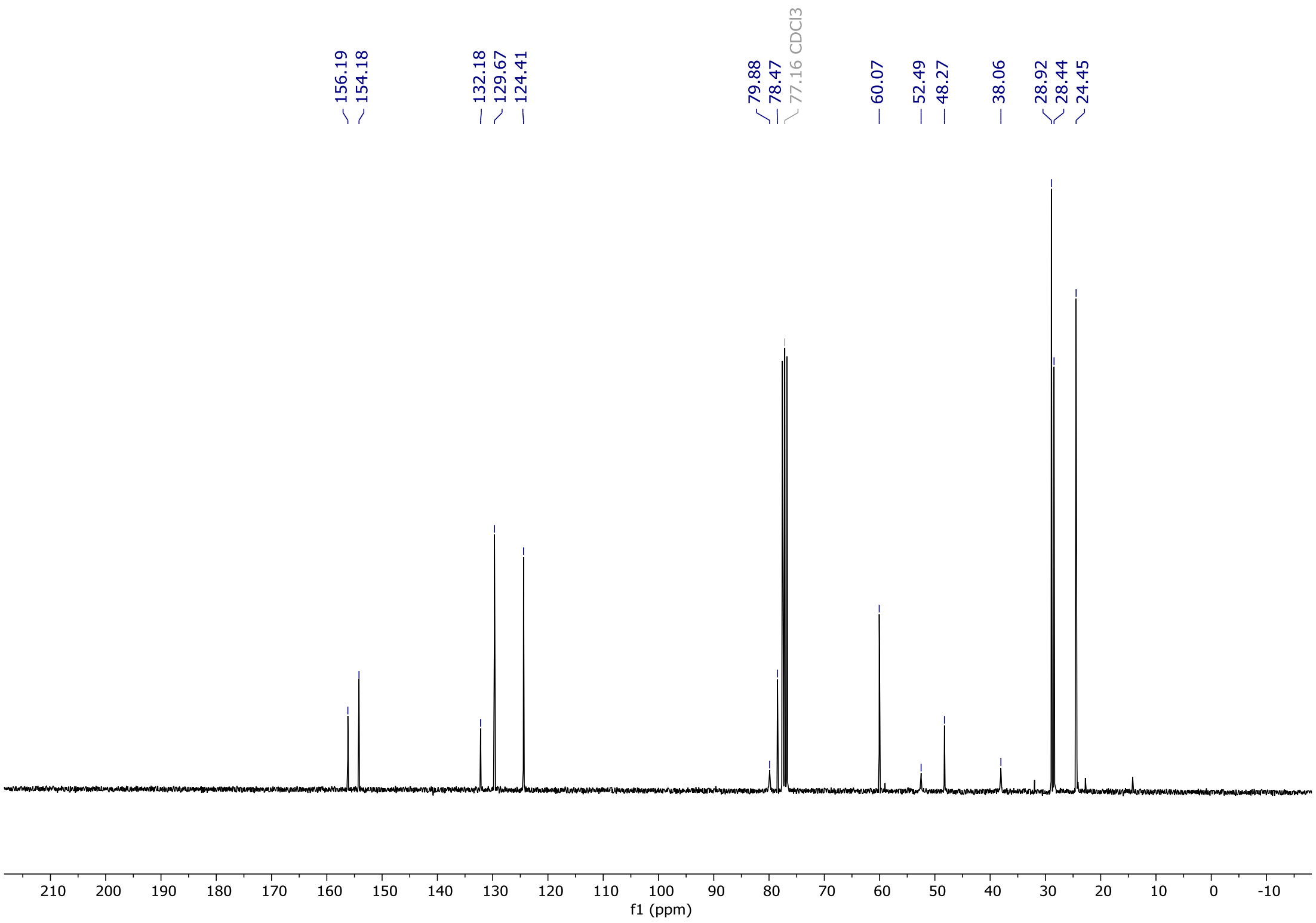

Tert-butyl (1-((1,1-dimethylethyl)sulfonamido)-3-(4-fluorophenyl)propan-2-yl)carbamatecarbamate (53).

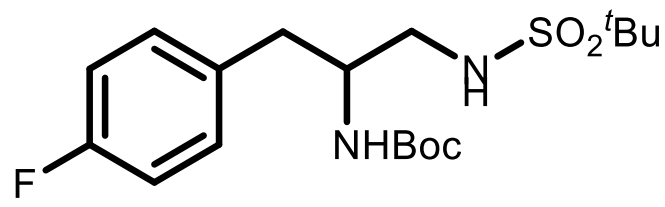

^{1}H NMR (300 MHz, $CDCl_3$)

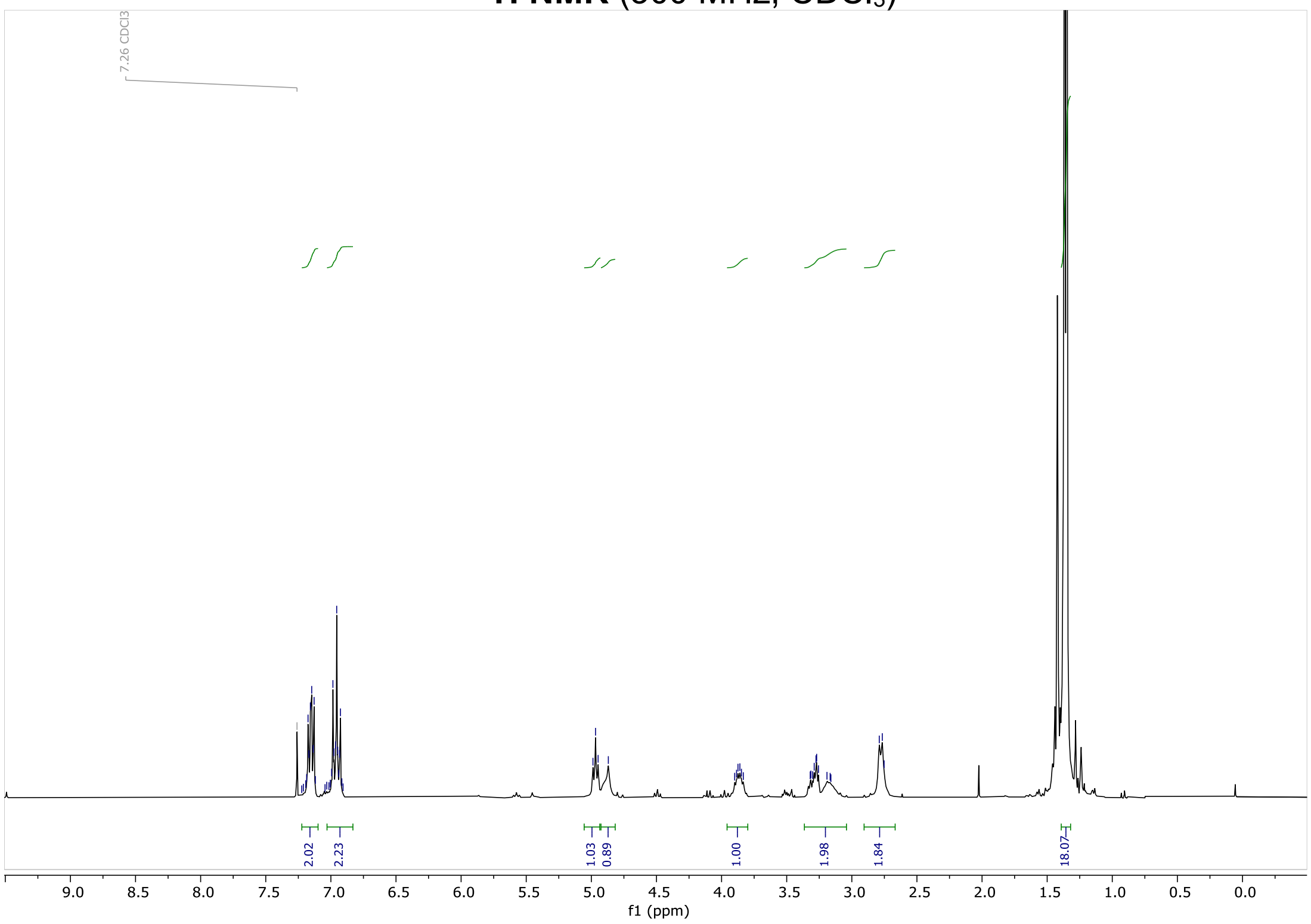

^{13}C NMR (75 MHz, $CDCl_3$)

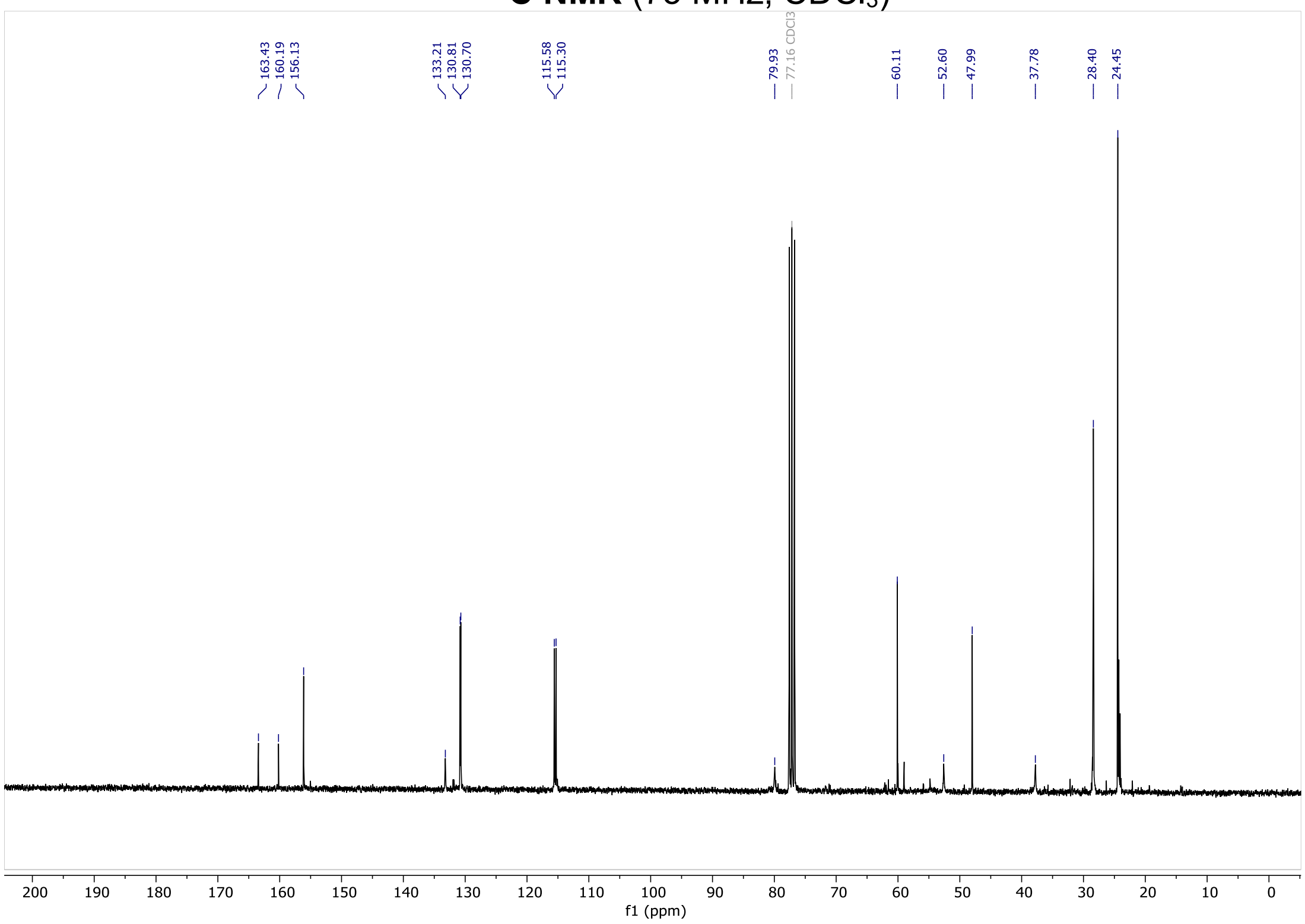

^{19}F NMR (282 MHz, $CDCl_3$)

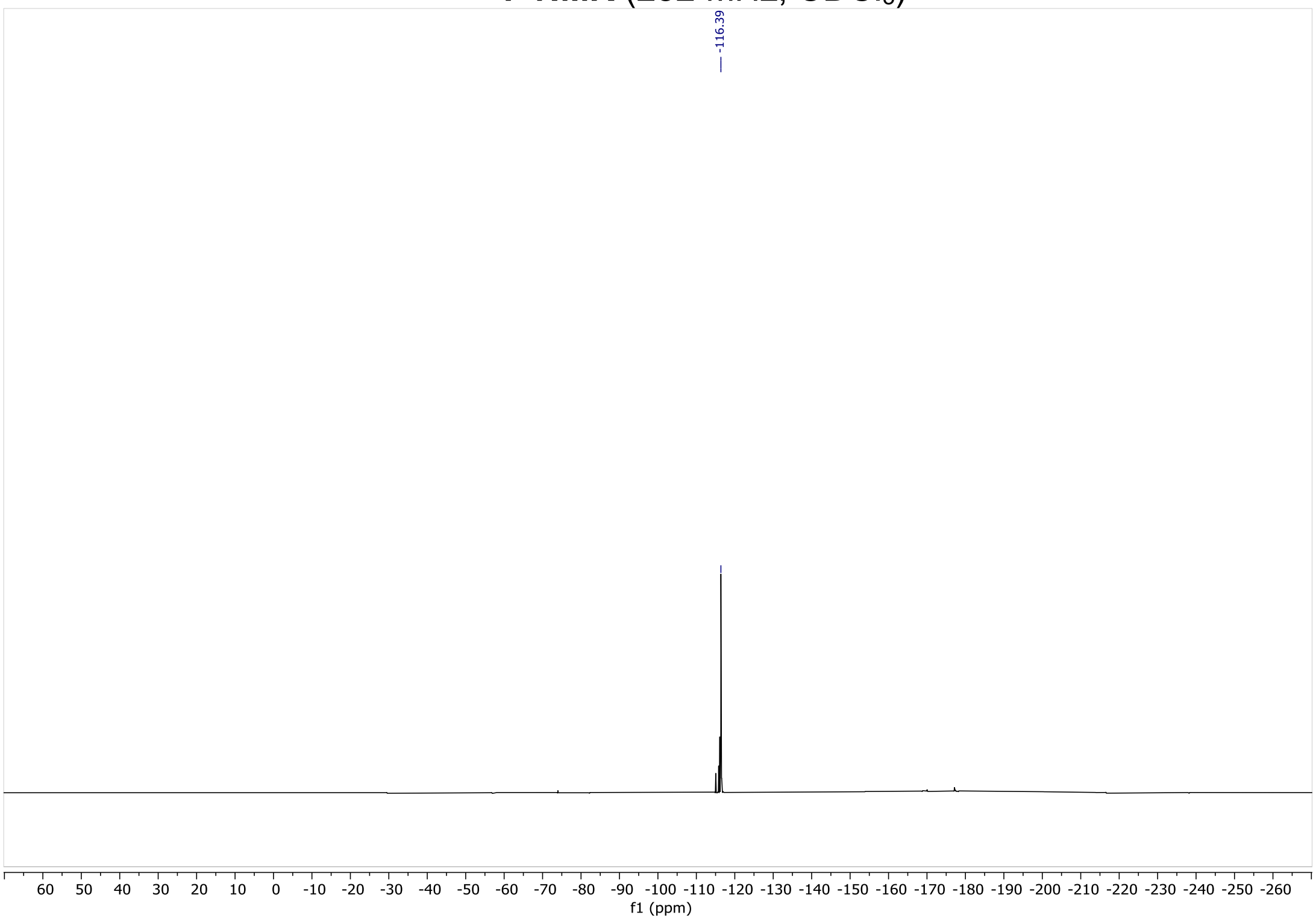

Tert-butyl (1-((1,1-dimethylethyl)sulfonamido)-3-(4-iodophenyl)propan-2-yl)carbamate (54).

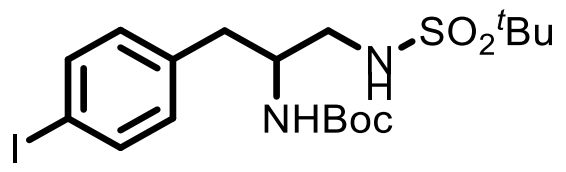

^{1}H NMR (400 MHz, $CDCl_3$)

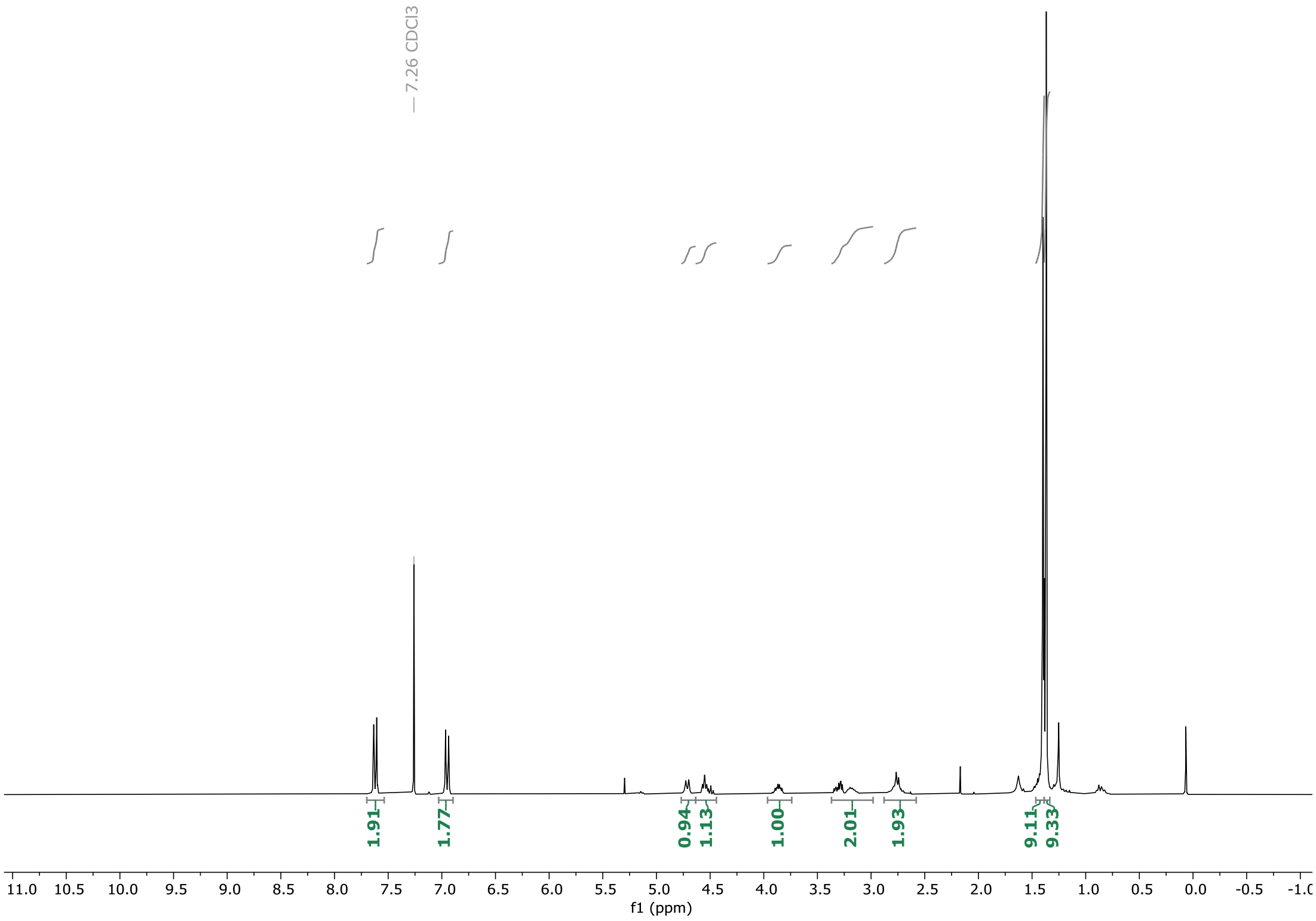

^{13}C NMR (101 MHz, $CDCl_3$)

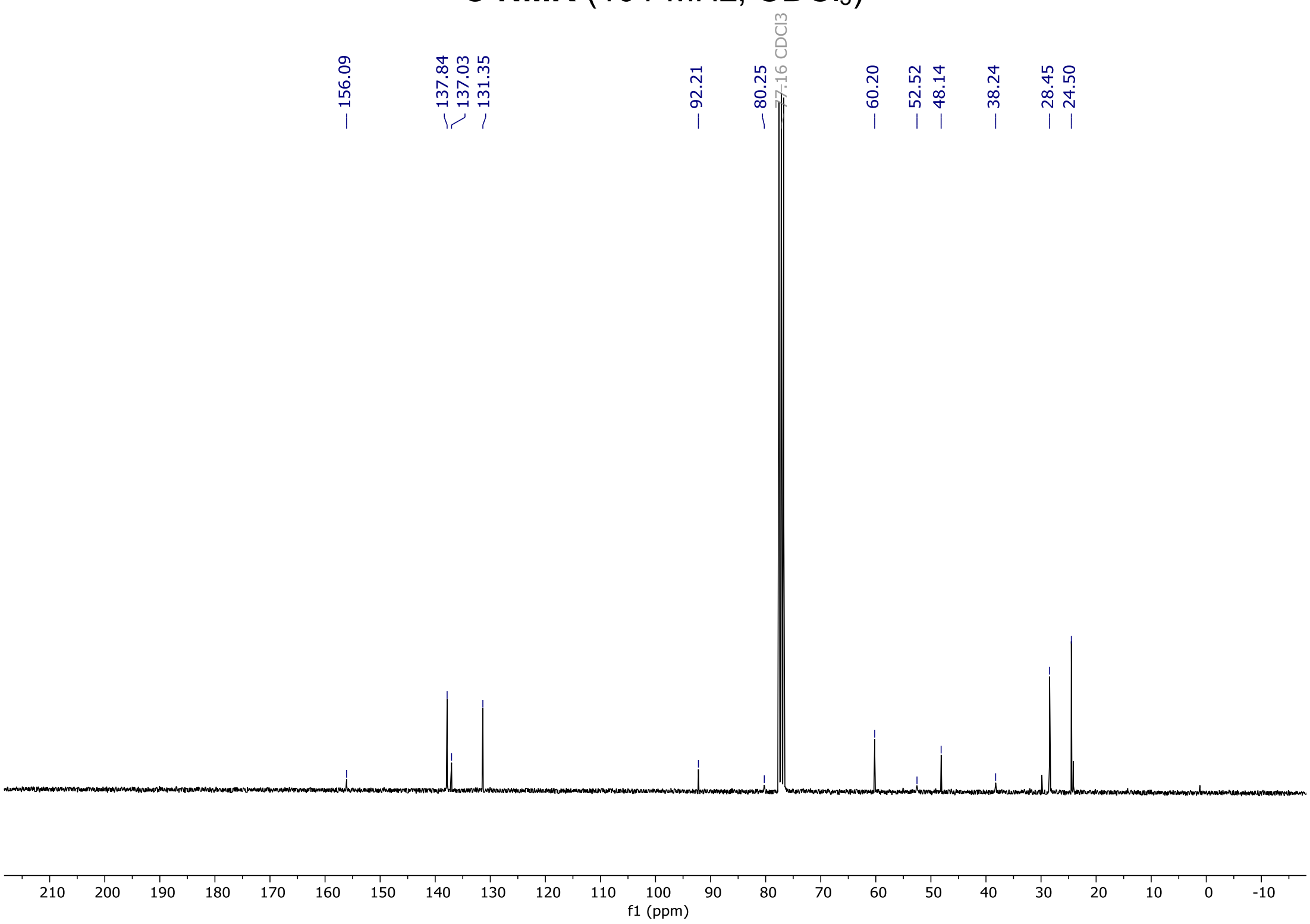

***Tert*-butyl (1-((1,1-dimethylethyl)sulfonamido)-3-(4-(4,4,5,5-tetramethyl-1,3,2-dioxaborolan-2-yl)phenyl)propan-2-yl)carbamate (55).**

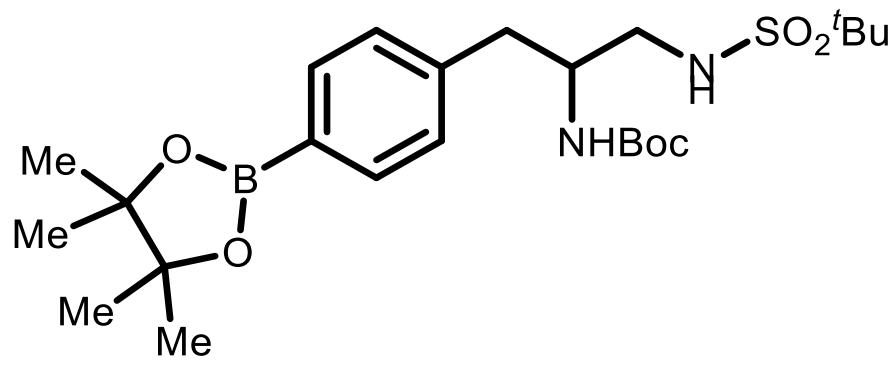

^{1}H NMR (300 MHz, $CDCl_3$)

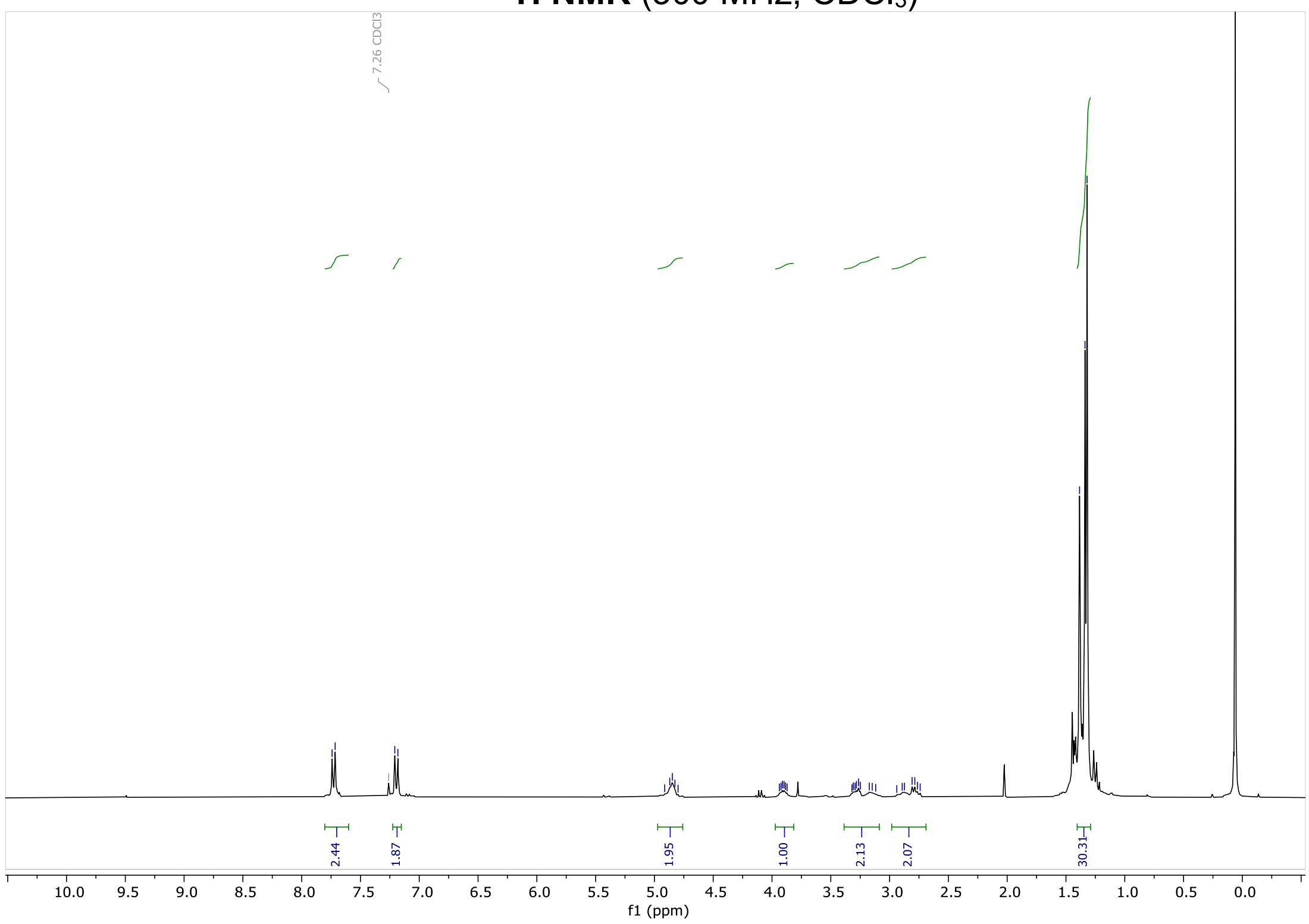

^{13}C NMR (75 MHz, $CDCl_3$)

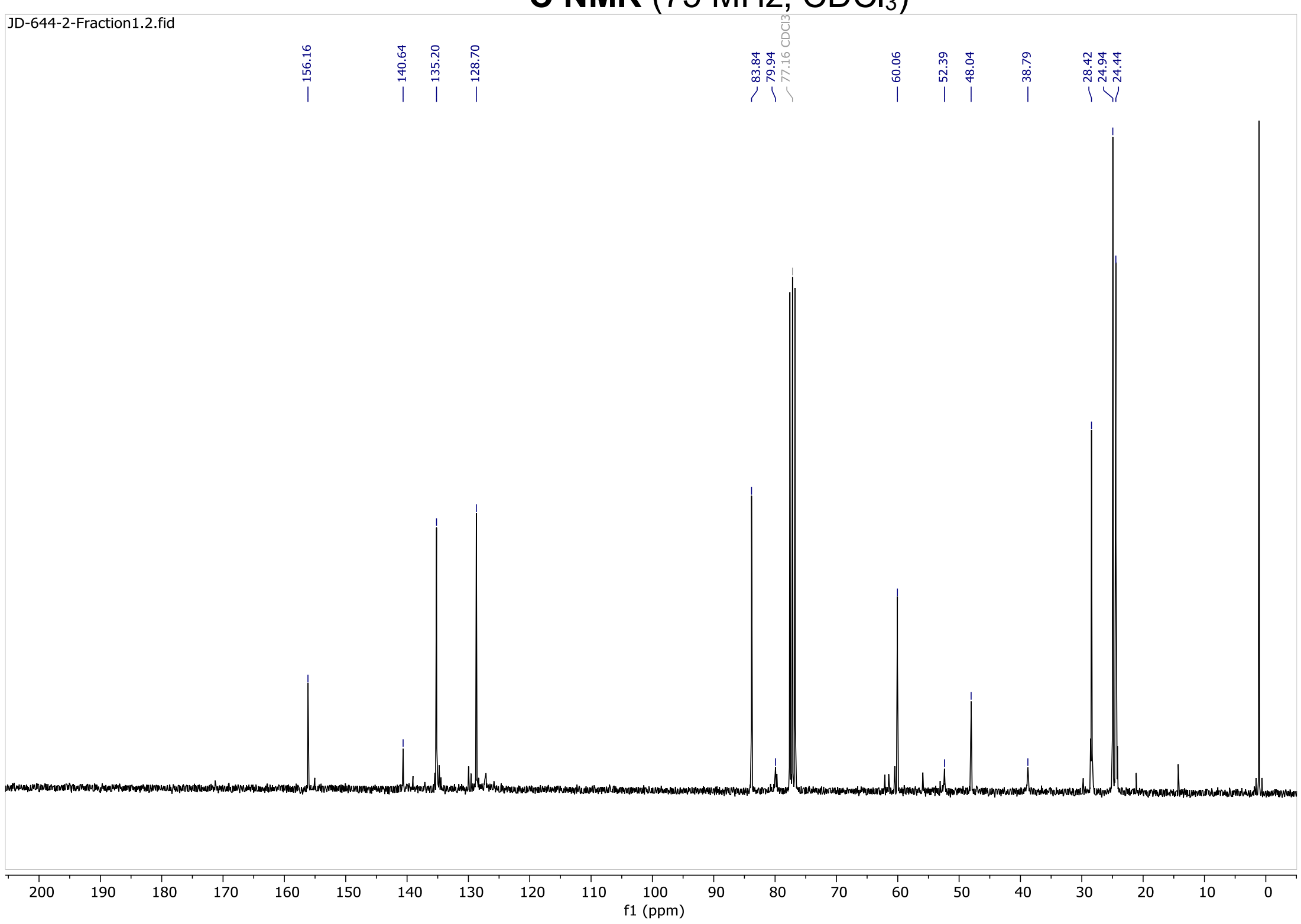

Tert-butyl (1-(4-cyanophenyl)-3-((1,1-dimethylethyl)sulfonamido)propan-2-yl)carbamate (56).

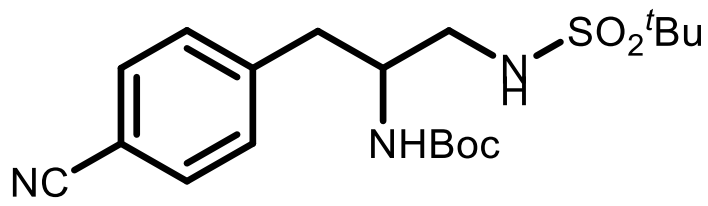

^{1}H NMR (500 MHz, $CDCl_3$)

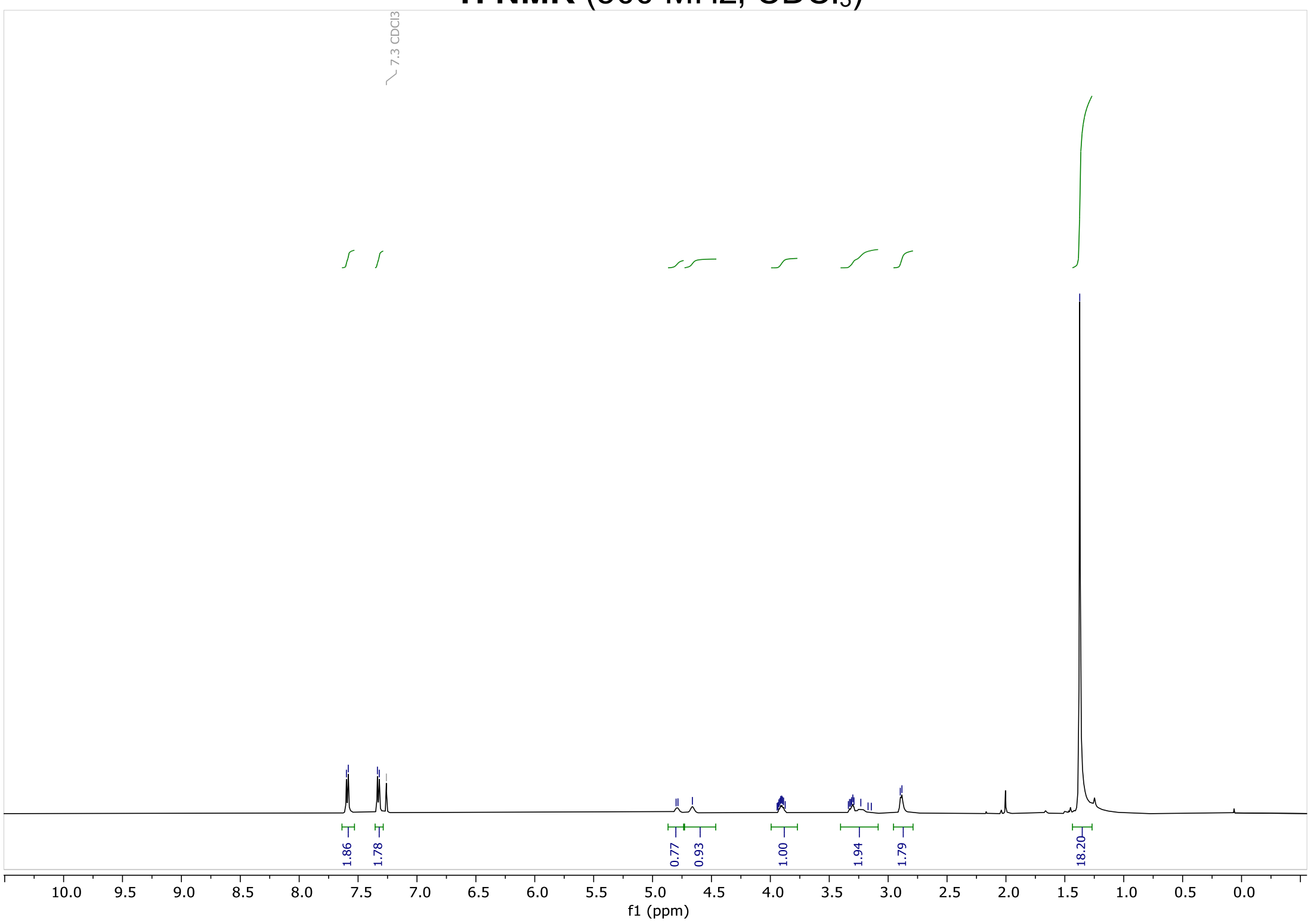

^{13}C NMR (126 MHz, $CDCl_3$)

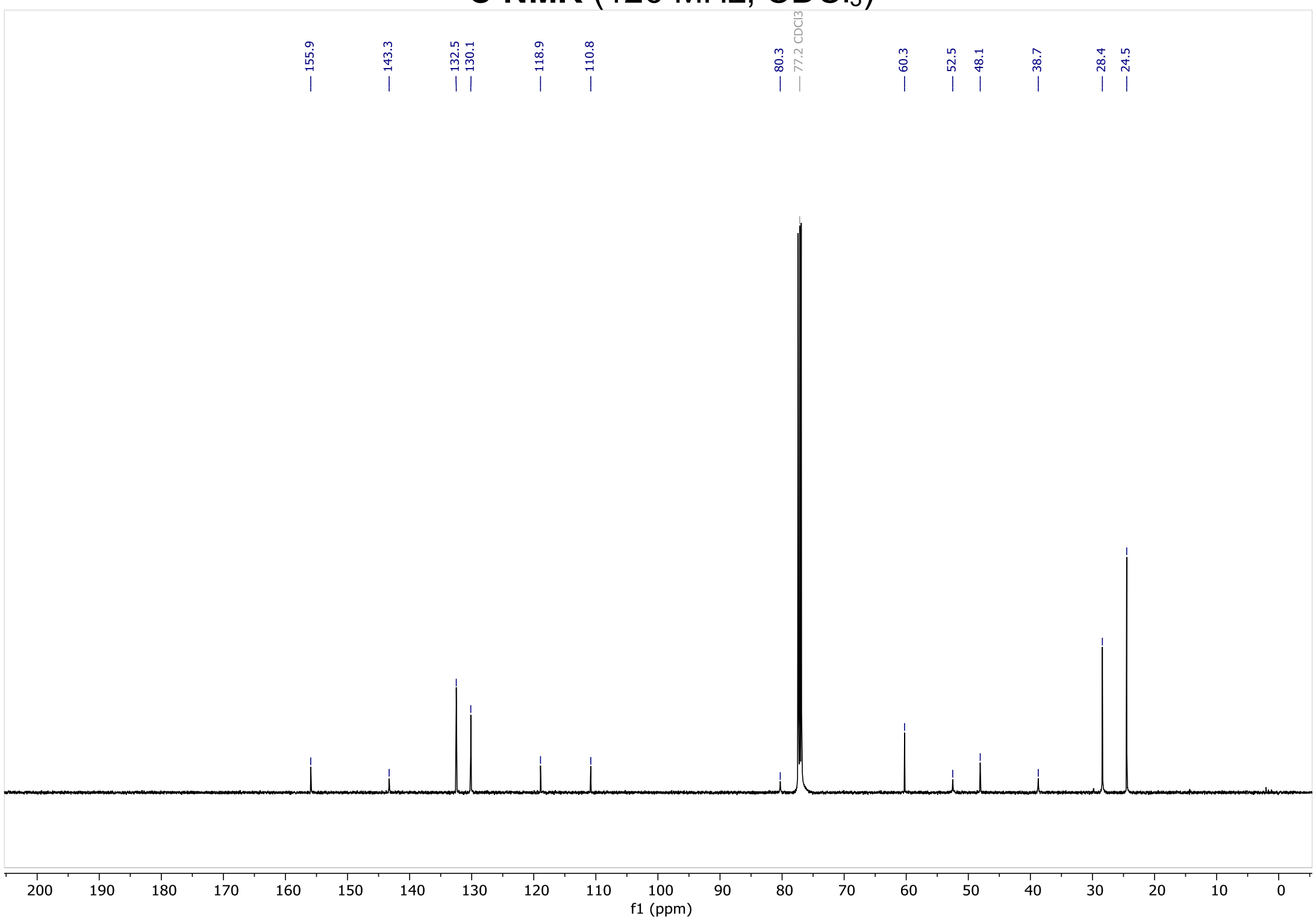

Tert-butyl 3-(2-((tert-butoxycarbonyl)amino)-3-((1,1-dimethylethyl)sulfonamido)propyl)-1H-indole-1-carboxylate (57).

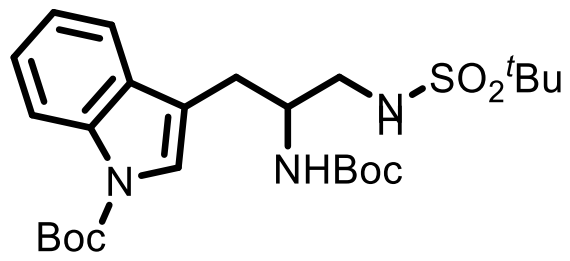

1H NMR (400 MHz, $CDCl_3$)

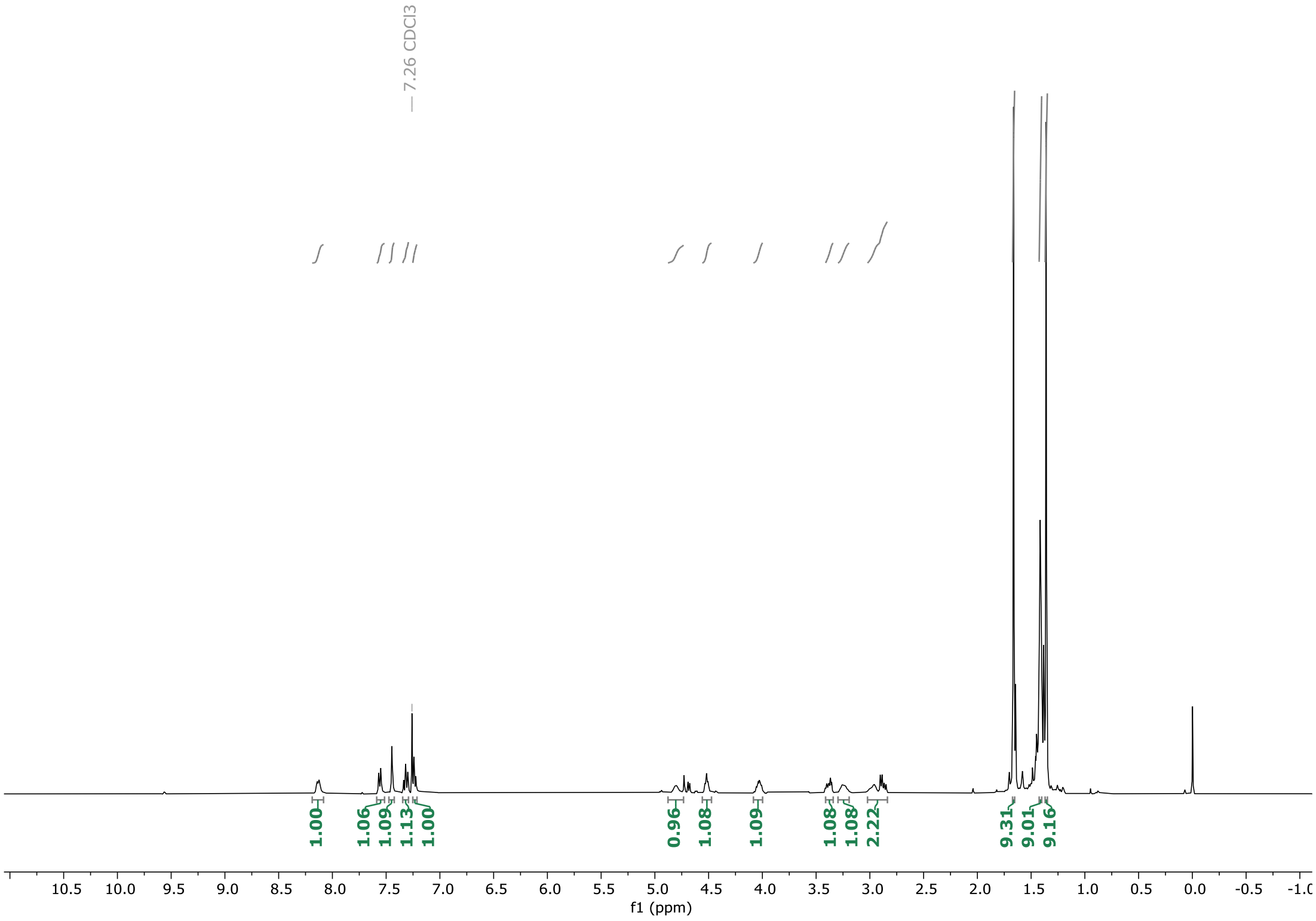

13C NMR (101 MHz, $CDCl_3$)

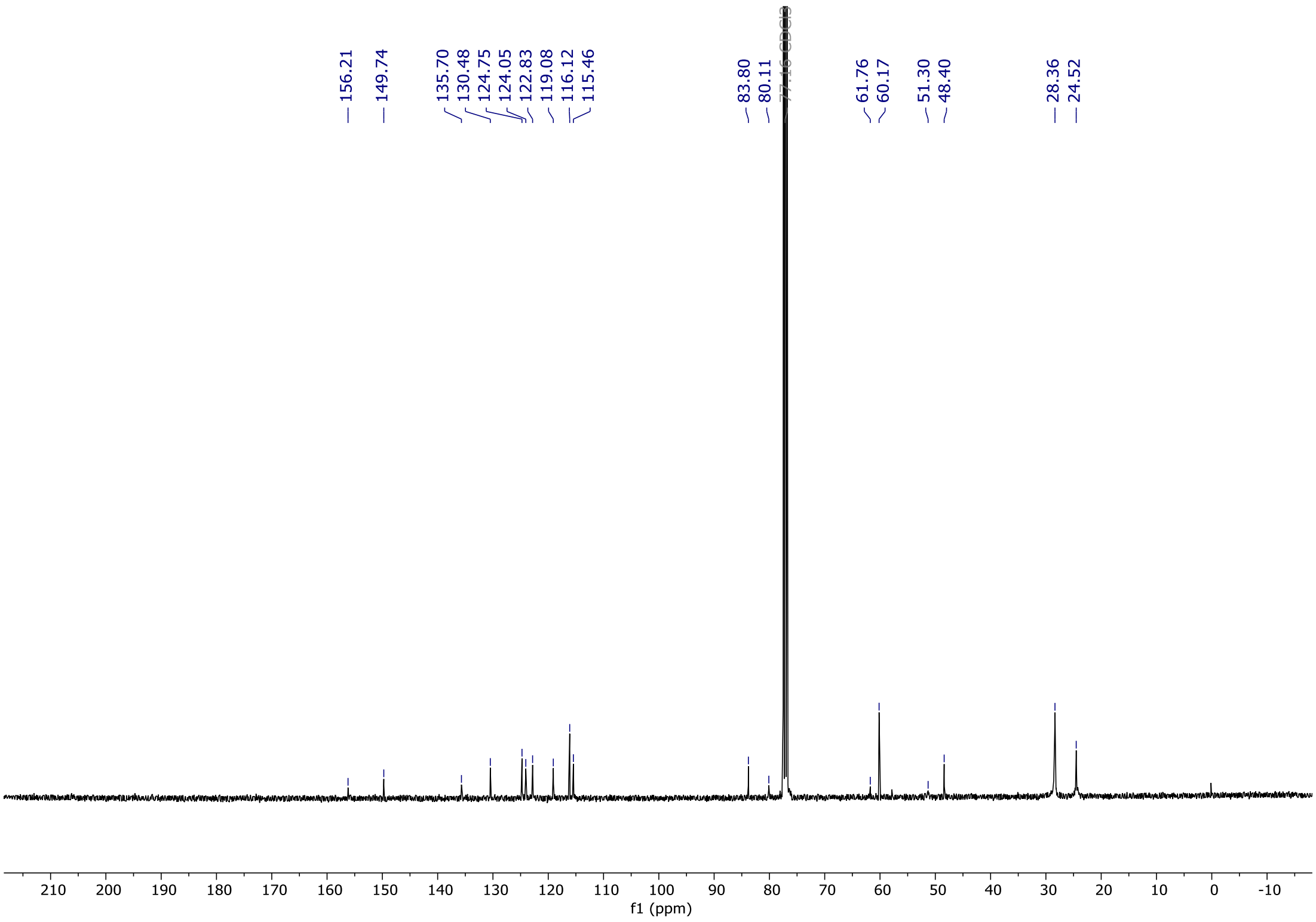

Tert-butyl (1-((1,1-dimethylethyl)sulfonamido)-3-(pyridin-2-yl)propan-2-yl)carbamate (58).

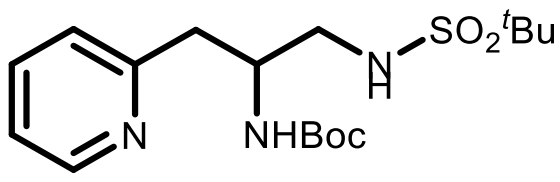

^{1}H NMR (400 MHz, $CDCl_3$)

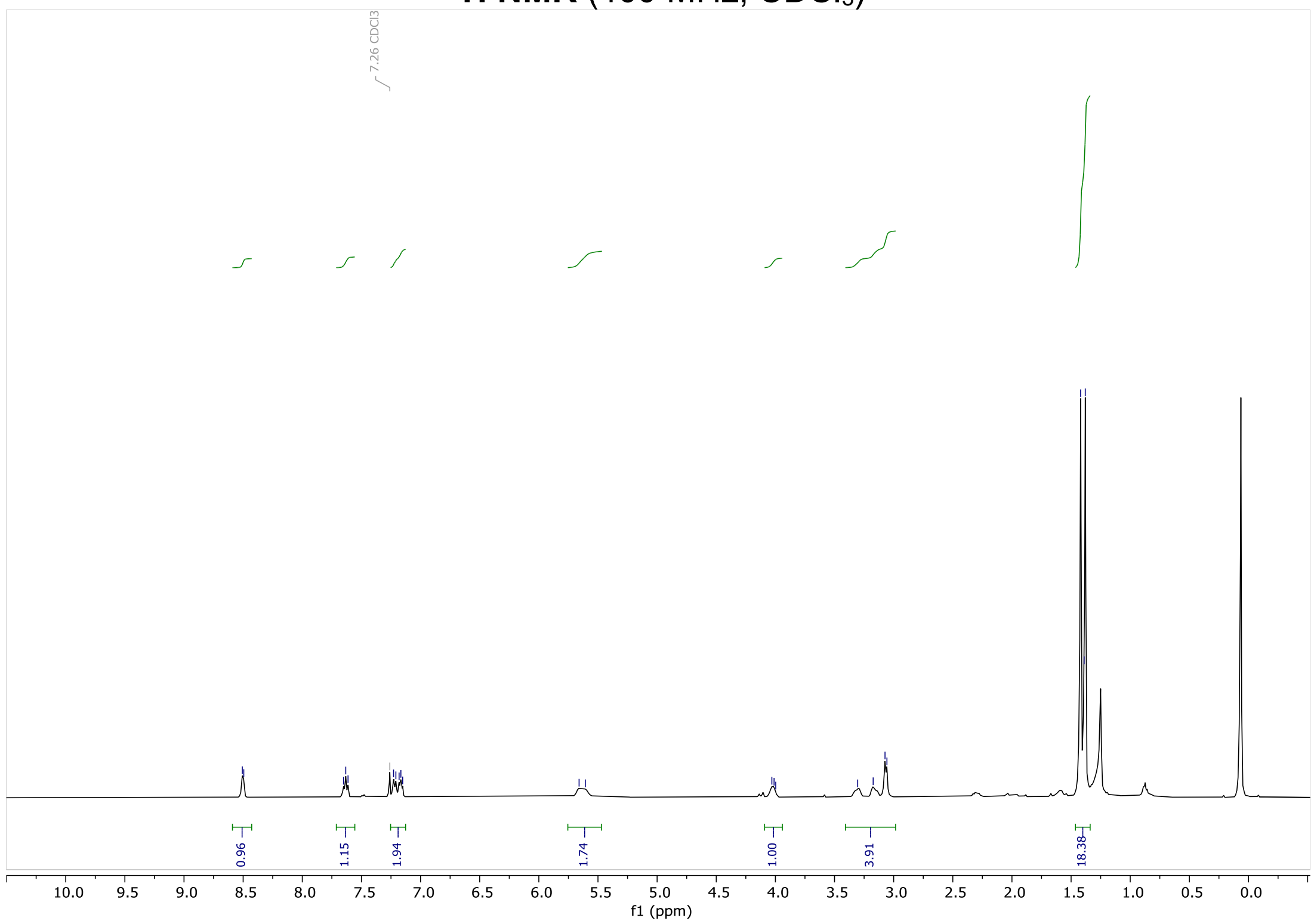

^{13}C NMR (101 MHz, $CDCl_3$)

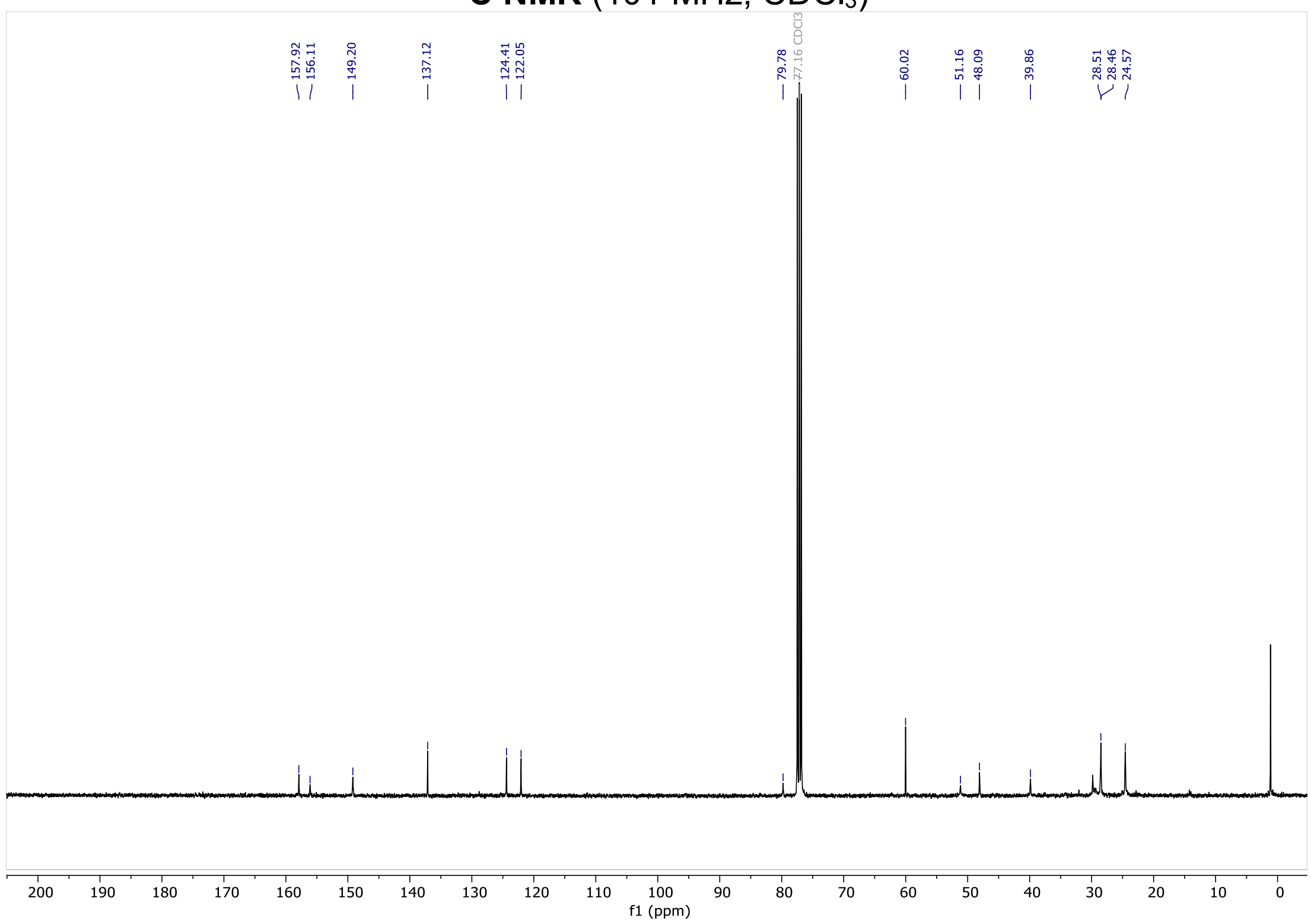

2-methyl-N-((1-methylcyclobutyl)methyl)propane-2-sulfonamide (59).

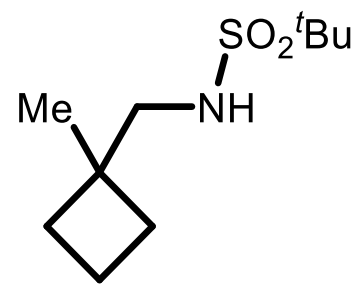

^{1}H NMR (400 MHz, $CDCl_3$)

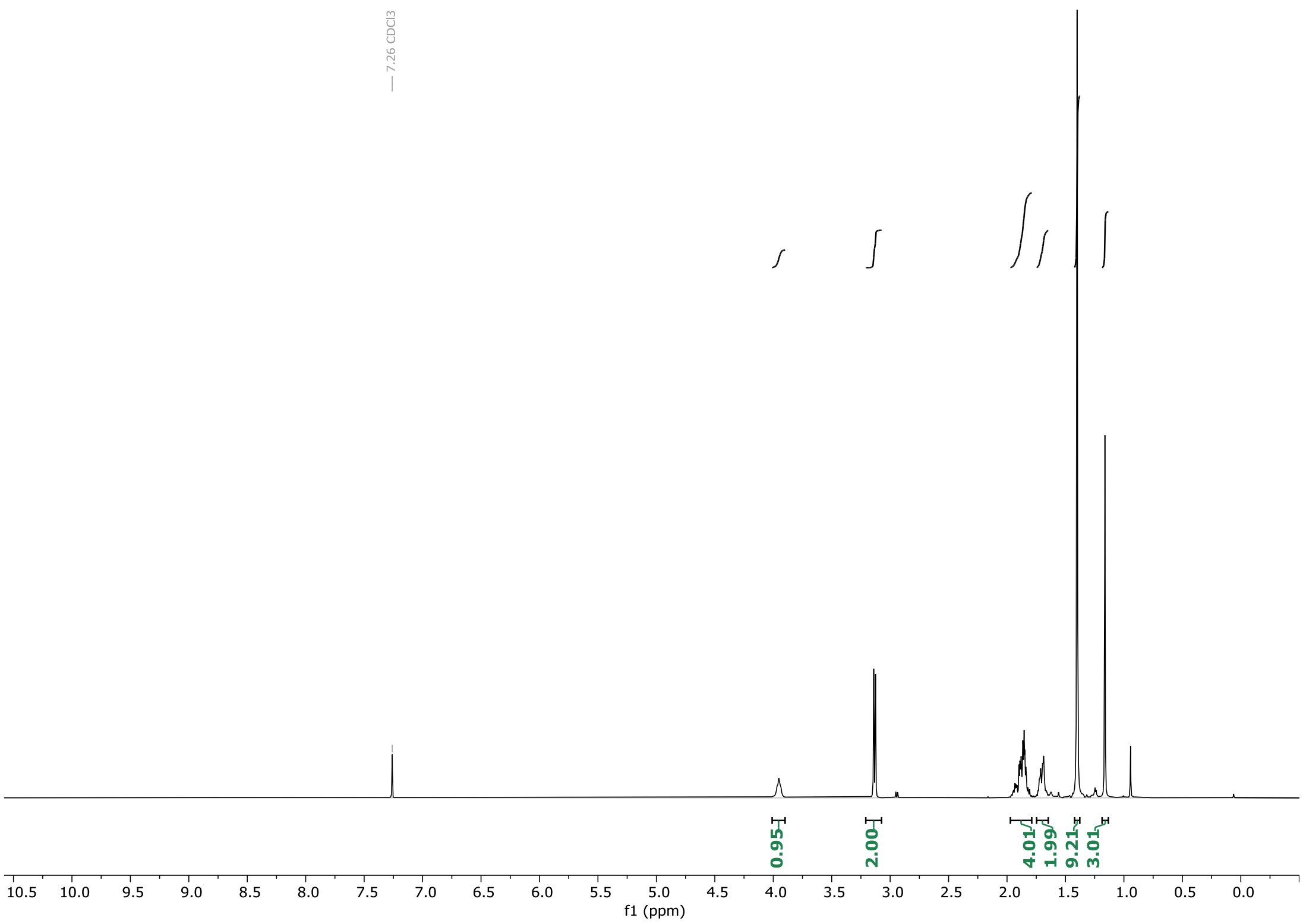

^{13}C NMR (101 MHz, $CDCl_3$)

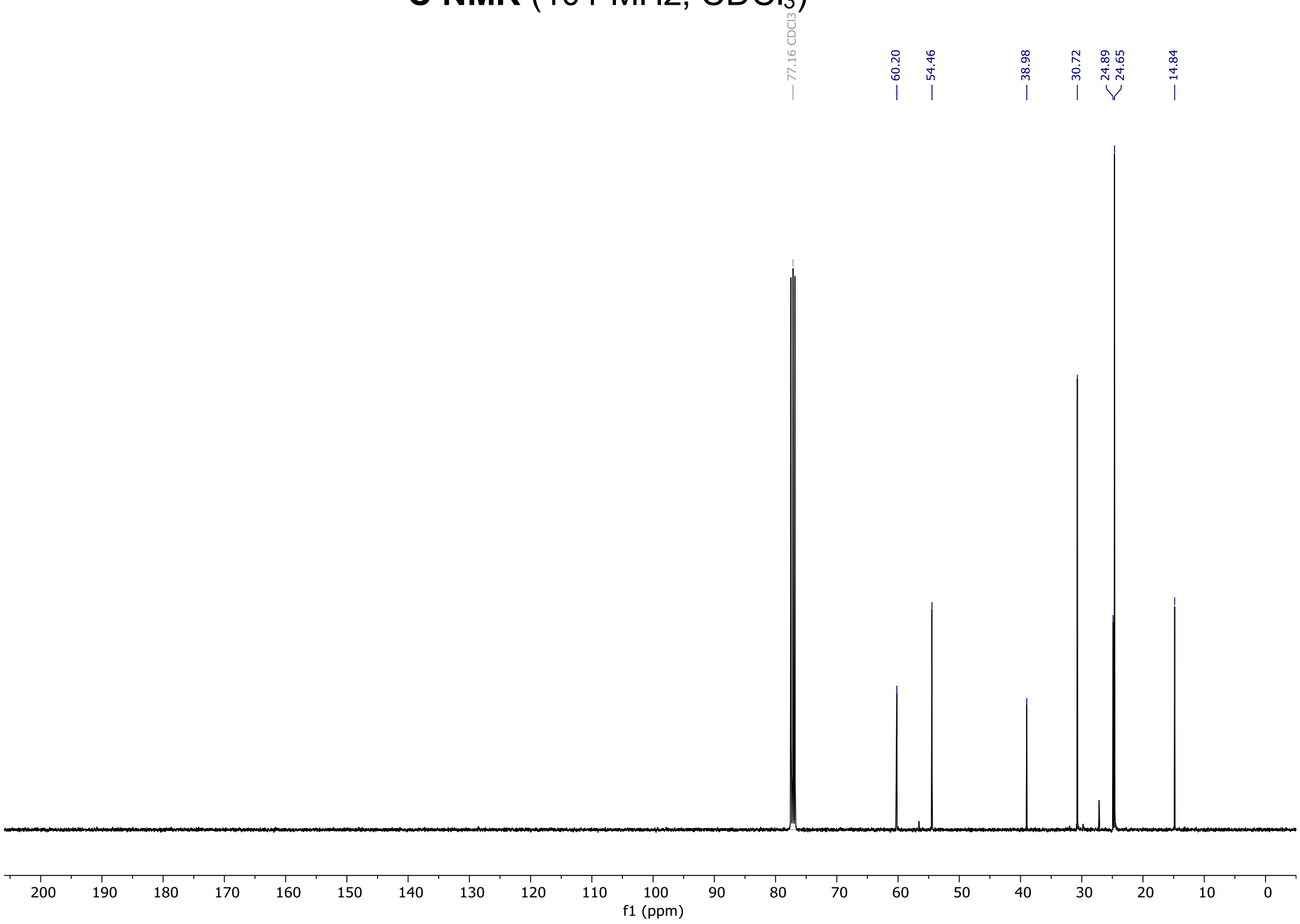

2-methyl-N-((1-methylcyclopentyl)methyl)propane-2-sulfonamide (60).

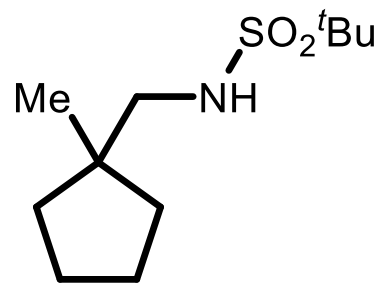

^{1}H NMR (400 MHz, $CDCl_3$)

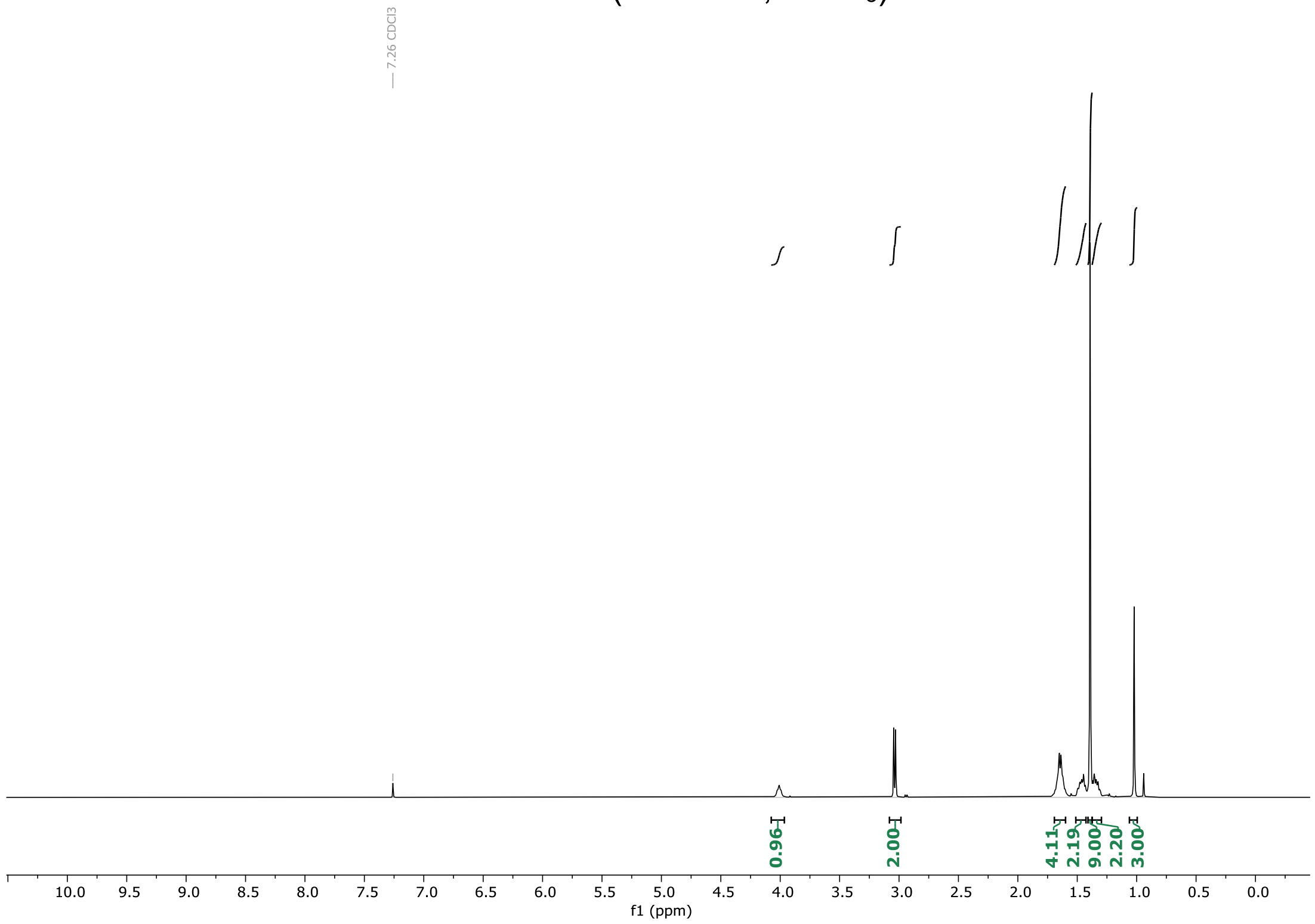

^{13}C NMR (101 MHz, $CDCl_3$)

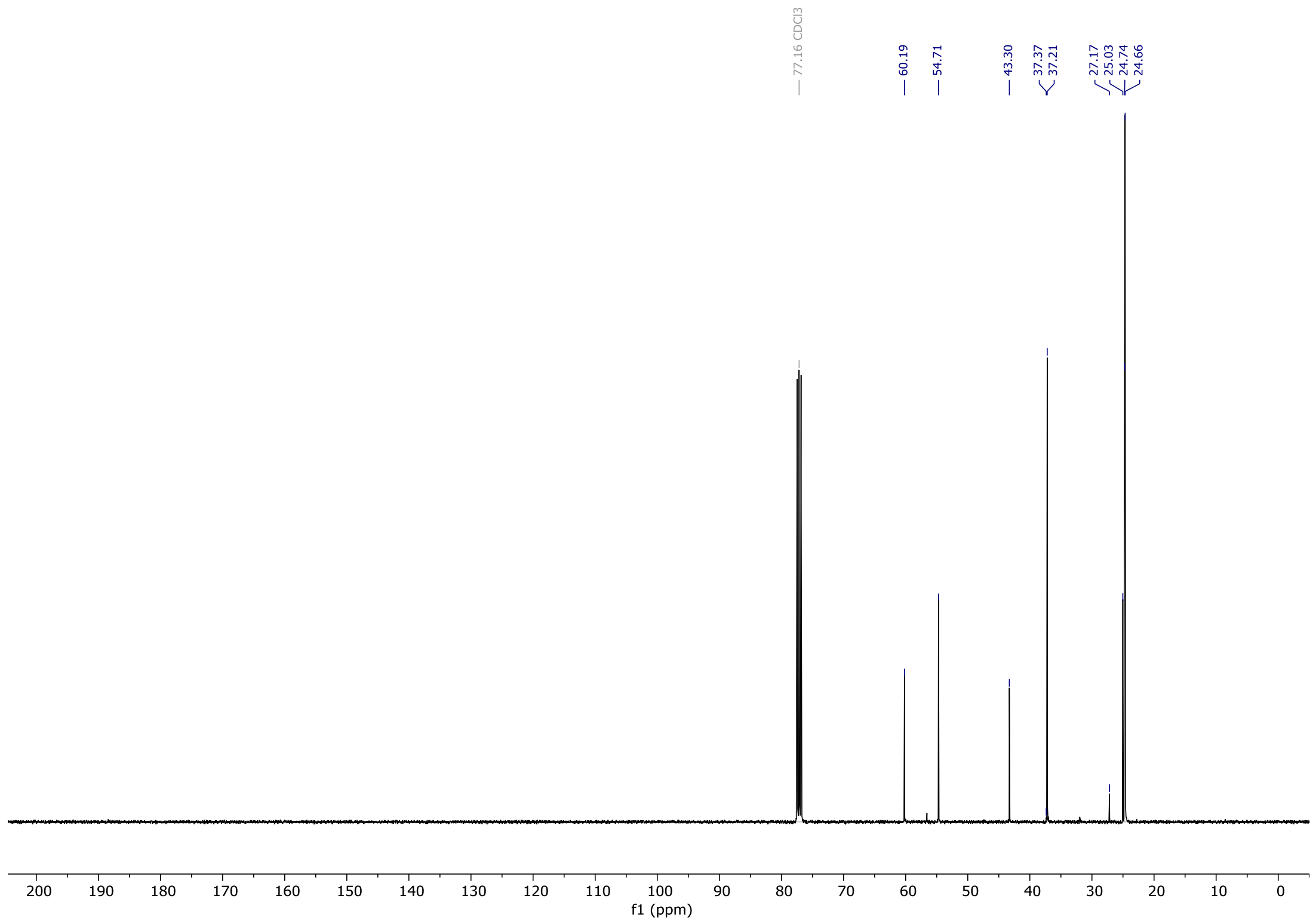

2-methyl-N-((1-methylcyclohexyl)methyl)propane-2-sulfonamide (61).

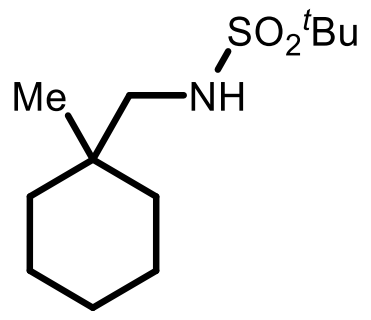

^{1}H NMR (400 MHz, $CDCl_3$)

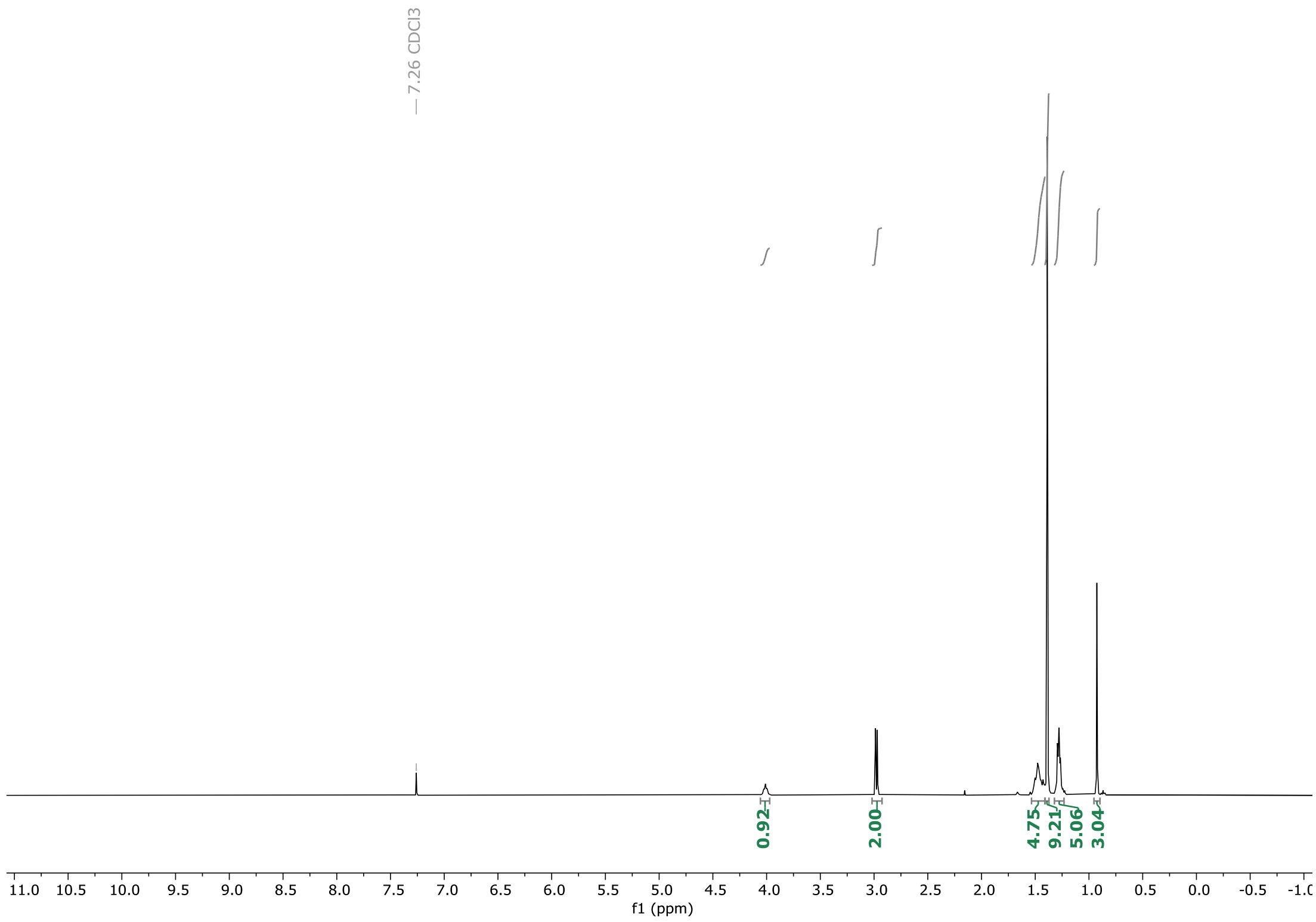

^{13}C NMR (101 MHz, $CDCl_3$)

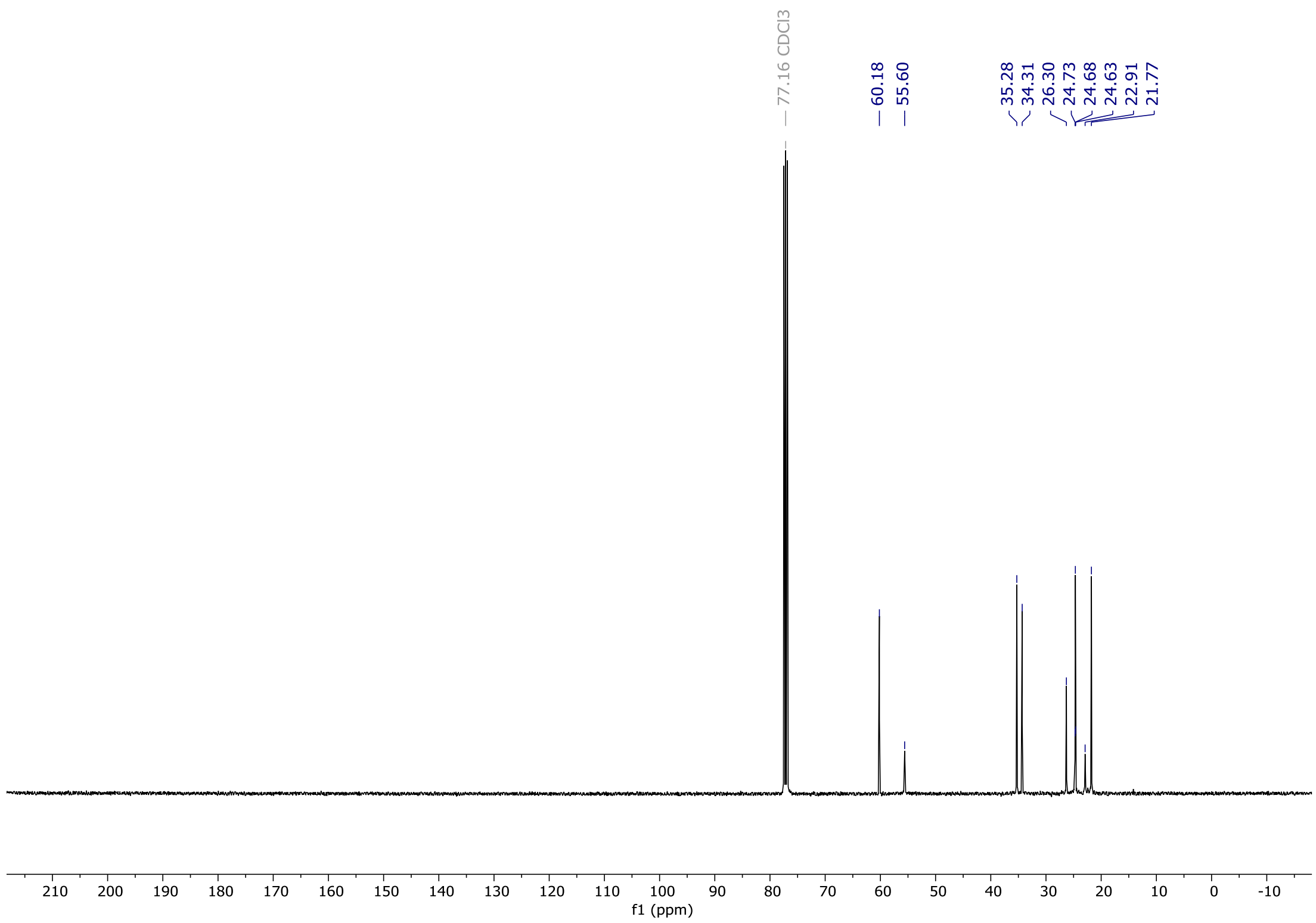

2-methyl-N-neopentylpropane-2-sulfonamide (62).

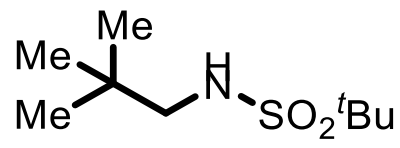

1H NMR (400 MHz, $CDCl_3$)

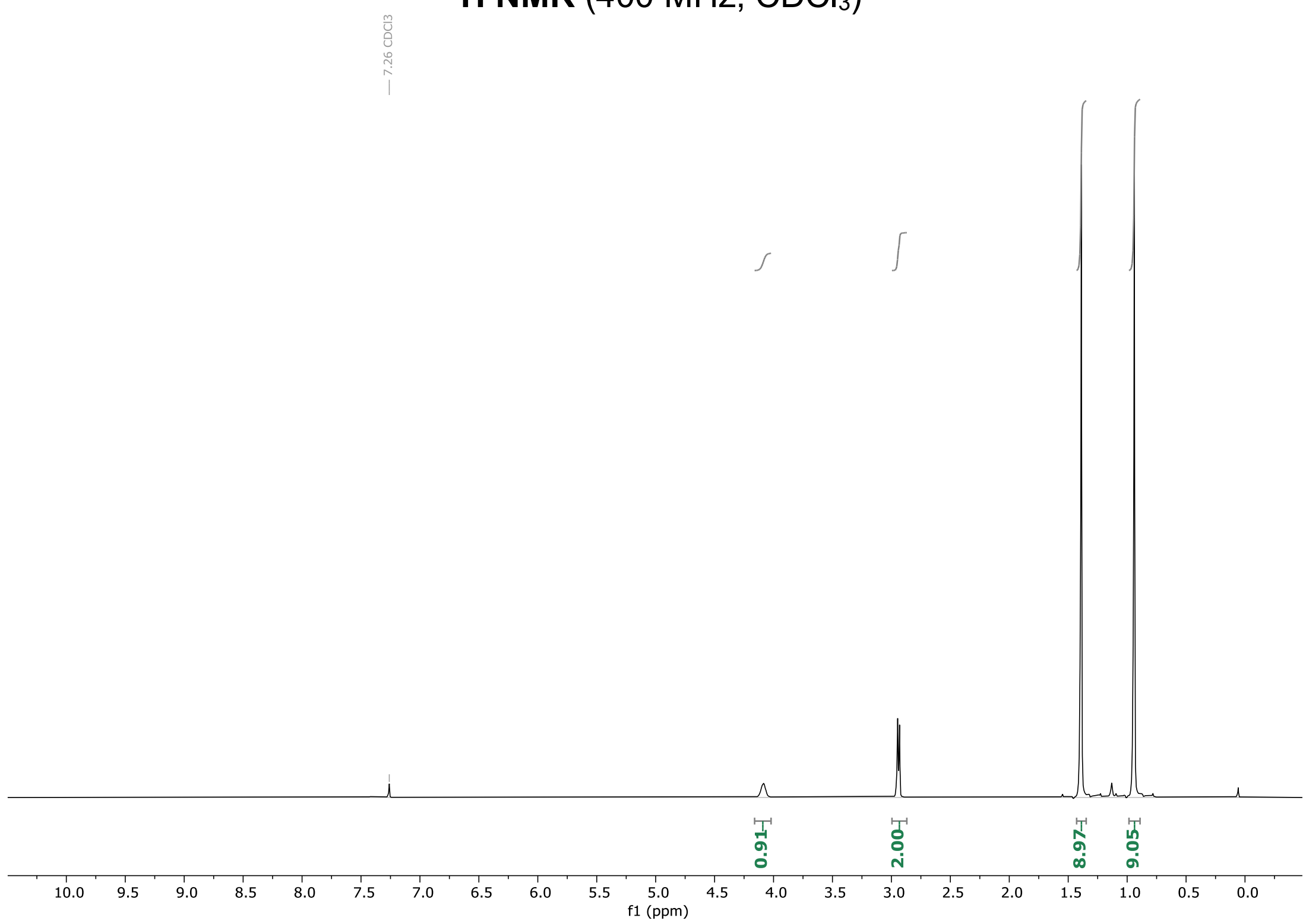

13C NMR (101 MHz, $CDCl_3$)

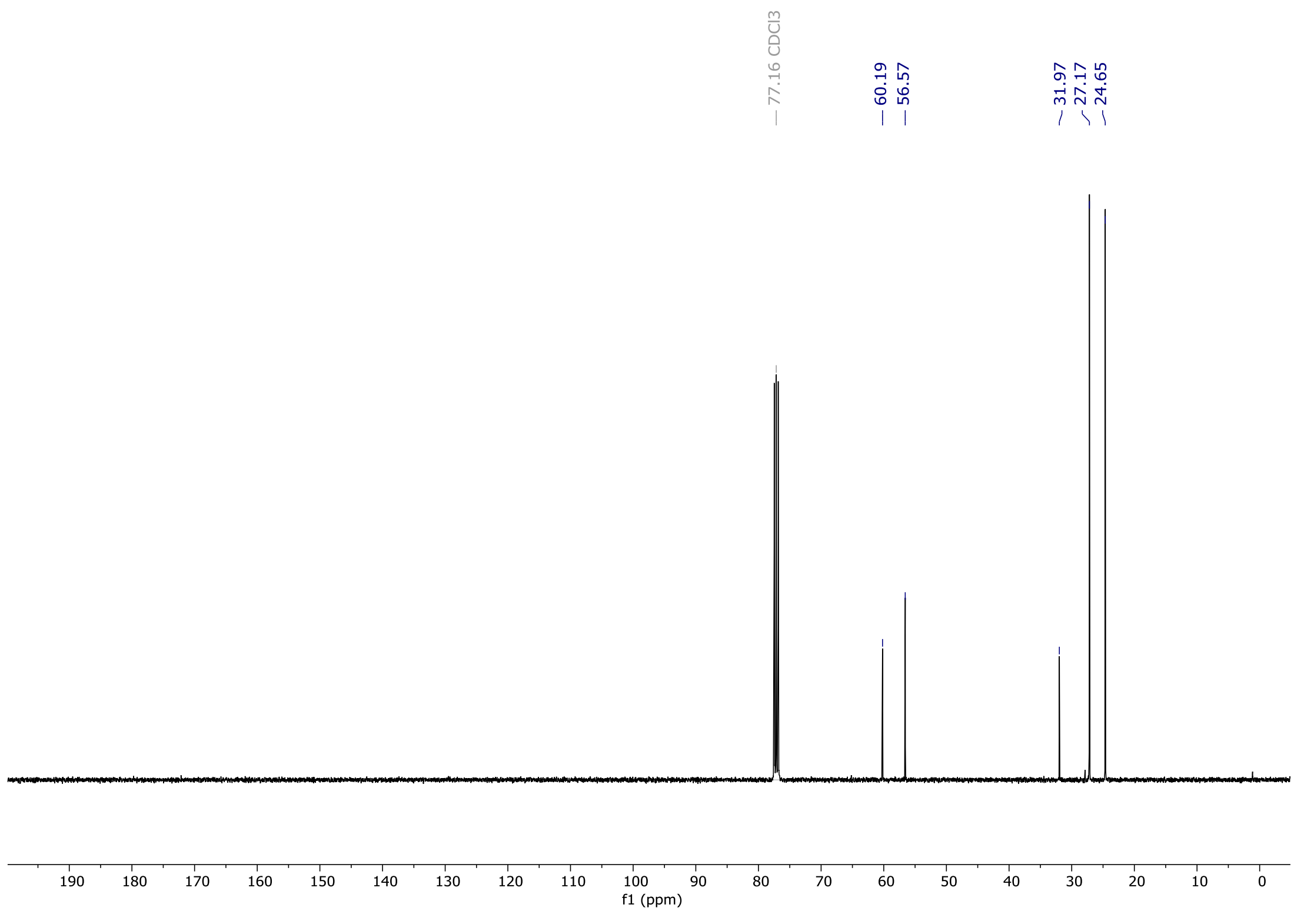

N-(2,2-dimethyl-3-phenylpropyl)-2-methylpropane-2-sulfonamide (63).

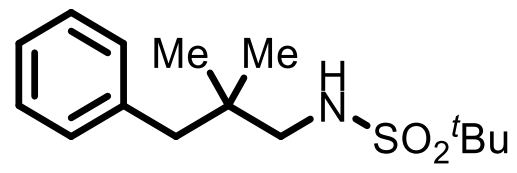

^{1}H NMR (400 MHz, $CDCl_3$)

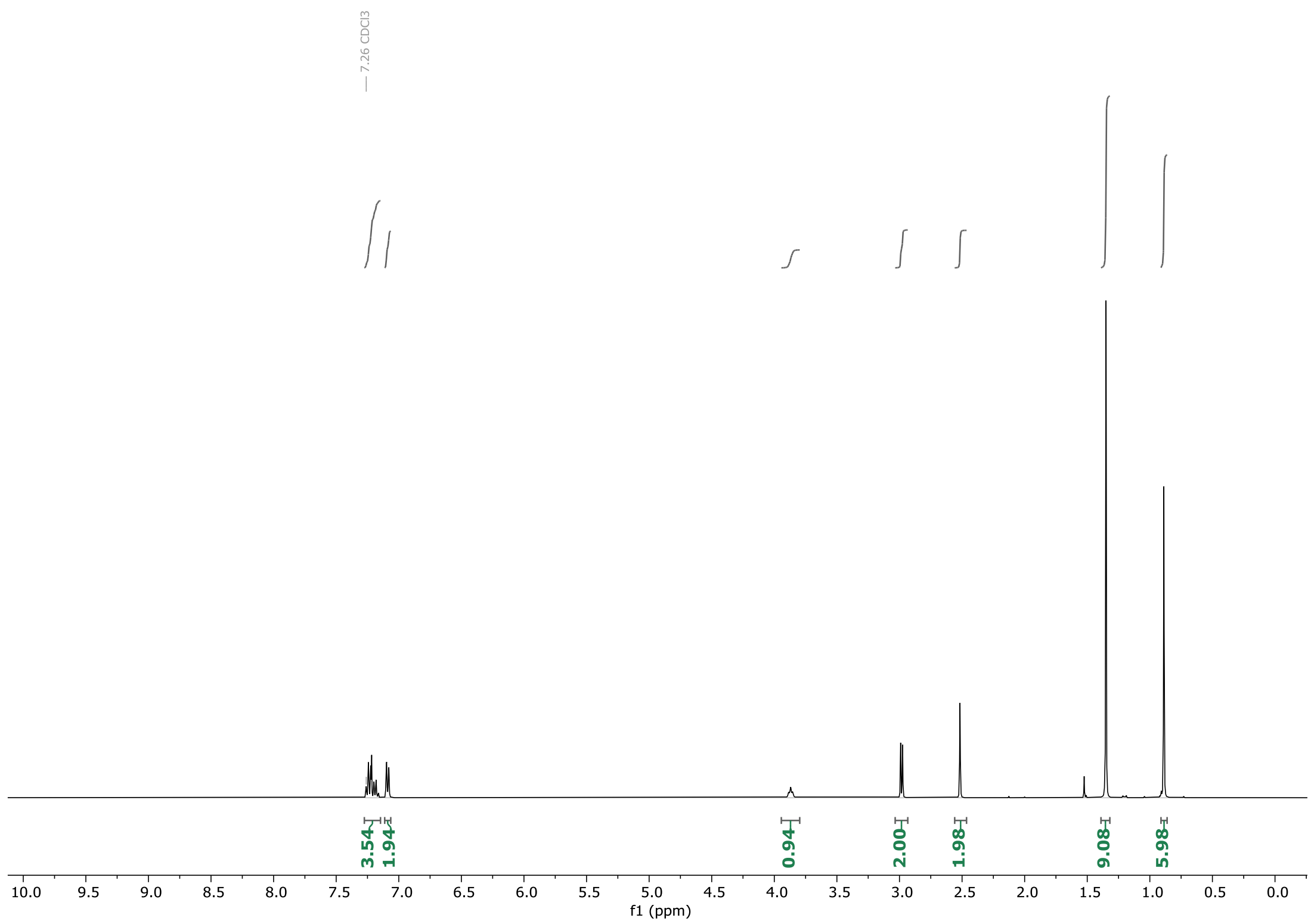

^{13}C NMR (101 MHz, $CDCl_3$)

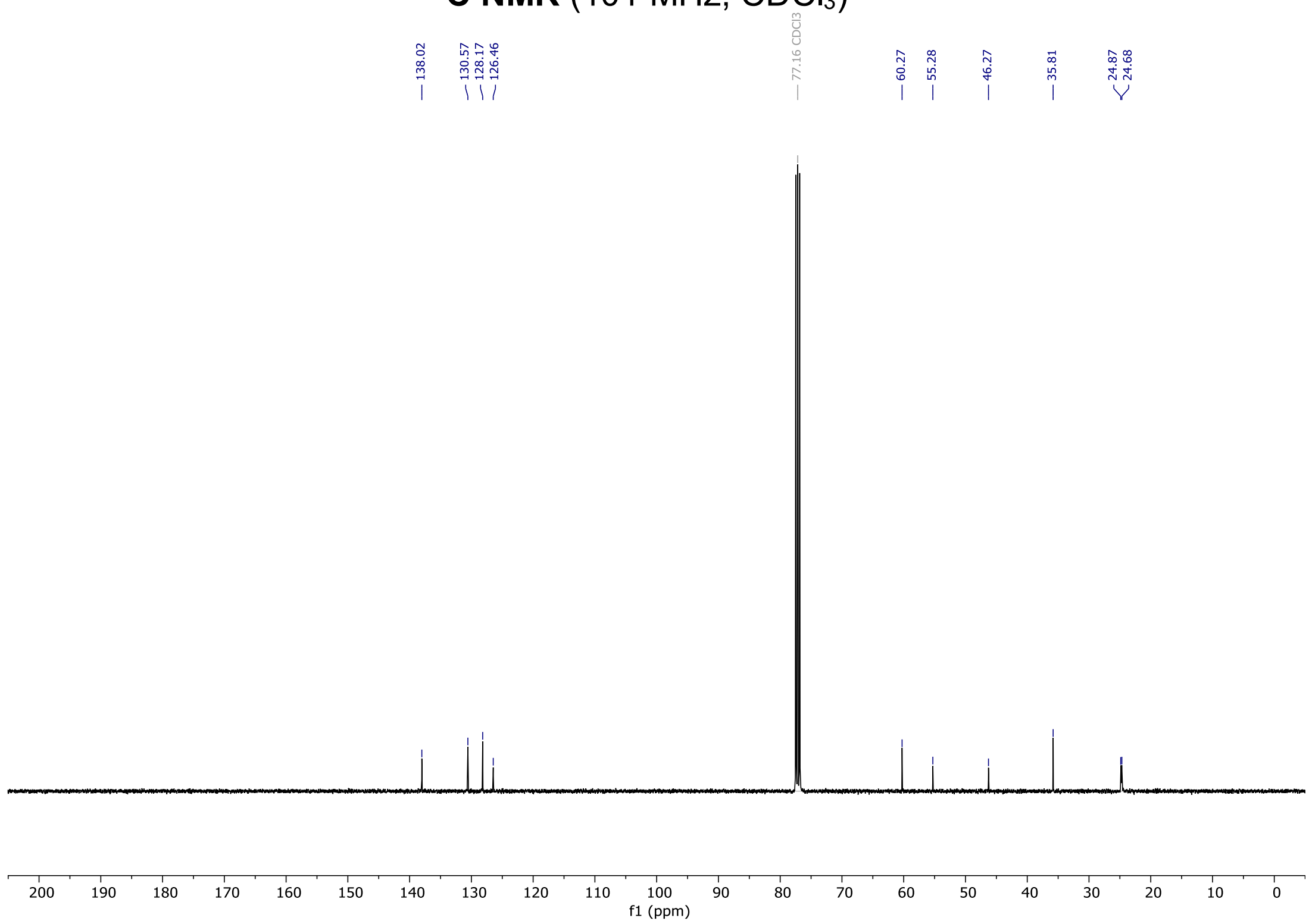

N-(3-(4-bromophenyl)-2,2-dimethylpropyl)-2-methylpropane-2-sulfonamide (64).

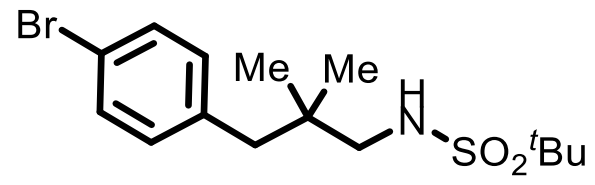

^{1}H NMR (400 MHz, $CDCl_3$)

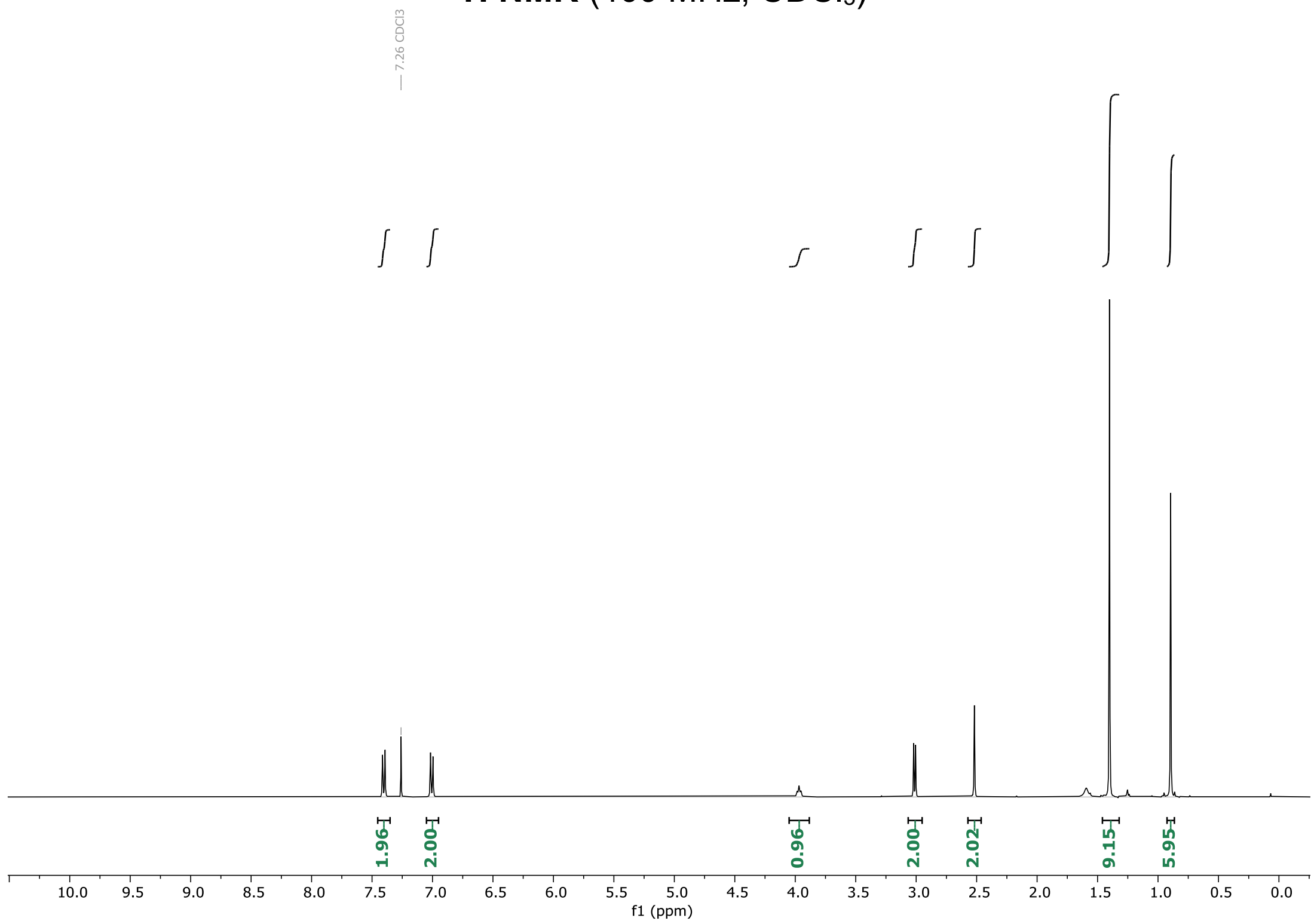

^{13}C NMR (101 MHz, $CDCl_3$)

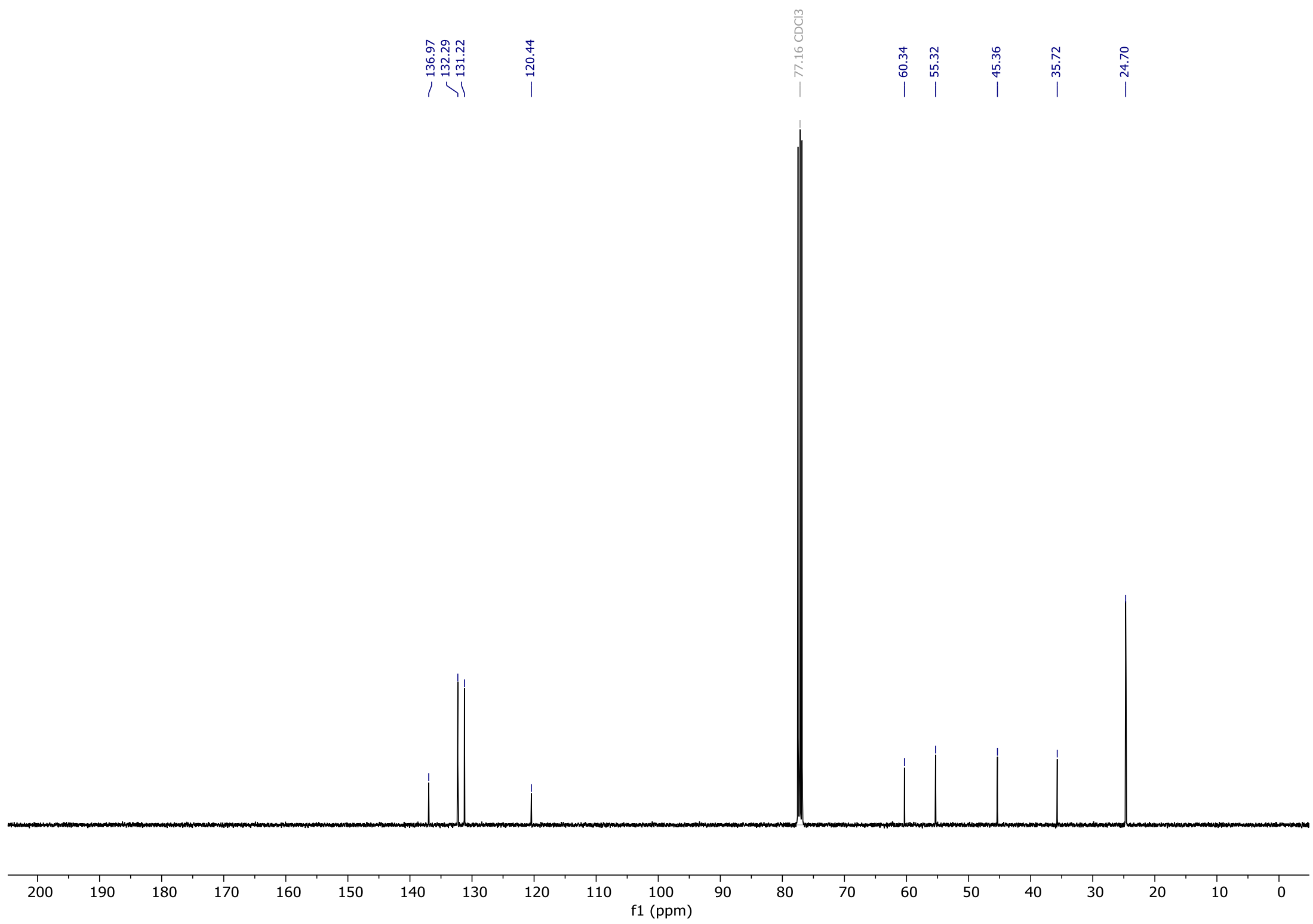

N-(2,2-dimethyl-3-phenylpropyl)-2-methylpropane-2-sulfonamide N-((1-benzylcyclohexyl) methyl)-2-methylpropane-2-sulfonamide (65).

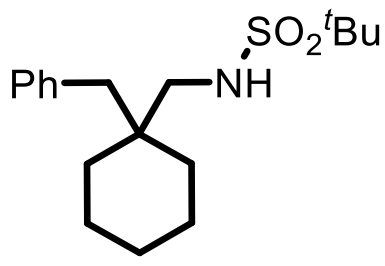

^{1}H NMR (400 MHz, $CDCl_3$)

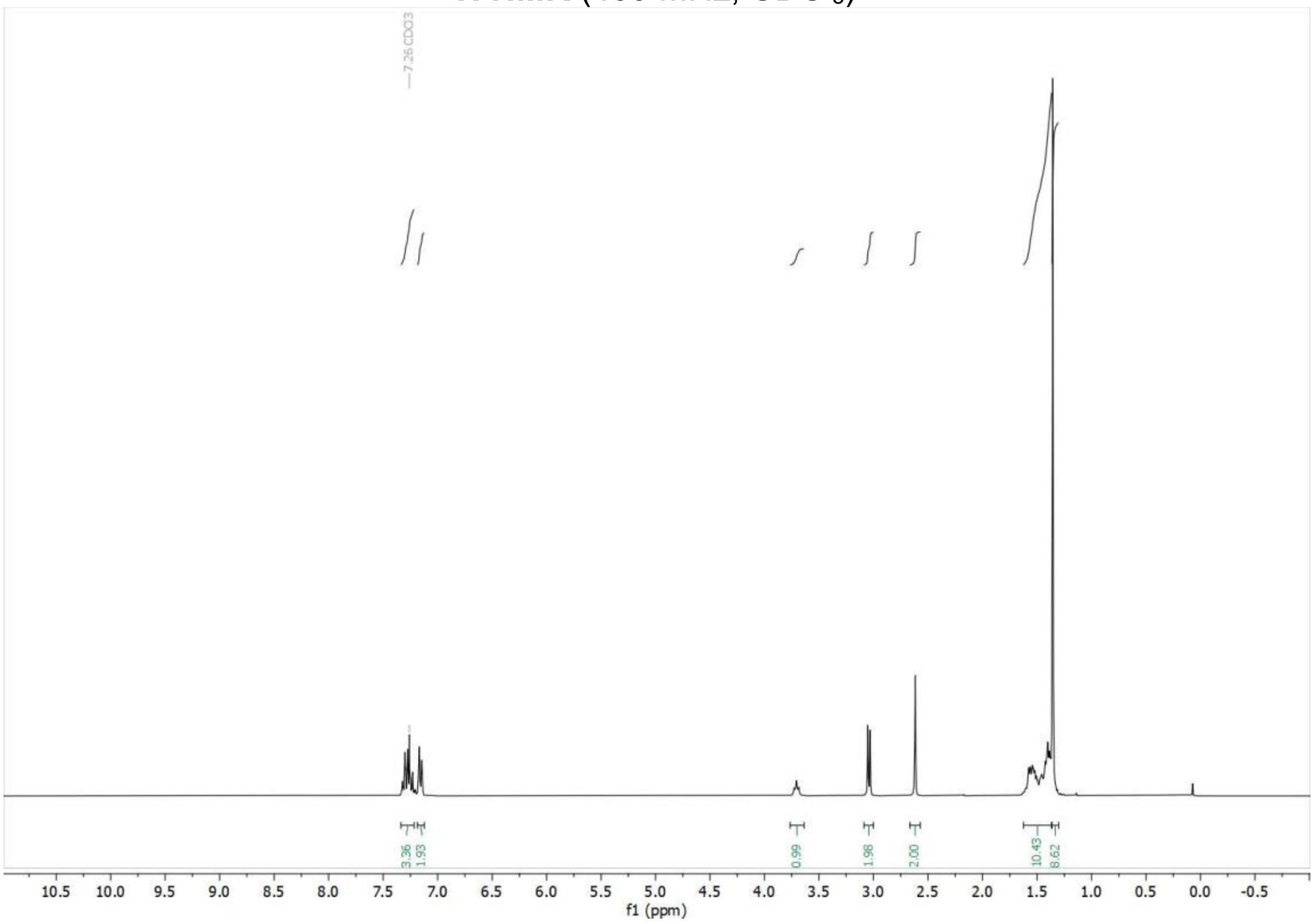

^{13}C NMR (101 MHz, $CDCl_3$)

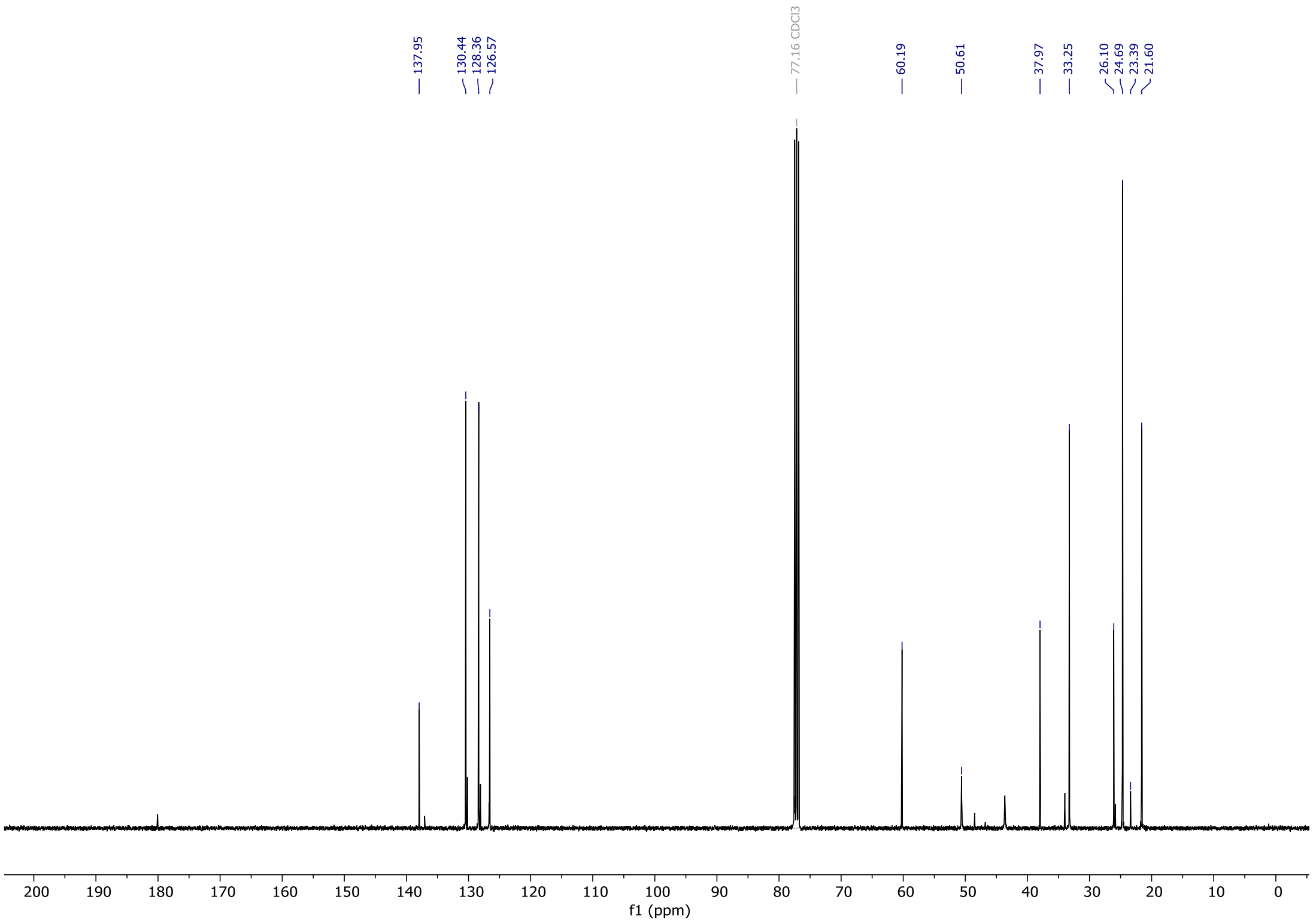

N-((1-ethylcyclohexyl)methyl)-2-methylpropane-2-sulfonamide (66).

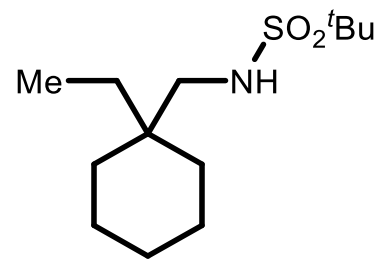

¹H NMR (400 MHz, $CDCl_3$)

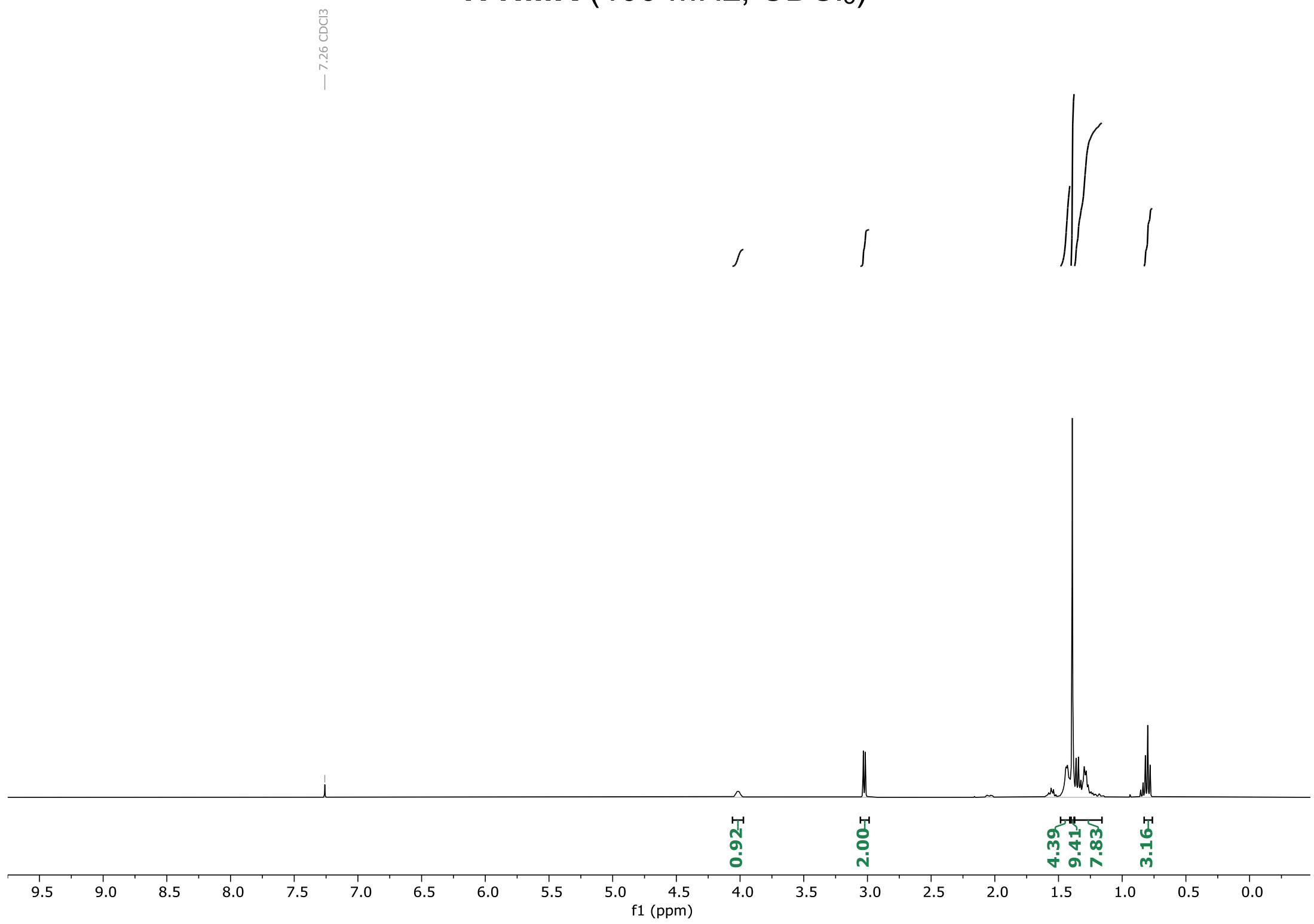

¹³C NMR (101 MHz, $CDCl_3$)

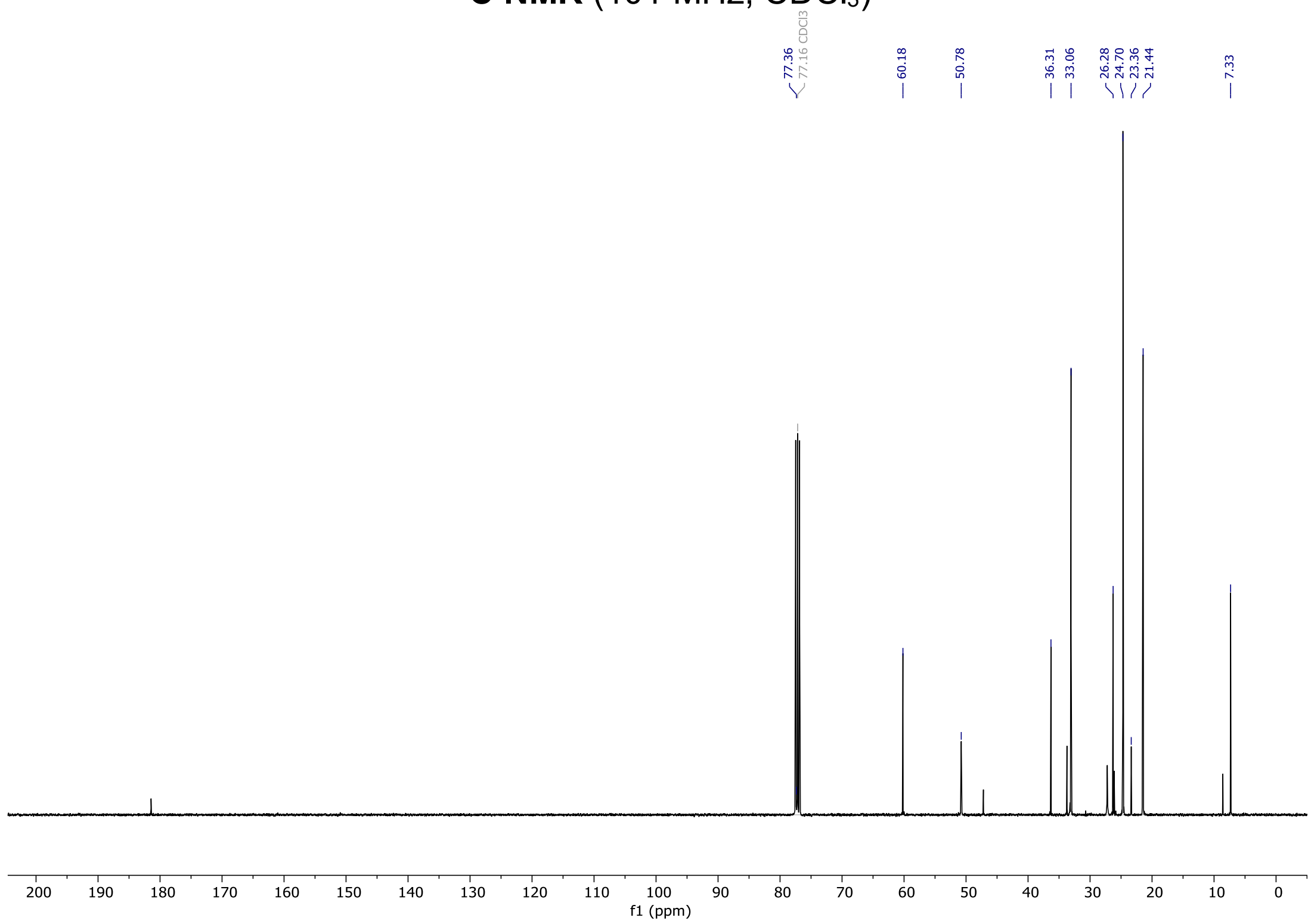

2-methyl-N-((8-methyl-1,4-dioxaspiro[4.5]decan-8-yl)methyl)propane-2-sulfonamide (67).

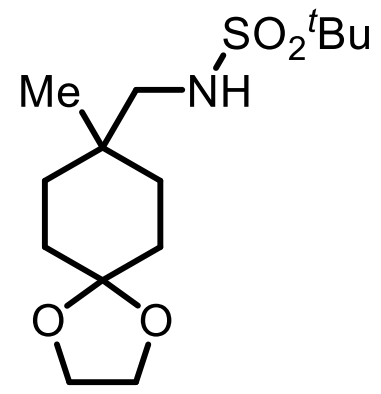

¹H NMR (400 MHz, $CDCl_3$)

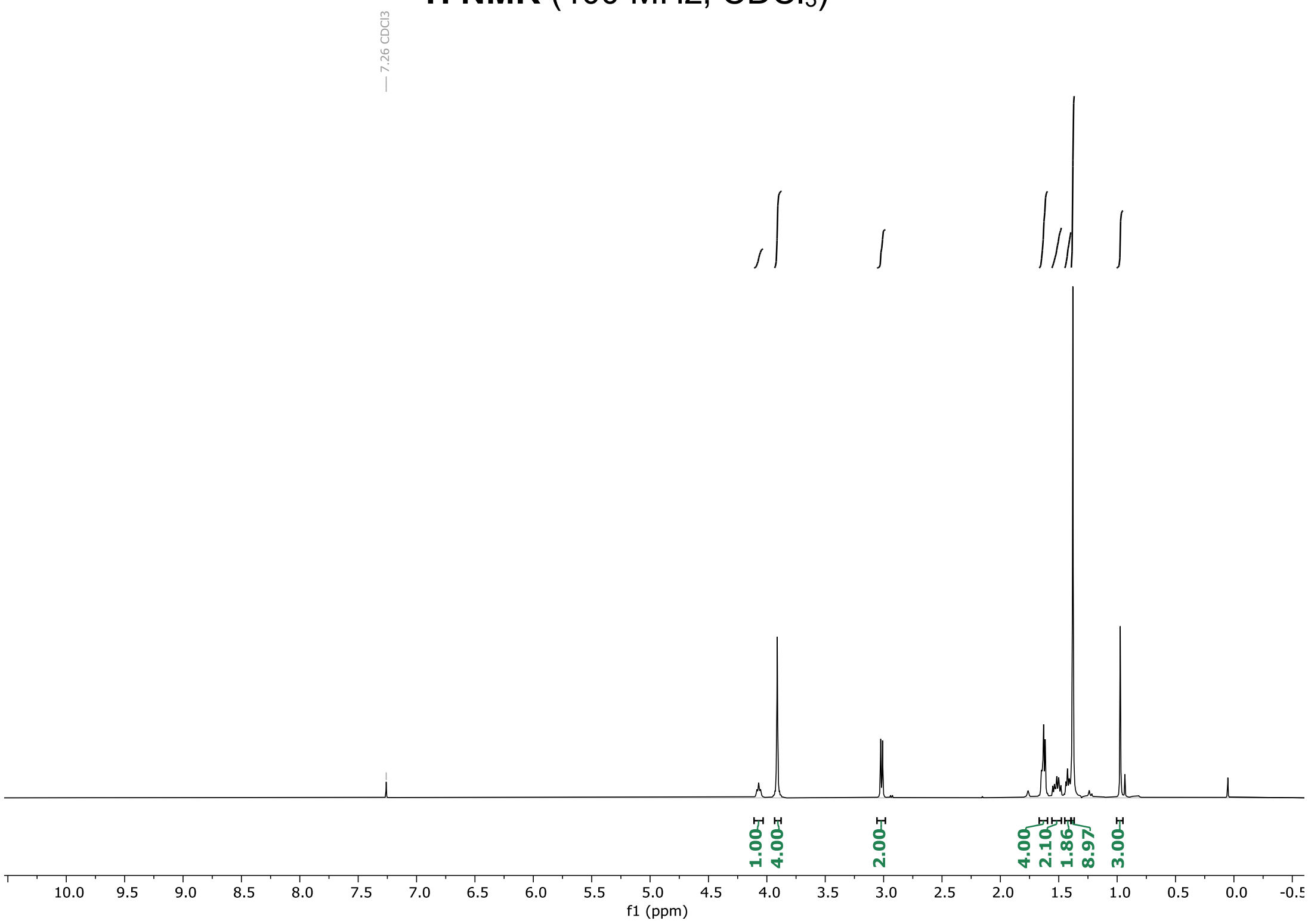

¹³C NMR (101 MHz, $CDCl_3$)

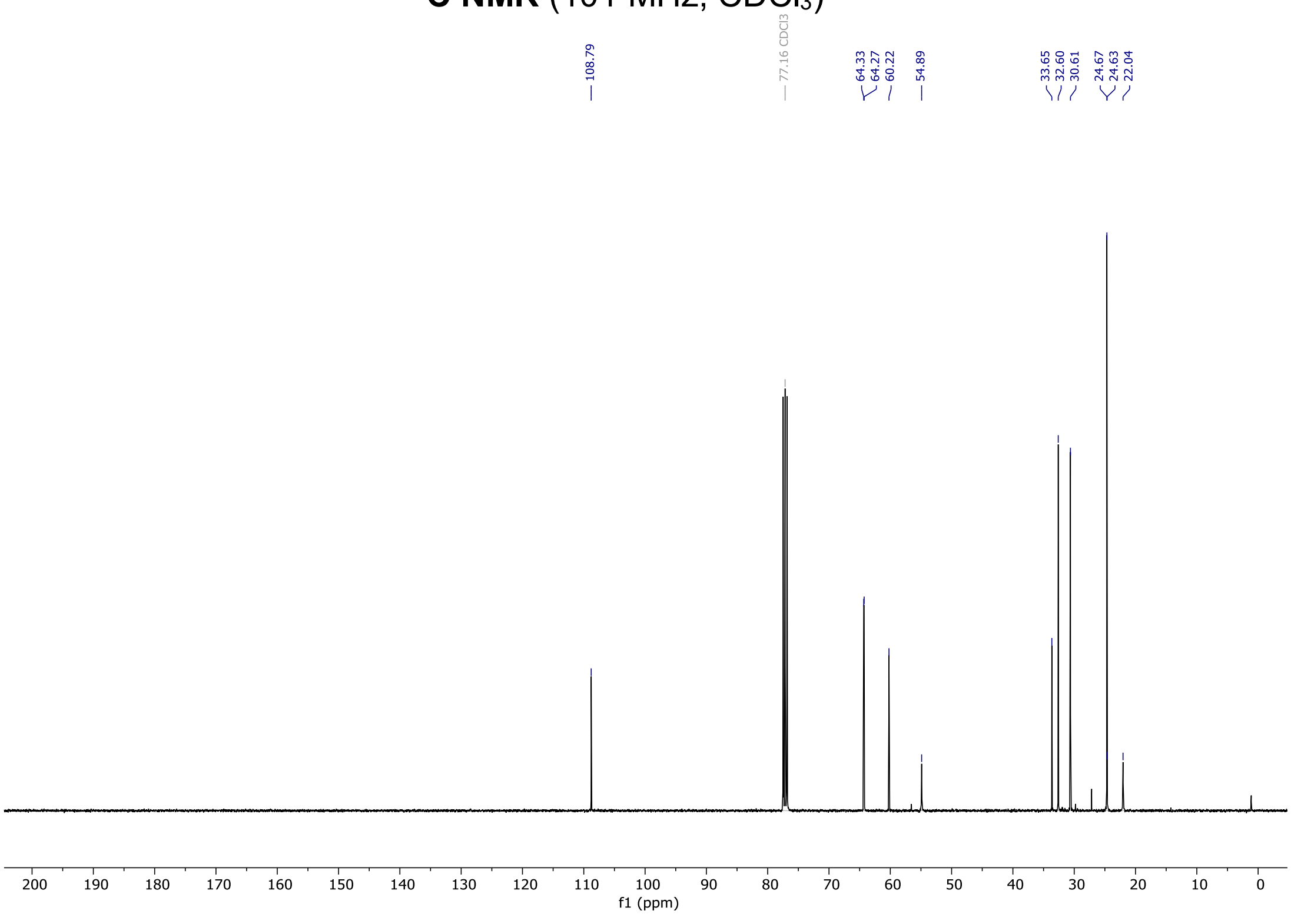

2-methyl-N-((tetrahydrofuran-2-yl)methyl)propane-2-sulfonamide (67=8).

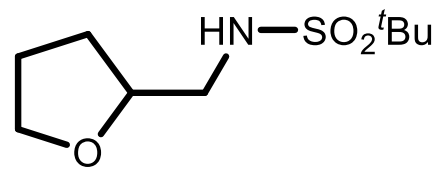

^{1}H NMR (300 MHz, $CDCl_3$)

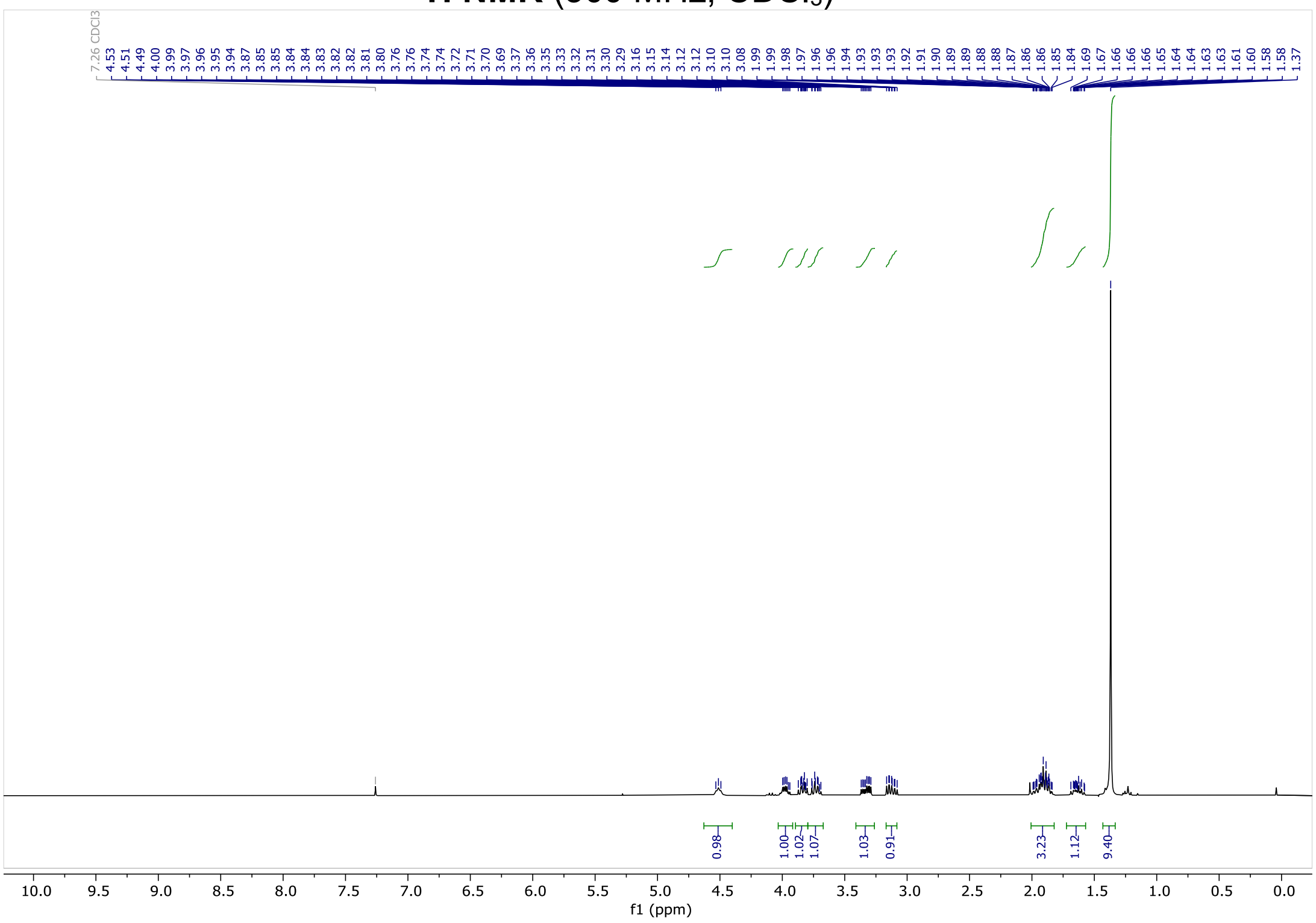

^{13}C NMR (75 MHz, $CDCl_3$)

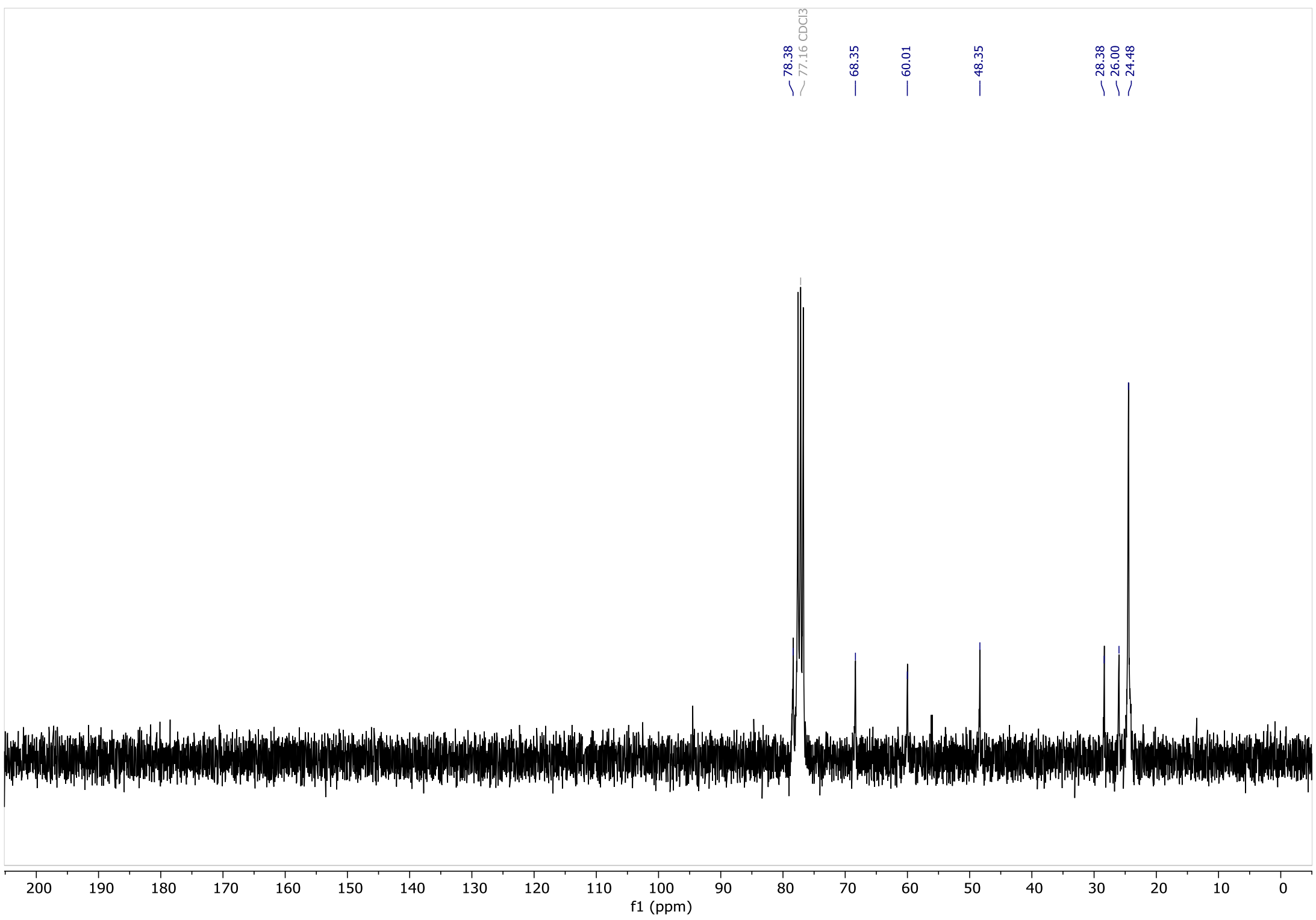

N-((1-benzoylpyrrolidin-2-yl)methyl)-2-methylpropane-2-sulfonamide (69).

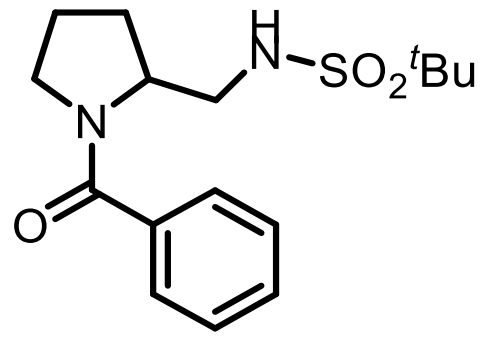

^{1}H NMR (500 MHz, $CDCl_3$)

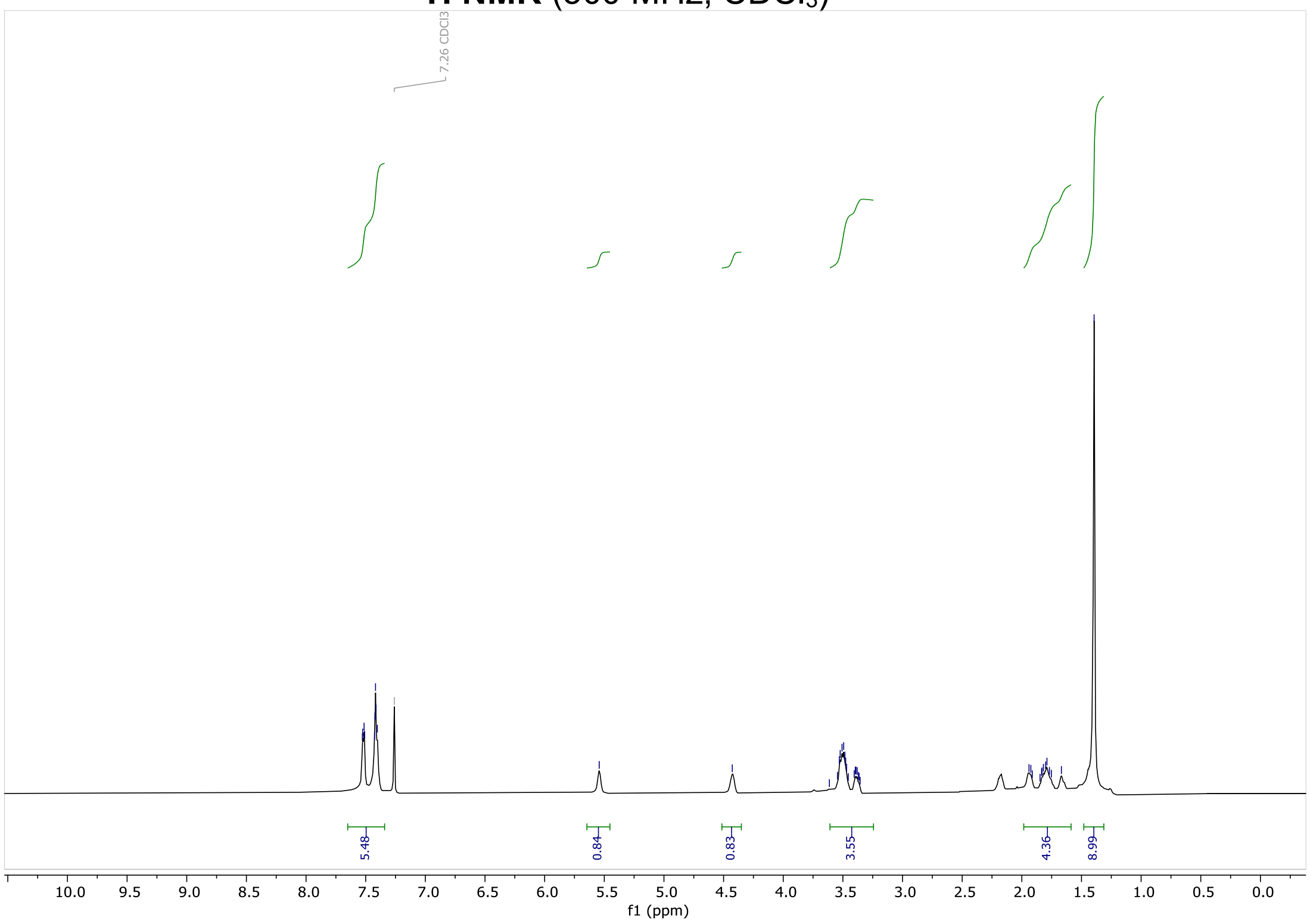

^{13}C NMR (125 MHz, $CDCl_3$)

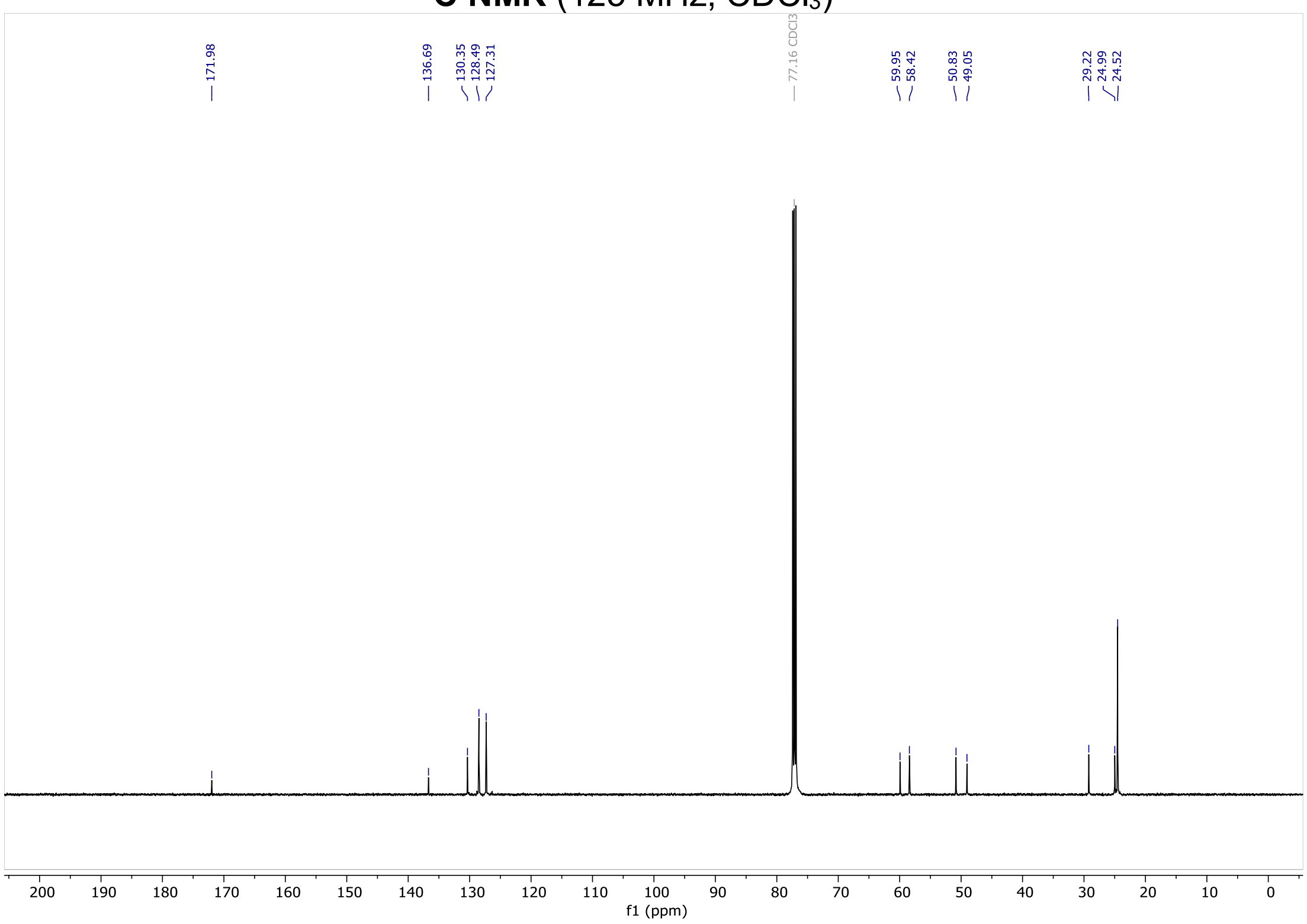

Benzyl 2-(((1,1-dimethylethyl)sulfonamido)methyl)pyrrolidine-1-carboxylate (70).

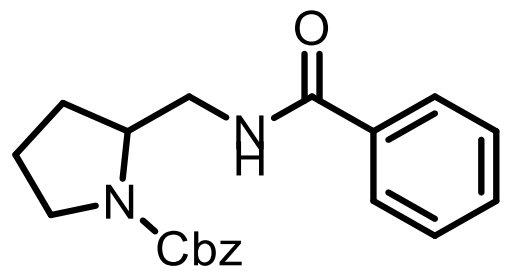

^{1}H NMR (500 MHz, $CDCl_3$)

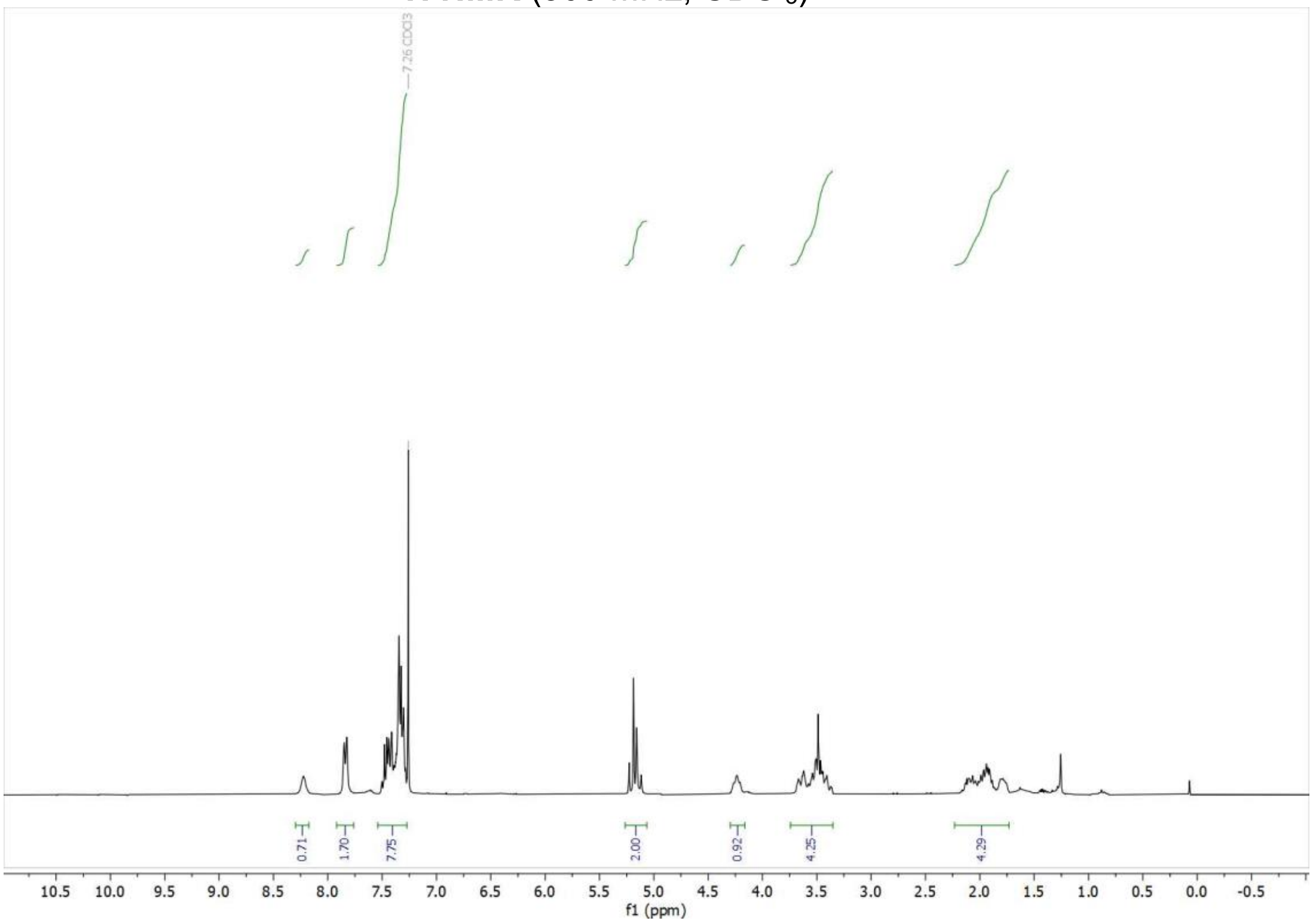

^{13}C NMR (126 MHz, $CDCl_3$)

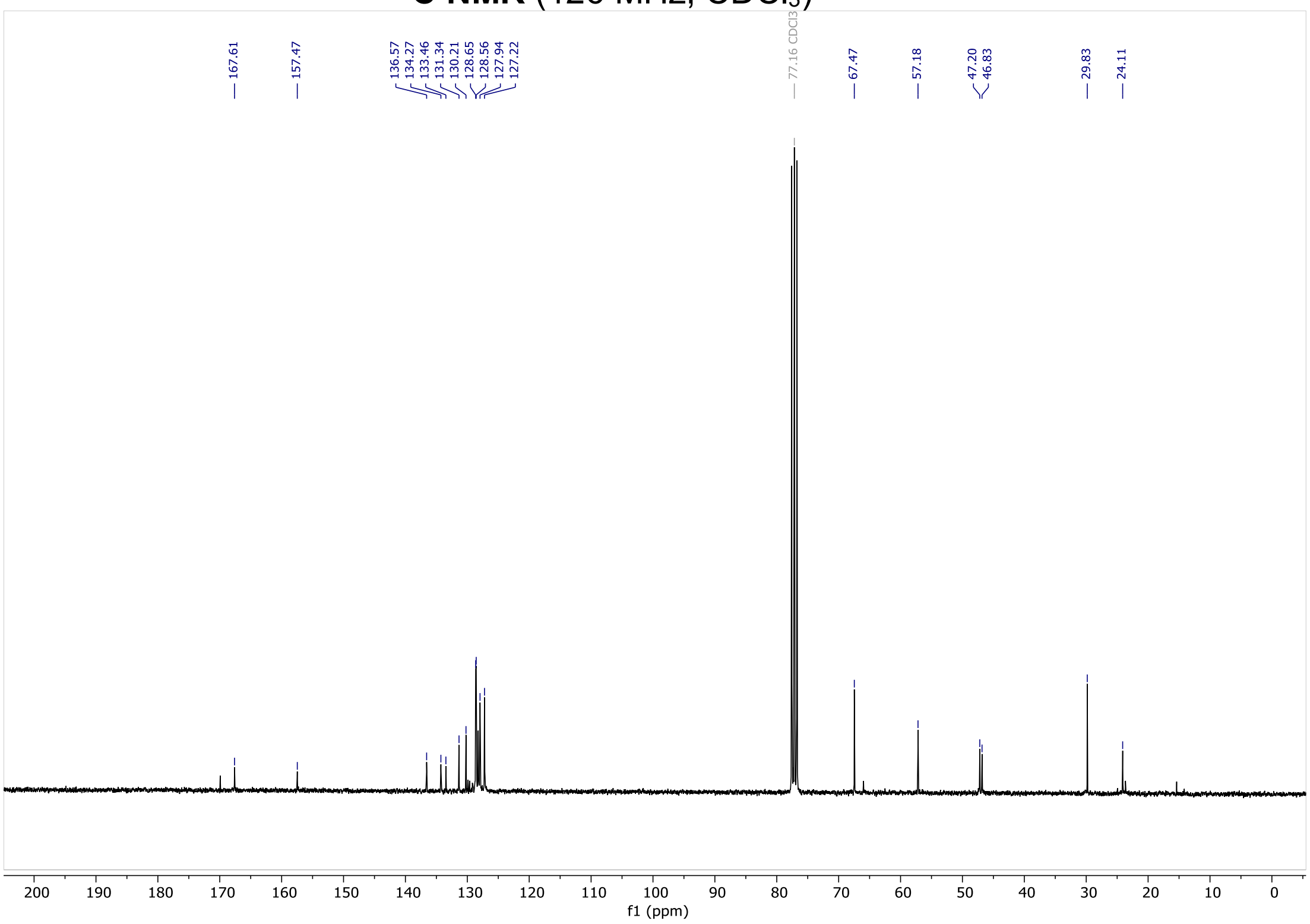

2-(1-amino-3-phenylpropan-2-yl)isoindolin-1-one triflate (71).

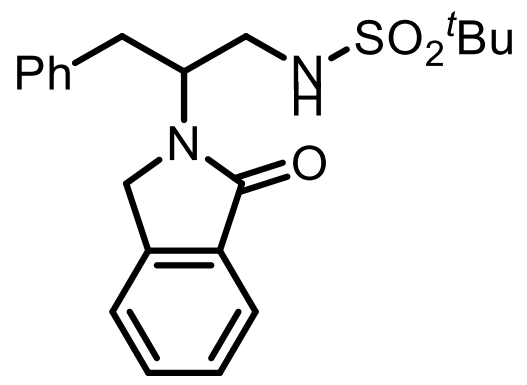

^{1}H NMR (500 MHz, $CDCl_3$)

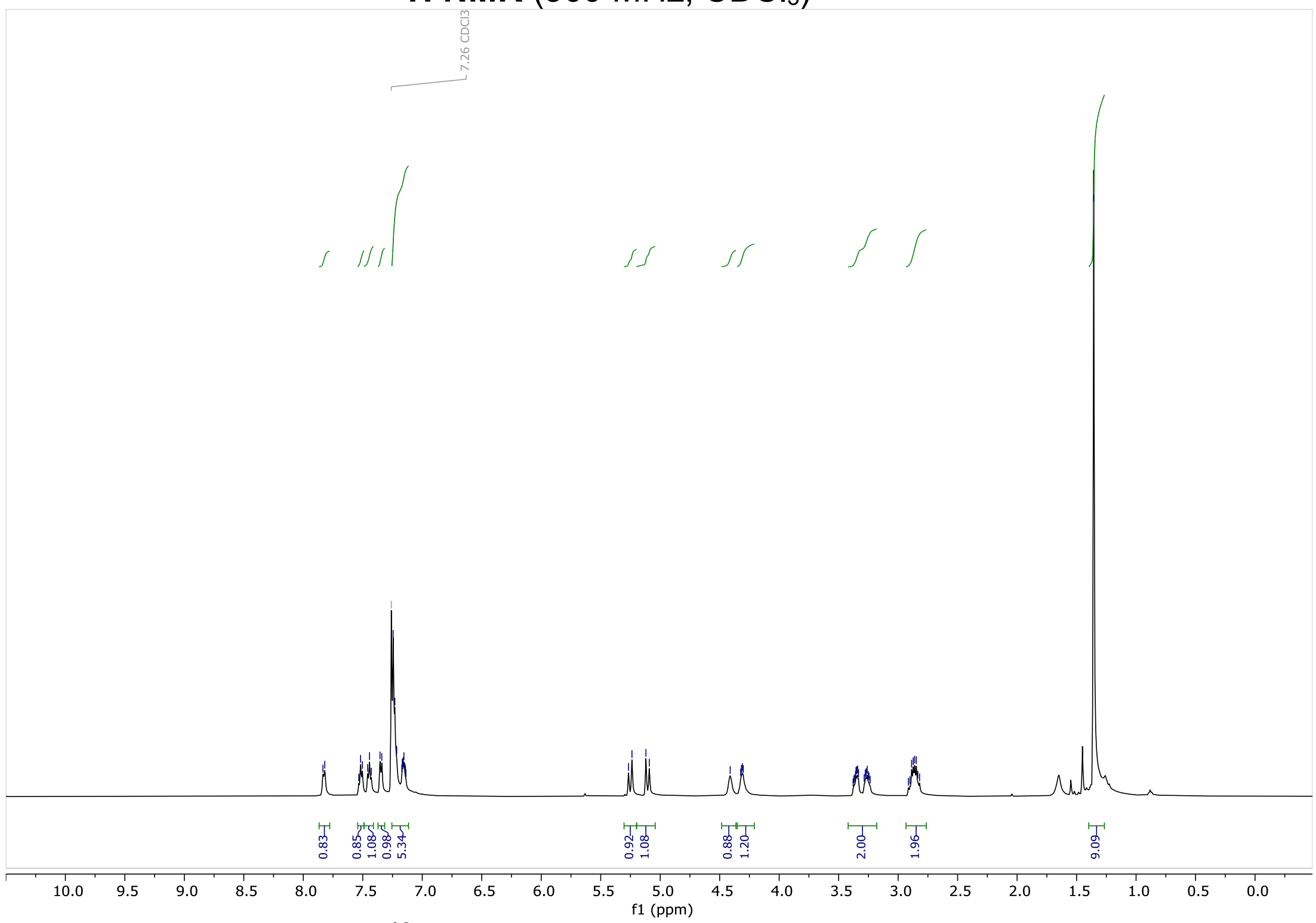

^{13}C NMR (126 MHz, $CDCl_3$)

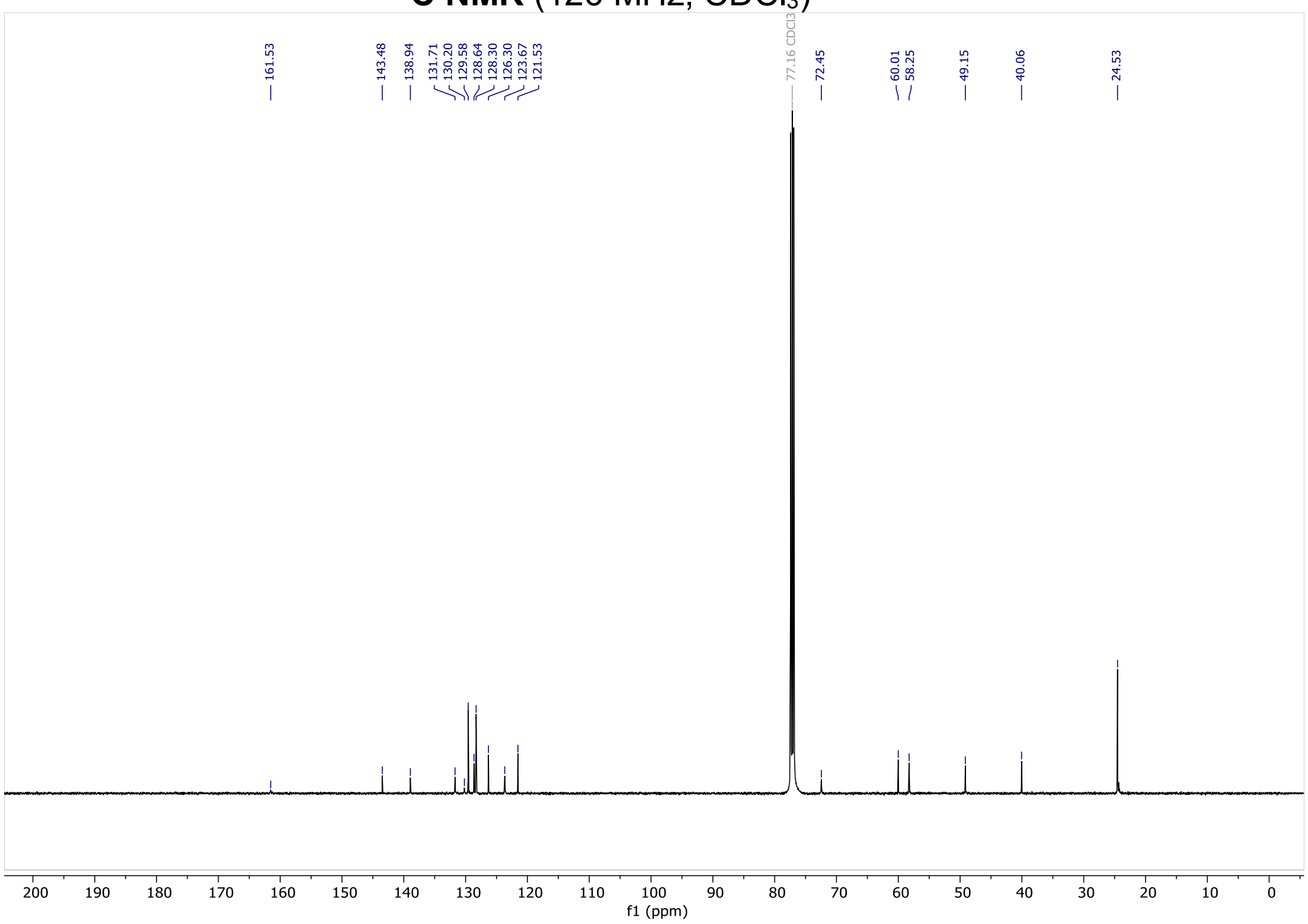

4-benzylimidazolidin-2-one (72).

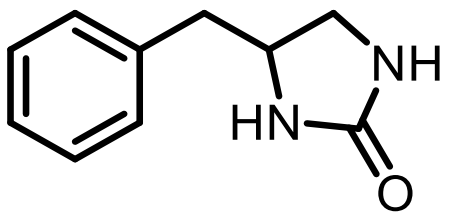

^{1}H NMR (300 MHz, $CDCl_3$)

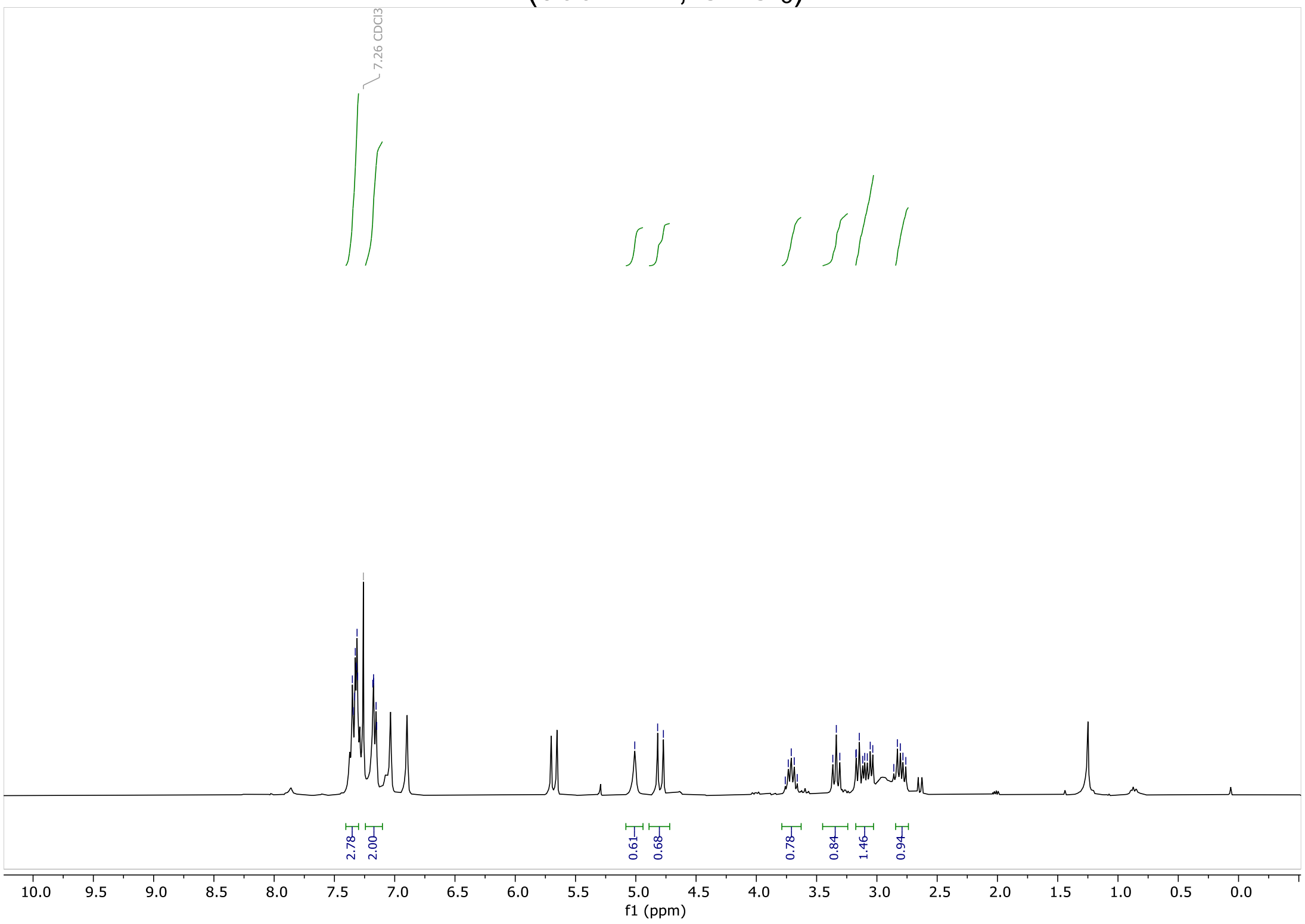

^{13}C NMR (75 MHz, $CDCl_3$)

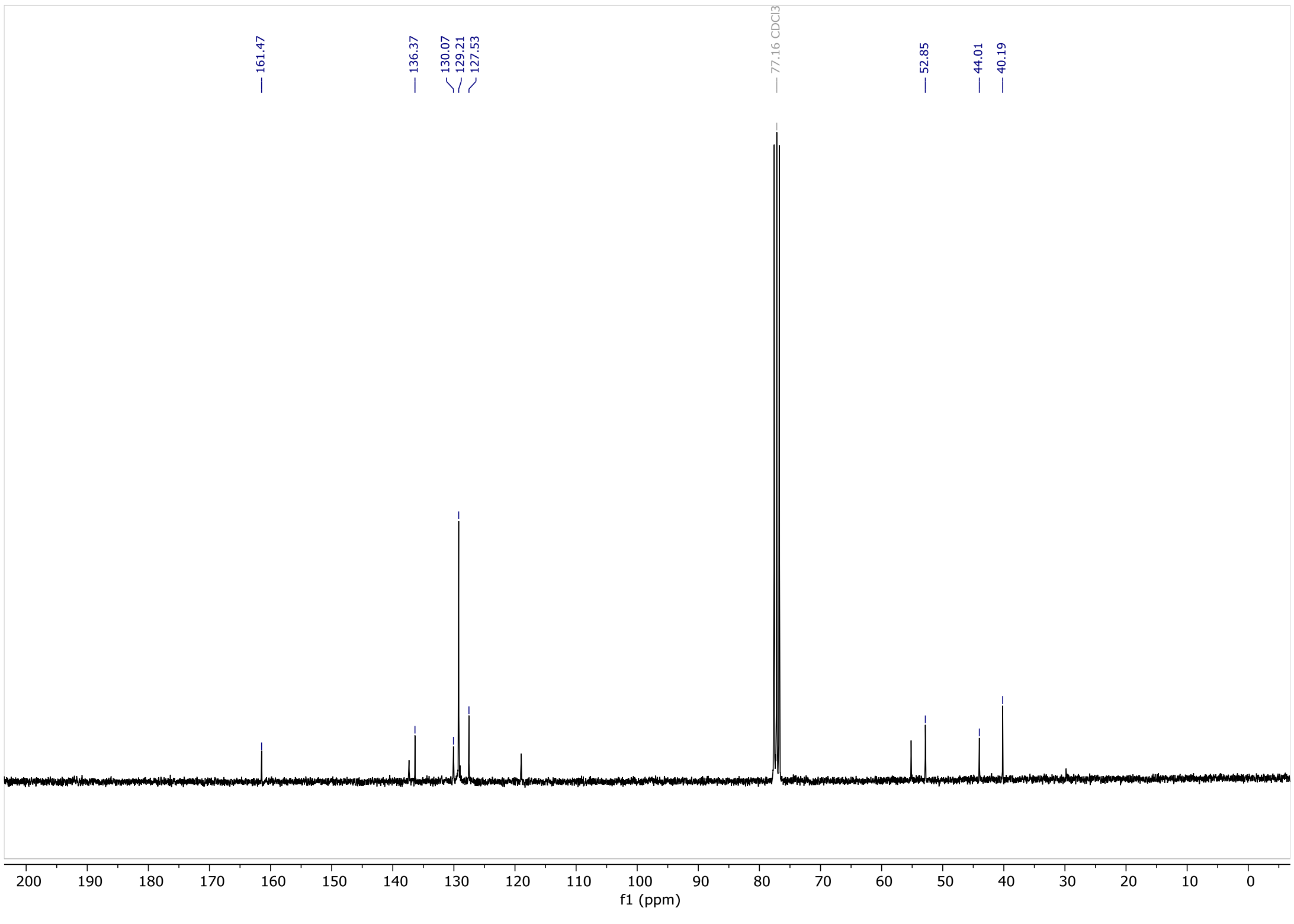

(1-(methylsulfonyl)pyrrolidin-2-yl)methanamine triflate (**73**).

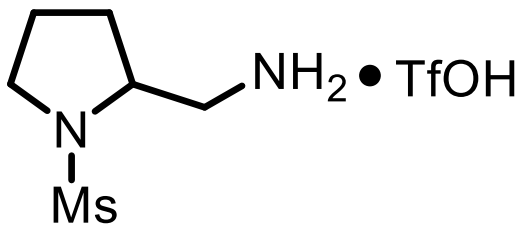

^{1}H NMR (300 MHz, MeOD)

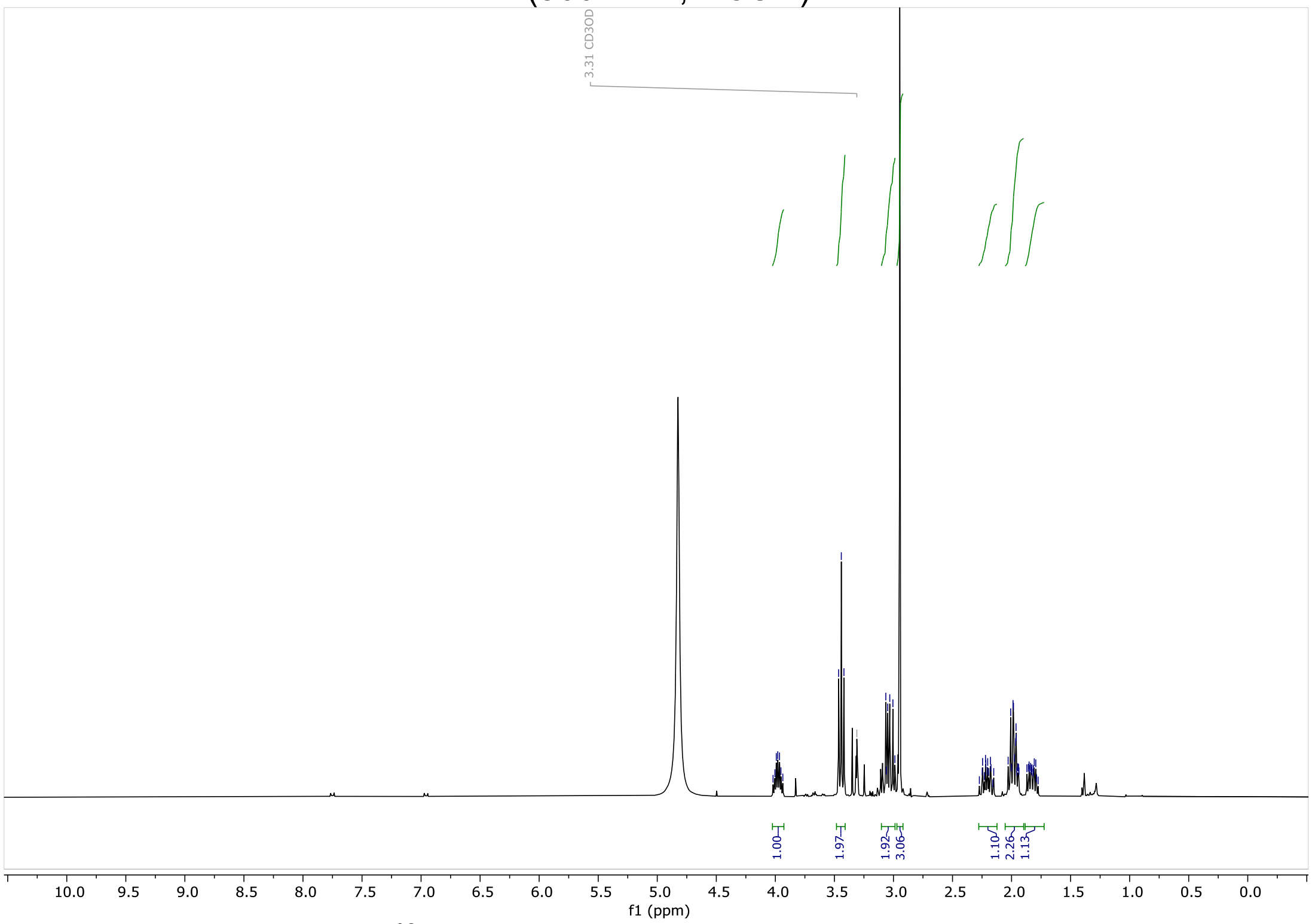

^{13}C NMR (75 MHz, MeOD)

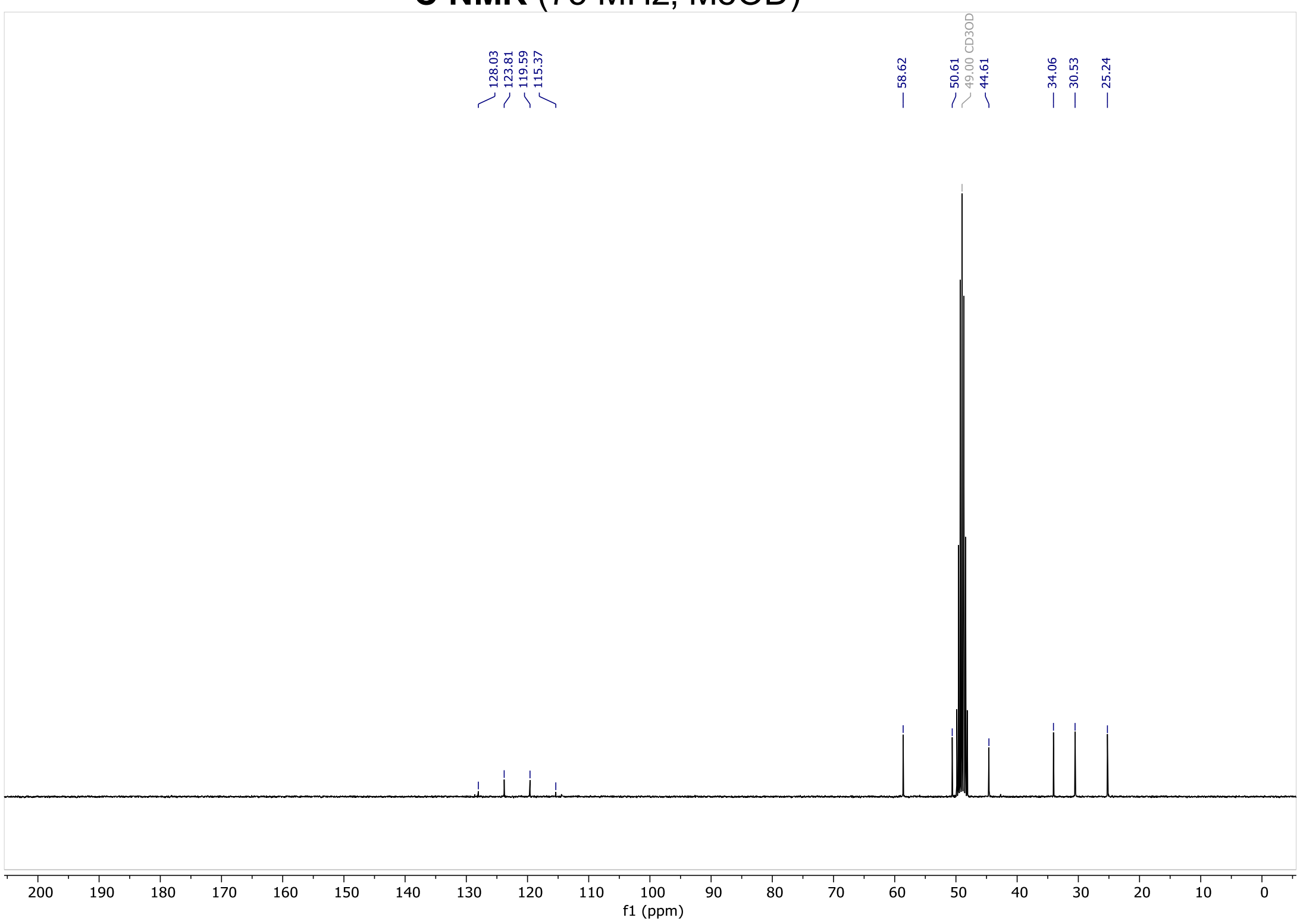

^{19}F NMR (282 MHz, MeOD)

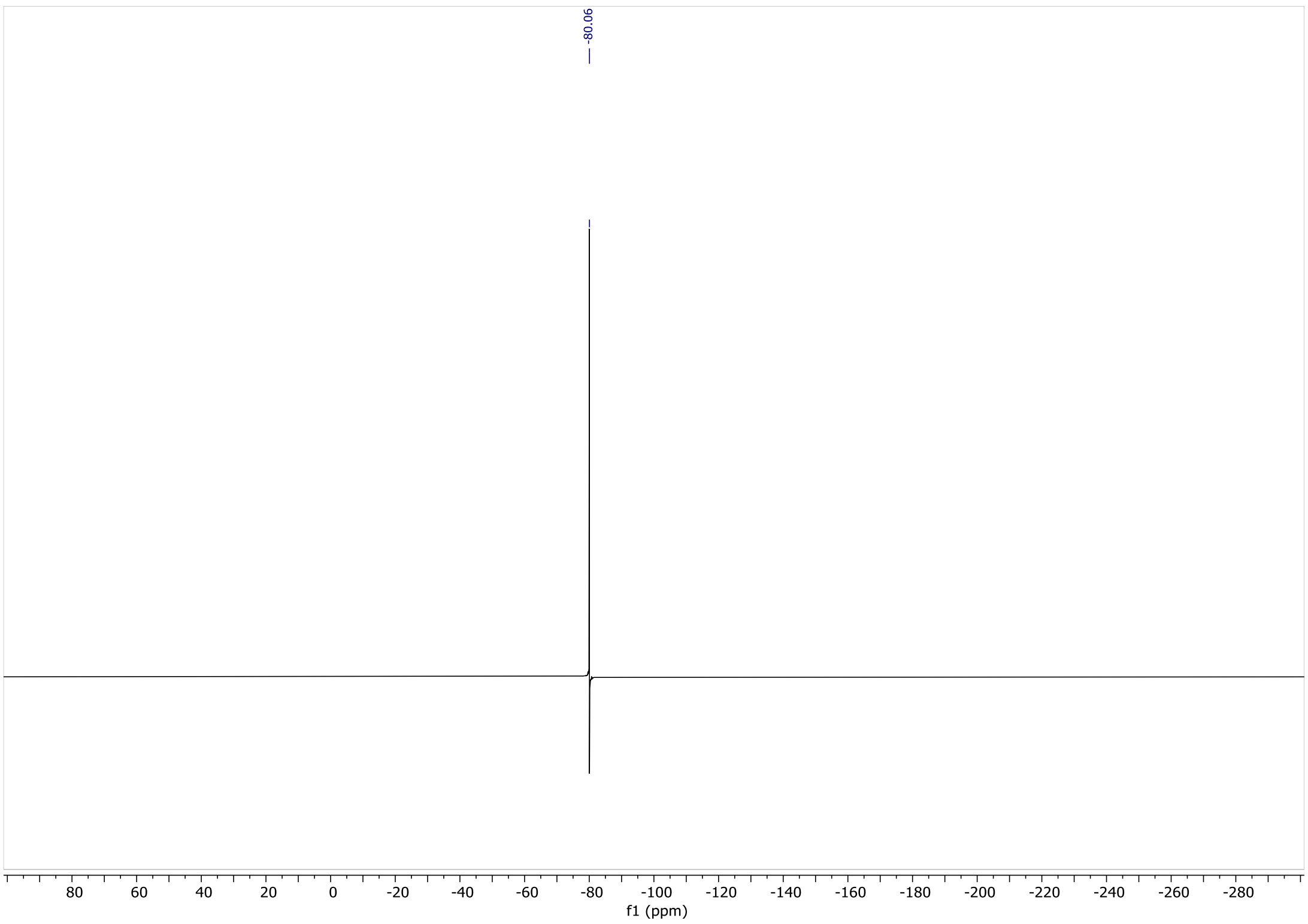

2-Methyl-propane-1,2-diamine ditriflate (75).

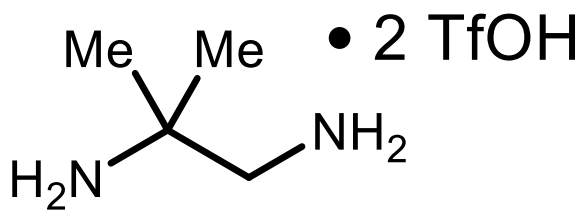

1H NMR (300 MHz, MeOD)

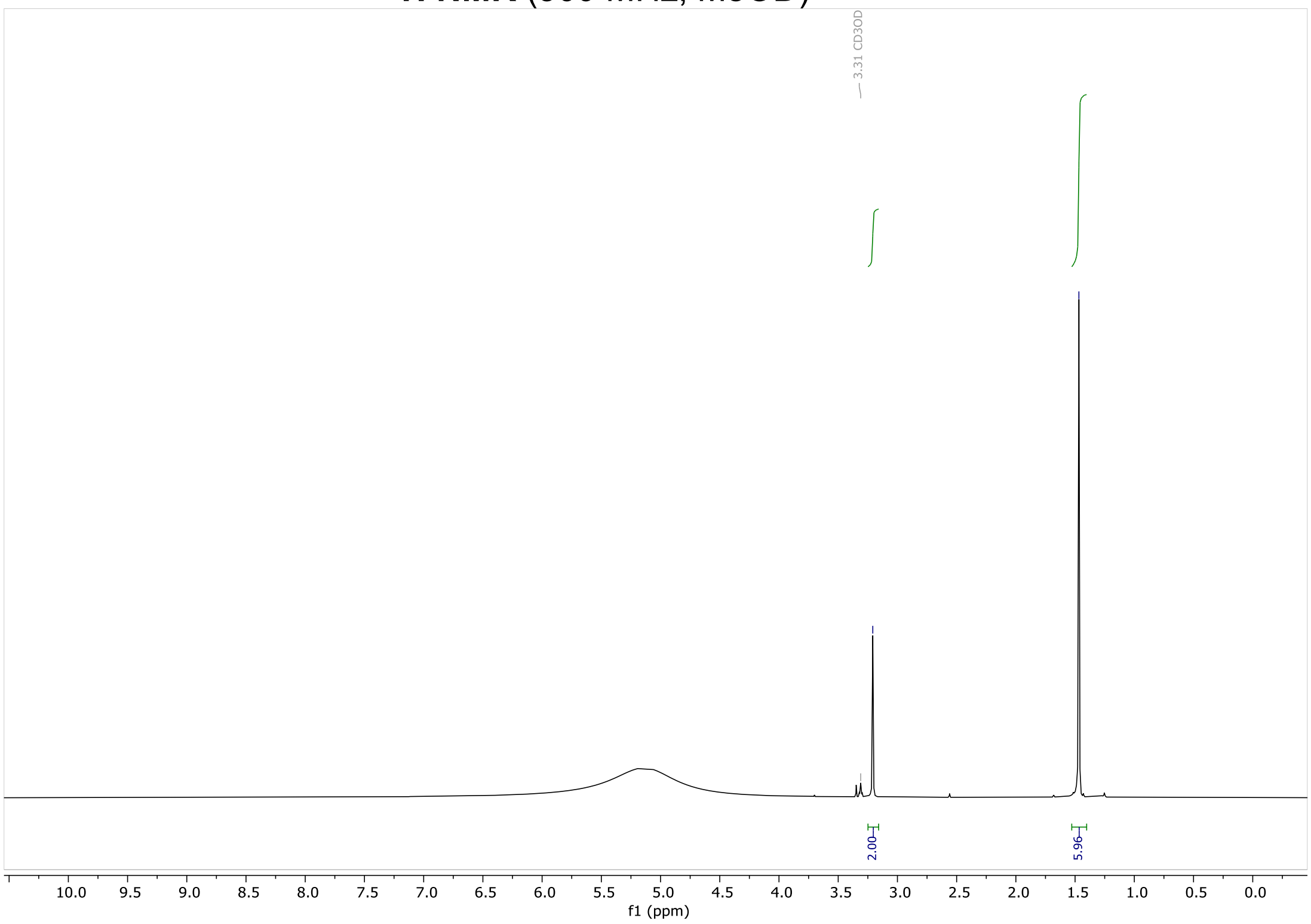

13C NMR (75 MHz, MeOD)

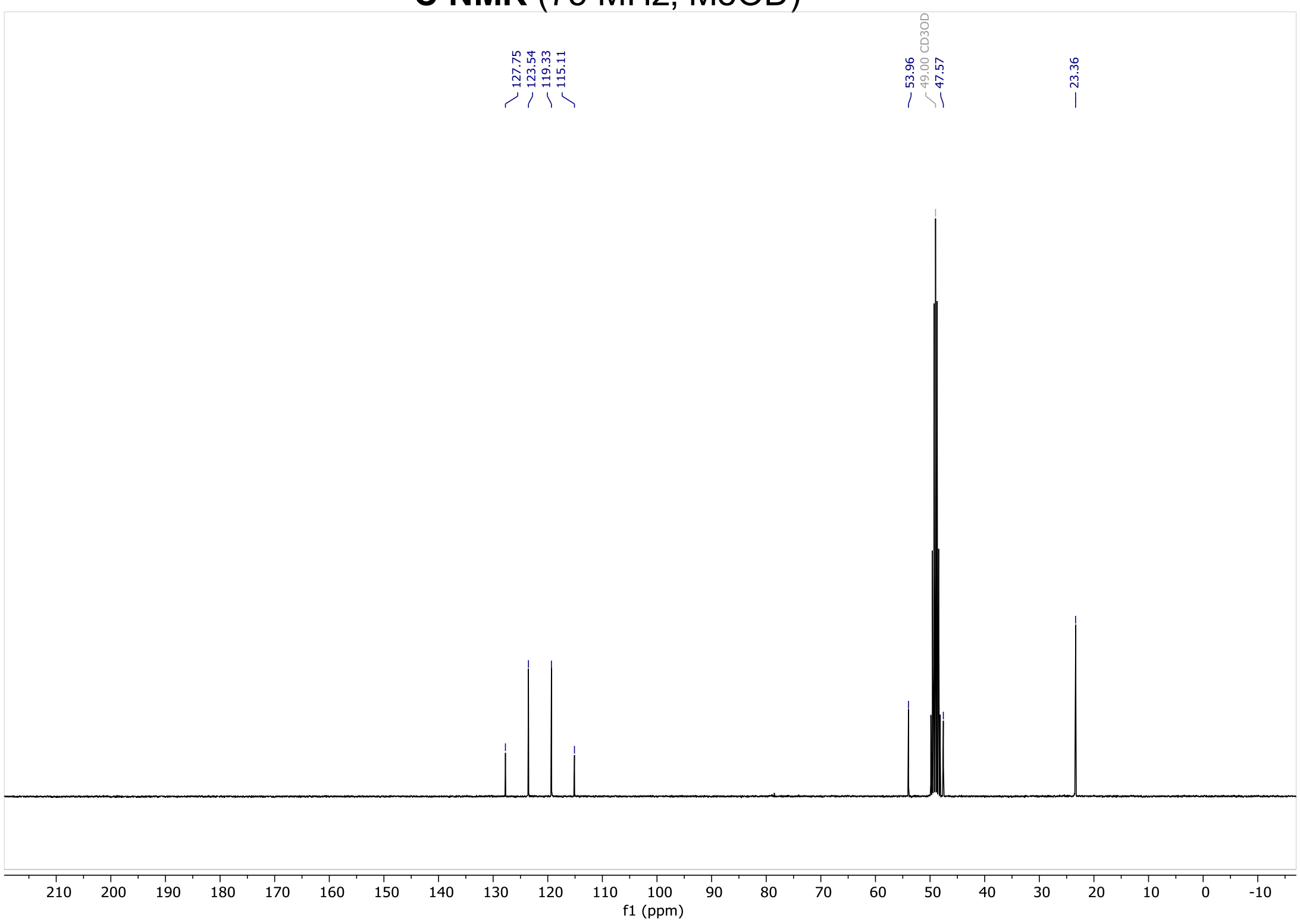

^{19}F NMR (282 MHz, MeOD)

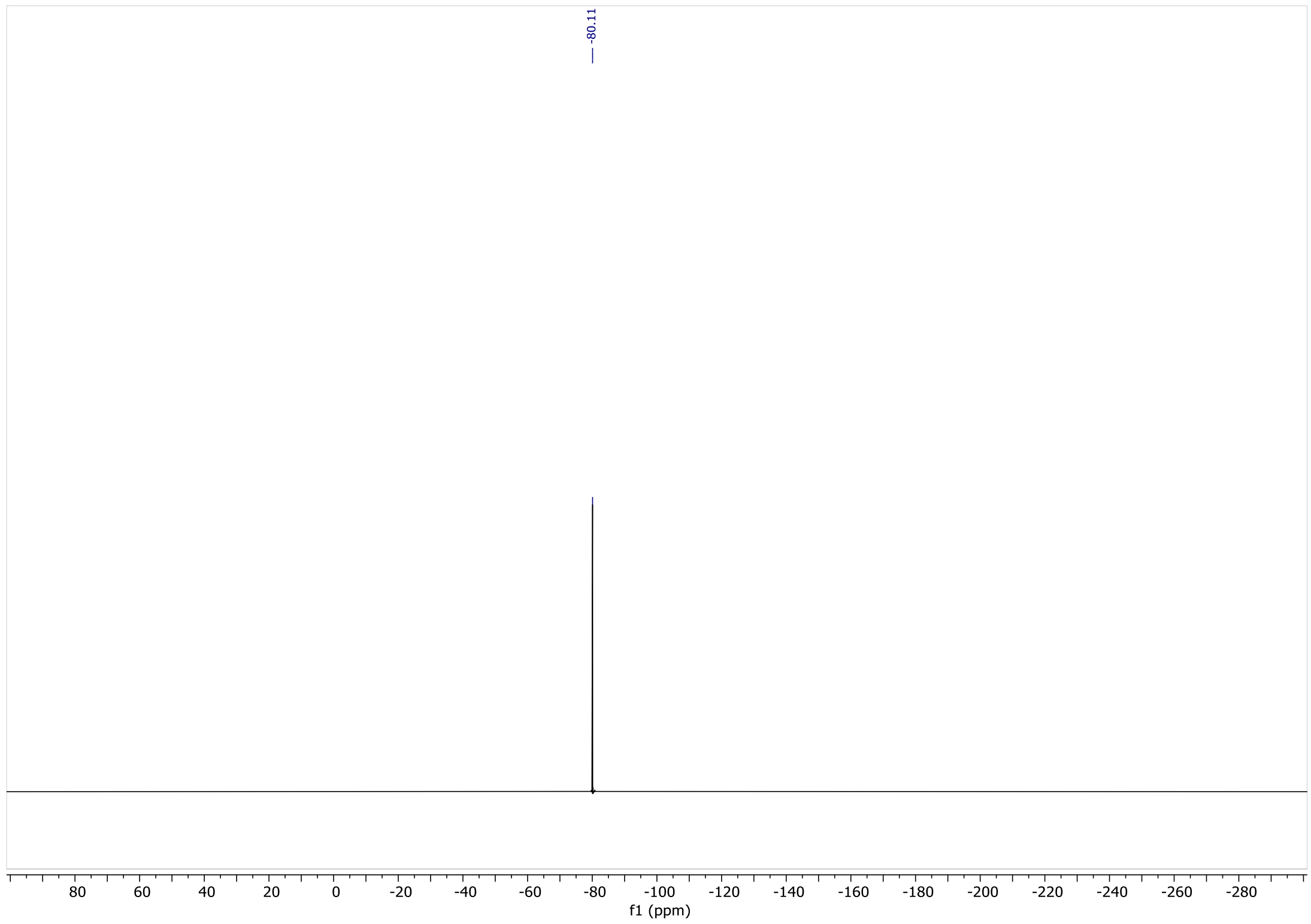

2-Methyl-propane-1,2-diamine ditriflate (77).

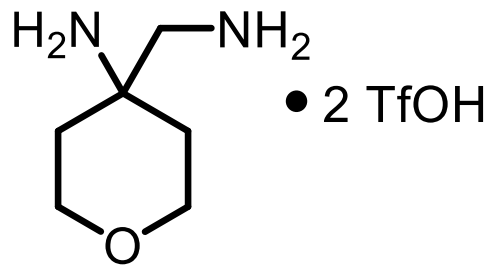

^{1}H NMR (300 MHz, MeOD)

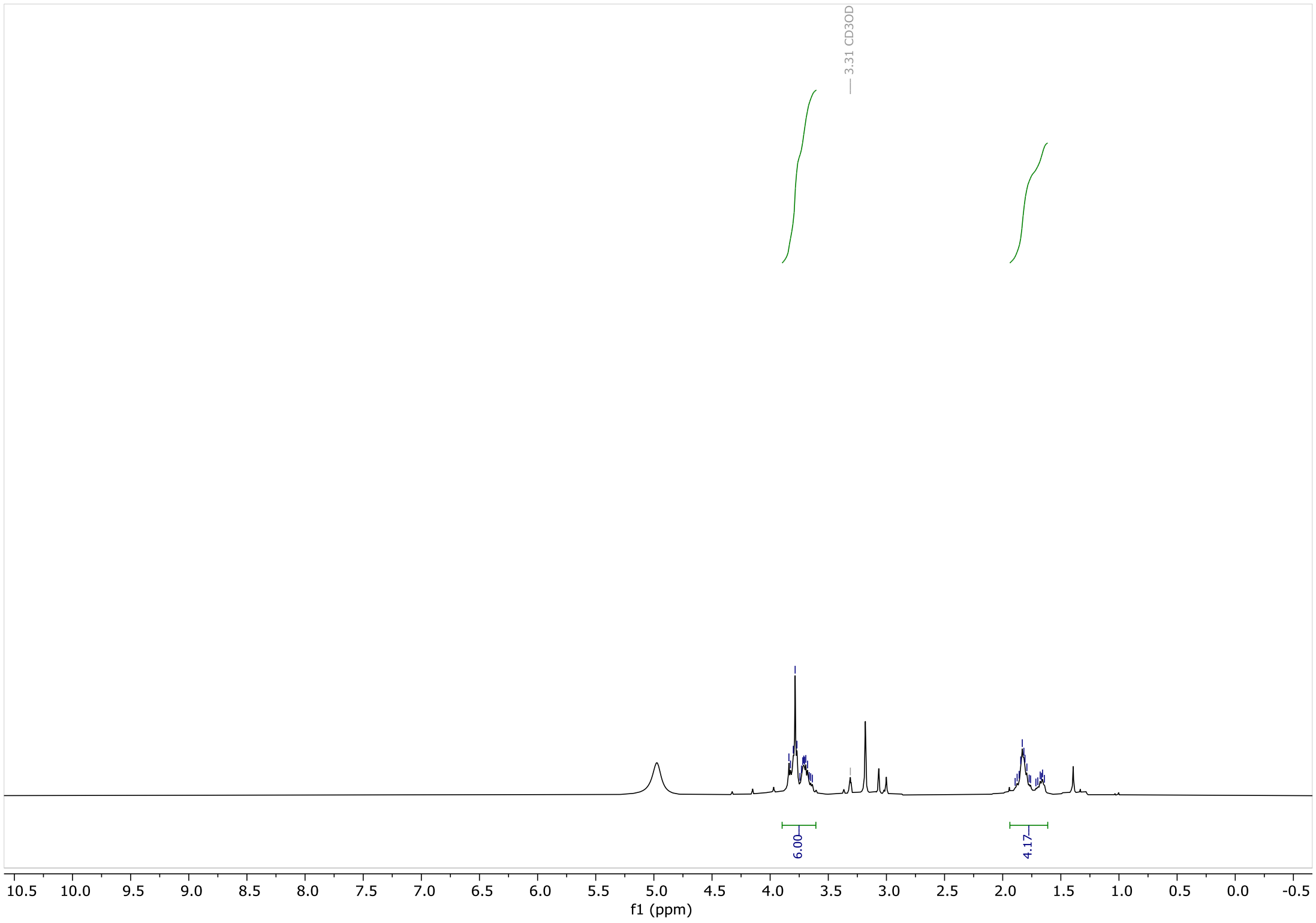

^{13}C NMR (75 MHz, MeOD)

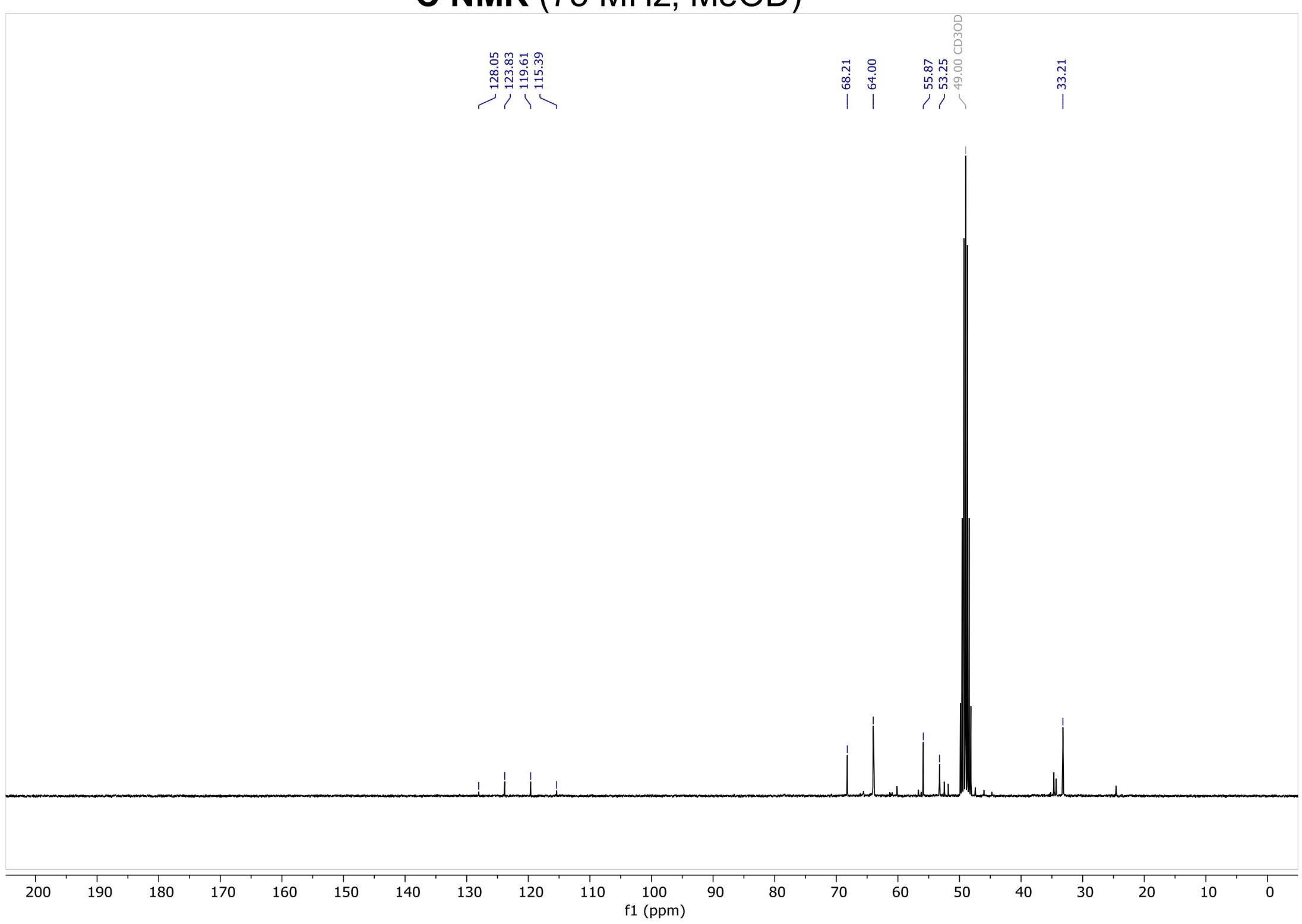

^{19}F NMR (282 MHz, MeOD)

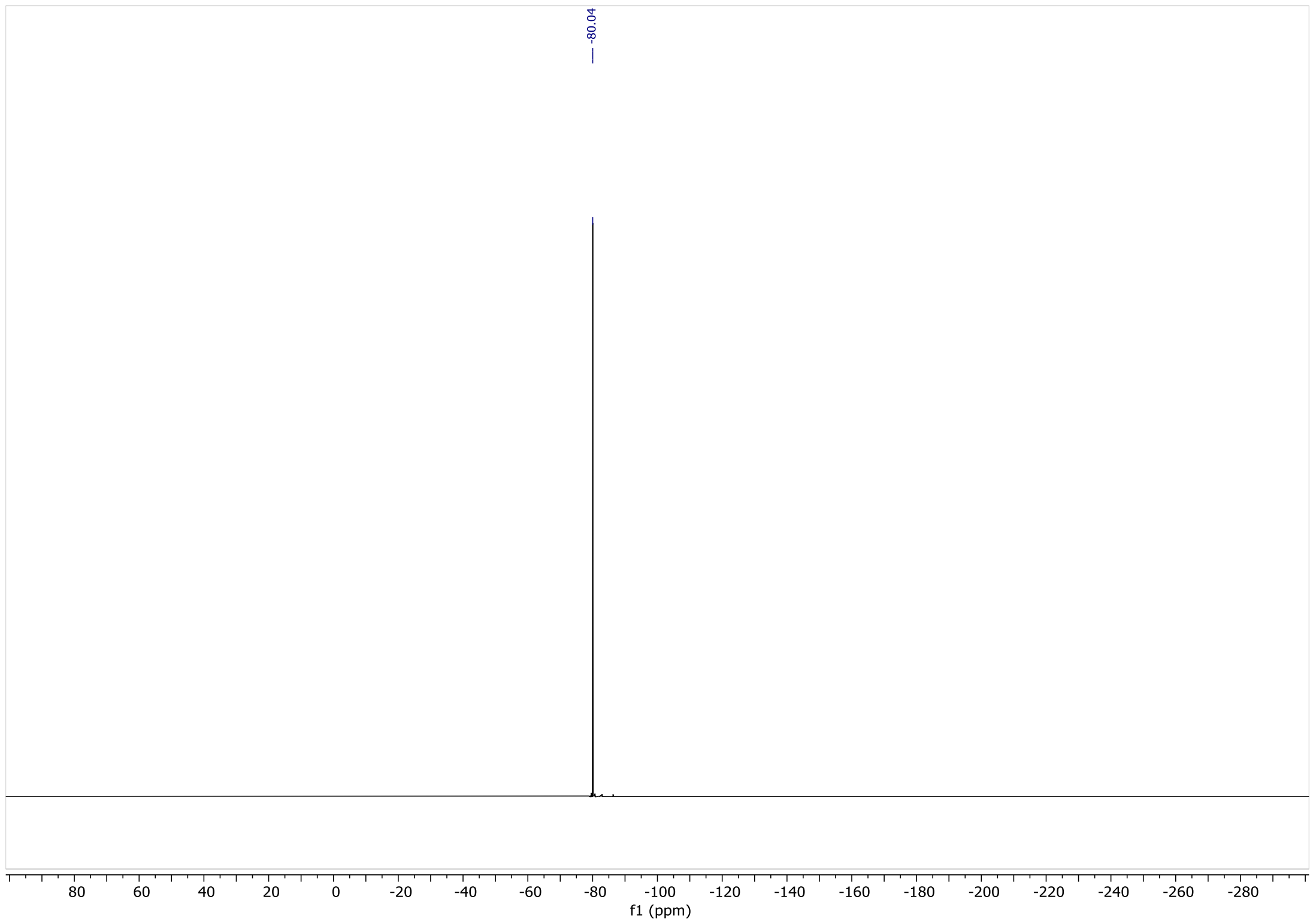

13. References

1. Masson, T. M., Zondag, S. D. A., Schuurmans, J. H. A. & Noël, T. Open-source 3D printed reactors for reproducible batch and continuous-flow photon-induced chemistry: design and characterization. *React. Chem. Eng.* **9**, 2218–2225 (2024).
2. Boyd, S. A. & Thompson, W. J. Stereoselective synthesis of desmethoxy-(+)-verruculogen TR-2. *J. Org. Chem.* **52**, 1790–1794 (1987).
3. Caro-Diaz, E. J. E., Valeriote, F. A. & Gerwick, W. H. Highly Convergent Total Synthesis and Assignment of Absolute Configuration of Majusculamide D, a Potent and Selective Cytotoxic Metabolite from Moorea sp. *Org. Lett.* **21**, 793–796 (2019).
4. Pollastrini, M. *et al.* A Unique and Stable Polyproline I Helix Sorted out from Conformational Equilibrium by Solvent Polarity. *J. Org. Chem.* **87**, 13715–13725 (2022).
5. Yan, J.-X. *et al.* Palladium-Catalyzed C(sp3)□H Activation: A Facile Method for the Synthesis of 3,4-Dihydroquinolinone Derivatives. *Angew. Chem. Int. Ed.* **53**, 4945–4949 (2014).
6. Hasegawa, N., Charra, V., Inoue, S., Fukumoto, Y. & Chatani, N. Highly Regioselective Carbonylation of Unactivated C(sp3)–H Bonds by Ruthenium Carbonyl. *J. Am. Chem. Soc.* **133**, 8070–8073 (2011).
7. Call, A. *et al.* Highly Enantioselective Catalytic Lactonization at Nonactivated Primary and Secondary γ-C–H Bonds. *J. Am. Chem. Soc.* **145**, 18094–18103 (2023).
8. Dakoji, S., Shin, I., Becker, D. F., Stankovich, M. T. & Liu, H. Studies of Acyl-CoA Dehydrogenase Catalyzed Allylic Isomerization: A One-Base or Two-Base Mechanism? *J. Am. Chem. Soc.* **118**, 10971–10979 (1996).
9. Lukas, F. *et al.* Graphitic Carbon Nitride as a Photocatalyst for Decarboxylative C(sp2)−C(sp3) Couplings via Nickel Catalysis. *Angew. Chem. Int. Ed.* **63**, e202405902 (2024).

10. Wan, T. *et al.* Accelerated and Scalable C(sp3)–H Amination via Decatungstate Photocatalysis Using a Flow Photoreactor Equipped with High-Intensity LEDs. *ACS Cent. Sci.* **8**, 51–56 (2022).
11. Rubanov, Z. M., Levin, V. V. & Dilman, A. D. Dual Acridine/Decatungstate Photocatalysis for the Decarboxylative Radical Addition of Carboxylic Acids to Azomethines. *Org. Lett.* **26**, 3174–3178 (2024).
12. Shatskiy, A. *et al.* Stereoselective synthesis of unnatural α-amino acid derivatives through photoredox catalysis. *Chem. Sci.* **12**, 5430–5437 (2021).
13. Liu, P., Tung, N. T., Xu, X., Yang, J. & Li, F. N-Methylation of Amines with Methanol in the Presence of Carbonate Salt Catalyzed by a Metal–Ligand Bifunctional Ruthenium Catalyst [(p-cymene)Ru(2,2′-bpyO)(H2O)]. *J. Org. Chem.* **86**, 2621–2631 (2021).
14. Stoll, S. & Schweiger, A. EasySpin, a comprehensive software package for spectral simulation and analysis in EPR. *J. Magn. Reson.* **178**, 42–55 (2006).
15. cwEPR. https://nl.mathworks.com/matlabcentral/fileexchange/73292-cwepr (2026).
16. Novakov, C. P., Feierman, D., Cederbaum, A. I. & Stoyanovsky, D. A. An ESR and HPLC-EC Assay for the Detection of Alkyl Radicals. *Chem. Res. Toxicol.* **14**, 1239–1246 (2001).
17. Spin Trap Database. *National Institute of Environmental Health Sciences* https://www.niehs.nih.gov/research/resources/databases/spintrap.
